# Catalogue of Tenebrionidae (Coleoptera) of North America

**DOI:** 10.3897/zookeys.728.20602

**Published:** 2018-01-15

**Authors:** Yves Bousquet, Donald B. Thomas, Patrice Bouchard, Aaron D. Smith, Rolf L. Aalbu, M. Andrew Johnston, Warren E. Steiner Jr.

**Affiliations:** 1 Canadian National Collection of Insects, Arachnids and Nematodes, Agriculture and Agri-Food Canada, Ottawa, Ontario, K1A 0C6, Canada; 2 USDA ARS, Cattle Fever Tick Research Laboratory, 22675 N Moorefield Rd, Edinburg, Texas, 78596, USA; 3 Northern Arizona University, Department of Biological Sciences, POB 5640, Flagstaff, Arizona, 86011, USA; 4 California Academy of Sciences, Department of Entomology, 55 Music Concourse Dr, Golden Gate Park, San Francisco, California, 94118, USA; 5 Arizona State University, School of Life Sciences, POB 874501, Tempe, Arizona, 85287, USA; 6 Smithsonian Institution, Department of Entomology, NHB 187, Washington, DC, 20013, USA

**Keywords:** North America, nomenclature, *Lepidocnemeplatia*, new genus, new replacement names

## Abstract

This catalogue includes all valid family-group (8 subfamilies, 52 tribes, 14 subtribes), genus-group (349 genera, 86 subgenera), and species-group names (2825 species, 215 subspecies) of darkling beetles (Coleoptera: Tenebrionidae) known to occur in North America^1^ and their available synonyms. Data on extant, subfossil and fossil taxa are given. For each name the author and year and page number of the description are provided, with additional information (e.g., type species for genus-group names, author of synonymies for invalid taxa) depending on the taxon rank.

Several new nomenclatural acts are included. One new genus, *Lepidocnemeplatia* Bousquet and Bouchard, is described. *Spelaebiosis* Bousquet and Bouchard [for *Ardoinia* Özdikmen, 2004], *Blapstinus marcuzzii* Aalbu [for *Blapstinus kulzeri* Marcuzzi, 1977], and *Hymenorus campbelli* Bouchard [for *Hymenorus oculatus* Doyen and Poinar, 1994] are proposed as new replacement names. Supporting evidence is provided for the conservation of usage of *Tarpela micans* (Fabricius, 1798) *nomen protectum* over *Tarpela vittata* (Olivier, 1793) *nomen oblitum*. The generic names *Psilomera* Motschulsky, 1870 [= *Stenomorpha* Solier, 1836], *Steneleodes* Blaisdell, 1909 [= *Xysta* Eschscholtz, 1829], *Ooconibius* Casey, 1895 and *Euconibius* Casey, 1895 [= *Conibius* LeConte, 1851] are new synonyms (valid names in square brackets). The following 127 new synonymies of species-group names, listed in their original combination, are proposed (valid names, in their current combination, placed in square brackets): *Bothrasida mucorea* Wilke, 1922 [= *Pelecyphorus guanajuatensis* (Champion, 1884)]; *Parasida zacualpanicola* Wilke, 1922 [= *Pelecyphorus asidoides* Solier, 1836]; *Stenosides kulzeri* Pallister, 1954, *Stenosides bisinuatus* Pallister, 1954, and *Parasida trisinuata* Pallister, 1954 [= *Pelecyphorus dispar* (Champion, 1892)]; *Asida favosa* Champion, 1884 and *Asida similata* Champion, 1884 [= *Pelecyphorus fallax* (Champion, 1884)]; *Ologlyptus bicarinatus* Champion, 1884 [= *Pelecyphorus indutus* (Champion, 1884)]; *Parasida laciniata* Casey, 1912 and *Parasida cristata* Pallister, 1954 [= *Pelecyphorus liratus* (LeConte, 1854)]; *Parasida esperanzae* Wilke, 1922 and *Parasida mixtecae* Wilke, 1922 [= *Pelecyphorus longipennis* (Champion, 1884)]; *Parasida tolucana* Casey, 1912 [= *Pelecyphorus scutellaris* (Champion, 1884)]; *Parasida purpusi* Wilke, 1922 [= *Pelecyphorus tristis* (Champion, 1884)]; *Astrotus nosodermoides* Champion, 1892 [= *Pelecyphorus erosus* (Champion, 1892)]; Astrotus seticornis var. humeralis Champion, 1884 [= *Pelecyphorus seticornis* (Champion, 1884)]; *Pactostoma breviuscula* Casey, 1912, *Pactostoma exoleta* Casey, 1912, *Pactostoma luteotecta* Casey, 1912, *Pactostoma monticola* Casey, 1912, *Pactostoma obtecta* Casey, 1912, and *Pactostoma sigillata* Casey, 1912 [=*Pelecyphorus anastomosis* (Say, 1824)]; *Ologlyptus canus* Champion, 1884 and *Ologlyptus sinuaticollis* Champion, 1884 [= *Pelecyphorus graciliformis* (Solier, 1836)]; *Gonasida elata reducta* Casey, 1912, *Gonasida elata prolixa* Casey, 1912, and *Gonasida aucta* Casey, 1912 [= *Philolithus elatus compar* (Casey, 1912)]; *Gonasida alaticollis* Casey, 1912 [= *Philolithus elatus difformis* (LeConte, 1854)]; *Gonasida gravida* Casey, 1912 [= *Philolithus elatus elatus* (LeConte, 1853)]; *Pelecyphorus aegrotus limbatus* Casey, 1912 [= *Philolithus aegrotus aegrotus* (LeConte, 1861)]; *Pelecyphorus corporalis* Casey, 1912, *Pelecyphorus reptans* Casey, 1912, *Pelecyphorus socer* Casey, 1912, *Pelecyphorus abscissus* Casey, 1912, *Pelecyphorus fumosus* Casey, 1912, *Pelecyphorus parvus* Casey, 1912, *Pelecyphorus morbillosus pacatus* Casey, 1912, *Pelecyphorus morbillosus sobrius* Casey, 1912, *Pelecyphorus piceus* Casey, 1912, *Pelecyphorus piceus crudelis* Casey, 1912, *Pelecyphorus snowi* Casey, 1912, and *Pelecyphorus subtenuis* Casey, 1912 [= *Philolithus morbillosus* (LeConte, 1858)]; *Bothrasida sanctae-agnae* Wilke, 1922 [= *Stenomorpha funesta* (Champion, 1884)]; *Asida flaccida* Horn, 1896 [= *Stenomorpha embaphionides* (Horn, 1894)]; *Asida angustula* Casey, 1890, *Stethasida stricta* Casey, 1912, *Stethasida muricatula languida* Casey, 1912, *Stethasida pertinax* Casey, 1912, *Stethasida socors* Casey, 1912, *Stethasida angustula inepta* Casey, 1912, *Stethasida tenax* Casey, 1912, and *Stethasida vegrandis* Casey, 1912 [= *Stenomorpha muricatula* (LeConte, 1851)]; *Stethasida obsoleta expansa* Casey, 1912, *Stethasida obsoleta opacella* Casey, 1912, *Stethasida brevipes* Casey, 1912, *Stethasida torpida* Casey, 1912, *Stethasida convergens* Casey, 1912, *Stethasida discreta* Casey, 1912, *Stethasida longula* Casey, 1912, *Stethasida adumbrata* Casey, 1912, *Stethasida occulta* Casey, 1912, *Stethasida tarsalis* Casey, 1912, *Stethasida unica* Casey, 1912, and *Pelecyphorus laevigatus* Papp, 1961 [= *Stenomorpha obsoleta* (LeConte, 1851)]; *Trichiasida eremica* Wilke, 1922 [= *Stenomorpha difficilis* (Champion, 1884)]; *Trichiasida lineatopilosa* Casey, 1912 [= *Stenomorpha hirsuta* (LeConte, 1851)]; *Trichiasida tenella* Casey, 1912 [= *Stenomorpha hispidula* (LeConte, 1851)]; *Trichiasida duplex* Casey, 1912 [= *Stenomorpha villosa* (Champion, 1884)]; *Alaudes squamosa* Blaisdell, 1919, *Alaudes testacea* Blaisdell, 1919, and *Alaudes fallax* Fall, 1928 [= *Alaudes singularis* Horn, 1870]; *Edrotes barrowsi* Dajoz, 1999 [=*Edrotes ventricosus* LeConte, 1851]; *Nyctoporis tetrica* Casey, 1907 and *Nyctoporis maura* Casey, 1907 [= *Nyctoporis aequicollis* Eschscholtz, 1831]; *Nyctoporis pullata* Casey, 1907 [= *Nyctoporis sponsa* Casey, 1907]; Eleodes tibialis forma oblonga Blaisdell, 1909 [= *Eleodes tibialis* Blaisdell, 1909]; *Eleodes* (*manni* var.) *variolosa* Blaisdell, 1917 [= *Eleodes constrictus* LeConte, 1858]; Eleodes cordata forma sublaevis Blaisdell, 1909, Eleodes cordata forma intermedia Blaisdell, 1909, Eleodes cordata forma oblonga Blaisdell, 1909, Eleodes cordata forma elongata Blaisdell, 1909, and *Eleodes* (*cordata* var.) *adulterina* Blaisdell, 1917 [= *Eleodes cordata* Eschscholtz, 1829]; Eleodes hornii var. monticula Blaisdell, 1918 and *Eleodes manni sierra* Blaisdell, 1925 [= *Eleodes fuchsii* Blaisdell, 1909]; Eleodes parvicollis var. squalida Blaisdell, 1918 [= *Eleodes parvicollis* Eschscholtz, 1829]; *Eleodes reflexicollis* Mannerheim, 1843 and Eleodes parvicollis forma farallonica Blaisdell, 1909 [= *Eleodes planata* Eschscholtz, 1829]; *Eleodes indentata* Blaisdell, 1935 [= *Eleodes rotundipennis* LeConte, 1857]; *Eleodes intricata* Mannerheim, 1843 [= *Eleodes scabrosa* Eschscholtz, 1829]; *Eleodes horni fenyesi* Blaisdell, 1925 [= *Eleodes tenebrosa* Horn, 1870]; Eleodes cordata var. horrida Blaisdell, 1918 [= *Eleodes tuberculata* Eschscholtz, 1829]; *Eleodes oblonga* Blaisdell, 1933 [= *Eleodes versatilis* Blaisdell, 1921]; *Eleodes dentipes marinae* Blaisdell, 1921 [= *Eleodes dentipes* Eschscholtz, 1829]; Eleodes carbonaria forma glabra Blaisdell, 1909 [= *Eleodes carbonaria carbonaria* (Say, 1824)]; Eleodes granosa forma fortis Blaisdell, 1909 [= *Eleodes granosa* LeConte, 1866]; Eleodes pilosa forma ordinata Blaisdell, 1909 [= *Eleodes pilosa* Horn, 1870]; *Trogloderus costatus pappi* Kulzer, 1960 [= *Trogloderus tuberculatus* Blaisdell, 1909]; *Trogloderus costatus mayhewi* Papp, 1961 [= *Trogloderus vandykei* La Rivers, 1946]; *Bolitophagus cristatus* Gosse, 1840 [= *Bolitotherus cornutus* (Fabricius, 1801)]; *Eleates explanatus* Casey, 1890 [= *Eleates depressus* (Randall, 1838)]; *Blapstinus sonorae* Casey, 1890 [= *Blapstinus brevicollis* LeConte, 1851]; *Blapstinus falli* Blaisdell, 1929 [= *Blapstinus castaneus* Casey, 1890]; *Blapstinus brunneus* Casey, 1890 and *Blapstinus coronadensis* Blaisdell, 1892 [=*Blapstinus histricus* Casey, 1890]; *Blapstinus hesperius* Casey, 1890 [=*Blapstinus intermixtus* Casey, 1890]; *Blapstinus cinerascens* Fall, 1929 [= *Blapstinus lecontei* Mulsant and Rey, 1859]; *Blapstinus niger* Casey, 1890 and *Blapstinus cribricollis* Casey, 1890 [= *Blapstinus pimalis* Casey, 1885]; *Blapstinus arenarius* Casey, 1890 [= *Blapstinus pratensis* LeConte, 1859]; *Blapstinus gregalis* Casey, 1890 [= *Blapstinus substriatus* Champion, 1885]; *Blapstinus hydropicus* Casey, 1890 [= *Blapstinus sulcatus* LeConte, 1851]; *Blapstinus hospes* Casey, 1890 [= *Blapstinus vestitus* LeConte, 1859]; *Notibius reflexus* Horn, 1894 [= *Conibius opacus* (LeConte, 1866)]; *Notibius affinis* Champion, 1885 [=*Conibius rugipes* (Champion, 1885)]; *Conibius parallelus* LeConte, 1851 [= *Conibius seriatus* LeConte, 1851]; *Nocibiotes rubripes* Casey, 1895 [=*Nocibiotes caudatus* Casey, 1895]; *Nocibiotes gracilis* Casey, 1895 and *Nocibiotes acutus* Casey, 1895 [=*Nocibiotes granulatus* (LeConte, 1851)]; *Conibius alternatus* Casey, 1890 [= *Tonibius sulcatus* (LeConte, 1851)]; *Pedinus suturalis* Say, 1824 [= *Alaetrinus minimus* (Palisot de Beauvois, 1817)]; *Menedrio longipennis* Motschulsky, 1872 [= *Tenebrio obscurus* Fabricius, 1792]; *Hymenophorus megops* Hatch, 1965 and *Telesicles magnus* Hatch, 1965 [= *Hymenorus sinuatus* Fall, 1931]; *Andrimus concolor* Casey, 1891 and *Andrimus convergens* Casey, 1891 [= *Andrimus murrayi* (LeConte, 1866)]; *Mycetochara marshalli* Campbell, 1978 [= *Mycetochara perplexata* Marshall, 1970]; *Phaleria globosa* LeConte, 1857 [= *Phaleria picta* Mannerheim, 1843]. The following subspecies of *Trogloderus costatus* LeConte, 1879 are given species rank: *Trogloderus nevadus* La Rivers, 1943, *Trogloderus tuberculatus* Blaisdell, 1909, and *Trogloderus vandykei* La Rivers, 1946. The following taxa, previously thought to be junior synonyms, are considered valid: *Amphidora* Eschscholtz, 1829; *Xysta* Eschscholtz, 1829; *Helops confluens* (Casey, 1924). Two new combinations are proposed: *Stenomorpha spinimana* (Champion, 1892) and *Stenomorpha tenebrosa* (Champion, 1892) [from the genus *Parasida* Casey, 1912]. The type species [placed in square brackets] of the following 12 genus-group taxa are designated for the first time: *Lagriola* Kirsch, 1874 [*Lagriola operosa* Kirsch, 1874]; *Locrodes* Casey, 1907 [*Emmenastus piceus* Casey, 1890]; *Falacer* Laporte, 1840 [*Acanthopus cupreus* Laporte, 1840 (= *Helops contractus* Palisot de Beauvois, 1812)]; *Blapylis* Horn, 1870 [*Eleodes cordata* Eschscholtz, 1829]; *Discogenia* LeConte, 1866 [*Eleodes scabricula* LeConte, 1858]; *Metablapylis* Blaisdell, 1909 [*Eleodes nigrina* LeConte, 1858]; *Steneleodes* Blaisdell, 1909 [*Eleodes longicollis* LeConte, 1851]; *Scaptes* Champion, 1886 [*Scaptes squamulatus* Champion, 1886 (= *Asida tropica* Kirsch, 1866)]; *Aspidius* Mulsant and Rey, 1859 [*Blaps punctata* Fabricius, 1792]; *Cryptozoon* Schaufuss, 1882 [*Cryptozoon civile* Schaufuss, 1882]; *Halophalerus* Crotch, 1874 [*Phaleria rotundata* LeConte, 1851]; *Dignamptus* LeConte, 1878 [*Dignamptus stenochinus* LeConte, 1878]. Two species previously known from South America [*Nilio lebasi* J. Thomson and *Platydema erotyloides* Chevrolat] are reported for the first time from North America.

## Abstract (es)

Este catálogo incluye todos los grupos válidos de nivel famili (8 subfamilias, 52 tribus, 14 subtribus), de nivel género (349 géneros, 86 subgéneros) y de nivel especie (2825 especies, 215 subespecies) de tenebriónidos (Coleoptera: Tenebrionidae) presentes en América del Norte y sus sinónimos disponibles. Se dan datos sobre los táxones existentes, subfósiles y fósiles. Para cada nombre, el autor, el año y el número de página de la descripción se complementan con información adicional dependiendo de la categoría del taxon (por ejemplo, especies tipo para nombres de nivel género, autor de sinonimias para táxones no válidos).

Se incluyen varios nuevos actos nomenclaturales. Un nuevo género, *Lepidocnemeplatia* Bousquet y Bouchard, se describe. *Spelaebiosis* Bousquet y Bouchard [para *Ardoinia* Özdikmen, 2004], *Blapstinus marcuzzii* Aalbu [para *Blapstinus* kulzeri Marcuzzi, 1977] e *Hymenorus campbelli* Bouchard [para *Hymenorus oculatus* Doyen y Poinar, 1994] se proponen como nuevos nombres de reemplazo. Se proporciona evidencia de uso para la conservación de *Tarpela micans* (Fabricius, 1798) *nomen protectum* sobre *Tarpela vittata* (Olivier, 1793) *nomen oblitum*. Los nombres genéricos *Psilomera* Motschulsky, 1870 [= *Stenomorpha* Solier, 1836], *Steneleodes* Blaisdell, 1909 [= *Xysta* Eschscholtz, 1829], *Ooconibius* Casey, 1895 y *Euconibius* Casey, 1895 [= *Conibius* LeConte, 1851] sonnuevos sinónimos (nombres válidos en corchetes). Se proponen las siguientes 127 nuevas sinonimias de nombres de nivel especie (ver lista en el «Resumen»), enumerados en su combinación original (nombres válidos, listados en su combinación actual, colocados entre corchetes). Las siguientes subespecies de *Trogloderus costatus* LeConte, 1879 reciben categoría de especie: *Trogloderus nevadus* La Rivers, 1943, *Trogloderus tuberculatus* Blaisdell, 1909 y *Trogloderus vandykei* La Rivers, 1946. Los siguientes táxones, que anteriormente se consideraban sinónimos más modernos, se consideran válidos: *Amphidora* Eschscholtz, 1829, *Xysta* Eschscholtz, 1829, *Helops confluens* (Casey, 1924). Se proponen dos nuevas combinaciones: *Stenomorpha spinimana* (Champion, 1892) y *Stenomorpha tenebrosa* (Champion, 1892) [del género *Parasida* Casey, 1912]. Las especies tipo [colocadas entre corchetes] de los siguientes 12 táxones del nivel génerosse designan por primera vez: *Lagriola* Kirsch, 1874 [*Lagriola operosa* Kirsch, 1874]; *Locrodes* Casey, 1907 [*Emmenastus piceus* Casey, 1890]; *Falacer* Laporte, 1840 [*Acanthopus cupreus* Laporte, 1840 (= *Helops contractus* Palisot de Beauvois, 1812)]; *Blapylis* Horn, 1870 [*Eleodes cordata* Eschscholtz, 1829]; *Discogenia* LeConte, 1866 [*Eleodes scabricula* LeConte, 1858]; *Metablapylis* Blaisdell, 1909 [*Eleodes nigrina* LeConte, 1858]; *Steneleodes* Blaisdell, 1909 [*Eleodes longicollis* LeConte, 1851]; *Scaptes* Champion, 1886 [*Scaptes squamulatus* Champion, 1886 (= *Asida tropica* Kirsch, 1866)]; *Aspidius* Mulsant y Rey, 1859 [*Blaps punctata* Fabricius, 1792]; *Cryptozoon* Schaufuss, 1882 [*Cryptozoon civile* Schaufuss, 1882]; *Halophalerus* Crotch, 1874 [*Phaleria rotundata* LeConte, 1851]; *Dignamptus* LeConte, 1878 [*Dignamptus stenochinus* LeConte, 1878]. Dos especies previamente conocidas de América del Sur [*Nilio lebasi* J. Thomson y *Platydema erotyloides* Chevrolat] se mencionan por primera vez de América del Norte.

## Abstract (fr)

Ce catalogue liste tous les noms de niveau famille (8 sous-familles, 52 tribus, 14 sous-tribus), genre (349 genres, 86 sous-genres), et espèce (2825 espèces, 215 sous-espèces) de Tenebrionidae (Coleoptera:) recensés en Amérique du Nord ainsi que leurs synonymes disponibles. Le catalogue inclut les taxons actuels, subfossiles et fossiles. L’auteur ainsi que l’année et la pagination de la description sont inclus pour chaque nom scientifique et, pour certains de ces noms, des renseignements additionnels (par exemple, l’espèce-type pour les noms de niveau genre, l’auteur de la synonymie des noms invalides des niveaux genre et espèce).

Plusieurs nouveaux actes nomenclaturaux sont proposés. On décrit un nouveau genre, *Lepidocnemeplatia* Bousquet et Bouchard. *Spelaebiosis* Bousquet et Bouchard [pour *Ardoinia* Özdikmen, 2004], *Blapstinus marcuzzii* Aalbu [pour *Blapstinus kulzeri* Marcuzzi, 1977] et *Hymenorus campbelli* Bouchard [pour *Hymenorus oculatus* Doyen and Poinar, 1994] sont des nouveaux noms de remplacement. On fournit les évidences nécessaires pour conserver l’usage actuel du nom *Tarpela micans* (Fabricius, 1798) *nomen protectum* sur celui de *Tarpela vittata* (Olivier, 1793) *nomen oblitum*. Les noms génériques *Psilomera* Motschulsky, 1870 [= *Stenomorpha* Solier, 1836], *Steneleodes* Blaisdell, 1909 [= *Xysta* Eschscholtz, 1829], *Ooconibius* Casey, 1895 et *Euconibius* Casey, 1895 [= *Conibius* LeConte, 1851] sont des nouveaux synonymes (noms valides placés entre crochets). On propose 127 nouveaux synonymes de noms de niveau espèce (voir la liste dans « Abstract »), listés par leur combinaison originelle (noms valides, listés par leur combinaison actuelle, placés entre crochets). On élève au rang d’espèce les sous-espèces suivantes de *Trogloderus costatus* LeConte, 1879: *Trogloderus nevadus* La Rivers, 1943, *Trogloderus tuberculatus* Blaisdell, 1909, et *Trogloderus vandykei* La Rivers, 1946. On redonne le statut de taxons valides aux entités suivantes considérées à tort comme invalides: *Amphidora* Eschscholtz, 1829, *Xysta* Eschscholtz, 1829, et *Helops confluens* (Casey, 1924). On propose les combinaisons nouvelles suivantes: *Stenomorpha spinimana* (Champion, 1892) et *Stenomorpha tenebrosa* (Champion, 1892) [auparavant placés dans le genre *Parasida* Casey, 1912]. On désigne pour la première fois l’espèce type [placée entre crochets] des 12 noms suivants de niveau genre: *Lagriola* Kirsch, 1874 [*Lagriola operosa* Kirsch, 1874]; *Locrodes* Casey, 1907 [*Emmenastus piceus* Casey, 1890]; *Falacer* Laporte, 1840 [*Acanthopus cupreus* Laporte, 1840 (= *Helops contractus* Palisot de Beauvois, 1812)]; *Blapylis* Horn, 1870 [*Eleodes cordata* Eschscholtz, 1829]; *Discogenia* LeConte, 1866 [*Eleodes scabricula* LeConte, 1858]; *Metablapylis* Blaisdell, 1909 [*Eleodes nigrina* LeConte, 1858]; *Steneleodes* Blaisdell, 1909 [*Eleodes longicollis* LeConte, 1851]; *Scaptes* Champion, 1886 [*Scaptes squamulatus* Champion, 1886 (= *Asida tropica* Kirsch, 1866)]; *Aspidius* Mulsant and Rey, 1859 [*Blaps punctata* Fabricius, 1792]; *Cryptozoon* Schaufuss, 1882 [*Cryptozoon civile* Schaufuss, 1882]; *Halophalerus* Crotch, 1874 [*Phaleria rotundata* LeConte, 1851]; *Dignamptus* LeConte, 1878 [*Dignamptus stenochinus* LeConte, 1878]. Deux espèces, auparavant documentées seulement de l’Amérique du Sud [*Nilio lebasi* J. Thomson and *Platydema erotyloides* Chevrolat], sont rapportées pour la première fois d’Amérique du Nord.

## Introduction

Darkling beetles (Coleoptera: Tenebrionidae) form a species-rich and morphologically diverse family with approximately 2300 genera and 20000 species worldwide (Matthews et al. 2010), and many more taxa to be described. The first thorough classification of the Tenebrionidae was provided by Lacordaire (1859) and was based entirely on the external morphology of adults. With relatively few exceptions, his classification schema was followed by subsequent workers for approximately 100 years (Watt 1967). The family classification was eventually reviewed using morphological characters of immature stages (Watt 1975) although significant changes did not appear until the first comprehensive investigation of adult internal structures including defense glands, female ovipositor, and female genital tube (Tschinkel and Doyen 1980, Doyen and Tschinkel 1982). The first higher-level phylogeny of the family based on molecular data was published only recently (Kergoat et al. 2014). As a result of these comparative and phylogenetic studies, several taxa previously treated as separate families (e.g., Lagriidae, Alleculidae, Nilionidae) are now included within Tenebrionidae. Additionally, many taxa previously included in Tenebrionidae are now classified in other families (see Table 1 for North American genera, Aalbu (2006) for worldwide taxa).

**Table 1.** List of North American genera previously included in Tenebrionidae but currently classified in another family.

Genus	Current placement
*Boros* Herbst, 1797	Boridae
*Dacoderus* LeConte, 1858	Salpingidae
*Megazopherus* Casey, 1907	Zopheridae
*Meralius* Casey, 1907	Zopheridae
*Noserodes* Casey, 1907	Zopheridae
*Noserus* LeConte, 1862	Zopheridae
*Nosoderma* Solier, 1841	Zopheridae
*Phellopsis* LeConte, 1862	Zopheridae
*Phloeodes* LeConte, 1862	Zopheridae
*Sesaspis* Casey, 1907	Zopheridae
*Usechus* Motschulsky, 1845	Zopheridae
*Verodes* Casey, 1907	Zopheridae
*Zopherinus* Casey, 1907	Zopheridae
*Zopherodes* Casey, 1907	Zopheridae
*Zopherus* Laporte, 1840	Zopheridae

The aim of this work is to synthesize available taxonomic, nomenclatural, and distributional information for all darkling beetles known from North America.

## Methods

### Nomenclatural data

All nomenclaturally available family-, genus- and species-group names are included. Extant taxa and subfossils from the Pleistocene (see Doyen and Miller 1980, Doyen and Poinar 1994) are given in the main catalogue. Impression fossils from major North American deposits are listed in Appendix 1, although the taxonomic assignment of these often-fragmentary fossils needs to be confirmed. Fossil species described from amber are listed in Appendix 2. Taxa incorrectly recorded from North America are given in Appendix 3. Subfamilies are listed in a phylogenetic framework but valid tribal, generic, and specific names are given in alphabetic order; listings of all invalid names are chronological.

The author and year and page number of the original description are provided for each scientific name. The type genus for each family-group name and the type species and type fixation for each genus-group name are included. The reference in which a given generic or specific name is first placed in synonymy with the current valid name is listed. Type-species designations in Lucas (1920) were accepted when a single species was listed under a particular genus-group name (see Alonso-Zarazaga and Lyal 2009, Bousquet et al. 2015). For genera with valid subgenera, synonyms are given under the nominotypical subgenus when relevant. Every species-group name are listed in its original combination, as given in the publication even if the agreement in gender with the generic name is incorrect. In cases where a species-group name older than the one currently recognized as valid is available but of doubtful application (e.g., *Latridius pubescens* Say, 1826), we have retained usage of the younger, accepted name as valid and treated the older name as *nomen dubium*. The synonymy list for adventive species focuses primarily on names used in the North American literature, other sources (e.g., Löbl et al. 2008a) can be consulted for data on all invalid names.

The classification used follows Bouchard et al. (2011) but also includes corrections and subsequent additions.

The gender of all valid genera listed in the catalogue has been determined following the provision of Article 30 (ICZN 1999) and indicated after the name using the initials M [Masculine], F [Feminine], and N [Neuter]. Therefore, the gender of the generic names *Liodema*, *Platydema*, and *Scaphidema* is herein treated as feminine following Article 30.1.3 (ICZN 1999; see Kerzhner (2003) and Löbl and Smetana (2010: 34) for further comments); the ending is derived from the Greek “*demas* (body silhouette).” The gender of *Alaudes* is treated as masculine following Article 30.2.4 (ICZN 1999). The gender of *Eleodes*, originally treated as feminine by Eschscholtz (1829), was changed to masculine by Somerby and Doyen (1976) and has been followed subsequently in the literature. However, *Eleodes* is feminine following Article 30.1.4.4 (ICZN 1999) which says that “a compound genus-group name ending in the suffix ... -*odes* is to be treated as masculine unless its author, when establishing the name, stated that it had another gender or treated it as such by combining it with an adjectival species-group name in another gender form.”

If necessary, the ending of all valid species-group names has been modified according to the gender of the generic name with which the species is currently combined. All specific names that are nouns in apposition need not agree in gender with the generic name and retain their original endings.

The author(s) of every new nomenclatural act proposed in the catalogue is given in square brackets (e.g., “[ADS]”) except for the first typification of genus-group names. One of these new acts needs further development. The genus-group taxon *Lepidocnemeplatia* was proposed by Kaszab (1938: 80) as a subgenus of *Cnemeplatia* Costa, 1847 to include two species, *Cnemeplatia laticollis* Champion, 1885 and *C. sericea* Horn, 1870. Unfortunately, Kaszab did not designate a type species for his new genus and therefore the name is unavailable from that date (ICZN 1999: Article 13.3). Löbl and Merkl (2003: 245) designated *C. sericea* Horn, 1870 as type species of *Lepidocnemeplatia*, the first typification for the taxon, and subsequently Löbl et al. (2008a: 140) credited authorship of the name to Löbl and Merkl (2003). However, since Löbl and Merkl (2003: 245) failed to indicate that they were establishing a new nominal taxon, a mandatory requirement for all new names published after 1999 (ICZN 1999: Article 16.1), the name cannot be attributed to them and is still a *nomen nudum*. In order to make the name available we here proposed the generic name *Lepidocnemeplatia*^2^ Bousquet and Bouchard, new genus; type species (here designated): *Cnemeplatia sericea* Horn, 1870. The reader is referred to Kaszab (1938: 79–80, couplet 1" of his key) for a description of the character states that differentiate the taxon (ICZN 1999: Article 13.1.2). A summary of all new nomenclatural acts is available in the Abstract.

### Distributional data

This catalogue documents all species of Tenebrionidae from Greenland, Alaska, and Canada south to Panama, and also includes islands of the West Indies. Records from the Netherlands Antilles (consisting of several islands in the West Indies), the Venezuelan island Margarita, and Trinidad and Tobago off the northeast coast of Venezuela are not included since the fauna of these islands is more closely affiliated with the South American fauna. When known, the states (for Mexico and continental United States) and the provinces and territories (for Canada) are listed in parentheses for each species. For West Indian records, we give the political unit names for species found in the Lucayan Archipelago and Greater Antilles, occasionally with specific islands in parenthesis; political unit names are not provided for species occurring in the Lesser Antilles (LAN) and Virgin Islands (VIS), though sometimes we include specific islands in parentheses, especially when the species is known from only one or two islands. Further details regarding the West Indies geographical units can be found in Ivie and Hart (2016: Table 1).

Many Mexican state records in this catalogue came from localities listed by Champion in volume IV, parts 1 and 2, of the *Biologia Centrali-Americana* [1884–1893] and the gazetteer of Selander and Vaurie (1962) was used consistently to associate localities with states (including the federal district).

Amongst the geographical units used (see list below), HIS (for Hispaniola) is used when only the island record is known and LC (for Lower California) when only the peninsula record is known. South America (SA) is placed at the end of a species record list to indicate that the species extends into South America. Distributional records listed in square brackets (e.g., “[NM]”) are considered doubtful. South American species introduced accidentally into North America are indicated with a subscript “_i_” beside the country record (i.e., “USA_i_”).

### Bibliographic data

References are provided for all scientific names included if a page number (related to the description of the taxon) is provided after the year of publication. Based on evidence previously published (see Bousquet 2016a: 211, 265), the dates of publication of Germar’s *Coleopterorum species novae* dated 1824 on its title page, and of Hope’s *The Coleopterist’s manual, part the third*, dated 1840 on its title page, are given as 1823 and 1841 respectively.

As discussed by Bousquet (2016b), Mäklin's “Monographie der Gattung *Strongylium*” was published in 1867, not 1864 as given by several authors.

### List of acronyms used for geographic units^3^

**BAH** Bahamas

**BEL** Belize

**BER** Bermuda

**CAN** Canada [AB: Alberta; BC: British Columbia; MB: Manitoba; NB: New Brunswick; NF: Newfoundland and Labrador; NS: Nova Scotia; NT: Northwest Territories; NU: Nunavut; ON: Ontario; PE: Prince Edward Island; QC: Quebec; SK: Saskatchewan; YT: Yukon Territory]

**CAY** Cayman Islands

**CRI** Costa Rica

**CUB** Cuba

**DOM** Dominican Republic

**GRE** Greenland

**GUA** Guatemala

**HAI** Haiti

**HIS** Hispaniola

**HON** Honduras

**JAM** Jamaica

**LAN** Lesser Antilles (including among others Anguilla, Antigua and Barbuda, Montserrat, Guadeloupe, Dominica, Martinique, Saint Lucia, Grenada, Barbados)

**LC** Lower California

**MEX** Mexico [AG: Aguascalientes; BC: Baja California; BS: Baja California Sur; CA: Campeche; CH: Chihuahua; CI: Chiapas; CL: Colima; CO: Coahuila; DU: Durango; FD: Federal District; GE: Guerrero; GU: Guanajuato; HI: Hidalgo; JA: Jalisco; ME: México; MI: Michoacán; MO: Morelos; NA: Nayarit; NL: Nuevo León; OA: Oaxaca; PU: Puebla; QR: Quintana Roo; QU: Querétaro; SI: Sinaloa; SL: San Luis Potosí; SO: Sonora; TA: Tamaulipas; TB: Tabasco; TL: Tlaxcala; VE: Veracruz; YU: Yucatán; ZA: Zacatecas]

**NIC** Nicaragua

**PAN** Panama

**PRI** Puerto Rico [includes Vieques]

**SAL** El Salvador

**TUR** Turks and Caicos Islands

**USA** United States of America [AK: Alaska; AL: Alabama; AR: Arkansas; AZ: Arizona; CA: California; CO: Colorado; CT: Connecticut; DC: District of Columbia; DE: Delaware; FL: Florida; GA: Georgia; IA: Iowa; ID: Idaho; IL: Illinois; IN: Indiana; KS: Kansas; KY: Kentucky; LA: Louisiana; MA: Massachusetts; MD: Maryland; ME: Maine; MI: Michigan; MN: Minnesota; MO: Missouri; MS: Mississippi; MT: Montana; NC: North Carolina; ND: North Dakota; NE: Nebraska; NH: New Hampshire; NJ: New Jersey; NM: New Mexico; NV: Nevada; NY: New York; OH: Ohio; OK: Oklahoma; OR: Oregon; PA: Pennsylvania; RI: Rhode Island; SC: South Carolina; SD: South Dakota; TN: Tennessee; TX: Texas; UT: Utah; VA: Virginia; VT: Vermont; WA: Washington; WI: Wisconsin; WV: West Virginia; WY: Wyoming]

**VIS** Virgin Islands [includes US Virgin Islands and British Virgin Islands: Saint Thomas, Saint Croix]

**SA** South America

## Results

### Overall diversity

A total of 128 valid and invalid family-group, 612 genus-group, and 4065 species-group taxa (excluding fossils listed in Appendices 1, 2) are listed in this catalogue. The subfamily Pimeliinae is the most diverse with 908 valid species-group taxa, followed by the Tenebrioninae (808), Alleculinae (418), Stenochiinae (361), Diaperinae (260), Lagriinae (256), Phrenapatinae (22), and Nilioninae (6). Thirty-seven species in three subfamilies are adventive (Table 2), several of which are pests of stored grain products. Mexico and the continental United States of America are by far the most diverse political regions with 1224 and 1230 valid species-group taxa respectively, while at the other extreme Greenland and Bermuda only have two each (Table 3).

**Table 2.** List of adventive species documented in North America. Data on the origin, date of first detection in North America, and microhabitat associations in nature are given for each species as far as known. * = pest of stored grain products.

Species	Origin	Date of detection	Microhabitat	Placement
*Alphitobius diaperinus* (Panzer, 1797)	Africa	<1866	*Animal nests, caves, guano	Tenebrioninae : Alphitobiini
*Alphitobius laevigatus* (Fabricius, 1781)	Africa	<1866	*Animal nests, caves, guano	Tenebrioninae : Alphitobiini
*Alphitophagus bifasciatus* (Say, 1824)	Europe, probably	<1824	*Animal nests, caves, guano	Diaperinae : Diaperini: Adelinina
*Anchophthalmops menouxi* (Mulsant and Rey, 1853)	Africa; probably not established	<1870	Leaf litter on sandy soil, probably	Tenebrioninae : Pedinini: Platynotina
*Blaps (Blaps) lethifera lethifera* Marsham, 1802	Europe	<1889	Under rocks, wood, in caves	Tenebrioninae : Blaptini : Blaptina
*Blaps (Blaps) mucronata* Latreille, 1804	Europe	<1889	Under rocks, wood, in caves	Tenebrioninae : Blaptini : Blaptina
*Ceropria induta* (Wiedemann, 1819)	Asia	1998	Polypore fungi, rotten wood	Diaperinae : Diaperini: Diaperina
Ellipsodes (Anthrenopsis) ziczac (Motschulsky, 1873)	Asia, probably via Antilles	1891	Under leaf litter on sandy soil	Diaperinae : Crypticini
Gnatocerus (Echocerus) maxillosus (Fabricius, 1801)	Asia, probably via Europe	<1866	*Animal nests, caves, under bark	Diaperinae : Diaperini: Adelinina
Gnatocerus (Gnatocerus) cornutus (Fabricius, 1798)	Asia, probably via Europe	<1866	*Animal nests, caves, under bark	Diaperinae : Diaperini: Adelinina
*Gondwanocrypticus pictus* (Gebien, 1928)	South America	1954	Under leaf litter near ant nests	Diaperinae : Crypticini
*Gondwanocrypticus platensis* (Fairmaire, 1884)	South America	1929	Under leaf litter near ant nests	Diaperinae : Crypticini
Gonocephalum (Gonocephalum) sericeum (Baudi di Selve, 1875)	Northwest Africa + Arabian Peninsula	1980	Under leaf litter, wood, rocks	Tenebrioninae : Opatrini: Opatrina
*Latheticus oryzae* Waterhouse, 1880	Old World	1908	*Animal nests, caves, under bark	Tenebrioninae : Triboliini
*Leichenum canaliculatum variegatum* (Klug, 1833)	Madagascar	1906	Under leaf litter on sandy soil	Tenebrioninae : Pedinini: Leichenina
*Lyphia tetraphylla* (Fairmaire, 1857)	Europe	<1902	In dead wood, other insect burrows	Tenebrioninae : Triboliini
*Myrmechixenus lathridioides* Crotch, 1873	Europe	<1883	Under leaf litter, in soil	Diaperina : Myrmechixenini
*Opatroides punctulatus* Brulle, 1832	Middle East	2003	Under leaf litter, wood, rocks	Tenebrioninae : Opatrini: Opatrina
*Palorus cerylonoides* (Pascoe, 1863)	Indo-Malayan, probably	2004	Under bark dry wood, plant debris	Tenebrioninae : Palorini
*Palorus genalis* Blair, 1930	Old World	1937	Under bark dry wood, plant debris	Tenebrioninae : Palorini
*Palorus ratzeburgii* (Wissmann, 1848)	North Africa, probably	<1897	*Under bark dry wood, plant debris	Tenebrioninae : Palorini
*Palorus subdepressus* (Wollaston, 1864)	Africa, probably	<1882	*Under bark dry wood, plant debris	Tenebrioninae : Palorini
*Pentaphyllus testaceus* (Hellwig, 1792)	Europe	2005	Polypore fungi, under bark	Diaperinae : Diaperini: Diaperina
*Platydema woldai* Triplehorn and Phillips, 1998	Central America, probably	1964	With orchid plants; at lights in forest	Diaperinae : Diaperini: Diaperina
*Plesiophthalmus spectabilis* Harold, 1875	Asia; probably not established	2013	Rotten wood	Tenebrioninae : Amarygmini
*Poecilocrypticus formicophilus* Gebien, 1928	South America	1978	Under leaf litter near ant nests	Diaperinae : Crypticini
*Strongylium cultellatum* Maklin, 1867	Asia	2010	Dead standing wood	Stenochiinae : Stenochiini
*Tenebrio molitor* Linnaeus, 1758	Africa, probably	<1837	*Animal nests, caves	Tenebrioninae : Tenebrionini
*Tenebrio obscurus* Fabricius, 1792	Africa, probably	<1869	*Animal nests, caves	Tenebrioninae : Tenebrionini
*Trachyscelis aphodioides* Latreille, 1809	Europe	<1846	Under plant debris on beach sand	Diaperinae : Trachyscelini
Tribolium (Tribolium) castaneum (Herbst, 1797)	Africa, probably	<1866	*Under bark dry wood, plant debris	Tenebrioninae : Triboliini
Tribolium (Tribolium) confusum Jacquelin du Val, 1862	Africa, probably	<1893	*Under bark dry wood, plant debris	Tenebrioninae : Triboliini
Tribolium (Tribolium) destructor Uyttenboogaart, 1934	Africa	<1948	*Under bark dry wood, plant debris	Tenebrioninae : Triboliini
Tribolium (Tribolium) madens (Charpentier, 1825)	Africa, probably	<1866	*Under bark dry wood, plant debris	Tenebrioninae : Triboliini
*Tyrtaeus dobsoni* Hinton, 1947	Unknown; probably via Europe	2002	Under bark and in dead wood	Diaperinae : Gnathidiini : Anopidiina
*Ulomina carinata* Baudi di Selve, 1876	Asia	1952	Under bark dry wood, plant debris	Tenebrioninae : Palorini
*Ulomoides ocularis* (Casey, 1891)	Asia	<1891	Dry pods of *Tamarindus* L.	Diaperinae : Diaperini: Diaperina

**Table 3.** Number of valid species-group taxa by political region. Data excludes impression and amber fossils.

Political region	Lagriinae	Nilioninae	Phrenapatinae	Pimeliinae	Tenebrioninae	Alleculinae	Diaperinae	Stenochiinae	Total
BAH: Bahamas	0	0	0	8	22	14	25	1	70
BEL: Belize	20	0	3	2	20	5	32	28	110
BER: Bermuda	0	0	0	0	1	0	1	0	2
CAN: Canada	7	0	2	8	58	28	24	10	137
CAY: Cayman Islands	1	0	0	1	14	4	16	0	36
CRI: Costa Rica	44	0	6	12	27	23	40	44	196
CUB: Cuba	2	0	0	23	57	25	37	31	175
DOM: Dominican Republic	0	0	0	2	18	13	19	19	71
GRE: Greenland	0	0	0	0	2	0	0	0	2
GUA: Guatemala	60	2	4	20	70	57	66	71	350
HAI: Haiti	2	0	0	3	10	8	13	11	47
HON: Honduras	1	0	0	8	10	5	16	3	43
JAM: Jamaica	2	0	1	3	19	11	17	0	53
LAN: Lesser Antilles	11	0	3	6	50	11	31	18	130
MEX: Mexico	101	1	4	392	348	135	129	115	1225
NIC: Nicaragua	41	1	3	16	49	19	36	62	227
PAN: Panama	70	5	8	13	51	41	60	87	335
PRI: Puerto Rico	3	0	1	2	22	4	19	10	61
SAL: El Salvador	0	0	0	2	4	4	8	0	18
TUR: Turks and Caicos Islands	0	0	0	0	1	2	2	0	5
USA: United States of America	38	0	2	546	362	154	79	46	1227
VIS: Virgin Islands	0	0	0	0	3	2	1	1	7

**Figure 1.**
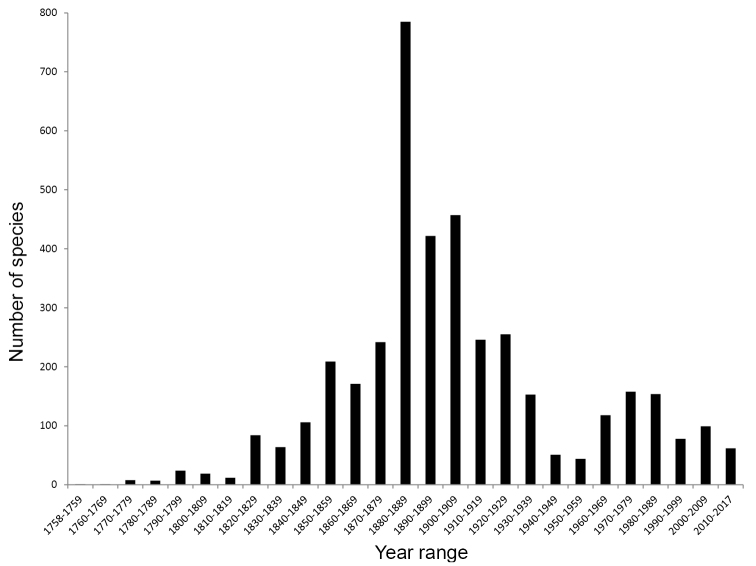
Number of North American species-group taxa described over time, by decade. Data excludes adventive species as well as impression and amber fossils.

**Figure 2.**
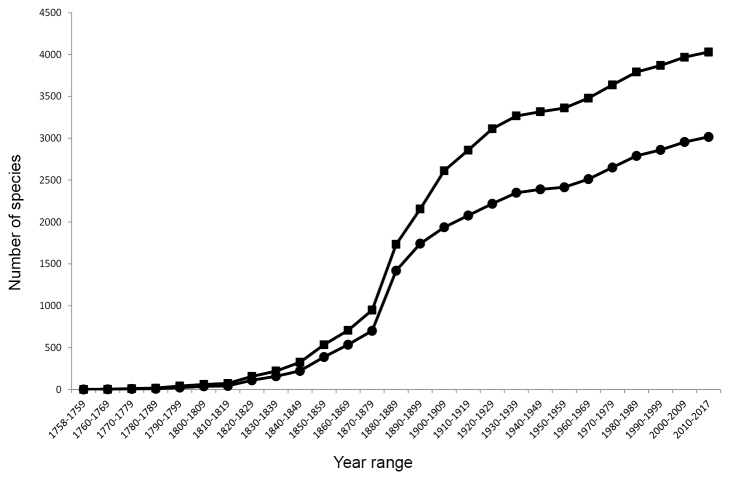
Cumulative number of North American species-group taxa described over time, by decade. Data excludes adventive species as well as impression and amber fossils. Line with square marker = total available taxa, line with circular marker = currently valid taxa.

A significant proportion of new species-group taxa (41%) were described between the years 1880 and 1910 (Fig. 1). A noticeable decrease in the number of new species-group taxa proposed occurred between 1940–1960, with a small resurgence since then (at a rate of approximately 100 new taxa per decade, see Figs 1, 2). Over 3000 North American species-group taxa are currently recognized as valid (Fig. 2), approximately 15% of the world fauna.

### Significant contributions (see Table 4)

The British entomologist George Charles Champion [b. 1851, d. 1927], working on the fauna of Mexico and Central America, proposed the highest number of new tenebrionid taxa found on the continent (83 genus-group, 906 species-group taxa) followed by the American Thomas Lincoln Casey [b. 1857, d. 1925], working mainly on the fauna of the United States of America (80 genus-group, 792 species-group taxa). Frank Ellsworth Blaisdell [b. 1862, d. 1947] (338 species-group taxa), John Lawrence LeConte [b. 1825, d. 1883] (270 species-group taxa), George Henry Horn [b. 1840, d. 1897] (133 species-group taxa), and John Milton Campbell [b. 1935] (103 species-group taxa) also contributed significantly to describing the North American darkling beetle fauna.

**Table 4.** Significant contributions to the description of new North American Tenebrionidae genus-group and species-group taxa (list includes the top ten contributors in each category). Data excludes adventive taxa as well as impression and amber fossils. * = given in parentheses is the country where the person produced taxonomic works, when different from the country of origin.

Author	Country of origin*	New genus-group names	New species-group names
Blaisdell, Frank Ellsworth	USA	20	338
Campbell, John Milton	USA (Canada)	10	103
Casey, Thomas Lincoln	USA	80	792
Champion, George Charles	United Kingdom	83	906
Dejean, Pierre Francois Marie Auguste	France	16	0
Doyen, John Thomas	USA	5	79
Fall, Henry Clinton	USA	1	66
Horn, George Henry	USA	20	133
Laporte, Francois Louis (Comte de Castelnau)	United Kingdom (France)	12	40
LeConte, John Lawrence	USA	56	277
Maklin, Friedrich [Fredrik] Wilhelm	Finland	3	66
Marcuzzi, Giorgio	Italy	7	84
Motschulsky, Victor de	Russia	12	24
Mulsant, Martial Etienne	France	13	18
Pascoe, Francis Polkinghorne	United Kingdom	12	4
Solier, Antoine Joseph Jean	France	17	35
Triplehorn, Charles Albert	USA	4	86

## Catalogue of Tenebrionidae (Coleoptera) of North America

**Family TENEBRIONIDAE Latreille, 1802**

Tenebrionites Latreille, 1802: 165. Type genus: *Tenebrio* Linnaeus, 1758.

**Subfamily LAGRIINAE Latreille, 1825**

Lachnaedes Billberg, 1820a: 34. Type genus: *Lachna* Billberg, 1820 (= *Lagria* Fabricius, 1775). *Nomen oblitum* (see Favret and Bouchard 2016).

Lagriariae Latreille, 1825: 381. Type genus: *Lagria* Fabricius, 1775. Note. Use of younger family-group name conserved over Lachnina Billberg, 1820 (ICZN 1999: Article 40.2) (see Bouchard et al. 2005).

**Tribe Belopini Reitter, 1917**

Belopinae Reitter, 1917: 59. Type genus: *Belopus* Gebien, 1911.

**Genus *Adelonia* Laporte, 1840** [F]

*Adelonia* Laporte, 1840: 221. Type species: *Uloma filiformis* Laporte, 1840, monotypy.

*Merotemnus* Horn, 1870: 367. Type species: *Merotemnus elongatus* Horn, 1870 (= *Uloma filiformis* Laporte, 1840), monotypy. Synonymy: Spilman (1961a: 49).

*Rhacius* Champion, 1885: 120. Type species: *Rhacius sulcatulus* Champion, 1885, subsequent designation (Gebien 1941: 805). Synonymy: Spilman (1961a: 50).

***Adelonia filiformis* (Laporte, 1840)** MEX (BC BS)

*Uloma filiformis* Laporte, 1840: 221.

*Merotemnus elongatus* Horn, 1870: 367. Synonymy: Gebien (1908b: 160).

***Adelonia insularis* Doyen, 1983** MEX (NA [Islas Marías])

*Adelonia insularis* Doyen, 1983: 85.

***Adelonia quadricollis* (Champion, 1885)** GUA BEL PAN / SA

*Rhacius quadricollis* Champion, 1885: 121.

***Adelonia sulcatula* (Champion, 1885)** ^4^ USA (TX) MEX (GE JA MO OA PU SO VE YU) GUA HON NIC CRI PAN / CUB CAY JAM / SA

*Rhacius sulcatulus* Champion, 1885: 121.

**Genus *Rhypasma* Pascoe, 1862** [N]

*Rhypasma* Pascoe, 1862: 325. Type species: *Rhypasma pusillum* Pascoe, 1862, monotypy.

*Derosimus* Fairmaire, 1904: 62. Type species: *Derosimus quadricollis* Fairmaire, 1904, monotypy. Synonymy: Blair (1935: 104).

***Rhypasma costaricense* Marcuzzi, 1976** CRI

*Rhypasma costaricense* Marcuzzi, 1976: 119.

***Rhypasma haitianum* Marcuzzi, 1954** CUB HAI

*Rhypasma haitianum* Marcuzzi, 1954a: 82.

***Rhypasma livae* Ferrer and Ødegaard, 2005** NIC PAN

*Rhypasma livae* Ferrer and Ødegaard, 2005: 635.

**Tribe Eschatoporiini Blaisdell, 1906**

Eschatoporini Blaisdell, 1906: 78. Type genus: *Eschatoporis* Blaisdell, 1906.

**Genus *Eschatoporis* Blaisdell, 1906** [M]

*Eschatoporis* Blaisdell, 1906: 76. Type species: *Eschatoporis nunenmacheri* Blaisdell, 1906, monotypy.

***Eschatoporis nunenmacheri* Blaisdell, 1906** USA (CA)

*Eschatoporis nunenmacheri* Blaisdell, 1906: 78.

***Eschatoporis styx* Aalbu, Kanda and Smith, 2017** USA (CA)

*Eschatoporis styx* Aalbu, Kanda and Smith, 2017: 140.

**Tribe Goniaderini Lacordaire, 1859**

Goniadérides Lacordaire, 1859: 390. Type genus: *Goniadera* Perty, 1832.

Phobeliina Ardoin, 1961: 33. Type genus: *Phobelius* Blanchard, 1845.

**Genus *Anaedus* Blanchard, 1842** [M]

*Aspisoma* Duponchel and Chevrolat, 1841: 210 [junior homonym of *Aspisoma* Laporte, 1833]. Type species: *Aspisoma fulvipenne* Duponchel and Chevrolat, 1841, original designation.

*Anaedus* Blanchard, 1842: pl. 14. Type species: *Anaedus punctatissimus* Blanchard, 1842, monotypy. Synonymy: Lacordaire (1859: 396).

*Anaedes* Agassiz, 1846: 36. Unjustified emendation of *Anaedus* Blanchard, 1842, not in prevailing usage.

*Aspidosoma* Agassiz, 1846: 36. Unjustified emendation of *Aspisoma* Duponchel and Chevrolat, 1841, not in prevailing usage.

***Anaedus aeneotinctus* Champion, 1893** MEX (GE)

*Anaedus aeneotinctus* Champion, 1893a: 543.

***Anaedus apicicornis* Champion, 1886** MEX (JA) PAN

*Anaedus apicicornis* Champion, 1886: 236.

***Anaedus brevicollis* Champion, 1886** GUA

*Anaedus brevicollis* Champion, 1886: 236.

**Figure 3.**
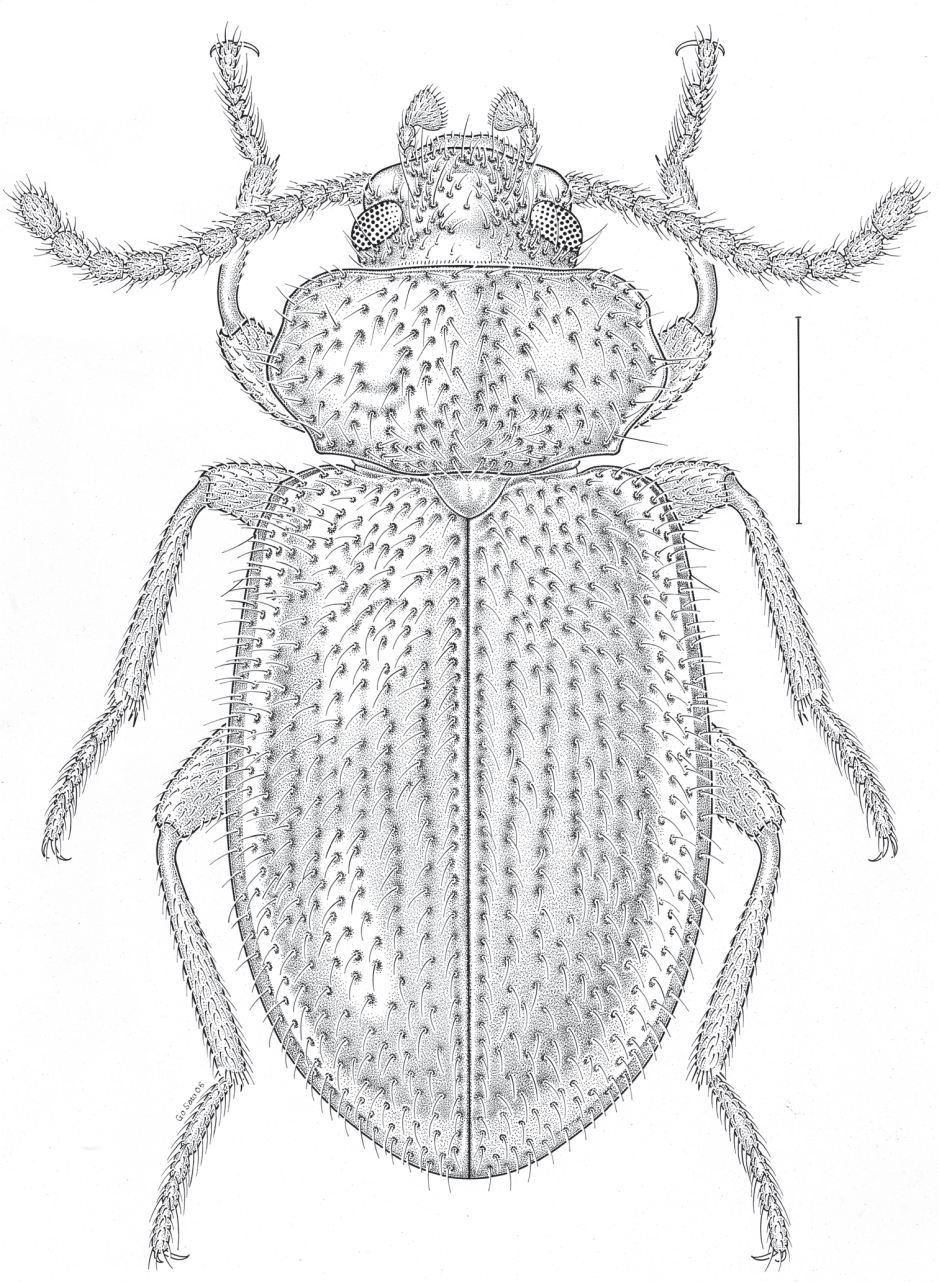
*Anaedus brunneus* (Ziegler, 1844). Scale bar = 1 mm.

***Anaedus brunneus* (Ziegler, 1844)** [Fig. 3] CAN (ON) USA (AL AR DC FL IN KS KY LA MA MD MO MS NC NJ OH PA RI SC TN VA WI)

*Pandarus brunneus* Ziegler, 1844: 45.

***Anaedus impressicollis* Pic, 1917** MEX

*Anaedus impressicollis* Pic, 1917: 19.

***Anaedus inangulatus* (Pic, 1934)** NIC

*Aspisoma inangulata* Pic, 1934: 35.

***Anaedus longicornis* Champion, 1886** USA (TX) MEX (GU OA) GUA

*Anaedus longicornis* Champion, 1886: 235.

***Anaedus maculatus* Champion, 1886** NIC PAN

*Anaedus maculatus* Champion, 1886: 235.

***Anaedus marginatus* Champion, 1886** NIC PAN

*Anaedus marginatus* Champion, 1886: 236.

***Anaedus mexicanus* Champion, 1886** MEX (VE)

*Anaedus mexicanus* Champion, 1886: 234.

***Anaedus nitidissimus* Pic, 1917** CRI

*Anaedus nitidissimus* Pic, 1917: 20.

***Anaedus pallidus* Schaeffer, 1915** USA (TX)

*Anaedus pallidus* Schaeffer, 1915: 238.

***Anaedus punctatissimus* Blanchard, 1842** MEX (DU JA OA PU SI VE) GUA NIC CRI PAN/ SA

*Anaedus punctatissimus* Blanchard, 1842: pl. 14.

***Anaedus quadrinotatus* Champion, 1896** LAN

*Anaedus quadrinotatus* Champion, 1896: 26.

***Anaedus rotundicollis* LeConte, 1851** USA (AZ) MEX (BS)

*Anoedus rotundicollis* LeConte, 1851: 150.

***Anaedus setulosus* Champion, 1886** MEX (TB) NIC PAN / SA

*Anaedus setulosus* Champion, 1886: 237.

***Anaedus similis* Champion, 1886** MEX (VE) GUA NIC

*Anaedus similis* Champion, 1886: 234.

***Anaedus texanus* Linell, 1899** USA (TX)

*Anoedus texanus* Linell, 1899: 182.

***Anaedus villosus* Champion, 1893** GUA CRI PAN

*Anaedus villosus* Champion, 1893a: 543.

**Genus *Goniadera* Perty, 1832** [F]

*Goniadera* Perty, 1832: 62^5^. Type species: *Goniadera crenata* Perty, 1832, monotypy.

*Goniodera* Agassiz, 1846: 165. Unjustified emendation of *Goniadera* Perty, 1832, not in prevailing usage.

**Subgenus Aemymone Bates, 1868**

*Aemymone* Bates, 1868: 314. Type species: *Aemymone cariosa* Bates, 1868, original designation.

***Goniadera championi* Ferrer and Delatour, 2007** MEX (VE) PAN

*Aemymone crenata* Champion, 1893a: 542 [junior secondary homonym of *Goniadera crenata* Perty, 1832].

*Goniadera championi* Ferrer and Delatour, 2007: 286. Replacement name for *Goniadera crenata* (Champion, 1893).

**Subgenus Goniadera Perty, 1832**

*Goniadera* Perty, 1832: 62. Type species: *Goniadera crenata* Perty, 1832, monotypy.

***Goniadera alternata* Champion, 1886** MEX (VE) GUA BEL PAN

*Goniadera alternata* Champion, 1886: 231.

***Goniadera dissipata* Kirsch, 1866** PAN / JAM LAN / SA

*Goniadera dissipata* Kirsch, 1866: 197.

***Goniadera nicaraguensis* Champion, 1886** NIC

*Goniadera nicaraguensis* Champion, 1886: 230.

***Goniadera obscuriceps* Pic, 1913** NIC / SA

*Goniadera obscuriceps* Pic, 1913a: 125.

***Goniadera oculata oculata* Champion, 1886** MEX (GE OA VE YU) BEL NIC CRI PAN

*Goniadera oculata* Champion, 1886: 230.

***Goniadera pilosa* Champion, 1886** NIC CRI PAN

*Goniadera pilosa* Champion, 1886: 230.

***Goniadera pseudorepanda* Ferrer and Delatour, 2007** MEX (GE VE YU) GUA NIC CRI / SA

*Goniadera pseudorepanda* Ferrer and Delatour, 2007: 296.

***Goniadera repanda* (Fabricius, 1801)** MEX (JA VE) GUA BEL NIC CRI / SA

*Melandrya repanda* Fabricius, 1801a: 165.

**Subgenus Opatresthes Gebien, 1928**

*Opatresthes* Gebien, 1928b: 192. Type species: *Opatresthes binodosus* Gebien, 1928, subsequent designation (Gebien 1941: 817).

***Goniadera maesi* Ferrer and Delatour, 2007** NIC

*Goniadera maesi* Ferrer and Delatour, 2007: 287.

***Goniadera quadrinodosa* (Gebien, 1928)** ^6^ CRI / SA

*Opatresthes quadrinodosus* Gebien, 1928b: 193.

**Genus *Paratenetus* Spinola, 1844** [M]

*Paratenetus* Spinola, 1844: 116. Type species: *Paratenetus punctatus* Spinola, 1844, subsequent designation (Lucas 1920: 483).

*Lagriola* Kirsch, 1874: 409. Type species: *Lagriola operosa* Kirsch, 1874, **present designation**. Synonymy: Matthews and Lawrence (2015: 289).

*Storthephora* Mäklin, 1875a: 658. Type species: *Storthephora denticollis* Mäklin, 1875, subsequent designation (Bousquet and Bouchard 2014: 26). Synonymy: Champion (1893c: 47).

***Paratenetus brevipennis* Champion, 1886** PAN

*Paratenetus brevipennis* Champion, 1886: 242.

***Paratenetus championi* Matthews and Lawrence, 2015** PAN

*Paratenetus denticulatus* Champion, 1886: 243 [secondary homonym of *Paratenetus denticulatus* (Kirsch, 1874)].

*Paratenetus championi* Matthews and Lawrence, 2015: 311. Replacement name for *Paratenetus denticulatus* Champion, 1886.

***Paratenetus constrictus* Champion, 1893** MEX (CI TB VE) GUA BEL CRI PAN

*Paratenetus constrictus* Champion, 1893a: 546.

***Paratenetus corticarioides* Champion, 1886** MEX (OA) GUA

*Paratenetus corticarioides* Champion, 1886: 241.

***Paratenetus crenulatus* Champion, 1886** PAN

*Paratenetus crenulatus* Champion, 1886: 242.

***Paratenetus exutus* Bousquet and Bouchard, 2014** CAN (AB MB NB NS ON QC SK) USA (AL AR CT DC FL IA IL IN KS KY LA MD ME MI MN MO MS NC ND NJ NY OH OK PA TN TX VA WI WV)

*Paratenetus exutus* Bousquet and Bouchard, 2014: 39.

***Paratenetus foveithorax* Ferrer and Ødegaard, 2005** PAN

*Paratenetus foveithorax* Ferrer and Ødegaard, 2005: 635.

**Figure 4.**
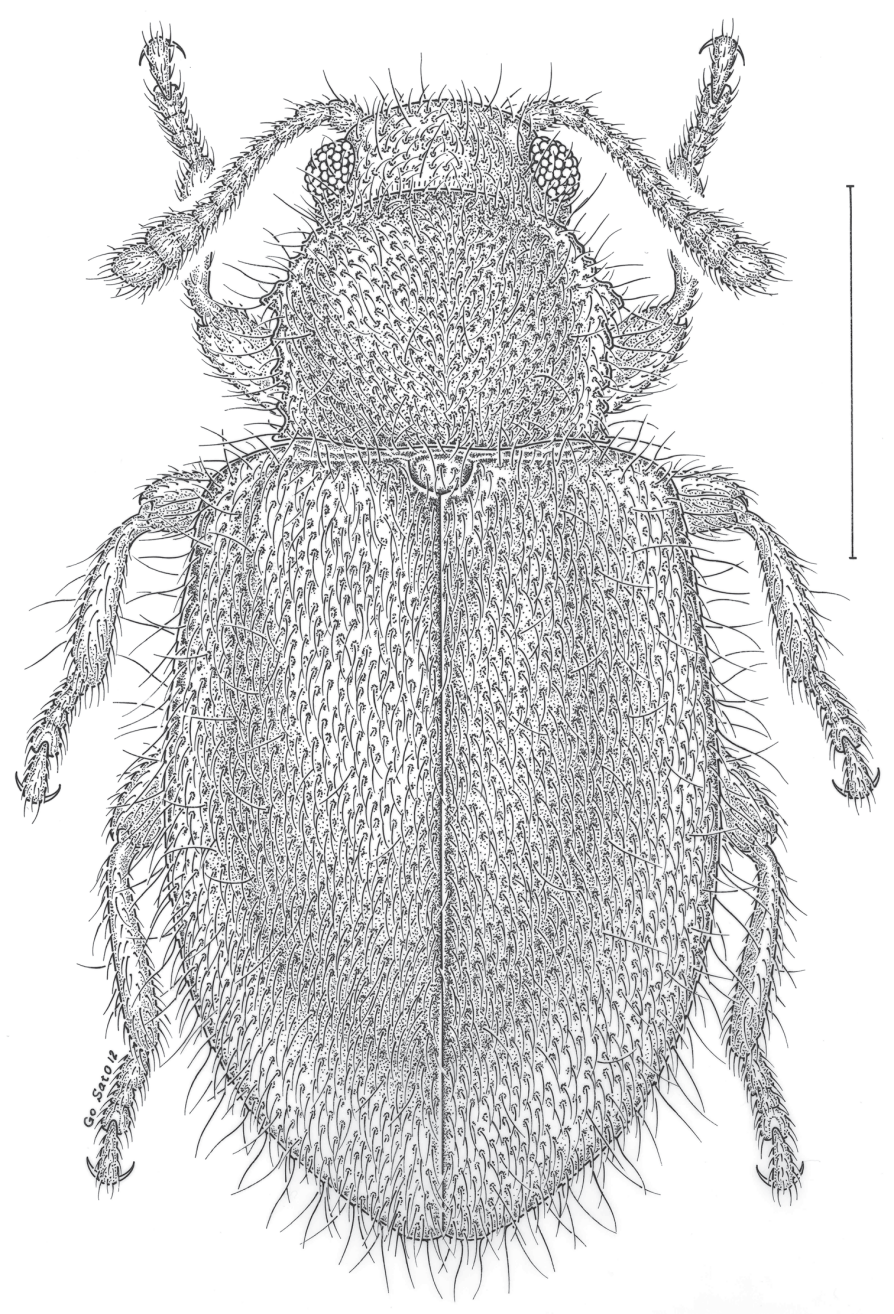
*Paratenetus fuscus* LeConte, 1850. Scale bar = 1 mm.

***Paratenetus fuscus* LeConte, 1850** [Fig. 4] CAN (AB BC MB NT ON QC SK) USA (CO CT DC IA KS MA MD MI MT ND NE NM NY OH RI SD TN VT WI WY)

*Paratenetus fuscus* LeConte, 1850: 223.

*Paratenetus crinitus* Fall, 1907a: 253. Synonymy: Bousquet and Bouchard (2014: 31).

***Paratenetus gibbipennis* Motschulsky, 1869** CAN (MB ON QC) USA (AL CT GA IL MA ME MI MN MO NC ND NE NH NJ NY OH PA RI SC TN TX VA WI)

*Paratenetus gibbipennis* Motschulsky, 1869: 193.

*Paratenetus cribratus* Motschulsky, 1869: 193. Synonymy: Bousquet and Bouchard (2014: 29).

***Paratenetus grandicornis* Motschulsky, 1869** NIC PAN

*Paratenetus grandicornis* Motschulsky, 1869: 193.

***Paratenetus inermis* Champion, 1893** GUA

*Paratenetus inermis* Champion, 1893a: 545.

***Paratenetus koltzei* Pic, 1939** MEX

*Paratenetus koltzei* Pic, 1939: 9.

***Paratenetus longicornis* Pic, 1925** LAN (Guadeloupe)

*Paratenetus longicornis* Pic, 1925a: 6.

***Paratenetus mexicanus* Pic, 1925** MEX (SI)

*Paratenetus mexicanus* Pic, 1925a: 6.

***Paratenetus nigricornis* Champion, 1893** MEX (TB VE) GUA BEL PAN

*Paratenetus nigricornis* Champion, 1893a: 544.

***Paratenetus obovatus* Champion, 1886** BEL

*Paratenetus obovatus* Champion, 1886: 241.

***Paratenetus punctatus* Spinola, 1844** CAN (MB NB ON QC) USA (AR CT DC GA IA IL IN KS KY LA MA MD ME MI MN MO MS NC NH NJ NY OH OK PA RI SC TN TX VA VT WI WV WY)

*Latridius pubescens* Say, 1826: 265 [*nomen dubium*, see Bousquet and Bouchard (2014: 33)].

*Paratenetus punctatus* Spinola, 1844: 118.

***Paratenetus punctulatus* Champion, 1893** MEX (TB VE) BEL

*Paratenetus punctulatus* Champion, 1893a: 545.

***Paratenetus ruficornis* Champion, 1886** PAN

*Paratenetus ruficornis* Champion, 1886: 239.

***Paratenetus sexdentatus* Champion, 1893** GUA BEL PAN

*Paratenetus sexdentatus* Champion, 1893a: 546.

***Paratenetus testaceus* Pic, 1920** MEX (PU) CRI

*Paratenetus testaceus* Pic, 1920: 2.

***Paratenetus texanus* Bousquet and Bouchard, 2014** USA (FL LA TX) MEX (CI NA TA)

*Paratenetus texanus* Bousquet and Bouchard, 2014: 45.

***Paratenetus tibialis* Champion, 1886** MEX (GE TB VE) GUA BEL

*Paratenetus tibialis* Champion, 1886: 239.

***Paratenetus tropicalis* Motschulsky, 1869** MEX (TB VE) GUA BEL PAN

*Paratenetus tropicalis* Motschulsky, 1869: 193.

***Paratenetus tuberculatus* Champion, 1886** PAN

*Paratenetus tuberculatus* Champion, 1886: 242.

***Paratenetus villosus* Champion, 1886** MEX (VE) GUA PAN

*Paratenetus villosus* Champion, 1886: 240.

**Genus *Phobelius* Blanchard, 1842** [M]

*Phobelius* Blanchard, 1842: pl. 14. Type species: *Phobelius crenatus* Blanchard, 1842, monotypy.

***Phobelius mexicanus* Doyen, 1990** MEX (JA) GUA

*Phobelius mexicanus* Doyen, 1990: 217.

***Phobelius nevermanni* Kulzer, 1961** CRI

*Phobelius nevermanni* Kulzer, 1961a: 226.

**Genus *Phymatestes* Pascoe, 1867** [M]

*Phymatodes* Dejean, 1834: 203. Type species: *Lagria tuberculata* Fabricius, 1792, monotypy. Note. Dejean’s name has been suppressed by the Commission in Opinion 1525 (ICZN 1989).

*Phymatestes* Pascoe, 1867: 142. Replacement name for *Phymatodes* Dejean, 1834 [as *Phymatodes* Blanchard, 1845]. Note. This name has been placed on the Official List of Generic Names in Zoology in Opinion 1525 (ICZN 1989).

***Phymatestes agnei* Ferrer and Ødegaard, 2005** PAN

*Phymatestes agnei* Ferrer and Ødegaard, 2005: 634.

***Phymatestes charbonnelae* Ferrer and Moraguès, 2003** LAN (Grenada)

*Phymatestes charbonnelae* Ferrer and Moraguès, 2003: 161.

**Genus *Prateus* LeConte, 1862** [M]

*Prateus* LeConte, 1862a: 238. Type species: *Prateus fusculus* LeConte, 1862, original designation.

***Prateus fusculus* LeConte, 1862** USA (AL AR DC FL MD MS NC NY OH OK SC TN TX VA WV) MEX (TA)

*Prateus fusculus* LeConte, 1862a: 238.

**Genus *Xanthicles* Champion, 1886** [M]

*Xanthicles* Champion, 1886: 231. Type species: *Xanthicles caraboides* Champion, 1886, subsequent designation (Gebien 1941: 815).

***Xanthicles caraboides* Champion, 1886** CRI

*Xanthicles caraboides* Champion, 1886: 232.

***Xanthicles hirsutus* Champion, 1886** CRI

*Xanthicles hirsutus* Champion, 1886: 232.

**Tribe Lagriini Latreille, 1825**

Lagriariae Latreille, 1825: 381. Type genus: *Lagria* Fabricius, 1775.

**Subtribe Statirina Blanchard, 1845**

Statyrites Blanchard, 1845: 39. Type genus: *Statira* Lepeletier and Audinet-Serville, 1828.

**Genus *Arthromacra* Kirby, 1837** [F]

*Arthromacra* Kirby, 1837: 238. Type species: *Arthromacra donacioides* Kirby, 1837 (= *Lagria aenea* Say, 1824), monotypy.

*Macrarthra* Agassiz, 1846: 219. Unjustified emendation of *Arthromacra* Kirby, 1837, not in prevailing usage.

***Arthromacra aenea aenea* (Say, 1824)** CAN (MB NB NS ON PE QC) USA (CT DC DE MA MD ME MI NC NH NJ NY OH PA TN VA VT WI WV)

*Lagria aenea* Say, 1824b: 287 [junior primary homonym of *Lagria aenea* Fabricius, 1775].

*Arthromacra donacioides* Kirby, 1837: 239. Synonymy: LeConte (1859d: 191).

*Lagria viridis* Melsheimer, 1845: 311. Synonymy: Parsons (1976: 222).

***Arthromacra aenea glabricollis* Blatchley, 1910** USA (IL IN KY MO OH PA VA WI)

*Arthromacra glabricollis* Blatchley, 1910: 1285.

***Arthromacra aenea lengi* Parsons, 1976** USA (GA NC PA SC TN WV)

*Arthromacra aenea lengi* Parsons, 1976: 224.

***Arthromacra aenea rugosecollis* Leng, 1914** USA (GA NC TN)

Arthromacra aenea var. rugosecollis Leng, 1914: 287.

***Arthromacra appalachiana* Leng, 1917** USA (NC SC TN VA)

*Arthromacra appalachiana* Leng, 1917: 18.

***Arthromacra pilosella* Leng, 1917** USA (KY NC SC TN)

*Arthromacra pilosella* Leng, 1917: 18.

***Arthromacra robinsoni* Leng, 1914** USA (NC SC VA)

*Arthromacra robinsoni* Leng, 1914: 286.

**Genus *Colparthrum* Kirsch, 1866** [N]

*Colparthrum* Kirsch, 1866: 204. Type species: *Colparthrum gerstaeckeri* Kirsch, 1866, monotypy.

**Subgenus Colparthrum Kirsch, 1866**

*Colparthrum* Kirsch, 1866: 204. Type species: *Colparthrum gerstaeckeri* Kirsch, 1866, monotypy.

***Colparthrum aenescens* Borchmann, 1936** CRI

*Colparthrum aenescens* Borchmann, 1936: 444.

***Colparthrum decoratum bilunulatum* (Pic, 1912)** PAN

*Statira bilunulata* Pic, 1912b: 76.

***Colparthrum decoratum decoratum* (Mäklin, 1863)** MEX (VE) GUA NIC PAN

*Statira decorata* Mäklin, 1863: 588.

***Colparthrum decoratum maklini* Borchmann, 1936** ^7^ [No region originally mentioned but presumably from Mexico and/or Central America]

*Colparthrum decoratum* var. *mäklini* Borchmann, 1936: 447.

***Colparthrum foveiceps* Champion, 1889** PAN

*Colparthrum foveiceps* Champion, 1889: 68.

***Colparthrum grande* Borchmann, 1936** CRI

*Colparthrum grandis* Borchmann, 1936: 439.

***Colparthrum majus* Borchmann, 1916** MEX

Colparthrum decoratum var. major Borchmann, 1916: 233.

**Subgenus Pseudocolparthrum Borchmann, 1916**

*Pseudocolparthrum* Borchmann, 1916: 236. Type species: *Colparthrum calcaratum* Champion, 1889, subsequent designation (Borchmann 1936: 452).

***Colparthrum calcaratum* Champion, 1889** NIC CRI PAN

*Colparthrum calcaratum* Champion, 1889: 71.

***Colparthrum sulcicolle* Champion, 1889** NIC PAN

*Colparthrum sulcicolle* Champion, 1889: 69.

***Colparthrum vitticolle* Champion, 1889** NIC

*Colparthrum vitticolle* Champion, 1889: 70.

**Genus *Disema* Mäklin, 1875** [F]

*Disema* Mäklin, 1875a: 646. Type species: *Disema bimaculata* Mäklin, 1875, subsequent designation (Lucas 1920: 244).

***Disema singularis* (Champion, 1889)** PAN

*Sphragidophorus singularis* Champion, 1889: 64.

**Genus *Epicydes* Champion, 1889** [M]

*Epicydes* Champion, 1889: 60. Type species: *Epicydes oculatus* Champion, 1889, subsequent designation (Borchmann 1936: 429).

**Subgenus Cybostira Borchmann, 1936**

*Cybostira* Borchmann, 1936: 430. Type species: *Cybostira caligata* Borchmann, 1936, original designation.

***Epicydes caligatus* (Borchmann, 1936)** CRI

*Cybostira caligata* Borchmann, 1936: 430.

**Subgenus Epicydes Champion, 1889**

*Epicydes* Champion, 1889: 60. Type species: *Epicydes oculatus* Champion, 1889, subsequent designation (Borchmann 1936: 429).

***Epicydes oculatus* Champion, 1889** MEX (OA VE) GUA

*Epicydes oculatus* Champion, 1889: 61.

***Epicydes vicinus* Champion, 1889** GUA NIC

*Epicydes vicinus* Champion, 1889: 61.

**Genus *Meniscophorus* Champion, 1889** [M]

*Meniscophorus* Champion, 1889: 64. Type species: *Meniscophorus amazonicus* Champion, 1889, subsequent designation (Lucas 1920: 404).

***Meniscophorus costatus* Champion, 1889** PAN

*Meniscophorus costatus* Champion, 1889: 65.

**Genus *Meropria* Borchmann, 1921** [F]

*Meropria* Borchmann, 1921: 228. Type species: *Statira glabrata* Mäklin, 1863, original designation.

***Meropria chiriquina* (Champion, 1889)** PAN

*Statira chiriquina* Champion, 1889: 9.

***Meropria denticulata* (Champion, 1889)** PAN / SA

*Statira denticulata* Champion, 1889: 7.

***Meropria glabrata* (Mäklin, 1863)** MEX (GE MO OA VE) GUA BEL CRI

*Statira glabrata* Mäklin, 1863: 587.

***Meropria interrupta* (Champion, 1889)** GUA NIC PAN

*Statira interrupta* Champion, 1889: 8.

***Meropria unidentata* (Champion, 1889)** MEX (CI VE) GUA BEL

*Statira unidentata* Champion, 1889: 8.

**Genus *Nevermanniella* Borchmann, 1936** [F]

*Nevermanniella* Borchmann, 1936: 332. Type species: *Statira albolineata* Champion, 1889, original designation.

***Nevermanniella albolineata* (Champion, 1889)** MEX (VE) BEL NIC

*Statira albolineata* Champion, 1889: 36.

**Genus *Othryades* Champion, 1889** [M]

*Othryades* Champion, 1889: 72. Type species: *Othryades fragilicornis* Champion, 1889, monotypy.

***Othryades fragilicornis* Champion, 1889** PAN

*Othryades fragilicornis* Champion, 1889: 72.

**Genus *Rhaibodera* Borchmann, 1921** [F]

*Rhaibodera* Borchmann, 1921: 219. Type species: *Rhaibodera pachycera* Borchmann, 1921, original designation.

***Rhaibodera crassicornis* (Champion, 1889)** MEX (TB)

*Statira crassicornis* Champion, 1889: 18.

**Genus *Rhosaces* Champion, 1889** [M]

*Rhosaces* Champion, 1889: 73. Type species: *Rhosaces clavipes* Champion, 1889, monotypy.

***Rhosaces clavipes* Champion, 1889** PAN

*Rhosaces clavipes* Champion, 1889: 73.

**Genus *Sphragidophorus* Champion, 1889** [M]

*Sphragidophorus* Champion, 1889: 61. Type species: *Statira cyanipennis* Mäklin, 1863, subsequent designation (Lucas 1920: 603).

***Sphragidophorus cyanipennis* (Mäklin, 1863)** MEX (VE) GUA PAN

*Statira cyanipennis* Mäklin, 1863: 591.

***Sphragidophorus ocularis* Borchmann, 1936** CRI

*Sphragidophorus ocularis* Borchmann, 1936: 507.

***Sphragidophorus violaceus* Champion, 1889** PAN

*Sphragidophorus violaceus* Champion, 1889: 63.

**Genus *Statira* Lepeletier and Audinet-Serville, 1828** [F]

*Statira* Lepeletier and Audinet-Serville, 1828: 479. Type species: *Statira agroides* Lepeletier and Audinet-Serville, 1828, subsequent designation (Blanchard 1844: pl. 53bis).

**Subgenus Spinostatira Pic, 1918**

*Spinostatira* Pic, 1918a: 22. Type species: *Statira spinipes* Pic, 1918, subsequent designation (Borchmann 1936: 247).

***Statira costaricensis* Champion, 1889** CRI / SA

*Statira costaricensis* Champion, 1889: 36.

**Subgenus Statira Lepeletier and Audinet-Serville, 1828**

*Statira* Lepeletier and Audinet-Serville, 1828: 479. Type species: *Statira agroides* Lepeletier and Audinet-Serville, 1828, subsequent designation (Blanchard 1844: pl. 53bis).

***Statira aeneipennis* Champion, 1889** GUA

*Statira aeneipennis* Champion, 1889: 25.

***Statira aeneotincta* Champion, 1889** MEX (VE) GUA

*Statira aeneotincta* Champion, 1889: 27.

***Statira aerata* Champion, 1889** MEX (VE) GUA

*Statira aerata* Champion, 1889: 26.

***Statira agraeformis* Champion, 1889** PAN

*Statira agraeformis* Champion, 1889: 12.

***Statira albofasciata* Champion, 1889** MEX (VE) GUA PAN

*Statira albofasciata* Champion, 1889: 44.

***Statira alternans* Champion, 1889** MEX (OA)

*Statira alternans* Champion, 1889: 30.

***Statira amicta* Borchmann, 1936** CRI

*Statira amicta* Borchmann, 1936: 294.

***Statira analis* Borchmann, 1921** MEX

*Statira analis* Borchmann, 1921: 273.

***Statira angustula* Champion, 1889** GUA

*Statira angustula* Champion, 1889: 15.

***Statira antennalis* Borchmann, 1936** CRI

*Statira antennalis* Borchmann, 1936: 313.

***Statira asperata* Champion, 1889** PAN / LAN / SA

*Statira asperata* Champion, 1889: 49.

*Statira antillarum* Champion, 1896: 36. Synonymy: Champion (1917: 230).

***Statira basalis* Horn, 1888** USA (AL AR FL GA LA MD MO MS NC SC TX VA)

*Statira basalis* Horn, 1888: 31.

***Statira bicolor* Champion, 1889** PAN

*Statira bicolor* Champion, 1889: 47.

***Statira biseriata* Borchmann, 1921** MEX

*Statira biseriata* Borchmann, 1921: 263.

***Statira borchmanni* Nevermann, 1926** CRI

*Statira borchmanni* Nevermann, 1926: 113.

***Statira brevipilis* Champion, 1889** MEX (VE)

*Statira brevipilis* Champion, 1889: 39.

***Statira caeruleipennis* Champion, 1889** MEX (MI)

*Statira caeruleipennis* Champion, 1889: 14.

***Statira ciliata* Champion, 1889** GUA

*Statira ciliata* Champion, 1889: 42.

***Statira collaris* Champion, 1889** MEX (VE)

*Statira collaris* Champion, 1889: 13.

***Statira colorata* Fall, 1909** MEX (BC BS)

*Statira colorata* Fall, 1909: 165.

***Statira conspicillata conspicillata* Mäklin, 1863** MEX (CI TB VE) GUA BEL NIC PAN

*Statira conspicillata* Mäklin, 1863: 589.

***Statira conspicillata lateannulata* Borchmann, 1936** GUA

Statira conspicillata var. lateannulata Borchmann, 1936: 291.

***Statira corrosa* Champion, 1889** MEX GUA / SA

*Statira corrosa* Champion, 1889: 37.

***Statira cribrata* Champion, 1889** GUA

*Statira cribrata* Champion, 1889: 42.

***Statira croceicollis* Mäklin, 1863** USA (AL FL GA MS)

*Statira croceicollis* Mäklin, 1863: 594.

***Statira cruciata* Champion, 1917** NIC

*Statira cruciata* Champion, 1917: 254.

***Statira cupreotincta* Champion, 1889** NIC PAN

*Statira cupreotincta* Champion, 1889: 23.

***Statira curticollis* Champion, 1889** MEX (GE ME MO OA) GUA

*Statira curticollis* Champion, 1889: 24.

***Statira defecta* Schaeffer, 1905** USA (AZ NM)

*Statira defecta* Schaeffer, 1905b: 175.

***Statira dolera* Parsons, 1966** USA (FL GA)

*Statira dolera* Parsons, 1966: 249.

***Statira dumalis* Parsons, 1973** USA (CA)

*Statira dumalis* Parsons, 1973: 1.

***Statira erina* Parsons, 1976** USA (TX)

*Statira erina* Parsons, 1976: 219.

***Statira evanescens evanescens* Champion, 1889** MEX (DU GE JA MO OA VE) NIC

*Statira evanescens* Champion, 1889: 34.

***Statira evanescens obscuripennis* Borchmann, 1921** MEX

Statira evanescens var. obscuripennis Borchmann, 1921: 260.

***Statira flohri* Champion, 1893** MEX (OA)

*Statira flohri* Champion, 1893b: 452.

***Statira foveicollis* Champion, 1889** BEL NIC PAN

*Statira foveicollis* Champion, 1889: 18.

***Statira fulva* Fleutiaux and Sallé, 1890** LAN

*Statira fulva* Fleutiaux and Sallé, 1890: 431.

**Figure 5.**
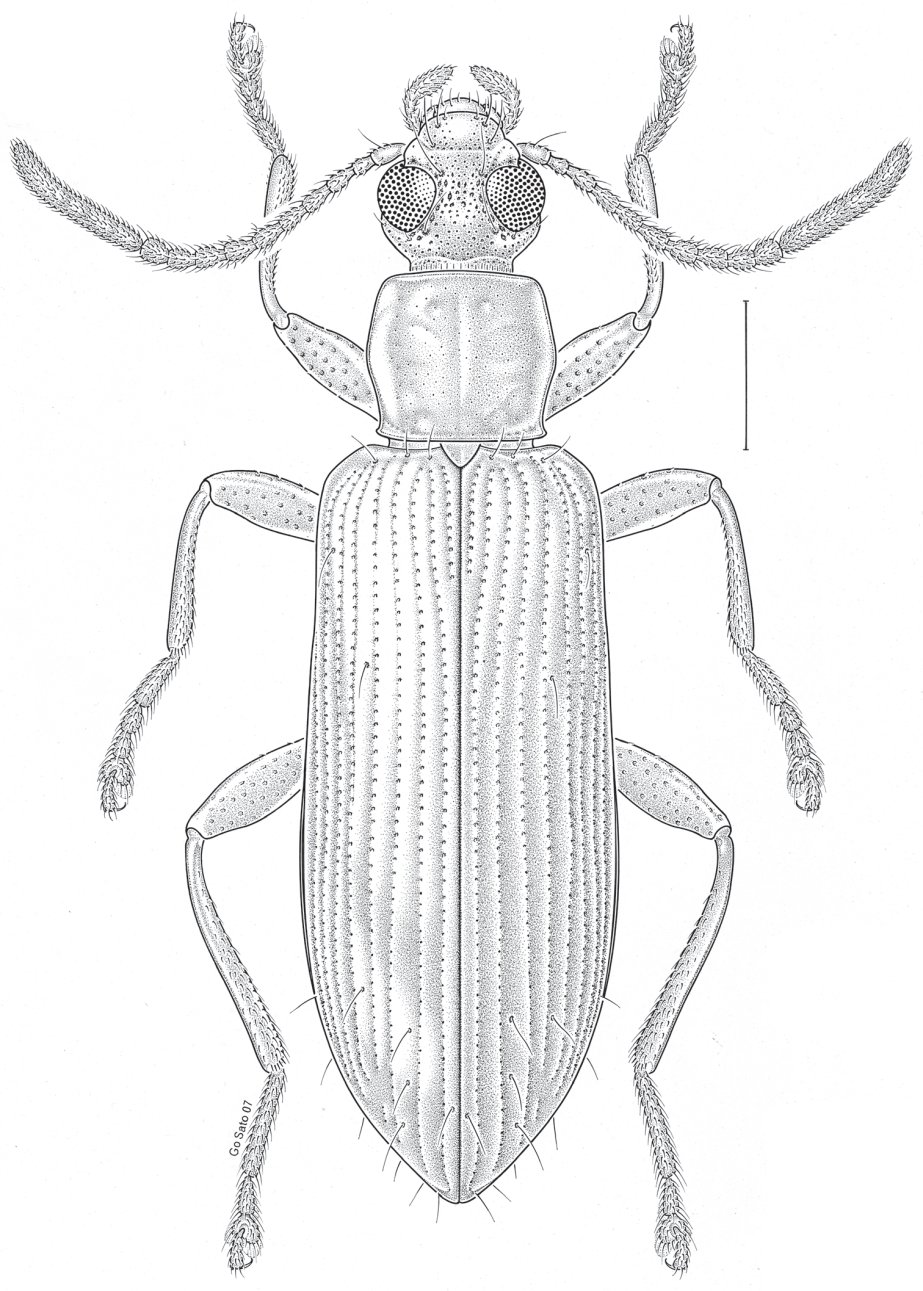
*Statira gagatina* Melsheimer, 1845. Scale bar = 1 mm.

***Statira gagatina* Melsheimer, 1845** [Fig. 5] CAN (QC) USA (AL AR CT DC DE IA IL IN KS KY MA MD MI MS NC NJ NY OH PA SC TN VT WI)

*Statyra gagatina* Melsheimer, 1845: 311.

*Statyra resplendens* Melsheimer, 1845: 311. Synonymy: Parsons (1976: 220).

*Lagria fusca* Melsheimer, 1845: 311. Synonymy: Parsons (1966: 252).

***Statira glabricollis* Borchmann, 1936** CRI

*Statira glabricollis* Borchmann, 1936: 315.

***Statira guatemalensis* Champion, 1889** GUA

*Statira guatemalensis* Champion, 1889: 48.

***Statira guttata* Borchmann, 1921** MEX (“Tenancingo”)

*Statira guttata* Borchmann, 1921: 250.

***Statira haitiensis* Champion, 1917** HAI

*Statira haitiensis* Champion, 1917: 255.

***Statira heliconiae* Borchmann, 1936** CRI

*Statira heliconiae* Borchmann, 1936: 292.

***Statira heliophila* Borchmann, 1936** CRI

*Statira heliophila* Borchmann, 1936: 276.

***Statira hirsuta* Champion, 1889** USA (TX) MEX (CI GE JA VE) NIC

*Statira hirsuta* Champion, 1889: 50.

*Statira simulans* Schaeffer, 1905a: 180. Synonymy: Parsons (1966: 246).

***Statira horrida* Champion, 1889** GUA

*Statira horrida* Champion, 1889: 38.

***Statira huachucae* Schaeffer, 1905** USA (AZ NM)

*Statira huachucae* Schaeffer, 1905b: 176.

***Statira ignita* Champion, 1889** MEX (VE)

*Statira ignita* Champion, 1889: 23.

***Statira inaequicollis* Borchmann, 1936** CRI

*Statira inaequicollis* Borchmann, 1936: 287.

***Statira inconstans* Champion, 1889** GUA NIC

*Statira inconstans* Champion, 1889: 16.

***Statira ingae* Borchmann, 1936** CRI

*Statira ingae* Borchmann, 1936: 295.

***Statira ingens* Champion, 1889** NIC CRI PAN

*Statira ingens* Champion, 1889: 12.

***Statira irazuensis* Champion, 1889** CRI

*Statira irazuensis* Champion, 1889: 22.

***Statira irregularis* Champion, 1889** GUA

*Statira irregularis* Champion, 1889: 45.

***Statira isthmiaca* Champion, 1889** PAN

*Statira isthmiaca* Champion, 1889: 19.

***Statira laevicollis* Champion, 1889** MEX (CL GE)

*Statira laevicollis* Champion, 1889: 46.

***Statira latitator* Parsons, 1973** USA (CA) MEX (BC)

*Statira latitator* Parsons, 1973: 3.

***Statira leptotracheloides* Champion, 1889** MEX (DU)

*Statira leptotracheloides* Champion, 1889: 52.

***Statira liebecki* Leng, 1924** USA (AL FL SC)

*Statira liebecki* Leng, 1924: 187.

***Statira limbata* Champion, 1889** MEX (TB VE YU) BEL

*Statira limbata* Champion, 1889: 14.

***Statira limonis* Borchmann, 1936** CRI

*Statira limonis* Borchmann, 1936: 315.

***Statira marmorata* Champion, 1889** MEX (VE) GUA

*Statira marmorata* Champion, 1889: 43.

***Statira mediosignata* Borchmann, 1921** MEX (“Santiago Ixcuintla”)

*Statira mediosignata* Borchmann, 1921: 278.

***Statira melanocephala* Mäklin, 1863** MEX (VE)

*Statira melanocephala* Mäklin, 1863: 593.

***Statira metallica* Champion, 1889** NIC CRI PAN

*Statira metallica* Champion, 1889: 16.

***Statira mexicana* Champion, 1889** MEX (PU VE)

*Statira mexicana* Champion, 1889: 26.

***Statira microps* Champion, 1889** MEX (CI TB) GUA

*Statira microps* Champion, 1889: 44.

***Statira minima minima* Champion, 1889** NIC PAN

*Statira minima* Champion, 1889: 47.

***Statira minima subatra* Borchmann, 1936** “Mittelamerika”

Statira minima var. subatra Borchmann, 1936: 276.

***Statira multiformis* Champion, 1889** MEX (VE) GUA NIC PAN

*Statira multiformis* Champion, 1889: 19.

***Statira multipunctata* Champion, 1889** MEX (MO)

*Statira multipunctata* Champion, 1889: 49.

***Statira nevermanni* Borchmann, 1936** CRI

*Statira nevermanni* Borchmann, 1936: 255.

***Statira nigripennis affinis* Mäklin, 1875** MEX

*Statira affinis* Mäklin, 1875a: 642.

***Statira nigripennis championi* Pic, 1912** MEX

Statira nigripennis var. championi Pic, 1912a: 20.

***Statira nigripennis humeralis* Mäklin, 1875** MEX

*Statira humeralis* Mäklin, 1875a: 642.

***Statira nigripennis nigripennis* Mäklin, 1875** MEX (JA MO)

*Statira nigripennis* Mäklin, 1875a: 641.

***Statira nigroaenea* Champion, 1889** MEX (DU)

*Statira nigroaenea* Champion, 1889: 40.

***Statira nigrofasciata* Borchmann, 1921** MEX (“Navarrete”)

*Statira nigrofasciata* Borchmann, 1921: 257.

***Statira nigromaculata* Champion, 1889** USA (AZ TX) MEX (OA VE) GUA

*Statira nigromaculata* Champion, 1889: 33.

***Statira nigrosparsa* Mäklin, 1863** MEX (GE VE) GUA NIC

*Statira nigrosparsa* Mäklin, 1863: 590.

***Statira nodulosa* Champion, 1889** GUA

*Statira nodulosa* Champion, 1889: 31.

***Statira opaca* Borchmann, 1936** CRI

*Statira opaca* Borchmann, 1936: 283.

***Statira opacicollis* Horn, 1888** USA (AZ)

*Statira opacicollis* Horn, 1888: 30.

***Statira paradoxa* Borchmann, 1936** CRI

*Statira paradoxa* Borchmann, 1936: 306.

***Statira patricia* Borchmann, 1921** CRI

*Statira patricia* Borchmann, 1921: 269.

***Statira penicillata* Champion, 1889** MEX (VE)

*Statira penicillata* Champion, 1889: 30.

***Statira perforata* Champion, 1917** MEX

*Statira perforata* Champion, 1917: 262.

***Statira pici* Blackwelder, 1945** CRI

*Statira bimaculata* Borchmann, 1936: 291 [junior primary homonym of *Statira bimaculata* Pic, 1912].

*Statira pici* Blackwelder, 1945: 500. Replacement name for *Statira bimaculata* Borchmann, 1936.

***Statira picta* Champion, 1889** NIC PAN

*Statira picta* Champion, 1889: 36.

***Statira pilifera* Champion, 1893** MEX (VE)

*Statira pilifera* Champion, 1893b: 451.

***Statira pilipes* Champion, 1889** MEX (CI)

*Statira pilipes* Champion, 1889: 43.

***Statira pluripunctata* Horn, 1888** USA (AZ NM TX UT) MEX (GE)

*Statira pluripunctata* Horn, 1888: 29.

*Statira sulcicrus* Champion, 1889: 51. Synonymy: Parsons (1966: 245).

***Statira pueblensis* Champion, 1889** MEX (PU)

*Statira pueblensis* Champion, 1889: 51.

***Statira pulchella* Mäklin, 1863** USA (TX) MEX (SL TA VE) NIC

*Statira pulchella* Mäklin, 1863: 589.

***Statira punctatissima* Champion, 1889** MEX (CI)

*Statira punctatissima* Champion, 1889: 38.

***Statira punctipennis* Champion, 1889** GUA

*Statira punctipennis* Champion, 1889: 28.

***Statira reticulaticollis* Borchmann, 1936** NIC CRI

*Statira reticulaticollis* Borchmann, 1936: 269.

***Statira robusta* Schaeffer, 1905** USA (AZ CO NM TX)

*Statira robusta* Schaeffer, 1905a: 180.

***Statira rugicollis* Champion, 1889** MEX (VE)

*Statira rugicollis* Champion, 1889: 48.

***Statira rugipes* Champion, 1889** MEX (DU)

*Statira rugipes* Champion, 1889: 52.

***Statira schmidti* Borchmann, 1936** CRI

*Statira schmidti* Borchmann, 1936: 304.

***Statira scitula* Champion, 1889** MEX (VE) GUA

*Statira scitula* Champion, 1889: 10.

***Statira setigera* Champion, 1889** MEX (GU)

*Statira setigera* Champion, 1889: 41.

***Statira simplex* Borchmann, 1936** CRI

*Statira simplex* Borchmann, 1936: 284.

***Statira sobrina* Champion, 1889** MEX

*Statira sobrina* Champion, 1889: 22.

***Statira spiculifera* Champion, 1893** MEX (VE)

*Statira spiculifera* Champion, 1893b: 451.

***Statira suavis* Champion, 1889** MEX (DU)

*Statira suavis* Champion, 1889: 15.

***Statira subnitida* LeConte, 1866** MEX (BC BS)

*Statira subnitida* LeConte, 1866b: 141.

***Statira testacea* Champion, 1889** MEX (VE)

*Statira testacea* Champion, 1889: 28.

***Statira tolensis* Champion, 1889** PAN

*Statira tolensis* Champion, 1889: 20.

***Statira triangulifer* Champion, 1889** MEX (CI VE) GUA BEL

*Statira triangulifer* Champion, 1889: 34.

***Statira tristis* Mäklin, 1875** MEX

*Statira tristis* Mäklin, 1875a: 639.

***Statira tropicalis* Champion, 1889** MEX (VE) GUA BEL NIC

*Statira tropicalis* Champion, 1889: 10.

***Statira tuberculifera* Champion, 1889** GUA

*Statira tuberculifera* Champion, 1889: 32.

***Statira tuberosa* Champion, 1889** MEX (VE)

*Statira tuberosa* Champion, 1889: 31.

***Statira variabilis inexpecta* Borchmann, 1936** PAN

Statira variabilis var. inexpecta Borchmann, 1936: 282.

***Statira variabilis variabilis* Champion, 1889** GUA PAN

*Statira variabilis* Champion, 1889: 11.

***Statira veraecrucis* Champion, 1889** MEX (VE)

*Statira veraecrucis* Champion, 1889: 35.

***Statira veraepacis* Champion, 1889** GUA

*Statira veraepacis* Champion, 1889: 24.

***Statira vilis* Mäklin, 1863** MEX (CI TB VE) GUA BEL PAN

*Statira vilis* Mäklin, 1863: 592.

***Statira villosa* Champion, 1889** MEX (GE)

*Statira villosa* Champion, 1889: 39.

***Statira viridicollis* Champion, 1889** PAN

*Statira viridicollis* Champion, 1889: 17.

***Statira vittata* Champion, 1896** LAN

*Statira vittata* Champion, 1896: 37.

**Genus *Uroplatopsis* Champion, 1889** [F]

*Uroplatopsis* Champion, 1889: 53. Type species: *Uroplatopsis imitator* Champion, 1889, subsequent designation (Lucas 1920: 666).

***Uroplatopsis appendiculata* Champion, 1889** PAN

*Uroplatopsis appendiculata* Champion, 1889: 59.

***Uroplatopsis dilaticornis* Champion, 1889** PAN

*Uroplatopsis dilaticornis* Champion, 1889: 58.

***Uroplatopsis excavata* Champion, 1889** PAN

*Uroplatopsis excavata* Champion, 1889: 59.

***Uroplatopsis imitator* Champion, 1889** NIC

*Uroplatopsis imitator* Champion, 1889: 54.

***Uroplatopsis mimica* Champion, 1889** PAN

*Uroplatopsis mimica* Champion, 1889: 57.

***Uroplatopsis planicollis* Champion, 1889** PAN

*Uroplatopsis planicollis* Champion, 1889: 56.

***Uroplatopsis reducta* Pic, 1931** CRI

*Uroplatopsis reducta* Pic, 1931: 33.

***Uroplatopsis reticulata* Champion, 1889** PAN

*Uroplatopsis reticulata* Champion, 1889: 56.

***Uroplatopsis simulans nevermanni* Borchmann, 1936** CRI

Uroplatopsis simulans var. nevermanni Borchmann, 1936: 482.

***Uroplatopsis simulans simulans* Champion, 1889** PAN

*Uroplatopsis simulans* Champion, 1889: 58.

***Uroplatopsis vermiculata* Champion, 1889** NIC

*Uroplatopsis vermiculata* Champion, 1889: 55.

**Tribe Lupropini Lesne, 1926**

Lypropsini Lesne, 1926: 68. Type genus: *Luprops* Hope, 1833 [as *Lyprops*, incorrect subsequent spelling of the type genus name, not in prevailing usage].

**Genus *Lorelus* Sharp, 1876** [M]

*Lorelus* Sharp, 1876: 76. Type species: *Lorelus priscus* Sharp, 1876, monotypy.

*Lorelopsis* Champion, 1896: 15. Type species: *Lorelopsis pilosus* Champion, 1896, monotypy. Synonymy: Doyen (1993: 295).

***Lorelus angustulus* Champion, 1913** GUA

*Lorelus angustulus* Champion, 1913: 165.

***Lorelus bicolor* Doyen, 1993** PRI

*Lorelus bicolor* Doyen, 1993: 296.

***Lorelus brevicornis* Champion, 1896** LAN

*Lorelus brevicornis* Champion, 1896: 14.

***Lorelus breviusculus* Champion, 1913** PAN

*Lorelus breviusculus* Champion, 1913: 165.

***Lorelus cribricollis* Kaszab, 1940** LAN (Guadeloupe)

*Lorelus cribricollis* Kaszab, 1940: 156.

***Lorelus curticollis* Champion, 1913** MEX (VE) GUA PAN

*Lorelus curticollis* Champion, 1913: 164.

***Lorelus curvipes* Champion, 1913** GUA

*Lorelus curvipes* Champion, 1913: 163.

***Lorelus exilis* Champion, 1913** GUA

*Lorelus exilis* Champion, 1913: 166.

***Lorelus glabratus* Doyen, 1993** PRI

*Lorelus glabratus* Doyen, 1993: 297.

***Lorelus guadeloupensis* Kaszab, 1940** LAN (Guadeloupe)

*Lorelus guadeloupensis* Kaszab, 1940: 155.

***Lorelus pilosus* (Champion, 1896)** LAN

*Lorelopsis pilosus* Champion, 1896: 16.

***Lorelus trapeziderus* Champion, 1913** GUA PAN

*Lorelus trapeziderus* Champion, 1913: 167.

***Lorelus wolcotti* Doyen, 1993** PRI

*Lorelus wolcotti* Doyen, 1993: 295.

**Incertae sedis: Lagriinae**

**Genus *Pseudesarcus* Champion, 1913** ^8^ [M]

*Pseudesarcus* Champion, 1913: 115. Type species: *Pseudesarcus villosus* Champion, 1913, original designation.

***Pseudesarcus villosus* Champion, 1913** PAN

*Pseudesarcus villosus* Champion, 1913: 116.

**Subfamily NILIONINAE Oken, 1843**

Nilioniden Oken, 1843: 484. Type genus: *Nilio* Latreille, 1802.

**Genus *Nilio* Latreille, 1802** [M]

*Nilio* Latreille, 1802: 179 [as *Nilion*]^9^. Type species: *Coccinella villosa* Fabricius, 1787, monotypy.

**Subgenus Nilio Latreille, 1802**

*Nilio* Latreille, 1802: 179 [as *Nilion*]. Type species: *Coccinella villosa* Fabricius, 1787, monotypy.

***Nilio chiriquensis* Champion, 1888** PAN

*Nilio chiriquensis* Champion, 1888: 471.

***Nilio fulvopilosus* Champion, 1888** PAN

*Nilio fulvo-pilosus* Champion, 1888: 471.

***Nilio lebasi* J. Thomson, 1860** ^9^ PAN / SA **New North American record**

*Nilio lebasei* J. Thomson, 1860: 10.

***Nilio sallei* J. Thomson, 1860** MEX (VE) GUA

*Nilio sallei* J. Thomson, 1860: 10.

*Nilio sallaei* Champion, 1888: 470. Unjustified emendation of *Nilio sallei* J. Thomson, 1860, not in prevailing usage.

***Nilio thomsoni* Champion, 1888** GUA NIC PAN

*Nilio thomsoni* Champion, 1888: 471.

***Nilio villosus* (Fabricius, 1787)** PAN / SA

*Coccinella villosa* Fabricius, 1787: 379 [junior primary homonym of *Coccinella villosa* Geoffroy, 1785].

**Subfamily PHRENAPATINAE Solier, 1834**

Phrépatides Solier, 1834: 488. Type genus: *Phrenapates* Gray, 1832.

**Tribe Archaeoglenini Watt, 1975**

Archaeoglenini Watt, 1975: 412. Type genus: *Archaeoglenes* Broun, 1893.

**Genus *Archaeoglenes* Broun, 1893** [M]

*Archaeoglenes* Broun, 1893: 188. Type species: *Archaeoglenes costipennis* Broun, 1893, monotypy.

***Archaeoglenes bollensis* Watrous, 1982** PAN

*Archaeoglenes bollensis* Watrous, 1982: 140.

***Archaeoglenes occidentalis* Lawrence, 1979** MEX (CI) BEL PAN / SA

*Archaeoglenes occidentalis* Lawrence [in Doyen and Lawrence], 1979: 358.

***Archaeoglenes pecki* Lawrence, 1979** JAM

*Archaeoglenes pecki* Lawrence [in Doyen and Lawrence], 1979: 358.

***Archaeoglenes puntaensis* Watrous, 1982** PAN

*Archaeoglenes puntaensis* Watrous, 1982: 141.

**Tribe Penetini Lacordaire, 1859**

Pénétides Lacordaire, 1859: 318. Type genus: *Peneta* Lacordaire, 1859.

Phthorini Boddy, 1965: 144. Type genus: *Phtora* Mulsant, 1854 [as *Phthora*, incorrect subsequent spelling, not in prevailing usage].

**Genus *Clamoris* des Gozis, 1886** [F]

*Phtora* Mulsant, 1854: 228 [junior homonym of *Phtora* Germar, 1835]. Type species: *Phtora crenata* Mulsant, 1854, monotypy.

*Clamoris* des Gozis, 1886: 25. Replacement name for *Phtora* Mulsant, 1854.

*Phthora* Champion, 1893a: 531. Unjustified emendation of *Phtora* Mulsant, 1854, not in prevailing usage.

**Figure 6.**
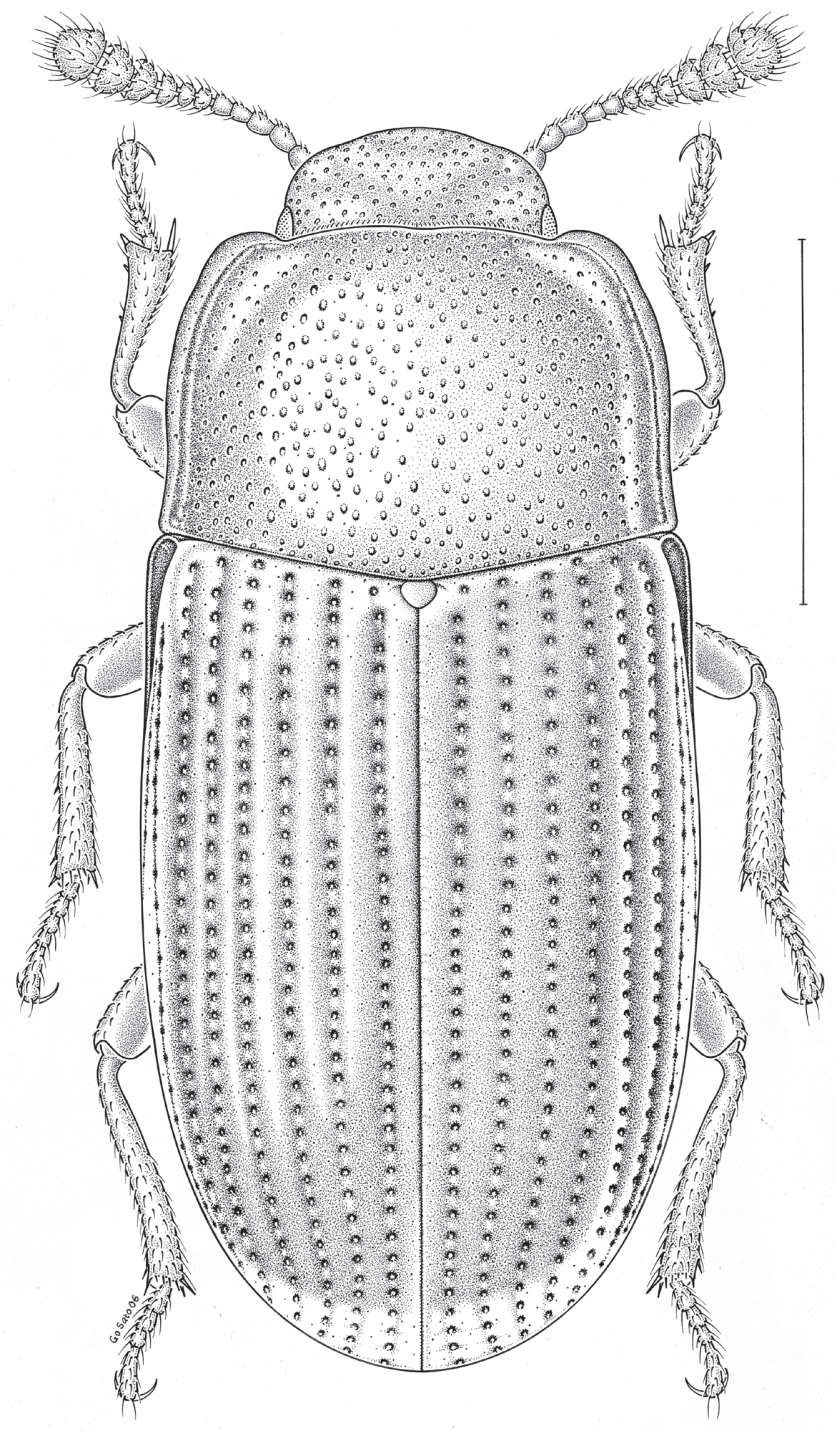
*Clamoris americana* (Horn, 1874). Scale bar = 1 mm.

***Clamoris americana* (Horn, 1874)** [Fig. 6] CAN (BC) USA (CA OR WA)

*Phthora americana* Horn, 1874a: 35.

***Clamoris armata* (Champion, 1893)** GUA

*Phthora armata* Champion, 1893a: 532.

***Clamoris elongata* (Champion, 1893)** MEX (VE) NIC

*Phthora elongata* Champion, 1893a: 532.

**Genus *Cleolaus* Champion, 1886** [M]

*Cleolaus* Champion, 1886: 142. Type species: *Peneta sommeri* Lacordaire, 1859, original designation.

***Cleolaus sommeri* (Lacordaire, 1859)** MEX (OA)

*Peneta sommeri* Lacordaire, 1859: 320.

**Genus *Daochus* Champion, 1886** [M]

*Daochus* Champion, 1886: 139. Type species: *Daochus mandibularis* Champion, 1886, monotypy.

***Daochus mandibularis* Champion, 1886** GUA BEL

*Daochus mandibularis* Champion, 1886: 140.

**Genus *Dioedus* LeConte, 1862** [M]

*Dioedus* LeConte, 1862a: 238. Type species: *Dioedus punctatus* LeConte, 1862, monotypy.

*Arrhabaeus* Champion, 1886: 144. Type species: *Arrhabaeus convexus* Champion, 1886, monotypy. Synonymy: Kaszab (1977b: 314).

***Dioedus convexus* (Champion, 1886)** CRI PAN

*Arrhabaeus convexus* Champion, 1886: 145.

***Dioedus debilis* (Champion, 1896)** LAN

*Arrhabaeus debilis* Champion, 1896: 20.

***Dioedus guadeloupensis* (Fleutiaux and Sallé, 1890)** LAN

*Arrhabaeus guadeloupensis* Fleutiaux and Sallé, 1890: 424.

***Dioedus minor* (Fleutiaux and Sallé, 1890)** LAN

*Arrhabaeus guadeloupensis minor* Fleutiaux and Sallé, 1890: 425.

**Figure 7.**
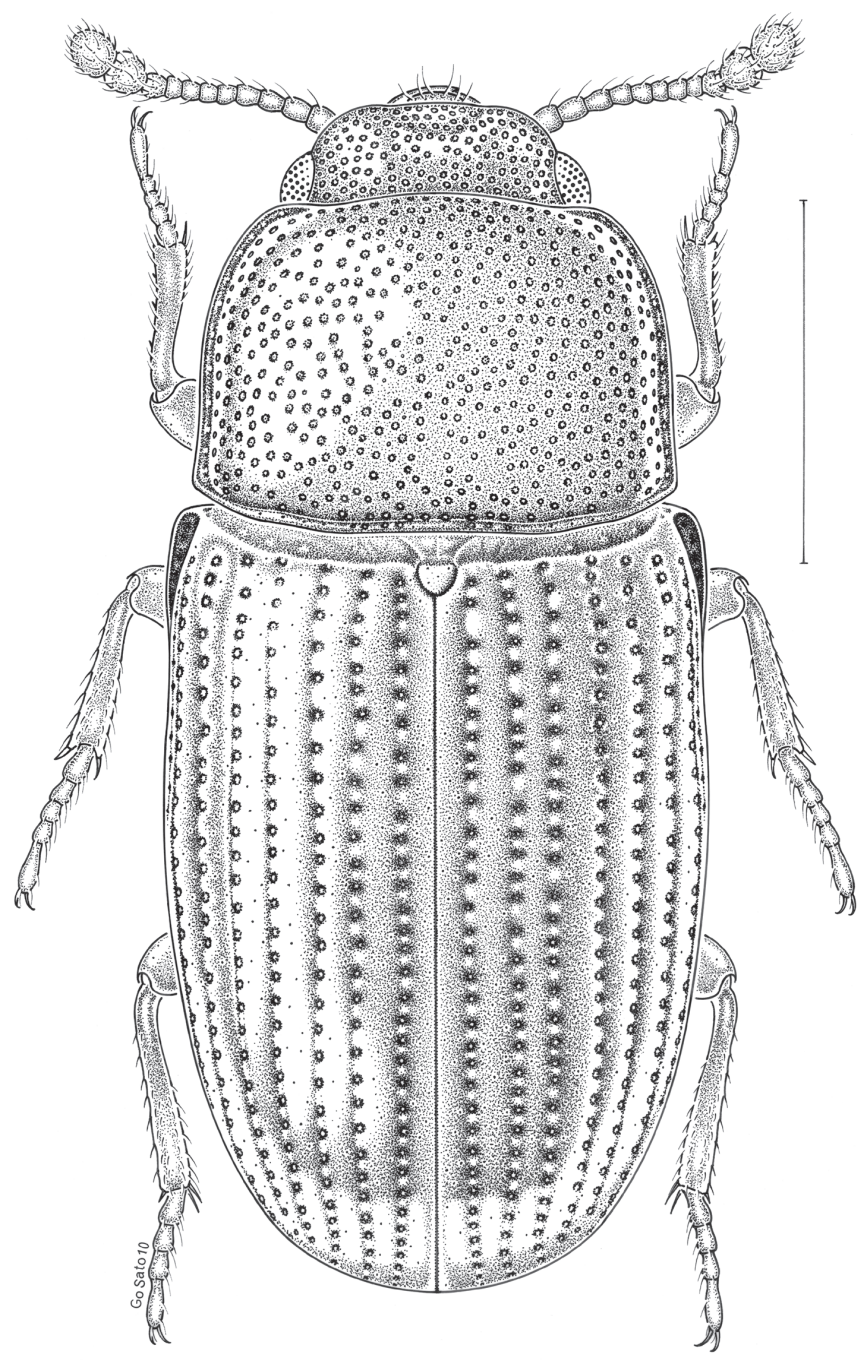
*Dioedus punctatus* LeConte, 1862. Scale bar = 1 mm.

***Dioedus punctatus* LeConte, 1862** [Fig. 7] CAN (ON) USA (AL CT DC FL IA IL IN KS MD MI MO NC NJ NY OH SC VA WI WV) / PRI

*Dioedus punctatus* LeConte, 1862a: 238.

**Genus *Peneta* Lacordaire, 1859** [F]

*Peneta* Lacordaire, 1859: 319. Type species: *Peneta lebasii* Lacordaire, 1859, subsequent designation (Lucas 1920: 492).

***Peneta costaricensis* Gebien, 1928** CRI

*Peneta costaricensis* Gebien, 1928a: 147.

***Peneta nevermanni* Gebien, 1928** CRI

*Peneta nevermanni* Gebien, 1928a: 148.

***Peneta nuchicornis* Gebien, 1928** CRI PAN

*Peneta nuchicornis* Gebien, 1928a: 146.

***Peneta obtusicornis* Kirsch, 1866** PAN / SA

*Peneta obtusicornis* Kirsch, 1866: 191.

*Peneta panamensis* Champion, 1886: 142. Synonymy: Champion (1893a: 531).

**Genus *Telchis* Champion, 1886** [M]

*Telchis* Champion, 1886: 142. Type species: *Telchis clavicornis* Champion, 1886, monotypy.

***Telchis clavicornis* Champion, 1886** PAN

*Telchis clavicornis* Champion, 1886: 143.

**Genus *Zypoetes* Champion, 1893** [M]

*Zypoetes* Champion, 1893a: 532. Type species: *Zypoetes epieroides* Champion, 1893, monotypy.

***Zypoetes epieroides* Champion, 1893** MEX (VE) GUA BEL NIC

*Zypoetes epieroides* Champion, 1893a: 533.

**Tribe Phrenapatini Solier, 1834**

Phrépatides Solier, 1834: 488. Type genus: *Phrenapates* Gray, 1832.

**Genus *Delognatha* Lacordaire, 1859** [F]

*Delognatha* Lacordaire, 1859: 315. Type species: *Delognatha lacordairei* Lacordaire, 1859, subsequent designation (Gebien 1940: 756). Note. The name *Delognatha* Agassiz, 1846 has been suppressed for the purposes of both the Principle of Priority and the Principle of Homonymy in Opinion 2250 (ICZN 2010).

***Delognatha persimilis* Gebien, 1928** CRI

*Delognatha persimilis* Gebien, 1928a: 142.

**Genus *Phrenapates* Gray, 1832** [M]

*Phrenapates* Gray [in Griffith and Pidgeon], 1832: 91. Type species: *Phrenapates bennettii* Gray, 1832, monotypy.

***Phrenapates bennettii* Gray, 1832** GUA NIC CRI PAN / SA

*Phrenapates bennettii* Gray [in Griffith and Pidgeon], 1832: 91.

**Subfamily PIMELIINAE Latreille, 1802**

Pimeliariae Latreille, 1802: 166. Type genus: *Pimelia* Fabricius, 1775.

**Tribe Anepsiini LeConte, 1862**

Anepsiini LeConte, 1862a: 215. Type genus: *Anepsius* LeConte, 1851.

Batuliini Horn, 1870: 270. Type genus: *Batulius* LeConte, 1851.

Anchommini Horn, 1878b: 558. Type genus: *Anchomma* LeConte, 1858.

**Genus *Anchomma* LeConte, 1858** [N]

*Anchomma* LeConte, 1858b: 63. Type species: *Anchomma costatum* LeConte, 1858, monotypy.

***Anchomma costatum* LeConte, 1858** USA (CA)

*Anchomma costatum* LeConte, 1858b: 63.

**Genus *Anepsius* LeConte, 1851** [M]

*Anepsius* LeConte, 1851: 147. Type species: *Anepsius delicatulus* LeConte, 1851, monotypy.

***Anepsius delicatulus* LeConte, 1851** USA (AZ CA NV UT) MEX (SO)

*Anepsius delicatulus* LeConte, 1851: 148.

*Anepsius catenulosus* Casey, 1907: 505. Synonymy (with *A. atratus* Casey): Casey (1911: 254).

*Anepsius atratus* Casey, 1907: 506. Synonymy: Doyen (1987: 351).

*Anepsius brunneus* Casey, 1907: 506. Synonymy: Doyen (1987: 351).

*Anepsius nebulosus* Casey, 1907: 507. Synonymy: Doyen (1987: 351).

*Anepsius bicolor* Casey, 1907: 507. Synonymy: Doyen (1987: 351).

*Anepsius deficiens* Casey, 1907: 507. Synonymy: Doyen (1987: 351).

***Anepsius minutus* Doyen, 1987** USA (TX) MEX (NL)

*Anepsius minutus* Doyen, 1987: 352.

**Figure 8.**
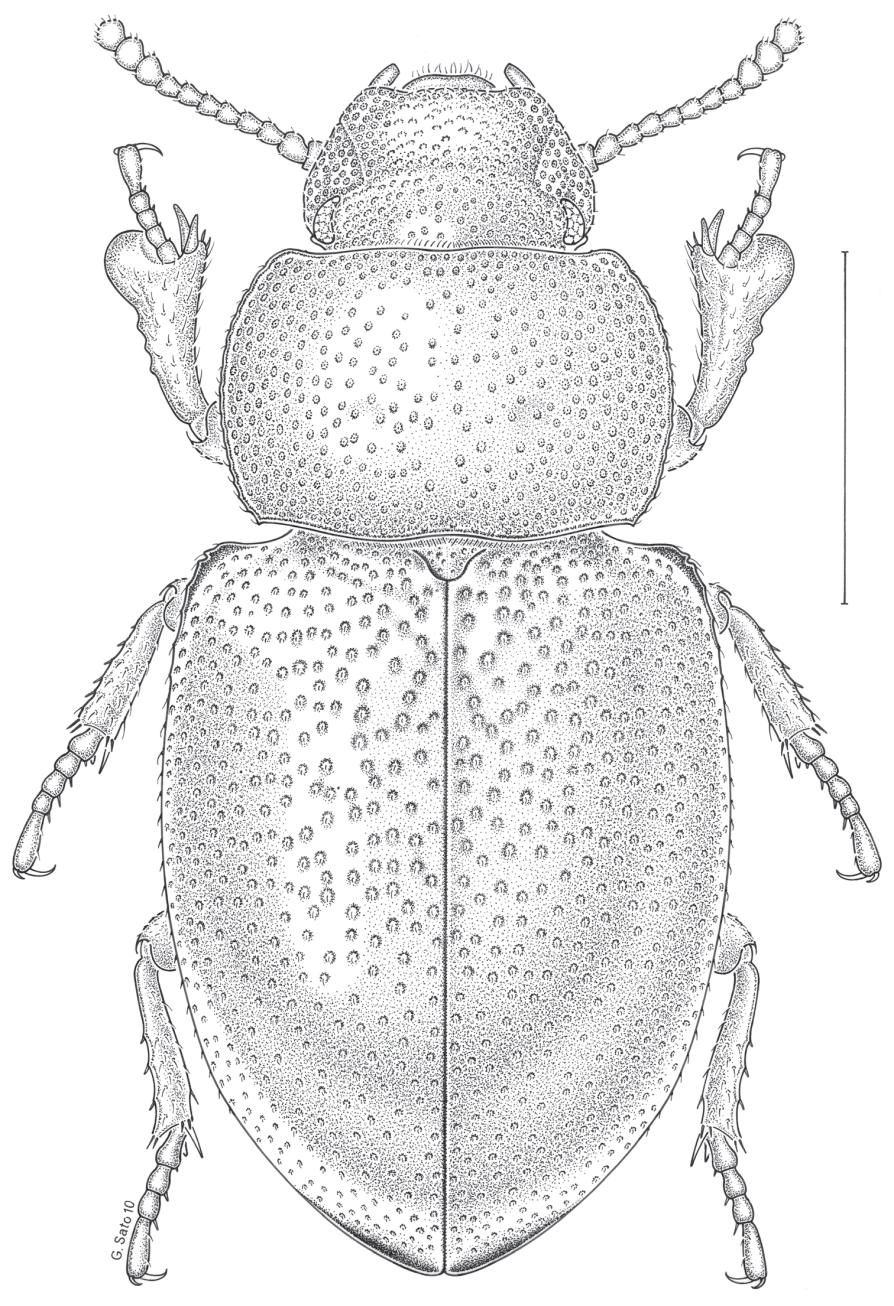
*Anepsius montanus* Casey, 1891. Scale bar = 1 mm.

***Anepsius montanus* Casey, 1891** [Fig. 8] CAN (AB) USA (CO ND NE NM WY)

*Anepsius montanus* Casey, 1891: 55.

***Anepsius valens* Casey, 1907** USA (AZ)

*Anepsius valens* Casey, 1907: 504.

**Genus *Batuliodes* Casey, 1907** [M]

*Batuliodes* Casey, 1907: 499. Type species: *Batulius rotundicollis* LeConte, 1851, original designation.

***Batuliodes confluens* (Blaisdell, 1923)** MEX (BC BS)

*Anepsius confluens* Blaisdell, 1923: 243.

*Anepsius angulatus* Blaisdell, 1923: 244. Synonymy: Doyen (1987: 366).

***Batuliodes obesus* Doyen, 1987** USA (CA)

*Batuliodes obesus* Doyen, 1987: 369.

***Batuliodes rotundicollis* (LeConte, 1851)** USA (AZ CA NV)

*Batulius rotundicollis* LeConte, 1851: 148.

***Batuliodes spatulatus* Doyen, 1987** USA (AZ CA UT) MEX (SO)

*Batuliodes spatulatus* Doyen, 1987: 368.

***Batuliodes wasbaueri* Doyen, 1987** USA (CA) MEX (BC)

*Batuliodes wasbaueri* Doyen, 1987: 367.

**Genus *Batuliomorpha* Doyen, 1987** [F]

*Batuliomorpha* Doyen, 1987: 359. Type species: *Batuliomorpha comata* Doyen, 1987, original designation.

***Batuliomorpha comata* Doyen, 1987** USA (AZ CA)

*Batuliomorpha comata* Doyen, 1987: 361.

***Batuliomorpha imperialis* Doyen, 1987** USA (CA)

*Batuliomorpha imperialis* Doyen, 1987: 359.

***Batuliomorpha tibiodentata* Doyen, 1987** MEX (BS)

*Batuliomorpha tibiodentata* Doyen, 1987: 362.

**Genus *Batulius* LeConte, 1851** [M]

*Batulius* LeConte, 1851: 148. Type species: *Batulius setosus* LeConte, 1851, subsequent designation (Casey 1907: 497).

***Batulius setosus* LeConte, 1851** USA (AZ CA) MEX (BC)

*Batulius setosus* LeConte, 1851: 148.

**Tribe Asidini Fleming, 1821**

Asidadae Fleming, 1821: 51. Type genus: *Asida* Latreille, 1802.

Astroti Horn, 1870: 289. Type genus: *Astrotus* J.L. LeConte, 1858.

Craniotini LeConte and Horn, 1883: 361. Type genus: *Craniotus* LeConte, 1851.

**Genus *Ardamimicus* Smith, 2013** [M]

*Ardamimicus* Smith, 2013: 601. Type species: *Ardamimicus cognatoi* Smith, 2013, original designation.

***Ardamimicus cognatoi* Smith, 2013** USA (TX) MEX (CH DU)

*Ardamimicus cognatoi* Smith, 2013: 602.

**Genus *Craniotus* LeConte, 1851** [M]

*Craniotus* LeConte, 1851: 142. Type species: *Craniotus pubescens* LeConte, 1851, monotypy.

***Craniotus mardecortesi* Aalbu, Smith and Sánchez Piñero, 2015** MEX (BC)

*Craniotus mardecortesi* Aalbu, Smith and Sánchez Piñero, 2015: 94.

***Craniotus pubescens* LeConte, 1851** USA (AZ CA NV) MEX (BC)

*Craniotus pubescens* LeConte, 1851: 142.

*Craniotus blaisdelli* Tanner, 1963: 169. Synonymy: Aalbu et al. (2015: 96).

***Craniotus triplehorni* Aalbu, Smith and Sánchez Piñero, 2015** MEX (BC)

*Craniotus triplehorni* Aalbu, Smith and Sánchez Piñero, 2015: 95.

**Genus *Ferveoventer* Smith, 2013** [M]

*Ferveoventer* Smith, 2013: 604. Type species: *Ferveoventer browni* Smith, 2013, original designation.

***Ferveoventer browni* Smith, 2013** USA (NM TX)

*Ferveoventer browni* Smith, 2013: 605.

***Ferveoventer planatus* (Champion, 1884)** MEX (MO)

*Ologlyptus planatus* Champion, 1884: 69.

**Genus *Heterasida* Casey, 1912** [F]

*Heterasida* Casey, 1912: 76, 165. Type species: *Pelecyphorus bifurcus* LeConte, 1861, original designation.

***Heterasida bifurcus* (LeConte, 1861)** ^10^ MEX (BS)

*Pelecyphorus bifurcus* LeConte, 1861a: 337.

***Heterasida connivens* (LeConte, 1866)** MEX (BS)

*Pelecyphorus connivens* LeConte, 1866b: 110.

*Heterasida tantilla* Casey, 1912: 167. Synonymy: Smith (2013: 607).

*Heterasida exilis* Casey, 1912: 168. Synonymy: Smith (2013: 607).

**Genus *Litasida* Casey, 1912** [F]

*Litasida* Casey, 1912: 77, 184. Type species: *Litasida townsendi* Casey, 1912, original designation.

***Litasida townsendi* Casey, 1912** USA (AZ) MEX (CH)

*Litasida townsendi* Casey, 1912: 185.

**Genus *Micrasida* Smith, 2013** [F]

*Micrasida* Smith, 2013: 608. Type species: *Micrasida obrienorum* Smith, 2013, original designation.

***Micrasida obrienorum* Smith, 2013** MEX (NL)

*Micrasida obrienorum* Smith, 2013: 608.

**Genus *Microschatia* Solier, 1836** [F]

*Microschatia* Solier, 1836: 474. Type species: *Microschatia punctata* Solier, 1836, monotypy.

*Pycnonotida* Casey, 1912: 75, 89. Type species: *Microschatia inaequalis* LeConte, 1851, original designation. Synonymy: Brown and Doyen (1992: 546).

*Acroschatia* Wilke, 1922: 269. Type species: *Microschatia robusta* Horn, 1893, original designation. Synonymy: Brown and Doyen (1992: 546).

***Microschatia cedrosensis* Brown and Doyen, 1992** MEX (BS)

*Microschatia cedrosensis* Brown and Doyen, 1992: 568.

***Microschatia championi* Horn, 1893** USA (AZ CA) MEX (BC BS)

*Microschatia championi* Horn, 1893: 140.

***Microschatia costulata* Brown and Doyen, 1992** USA (CA) MEX (BC)

*Microschatia costulata* Brown and Doyen, 1992: 570.

***Microschatia inaequalis* LeConte, 1851** USA (CA) MEX (BC)

*Microschatia inaequalis* LeConte, 1851: 129.

*Microschatia puncticollis* LeConte, 1851: 129. Synonymy: Horn (1870: 282).

*Pycnonotida laxicollis* Casey, 1912: 91. Synonymy: Brown and Doyen (1992: 572).

*Pycnonotida araneoides* Casey, 1912: 92. Synonymy: Brown and Doyen (1992: 572).

*Pycnonotida inaequalis diversa* Casey, 1912: 92. Synonymy: Brown and Doyen (1992: 572).

*Pycnonotida impar* Casey, 1912: 93. Synonymy: Brown and Doyen (1992: 572).

***Microschatia morata* Horn, 1878** USA (AZ NM) MEX (CH DU SO)

*Microschatia morata* Horn, 1878a: 56.

***Microschatia planata* Doyen and Brown, 1992** MEX (BC BS)

*Microschatia planata* Doyen and Brown [in Brown and Doyen], 1992: 576.

***Microschatia polita* Horn, 1893** USA (AZ)

*Microschatia polita* Horn, 1893: 141.

***Microschatia punctata* Solier, 1836** MEX (HI QU)

*Microschatia punctata* Solier, 1836: 475.

***Microschatia robusta* Horn, 1893** USA (TX) MEX (CH CO NL TA)

*Microschatia robusta* Horn, 1893: 142.

***Microschatia rockefelleri* Pallister, 1954** MEX (CH DU)

*Microschatia rockefelleri* Pallister, 1954: 15.

***Microschatia solieri* Brown and Doyen, 1992** MEX (HI)

*Microschatia solieri* Brown and Doyen, 1992: 552.

***Microschatia sulcipennis* LeConte, 1858** USA (TX)

*Microschatia sulcipennis* LeConte, 1858a: 18.

**Genus *Pelecyphorus* Solier, 1836** [M]

*Pelecyphorus* Solier, 1836: 467. Type species: *Pelecyphorus mexicanus* Solier, 1836, subsequent designation (Hope 1841: 110).

**Subgenus Astrotus LeConte, 1858**

*Astrotus* LeConte, 1858a: 19. Type species: *Microschatia contorta* LeConte, 1853, original designation.

***Pelecyphorus alveolatus* (Casey, 1912)** USA (TX)

*Astrotus alveolatus* Casey, 1912: 83.

***Pelecyphorus contortus* (LeConte, 1853)** USA (TX)

*Microschatia contorta* LeConte, 1853: 446.

***Pelecyphorus fasciculatus* (Champion, 1892)** MEX (MO)

*Asida fasciculata* Champion, 1892: 495.

***Pelecyphorus guanajuatensis* (Champion, 1884)** MEX (CH DU GU)

*Asida guanajuatensis* Champion, 1884: 56.

*Bothrasida mucorea* Wilke, 1922: 270. **New synonymy** [ADS].

***Pelecyphorus hebes* (Champion, 1892)** MEX (DU)

*Ologlyptus hebes* Champion, 1892: 506.

***Pelecyphorus regularis* (Horn, 1870)** USA (TX) MEX (TA)

*Astrotus regularis* Horn, 1870: 290.

**Subgenus Pelecyphorus Solier, 1836**

*Pelecyphorus* Solier, 1836: 467. Type species: *Pelecyphorus mexicanus* Solier, 1836, subsequent designation (Hope 1841: 110).

***Pelecyphorus mexicanus* Solier, 1836** MEX (PU QU VE)

*Pelecyphorus mexicanus* Solier, 1836: 469.

**Subgenus Pleisiasida Smith, 2013**

*Parasida* Casey, 1912: 76, 126 [junior homonym of *Parasida* Daday, 1904]. Type species: *Parasida laciniata* Casey, 1912, original designation.

*Pleisiasida* Smith, 2013: 610. Replacement name for *Parasida* Casey, 1912.

***Pelecyphorus asidoides* Solier, 1836** MEX (HI ME)^11^

*Pelecyphorus asidoides* Solier, 1836: 471.

*Parasida zacualpanicola* Wilke, 1922: 272. **New synonymy** [ADS].

***Pelecyphorus bibasalis* (Casey, 1912)** MEX (DU)

*Parasida bibasalis* Casey, 1912: 128.

***Pelecyphorus dispar* (Champion, 1892)** MEX (CH DU)

*Asida dissimilis* Champion, 1884: 59 [junior primary homonym of *Asida dissimilis* Allard, 1869].

*Asida dispar* Champion, 1892: 496. Replacement name for *Asida dissimilis* Champion, 1884.

*Stenosides kulzeri* Pallister, 1954: 12. **New synonymy** [ADS].

*Stenosides bisinuatus* Pallister, 1954: 13. **New synonymy** [ADS].

*Parasida trisinuata* Pallister, 1954: 22. **New synonymy** [ADS].

***Pelecyphorus fallax* (Champion, 1884)** MEX (DU FD GU HI ME PU)

*Asida fallax* Champion, 1884: 57.

*Asida favosa* Champion, 1884: 58. **New synonymy** [ADS].

*Asida similata* Champion, 1884: 58. **New synonymy** [ADS].

***Pelecyphorus foveolatus* Solier, 1836** MEX (OA VE)

*Pelecyphorus foveolatus* Solier, 1836: 472.

***Pelecyphorus indutus* (Champion, 1884)** MEX (OA)

*Asida induta* Champion, 1884: 56.

*Ologlyptus bicarinatus* Champion, 1884: 69. **New synonymy** [ADS].

***Pelecyphorus intricatus* (Champion, 1892)** MEX (CH JA)

*Asida intricata* Champion, 1892: 493.

***Pelecyphorus laticollis* (Champion, 1884)** MEX (DU GU)

*Asida laticollis* Champion, 1884: 58.

***Pelecyphorus liratus* (LeConte, 1854)** USA (AZ NM) MEX (CH DU)

*Euschides liratus* LeConte, 1854c: 223.

*Parasida laciniata* Casey, 1912: 128. **New synonymy** [ADS].

*Parasida cristata* Pallister, 1954: 24. **New synonymy** [ADS].

***Pelecyphorus longipennis* (Champion, 1884)** MEX (OA PU VE)

*Asida longipennis* Champion, 1884: 56.

*Parasida esperanzae* Wilke, 1922: 271. **New synonymy** [ADS].

*Parasida mixtecae* Wilke, 1922: 271. **New synonymy** [ADS].

***Pelecyphorus obliviosus* (Wilke, 1922)** MEX (CH DU)

*Parasida obliviosa* Wilke, 1922: 270.

***Pelecyphorus planatulus* (Casey, 1912)** MEX (DU)

*Parasida planatula* Casey, 1912: 129.

***Pelecyphorus scutellaris* (Champion, 1884)** MEX (DU FD ME OA PU VE)

*Asida scutellaris* Champion, 1884: 56.

*Parasida tolucana* Casey, 1912: 130. **New synonymy** [ADS].

***Pelecyphorus sexcostatus* LeConte, 1861** MEX (BS)

*Pelecyphorus sexcostatus* LeConte, 1861a: 337.

***Pelecyphorus tristis* (Champion, 1884)** MEX (PU VE)

*Asida tristis* Champion, 1884: 55.

*Parasida purpusi* Wilke, 1922: 271. **New synonymy** [ADS].

**Subgenus Poliorcetes Champion, 1884**

*Poliorcetes* Champion, 1884: 70. Type species: *Poliorcetes platesthoides* Champion, 1884, monotypy.

***Pelecyphorus platesthoides* (Champion, 1884)** MEX (OA)

*Poliorcetes platesthoides* Champion, 1884: 71.

**Subgenus Sicharbas Champion, 1884**

*Sicharbas* Champion, 1884: 67. Type species: *Sicharbas lobatus* Champion, 1884, monotypy.

***Pelecyphorus debilis* (Champion, 1884)** MEX (PU)

*Astrotus debilis* Champion, 1884: 66.

***Pelecyphorus erosus* (Champion, 1892)** MEX (HI)

*Astrotus erosus* Champion, 1892: 504.

*Astrotus nosodermoides* Champion, 1892: 505. **New synonymy** [ADS].

***Pelecyphorus lobatus* (Champion, 1884)** MEX (GE MO)

*Sicharbas lobatus* Champion, 1884: 67.

***Pelecyphorus seticornis* (Champion, 1884)** MEX (ME)

*Astrotus seticornis* Champion, 1884: 67.

Astrotus seticornis var. humeralis Champion, 1884: 67. **New synonymy** [ADS].

***Pelecyphorus undatus* (Champion, 1892)** MEX (DU)

*Astrotus undatus* Champion, 1892: 504.

**Subgenus Stenosides Solier, 1836**

*Stenosides* Solier, 1836: 484. Type species: *Stenosides graciliformis* Solier, 1836, monotypy.

*Pactostoma* LeConte, 1858a: 19. Type species: *Asida anastomosis* Say, 1824, original designation. Synonymy (with *Ologlyptus* Lacordaire): LeConte (1862a: 222).

*Ologlyptus* Lacordaire, 1859: 158. Unnecessary replacement name for *Stenosides* Solier, 1836.

***Pelecyphorus anastomosis* (Say, 1824)** USA (AR AZ CO KS NM TX) MEX (CH DU)

*Asida anastomosis* Say, 1824a: 256.

*Pactostoma anastomosis salebrosa* Casey, 1912: 87. Synonymy: Pallister (1954: 12).

*Pactostoma breviuscula* Casey, 1912: 87. **New synonymy** [ADS].

*Pactostoma exoleta* Casey, 1912: 87. **New synonymy** [ADS].

*Pactostoma luteotecta* Casey, 1912: 88. **New synonymy** [ADS].

*Pactostoma monticola* Casey, 1912: 88. **New synonymy** [ADS].

*Pactostoma obtecta* Casey, 1912: 89. **New synonymy** [ADS].

*Pactostoma sigillata* Casey, 1912: 89. **New synonymy** [ADS].

***Pelecyphorus graciliformis* (Solier, 1836)** MEX (FD HI OA PU SL)

*Stenosides graciliformis* Solier, 1836: 486.

*Ologlyptus canus* Champion, 1884: 68. **New synonymy** [ADS].

*Ologlyptus sinuaticollis* Champion, 1884: 69. **New synonymy** [ADS].

***Pelecyphorus limosus* (Champion, 1884)** MEX

*Astrotus limosus* Champion, 1884: 66.

***Pelecyphorus texanus* (Wickham, 1903)** USA (TX)

*Ologlyptus texanus* Wickham, 1903: 72.

**Subgenus Ucalegon Champion, 1884**

*Ucalegon* Champion, 1884: 65. Type species: *Ucalegon pulchellus* Champion, 1884, monotypy.

***Pelecyphorus pulchellus* (Champion, 1884)** MEX (GE OA)

*Ucalegon pulchellus* Champion, 1884: 65.

**Subgenus Zaleucus Champion, 1892**

*Zamolxis* Champion, 1884: 70 [junior homonym of *Zamolxis* Stål, 1865]. Type species: *Zamolxis dilatatus* Champion, 1884, monotypy.

*Zaleucus* Champion, 1892: 491. Replacement name for *Zamolxis* Champion, 1884.

***Pelecyphorus dilatatus* (Champion, 1884)** MEX (PU)

*Zamolxis dilatatus* Champion, 1884: 70.

**Genus *Philolithus* Lacordaire, 1858** [M]

*Philolithus* Lacordaire [in LeConte], 1858a: 18. Type species: *Pelecyphorus carinatus* LeConte, 1851, subsequent designation (Casey 1912: 79).

**Subgenus Glyptasida Casey, 1912**

*Glyptasida* Casey, 1912: 75, 95. Type species: *Pelecyphorus sordidus* LeConte, 1853, original designation.

***Philolithus aeger* (LeConte, 1858)** USA (NM TX)

*Pelecyphorus aeger* LeConte, 1858a: 19.

*Glyptasida sycophanta* Casey, 1912: 104. Synonymy: Lockwood and Pollock (2009: 21).

***Philolithus rugosissimus* (Champion, 1884)** USA (AZ NM) MEX (CH CO SL)

*Asida rugosissima* Champion, 1884: 53.

**Figure 9.**
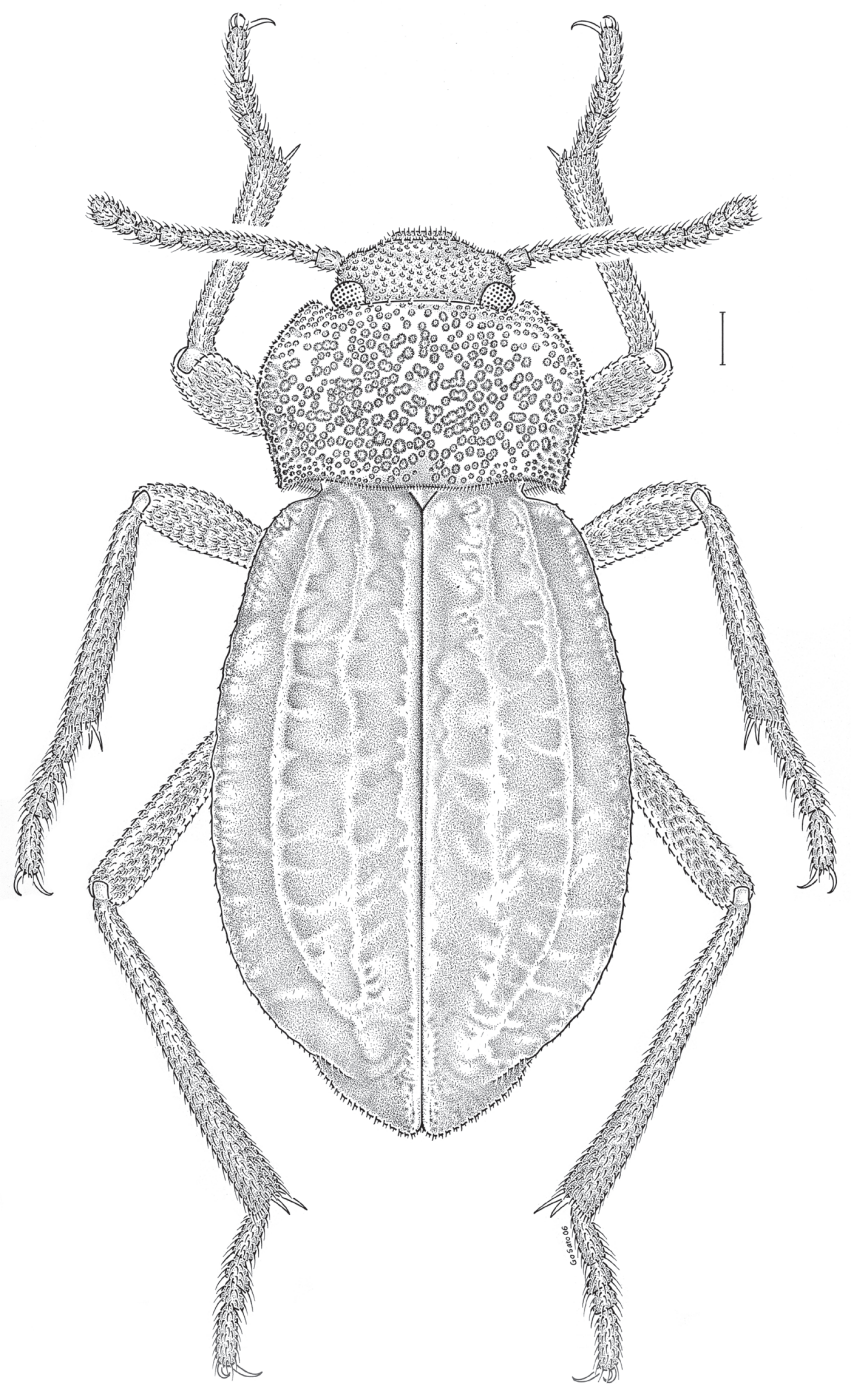
Philolithus (Glyptasida) sordidus (LeConte, 1853). Scale bar = 1 mm.

***Philolithus sordidus* (LeConte, 1853)** [Fig. 9] CAN (AB SK) USA (AZ CO KS MT ND NE NM OK SD TX UT WY) MEX (CH CO DU ZA)

*Pelecyphorus sordidus* LeConte, 1853: 445.

*Pelecyphorus subcostatus* LeConte, 1853: 446. Synonymy: Henshaw (1882: 255).

*Pelecyphorus irregularis* LeConte, 1858a: 19. Synonymy: Champion (1892: 492).

*Pelecyphorus costipennis* LeConte, 1858a: 20. Synonymy: Champion (1892: 492).

*Asida interrupta* Champion, 1884: 53. Synonymy: Champion (1892: 492).

*Glyptasida parvicollis* Casey, 1912: 97. Synonymy: Lockwood and Pollock (2009: 8).

*Glyptasida sordida porcatula* Casey, 1912: 97. Synonymy: Lockwood and Pollock (2009: 8).

*Glyptasida subpubescens* Casey, 1912: 98. Synonymy: Lockwood and Pollock (2009: 8).

*Glyptasida turgescens* Casey, 1912: 98. Synonymy: Lockwood and Pollock (2009: 8).

*Glyptasida turgescens furtiva* Casey, 1912: 99. Synonymy: Lockwood and Pollock (2009: 8).

*Glyptasida turgescens obesa* Casey, 1912: 99. Synonymy: Lockwood and Pollock (2009: 8).

*Glyptasida procrustes* Casey, 1912: 99. Synonymy: Lockwood and Pollock (2009: 8).

*Glyptasida costipennis fulvisetis* Casey, 1912: 100. Synonymy: Lockwood and Pollock (2009: 8).

*Glyptasida strigipennis* Casey, 1912: 100. Synonymy: Lockwood and Pollock (2009: 8).

*Glyptasida turbulenta* Casey, 1912: 101. Synonymy: Lockwood and Pollock (2009: 8).

*Glyptasida aegra imperfecta* Casey, 1912: 102. Synonymy: Lockwood and Pollock (2009: 7).

*Glyptasida aegra pigra* Casey, 1912: 102. Synonymy: Lockwood and Pollock (2009: 8).

*Glyptasida aegra plena* Casey, 1912: 102. Synonymy: Lockwood and Pollock (2009: 8).

*Glyptasida heres* Casey, 1912: 103. Synonymy: Lockwood and Pollock (2009: 8).

*Glyptasida crenicollis* Casey, 1912: 103. Synonymy: Lockwood and Pollock (2009: 8).

**Subgenus Gonasida Casey, 1912**

*Gonasida* Casey, 1912: 75, 117. Type species: *Pelecyphorus elatus* LeConte, 1853, original designation.

***Philolithus elatus compar* (Casey, 1912)** USA (CO ID KS NE NM NV OR UT WY)

*Gonasida compar* Casey, 1912: 120.

*Gonasida elata reducta* Casey, 1912: 121. **New synonymy** [based on Brown (1971: 262) unpublished thesis].

*Gonasida elata prolixa* Casey, 1912: 121. **New synonymy** [based on Brown (1971: 262) unpublished thesis].

*Gonasida aucta* Casey, 1912: 122. **New synonymy** [based on Brown (1971: 262) unpublished thesis].

***Philolithus elatus difformis* (LeConte, 1854)** USA (AZ NM UT) MEX (CH)

*Pelecyphorus difformis* LeConte, 1854c: 223.

*Gonasida alaticollis* Casey, 1912: 122. **New synonymy** [based on Brown (1971: 255) unpublished thesis].

***Philolithus elatus elatus* (LeConte, 1853)** USA (AZ NM TX) MEX (CH)

*Pelecyphorus elatus* LeConte, 1853: 445.

*Gonasida gravida* Casey, 1912: 119. **New synonymy** [based on Brown (1971: 251) unpublished thesis].

***Philolithus elatus infernus* (Casey, 1912)** USA (AZ NM UT)

*Gonasida inferna* Casey, 1912: 119.

**Subgenus Herthasida Wilke, 1922**

*Herthasida* Wilke, 1922: 269. Type species: *Asida ingens* Champion, 1892, monotypy.

***Philolithus ingens* (Champion, 1892)** MEX (CO)

*Asida ingens* Champion, 1892: 503.

**Subgenus Philolithus Lacordaire, 1858**

*Philolithus* Lacordaire [in LeConte], 1858a: 18. Type species: *Pelecyphorus carinatus* LeConte, 1851, subsequent designation (Casey 1912: 79).

***Philolithus actuosus* (Horn, 1870)** USA (CA)

*Asida actuosa* Horn, 1870: 284.

***Philolithus adversus* (Casey, 1912)** USA (AZ)

*Pelecyphorus adversus* Casey, 1912: 114.

***Philolithus aegrotus aegrotus* (LeConte, 1861)** MEX (BC)

*Pelecyphorus aegrotus* LeConte, 1861a: 337.

*Pelecyphorus aegrotus limbatus* Casey, 1912: 107. **New synonymy** [ADS].

***Philolithus carinatus* (LeConte, 1851)** USA (CA)

*Pelecyphorus carinatus* LeConte, 1851: 128 [junior secondary homonym of *Asida carinata* Solier, 1836].

*Asida carinifera* Gebien, 1910a: 128. Replacement name for *Asida carinata* (LeConte, 1851)^12^.

**Figure 10.**
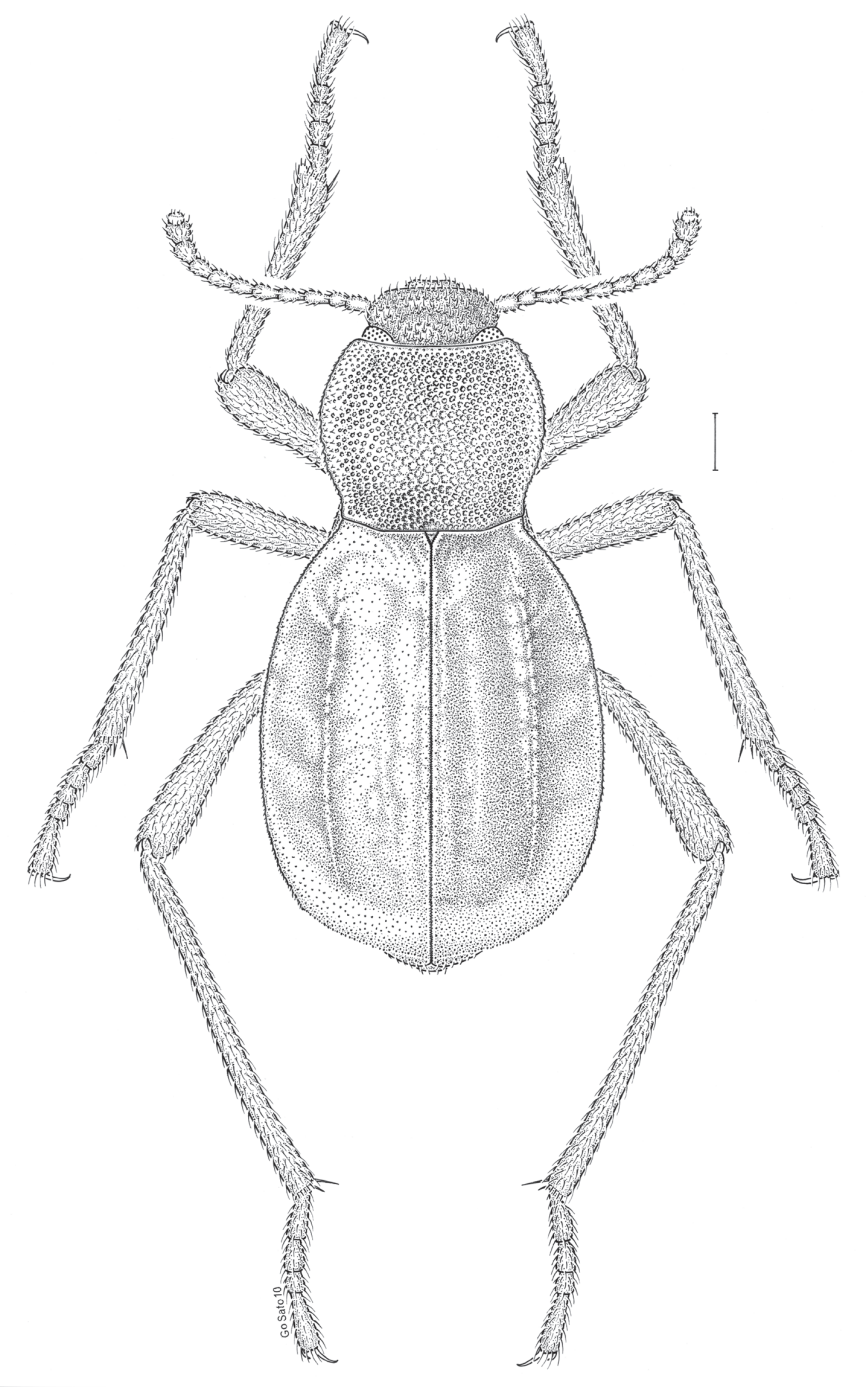
Philolithus (Philolithus) densicollis (Horn, 1894). Scale bar = 1 mm.

***Philolithus densicollis* (Horn, 1894)** [Fig. 10] CAN (BC) USA (OR WA)

*Asida densicollis* Horn, 1894b: 417.

*Pelecyphorus corrosus* Casey, 1912: 117. Synonymy: Boddy (1965: 137).

***Philolithus haruspex ellipsipennis* (Casey, 1912)** USA (UT)

*Pelecyphorus haruspex ellipsipennis* Casey, 1912: 116.

***Philolithus haruspex haruspex* (Casey, 1912)** USA (AZ ID OR NV UT)

*Pelecyphorus haruspex* Casey, 1912: 115.

***Philolithus jaegeri* (Papp, 1961)** USA (CA)

*Pelecyphorus jaegeri* Papp, 1961b: 107.

***Philolithus morbillosus* (LeConte, 1858)** USA (AZ) MEX (SO)

*Pelecyphorus morbillosus* LeConte, 1858b: 74.

*Pelecyphorus corporalis* Casey, 1912: 107. **New synonymy** [ADS].

*Pelecyphorus reptans* Casey, 1912: 108. **New synonymy** [ADS].

*Pelecyphorus socer* Casey, 1912: 108. **New synonymy** [ADS].

*Pelecyphorus abscissus* Casey, 1912: 109. **New synonymy** [ADS].

*Pelecyphorus fumosus* Casey, 1912: 109. **New synonymy** [ADS].

*Pelecyphorus parvus* Casey, 1912: 110. **New synonymy** [ADS].

*Pelecyphorus morbillosus pacatus* Casey, 1912: 110. **New synonymy** [ADS].

*Pelecyphorus morbillosus sobrius* Casey, 1912: 110. **New synonymy** [ADS].

*Pelecyphorus piceus* Casey, 1912: 111. **New synonymy** [ADS].

*Pelecyphorus piceus crudelis* Casey, 1912: 111. **New synonymy** [ADS].

*Pelecyphorus snowi* Casey, 1912: 111. **New synonymy** [ADS].

*Pelecyphorus subtenuis* Casey, 1912: 112. **New synonymy** [ADS].

***Philolithus opimus* (Casey, 1912)** USA (CA)

*Pelecyphorus opimus* Casey, 1912: 115.

***Philolithus pantex* (Casey, 1912)** USA (NV UT)

*Pelecyphorus pantex* Casey, 1912: 116.

***Philolithus porcatus* (Papp, 1961)** USA (CA)

*Pelecyphorus porcatus* Papp, 1961b: 109.

***Philolithus quadripennis* (Casey, 1912)** MEX (LC)

*Pelecyphorus quadripennis* Casey, 1912: 113.

***Philolithus reflexus* (Casey, 1912)** USA (CA)

*Pelecyphorus reflexus* Casey, 1912: 114.

***Philolithus rugosus* (Papp, 1961)** USA (CA)

*Pelecyphorus rugosus* Papp, 1961c: 157.

***Philolithus sophistes* (Casey, 1912)** USA (CA)

*Pelecyphorus sophistes* Casey, 1912: 113.

***Philolithus uteanus* (Casey, 1924)** USA (UT)

*Pelecyphorus uteanus* Casey, 1924: 308.

**Subgenus Tisamenes Champion, 1884**

*Tisamenes* Champion, 1884: 64. Type species: *Tisamenes truquii* Champion, 1884, monotypy.

***Philolithus truquii* (Champion, 1884)** MEX (FD HI)

*Tisamenes truquii* Champion, 1884: 64.

**Genus *Stenomorpha* Solier, 1836** [F]

*Stenomorpha* Solier, 1836: 487. Type species: *Stenomorpha blapsoides* Solier, 1836, subsequent designation (Desmarest 1860: 150).

*Euschides* LeConte, 1851: 127. Unnecessary replacement name for *Stenomorpha* Solier, 1836.

**Subgenus Asidina Casey, 1912**

*Asidina* Casey, 1912: 169. Type species: *Pelecyphorus parallelus* LeConte, 1851, original designation.

***Stenomorpha confluens* (LeConte, 1851)** USA (AZ CA) MEX (BC SO)

*Pelecyphorus confluens* LeConte, 1851: 128.

***Stenomorpha parallela* (LeConte, 1851)** USA (AZ CA) MEX (SO)

*Pelecyphorus parallelus* LeConte, 1851: 128 [junior secondary homonym of *Asida parallela* Solier, 1836].

*Asida neglecta* Gebien, 1910a: 134. Replacement name for *Asida parallela* (LeConte, 1851)^13^.

*Asidina teres* Casey, 1912: 171. Synonymy: Triplehorn and Brown (1971: 84).

*Asida parallela terricola* Blaisdell, 1923: 254. Synonymy: Triplehorn and Brown (1971: 84).

***Stenomorpha rugicollis* (Triplehorn and Brown, 1971)** USA (AZ)

*Asidina rugicollis* Triplehorn and Brown, 1971: 76.

***Stenomorpha semilaevis* (Horn, 1870)** USA (AZ CA NV)

*Asida semilaevis* Horn, 1870: 284.

**Subgenus Asidopsis Casey, 1912**

*Asidopsis* Casey, 1912: 77, 185. Type species: *Asida opaca* Say, 1824, original designation.

***Stenomorpha abbreviata* (Casey, 1912)** USA (NM)

*Asidopsis abbreviata* Casey, 1912: 198.

***Stenomorpha cochisensis* (Casey, 1912)** USA (AZ NM)

*Asidopsis cochisensis* Casey, 1912: 189.

***Stenomorpha coenosa* (Casey, 1912)** USA (NM)

*Asidopsis coenosa* Casey, 1912: 200.

***Stenomorpha collaris* (Champion, 1892)** MEX (AG GU JA)

*Asida marginicollis* Champion, 1884: 60 [junior primary homonym of *Asida marginicollis* Rosenhauer, 1856].

*Asida collaris* Champion, 1892: 499. Replacement name for *Asida marginicollis* Champion, 1884.

***Stenomorpha collega* (Casey, 1912)** USA (KS)

*Asidopsis collega* Casey, 1912: 198.

***Stenomorpha consentanea* (Casey, 1912)** USA (CA)

*Asidopsis consentanea* Casey, 1912: 192.

***Stenomorpha divaricata* (Blaisdell, 1923)** MEX (BS)

*Asida divaricata* Blaisdell, 1923: 255.

***Stenomorpha dolosa* (Casey, 1912)** USA (MT SD WY)

*Asidopsis dolosa* Casey, 1912: 194.

***Stenomorpha durangoensis* (Casey, 1912)** MEX (DU)

*Asidopsis durangoensis* Casey, 1912: 201.

***Stenomorpha eximia* (Casey, 1912)** USA (TX)

*Asidopsis eximia* Casey, 1912: 188.

***Stenomorpha forreri* (Champion, 1884)** MEX (DU)

*Asida forreri* Champion, 1884: 55.

***Stenomorpha gracilipes* (Casey, 1912)** USA (AZ)

*Asidopsis gracilipes* Casey, 1912: 189.

***Stenomorpha humeralis* (Triplehorn and Flores, 2002)** MEX (CH)

*Asidopsis humeralis* Triplehorn and Flores, 2002: 288.

***Stenomorpha immunda* (Casey, 1912)** USA (NM) MEX (DU)

*Asidopsis immunda* Casey, 1912: 199.

***Stenomorpha macra* (Horn, 1883)** USA (AZ NM)

*Asidopsis macra* Horn, 1883: 304.

***Stenomorpha mancipata* (Horn, 1878)** USA (NM) MEX (CH)

*Asida mancipata* Horn, 1878a: 56.

*Asidopsis woodgatei* Casey, 1912: 197. Synonymy: Triplehorn and Flores (2002: 285).

***Stenomorpha nitidula* (Casey, 1912)** USA (NM)

*Asidopsis nitidula* Casey, 1912: 196.

***Stenomorpha obsidiana* (Casey, 1912)** USA (CO)

*Asidopsis obsidiana* Casey, 1912: 193.

***Stenomorpha olsoni* (Triplehorn and Flores, 2002)** USA (AZ)

*Asidopsis olsoni* Triplehorn and Flores, 2002: 286.

**Figure 11.**
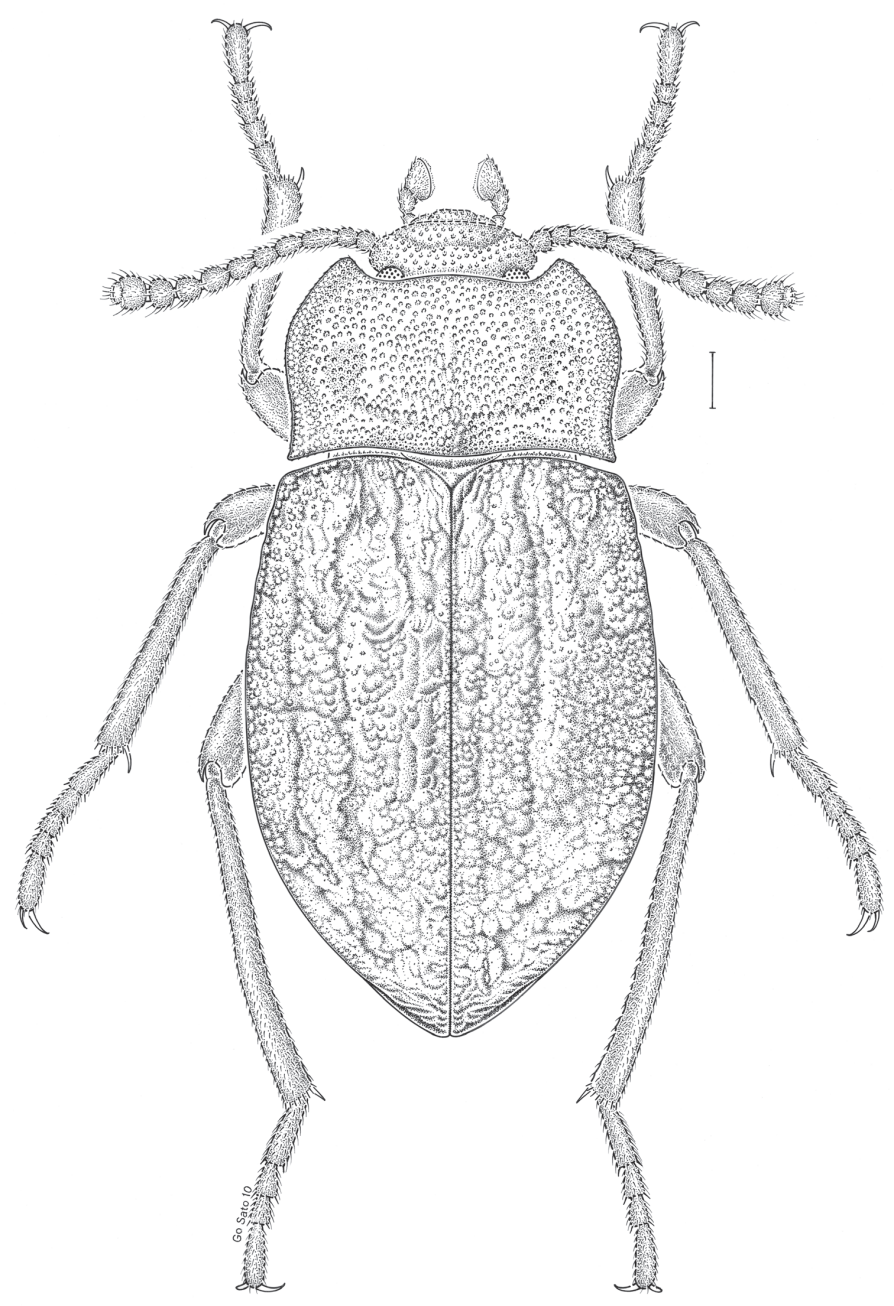
Stenomorpha (Asidopsis) opaca (Say, 1824). Scale bar = 1 mm.

***Stenomorpha opaca* (Say, 1824)** [Fig. 11] CAN (AB SK) USA (CO KS MT ND NE NM SD TX)

*Asida opaca* Say, 1824a: 254.

***Stenomorpha pinalica* (Casey, 1912)** USA (AZ)

*Asidopsis pinalica* Casey, 1912: 190.

***Stenomorpha planata* (Horn, 1894)** USA (CA) MEX

*Asida planata* Horn, 1894b: 415.

***Stenomorpha polita futilis* (Casey, 1912)** USA (KS)

*Asidopsis polita futilis* Casey, 1912: 194.

***Stenomorpha polita polita* (Say, 1824)** CAN (AB SK) USA (CO ID KS MT NE NM OK SD TX WY)

*Asida polita* Say, 1824a: 255.

***Stenomorpha polita subopaca* (Casey, 1912)** USA (KS)

*Asidopsis polita subopaca* Casey, 1912: 193.

***Stenomorpha quadricollis* (Horn, 1880)** USA (AZ NM)

*Asidopsis quadricollis* Horn, 1880: 151.

***Stenomorpha servilis* (Casey, 1912)** USA (CO)

*Asidopsis servilis* Casey, 1912: 199.

***Stenomorpha suavis* (Casey, 1912)** USA (AZ)

*Asidopsis suavis* Casey, 1912: 190.

***Stenomorpha tensa* (Casey, 1912)** USA (CO)

*Asidopsis tensa* Casey, 1912: 197.

**Subgenus Bothrasida Casey, 1912**

*Bothrasida* Casey, 1912: 76, 122. Type species: *Asida clathrata* Champion, 1884, original designation.

***Stenomorpha baroni* (Casey, 1912)** MEX (GE)

*Bothrasida baroni* Casey, 1912: 124.

***Stenomorpha clathrata* (Champion, 1884)** MEX (GE ME MO OA PU)

*Asida clathrata* Champion, 1884: 54.

***Stenomorpha funesta* (Champion, 1884)** MEX (PU)

*Asida funesta* Champion, 1884: 53.

*Bothrasida sanctae-agnae* Wilke, 1922: 270. **New synonymy** [ADS].

**Subgenus Megasida Casey, 1912**

*Megasida* Casey, 1912: 77, 202. Type species: *Asida obliterata* Champion, 1892, original designation.

***Stenomorpha foeda* (Champion, 1892)** MEX (CO DU)

*Asida foeda* Champion, 1892: 498.

***Stenomorpha latissima* (Champion, 1892)** MEX (DU)

*Asida latissima* Champion, 1892: 500.

***Stenomorpha magnifica* (Pallister, 1954)** MEX (DU)

*Megasida magnifica* Pallister, 1954: 30.

***Stenomorpha moricoides* (Champion, 1892)** MEX (CO DU)

*Asida moricoides* Champion, 1892: 497.

***Stenomorpha obliterata* (Champion, 1892)** USA (NM TX) MEX (CH)

*Asida obliterata* Champion, 1892: 497.

***Stenomorpha rufipes* (Champion, 1884)** MEX (SL)

*Asida rufipes* Champion, 1884: 62.

***Stenomorpha segregata* (Champion, 1892)** MEX (CH CO DU)

*Asida segregata* Champion, 1892: 497.

***Stenomorpha tarda* (Champion, 1892)** MEX (CO)

*Asida tarda* Champion, 1892: 498.

***Stenomorpha tenuicollis* (Triplehorn, 1967)** USA (AZ NM TX)

*Megasida tenuicollis* Triplehorn, 1967: 40.

***Stenomorpha zacatecensis* (Pallister, 1954)** MEX (ZA)

*Megasida zacatecensis* Pallister, 1954: 32.

**Subgenus Notiasida Casey, 1912**

*Notiasida* Casey, 1912: 76, 124. Type species: *Notiasida abstrusa* Casey, 1912, original designation.

***Stenomorpha abstrusa* (Casey, 1912)** MEX (FD)

*Notiasida abstrusa* Casey, 1912: 125.

***Stenomorpha evertissima* (Casey, 1912)** MEX (CH DU)

*Notiasida evertissima* Casey, 1912: 126.

***Stenomorpha geminata* (Champion, 1892)** MEX (CH DU)

*Asida geminata* Champion, 1892: 492.

***Stenomorpha lata* (Champion, 1884)** MEX (SL)

*Asida lata* Champion, 1884: 60.

***Stenomorpha lugubris* (Wilke, 1922)** MEX (SL)

*Pelecyphorus lugubris* Wilke, 1922: 269.

***Stenomorpha suturalis* (Champion, 1884)** MEX (FD OA VE)

*Asida suturalis* Champion, 1884: 55.

**Subgenus Platasida Casey, 1912**

*Platasida* Casey, 1912: 77, 182. Type species: *Asida embaphionides* Horn, 1894, original designation.

***Stenomorpha embaphionides* (Horn, 1894)** MEX (BS)

*Asida embaphionides* Horn, 1894b: 419.

*Asida flaccida* Horn, 1896: 379. **New synonymy** [ADS].

**Subgenus Pycnomorpha Motschulsky, 1870**

*Pycnomorpha* Motschulsky, 1870: 398. Type species: *Pycnomorpha californica* Motschulsky, 1870, monotypy.

***Stenomorpha californica* (Motschulsky, 1870)** MEX (BC)

*Pycnomorpha californica* Motschulsky, 1870: 399.

***Stenomorpha gabbii* (Horn, 1880)** USA (CA) MEX (BS)

*Asida gibbicollis* Horn, 1870: 288 [junior primary homonym of *Asida gibbicollis* Pérez Arcas, 1865].

*Asida gabbii* Horn, 1880: 152. Replacement name for *Asida gibbicollis* Horn, 1870.

***Stenomorpha tumidicollis* Blaisdell, 1943** MEX (BC)

*Stenomorpha tumidicollis* Blaisdell, 1943: 226.

**Subgenus Stenomorpha Solier, 1836**

*Stenomorpha* Solier, 1836: 487. Type species: *Stenomorpha blapsoides* Solier, 1836, subsequent designation (Desmarest 1860: 150).

*Psilomera* Motschulsky, 1870: 400. Type species: *Pelecyphorus angulatus* LeConte, 1851, monotypy. **New synonymy** [YB].

***Stenomorpha advena* (Casey, 1912)** USA (CO)

*Euschides advena* Casey, 1912: 143.

***Stenomorpha amplicollis* (Casey, 1912)** USA (CA)

*Euschides amplicollis* Casey, 1912: 153.

***Stenomorpha angulata* (LeConte, 1851)** USA (CA)

*Pelecyphorus angulatus* LeConte, 1851: 127.

***Stenomorpha blanda* (Champion, 1884)** MEX (AG GU JA)

*Asida blanda* Champion, 1884: 63.

***Stenomorpha blapsoides alutacea* Wilke, 1922** MEX (ME)

*Stenomorpha blapsoides alutacea* Wilke, 1922: 272.

***Stenomorpha blapsoides blapsoides* Solier, 1836** MEX (CO FD GU JA ME OA PU SI VE)

*Stenomorpha blapsoides* Solier, 1836: 491.

***Stenomorpha brevimargo* (Casey, 1912)** USA (AZ)

*Euschides brevimargo* Casey, 1912: 134.

***Stenomorpha caliginosa* (Casey, 1912)** USA (AZ)

*Euschides caliginosus* Casey, 1912: 137.

***Stenomorpha captiosa* (Horn, 1870)** USA (CA)

*Asida captiosa* Horn, 1870: 287.

***Stenomorpha clarissae* Wilke, 1922** MEX (ME)

*Stenomorpha clarissae* Wilke, 1922: 273.

***Stenomorpha compressa* (Horn, 1870)** USA (CA)

Asida lecontei var. compressa Horn, 1870: 287.

***Stenomorpha congruens congruens* (Casey, 1912)** USA (NM)

*Euschides congruens* Casey, 1912: 164.

***Stenomorpha congruens lubrica* (Casey, 1912)** USA (AZ)

*Euschides congruens lubricus* Casey, 1912: 164.

***Stenomorpha consobrina* (Horn, 1870)** USA (ID OR)

*Asida consobrina* Horn, 1870: 287.

***Stenomorpha consors* (Casey, 1912)** USA (NM)

*Euschides consors* Casey, 1912: 162.

***Stenomorpha consueta* (Casey, 1912)** USA (AZ)

*Euschides consuetus* Casey, 1912: 161.

***Stenomorpha convexa* (LeConte, 1859)** USA (CA KS)

*Euschides convexa* LeConte, 1859a: 14.

***Stenomorpha convexicollis* (LeConte, 1854)** USA (CA CO KS NM TX) MEX (CH DU)

*Euschides convexicollis* LeConte, 1854c: 224.

***Stenomorpha corrugans* (Casey, 1912)** USA (AZ)

*Euschides corrugans* Casey, 1912: 143.

***Stenomorpha costata* Solier, 1836** MEX (ME VE)

*Stenomorpha costata* Solier, 1836: 490.

***Stenomorpha crassa* (Casey, 1912)** USA (CA)

*Euschides crassus* Casey, 1912: 154.

***Stenomorpha cressoni* (Blaisdell, 1933)** USA (CA)

*Euschides cressoni* Blaisdell, 1933b: 191.

***Stenomorpha cribrata* (Casey, 1912)** USA (NM)

*Euschides cribratus* Casey, 1912: 140.

***Stenomorpha crinita* (Casey, 1912)** USA (OR)

*Euschides crinitus* Casey, 1912: 148.

***Stenomorpha deceptor* (Casey, 1912)** USA (CA)

*Euschides deceptor* Casey, 1912: 154.

***Stenomorpha directa* (Casey, 1912)** USA (AZ)

*Euschides directa* Casey, 1912: 141.

***Stenomorpha evanescens* (Casey, 1912)** USA (CA)

*Euschides evanescens* Casey, 1912: 146.

***Stenomorpha facilis* (Casey, 1912)** USA (KS)

*Euschides facilis* Casey, 1912: 165.

***Stenomorpha fastigiosa* (Casey, 1912)** USA (NM)

*Euschides fastigiosus* Casey, 1912: 157.

***Stenomorpha globicollis* (Casey, 1912)** USA (NE)

*Euschides globicollis* Casey, 1912: 158.

***Stenomorpha gracilior* (Casey, 1912)** USA (KS)

*Euschides gracilior* Casey, 1912: 160.

***Stenomorpha gravidipes* (Casey, 1912)** USA (CA)

*Euschides gravidipes* Casey, 1912: 155.

***Stenomorpha huachucae* (Casey, 1912)** USA (AZ)

*Euschides huachucae* Casey, 1912: 162.

***Stenomorpha implicans* (Casey, 1912)** USA (AZ)

*Euschides implicans* Casey, 1912: 136.

***Stenomorpha inhabilis inhabilis* (Casey, 1912)** USA (NM)

*Euschides inhabilis* Casey, 1912: 156.

***Stenomorpha inhabilis retusa* (Casey, 1912)** USA (KS)

*Euschides inhabilis retusus* Casey, 1912: 157.

***Stenomorpha integer* (Casey, 1912)** USA (CA)

*Euschides integer* Casey, 1912: 153.

***Stenomorpha lecontei lecontei* (Horn, 1870)** USA (CA)

*Pelecyphorus costipennis* LeConte, 1859b: 76 [junior primary homonym of *Pelecyphorus costipennis* LeConte, 1858].

*Asida lecontei* Horn, 1870: 286. Replacement name for *Asida costipennis* (LeConte, 1859).

***Stenomorpha lecontei gigantea* (Blaisdell, 1921)** USA (CA)

*Euschides lecontei gigantea* Blaisdell, 1921b: 209.

***Stenomorpha lecontella lecontella* (Blaisdell, 1936)** USA (CA)

*Euschides lecontella* Blaisdell, 1936b: 227.

***Stenomorpha lecontella tempestalis* (Blaisdell, 1936)** USA (CA)

*Euschides lecontella tempestalis* Blaisdell, 1936b: 229.

***Stenomorpha luctata* (Horn, 1870)** USA (CA NV)

*Asida luctata* Horn, 1870: 286.

***Stenomorpha marginata duplicans* (Casey, 1912)** USA [AZ]

*Euschides marginatus duplicans* Casey, 1912: 136.

***Stenomorpha marginata esuriens* (Casey, 1912)** USA (CA)

*Euschides marginatus esuriens* Casey, 1912: 137.

***Stenomorpha marginata marginata* (LeConte, 1851)** USA (AZ CA NM TX) MEX (CH SO)

*Pelecyphorus marginatus* LeConte, 1851: 128.

***Stenomorpha maritima imula* (Casey, 1912)** USA (CA)

*Euschides maritimus imulus* Casey, 1912: 151.

***Stenomorpha maritima maritima* (Casey, 1912)** USA (CA)

*Euschides maritimus* Casey, 1912: 151.

***Stenomorpha mckittricki* (Pierce, 1954)** USA (CA)^14^

*Parasida mckittricki* Pierce, 1954a: 43.

***Stenomorpha montezuma* Wilke, 1922** MEX (DU)

*Stenomorpha montezuma* Wilke, 1922: 272.

***Stenomorpha musiva* Wilke, 1922** MEX (ME)

*Stenomorpha musiva* Wilke, 1922: 273.

***Stenomorpha neutralis* (Casey, 1912)** USA (CA)

*Euschides neutralis* Casey, 1912: 146.

***Stenomorpha oblonga* (Casey, 1924)** USA (NM)

*Euschides oblongus* Casey, 1924: 309.

***Stenomorpha obovata gliscans* (Casey, 1912)** USA (AZ)

*Euschides obovatus gliscans* Casey, 1912: 161.

***Stenomorpha obovata nitidipennis* (Casey, 1912)** USA (AZ)

*Euschides obovatus nitidipennis* Casey, 1912: 160.

***Stenomorpha obovata obovata* (LeConte, 1851)** USA (AZ CA TX) MEX (CH)

*Euschides obovata* LeConte, 1851: 127.

***Stenomorpha oregonensis* (Casey, 1924)** USA (OR)

*Euschides oregonensis* Casey, 1924: 309.

***Stenomorpha orizabae* Wilke, 1922** MEX (VE)

*Stenomorpha orizabae* Wilke, 1922: 273.

***Stenomorpha papagoana* (Casey, 1912)** USA (AZ)

*Euschides papagoanus* Casey, 1912: 163.

***Stenomorpha pollens pollens* (Casey, 1912)** USA (AZ)

*Euschides pollens* Casey, 1912: 134.

***Stenomorpha pollens proxima* (Casey, 1912)** USA (AZ)

*Euschides pollens proximus* Casey, 1912: 134.

***Stenomorpha procurrens* (Casey, 1912)** USA (AZ)

*Euschides procurrens* Casey, 1912: 137.

***Stenomorpha puncticollis* (LeConte, 1866)** USA (OR WA)

*Euschides puncticollis* LeConte, 1866b: 111 [junior secondary homonym of *Asida puncticollis* Solier, 1836].

*Asida robusta* Gebien, 1910a: 135. Replacement name for *Asida puncticollis* (LeConte, 1866)^15^.

***Stenomorpha rimata rimata* (LeConte, 1854)** USA (TX)

*Pelecyphorus rimatus* LeConte, 1854c: 223.

***Stenomorpha rimata subplanata* (Casey, 1912)** USA (AZ)

*Euschides rimatus subplanatus* Casey, 1912: 139.

***Stenomorpha rudis* (Casey, 1912)** USA (AZ)

*Euschides rudis* Casey, 1912: 139.

***Stenomorpha rugata* (Casey, 1912)** USA (AZ)

*Euschides rugatus* Casey, 1912: 138.

***Stenomorpha rustica* (Casey, 1912)** USA (AZ)

*Euschides rusticus* Casey, 1912: 135.

***Stenomorpha satiata* (Casey, 1912)** USA (AZ)

*Euschides satiatus* Casey, 1912: 138.

***Stenomorpha semirufa* (Casey, 1912)** USA (AZ)

*Euschides semirufus* Casey, 1912: 140.

***Stenomorpha severa* (Casey, 1912)** USA (NM)

*Euschides severus* Casey, 1912: 144.

***Stenomorpha socialis* (Casey, 1912)** USA (NM)

*Euschides socialis* Casey, 1912: 162.

***Stenomorpha speculata* (Blaisdell, 1936)** USA (CA)

*Euschides speculatus* Blaisdell, 1936b: 225.

***Stenomorpha sphaericollis* (Champion, 1884)** MEX (AG SL)

*Asida sphaericollis* Champion, 1884: 64.

***Stenomorpha sponsor* (Casey, 1912)** USA (AZ)

*Euschides sponsor* Casey, 1912: 135.

***Stenomorpha spurcans* (Casey, 1912)** USA (CA)

*Euschides spurcans* Casey, 1912: 145.

***Stenomorpha strigosula* (Casey, 1912)** USA (AZ)

*Euschides strigosulus* Casey, 1912: 163.

***Stenomorpha subcruenta* (Casey, 1912)** USA (NM)

*Euschides subcruentus* Casey, 1912: 140.

***Stenomorpha subcylindrica* (Horn, 1870)** USA (AZ)

Asida marginata var. subcylindrica Horn, 1870: 288.

***Stenomorpha subelegans* (Casey, 1912)** USA (CA)

*Euschides subelegans* Casey, 1912: 152.

***Stenomorpha tetrica* (Casey, 1912)** USA (UT)

*Euschides tetricus* Casey, 1912: 149.

***Stenomorpha tularensis* (Casey, 1912)** USA (CA)

*Euschides tularensis* Casey, 1912: 152.

***Stenomorpha uhdei* Wilke, 1922** MEX (ME)

*Stenomorpha uhdei* Wilke, 1922: 273.

***Stenomorpha umbrosa* (Champion, 1884)** MEX (GU)

*Asida umbrosa* Champion, 1884: 62.

***Stenomorpha vigens* (Casey, 1912)** USA (AZ)

*Euschides vigens* Casey, 1912: 159.

**Subgenus Stethasida Casey, 1912**

*Stethasida* Casey, 1912: 78, 203. Type species: *Pelecyphorus muricatulus* LeConte, 1851, original designation.

***Stenomorpha flohri* (Champion, 1892)** MEX (JA)

*Asida flohri* Champion, 1892: 496.

***Stenomorpha muricatula* (LeConte, 1851)** USA (CA)

*Pelecyphorus muricatulus* LeConte, 1851: 128.

*Asida angustula* Casey, 1890b: 370. **New synonymy** [ADS].

*Stethasida stricta* Casey, 1912: 210. **New synonymy** [ADS].

*Stethasida muricatula languida* Casey, 1912: 211. **New synonymy** [ADS].

*Stethasida pertinax* Casey, 1912: 211. **New synonymy** [ADS].

*Stethasida socors* Casey, 1912: 212. **New synonymy** [ADS].

*Stethasida angustula inepta* Casey, 1912: 213. **New synonymy** [ADS].

*Stethasida tenax* Casey, 1912: 213. **New synonymy** [ADS].

*Stethasida vegrandis* Casey, 1912: 214. **New synonymy** [ADS].

***Stenomorpha obsoleta* (LeConte, 1851)** USA (CA)

*Pelecyphorus obsoletus* LeConte, 1851: 128.

*Stethasida obsoleta expansa* Casey, 1912: 205. **New synonymy** [ADS].

*Stethasida obsoleta opacella* Casey, 1912: 205. **New synonymy** [ADS].

*Stethasida brevipes* Casey, 1912: 206. **New synonymy** [ADS].

*Stethasida torpida* Casey, 1912: 206. **New synonymy** [ADS].

*Stethasida convergens* Casey, 1912: 207. **New synonymy** [ADS].

*Stethasida discreta* Casey, 1912: 207. **New synonymy** [ADS].

*Stethasida longula* Casey, 1912: 207. **New synonymy** [ADS].

*Stethasida adumbrata* Casey, 1912: 208. **New synonymy** [ADS].

*Stethasida occulta* Casey, 1912: 208. **New synonymy** [ADS].

*Stethasida tarsalis* Casey, 1912: 208. **New synonymy** [ADS].

*Stethasida unica* Casey, 1912: 209. **New synonymy** [ADS].

*Pelecyphorus laevigatus* Papp, 1961c: 159. **New synonymy** [ADS].

**Subgenus Trichiasida Casey, 1912**

*Trichiasida* Casey, 1912: 77, 172. Type species: *Pelecyphorus hirsutus* LeConte, 1851, original designation.

***Stenomorpha acerba* (Horn, 1878)** USA (AZ NV UT)

*Asida acerba* Horn, 1878a: 56.

***Stenomorpha difficilis* (Champion, 1884)** MEX (HI ME SL)

*Asida difficilis* Champion, 1884: 61.

*Trichiasida eremica* Wilke, 1922: 274. **New synonymy** [ADS].

***Stenomorpha hirsuta* (LeConte, 1851)** USA (CA)

*Pelecyphorus hirsutus* LeConte, 1851: 127.

*Trichiasida lineatopilosa* Casey, 1912: 175. **New synonymy** [ADS].

***Stenomorpha hispidula* (LeConte, 1851)** USA (AZ CA)

*Pelecyphorus hispidulus* LeConte, 1851: 127.

*Trichiasida tenella* Casey, 1912: 177. **New synonymy** [ADS].

***Stenomorpha horrida* (Champion, 1892)** USA (TX) MEX (TA)

*Asida horrida* Champion, 1892: 500.

***Stenomorpha idahoensis* (Boddy, 1957)** USA (ID)

*Trichiasida idahoensis* Boddy, 1957: 187.

***Stenomorpha ignava* (Casey, 1912)** USA (AZ)

*Trichiasida ignava* Casey, 1912: 180.

***Stenomorpha impetrata* (Horn, 1894)** USA (CA)

*Asida impetrata* Horn, 1894b: 418.

***Stenomorpha impotens* (Casey, 1912)** USA (AZ)

*Trichiasida impotens* Casey, 1912: 180.

***Stenomorpha lutulenta* (Doyen, 1990)** MEX (JA)

*Trichiasida lutulenta* Doyen, 1990: 225.

***Stenomorpha palmeri* (Champion, 1884)** MEX (SL)

*Asida palmeri* Champion, 1884: 59.

***Stenomorpha pubescens* (Champion, 1884)** MEX

*Asida pubescens* Champion, 1884: 61.

***Stenomorpha subpilosa* Solier, 1836** MEX (PU)

*Stenomorpha subpilosa* Solier, 1836: 490.

***Stenomorpha thoracica* (Champion, 1884)** MEX

*Asida thoracica* Champion, 1884: 62.

***Stenomorpha unicostata* (Champion, 1892)** MEX (GE)

*Asida unicostata* Champion, 1892: 501.

***Stenomorpha villosa* (Champion, 1884)** MEX (PU)

*Asida villosa* Champion, 1884: 60.

*Trichiasida duplex* Casey, 1912: 178^16^. **New synonymy** [ADS].

[incertae sedis]

***Stenomorpha catalinae* (Blaisdell, 1923)** MEX (BS)

*Asida catalinae* Blaisdell, 1923: 256.

***Stenomorpha furcata* (Champion, 1892)** USA (TX) MEX (CO DU)

*Asida furcata* Champion, 1892: 499.

***Stenomorpha granicollis* (Blaisdell, 1923)** MEX (SO)

*Asida granicollis* Blaisdell, 1923: 256.

***Stenomorpha roosevelti* Smith, Miller and Wheeler, 2011** MEX (CO)

*Stenomorpha roosevelti* Smith, Miller and Wheeler, 2011: 30.

***Stenomorpha spinimana* (Champion, 1892)** MEX (DU) **New combination** [ADS].

*Asida spinimanus* Champion, 1892: 494.

***Stenomorpha subvittata* (Horn, 1894)** MEX (BS)

*Asida subvittata* Horn, 1894b: 416.

***Stenomorpha tenebrosa* (Champion, 1892)** MEX (CO) **New combination** [ADS].

*Asida tenebrosa* Champion, 1892: 495.

***Stenomorpha wickhami* (Horn, 1894)** USA (AZ CA)

*Asida wickhami* Horn, 1894b: 420.

*Asidina liberta* Casey, 1912: 171. Synonymy: Triplehorn and Brown (1971: 84).

**Tribe Branchini LeConte, 1862**

Branchini LeConte, 1862a: 222. Type genus: *Branchus* LeConte, 1862

**Genus *Anectus* Horn, 1866** [M]

*Anectus* Horn, 1866: 399. Type species: *Anectus vestitus* Horn, 1866, monotypy.

***Anectus vestitus* Horn, 1866** HON

*Anectus vestitus* Horn, 1866: 399.

**Genus *Branchus* LeConte, 1862** ^17^ [M]

*Branchus* LeConte, 1862a: 222. Type species: *Branchus floridanus* LeConte, 1862, monotypy.

***Branchus floridanus* LeConte, 1862** USA (FL)

*Branchus floridanus* LeConte, 1862a: 223.

***Branchus geraceorum* Steiner, 2006** BAH

*Branchus geraceorum* Steiner, 2006: 10.

***Branchus jamaicensis* Marcuzzi, 1977** JAM

*Branchus jamaicensis* Marcuzzi, 1977: 10.

***Branchus obscurus* Horn, 1866** MEX (GE) GUA NIC

*Branchus obscurus* Horn, 1866: 398.

***Branchus opatroides* Champion, 1892** MEX (JA VE)

*Branchus opatroides* Champion, 1892: 507.

***Branchus saxatilis* Steiner, 2005** BAH

*Branchus saxatilis* Steiner, 2005: 443.

***Branchus whiteheadi* Steiner, 1991** USA (TX)

*Branchus whiteheadi* Steiner, 1991: 426.

***Branchus woodii* LeConte, 1866** BAH CUB

*Branchus woodii* LeConte, 1866b: 111.

**Genus *Oxinthas* Champion, 1884** [M]

*Oxinthas* Champion, 1884: 72. Type species: *Oxinthas praocioides* Champion, 1884, monotypy.

***Oxinthas nicaraguensis* Merkl, 1992** NIC

*Oxinthas nicaraguensis* Merkl, 1992: 89.

***Oxinthas praocioides* Champion, 1884** MEX (OA)

*Oxinthas praocioides* Champion, 1884: 72.

**Tribe Cnemeplatiini Jacquelin du Val, 1861**

Cnéméplatiites Jacquelin du Val, 1861: 286. Type genus: *Cnemeplatia* Costa, 1847.

**Subtribe Cnemeplatiina Jacquelin du Val, 1861**

Cnéméplatiites Jacquelin du Val, 1861: 286. Type genus: *Cnemeplatia* Costa, 1847.

**Genus *Alaudes* Horn, 1870** [M]

*Alaudes* Horn, 1870: 361. Type species: *Alaudes singularis* Horn, 1870, monotypy.

***Alaudes alternatus* Fall, 1928** USA (CA)

*Alaudes alternata* Fall, 1928: 148.

***Alaudes setigerus* Blaisdell, 1919** USA (CA)

*Alaudes setigera* Blaisdell, 1919a: 310.

***Alaudes singularis* Horn, 1870** USA (CA ID NV OR)

*Alaudes singularis* Horn, 1870: 362.

*Alaudes squamosa* Blaisdell, 1919a: 309. **New synonymy** [RLA].

*Alaudes testacea* Blaisdell, 1919a: 311. **New synonymy** [RLA].

*Alaudes fallax* Fall, 1928: 150. **New synonymy** [RLA].

**Genus *Lepidocnemeplatia* Bousquet and Bouchard, new genus** [F]

*Lepidocnemeplatia* Bousquet and Bouchard, new genus. Type species: *Cnemeplatia sericea* Horn, 1870.

***Lepidocnemeplatia laticollis* (Champion, 1885)** MEX (ME) NIC CRI PAN / SA

*Cnemeplatia laticollis* Champion, 1885: 136.

***Lepidocnemeplatia sericea* (Horn, 1870)** USA (AZ CA NV OR TX WA) MEX (BS CH DU MO NL PU SO VE) NIC

*Cnemeplatia sericea* Horn, 1870: 360.

**Tribe Cnemodinini Gebien, 1910**

Cnemodini Horn, 1870: 266. Type genus: *Cnemodus* Horn, 1870 (= *Cnemodinus* Cockerell, 1906).

Cnemodininae Gebien, 1910a: 4. Type genus: *Cnemodinus* Cockerell, 1906.

**Genus *Cnemodinus* Cockerell, 1906** [M]

*Cnemodus* Horn, 1870: 266 [junior homonym of *Cnemodus* Herrich-Schaeffer, 1850]. Type species: *Cnemodus testaceus* Horn, 1870, monotypy.

*Cnemodinus* Cockerell, 1906: 242. Replacement name for *Cnemodus* Horn, 1870.

***Cnemodinus angustus* (Casey, 1907)** USA (AZ)

*Cnemodus angustus* Casey, 1907: 284.

***Cnemodinus subhyalinus* (Casey, 1907)** USA (UT)

*Cnemodus subhyalinus* Casey, 1907: 285.

***Cnemodinus testaceus* (Horn, 1870)** USA (AZ CA)

*Cnemodus testaceus* Horn, 1870: 266.

**Tribe Coniontini G.R. Waterhouse, 1858**

Coniontidae G.R. Waterhouse, 1858: 59. Type genus: *Coniontis* Eschscholtz, 1829.

Coelini Casey, 1907: 500. Type genus: *Coelus* Eschscholtz, 1829.

Eusatti Doyen, 1984b: 11. Type genus: *Eusattus* LeConte, 1851.

**Genus *Coelus* Eschscholtz, 1829** [M]

*Coelus* Eschscholtz, 1829: 5. Type species: *Coelus ciliatus* Eschscholtz, 1829, monotypy.

*Coelomorpha* Casey, 1890a: 182. Type species: *Coelomorpha maritima* Casey, 1890, monotypy. Synonymy: Doyen (1972: 371).

*Pseudocoelus* Casey, 1908: 152. Type species: *Coelus pacificus* Fall, 1897, subsequent designation (Doyen 1976: 608). Synonymy: Blaisdell (1919b: 322).

**Figure 12.**
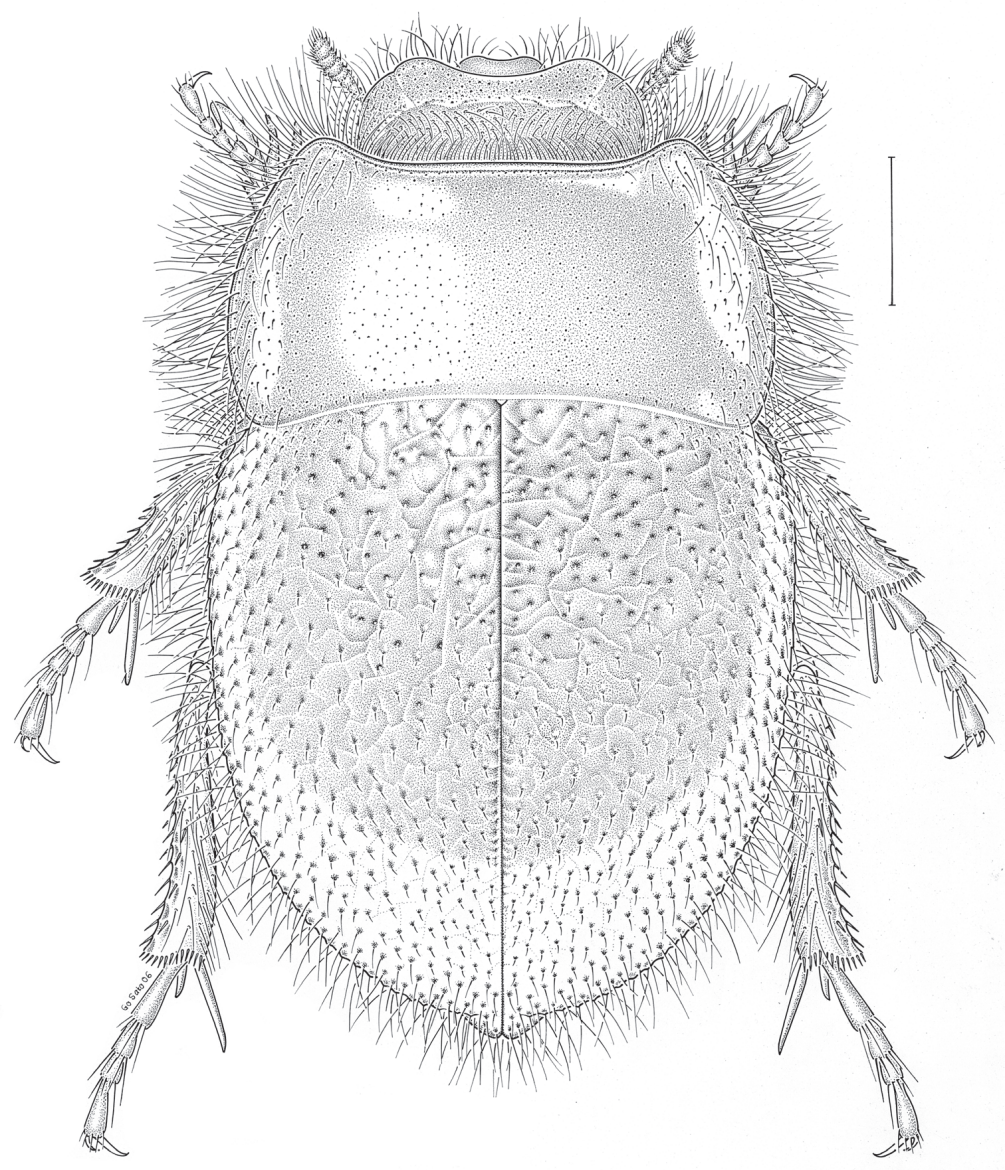
*Coelus ciliatus* Eschscholtz, 1829. Scale bar = 1 mm.

***Coelus ciliatus* Eschscholtz, 1829** [Fig. 12] CAN (BC) USA (CA OR WA) MEX (BC)

*Coelus ciliatus* Eschscholtz, 1829: 5.

*Coelus arenarius* Casey, 1890a: 179. Synonymy: Doyen (1976: 614).

*Coelus latus* Casey, 1895: 612. Synonymy (with *C. arenarius* Casey): Fall (1901: 166).

*Coelus curtulus* Casey, 1895: 612. Synonymy: Fall (1901: 166).

*Coelus ciliatus longulus* Casey, 1908: 154. Synonymy: Blaisdell (1919b: 333).

*Coelus debilis* Casey, 1908: 155. Synonymy: Doyen (1976: 614).

*Coelus sternalis* Casey, 1908: 156. Synonymy: Doyen (1976: 614).

*Coelus obscurus* Casey, 1908: 156. Synonymy (with *C. arenarius* Casey): Blaisdell (1919b: 334).

*Coelus scolopax* Casey, 1908: 157. Synonymy (with *C. arenarius* Casey): Blaisdell (1919b: 334).

*Coelus amplicollis* Casey, 1908: 157. Synonymy (with *C. latus* Casey): Blaisdell (1919b: 334).

Coelus ciliatus var. sparsus Blaisdell, 1919b: 325. Synonymy (with *C. ciliatus debilis* Casey): Gebien (1938: 409).

***Coelus globosus* LeConte, 1851** USA (CA) MEX (BC)

*Coelus globosus* LeConte, 1851: 133.

*Coelus grossus* Casey, 1890a: 178. Synonymy: Doyen (1976: 617).

*Coelus solidus* Casey, 1908: 153. Synonymy (with *C. globosus grossus* Casey): Blaisdell (1919b: 333).

*Coelus saginatus* Casey, 1908: 154. Synonymy: Doyen (1976: 617).

***Coelus gracilis* Blaisdell, 1939** USA (CA)

*Coelus gracilis* Blaisdell, 1939a: 16.

***Coelus maritimus* (Casey, 1889)** MEX (BC)

*Coelomorpha maritima* Casey, 1890a: 183.

*Coelomorpha pallens* Casey, 1908: 160. Synonymy: Doyen (1976: 620).

***Coelus pacificus* Fall, 1897** USA (CA)

*Coelus pacificus* Fall, 1897: 241.

*Coelus remotus* Fall, 1897: 241. Synonymy: Doyen (1976: 618).

**Genus *Coniontis* Eschscholtz, 1829** [F]

*Coniontis* Eschscholtz, 1829: 7. Type species: *Coniontis viatica* Eschscholtz, 1829, subsequent designation (Casey 1908: 57).

*Coelotaxis* Horn, 1876a: 200. Type species: *Coelotaxis punctulata* Horn, 1876, subsequent designation (Gebien 1938: 289). Synonymy: Doyen (1972: 373).

*Coniontellus* Casey, 1890b: 388. Type species: *Coniontis obesa* LeConte, 1851, subsequent designation (Casey 1908: 57). Synonymy: Doyen (1972: 373).

*Coniontides* Casey, 1908: 57, 78. Type species: *Coniontis lata* LeConte, 1866, original designation. Synonymy: Doyen (1972: 373).

*Crypticomorpha* Casey, 1908: 81, 140. Type species: *Coniontis tenuis* Casey, 1908, monotypy. Synonymy: Aalbu et al. (2002: 487).

*Brachyontis* Casey, 1908: 82, 141. Type species: *Coniontis globulina* Casey, 1895, monotypy. Synonymy: Aalbu et al. (2002: 487).

***Coniontis abdominalis* LeConte, 1859** USA (CA)

*Coniontis abdominalis* LeConte, 1859b: 77.

*Coniontis strenua* Casey, 1908: 84. Synonymy: Doyen (1977: 2).

*Coniontis tristis* Casey, 1908: 84. Synonymy: Doyen (1977: 2).

*Coniontis gravis* Casey, 1908: 85. Synonymy: Doyen (1977: 2).

*Coniontis rugosa* Casey, 1908: 85. Synonymy: Doyen (1977: 2).

*Coniontis tenebrosa* Casey, 1908: 86. Synonymy: Doyen (1977: 2).

*Coniontis abdominalis caseyi* Pierce, 1954c: 145. Synonymy: Doyen and Miller (1980: 3).

*Coniontis abdominalis labreae* Pierce, 1954c: 146. Synonymy: Doyen and Miller (1980: 3).

*Coniontis abdominalis fragmans* Pierce, 1954c: 148. Synonymy: Doyen and Miller (1980: 3).

*Coniontis tristis alpha* Pierce, 1954c: 148. Synonymy: Doyen and Miller (1980: 3).

*Coniontis tristis asphalti* Pierce, 1954c: 149. Synonymy: Doyen and Miller (1980: 3).

*Coniontis tristis latigula* Pierce, 1954c: 149. Synonymy: Doyen and Miller (1980: 3).

*Coniontis blissi* Pierce, 1954c: 149. Synonymy: Doyen and Miller (1980: 3).

*Coniontis pectoralis paraelliptica* Pierce, 1954c: 153. Synonymy: Doyen and Miller (1980: 3).

*Coniontis pectoralis interrupta* Pierce, 1954c: 154. Synonymy: Doyen and Miller (1980: 3).

***Coniontis affinis* LeConte, 1851** USA (CA OR)

*Coniontis affinis* LeConte, 1851: 130.

*Coniontis expansa* Casey, 1908: 120. Synonymy: Doyen (1977: 2).

*Coniontis franciscana* Casey, 1908: 120. Synonymy: Doyen (1977: 2).

*Coniontis truncata* Casey, 1908: 120. Synonymy: Doyen (1977: 2).

*Coniontis suturalis* Casey, 1908: 121. Synonymy: Doyen (1977: 2).

*Coniontis audax* Casey, 1908: 121. Synonymy: Doyen (1977: 2).

*Coniontis symmetrica* Casey, 1908: 122. Synonymy: Doyen (1977: 2).

*Coniontis convergens* Casey, 1908: 122. Synonymy: Doyen (1977: 2).

*Coniontis anxia* Casey, 1908: 123. Synonymy: Doyen (1977: 2).

*Coniontis affinis patruelis* Casey, 1908: 123. Synonymy: Doyen (1977: 2).

*Coniontis oregona* Casey, 1908: 124. Synonymy: Doyen (1977: 2).

*Coniontis pagana* Casey, 1908: 130. Synonymy: Doyen (1977: 2).

***Coniontis atronitens* Casey, 1908** USA (CA)

*Coniontis atronitens* Casey, 1908: 110.

***Coniontis blaisdelli* Casey, 1908** USA (CA)

*Coniontis blaisdelli* Casey, 1908: 97.

***Coniontis callida* Casey, 1908** USA (CA)

*Coniontis callida* Casey, 1908: 128.

*Coniontis shastanica* Casey, 1908: 128. Synonymy: Doyen (1977: 2).

*Coniontis conferta* Casey, 1908: 129. Synonymy: Doyen (1977: 2).

*Coniontis agrestis* Casey, 1908: 131. Synonymy: Doyen (1977: 2).

*Coniontis congesta* Casey, 1908: 131. Synonymy: Doyen (1977: 2).

***Coniontis costulata* Casey, 1908** USA (CA)

*Coniontis costulata* Casey, 1908: 89.

***Coniontis elliptica* Casey, 1884** USA (CA)

*Coniontis elliptica* Casey, 1884: 46.

*Coniontis laevigata* Casey, 1908: 88. Synonymy: Doyen (1977: 2).

*Coniontis elliptica catalinae* Casey, 1908: 88. Synonymy: Doyen (1977: 2).

*Coniontis cuneata* Casey, 1908: 111. Synonymy: Gebien (1938: 286).

***Coniontis elongata* Casey, 1890** USA (CA)

*Coniontis elongata* Casey, 1890b: 380.

*Coniontis rotundicollis* Casey, 1908: 97. Synonymy: Doyen (1977: 3).

*Coniontis innocua* Casey, 1908: 99. Synonymy: Doyen (1977: 3).

*Coniontis elongata limatula* Casey, 1908: 99. Synonymy: Doyen (1977: 3).

*Coniontis cylindrica* Casey, 1908: 100. Synonymy: Doyen (1977: 3).

*Coniontis obsidiana* Casey, 1908: 100. Synonymy: Blaisdell (1935b: 120).

*Coniontis longicollis* Casey, 1908: 101. Synonymy: Doyen (1977: 3).

***Coniontis eschscholtzii* Mannerheim, 1840** USA (CA)

*Coniontis eschscholtzii* Mannerheim, 1840: 138.

***Coniontis extricata* Casey, 1908** USA (CA OR)

*Coniontis extricata* Casey, 1908: 124.

*Coniontis marginata* Casey, 1908: 125. Synonymy: Doyen (1977: 3).

*Coniontis minuta* Casey, 1908: 126. Synonymy: Doyen (1977: 3).

*Coniontis parva* Casey, 1908: 126. Synonymy: Doyen (1977: 3).

*Coniontis perpolita* Casey, 1908: 127. Synonymy: Doyen (1977: 3).

*Coniontis pudica* Casey, 1908: 127. Synonymy: Doyen (1977: 3).

*Coniontis nemoralis slevini* Blaisdell, 1924a: 86. Synonymy: Doyen (1977: 3).

***Coniontis farallonica* Casey, 1895** USA (CA)

*Coniontis farallonica* Casey, 1895: 610.

***Coniontis genitiva* Casey, 1890** USA (CA)

*Coniontis genitiva* Casey, 1890b: 385.

*Coniontis verna* Casey, 1908: 94. Synonymy: Doyen (1977: 3).

*Coniontis opacicollis* Casey, 1908: 101. Synonymy: Doyen (1977: 3).

***Coniontis globulina* Casey, 1895** USA (CA)

*Coniontis globulina* Casey, 1895: 610.

***Coniontis histrio* Casey, 1908** USA (AZ)

*Coniontis histrio* Casey, 1908: 91.

***Coniontis hoppingi* Blaisdell, 1918** USA (CA)

*Coniontis hoppingi* Blaisdell, 1918a: 7.

***Coniontis inaequalis* Casey, 1890** USA (CA)

*Coniontis inaequalis* Casey, 1890b: 375.

***Coniontis inornata* Casey, 1908** USA (CA)

*Coniontis inornata* Casey, 1908: 130.

***Coniontis integer* Casey, 1908** USA (CA)

*Coniontis integer* Casey, 1908: 87.

***Coniontis keiferi* (Blaisdell, 1943)** MEX (BC)

*Coniontides keiferi* Blaisdell, 1943: 189.

***Coniontis lamentabilis* Blaisdell, 1924** USA (CA)

*Coniontis lamentabilis* Blaisdell, 1924a: 85.

***Coniontis lanei* Boddy, 1957** USA (ORWA)

*Coniontis lanei* Boddy, 1957: 191.

***Coniontis lariversi* Blaisdell, 1941** USA (NV)

*Coniontis lariversi* Blaisdell, 1941c: 131.

***Coniontis lassenica* Casey, 1908** USA (CA NV)

*Coniontis lassenica* Casey, 1908: 95.

*Coniontis nevadensis* Casey, 1908: 95. Synonymy: Doyen (1977: 3).

*Coniontis nevadensis carsonica* Casey, 1908: 95. Synonymy: Doyen (1977: 3).

***Coniontis lata* LeConte, 1866** USA (CA)

*Coniontis lata* LeConte, 1866b: 113.

Coniontis lata var. insularis Casey, 1890b: 377. Synonymy: Doyen (1977: 3).

*Coniontides finitimus* Casey, 1908: 79. Synonymy: Doyen (1977: 3).

*Coniontides clementinus* Casey, 1908: 80. Synonymy: Blaisdell (1921b: 212).

***Coniontis malkini* (Boddy, 1957)** USA (CA)

*Coniontellus malkini* Boddy, 1957: 188.

***Coniontis microsticta* Casey, 1908** USA (CA)

*Coniontis microsticta* Casey, 1908: 107.

*Coniontis inconspicua* Casey, 1908: 108. Synonymy: Blaisdell (1924a: 84).

***Coniontis muscula* Blaisdell, 1918** USA (CA)

Coniontis globulina var. muscula Blaisdell, 1918a: 9.

***Coniontis nemoralis borealis* Boddy, 1957** USA (CA OR)

*Coniontis nemoralis borealis* Boddy, 1957: 192.

***Coniontis nemoralis nemoralis* Eschscholtz, 1829** USA (CA)

*Coniontis nemoralis* Eschscholtz, 1829: 8.

***Coniontis obesa* LeConte, 1851** USA (CA CO ID MT NV OR WY)

*Coniontis obesus* LeConte, 1851: 131.

*Coniontellus inflatus* Casey, 1890b: 389. Synonymy: Doyen (1977: 3).

*Coniontellus subglaber* Casey, 1890b: 389. Synonymy: Doyen (1977: 3).

*Coniontellus hystrix* Casey, 1908: 142. Synonymy (with *C. inflatus* Casey): La Rivers (1947b: 214).

*Coniontellus longipennis* Casey, 1908: 143. Synonymy (with *C. inflatus* Casey): La Rivers (1947b: 214).

*Coniontellus ampliatus* Casey, 1908: 144. Synonymy (with *C. inflatus* Casey): La Rivers (1947b: 214).

*Coniontellus argutus* Casey, 1908: 145. Synonymy: Doyen (1977: 3).

*Coniontellus micans* Casey, 1908: 145. Synonymy: Doyen (1977: 3).

***Coniontis oblonga* Casey, 1908** USA (CA)

*Coniontis oblonga* Casey, 1908: 92.

***Coniontis opaca* Horn, 1870** USA (CA)

*Coniontis opaca* Horn, 1870: 296.

*Coniontis ancilla* Casey, 1908: 91. Synonymy: Doyen (1977: 3).

*Coniontis degener* Casey, 1908: 93. Synonymy: Doyen (1977: 3).

**Figure 13.**
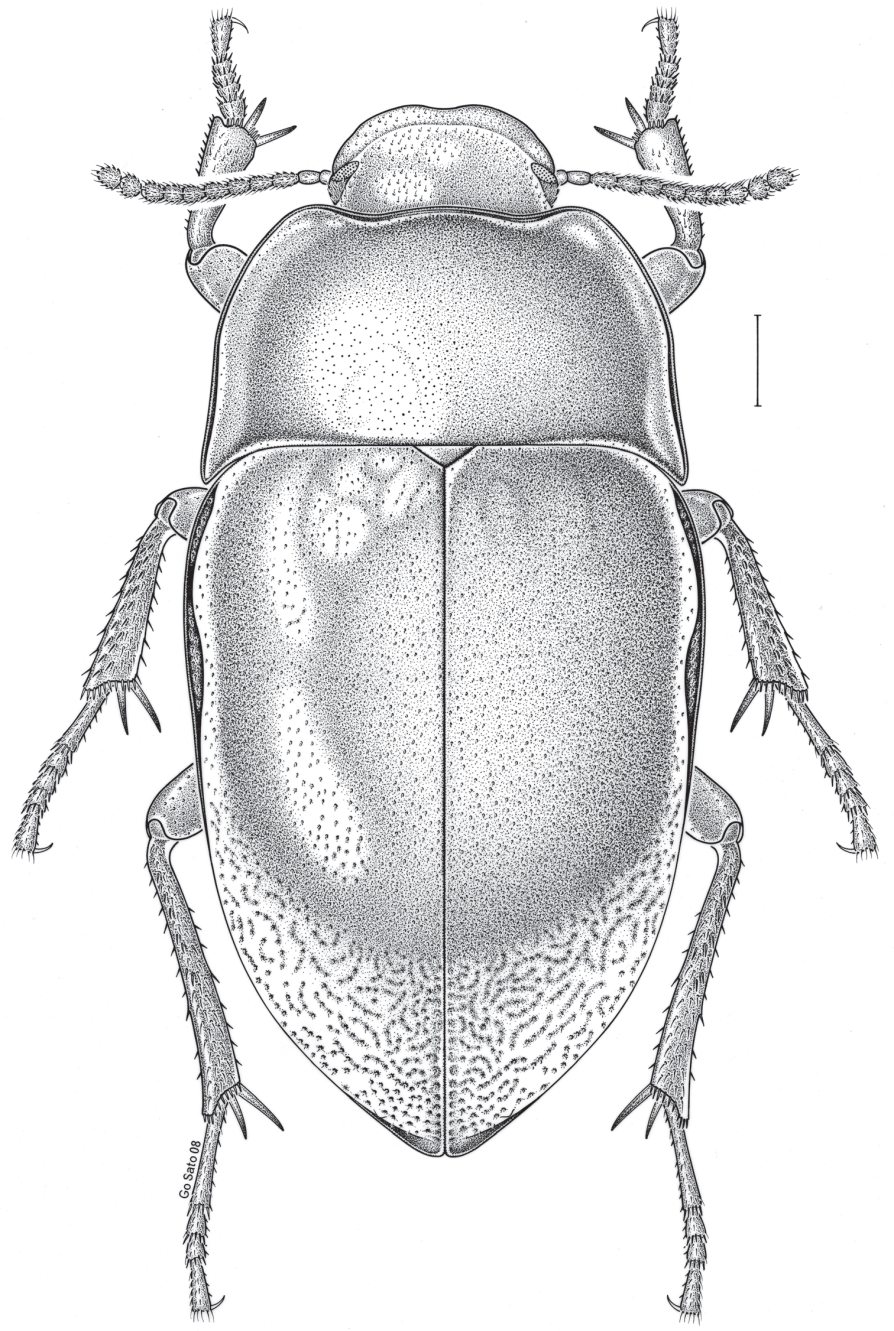
*Coniontis ovalis* LeConte, 1851. Scale bar = 1 mm.

***Coniontis ovalis* LeConte, 1851** [Fig. 13] CAN (BC) USA (AK CA CO ID MT NV OR UT WA)

*Coniontis ovalis* LeConte, 1851: 131.

*Coniontis alutacea* Casey, 1890b: 383. Synonymy: Doyen (1977: 4).

*Coniontis breviuscula* Casey, 1908: 133. Synonymy: Boddy (1957: 190).

*Coniontis sculptipennis* Casey, 1908: 134. Synonymy: Boddy (1957: 190).

*Coniontis regularis* Casey, 1908: 134. Synonymy: Doyen (1977: 4).

*Coniontis punctata* Casey, 1908: 135. Synonymy (with *C. regularis* Casey): Boddy (1965: 141).

*Coniontis parilis* Casey, 1908: 135. Synonymy: Boddy (1957: 190).

*Coniontis vancouveri* Casey, 1908: 136. Synonymy: Boddy (1957: 190).

*Coniontis uteana* Casey, 1908: 136. Synonymy: Doyen (1977: 4).

*Coniontis inepta* Casey, 1908: 137. Synonymy: Doyen (1977: 4).

*Coniontis oblita* Casey, 1908: 137. Synonymy: Doyen (1977: 4).

*Coniontis arida* Casey, 1908: 138. Synonymy: Doyen (1977: 4).

*Coniontis weidti* Casey, 1908: 138. Synonymy: Doyen (1977: 4).

*Coniontis acerba* Casey, 1908: 139. Synonymy: Doyen (1977: 4).

*Coniontis anita* Casey, 1908: 139. Synonymy: Doyen (1977: 4).

*Coniontis corvina* Casey, 1908: 140. Synonymy: Doyen (1977: 4).

*Coniontis ovalis okanagani* Boddy, 1957: 190. Synonymy: Doyen (1977: 4).

***Coniontis pallidicornis* Casey, 1890** USA (CA)

*Coniontis pallidicornis* Casey, 1890b: 385.

*Coniontis obsolescens* Casey, 1908: 92. Synonymy: Doyen (1977: 4).

***Coniontis parallela* Casey, 1890** USA (CA)

*Coniontis parallela* Casey, 1890b: 386.

***Coniontis parviceps* Casey, 1890** USA (CA) MEX (BC)

*Coniontis parviceps* Casey, 1890b: 387.

*Coniontis filiola* Casey, 1908: 115. Synonymy: Doyen (1977: 4).

***Coniontis pectoralis* Casey, 1908** USA (CA)

*Coniontis pectoralis* Casey, 1908: 86.

*Coniontis levettei* Casey, 1908: 87. Synonymy: Doyen (1977: 4).

*Coniontis picescens* Casey, 1908: 87. Synonymy: Doyen (1977: 4).

***Coniontis perspicua* Casey, 1908** USA (CA)

*Coniontis perspicua* Casey, 1908: 114.

***Coniontis proba* Casey, 1908** USA (ID OR)

*Coniontis proba* Casey, 1908: 105.

***Coniontis puncticollis* LeConte, 1851** USA (CA)

*Coniontis puncticollis* LeConte, 1851: 131.

*Coniontis exigua* Casey, 1908: 106. Synonymy: Doyen (1977: 4).

*Coniontis paupercula* Casey, 1908: 106. Synonymy: Doyen (1977: 4).

*Coniontis inflexula* Casey, 1908: 107. Synonymy (with *C. exigua* Casey): Casey (1911: 253).

*Coniontis picipes* Casey, 1908: 107. Synonymy: Doyen (1977: 4).

***Coniontis punctipes* Casey, 1890** USA (CA)

*Coniontis punctipes* Casey, 1890b: 380.

***Coniontis punctulata* (Horn, 1876)** USA (CA) MEX

*Coelotaxis punctulata* Horn, 1876a: 201.

*Coelotaxis muricata* Horn, 1876a: 201. Synonymy: Doyen (1977: 4).

*Coelotaxis angustula* Casey, 1890a: 177. Synonymy: Doyen (1977: 4).

*Coelotaxis densa* Casey, 1908: 149. Synonymy: Doyen (1977: 4).

*Coelotaxis frontalis* Casey, 1908: 149. Synonymy: Doyen (1977: 4).

***Coniontis rainieri* Boddy, 1957** USA (WA)

*Coniontis rainieri* Boddy, 1957: 191.

***Coniontis remnans* Pierce, 1954** USA (CA)^18^

*Coniontis remnans* Pierce, 1954c: 155.

***Coniontis robusta* Horn, 1870** USA (CA)

*Coniontis robusta* Horn, 1870: 296.

*Coniontis luctuosa* Casey, 1908: 89. Synonymy: Doyen (1977: 5).

***Coniontis sanfordii* Blaisdell, 1895** USA (CA)

*Coniontis sanfordii* Blaisdell, 1895: 235.

***Coniontis santarosae* Blaisdell, 1921** USA (CA)

*Coniontis santarosae* Blaisdell, 1921b: 209.

***Coniontis setosa* Casey, 1890** USA (ID NV OR UT WA)

*Coniontis setosus* Casey, 1890b: 387.

*Coniontis obtusa* Casey, 1908: 116. Synonymy: Doyen (1977: 5).

*Coniontis wickhami* Casey, 1908: 117. Synonymy: Doyen (1977: 5).

*Coniontis lanuginosa* Casey, 1908: 117. Synonymy: Doyen (1977: 5).

*Coniontis pubifera* Casey, 1908: 118. Synonymy: Boddy (1965: 140).

***Coniontis sparsa* Casey, 1908** USA (CA)

*Coniontis sparsa* Casey, 1908: 110.

***Coniontis subpubescens* LeConte, 1851** USA (CA OR)

*Coniontis subpubescens* LeConte, 1851: 131.

*Coniontis montana* Casey, 1890b: 384. Synonymy: Doyen (1977: 5).

*Coniontis canonica* Casey, 1908: 114. Synonymy: Blaisdell (1918a: 13).

***Coniontis tenuis* Casey, 1908** USA (CA)

*Coniontis tenuis* Casey, 1908: 141.

***Coniontis thoracica* Casey, 1908** USA (CA)

*Coniontis thoracica* Casey, 1908: 104.

***Coniontis timida* Casey, 1908** USA (CA)

*Coniontis timida* Casey, 1908: 102.

*Coniontis conicicollis* Casey, 1908: 102. Synonymy: Doyen (1977: 5).

*Coniontis lucidula* Casey, 1908: 103. Synonymy: Doyen (1977: 5).

*Coniontis protensa* Casey, 1908: 104. Synonymy: Doyen (1977: 5).

***Coniontis vandykei* Blaisdell, 1921** USA (CA)

*Coniontides vandykei* Blaisdell, 1921b: 212.

***Coniontis ventura* Blaisdell, 1924** USA (CA)

*Coniontis globulina ventura* Blaisdell, 1924a: 83.

***Coniontis viatica* Eschscholtz, 1829** USA (CA)

*Coniontis viatica* Eschscholtz, 1829: 7.

***Coniontis wadei* Casey, 1924** USA (WA)

*Coniontis wadei* Casey, 1924: 313.

**Genus *Conisattus* Casey, 1895** [M]

*Conisattus* Casey, 1895: 614. Type species: *Conisattus rectus* Casey, 1895, monotypy.

***Conisattus rectus* Casey, 1895** USA (OR WA)

*Conisattus rectus* Casey, 1895: 614.

*Conisattus nelsoni* Boddy, 1957: 188. Synonymy: Doyen (1984b: 26).

**Genus *Eusattus* LeConte, 1851** [M]

*Eusattus* LeConte, 1851: 131. Type species: *Eusattus difficilis* LeConte, 1851, subsequent designation (Casey 1908: 56).

*Discodemus* LeConte, 1862a: 223. Type species: *Zophosis reticulata* Say, 1824, monotypy. Synonymy: LeConte (1866a: 60).

*Conipinus* LeConte, 1862a: 223. Type species: *Eusattus dubius* LeConte, 1851, subsequent designation (Gebien 1938: 284). Synonymy: LeConte (1866a: 60).

*Nesostes* Casey, 1908: 56, 58. Type species: *Eusattus robustus* LeConte, 1866, original designation. Synonymy: Triplehorn (1968b: 379).

*Megasattus* Casey, 1908: 56. Type species: *Eusattus erosus* Horn, 1870, original designation. Synonymy: Triplehorn (1968b: 379).

*Eusattodes* Casey, 1908: 56. Type species: *Eusattus laevis* LeConte, 1866, original designation. Synonymy: Triplehorn (1968b: 379).

*Sphaeriontis* Casey, 1908: 56, 75. Type species: *Eusattus muricatus* LeConte, 1851, original designation. Synonymy: La Rivers (1949: 180).

*Coelosattus* Blaisdell, 1927: 166. Type species: *Coelosattus fortineri* Blaisdell, 1927 (= *Eusattus dilatatus* LeConte, 1851), monotypy. Synonymy: Doyen (1972: 373).

***Eusattus araneosus* (Blaisdell, 1923)** MEX (BS)

*Megasattus araneosus* Blaisdell, 1923: 266.

***Eusattus arenarius* Doyen, 1984** MEX (BC BS)

*Eusattus arenarius* Doyen, 1984b: 75.

***Eusattus aridus* Doyen, 1984** MEX (BC BS)

*Eusattus aridus* Doyen, 1984b: 43.

***Eusattus catalinensis* Doyen, 1984** MEX (BS)

*Eusattus catalinensis* Doyen, 1984b: 45.

***Eusattus catavinus* Doyen, 1984** MEX (BC)

*Eusattus catavinus* Doyen, 1984b: 46.

***Eusattus cedrosensis* Doyen, 1984** MEX (BC)

*Eusattus cedrosensis* Doyen, 1984b: 47.

***Eusattus ceralboensis* Doyen, 1984** MEX (BS)

*Eusattus ceralboensis* Doyen, 1984b: 48.

***Eusattus cienegus* Doyen, 1984** MEX (CO)

*Eusattus cienegus* Doyen, 1984b: 49.

***Eusattus ciliatoides* Doyen, 1984** MEX (BC)

*Eusattus ciliatoides* Doyen, 1984b: 75.

***Eusattus ciliatus* Horn, 1894** MEX (BC)

*Eusattus ciliatus* Horn, 1894b: 422.

***Eusattus convexus* LeConte, 1851** USA (AZ CO KS MO NM OK TX UT WY) MEX (CH DU SO)

*Eusattus convexus* LeConte, 1851: 132.

*Eusattus sculptus* Champion, 1892: 510. Synonymy: Doyen (1984b: 35).

*Eusattus rotundus* Casey, 1908: 72. Synonymy: Doyen (1977: 5).

*Eusattus turgidus* Casey, 1908: 73. Synonymy: Doyen (1977: 5).

*Eusattus subnitens* Casey, 1908: 73. Synonymy: Doyen (1984b: 35).

*Eusattus peropacus* Casey, 1908: 74. Synonymy: Doyen (1977: 5).

*Eusattus acutus* Casey, 1908: 74. Synonymy: Doyen (1977: 5).

*Eusattus quadratus* Casey, 1924: 311. Synonymy: Doyen (1977: 5).

*Eusattus subvelutinus* Casey, 1924: 312. Synonymy: Doyen (1977: 5).

*Eusattus woodgatei* Casey, 1924: 312. Synonymy: Doyen (1977: 5).

***Eusattus costatus* Horn, 1870** USA (CA) MEX (BC BS)

*Eusattus costatus* Horn, 1870: 293.

*Megasattus sternalis* Blaisdell, 1923: 268. Synonymy: Doyen (1984b: 50).

***Eusattus crypticus* Doyen, 1984** MEX (BS)

*Eusattus crypticus* Doyen, 1984b: 90.

***Eusattus depressus* Champion, 1884** MEX (CH NA SI SO)

*Eusattus depressus* Champion, 1884: 75.

*Eusattus puncticeps* Blaisdell, 1923: 269. Synonymy: Doyen (1984b: 87).

***Eusattus difficilis* LeConte, 1851** USA (CA NV) MEX (BC SO)

*Eusattus difficilis* LeConte, 1851: 132.

*Eusattus coquilletti* Linell, 1899: 180. Synonymy: Doyen (1977: 5).

*Eusattus agnatus* Casey, 1908: 70. Synonymy: Doyen (1977: 5).

*Eusattus compositus* Casey, 1908: 71. Synonymy: Doyen (1977: 5).

*Eusattus congener* Casey, 1908: 71. Synonymy: Doyen (1977: 5).

*Eusattus acutangulus* Casey, 1908: 72. Synonymy: Doyen (1977: 5).

***Eusattus dilatatus* LeConte, 1851** USA (AZ CA) MEX (SO)

*Eusattus dilatatus* LeConte, 1851: 132.

*Coelosattus fortineri* Blaisdell, 1927: 167. Synonymy: Doyen (1977: 5).

***Eusattus dubius abditus* Doyen, 1984** MEX (BC)

*Eusattus dubius abditus* Doyen, 1984b: 82.

***Eusattus dubius arizonensis* Doyen, 1984** USA (AZ CA NV)

*Eusattus dubius arizonensis* Doyen, 1984b: 81.

***Eusattus dubius dubius* LeConte, 1851** USA (AZ CA NV UT)

*Eusattus dubius* LeConte, 1851: 132.

*Eusattus nanus* Casey, 1895: 613. Synonymy: Doyen (1977: 5).

*Eusattus oblongulus* Casey, 1908: 67. Synonymy: Doyen (1977: 5).

*Conipinus spaldingi* Casey, 1924: 313. Synonymy: Doyen (1977: 5).

***Eusattus dubius setosus* Doyen, 1984** MEX (BS)

*Eusattus dubius setosus* Doyen, 1984b: 82.

***Eusattus erosus erosus* Horn, 1870** MEX (BS)

*Eusattus erosus* Horn, 1870: 294.

***Eusattus erosus laeviventris* (Blaisdell, 1923)** MEX (BS)

*Megasattus laeviventris* Blaisdell, 1923: 267.

***Eusattus erosus manuelis* (Blaisdell, 1923)** MEX (BS)

*Megasattus erosus manuelis* Blaisdell, 1923: 266.

***Eusattus franciscanus* Doyen, 1984** MEX (BS)

*Eusattus franciscanus* Doyen, 1984b: 54.

***Eusattus hirsutus* Doyen, 1984** USA (NV)

*Eusattus hirsutus* Doyen, 1984b: 66.

***Eusattus laevis* LeConte, 1866** MEX (BS)

*Eusattus laevis* LeConte, 1866b: 113.

***Eusattus mexicanus* Champion, 1892** MEX (CO GE JA)

*Eusattus mexicanus* Champion, 1892: 510.

***Eusattus minimus* Doyen, 1984** MEX (NL)

*Eusattus minimus* Doyen, 1984b: 38.

***Eusattus muricatus diabloensis* Doyen, 1984** MEX (BC)

*Eusattus muricatus diabloensis* Doyen, 1984b: 71.

***Eusattus muricatus muricatus* LeConte, 1851** USA (AZ CA CO ID NM NV OR TX UT WA)

*Eusattus muricatus* LeConte, 1851: 132.

*Sphaeriontis acomana* Casey, 1908: 76. Synonymy: La Rivers (1949: 180).

*Sphaeriontis latissima* Casey, 1924: 310. Synonymy: La Rivers (1949: 180).

*Sphaeriontis fulvescens* Casey, 1924: 310. Synonymy: La Rivers (1949: 180).

***Eusattus nitidipennis* LeConte, 1851** MEX (CO GU NL PU VE ZA)

*Eusattus nitidipennis* LeConte, 1851: 133.

*Eusattus brevis* Champion, 1884: 75. Synonymy: Doyen (1977: 6).

***Eusattus obliteratus* Champion, 1892** MEX (CO DU)

*Eusattus obliteratus* Champion, 1892: 510^19^.

***Eusattus pallidus adustus* Doyen, 1984** MEX (BS)

*Eusattus pallidus adustus* Doyen, 1984b: 84.

***Eusattus pallidus immaculatus* Doyen, 1984** MEX (BS)

*Eusattus pallidus immaculatus* Doyen, 1984b: 85.

***Eusattus pallidus pallidus* Doyen, 1984** MEX (BS)

*Eusattus pallidus pallidus* Doyen, 1984b: 84.

***Eusattus phreatophilus* Doyen, 1984** USA (CA NV)

*Eusattus phreatophilus* Doyen, 1984b: 71.

***Eusattus planulus* Doyen, 1984** MEX (BS)

*Eusattus planulus* Doyen, 1984b: 57.

***Eusattus politus cruzensis* Doyen, 1984** USA (CA)

*Eusattus politus cruzensis* Doyen, 1984b: 93.

***Eusattus politus politus* Horn, 1883** USA (CA)

*Eusattus politus* Horn, 1883: 304.

*Eusattus vanduzeei* Blaisdell, 1921b: 214. Synonymy: Doyen (1984b: 93).

***Eusattus pons* Triplehorn, 1968** USA (TX) MEX (CH CO DU)

*Eusattus pons* Triplehorn, 1968b: 376.

***Eusattus productus* LeConte, 1858** USA (AZ CA) MEX (BC SO)

*Eusattus productus* LeConte, 1858a: 20.

*Eusattus explanatus* Casey, 1908: 68. Synonymy: Doyen (1977: 6).

*Eusattus vicinus* Casey, 1908: 68. Synonymy: Doyen (1977: 6).

*Eusattus lobatus* Casey, 1908: 68. Synonymy: Doyen (1977: 6).

***Eusattus puberulus* LeConte, 1854** USA (TX)

*Eusattus puberulus* LeConte, 1854a: 84.

***Eusattus reticulatus* (Say, 1824)** USA (AZ CO NM OK TX UT) MEX (CH SO)

*Tenebrio gibbus* DeGeer, 1778: 652 [junior primary homonym of *Tenebrio gibbus* Linnaeus, 1767].

*Tenebrio striatus* Retzius, 1783: 134 [junior primary homonym of *Tenebrio striatus* Müller, 1776]. Replacement name for *Tenebrio gibbus* DeGeer, 1778.

*Zophosis reticulata* Say, 1824a: 250. Synonymy: Ferrer and Holston (2011: 252).

*Discodemus corrosus* Casey, 1908: 61. Synonymy: Doyen (1977: 6).

*Discodemus brevipennis* Casey, 1908: 61. Synonymy: Doyen (1977: 6).

*Discodemus elongatulus* Casey, 1908: 61. Synonymy: Doyen (1977: 6).

*Discodemus depressulus* Casey, 1908: 62. Synonymy: Doyen (1977: 6).

*Discodemus subsericeus* Casey, 1908: 62. Synonymy: Doyen (1977: 6).

*Discodemus knausi* Casey, 1908: 62. Synonymy: Doyen (1977: 6).

***Eusattus robustus* LeConte, 1866** USA (CA)

*Eusattus robustus* LeConte, 1866b: 112.

*Eusattus robustus postremus* Casey, 1908: 59. Synonymy: Doyen (1977: 6).

***Eusattus rudei* Doyen, 1984** MEX (BS)

*Eusattus rudei* Doyen, 1984b: 91.

***Eusattus secutus* Horn, 1894** MEX (BS)

*Eusattus secutus* Horn, 1894b: 421.

***Eusattus venosus* Champion, 1892** MEX (CO JA NA)

*Eusattus venosus* Champion, 1892: 509.

***Eusattus vizcainensis* Doyen, 1984** MEX (BC BS)

*Eusattus vizcainensis* Doyen, 1984b: 85.

**Tribe Cryptoglossini LeConte, 1862**

Centrioptérides Lacordaire, 1859: 134 [*nomen oblitum*, see Aalbu 2006: 57]. Type genus: *Centrioptera* Mannerheim, 1843.

Cryptoglossini LeConte, 1862a: 220 [*nomen protectum*]. Type genus: *Cryptoglossa* Solier, 1837.

**Genus *Asbolus* LeConte, 1851** [M]

*Asbolus* LeConte, 1851: 129. Type species: *Asbolus verrucosus* LeConte, 1851, subsequent designation (Aalbu 2005: 721).

***Asbolus laevis* LeConte, 1851** USA (AZ CA) MEX (BC SO)

*Asbolus laevis* LeConte, 1851: 130.

*Cryptoglossa laevis subsimilis* Casey, 1924: 308. Synonymy: Aalbu (2005: 730).

***Asbolus mexicanus angularis* (Horn, 1894)** USA (AZ CA) MEX (BC BS)

*Centrioptera angularis* Horn, 1894b: 414.

***Asbolus mexicanus mexicanus* (Champion, 1884)** USA (NM TX) MEX (CH CO DU NL)

*Cryptoglossa mexicana* Champion, 1884: 73.

*Cryptoglossa granulifera* Champion, 1892: 508. Synonymy: Aalbu (2005: 722).

***Asbolus papillosus* (Triplehorn, 1964)** USA (CA) MEX (SO)

*Cryptoglossa laevis papillosa* Triplehorn, 1964a: 48.

***Asbolus verrucosus* LeConte, 1851** USA (AZ CA NM NV UT) MEX (BC SO)

*Asbolus verrucosus* LeConte, 1851: 129.

*Cryptoglossa verrucosa carinulatus* Blaisdell, 1945: 25. Synonymy: Aalbu (2005: 724).

**Genus *Cryptoglossa* Solier, 1837** [F]

*Cryptoglossa* Solier, 1837: 680. Type species: *Cryptoglossa bicostata* Solier, 1837, monotypy.

*Centrioptera* Mannerheim, 1843: 279. Type species: *Centrioptera caraboides* Mannerheim, 1843, monotypy. Synonymy: Aalbu et al. (2002: 486).

*Oochila* LeConte, 1862a: 220. Type species: *Asbolus infaustus* LeConte, 1854, original designation. Synonymy (with *Centrioptera* Mannerheim): Horn (1870: 278).

*Amblycyphus* Motschulsky, 1870: 401. Type species: *Amblycyphus asperatus* Motschulsky, 1870 (= *Centrioptera pectoralis* Blaisdell, 1921), monotypy. Synonymy: Aalbu et al. (1995: 483).

***Cryptoglossa asperata* (Horn, 1870)** MEX (BS)

*Centrioptera asperata* Horn, 1870: 279.

*Centrioptera asperata discreta* Blaisdell, 1923: 249. Synonymy: Aalbu (2005: 709).

*Centrioptera asperata subornata* Blaisdell, 1923: 249. Synonymy: Aalbu (2005: 709).

*Centrioptera asperata planata* Blaisdell, 1923: 250. Synonymy: Aalbu (2005: 709).

***Cryptoglossa bicostata* Solier, 1837** MEX (OA PU)

*Cryptoglossa bicostata* Solier, 1837: 681.

***Cryptoglossa caraboides* (Mannerheim, 1843)** MEX (GE MO PU)

*Centrioptera caraboides* Mannerheim, 1843: 280.

***Cryptoglossa infausta* (LeConte, 1854)** USA (TX) MEX (CO DU TA)

*Asbolus infaustus* LeConte, 1854a: 84.

*Centrioptera spiculosa* Champion, 1892: 508. Synonymy: Champion (1893a: 572).

*Centrioptera texana* Blaisdell, 1924b: 88. Synonymy: Aalbu (2005: 705).

***Cryptoglossa michelbacheri* (Blaisdell, 1943)** MEX (BS)

*Centrioptera michelbacheri* Blaisdell, 1943: 222.

***Cryptoglossa muricata* (LeConte, 1851)** USA (AZ CA NV UT) MEX (BC SO)

*Centrioptera muricata* LeConte, 1851: 142.

*Centrioptera utensis* Casey, 1907: 513. Synonymy: Aalbu (2005: 712).

*Centrioptera sculptiventris* Blaisdell, 1923: 247. Synonymy: Aalbu (2005: 712).

*Centrioptera serrata* Casey, 1924: 306. Synonymy: Aalbu (2005: 712).

*Centrioptera elongata* Casey, 1924: 306. Synonymy: Aalbu (2005: 712).

***Cryptoglossa seriata cerralvoensis* Aalbu, 2005** MEX (BS)

*Cryptoglossa seriata cerralvoensis* Aalbu, 2005: 721.

***Cryptoglossa seriata seriata* LeConte, 1861** USA (AZ CA) MEX (BS)

*Cryptoglossa seriata* LeConte, 1861a: 337.

***Cryptoglossa spiculifera pectoralis* (Blaisdell, 1921)** USA (CA) MEX (BC BS)

*Amblycyphus asperatus* Motschulsky, 1870: 404 [junior secondary homonym of *Cryptoglossa asperata* (Horn, 1870)^20^].

*Centrioptera pectoralis* Blaisdell, 1921b: 198. Synonymy: Aalbu et al. (1995: 483).

*Centrioptera dulzurae* Blaisdell, 1921b: 199. Synonymy: Aalbu et al. (1995: 483).

*Centrioptera chamberlini* Blaisdell, 1923: 246. Synonymy: Aalbu (2005: 717).

***Cryptoglossa spiculifera spiculifera* (LeConte, 1861)** MEX (BS)

*Centrioptera spiculifera* LeConte, 1861a: 337.

***Cryptoglossa variolosa* (Horn, 1870)** USA (AZ CA NM) MEX (SI SO)

*Centrioptera variolosa* Horn, 1870: 280.

**Genus *Schizillus* Horn, 1874** [M]

*Schizillus* Horn, 1874a: 33. Type species: *Schizillus laticeps* Horn, 1874, monotypy.

***Schizillus laticeps* Horn, 1874** USA (AZ CA NV UT) MEX (BC)

*Schizillus laticeps* Horn, 1874a: 33.

*Schizillus convexus* Blaisdell, 1921b: 203. Synonymy: Aalbu (2005: 732).

*Schizillus lomae* Blaisdell, 1921b: 206. Synonymy: Aalbu (2005: 732).

*Schizillus opacus* Casey, 1924: 307. Synonymy: Aalbu (2005: 732).

***Schizillus nunenmacheri* Blaisdell, 1921** USA (AZ CA NV UT)

*Schizillus nunenmacheri* Blaisdell, 1921b: 204.

*Schizillus beali* Parker, 1955: 148. Synonymy: Aalbu (2005: 734).

**Tribe Edrotini Lacordaire, 1859**

Édrotides Lacordaire, 1859: 31. Type genus: *Edrotes* LeConte, 1851.

Triorophi LeConte and Horn, 1883: 362. Type genus: *Triorophus* LeConte, 1851.

Auchmobii LeConte and Horn, 1883: 362. Type genus: *Auchmobius* LeConte, 1851.

Trimytini Casey, 1907: 278. Type genus: *Trimytis* Leconte, 1851.

Eurymetoponini Casey, 1907: 278. Type genus: *Eurymetopon* Eschscholtz, 1831.

Trientomini Casey, 1907: 278. Type genus: *Trientoma* Solier, 1835.

**Genus *Armalia* Casey, 1907** [F]

*Armalia* Casey, 1907: 289, 330. Type species: *Emmenastus texanus* LeConte, 1866, original designation.

***Armalia alata* (Champion, 1884)** GUA NIC

*Emmenastus alatus* Champion, 1884: 13.

*Emmenastus salvini* Champion, 1884: 13. Synonymy: Champion (1892: 482).

***Armalia angularis* Casey, 1907** USA (TX)

*Armalia angularis* Casey, 1907: 331.

***Armalia belti* (Champion, 1884)** MEX (YU) GUA HON NIC

*Emmenastus belti* Champion, 1884: 11.

*Emmenastus rotundicollis* Champion, 1884: 11. Synonymy: Champion (1892: 480).

*Emmenastus intermedius* Champion, 1884: 12. Synonymy: Champion (1892: 480).

***Armalia brevipennis* (Champion, 1884)** MEX (NA)

*Emmenastus brevipennis* Champion, 1884: 10.

***Armalia canaliculata* (Champion, 1884)** MEX (SI)

*Emmenastus canaliculatus* Champion, 1884: 10.

***Armalia chiriquensis* (Champion, 1884)** PAN / SA

*Emmenastus chiriquensis* Champion, 1884: 9.

***Armalia longicornis* (Champion, 1884)** GUA

*Emmenastus longicornis* Champion, 1884: 9.

***Armalia solitaria* (Champion, 1884)** MEX (OA)

*Emmenastus solitarius* Champion, 1884: 11.

***Armalia texana* (LeConte, 1866)** USA (TX)

*Emmenastus texanus* LeConte, 1866b: 108.

***Armalia variabilis* (Champion, 1884)** MEX (VE) HON

*Emmenastus variabilis* Champion, 1884: 10.

**Genus *Auchmobius* LeConte, 1851** [M]

*Auchmobius* LeConte, 1851: 139. Type species: *Auchmobius sublaevis* LeConte, 1851, monotypy.

***Auchmobius angelicus* Blaisdell, 1934** USA (CA)

*Auchmobius angelicus* Blaisdell, 1934b: 249.

***Auchmobius parvicollis* Blaisdell, 1934** USA (CA)

*Auchmobius parvicollis* Blaisdell, 1934b: 246.

***Auchmobius picipes* Blaisdell, 1934** USA (CA)

*Auchmobius picipes* Blaisdell, 1934b: 252.

***Auchmobius sanfordi* Blaisdell, 1934** USA (CA)

*Auchmobius sanfordi* Blaisdell, 1934b: 257.

***Auchmobius slevini* Blaisdell, 1934** USA (CA)

*Auchmobius slevini* Blaisdell, 1934b: 243.

***Auchmobius subboreus* Blaisdell, 1934** USA (CA NV)

*Auchmobius subboreus* Blaisdell, 1934b: 254.

***Auchmobius sublaevis* LeConte, 1851** USA (CA)

*Auchmobius sublaevis* LeConte, 1851: 140.

***Auchmobius subovalis* Blaisdell, 1934** USA (CA)

*Auchmobius subovalis* Blaisdell, 1934b: 238.

**Genus *Chilometopon* Horn, 1874** [N]

*Chilometopon* Horn, 1874a: 31. Type species: *Trimytis abnormis* Horn, 1870, subsequent designation (Casey 1907: 367).

*Prometopion* Casey, 1907: 366, 370. Type species: *Prometopion amplipenne* Casey, 1907 (= *Chilometopon helopioides* Horn, 1874), original designation. Synonymy: MacLachlan and Olson (1990: 72).

***Chilometopon abnorme* (Horn, 1870)** USA (AZ CA NV OR UT) MEX (BC SO)

*Trimytis abnormis* Horn, 1870: 261.

*Chilometopon castaneum* Casey, 1907: 373. Synonymy: MacLachlan and Olson (1990: 74).

*Chilometopon brevipenne* Casey, 1907: 374. Synonymy: MacLachlan and Olson (1990: 74).

*Chilometopon ensifer* Casey, 1907: 374. Synonymy: MacLachlan and Olson (1990: 74).

***Chilometopon brachystomum* Doyen, 1983** USA (AZ CA NV) MEX (BC)

*Chilometopon brachystomum* Doyen, 1983: 81.

***Chilometopon cribricolle* Blaisdell, 1923** MEX (BS)

*Chilometopon cribricolle* Blaisdell, 1923: 230.

***Chilometopon helopioides* Horn, 1874** USA (AZ CA ID NM NV UT) MEX (BC)

*Chilometopon helopioides* Horn, 1874a: 31.

*Prometopion amplipenne* Casey, 1907: 372. Synonymy: MacLachlan and Olson (1990: 78).

***Chilometopon microps* MacLachlan and Olson, 1990** USA (CA)

*Chilometopon microps* MacLachlan and Olson, 1990: 76.

***Chilometopon pallidum* Casey, 1890** USA (AZ CA NM NV TX UT) MEX (BC CH)

*Chilometopon pallidum* Casey, 1890b: 367.

***Chilometopon rugiceps* Blaisdell, 1923** MEX (BC)

*Chilometopon rugiceps* Blaisdell, 1923: 229.

**Genus *Cryptadius* LeConte, 1851** [M]

*Cryptadius* LeConte, 1851: 140. Type species: *Cryptadius inflatus* LeConte, 1851, monotypy.

***Cryptadius inflatus blaisdelli* Thomas, 1985** MEX (BS)

*Cryptadius inflatus blaisdelli* Thomas, 1985: 197.

***Cryptadius inflatus inflatus* LeConte, 1851** USA (CA) MEX (BC)

*Cryptadius inflatus* LeConte, 1851: 140.

*Cryptadius oviformis* Casey, 1907: 328. Synonymy: Thomas (1985: 196).

*Cryptadius punctipennis* Casey, 1907: 328. Synonymy: Thomas (1985: 196).

*Cryptadius curvipes* Casey, 1907: 329. Synonymy: Thomas (1985: 196).

***Cryptadius sonorae* Berry, 1974** MEX (BS SO)

*Cryptadius sonorae* Berry, 1974: 175.

***Cryptadius tarsalis* Blaisdell, 1923** MEX (BC BS SO)

*Cryptadius tarsalis* Blaisdell, 1923: 212.

*Cryptadius angulatus* Blaisdell, 1923: 210. Synonymy: Thomas (1985: 198).

*Cryptadius sinuatus* Blaisdell, 1923: 211. Synonymy: Thomas (1985: 198).

*Cryptadius andrewsi* Berry, 1977: 561. Synonymy: Thomas (1985: 198).

**Genus *Ditaphronotus* Casey, 1907** [M]

*Ditaphronotus* Casey, 1907: 341. Type species: *Emmenastus foveicollis* Champion, 1884, original designation.

***Ditaphronotus championi* Casey, 1907** NIC

*Ditaphronotus championi* Casey, 1907: 342.

***Ditaphronotus confusus* (Champion, 1884)** MEX (CI) GUA

*Emmenastus confusus* Champion, 1884: 15.

***Ditaphronotus foveicollis* (Champion, 1884)** GUA NIC CRI

*Emmenastus foveicollis* Champion, 1884: 14.

***Ditaphronotus laevicollis* (Champion, 1884)** PAN

*Emmenastus laevicollis* Champion, 1884: 15.

**Genus *Edrotes* LeConte, 1851** [M]

*Edrotes* LeConte, 1851: 140. Type species: *Edrotes ventricosus* LeConte, 1851, monotypy.

*Hedrotes* Gemminger [in Gemminger and Harold], 1870: 1816. Unjustified emendation of *Edrotes* LeConte, 1851, not in prevailing usage.

**Subgenus Edrotes LeConte, 1851**

*Edrotes* LeConte, 1851: 140. Type species: *Edrotes ventricosus* LeConte, 1851, monotypy.

***Edrotes fossor* Triplehorn, 1972** MEX (BS)

*Edrotes fossor* Triplehorn, 1972: 27.

***Edrotes leechi* Doyen, 1968** USA (AZ CO UT)

*Edrotes leechi* Doyen, 1968: 218.

***Edrotes rotundus* (Say, 1824)** USA (AZ CO NM TX UT) MEX (BC CH)

*Pimelia rotunda* Say, 1824a: 251.

*Edrotes globosus* Casey, 1890a: 175. Synonymy: La Rivers (1947a: 325).

*Edrotes inflatus* Casey, 1907: 454. Synonymy: La Rivers (1947a: 325).

*Edrotes puncticeps* Casey, 1907: 454. Synonymy: La Rivers (1947a: 325).

*Edrotes intermixtus* Casey, 1907: 455. Synonymy: La Rivers (1947a: 325).

*Edrotes oblongulus* Casey, 1907: 455. Synonymy: La Rivers (1947a: 325).

*Edrotes lineatus* Casey, 1907: 456. Synonymy: La Rivers (1947a: 325).

*Edrotes subaequalis* Casey, 1907: 456. Synonymy: La Rivers (1947a: 325).

*Edrotes angustulus* Casey, 1907: 456. Synonymy: La Rivers (1947a: 325).

*Edrotes desertus* Blaisdell, 1943: 212. Synonymy: La Rivers (1947a: 325).

***Edrotes ventricosus* LeConte, 1851** USA (AZ CA ID NV OR) MEX (BC BS SO)

*Edrotes ventricosus* LeConte, 1851: 141.

*Edrotes nitidus* Casey, 1890a: 175. Synonymy: La Rivers (1947a: 321).

*Edrotes orbus* Casey, 1907: 452. Synonymy: La Rivers (1947a: 321).

*Edrotes angusticollis* Casey, 1907: 452. Synonymy: La Rivers (1947a: 321).

*Edrotes longipennis* Casey, 1907: 453. Synonymy: La Rivers (1947a: 321).

*Edrotes mexicanus* Blaisdell, 1923: 241. Synonymy: La Rivers (1947a: 321).

*Edrotes asperatus* Blaisdell, 1923: 241. Synonymy: La Rivers (1947a: 321).

*Edrotes laticollis* Casey, 1924: 300. Synonymy: La Rivers (1947a: 321).

*Edrotes longicornis* Casey, 1924: 300. Synonymy: La Rivers (1947a: 321).

*Edrotes variipilis* Casey, 1924: 301. Synonymy: La Rivers (1947a: 321).

*Edrotes barrowsi* Dajoz, 1999: 320. **New synonymy** [RLA].

**Subgenus Odrotes La Rivers, 1947**

*Odrotes* La Rivers, 1947a: 320. Type species: *Edrotes arens* La Rivers, 1947, monotypy.

***Edrotes arens* La Rivers, 1947** USA (AZ CA) MEX (BC)

*Edrotes arens* La Rivers, 1947a: 320.

**Genus *Emmenastrichus* Horn, 1894** [M]

*Emmenastrichus* Horn, 1894b: 413. Type species: *Emmenastrichus cribratus* Horn, 1894, subsequent designation (Casey 1907: 289).

***Emmenastrichus cribratus* Horn, 1894** MEX (BS)

*Emmenastrichus cribratus* Horn, 1894b: 413.

***Emmenastrichus erosus* Horn, 1894** MEX (BS)

*Emmenastrichus erosus* Horn, 1894b: 414.

**Genus *Emmenides* Casey, 1907** [M]

*Emmenides* Casey, 1907: 329. Type species: *Emmenastus punctatus* LeConte, 1866, original designation.

***Emmenides apicalis* Blaisdell, 1923** MEX (BS)

*Emmenides apicalis* Blaisdell, 1923: 215.

***Emmenides catalinae* Blaisdell, 1923** MEX (BS)

*Emmenides catalinae* Blaisdell, 1923: 216.

***Emmenides igualensis* (Champion, 1892)** MEX (GE)

*Emmenastus igualensis* Champion, 1892: 484.

***Emmenides obsoletus* Blaisdell, 1923** MEX (BS)

*Emmenides obsoletus* Blaisdell, 1923: 216.

***Emmenides punctatus* (LeConte, 1866)** USA (AZ TX) MEX (BS)

*Emmenastus punctatus* LeConte, 1866b: 106.

***Emmenides subdescalceatus* Blaisdell, 1923** MEX (BS)

*Emmenides subdescalceatus* Blaisdell, 1923: 213.

**Genus *Eremocantor* Smith and Wirth, 2016** [M]

*Eremocantor* Smith and Wirth, 2016: 582. Type species: *Eremocantor marioni* Smith and Wirth, 2016, original designation.

***Eremocantor marioni* Smith and Wirth, 2016** USA (TX)

*Eremocantor marioni* Smith and Wirth, 2016: 583.

**Genus *Eschatomoxys* Blaisdell, 1935** [M]

*Eschatomoxys* Blaisdell, 1935d: 125. Type species: *Eschatomoxys wagneri* Blaisdell, 1935, original designation.

***Eschatomoxys andrewsi* Aalbu and Thomas, 2008** USA (CA)

*Eschatomoxys andrewsi* Aalbu and Thomas [in Pape et al.], 2008: 529.

***Eschatomoxys paco* Aalbu and Thomas, 2008** MEX (BC)

*Eschatomoxys paco* Aalbu and Thomas [in Pape et al.], 2008: 527.

***Eschatomoxys pholeter* Thomas and Pape, 2008** USA (AZ)

*Eschatomoxys pholeter* Thomas and Pape [in Pape et al.], 2008: 525.

***Eschatomoxys rosei* Aalbu and Thomas, 2008** MEX (BC)

*Eschatomoxys rosei* Aalbu and Thomas [in Pape et al.], 2008: 530.

***Eschatomoxys tanneri* Sorenson and Stones, 1959** USA (AZ UT)

*Eschatomoxys tanneri* Sorenson and Stones, 1959: 63.

***Eschatomoxys wagneri* Blaisdell, 1935** USA (AZ CA)

*Eschatomoxys wagneri* Blaisdell, 1935d: 125.

**Genus *Eurymetopon* Eschscholtz, 1831** [N]

*Eurymetopon* Eschscholtz, 1831: 5, 8. Type species: *Eurymetopon rufipes* Eschscholtz, 1831, subsequent designation (Casey 1907: 288).

***Eurymetopon ochraceum* Eschscholtz, 1831** USA (CA)

*Eurymetopon ochraceum* Eschscholtz, 1831: 8.

***Eurymetopon rufipes* Eschscholtz, 1831** USA (AZ CA) MEX (BS SO)

*Eurymetopon rufipes* Eschscholtz, 1831: 8.

**Genus *Garridoa* Marcuzzi, 1985** [F]

*Garridoa* Marcuzzi, 1985: 180. Type species: *Garridoa kaszabi* Marcuzzi, 1985, monotypy.

***Garridoa kaszabi* Marcuzzi, 1985** CUB

*Garridoa kaszabi* Marcuzzi, 1985: 180.

**Genus *Hylocrinus* Casey, 1907** [M]

*Hylocrinus* Casey, 1907: 289, 331. Type species: *Eurymetopon longulum* LeConte, 1851, original designation.

**Subgenus Hylocrinus Casey, 1907**

*Hylocrinus* Casey, 1907: 289, 331. Type species: *Eurymetopon longulum* LeConte, 1851, original designation.

***Hylocrinus ambiguus* (Champion, 1884)** PAN

*Emmenastus ambiguus* Champion, 1884: 13.

***Hylocrinus angustus* (Casey, 1890)** USA (AZ)

*Emmenastus angustus* Casey, 1890b: 352.

***Hylocrinus blaisdelli* Casey, 1907** USA (CA)

*Hylocrinus blaisdelli* Casey, 1907: 336.

***Hylocrinus breviusculus* Casey, 1907** USA (TX)

*Hylocrinus breviusculus* Casey, 1907: 334.

***Hylocrinus cunctans* Casey, 1907** USA (TX)

*Hylocrinus cunctans* Casey, 1907: 336.

***Hylocrinus delicatulus* Casey, 1907** USA (AZ NV UT)

*Hylocrinus delicatulus* Casey, 1907: 334.

***Hylocrinus depressulus* Casey, 1907** USA (CA)

*Hylocrinus depressulus* Casey, 1907: 335.

***Hylocrinus filitarsis* Casey, 1907** USA (CA)

*Hylocrinus filitarsis* Casey, 1907: 333.

***Hylocrinus guatemalensis* (Champion, 1884)** GUA

*Emmenastus guatemalensis* Champion, 1884: 14.

***Hylocrinus longulus* (LeConte, 1851)** USA (AZ CA) MEX (BC SO)

*Eurymetopon longulum* LeConte, 1851: 139.

***Hylocrinus magnus* Blaisdell, 1923** MEX (SO)

*Hylocrinus magnus* Blaisdell, 1923: 219.

***Hylocrinus tenuis* Casey, 1907** USA (AZ)

*Hylocrinus tenuis* Casey, 1907: 333.

**Subgenus Locrodes Casey, 1907**

*Locrodes* Casey, 1907: 332. Type species: *Emmenastus piceus* Casey, 1890, **present designation**.

***Hylocrinus brunnescens* Casey, 1907** USA (UT)

*Hylocrinus brunnescens* Casey, 1907: 338.

***Hylocrinus fraternus* Casey, 1907** USA (ID UT)

*Hylocrinus fraternus* Casey, 1907: 338.

***Hylocrinus insularis* Blaisdell, 1923** MEX (BS)

*Hylocrinus insularis* Blaisdell, 1923: 218.

***Hylocrinus laborans* Casey, 1907** USA (NV UT)

*Hylocrinus laborans* Casey, 1907: 337.

***Hylocrinus mexicanus* (Champion, 1892)** MEX (FD)

*Emmenastus mexicanus* Champion, 1892: 481.

***Hylocrinus oblongulus* Casey, 1907** USA (CA) MEX (BC BS)

*Hylocrinus oblongulus* Casey, 1907: 337.

***Hylocrinus parallelus* (Champion, 1884)** MEX (JA ME MO OA PU SI)

*Emmenastus parallelus* Champion, 1884: 12.

***Hylocrinus piceus* (Casey, 1890)** USA (CA)

*Emmenastus piceus* Casey, 1890b: 353.

***Hylocrinus seriatus* (Champion, 1892)** MEX (OA)

*Emmenastus seriatus* Champion, 1892: 482.

***Hylocrinus subapterus* (Champion, 1892)** MEX (DU)

*Emmenastus subapterus* Champion, 1892: 481.

***Hylocrinus tenebrosus* (Champion, 1884)** MEX (AG FD GU)

*Emmenastus tenebrosus* Champion, 1884: 12.

***Hylocrinus umbrosus* Casey, 1907** USA (UT)

*Hylocrinus umbrosus* Casey, 1907: 338.

**Subgenus Paravius Casey, 1907**

*Paravius* Casey, 1907: 332. Type species: *Emmenastus marginatus* Casey, 1890, monotypy.

***Hylocrinus marginatus* (Casey, 1890)** MEX (BC)

*Emmenastus marginatus* Casey, 1890b: 351.

***Hylocrinus vicinus* (Champion, 1884)** USA (CA)

*Emmenastus vicinus* Champion, 1884: 8.

**Genus *Melanastus* Casey, 1907** [M]

*Melanastus* Casey, 1907: 289. Type species: *Eurymetopon atrum* LeConte, 1851, original designation.

***Melanastus acuminatus* Casey, 1907** USA (CO)

*Melanastus acuminatus* Casey, 1907: 362.

**Figure 14.**
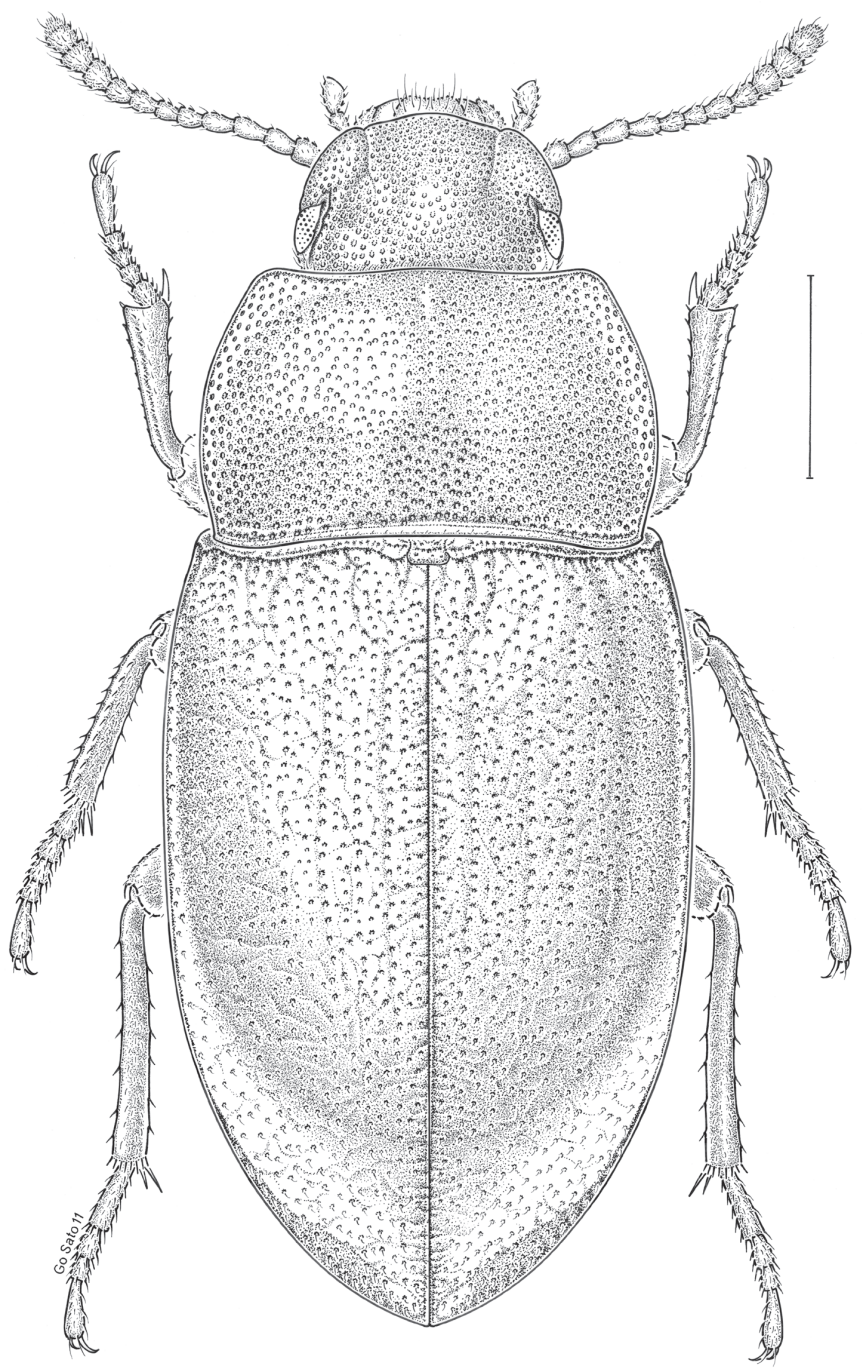
*Melanastus acutus* (Horn, 1870). Scale bar = 1 mm.

***Melanastus acutus* (Horn, 1870)** [Fig. 14] CAN (AB SK) USA (NE)

*Emmenastus acutus* Horn, 1870: 270.

***Melanastus aequicollis* Casey, 1907** USA (CA)

*Melanastus aequicollis* Casey, 1907: 360.

***Melanastus ater* (LeConte, 1851)** USA (CA ID OR)

*Eurymetopon atrum* LeConte, 1851: 139.

***Melanastus coarcticollis* (Casey, 1890)** USA (NM)

*Emmenastus coarcticollis* Casey, 1890b: 364.

***Melanastus crassicornis* (Casey, 1890)** USA (CA)

*Emmenastus crassicornis* Casey, 1890b: 363.

***Melanastus exiguus* Casey, 1907** USA (CO)

*Melanastus exiguus* Casey, 1907: 363.

***Melanastus exoletus* Casey, 1907** USA (CA)

*Melanastus exoletus* Casey, 1907: 357.

***Melanastus fallax* (Casey, 1890)** USA (NM)

*Emmenastus fallax* Casey, 1890b: 361.

***Melanastus finitimus* Casey, 1907** USA (CO)

*Melanastus finitimus* Casey, 1907: 359.

***Melanastus implicans* Casey, 1907** USA (CO)

*Melanastus implicans* Casey, 1907: 358.

***Melanastus lucidulus* Casey, 1907** USA (CA)

*Melanastus lucidulus* Casey, 1907: 358.

***Melanastus ludius* Casey, 1907** USA (UT)

*Melanastus ludius* Casey, 1907: 361.

***Melanastus moestus* Casey, 1907** USA (CA)

*Melanastus moestus* Casey, 1907: 355.

***Melanastus nitidus* (Casey, 1890)** USA (AZ)

*Emmenastus nitidus* Casey, 1890b: 362.

***Melanastus nuperus* Casey, 1907** USA (AZ)

*Melanastus nuperus* Casey, 1907: 364.

***Melanastus obesus* (LeConte, 1851)** USA (CA) MEX (BC BS)

*Eurymetopon obesum* LeConte, 1851: 139.

*Emmenastus nanulus* Casey, 1884: 45. Synonymy: Casey (1907b: 363).

***Melanastus obscurus*** Blaisdell, 1923 MEX (SO)

*Melanastus obscurus* Blaisdell, 1923: 226.

***Melanastus obtusus* (LeConte, 1866)** USA (CA)

*Emmenastus obtusus* LeConte, 1866b: 107.

***Melanastus otiosus* Casey, 1907** USA (CA)

*Melanastus otiosus* Casey, 1907: 358.

***Melanastus parvus* Casey, 1907** USA (CO)

*Melanastus parvus* Casey, 1907: 362.

***Melanastus sonoricus* Casey, 1907** MEX (CH MI)

*Melanastus sonoricus* Casey, 1907: 364.

***Melanastus sterilis* Casey, 1907** USA (CA)

*Melanastus sterilis* Casey, 1907: 357.

***Melanastus texanus* Blaisdell, 1926** USA (TX)

*Melanastus texanus* Blaisdell, 1926b: 22.

***Melanastus thoracicus* (Casey, 1890)** USA (CA)

*Emmenastus thoracicus* Casey, 1890b: 362.

***Melanastus vegrandis* Casey, 1907** USA (CA)

*Melanastus vegrandis* Casey, 1907: 360.

**Genus *Mencheres* Champion, 1884** [M]

*Mencheres* Champion, 1884: 5. Type species: *Mencheres nicaraguensis* Champion, 1884, subsequent designation (Lucas 1920: 403).

***Mencheres elongatus* Champion, 1884** GUA

*Mencheres elongatus* Champion, 1884: 6.

***Mencheres nicaraguensis* Champion, 1884** NIC

*Mencheres nicaraguensis* Champion, 1884: 5.

**Genus *Mesabates* Champion, 1884** [M]

*Mesabates* Champion, 1884: 3. Type species: *Mesabates latifrons* Champion, 1884, monotypy.

***Mesabates latifrons* Champion, 1884** MEX (OA PU)

*Mesabates latifrons* Champion, 1884: 3.

***Mesabates spissicornis* Champion, 1892** MEX (SI)

*Mesabates spissicornis* Champion, 1892: 479.

**Genus *Mesabatodes* Casey, 1907** [M]

*Mesabatodes* Casey, 1907: 517. Type species: *Mesabates inaequalis* Champion, 1892, original designation.

***Mesabatodes inaequalis* (Champion, 1892)** MEX (AG CH DU)

*Mesabates inaequalis* Champion, 1892: 480.

**Genus *Metoponium* Casey, 1907** [N]

*Metoponium* Casey, 1907: 288. Type species: *Eurymetopon abnorme* LeConte, 1851, original designation.

**Subgenus Metoponiopsis Casey, 1907**

*Metoponiopsis* Casey, 1907: 290. Type species: *Eurymetopon bicolor* Horn, 1870, monotypy.

***Metoponium bicolor* (Horn, 1870)** USA (AZ CA) MEX (BC)

*Eurymetopon bicolor* Horn, 1870: 268.

**Subgenus Metoponium Casey, 1907**

*Metoponium* Casey, 1907: 288. Type species: *Eurymetopon abnorme* LeConte, 1851, original designation.

***Metoponium abnorme abnorme* (LeConte, 1851)** USA (CA)

*Eurymetopon abnorme* LeConte, 1851: 138.

***Metoponium abnorme faustum* Casey, 1907** USA (CA)

*Metoponium faustum* Casey, 1907: 292.

***Metoponium abnorme laticolle* Casey, 1907** USA (AZ) MEX (BC BS)

*Metoponium laticolle* Casey, 1907: 291.

***Metoponium angelicum* Blaisdell, 1923** MEX (BC)

*Metoponium angelicum* Blaisdell, 1923: 203.

***Metoponium arizonicum* Casey, 1907** USA (AZ)

*Metoponium arizonicum* Casey, 1907: 294.

***Metoponium candidum* Casey, 1907** USA (AZ) MEX (SO)

*Metoponium candidum* Casey, 1907: 292.

***Metoponium cognitum* Casey, 1907** USA (TX)

*Metoponium cognitum* Casey, 1907: 305.

***Metoponium concors* Casey, 1907** USA (CA)

*Metoponium concors* Casey, 1907: 305.

***Metoponium congener* (Casey, 1890)** USA (TX)

*Eurymetopon congener* Casey, 1890b: 333.

***Metoponium convexicolle* (LeConte, 1851)** USA (AZ CA NV) MEX (BC BS)

*Eurymetopon convexicolle* LeConte, 1851: 139.

***Metoponium crassum* Casey, 1907** USA (AZ)

*Metoponium crassum* Casey, 1907: 299.

***Metoponium cribriceps* Casey, 1907** USA (NM TX)

*Metoponium cribriceps* Casey, 1907: 305.

***Metoponium cylindricum* (Casey, 1890)** USA (CA)

*Eurymetopon cylindricum* Casey, 1890b: 337.

***Metoponium dubium* (Casey, 1884)** USA (AZ)

*Eurymetopon dubium* Casey, 1884: 44.

*Eurymetopon carbonatum* Casey, 1884: 43. Synonymy: Horn (1885b: 110).

***Metoponium edax* Casey, 1907** USA (CA)

*Metoponium edax* Casey, 1907: 309.

***Metoponium egregium* Casey, 1907** USA (CA)

*Metoponium egregium* Casey, 1907: 300.

***Metoponium emarginatum* (Casey, 1884)** USA (AZ)

*Eurymetopon emarginatum* Casey, 1884: 41.

*Eurymetopon piceum* Casey, 1884: 40. Synonymy: Horn (1885b: 110).

*Eurymetopon papagonum* Casey, 1884: 42. Synonymy: Horn (1885b: 110).

*Eurymetopon sculptile* Casey, 1884: 44. Synonymy: Horn (1885b: 110).

***Metoponium erosum* Blaisdell, 1943** MEX (BS)

*Metoponium erosum* Blaisdell, 1943: 175.

***Metoponium extensum* Casey, 1907** USA (AZ)

*Metoponium extensum* Casey, 1907: 300.

***Metoponium fatigans* Casey, 1907** USA (AZ)

*Metoponium fatigans* Casey, 1907: 304.

***Metoponium fusculum* (Casey, 1890)** USA (AZ CA)

*Eurymetopon fusculum* Casey, 1890b: 335.

***Metoponium gravidum* Casey, 1907** USA (CA)

*Metoponium gravidum* Casey, 1907: 308.

***Metoponium gulosum* Casey, 1907** USA (CA)

*Metoponium gulosum* Casey, 1907: 307.

***Metoponium hebes* Casey, 1907** USA (AZ)

*Metoponium hebes* Casey, 1907: 301.

***Metoponium insulare* Casey, 1907** USA (CA)

*Metoponium insulare* Casey, 1907: 308.

***Metoponium integer* Casey, 1907** USA (CA)

*Metoponium integer* Casey, 1907: 310.

***Metoponium ludificans* Casey, 1907** USA (TX)

*Metoponium ludificans* Casey, 1907: 303.

***Metoponium molestum* Casey, 1907** USA (CA)

*Metoponium molestum* Casey, 1907: 309.

***Metoponium nevadense* Casey, 1907** USA (NV)

*Metoponium nevadense* Casey, 1907: 307.

***Metoponium opacipenne* Casey, 1907** USA (CA)

*Metoponium opacipenne* Casey, 1907: 309.

***Metoponium pacificum* Blaisdell, 1923** MEX (BS)

*Metoponium pacificum* Blaisdell, 1923: 202.

***Metoponium pallescens* Casey, 1907** USA (AZ)

*Metoponium pallescens* Casey, 1907: 293.

***Metoponium parvuliceps* Casey, 1907** USA (AZ)

*Metoponium parvuliceps* Casey, 1907: 296.

***Metoponium perforatum anceps* Casey, 1907** USA (NM)

*Metoponium anceps* Casey, 1907: 294.

***Metoponium perforatum congruens* Casey, 1907** USA (NM)

*Metoponium congruens* Casey, 1907: 293.

***Metoponium perforatum perforatum* (Casey, 1890)** USA (AZ)

*Eurymetopon perforatum* Casey, 1890b: 334.

***Metoponium phoenicis* Casey, 1907** USA (AZ)

*Metoponium phoenicis* Casey, 1907: 301.

***Metoponium politum* (Casey, 1890)** USA (TX)

*Eurymetopon politum* Casey, 1890b: 338.

***Metoponium probatum* Casey, 1907** USA (CA)

*Metoponium probatum* Casey, 1907: 310.

***Metoponium procerum* Casey, 1907** USA (AZ)

*Metoponium procerum* Casey, 1907: 297.

***Metoponium prolixum* Casey, 1907** USA (AZ)

*Metoponium prolixum* Casey, 1907: 298.

***Metoponium rufescens* Casey, 1907** USA (AZ)

*Metoponium rufescens* Casey, 1907: 302.

***Metoponium rufopiceum* Casey, 1907** USA (AZ)

*Metoponium rufopiceum* Casey, 1907: 296.

***Metoponium saginatum* Casey, 1907** USA (TX)

*Metoponium saginatum* Casey, 1907: 296.

***Metoponium socium socium* Casey, 1907** USA (AZ)

*Metoponium socium* Casey, 1907: 295.

***Metoponium socium subsimile* Casey, 1907** USA (AZ)

*Metoponium subsimile* Casey, 1907: 295.

***Metoponium subovale* Casey, 1907** USA (UT)

*Metoponium subovale* Casey, 1907: 307.

***Metoponium tersum* Casey, 1907** USA (CA)

*Metoponium tersum* Casey, 1907: 306.

***Metoponium testaceum* Casey, 1907** USA (CA)

*Metoponium testaceum* Casey, 1907: 303.

***Metoponium transversum* Blaisdell, 1943** MEX (BS)

*Metoponium transversum* Blaisdell, 1943: 174.

***Metoponium truncaticeps* Casey, 1907** USA (AZ)

*Metoponium truncaticeps* Casey, 1907: 299.

**Genus *Micrarmalia* Casey, 1907** [F]

*Micrarmalia* Casey, 1907: 516. Type species: *Emmenastus constrictus* Champion, 1892, monotypy.

***Micrarmalia constricta* (Champion, 1892)** MEX (GE MO)

*Emmenastus constrictus* Champion, 1892: 482.

**Genus *Micromes* Casey, 1907** [M]

*Micromes* Casey, 1907: 432, 441. Type species: *Stibia ovipennis* Horn, 1874, original designation.

***Micromes maritimus* (Casey, 1892)** USA (CA)

*Stibia maritima* Casey, 1891: 52.

***Micromes ovipennis* (Horn, 1874)** USA (CA)

*Stibia ovipennis* Horn, 1874a: 28.

**Genus *Orthostibia* Blaisdell, 1923** [F]

*Orthostibia* Blaisdell, 1923: 235. Type species: *Orthostibia frontalis* Blaisdell, 1923, original designation.

***Orthostibia fraterna* Blaisdell, 1943** MEX (BS)

*Orthostibia fraterna* Blaisdell, 1943: 211.

***Orthostibia frontalis* Blaisdell, 1923** MEX (BS)

*Orthostibia frontalis* Blaisdell, 1923: 236.

***Orthostibia muricata* Blaisdell, 1943** MEX (BS)

*Orthostibia muricata* Blaisdell, 1943: 210.

**Genus *Oxygonodera* Casey, 1907** [F]

*Oxygonodera* Casey, 1907: 433, 444. Type species: *Oxygonodera villosa* Casey, 1907, original designation.

***Oxygonodera grandiceps* Casey, 1907** USA (UT)

*Oxygonodera grandiceps* Casey, 1907: 446.

***Oxygonodera hispidula* (Horn, 1874)** USA (ID OR UT WA)

*Stibia hispidula* Horn, 1874a: 29.

***Oxygonodera villosa* Casey, 1907** USA (UT)

*Oxygonodera villosa* Casey, 1907: 445.

**Genus *Pescennius* Champion, 1884** [M]

*Pescennius* Champion, 1884: 3. Type species: *Pescennius villosus* Champion, 1884, monotypy.

***Pescennius villosus* Champion, 1884** MEX (PU)

*Pescennius villosus* Champion, 1884: 4.

**Genus *Pimeliopsis* Champion, 1892** [F]

*Pimeliopsis* Champion, 1892: 477. Type species: *Pimeliopsis granulata* Champion, 1892, monotypy.

***Pimeliopsis granulata* Champion, 1892** MEX (GE)

*Pimeliopsis granulata* Champion, 1892: 477.

**Genus *Posides* Champion, 1884** [M]

*Posides* Champion, 1884: 6. Type species: *Posides dissidens* Champion, 1884, monotypy.

***Posides dissidens* Champion, 1884** MEX (PU)

*Posides dissidens* Champion, 1884: 6.

**Genus *Soemias* Champion, 1884** [F]

*Soemias* Champion, 1884: 4. Type species: *Soemias minuta* Champion, 1884, monotypy.

***Soemias minuta* Champion, 1884** MEX (VE)

*Soemias minuta* Champion, 1884: 5.

**Genus *Steriphanides* Casey, 1907** [M]

*Steriphanides* Casey, 1907: 515. Type species: *Emmenastus stolidus* Champion, 1892, monotypy.

***Steriphanides stolidus* (Champion, 1892)** MEX (OA)

*Emmenastus stolidus* Champion, 1892: 483.

**Genus *Steriphanus* Casey, 1907** [M]

*Steriphanus* Casey, 1907: 289. Type species: *Emmenastus conicicollis* Casey, 1890, original designation.

***Steriphanus aridus* Casey, 1907** USA (AZ)

*Steriphanus aridus* Casey, 1907: 347.

***Steriphanus conicicollis* (Casey, 1890)** USA (AZ)

*Emmenastus conicicollis* Casey, 1890b: 355.

***Steriphanus convexus convexus* (LeConte, 1866)** USA (AZ NM TX)

*Emmenastus convexus* LeConte, 1866b: 107.

***Steriphanus convexus unicolor* Casey, 1907** USA (NM)

*Steriphanus unicolor* Casey, 1907: 346.

***Steriphanus curtus* (Champion, 1884)** MEX (DU PU)

*Emmenastus curtus* Champion, 1884: 16.

***Steriphanus discrepans* Casey, 1907** USA (AZ)

*Steriphanus discrepans* Casey, 1907: 343.

***Steriphanus discretus* (Casey, 1890)** USA (AZ)

*Emmenastus discretus* Casey, 1890b: 354.

***Steriphanus durus* Blaisdell, 1923** MEX (BC)

*Steriphanus durus* Blaisdell, 1923: 224.

***Steriphanus ellipticus* (Champion, 1884)** MEX (“Pensacola”)

*Emmenastus ellipticus* Champion, 1884: 8.

***Steriphanus estebani* Blaisdell, 1923** MEX (SO)

*Steriphanus estebani* Blaisdell, 1923: 225.

***Steriphanus glabratus* (Champion, 1884)** MEX (JA OA PU)

*Emmenastus glabratus* Champion, 1884: 16.

***Steriphanus hilaris* Casey, 1907** USA (AZ UT)

*Steriphanus hilaris* Casey, 1907: 345.

***Steriphanus lentus* (Champion, 1884)** MEX (CH CO DU)

*Emmenastus lentus* Champion, 1884: 16.

***Steriphanus libertus* Casey, 1907** USA (AZ)

*Steriphanus libertus* Casey, 1907: 350.

***Steriphanus lubricans* Casey, 1907** USA (AZ NV)

*Steriphanus lubricans* Casey, 1907: 345.

***Steriphanus lustrans* Casey, 1907** USA (AZ)

*Steriphanus lustrans* Casey, 1907: 344.

***Steriphanus mancus* (Champion, 1884)** MEX (GE PU)

*Emmenastus mancus* Champion, 1884: 15.

***Steriphanus nigrans* Casey, 1907** USA (AZ)

*Steriphanus nigrans* Casey, 1907: 347.

***Steriphanus nitescens* Casey, 1907** USA (TX)

*Steriphanus nitescens* Casey, 1907: 344.

***Steriphanus perovatus* Casey, 1907** USA (TX)

*Steriphanus perovatus* Casey, 1907: 351.

***Steriphanus picipes* (Champion, 1884)** MEX (OA)

*Emmenastus picipes* Champion, 1884: 17.

***Steriphanus placidus* Casey, 1907** MEX (FD)

*Steriphanus placidus* Casey, 1907: 347.

***Steriphanus proprius* Casey, 1907** USA (AZ)

*Steriphanus proprius* Casey, 1907: 347.

***Steriphanus pulvinatus* (Champion, 1884)** MEX (FD HI OA)

*Emmenastus pulvinatus* Champion, 1884: 17.

***Steriphanus rugicollis* (Champion, 1884)** MEX (SL)

*Emmenastus rugicollis* Champion, 1884: 17.

***Steriphanus rutilans* Casey, 1907** USA (TX)

*Steriphanus rutilans* Casey, 1907: 346.

***Steriphanus subopacus alutaceus* Casey, 1907** USA (AZ) MEX (BC SO)

*Steriphanus alutaceus* Casey, 1907: 347.

***Steriphanus subopacus peropacus* Casey, 1907** USA (AZ)

*Steriphanus peropacus* Casey, 1907: 349.

***Steriphanus subopacus subopacus* (Horn, 1870)** USA (AZ TX) MEX (BC BS SO)

*Emmenastus subopacus* Horn, 1870: 269.

*Steriphanus torpidus* Blaisdell, 1923: 221. Synonymy: Sánchez Piñero and Aalbu (2002: 132).

*Steriphanus mucronatus* Blaisdell, 1923: 223. Synonymy: Sánchez Piñero and Aalbu (2002: 132).

***Steriphanus tardus* Blaisdell, 1923** MEX (SO)

*Steriphanus tardus* Blaisdell, 1923: 222.

**Genus *Stibia* Horn, 1870** [F]

*Stibia* Horn, 1870: 260. Type species: *Stibia puncticollis* Horn, 1870, monotypy.

*Eutriorophus* Casey, 1924: 296. Type species: *Eutriorophus tuckeri* Casey, 1924, original designation. Synonymy: Blaisdell (1933b: 210).

***Stibia blairi* Blaisdell, 1936** USA (AZ CA) MEX (BC)

*Stibia blairi* Blaisdell, 1936a: 88.

***Stibia cribrata* Blaisdell, 1923** MEX (BS)

*Stibia cribrata* Blaisdell, 1923: 239^21^.

***Stibia fallaciosa fallaciosa* Blaisdell, 1936** MEX (BS)

*Stibia fallaciosa* Blaisdell, 1936a: 70.

***Stibia fallaciosa interstitialis* Blaisdell, 1936** MEX (BS)

*Stibia fallaciosa interstitialis* Blaisdell, 1936a: 73.

***Stibia ferruginea* Blaisdell, 1943** MEX (BS)

*Stibia ferruginea* Blaisdell, 1943: 208.

***Stibia freyi* Kulzer, 1959** MEX (SI)

*Stibia freyi* Kulzer, 1959: 614.

***Stibia granulata* Blaisdell, 1923** MEX (BS)

*Stibia granulata* Blaisdell, 1923: 238.

***Stibia imperialis* Blaisdell, 1936** USA (AZ CA)

*Stibia imperialis* Blaisdell, 1936a: 94.

***Stibia puncticollis martinensis* Blaisdell, 1936** MEX (BC)

*Stibia puncticollis martinensis* Blaisdell, 1936a: 83.

***Stibia puncticollis puncticollis* Horn, 1870** USA (CA) MEX (BC BS SO)

*Stibia puncticollis* Horn, 1870: 260.

*Stibia hannai* Blaisdell, 1925b: 329. Synonymy: Blaisdell (1936a: 81).

***Stibia sparsa* Blaisdell, 1923** MEX (BC BS)

*Stibia sparsa* Blaisdell, 1923: 237.

*Stibia tortugensis* Blaisdell, 1936a: 100. Synonymy: Sánchez Piñero and Aalbu (2002: 132).

***Stibia tanneri* Blaisdell, 1936** USA (CA)

*Stibia tanneri* Blaisdell, 1936a: 97.

***Stibia tuckeri* (Casey, 1924)** USA (AZ)

*Eutriorophus tuckeri* Casey, 1924: 297.

***Stibia williamsi* Blaisdell, 1925** MEX (BC)

*Stibia williamsi* Blaisdell, 1925b: 328.

**Genus *Stictodera* Casey, 1907** [F]

*Stictodera* Casey, 1907: 289, 352. Type species: *Emmenastus pinguis* LeConte, 1866, original designation.

***Stictodera pinguis* (LeConte, 1866)** MEX (BS)

*Emmenastus pinguis* LeConte, 1866b: 107.

**Genus *Telabis* Casey, 1890** [M]^22^

*Telabis* Casey, 1890b: 331. Type species: *Eurymetopon longipenne* Casey, 1890, subsequent designation (Casey 1907: 288).

***Telabis alienus* Casey, 1907** USA (AZ)

*Telabis aliena* Casey, 1907: 325.

***Telabis amicus* Casey, 1907** USA (UT)

*Telabis amica* Casey, 1907: 318.

***Telabis asperus* Casey, 1907** USA (CO)

*Telabis aspera* Casey, 1907: 322.

***Telabis blandus* Casey, 1907** USA (TX)

*Telabis blanda* Casey, 1907: 326.

***Telabis brevicollis* (Champion, 1884)** MEX (CO)

*Eurymetopon brevicolle* Champion, 1884: 7.

***Telabis compar* Casey, 1907** USA (AZ)

*Telabis compar* Casey, 1907: 321.

***Telabis crassulus* (Casey, 1890)** USA (AZ TX)

*Eurymetopon crassulum* Casey, 1890b: 344.

***Telabis curticollis* Casey, 1907** USA (AZ)

*Telabis curticollis* Casey, 1907: 321.

***Telabis debilis* (Casey, 1890)** USA (AZ)

*Eurymetopon debile* Casey, 1890b: 343.

***Telabis discors* (Casey, 1890)** USA (TX)

*Eurymetopon discors* Casey, 1890b: 342.

***Telabis famelicus* Casey, 1907** USA (NM)

*Telabis famelica* Casey, 1907: 323.

***Telabis fidelis* Casey, 1907** USA (CA)

*Telabis fidelis* Casey, 1907: 320.

***Telabis hirtipes* Blaisdell, 1923** MEX (BC BS)

*Telabis hirtipes* Blaisdell, 1923: 205.

***Telabis histricus* (Casey, 1890)** USA (AZ)

*Eurymetopon histricum* Casey, 1890b: 340.

***Telabis incisus* Casey, 1907** USA (CA)

*Telabis incisa* Casey, 1907: 322.

***Telabis inops* Casey, 1907** USA (AZ)

*Telabis inops* Casey, 1907: 325.

***Telabis latipennis* Blaisdell, 1923** MEX (BS)

*Telabis latipennis* Blaisdell, 1923: 207.

***Telabis lobifrons* Casey, 1907** USA (AZ)

*Telabis lobifrons* Casey, 1907: 318.

***Telabis longipennis* (Casey, 1890)** USA (NM)

*Eurymetopon longipenne* Casey, 1890b: 339.

***Telabis lunulatus* Blaisdell, 1923** MEX (BC BS)

*Telabis lunulata* Blaisdell, 1923: 206.

***Telabis lustrellus* Casey, 1907** USA (NM)

*Telabis lustrella* Casey, 1907: 323.

***Telabis mimeticus* Casey, 1907** USA (TX)

*Telabis mimetica* Casey, 1907: 319.

***Telabis muricatulus* (Casey, 1890)** USA (AZ TX)

*Eurymetopon muricatulum* Casey, 1890b: 341.

***Telabis nevadensis* Blaisdell, 1925** USA (NV)

*Telabis nevadensis* Blaisdell, 1925c: 372.

***Telabis obtusus* Casey, 1907** USA (AZ)

*Telabis obtusa* Casey, 1907: 317.

***Telabis opacellus* Casey, 1907** USA (CA)

*Telabis opacella* Casey, 1907: 316.

***Telabis ovalis* Casey, 1907** USA (AZ)

*Telabis ovalis* Casey, 1907: 324.

***Telabis pavidus* Casey, 1907** USA (NM)

*Telabis pavida* Casey, 1907: 324.

***Telabis prominens* Casey, 1907** USA (TX)

*Telabis prominens* Casey, 1907: 314.

*Telabis proxima* Casey, 1907: 315. Synonymy: Casey (1911: 253).

***Telabis punctulatus* (LeConte, 1866)** MEX (BC BS SO)

*Eurymetopon punctulatum* LeConte, 1866b: 105.

***Telabis rubidus* Casey, 1907** USA (TX)

*Telabis rubida* Casey, 1907: 315.

***Telabis serratus* (LeConte, 1866)** USA (AZ CA ID NM NV OR TX) MEX (BC BS SO)

*Eurymetopon serratum* LeConte, 1866b: 106.

***Telabis sodalis* (Horn, 1870)** USA (AZ CA) MEX (BS)

*Eurymetopon sodalis* Horn, 1870: 268.

***Telabis timidus* Casey, 1907** USA (AZ)

*Telabis timida* Casey, 1907: 320.

***Telabis uteanus* Casey, 1907** USA (UT)

*Telabis uteana* Casey, 1907: 317.

***Telabis vafer* Casey, 1907** USA (AZ)

*Telabis vafra* Casey, 1907: 315.

***Telabis vapidus* Casey, 1907** USA (TX)

*Telabis vapida* Casey, 1907: 322.

**Genus *Telaponium* Blaisdell, 1923** [N]

*Telaponium* Blaisdell, 1923: 209. Type species: *Telaponium castaneum* Blaisdell, 1923, original designation.

***Telaponium castaneum* Blaisdell, 1923** MEX (BS)

*Telaponium castaneum* Blaisdell, 1923: 209.

***Telaponium pingue* Blaisdell, 1943** MEX (BS)

*Telaponium pingue* Blaisdell, 1943: 179.

**Genus *Texaponium* Thomas, 1984** [N]

*Texaponium* Thomas, 1984: 658. Type species: *Cryptadius triplehorni* Berry, 1974, original designation.

***Texaponium triplehorni* (Berry, 1974)** USA (TX)

*Cryptadius triplehorni* Berry, 1974: 172.

**Genus *Tlascalinus* Casey, 1907** [M]

*Tlascalinus* Casey, 1907: 370. Type species: *Trimytis flohri* Champion, 1892, monotypy.

***Tlascalinus flohri* (Champion, 1892)** MEX (FD)

*Trimytis flohri* Champion, 1892: 478.

**Genus *Trichiotes* Casey, 1907** [M]

*Trichiotes* Casey, 1907: 432, 443. Type species: *Trichiotes seriatus* Casey, 1907, original designation.

***Trichiotes lightfooti* Wirth and Smith, 2017** MEX (CO)

*Trichiotes lightfooti* Wirth and Smith, 2017: 535.

***Trichiotes seriatus* Casey, 1907** USA (NM TX) MEX (CO NL)

*Trichiotes seriatus* Casey, 1907: 444.

**Genus *Trientoma* Solier, 1835** [F]

*Trientoma* Solier, 1835b: 256. Type species: *Trientoma varvasi* Solier, 1835, monotypy.

***Trientoma cayensis* Garrido and Gutiérrez, 1995** CUB

*Trientoma cayensis* Garrido and Gutiérrez, 1995b: 48.

***Trientoma convexipennis* Allard, 1883** CUB

*Trientoma convexipennis* Allard, 1883: 14.

***Trientoma garridoi* Marcuzzi, 1988** CUB

*Trientoma garridoi* Marcuzzi, 1988: 69.

***Trientoma guadeloupensis* Fleutiaux and Sallé, 1890** LAN

*Trientoma guadeloupensis* Fleutiaux and Sallé, 1890: 421.

***Trientoma jilae* Steiner, 2006** BAH

*Trientoma jilae* Steiner, 2006: 3.

***Trientoma kaszabi* Marcuzzi, 1985** CUB

*Trientoma kaszabi* Marcuzzi, 1985: 181.

***Trientoma kochi* Marcuzzi, 1977** CAY

*Trientoma kochi* Marcuzzi, 1977: 6.

***Trientoma laevis* Allard, 1883** HAI

*Trientoma laevis* Allard, 1883: 14.

***Trientoma maisiensis* Marcuzzi, 1988** CUB

*Trientoma maisiensis* Marcuzzi, 1988: 67.

*Trientoma zayasi* Marcuzzi, 1988: 70. Synonymy: Garrido and Gutiérrez (1995b: 48).

***Trientoma martinicensis* Allard, 1883** LAN (Martinique)

*Trientoma martinicensis* Allard, 1883: 14.

***Trientoma puertoricensis* Marcuzzi, 1977** PRI

*Trientoma puertoricensis* Marcuzzi, 1977: 7.

***Trientoma rugifrons* Champion, 1884** MEX / HIS

*Trientoma rugifrons* Champion, 1884: 2.

***Trientoma ryticephala* Allard, 1883** HAI

*Trientoma ryticephala* Allard, 1883: 14.

***Trientoma sallei* Kraatz, 1865** MEX / HAI DOM

*Trientoma sallei* Kraatz, 1865: 74.

*Trientoma mexicana* Champion, 1884: 2. Synonymy: Champion (1892: 479).

***Trientoma siboneyensis* Marcuzzi, 1988** CUB

*Trientoma siboneyensis* Marcuzzi, 1988: 71.

***Trientoma varvasi* Solier, 1835** CUB

*Trientoma varvasi* Solier, 1835b: 257.

***Trientoma voegeliorum* Steiner, 2006** BAH

*Trientoma voegeliorum* Steiner, 2006: 8.

***Trientoma wickhami* Casey, 1907** BAH

*Trientoma wickhami* Casey, 1907: 377.

**Genus *Trimytantron* Ardoin, 1977** [N]

*Trimytantron* Ardoin, 1977b: 381. Type species: *Trimytantron decui* Ardoin, 1977, original designation.

*Bielawskia* Marcuzzi, 1985: 179. Type species: *Bielawskia cubana* Marcuzzi, 1985 (= *Trimytantron decui* Ardoin, 1977), monotypy. Synonymy: Marcuzzi (1998a: 153).

***Trimytantron armasi* Garrido and Gutiérrez, 1997** CUB

*Trimytantron armasi* Garrido and Gutiérrez, 1997: 32.

***Trimytantron cavernicolous* Garrido and Gutiérrez, 1997** CUB

*Trimytantron cavernicolous* Garrido and Gutiérrez, 1997: 34.

***Trimytantron cubanum* Ardoin, 1977** CUB

*Trimytantron cubanum* Ardoin, 1977c: 388.

***Trimytantron decui* Ardoin, 1977** CUB

*Trimytantron decui* Ardoin, 1977b: 382.

*Bielawskia cubana* Marcuzzi, 1985: 179 [junior secondary homonym of *Trimytantron cubanum* Ardoin, 1977]. Synonymy: Garrido and Gutiérrez (1997: 30).

*Trimytantron garridoi* Marcuzzi, 1998a: 153. Replacement name for *Trimytantron cubanum* (Marcuzzi, 1985).

***Trimytantron escambrayense* Garrido and Gutiérrez, 1997** CUB

*Trimytantron escambrayensis* Garrido and Gutiérrez, 1997: 33.

***Trimytantron litorale* Garrido and Gutiérrez, 1997** CUB

*Trimytantron litoralis* Garrido and Gutiérrez, 1997: 30.

***Trimytantron minus*** ^23^ **Garrido and Gutiérrez, 1997** CUB

*Trimytantron minor* Garrido and Gutiérrez, 1997: 36.

***Trimytantron negreai* Ardoin, 1977** CUB

*Trimytantron negreai* Ardoin, 1977c: 387.

***Trimytantron poeyi* Ardoin, 1977** CUB

*Trimytantron poeyi* Ardoin, 1977b: 383.

***Trimytantron pumilum* Garrido and Gutiérrez, 1997** CUB

*Trimytantron pumilus* Garrido and Gutiérrez, 1997: 35.

***Trimytantron punctulaticeps* Garrido and Gutiérrez, 1997** CUB

*Trimytantron punctulaticeps* Garrido and Gutiérrez, 1997: 34.

***Trimytantron sierrae* Garrido and Gutiérrez, 1997** CUB

*Trimytantron sierrae* Garrido and Gutiérrez, 1997: 31.

***Trimytantron vinai* Ardoin, 1977** CUB

*Trimytantron viñai* Ardoin, 1977c: 388.

**Genus *Trimytis* LeConte, 1851** [F]

*Trimytis* LeConte, 1851: 141. Type species: *Trimytis pruinosa* LeConte, 1851, monotypy.

*Pimalius* Casey, 1907: 367. Type species: *Trimytis pulverea* Horn, 1870, original designation. Synonymy: MacLachlan and Olson (1990: 79).

***Trimytis ceralboensis* Blaisdell, 1943** MEX (BS)

*Trimytis ceralboensis* Blaisdell, 1943: 196.

***Trimytis obovata* Champion, 1892: 478** MEX (CH)

*Trimytis obovata* Champion, 1892: 478.

***Trimytis obtusa* Horn, 1894** MEX (BS)

*Trimytis obtusa* Horn, 1894b: 412.

***Trimytis pruinosa* LeConte, 1851** USA (AZ CO KS MT NE NM SD TX WY)

*Trimytis pruinosa* LeConte, 1851: 141.

*Trimytis nympha* Casey, 1907: 368. Synonymy: MacLachlan and Olson (1990: 80).

*Trimytis tonsa* Casey, 1907: 369. Synonymy: MacLachlan and Olson (1990: 80).

*Trimytis ignava* Casey, 1907: 369. Synonymy: MacLachlan and Olson (1990: 81).

*Trimytis trapezifera* Casey, 1924: 299. Synonymy: MacLachlan and Olson (1990: 81).

***Trimytis pulverea* Horn, 1870** USA (AZ)

*Trimytis pulverea* Horn, 1870: 261.

***Trimytis subsenilis* Blaisdell, 1923** MEX (SO)

*Trimytis subsenilis* Blaisdell, 1923: 227.

**Genus *Triorophus* LeConte, 1851** [M]

*Triorophus* LeConte, 1851: 141. Type species: *Triorophus laevis* LeConte, 1851, subsequent designation (Casey 1907: 432).

***Triorophus basalis* Casey, 1907** USA (AZ)

*Triorophus basalis* Casey, 1907: 437.

***Triorophus brevis* Casey, 1907** USA (TX)

*Triorophus brevis* Casey, 1907: 439.

***Triorophus gracilicornis* Casey, 1907** USA (CA)

*Triorophus gracilicornis* Casey, 1907: 437.

***Triorophus gravidulus* Casey, 1907** USA (AZ)

*Triorophus gravidulus* Casey, 1907: 437.

***Triorophus histrio* Casey, 1907** USA (AZ)

*Triorophus histrio* Casey, 1907: 437.

***Triorophus laevis laevis* LeConte, 1851** USA (AZ CA NV) MEX (SO)

*Triorophus laevis* LeConte, 1851: 141.

***Triorophus laevis politus* Casey, 1907** USA (CA NV)

*Triorophus politus* Casey, 1907: 435.

***Triorophus lariversi* Blaisdell, 1942** USA (NV)

*Triorophus lariversi* Blaisdell, 1942: 132.

***Triorophus laticeps* Casey, 1924** USA (TX)

*Triorophus laticeps* Casey, 1924: 297.

***Triorophus lecontei* Casey, 1890** USA (TX) MEX (CH DU)

*Triorophus lecontei* Casey, 1890b: 327.

***Triorophus longicornis* Casey, 1907** USA (AZ)

*Triorophus longicornis* Casey, 1907: 438.

***Triorophus mixtus* Casey, 1907** USA (TX)

*Triorophus mixtus* Casey, 1907: 440.

***Triorophus mundulus* Casey, 1907** USA (AZ)

*Triorophus mundulus* Casey, 1907: 436.

***Triorophus nevadensis* Casey, 1924** USA (NV)

*Triorophus nevadensis* Casey, 1924: 298.

***Triorophus nodiceps* LeConte, 1853** USA (TX) MEX (CO)

*Triorophus nodiceps* LeConte, 1853: 446.

***Triorophus puberulus* Casey, 1924** USA (CA)

*Triorophus puberulus* Casey, 1924: 298.

***Triorophus punctatus* LeConte, 1851** USA (CA)

*Triorophus punctatus* LeConte, 1851: 142.

***Triorophus rugiceps* LeConte, 1851** USA (CA ID)

*Triorophus rugiceps* LeConte, 1851: 142.^24^

***Triorophus simplex* Casey, 1907** USA (AZ)

*Triorophus simplex* Casey, 1907: 436.

***Triorophus subpubescens* Horn, 1870** USA (CA)

*Triorophus subpubescens* Horn, 1870: 259.

***Triorophus terebratulus* Casey, 1907** USA (AZ)

*Triorophus terebratulus* Casey, 1907: 436.

***Triorophus thoracicus* Casey, 1924** USA (AZ)

*Triorophus thoracicus* Casey, 1924: 298.

**Genus *Triphalopsis* Blaisdell, 1923** [F]

*Triphalopsis* Blaisdell, 1923: 232. Type species: *Triphalopsis partida* Blaisdell, 1923, original designation.

***Triphalopsis californica* Doyen, 1983** USA (CA) MEX (BC)

*Triphalopsis californicus* Doyen, 1983: 87.

***Triphalopsis impressicollis* Blaisdell, 1943** MEX (BC)

*Triphalopsis impressicollis* Blaisdell, 1943: 203.

***Triphalopsis partida* Blaisdell, 1923** MEX (BC BS SO)

*Triphalopsis partida* Blaisdell, 1923: 232.

*Triphalopsis minor* Blaisdell, 1923: 233. Synonymy: Sánchez Piñero and Aalbu (2002: 132).

**Genus *Triphalopsoides* Doyen, 1990** [M]

*Triphalopsoides* Doyen, 1990: 222. Type species: *Triphalopsoides lasiodorsa* Doyen, 1990, monotypy.

***Triphalopsoides lasiodorsa* Doyen, 1990** MEX (JA)

*Triphalopsoides lasiodorsa* Doyen, 1990: 224.

**Genus *Triphalus* LeConte, 1866** [M]

*Triphalus* LeConte, 1866b: 104. Type species: *Triphalus perforatus* LeConte, 1866, monotypy.

***Triphalus cribricollis* Horn, 1895** MEX (BS)

*Triphalus cribricollis* Horn, 1895: 251.

***Triphalus impressifrons* Blaisdell, 1943** MEX (BS)

*Triphalus impressifrons* Blaisdell, 1943: 202.

***Triphalus perforatus* LeConte, 1866** MEX (BS)

*Triphalus perforatus* LeConte, 1866b: 104.

***Triphalus subcylindricus* Blaisdell, 1923** MEX (BS)

*Triphalus subcylindricus* Blaisdell, 1923: 234.

**Genus *Troglogeneion* Aalbu, 1985** [N]

*Troglogeneion* Aalbu, 1985: 541. Type species: *Troglogeneion zapoteca* Aalbu, 1985, monotypy.

***Troglogeneion zapoteca* Aalbu, 1985** MEX (OA)

*Troglogeneion zapoteca* Aalbu, 1985: 542.

**Tribe Epitragini Blanchard, 1845**

Lygophila Rafinesque, 1815: 113 [*nomen oblitum*, see Bouchard et al. (2007: 386)]. Type genus: *Lygophilus* Rafinesque, 1815 (= *Epitragus* Latreille, 1802).

Épitragites Blanchard, 1845: 16 [*nomen protectum*]. Type genus: *Epitragus* Latreille, 1802.

**Genus *Bothrotes* Casey, 1907** [M]

*Bothrotes* Casey, 1907: 379, 398. Type species: *Epitragus canaliculatus* Say, 1824, original designation.

***Bothrotes angusticollis* (Champion, 1884)** MEX (GE JA SI)

*Epitragus angusticollis* Champion, 1884: 26.

***Bothrotes bicarinatus* (Champion, 1884)** MEX (CL VE)

*Epitragus bicarinatus* Champion, 1884: 25.

***Bothrotes canaliculatus acutus* (LeConte, 1866)** USA (FL KS NM OK TX) MEX (CO)

*Epitragus acutus* LeConte, 1866b: 108.

*Bothrotes fortis* Casey, 1907: 399. Synonymy: Freude (1967: 283).

*Bothrotes subrudis* Casey, 1907: 400. Synonymy: Freude (1967: 283).

*Bothrotes pensus* Casey, 1907: 400. Synonymy: Freude (1967: 283).

*Bothrotes knausi* Casey, 1907: 401. Synonymy: Freude (1967: 283).

***Bothrotes canaliculatus arundinis* (LeConte, 1866)** USA (DE GA MD NC NJ NY SC VA)

*Epitragus arundinis* LeConte, 1866b: 108.

*Bothrotes pinorum* Casey, 1924: 304. Synonymy: Freude (1967: 281).

***Bothrotes canaliculatus canaliculatus* (Say, 1824)** USA (AZ CO IL KS MO NM OH SD TX WI) MEX (CH DU SO ZA)

*Epitragus canaliculatus* Say, 1824b: 281.

***Bothrotes canaliculatus mexicanus* Freude, 1967** MEX (DU NL TA)

*Bothrotes canaliculatus mexicanus* Freude, 1967: 285.

***Bothrotes canus* (Champion, 1884)** MEX (GE)

*Epitragus canus* Champion, 1884: 34.

***Bothrotes cristatus* (Champion, 1892)** MEX (CO GE)

*Epitragus cristatus* Champion, 1892: 485.

***Bothrotes foveatus* (Champion, 1884)** MEX (OA VE)

*Epitragus foveatus* Champion, 1884: 29.

***Bothrotes hoegei* (Champion, 1884)** MEX (MI VE)

*Epitragus högei* Champion, 1884: 26.

***Bothrotes inaequalis* (Champion, 1884)** MEX (OA PU VE)

*Epitragus inaequalis* Champion, 1884: 32.

***Bothrotes incisus* (Champion, 1884)** MEX (NA [Islas Marías])

*Epitragus incisus* Champion, 1884: 28.

***Bothrotes littoralis* (Champion, 1884)** MEX (GE JA MO NA OA SI)

*Epitragus littoralis* Champion, 1884: 27.

***Bothrotes ornatus* (Champion, 1884)** MEX (DU GU PU VE)

*Epitragus ornatus* Champion, 1884: 26.

***Bothrotes plumbeus plumbeus* (LeConte, 1866)** USA (AZ CA CO KS NE NM SD TX)

*Epitragus plumbeus* LeConte, 1866b: 109.

*Bothrotes aeneicollis* Casey, 1907: 401. Synonymy: Freude (1967: 287).

*Bothrotes chalceus* Casey, 1907: 402. Synonymy: Freude (1967: 287).

*Bothrotes affinis* Casey, 1907: 405. Synonymy: Freude (1967: 287).

*Bothrotes pertinax* Casey, 1907: 405. Synonymy: Freude (1967: 287).

*Bothrotes picipennis* Casey, 1907: 406. Synonymy: Freude (1967: 287).

*Bothrotes secutor* Casey, 1907: 406. Synonymy: Freude (1967: 287).

Bothrotes secutor var. apertus Casey, 1907: 406. Synonymy: Freude (1967: 287).

*Bothrotes acomanus* Casey, 1907: 407. Synonymy: Freude (1967: 287).

*Bothrotes neglectus* Casey, 1907: 407. Synonymy: Freude (1967: 287).

*Bothrotes insitus* Casey, 1907: 408. Synonymy: Freude (1967: 287).

*Bothrotes funebris* Casey, 1907: 409. Synonymy: Freude (1967: 287).

***Bothrotes plumbeus rorulentus* (Champion, 1884)** MEX (CO GU SI)

*Epitragus rorulentus* Champion, 1884: 27.

***Bothrotes plumbeus tenebrosus* Casey, 1907** USA (AZ) MEX (SO)

*Bothrotes tenebrosus* Casey, 1907: 403.

*Bothrotes occipitalis* Casey, 1907: 403. Synonymy: Freude (1967: 296).

*Bothrotes confertus* Casey, 1907: 404. Synonymy: Freude (1967: 296).

*Bothrotes eversus* Casey, 1907: 404. Synonymy: Freude (1967: 296).

*Bothrotes perditus* Casey, 1907: 409. Synonymy: Freude (1967: 296).

*Bothrotes amplificans* Casey, 1907: 410. Synonymy: Freude (1967: 296).

*Bothrotes obsolescens* Casey, 1907: 411. Synonymy: Freude (1967: 296).

***Bothrotes scutatus occidentalis* Freude, 1967** MEX (CH CL JA NA OA)

*Bothrotes scutatus occidentalis* Freude, 1967: 304.

***Bothrotes scutatus scutatus* (Champion, 1884)** MEX (GU)

*Epitragus scutatus* Champion, 1884: 28.

**Genus *Conoecus* Horn, 1885** [M]

*Conoecus* Horn, 1885c: 159. Type species: *Conoecus ovipennis* Horn, 1885, monotypy.

***Conoecus ovipennis estriatus* Casey, 1907** USA (LA TX)

*Conoecus estriatus* Casey, 1907: 431.

***Conoecus ovipennis ovipennis* Horn, 1885** USA (TX)

*Conoecus ovipennis* Horn, 1885c: 159.

**Genus *Cyrtomius* Casey, 1907** [M]

*Cyrtomius* Casey, 1907: 379. Type species: *Cyrtomius cavicauda* Casey, 1907(=*Epitragus plicatus* Champion, 1884), original designation.

**Subgenus Cyrtomius Casey, 1907**

*Cyrtomius* Casey, 1907: 379. Type species: *Cyrtomius cavicauda* Casey, 1907(= *Epitragus plicatus* Champion, 1884), original designation.

***Cyrtomius chevrolati* (Champion, 1884)** MEX (DU GU MO PU VE) GUA NIC

*Epitragus chevrolati* Champion, 1884: 30.

***Cyrtomius freyi* Freude, 1967** MEX (GE JA MI PU)

*Cyrtomius freyi* Freude, 1967: 229.

***Cyrtomius plicatus* (Champion, 1884)** MEX (FD OA VE)

*Epitragus plicatus* Champion, 1884: 31.

*Cyrtomius cavicauda* Casey, 1907: 384. Synonymy: Freude (1967: 231).

**Subgenus Grandicyrtomius Freude, 1967**

*Grandicyrtomius* Freude, 1967: 225. Type species: *Epitragus grandis* Champion, 1884, original designation.

***Cyrtomius grandis* (Champion, 1884)** MEX (CI DU GE JA MO OA PU SI SO VE)

*Epitragus grandis* Champion, 1884: 31.

[incertae sedis]

***Cyrtomius gaigli* Freude, 1986** GUA

*Cyrtomius gaigli* Freude, 1986: 27.

***Cyrtomius polli* Freude, 1986** GUA

*Cyrtomius polli* Freude, 1986: 26.

**Genus *Epitragodes* Casey, 1890** [M]

*Epitragodes* Casey, 1890b: 365. Type species: *Epitragus tomentosus* LeConte, 1866, monotypy.

***Epitragodes tomentosus macilentus* Casey, 1907** USA (AL FL GA NC SC VA) / BAH

*Epitragodes tomentosus macilentus* Casey, 1907: 425.

*Epitragodes debilicollis* Casey, 1907: 423. Synonymy: Freude (1968: 86).

*Epitragodes pardalis* Casey, 1907: 423. Synonymy: Freude (1968: 86).

*Epitragodes cuprascens* Casey, 1907: 424. Synonymy: Freude (1968: 86).

***Epitragodes tomentosus tomentosus* (LeConte, 1866)** USA (FL GA) / BAH

*Epitragus tomentosus* LeConte, 1866b: 109.

*Epitragodes floridanus* Casey, 1907: 424. Synonymy: Freude (1968: 86).

*Epitragodes obesulus* Casey, 1907: 425. Synonymy: Freude (1968: 86).

**Genus *Epitragopsis* Casey, 1907** [F]

*Epitragopsis* Casey, 1907: 386. Type species: *Epitragus godmani* Champion, 1884, original designation.

***Epitragopsis communis* (Champion, 1884)** MEX (OA VE) GUA BEL HON

*Epitragus communis* Champion, 1884: 36.

***Epitragopsis godmani* (Champion, 1884)** PAN / SA

*Epitragus godmani* Champion, 1884: 36.

*Epitragopsis auratus* Marcuzzi, 1961: 8. Synonymy: Freude (1968: 71).

***Epitragopsis ruatanensis* (Champion, 1892)** HON NIC

*Epitragus ruatanensis* Champion, 1892: 488.

**Genus *Epitragosoma* Brown and Triplehorn, 2002** [N]

*Epitragosoma* Brown and Triplehorn, 2002: 515. Type species: *Epitragosoma arenaria* Brown and Triplehorn, 2002, original designation.

***Epitragosoma arenarium* Brown and Triplehorn, 2002** USA (NM TX)

*Epitragosoma arenaria* Brown and Triplehorn, 2002: 519.

**Genus *Epitragus* Latreille, 1802** [M]

*Epitragus* Latreille, 1802: 165. Type species: *Epitragus fuscus* Latreille, 1804, subsequent monotypy in Latreille (1804: 322).

**Subgenus Epitragus Latreille, 1802**

*Epitragus* Latreille, 1802: 165. Type species: *Epitragus fuscus* Latreille, 1804, subsequent monotypy in Latreille (1804: 322).

***Epitragus antillensis* Marcuzzi, 1961** JAM

*Epitragus antillensis* Marcuzzi, 1961: 28.

***Epitragus aurulentus aurulentus* Kirsch, 1866** NIC CRI PAN / CUB JAM HIS PRI LAN / SA

*Epitragus aurulentus* Kirsch, 1866: 189.

*Epitragus jamaicensis* Champion, 1896: 3. Synonymy: Freude (1967: 158).

***Epitragus emarginatus* Champion, 1884** PAN

*Epitragus emarginatus* Champion, 1884: 24.

*Epitragus consimilis* Marcuzzi, 1961: 34. Synonymy: Freude (1967: 164).

***Epitragus gaigli* Freude, 1986** GUA

*Epitragus gaigli* Freude, 1986: 25.

***Epitragus mexicanus* Marcuzzi, 1961** MEX (OA)

*Epitragus mexicanus* Marcuzzi, 1961: 29.

***Epitragus nigricans* Champion, 1884** PAN / SA

*Epitragus nigricans* Champion, 1884: 24.

*Epitragus puberulus* Kirsch, 1886: 332. Synonymy: Freude (1967: 162).

***Epitragus roscidus* Erichson, 1849** LAN / SA

*Epitragus roscidus* Erichson, 1849: 565.

*Epitragus exaratus* Champion, 1896: 2. Synonymy: Freude (1967: 166).

***Epitragus sallei* Champion, 1884** ^25^ MEX (CI VE YU) GUA HON NIC CRI / SA

*Epitragus sallaei* Champion, 1884: 24.

*Epitragus rigens* Casey, 1907: 381. Synonymy: Freude (1967: 155).

**Genus *Hemasodes* Casey, 1907** [M]

*Hemasodes* Casey, 1907: 378. Type species: *Schoenicus vestitus* Champion, 1884, original designation.

***Hemasodes vestitus* (Champion, 1884)** MEX (GE JA OA VE)

*Schoenicus vestitus* Champion, 1884: 22.

*Schoenicus yucatanensis* Champion, 1884: 22. Synonymy: Freude (1967: 182).

**Genus *Lobometopon* Casey, 1907** [N]

*Lobometopon* Casey, 1907: 379, 385. Type species: *Epitragus fusiformis* Casey, 1890, original designation.

***Lobometopon acutangulum* (Champion, 1884)** MEX (CI OA) GUA

*Epitragus acutangulus* Champion, 1884: 31.

***Lobometopon aeratum* (Champion, 1884)** MEX (CL GE JA NA OA VE)

*Epitragus aeratus* Champion, 1884: 33.

***Lobometopon aurichalceum* (Champion, 1884)** USA (AZ) MEX (GU OA SI)

*Epitragus aurichalceus* Champion, 1884: 33.

*Lobometopon tuckeri* Casey, 1924: 301. Synonymy: Freude (1968: 44).

***Lobometopon cupreum* (Champion, 1884)** GUA NIC CRI PAN

*Epitragus cupreus* Champion, 1884: 34.

*Lobometopon bicaviceps* Casey, 1907: 394. Synonymy: Freude (1968: 36).

*Lobometopon alveolatum* Casey, 1907: 394. Synonymy: Freude (1968: 36).

***Lobometopon fusiforme cribricolle* Casey, 1907** USA (KS NE NM SD TX) MEX (NL)

*Lobometopon cribricolle* Casey, 1907: 391.

*Lobometopon jucundum* Casey, 1907: 392. Synonymy: Freude (1968: 50).

*Lobometopon obscurum* Casey, 1907: 395. Synonymy: Freude (1968: 50).

***Lobometopon fusiforme fusiforme* (Casey, 1890)** USA (AZ) MEX (SO)

*Epitragus fusiformis* Casey, 1890b: 365.

*Lobometopon symmetricum* Casey, 1907: 389. Synonymy: Freude (1968: 50).

*Lobometopon pimalicum* Casey, 1907: 389. Synonymy: Freude (1968: 50).

*Lobometopon aeneopiceum* Casey, 1907: 390. Synonymy: Freude (1968: 50).

*Lobometopon docile* Casey, 1907: 389. Synonymy: Freude (1968: 50).

*Lobometopon propinquum* Casey, 1907: 391. Synonymy: Freude (1968: 50).

*Lobometopon aequipenne* Casey, 1907: 393. Synonymy: Freude (1968: 50).

*Lobometopon morrisoni* Casey, 1907: 393. Synonymy: Freude (1968: 50).

***Lobometopon fusiforme uintanum* Casey, 1907** USA (AZ NM NV UT)

*Lobometopon uintanum* Casey, 1907: 388.

*Lobometopon parvicolle* Casey, 1907: 392. Synonymy: Freude (1968: 50).

*Lobometopon alticola* Casey, 1924: 302. Synonymy: Freude (1968: 50).

*Lobometopon woodgatei* Casey, 1924: 302. Synonymy: Freude (1968: 50).

*Lobometopon provoanum* Casey, 1924: 303. Synonymy: Freude (1968: 50).

***Lobometopon guatemalense* (Champion, 1884)** GUA BEL SAL HON NIC CRI

*Epitragus guatemalensis* Champion, 1884: 32.

***Lobometopon lucidum* (Champion, 1884)** MEX (DU NA PU SI SO)

*Epitragus lucidus* Champion, 1884: 34.

***Lobometopon metallicum* (Champion, 1884)** MEX (CH CI CL DU FD GE GU ME MO OA PU QU SL VE) GUA CRI

*Epitragus metallicus* Champion, 1884: 29.

*Epitragus gracilis* Casey, 1890b: 366. Synonymy: Freude (1968: 40).

*Lobometopon aberrans* Casey, 1907: 387. Synonymy: Freude (1968: 40).

***Lobometopon micans* (Champion, 1884)** MEX (CI FD OA)

*Epitragus micans* Champion, 1884: 32.

***Lobometopon obovatum* (Champion, 1884)** MEX (GE OA)

*Epitragus obovatus* Champion, 1884: 35.

***Lobometopon ovale* (Casey, 1885)** USA (TX)

*Epitragus ovalis* Casey, 1885: 184.

***Lobometopon parviceps* (Champion, 1884)** MEX (CL OA)

*Epitragus parviceps* Champion, 1884: 34.

**Genus *Metopoloba* Casey, 1907** [F]

*Metopoloba* Casey, 1907: 379. Type species: *Epitragus pruinosus* Horn, 1870, original designation.

***Metopoloba pruinosa mexicana* Freude, 1967** MEX (SO)

*Metopoloba pruinosa mexicana* Freude, 1967: 259.

***Metopoloba pruinosa pruinosa* (Horn, 1870)** USA (AZ CA NV UT)

*Epitragus pruinosus* Horn, 1870: 264.

*Metopoloba bifossiceps* Casey, 1907: 413. Synonymy: Freude (1967: 248).

*Metopoloba proba* Casey, 1907: 414. Synonymy: Freude (1967: 248).

*Metopoloba punctiventris* Casey, 1907: 414. Synonymy: Freude (1967: 248).

*Metopoloba perpolita* Casey, 1907: 415. Synonymy: Freude (1967: 248).

*Metopoloba californica* Casey, 1907: 419. Synonymy: Freude (1967: 248).

*Lobometopon juabense* Casey, 1924: 303. Synonymy: Freude (1967: 248).

***Metopoloba pruinosa subpilosa* Blaisdell, 1943** MEX (BS)

*Metopoloba subpilosa* Blaisdell, 1943: 199.

***Metopoloba pruinosa subseriata* Casey, 1907** USA (AZ NM TX)

*Metopoloba subseriata* Casey, 1907: 415.

*Metopoloba snowi* Casey, 1907: 416. Synonymy: Freude (1967: 253).

*Metopoloba densiventris* Casey, 1907: 417. Synonymy: Freude (1967: 253).

*Metopoloba contaminans* Casey, 1907: 418. Synonymy: Freude (1967: 253).

*Metopoloba amplexa* Casey, 1907: 418. Synonymy: Freude (1967: 253).

*Metopoloba sublaeviceps* Casey, 1907: 418. Synonymy: Freude (1967: 253).

*Metopoloba angulata* Casey, 1907: 419. Synonymy: Freude (1967: 253).

***Metopoloba pruinosa werneri* Freude, 1967** MEX (BS)

*Metopoloba pruinosa werneri* Freude, 1967: 259.

**Genus *Ortheolus* Casey, 1907** [M]

*Ortheolus* Casey, 1907: 380. Type species: *Schoenicus oculatus* Champion, 1884, original designation.

***Ortheolus antillarum* (Champion, 1896)** LAN

*Schoenicus antillarum* Champion, 1896: 5.

*Schoenicus brunneus* Champion, 1896: 4. Synonymy: Freude (1968: 106).

***Ortheolus caraibicus caraibicus* Marcuzzi, 1961** LAN / SA

*Ortheolus caraibicus* Marcuzzi, 1961: 38.

***Ortheolus oculatus oculatus* (Champion, 1884)** PAN

*Schoenicus oculatus* Champion, 1884: 18.

***Ortheolus panamensis* (Champion, 1884)** CRI PAN

*Schoenicus panamensis* Champion, 1884: 18.

**Genus *Pechalius* Casey, 1907** [M]

*Pechalius* Casey, 1907: 379, 420. Type species: *Pechalius subvittatus* Casey, 1907, original designation.

*Epitragoma* Casey, 1907: 386. Type species: *Epitragus vestitus* Casey, 1891, monotypy. Synonymy: Freude (1968: 61).

***Pechalius bradleyi* Triplehorn, 1974** USA (NM)

*Pechalius bradleyi* Triplehorn, 1974: 73.

***Pechalius dentiger* (Horn, 1870)** USA (AZ) MEX (SO)

*Epitragus dentiger* Horn, 1870: 265.

***Pechalius pilosus* (Champion, 1884)** MEX (CH TA VE)

*Epitragus pilosus* Champion, 1884: 34.

***Pechalius subvittatus* Casey, 1907** USA (TX) MEX (DU)

*Pechalius subvittatus* Casey, 1907: 421.

***Pechalius vestitus* (Casey, 1891)** USA (AZ)

*Epitragus vestitus* Casey, 1891: 53.

**Genus *Phegoneus* Casey, 1907** [M]

*Phegoneus* Casey, 1907: 380, 426. Type species: *Epitragodes julichi* Casey, 1891, original designation.

**Subgenus Pectphegoneus Freude, 1968**

*Pectphegoneus* Freude, 1968: 90. Type species: *Schoenicus pectoralis* Champion, 1884, monotypy.

***Phegoneus pectoralis* (Champion, 1884)** MEX (CL GE JA MI MO PU)

*Schoenicus pectoralis* Champion, 1884: 21.

**Subgenus Phegoneus Casey, 1907**

*Phegoneus* Casey, 1907: 380, 426. Type species: *Epitragodes julichi* Casey, 1891, original designation.

***Phegoneus basalis* (Champion, 1884)** MEX (OA VE)

*Schoenicus basalis* Champion, 1884: 21.

***Phegoneus chalybeus* (Champion, 1884)** MEX (MI NA OA PU SI SL VE)

*Schoenicus chalybeus* Champion, 1884: 20.

***Phegoneus difficilis* (Champion, 1884)** MEX (DU GE JA MI OA SI VE)

*Schoenicus difficilis* Champion, 1884: 20.

***Phegoneus julichi* (Casey, 1891)** USA (TX)

*Epitragodes jülichi* Casey, 1891: 55.

***Phegoneus rufipes impressus* (Champion, 1884)** CRI

*Schoenicus impressus* Champion, 1884: 20.

***Phegoneus rufipes rufipes* (Champion, 1884)** MEX (YU) NIC

*Schoenicus rufipes* Champion, 1884: 19.

***Phegoneus salvini salvini* (Champion, 1884)** GUA SAL CRI

*Schoenicus salvini* Champion, 1884: 19.

*Schoenicus niger* Champion, 1884: 20. Synonymy: Freude (1968: 96).

***Phegoneus salvini subaeneus* Casey, 1907** PAN

*Phegoneus subaeneus* Casey, 1907: 428.

***Phegoneus viridis* (Champion, 1884)** MEX (CI CL DU GE JA OA PU SI) GUA CRI

*Schoenicus viridis* Champion, 1884: 19.

**Genus *Polemiotus* Casey, 1907** [M]

*Polemiotus* Casey, 1907: 379, 381. Type species: *Epitragus submetallicus* LeConte, 1854, original designation.

***Polemiotus submetallicus* (LeConte, 1854)** USA (AZ)

*Epitragus submetallicus* LeConte, 1854c: 224.

*Polemiotus humeralis* Casey, 1907: 382. Synonymy: Freude (1967: 223).

Polemiotus humeralis var. acuticauda Casey, 1907: 383. Synonymy: Freude (1967: 223).

**Genus *Schoenicus* LeConte, 1866** [M]

*Schoenicus* LeConte, 1866b: 109. Type species: *Schoenicus puberulus* LeConte, 1866, monotypy.

***Schoenicus puberulus* LeConte, 1866** USA (FL GA MD MS NC NJ NY SC)

*Schoenicus puberulus* LeConte, 1866b: 110.

**Genus *Tydeolus* Champion, 1884** [M]

*Tydeolus* Champion, 1884: 37. Type species: *Tydeolus atratus* Champion, 1884, subsequent designation (Kirby 1885: 80).

***Tydeolus atratus* Champion, 1884** MEX (PU)

*Tydeolus atratus* Champion, 1884: 37.

*Tydeolus tibialis* Champion, 1884: 37. Synonymy: Freude (1968: 122).

*Tydeolus singularis* Champion, 1884: 37. Synonymy: Freude (1968: 122).

**Tribe Nyctoporini Lacordaire, 1859**

Nyctoporides Lacordaire, 1859: 130. Type genus: *Nyctoporis* Eschscholtz, 1831.

**Genus *Nyctoporis* Eschscholtz, 1831** [F]

*Nyctoporis* Eschscholtz, 1831: 10, 11. Type species: *Nyctoporis cristata* Eschscholtz, 1831, subsequent designation (Hope 1841: 124).

*Emeax* Pascoe, 1866: 450. Type species: *Emeax sculpturatus* Pascoe, 1866 (= *Nyctoporis cristata* Eschscholtz, 1831), monotypy. Synonymy: LeConte (1873: 334).

*Enneacoides* Fairmaire, 1881: 277. Type species: *Enneacoides vinculiger* Fairmaire, 1881 (= *Nyctoporis carinata* LeConte, 1851), monotypy. Synonymy: Gebien (1908a: 287).

***Nyctoporis aequicollis* Eschscholtz, 1831** USA (CA)

*Nyctoporis aequicollis* Eschscholtz, 1831: 12.

*Nyctoporis tetrica* Casey, 1907: 510. **New synonymy** [RLA].

*Nyctoporis maura* Casey, 1907: 512. **New synonymy** [RLA].

***Nyctoporis carinata* LeConte, 1851** USA (CA)

*Nyctoporis carinata* LeConte, 1851: 138.

*Nyctoporis segnis* Casey, 1907: 511. Synonymy: Blaisdell (1931: 43).

*Enneacoides vinculiger* Fairmaire, 1881: 277. Synonymy: Gebien (1910: 118)^26^.

***Nyctoporis cristata* Eschscholtz, 1831** USA (CA)

*Nyctoporis cristata* Eschscholtz, 1831: 11.

*Nyctoporis galeata* LeConte, 1857: 49. Synonymy: Casey (1907b: 510).

*Emeax sculpturatus* Pascoe, 1866: 450. Synonymy: Carter (1914: 406).

***Nyctoporis sponsa* Casey, 1907** USA (CA)

*Nyctoporis sponsa* Casey, 1907: 510.

*Nyctoporis pullata* Casey, 1907: 510. **New synonymy** [RLA].

***Nyctoporis vandykei* Blaisdell, 1931** USA (CA)

*Nyctoporus* [sic!] *vandykei* Blaisdell, 1931: 41.

**Tribe Stenosini Schaum, 1859**

Tagénites Solier, 1834: 503. Type genus: *Tagenia* Latreille, 1802 (= *Stenosis* Herbst, 1799). Note. Use of younger name Stenosini conserved (Art. 40.2) (see Bouchard et al. 2005: 523).

Stenosidae Schaum, 1859: 66. Type genus: *Stenosis* Herbst, 1799.

Typhlusechini Casey, 1907: 281. Type genus: *Typhlusechus* Linell, 1897.

Araeoschizini Casey, 1907: 484. Type genus: *Araeoschizus* LeConte, 1851.

**Genus *Araeoschizus* LeConte, 1851** [M]

*Araeoschizus* LeConte, 1851: 138. Type species: *Araeoschizus costipennis* LeConte, 1851, monotypy.

***Araeoschizus aalbui* Papp, 1981** MEX (BS)

*Araeoschizus aalbui* Papp, 1981: 316.

***Araeoschizus agustinus* Papp, 1998** MEX (BC)

*Araeoschizus agustinus* Papp, 1998: 90.

***Araeoschizus airmeti* Tanner, 1945** USA (ID NV OR)

*Araeoschizus airmeti* Tanner, 1945: 125.

***Araeoschizus alinae* Dajoz, 1984** USA (UT)

*Araeoschizus alinae* Dajoz, 1984: 246.

***Araeoschizus andrewsi* Papp, 1981** USA (CA)

*Araeoschizus andrewsi* Papp, 1981: 318.

***Araeoschizus antennatus antennatus* Blaisdell, 1943** MEX (BC)

*Araeoschizus antennatus* Blaisdell, 1943: 215.

***Araeoschizus antennatus blaisdelli* Papp, 1989** MEX (BC)

*Araeoschizus antennatus blaisdelli* Papp, 1989: 338.

***Araeoschizus antennatus clarki* Papp, 1989** MEX (BC)

*Araeoschizus antennatus clarki* Papp, 1989: 335.

***Araeoschizus apachensis* Papp, 1981** USA (AZ)

*Araeoschizus apachensis* Papp, 1981: 367.

***Araeoschizus arizonicus* Dajoz, 1989** USA (AZ NM)

*Araeoschizus arizonicus* Dajoz, 1989b: 33.

***Araeoschizus armatus* Horn, 1870** USA (CA NV)

*Araeoschizus armatus* Horn, 1870: 275.

***Araeoschizus blomi* Papp, 1998** MEX (BC)

*Araeoschizus blomi* Papp, 1998: 93.

***Araeoschizus colossalis* Papp, 1981** USA (AZ)

*Araeoschizus colossalis* Papp, 1981: 346.

***Araeoschizus costipennis* LeConte, 1851** USA (CA)

*Araeoschizus costipennis* LeConte, 1851: 138.

***Araeoschizus decipiens* Horn, 1890** USA (AZ CO NM TX UT) MEX (CH DU SO)

*Araeoschizus decipiens* Horn, 1890: 342.

***Araeoschizus dolenterus* Papp, 1981** MEX (PU)

*Araeoschizus dolenterus* Papp, 1981: 349.

***Araeoschizus doyeni* Papp, 1981** USA (CA)

*Araeoschizus doyeni* Papp, 1981: 375.

***Araeoschizus duplicatus* Casey, 1907** USA (WY)

*Araeoschizus duplicatus* Casey, 1907: 491.

***Araeoschizus elegantulus* Papp, 1981** MEX (BS)

*Araeoschizus elegantulus* Papp, 1981: 325.

***Araeoschizus exiguus* Casey, 1907** USA (CA)

*Araeoschizus exiguus* Casey, 1907: 487.

***Araeoschizus expeditionis* Papp, 1981** MEX (DU)

*Araeoschizus expeditionis* Papp, 1981: 351.

***Araeoschizus fimbriatus* Casey, 1890** USA (AZ)

*Araeoschizus fimbriatus* Casey, 1890b: 369.

***Araeoschizus giulianii* Papp, 1981** MEX (SO)

*Araeoschizus giulianii* Papp, 1981: 393.

***Araeoschizus hardyi* Papp, 1981** USA (CA)

*Araeoschizus hardyi* Papp, 1981: 308.

***Araeoschizus hardyorum* Papp, 1981** USA (UT)

*Araeoschizus hardyorum* Papp, 1981: 395.

***Araeoschizus hystrix* Papp, 1981** USA (CA)

*Araeoschizus hystrix* Papp, 1981: 330.

***Araeoschizus interjectus* Papp, 1981** MEX (BS)

*Araeoschizus interjectus* Papp, 1981: 332.

***Araeoschizus kaszabi* Papp, 1981** USA (CA)

*Araeoschizus kaszabi* Papp, 1981: 397.

***Araeoschizus kubai* Papp, 1981** USA (AZ)

*Araeoschizus kubai* Papp, 1981: 400.

***Araeoschizus lariversi* Papp, 1981** USA (CA)

*Araeoschizus lariversi* Papp, 1981: 306.

***Araeoschizus lecontei* Papp, 1981** USA (AZ)

*Araeoschizus lecontei* Papp, 1981: 335.

***Araeoschizus limbatus* Blaisdell, 1943** MEX (BS)

*Araeoschizus limbatus* Blaisdell, 1943: 214.

***Araeoschizus magdae* Papp, 1989** MEX (GE)

*Araeoschizus magdae* Papp, 1989: 338.

***Araeoschizus mexicanus* Champion, 1892** MEX (DU GE OA)

*Araeoschizus mexicanus* Champion, 1892: 491.

***Araeoschizus microcephalus* Papp, 1981** MEX (CH)

*Araeoschizus microcephalus* Papp, 1981: 379.

***Araeoschizus muthi* Dajoz, 1998** USA (CA)

*Araeoschizus muthi* Dajoz, 1998: 87.

***Araeoschizus orientalis* Dajoz, 1991** USA (TX)

*Araeoschizus orientalis* Dajoz, 1991: 172.

***Araeoschizus percellosus* Papp, 1981** MEX (BC)

*Araeoschizus percellosus* Papp, 1981: 312.

***Araeoschizus problematicus* Papp, 1981** MEX (ZA)

*Araeoschizus problematicus* Papp, 1981: 402.

***Araeoschizus regularis* Horn, 1870** USA (AZ UT) MEX (SO)

*Araeoschizus regularis* Horn, 1870: 274.

***Araeoschizus rufus* Dajoz, 1991** USA (CA)

*Araeoschizus rufus* Dajoz, 1991: 165.

***Araeoschizus setosiformis* Papp, 1981** USA (UT)

*Araeoschizus setosiformis* Papp, 1981: 382.

***Araeoschizus similaris* Papp, 1981** USA (NM)

*Araeoschizus similaris* Papp, 1981: 384.

***Araeoschizus simplex* Casey, 1890** USA (AZ NM TX) MEX (CH)

*Araeoschizus simplex* Casey, 1890b: 369.

***Araeoschizus simulans* Casey, 1907** USA (CA)

*Araeoschizus simulans* Casey, 1907: 488.

***Araeoschizus squamulissimus* Papp, 1981** MEX (BC)

*Araeoschizus squamulissimus* Papp, 1981: 340.

***Araeoschizus sulcicollis disjunctus* Papp, 1981** USA (CA)

*Araeoschizus sulcicollis disjunctus* Papp, 1981: 364.

***Araeoschizus sulcicollis sulcicollis* Horn, 1870** USA (CA NV)

*Araeoschizus sulcicollis* Horn, 1870: 274.

***Araeoschizus tenuis* Casey, 1907** USA (AZ)

*Araeoschizus tenuis* Casey, 1907: 486.

***Araeoschizus texanus* Dajoz, 1989** USA (TX)

*Araeoschizus texanus* Dajoz, 1989a: 149.

***Araeoschizus utahensis* Papp, 1981** USA (UT)

*Araeoschizus utahensis* Papp, 1981: 389.

***Araeoschizus wasbauerorum* Papp, 1981** MEX (SO)

*Araeoschizus wasbauerorum* Papp, 1981: 342.

**Genus *Caribanosis* Nabozhenko, Kirejtshuk, Merkl, Varela, Aalbu and Smith, 2016** [M]

*Caribanosis* Nabozhenko, Kirejtshuk, Merkl, Varela, Aalbu and Smith, 2016: 568^27^. Type species: *Rhypasma quisqueyanus* Garrido and Varela, 2011, original designation.

***Caribanosis quisqueyanus* (Garrido and Varela, 2011)** DOM

*Rhypasma quisqueyanus* Garrido and Varela, 2011: 32.

**Genus *Discopleurus* Lacordaire, 1859** [M]

*Pleurophorus* Solier, 1851: 162 [junior homonym of *Pleurophorus* Mulsant, 1842]. Type species: *Pleurophorus quadricollis* Solier, 1851, monotypy.

*Discopleurus* Lacordaire, 1859: 105. Replacement name for *Pleurophorus* Solier, 1851.

***Discopleurus mesoamericanus* Aalbu and Andrews, 1996** HON CRI PAN

*Discopleurus mesoamericanus* Aalbu and Andrews, 1996: 27.

**Genus *Typhlusechus* Linell, 1897** [M]

*Typhlusechus* Linell, 1897: 154. Type species: *Typhlusechus singularis* Linell, 1897, original designation.

***Typhlusechus balsasensis* Aalbu and Andrews, 1985** MEX (MI)

*Typhlusechus balsasensis* Aalbu and Andrews, 1985: 4.

***Typhlusechus chemehuevii* Aalbu and Andrews, 1985** USA (CA)

*Typhlusechus chemehuevii* Aalbu and Andrews, 1985: 3.

***Typhlusechus ignotus* Doyen, 1990** MEX (JA)

*Typhlusechus ignotus* Doyen, 1990: 227.

***Typhlusechus peninsularis* Aalbu and Andrews, 1985** MEX (BS)

*Typhlusechus peninsularis* Aalbu and Andrews, 1985: 5.

***Typhlusechus singularis* Linell, 1897** USA (CA)

*Typhlusechus singularis* Linell, 1897: 155.

***Typhlusechus spilmani* Aalbu and Andrews, 1985** USA (TX) MEX (DU)

*Typhlusechus spilmani* Aalbu and Andrews, 1985: 6.

**Tribe Vacronini Gebien, 1910**

Vacroninae Gebien, 1910a: 118. Type genus: *Vacronus* Casey, 1907 (= *Alaephus* Horn, 1870).

**Genus *Alaephus* Horn, 1870** [M]

*Alaephus* Horn, 1870: 346. Type species: *Alaephus pallidus* Horn, 1870, monotypy.

*Vacronus* Casey, 1907: 501, 508. Type species: *Vacronus tenuicornis* Casey, 1907, original designation. Synonymy: Doyen and Lawrence (1979: 350).

***Alaephus convergens* Casey, 1924** USA (UT)

*Alaephus convergens* Casey, 1924: 324.

***Alaephus gracilicornis* Casey, 1924** USA (NM)

*Alaephus gracilicornis* Casey, 1924: 325.

***Alaephus gracilis* Fall, 1905** USA (AZ)

*Alaephus gracilis* Fall, 1905: 276.

***Alaephus longicornis* Casey, 1924** USA (CA)

*Alaephus longicornis* Casey, 1924: 325.

***Alaephus macilentus* Casey, 1891** USA (AZ CA NM)

*Alaephus macilentus* Casey, 1891: 61.

*Alaephus nitidipennis* Fall, 1905: 275. Synonymy: Fall (1907b: 175).

***Alaephus madarensis* Casey, 1924** USA (CA)

*Alaephus madarensis* Casey, 1924: 324.

***Alaephus nevadensis* Tanner, 1965** USA (NV)

*Alaephus nevadensis* Tanner [in Tanner and Packtam], 1965: 39.

***Alaephus pallidus* Horn, 1870** USA (CA)

*Alaephus pallidus* Horn, 1870: 346.

***Alaephus puberulus* Fall, 1907** USA (AZ UT)

*Alaephus puberulus* Fall, 1907b: 175.

***Alaephus quadricollis* Casey, 1924** USA (UT)

*Alaephus quadricollis* Casey, 1924: 326.

***Alaephus tenuicornis* (Casey, 1907)** USA (CA)

*Vacronus tenuicornis* Casey, 1907: 508.

**Genus *Eupsophulus* Cockerell, 1906** [M]

*Eupsophus* Horn, 1870: 347 [junior homonym of *Eupsophus* Fitzinger, 1843]. Type species: *Eupsophus castaneus* Horn, 1870, monotypy.

*Eupsophulus* Cockerell, 1906: 242. Replacement name for *Eupsophus* Horn, 1870.

***Eupsophulus brevipennis* Casey, 1924** USA (AZ)

*Eupsophulus brevipennis* Casey, 1924: 323.

***Eupsophulus castaneus* (Horn, 1870)** USA (AZ CA NV TX) MEX (BC CO SO)

*Eupsophus castaneus* Horn, 1870: 347.

***Eupsophulus horni* (Champion, 1885)** MEX (BS)

*Eupsophus horni* Champion, 1885: 122.

**Subfamily TENEBRIONINAE Latreille, 1802**

Tenebrionites Latreille, 1802: 165. Type genus: *Tenebrio* Linnaeus, 1758.

**Tribe Acropteronini Doyen, 1989**

Acropteronini Doyen, 1989: 288. Type genus: *Acropteron* Perty, 1832.

**Genus *Acropteron* Perty, 1832** [N]

*Acropteron* Perty, 1832: 64. Type species: *Acropteron rufipes* Perty, 1832, subsequent designation (Hope 1841: 133).

*Arthroplatus* Solier, 1851: 246. Type species: *Arthroplatus pallipes* Solier, 1851, monotypy. Synonymy: Mäklin (1862: 1).

***Acropteron agriloides* Mäklin, 1862** MEX (CI GE OA TB VE) GUA

*Acropteron agriloides* Mäklin, 1862: 17.

***Acropteron angulicolle* Champion, 1886** NIC

*Acropteron angulicolle* Champion, 1886: 255.

***Acropteron belti* Champion, 1886** NIC PAN

*Acropteron belti* Champion, 1886: 253.

***Acropteron brunneum* Mäklin, 1862** CRI / SA

*Acropteron brunneum* Mäklin, 1862: 15.

***Acropteron calcaratum* Champion, 1886** GUA

*Acropteron calcaratum* Champion, 1886: 255.

***Acropteron chabrieri* Fleutiaux and Sallé, 1890** LAN

*Acropteron chabrieri* Fleutiaux and Sallé, 1890: 429.

***Acropteron laevipes* Champion, 1886** NIC

*Acropteron laevipes* Champion, 1886: 257.

***Acropteron langurioides* Champion, 1886** PAN

*Acropteron langurioides* Champion, 1886: 254.

***Acropteron longipenne* Champion, 1886** GUA PAN

*Acropteron longipenne* Champion, 1886: 256.

***Acropteron maklini* Champion, 1886** PAN

*Acropteron mäklini* Champion, 1886: 254.

***Acropteron mexicanum* Champion, 1886** MEX (VE)

*Acropteron mexicanum* Champion, 1886: 256.

***Acropteron puncticolle* Champion, 1886** PAN

*Acropteron puncticolle* Champion, 1886: 256.

***Acropteron quadraticolle* Champion, 1896** LAN

*Acropteron quadraticolle* Champion, 1896: 29.

***Acropteron rugipes* Champion, 1886** NIC

*Acropteron rugipes* Champion, 1886: 257.

**Tribe Alphitobiini Reitter, 1917**

Alphitobiini Reitter, 1917: 58. Type genus: *Alphitobius* Stephens, 1829.

**Genus *Alphitobius* Stephens, 1829** [M]

*Alphitobius* Stephens, 1829: 19. Type species: *Helops picipes* Panzer, 1794 (= *Opatrum laevigatum* Fabricius, 1781), monotypy.

*Heterophaga* Dejean, 1834: 199. Type species: *Tenebrio mauritanicus* Fabricius, 1792 (= *Opatrum laevigatum* Fabricius, 1781), subsequent designation (Duponchel 1845: 601). Synonymy: Wollaston (1854: 498).

*Cryptops* Solier, 1851: 235. Type species: *Cryptops ulomoides* Solier, 1851 (= *Tenebrio diaperinus* Panzer, 1797), monotypy. Synonymy: Philippi (1887: 735).

*Microphyes* MacLeay, 1873: 286. Type species: *Microphyes rufipes* MacLeay, 1873 (= *Opatrum laevigatum* Fabricius, 1781), monotypy. Synonymy: Blair (1914: 486).

**Figure 15.**
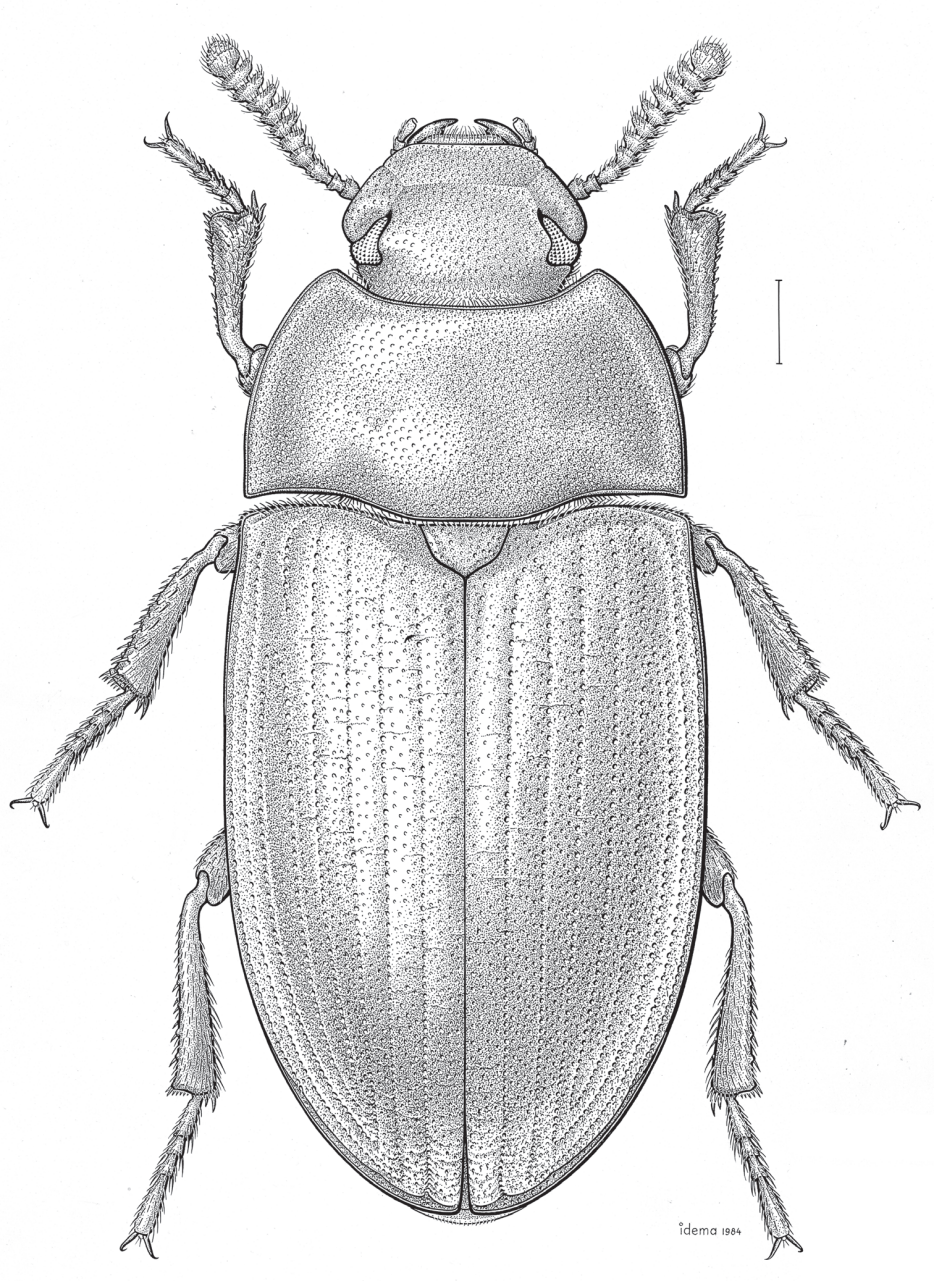
*Alphitobius diaperinus* (Panzer, 1797). Scale bar = 1 mm.

***Alphitobius diaperinus* (Panzer, 1797)** [Fig. 15] CAN (AB BC MB NB NF NS ON PE QC SK) USA (FL GA IN MD MI NC NY OH SC SD VA WA WI) MEX (CH DU GU PU QR VE) NIC / BAH CUB CAY JAM HAI DOM PRI LAN / SA – Adventive

*Tenebrio diaperinus* Panzer, 1797: 16.

*Cryptops ulomoides* Solier, 1851: 236. Synonymy: Schawaller and Grimm (2014: 174).

***Alphitobius laevigatus* (Fabricius, 1781)** CAN (BC NB ON QC) USA (CT FL GA ID IN NY OH OR PA SC SD WA WI) MEX (BS CH CO DU NL TA VE YU) BEL NIC CRI PAN / CUB CAY JAM PRI LAN / SA – Adventive

*Opatrum laevigatum* Fabricius, 1781: 90.

*Helops piceus* Olivier, 1793: 50. Synonymy: Blair (1914: 486).

*Helops picipes* Panzer, 1794: 4. Synonymy: Spilman (1966: 7).

Alphitobius piceus var. ruficolor Pic, 1925b: 11. Synonymy: Gebien (1940: 779).

**Tribe Amarygmini Gistel, 1848**

Amarygmiidae Gistel, 1848: [10]. Type genus: *Amarygmus* Dalman, 1823.

Mégacanthides Lacordaire, 1859: 467. Type genus: *Megacantha* Westwood, 1843.

Méracanthides Lacordaire, 1859: 464. Type genus: *Meracantha* Kirby, 1837.

**Genus *Cymatothes* Dejean, 1834** [M]

*Cymatothes* Dejean, 1834 [30 June]: 208. Type species: *Helops undatus* Fabricius 1792 (= *Erotylus nebulosus* Fabricius, 1781), monotypy.

*Physignathus* Gistel, 1834 [23 September]: 22 [junior homonym of *Physignathus* Cuvier, 1829]. Type species: *Helops undatus* Fabricius, 1792 (= *Erotylus nebulosus* Fabricius, 1781), monotypy. Synonymy: Bousquet and Bouchard (2017a: 132).

*Py﻿anisia* Laporte, 1840: 235. Type species: *Helops undatus* Fabricius, 1792 (= *Erotylus nebulosus* Fabricius, 1781), subsequent designation (Lacordaire 1859: 476). Synonymy: Chevrolat (1847: 643).

*Pyganisia* Hope, 1841: 133. Type species: *Helops undatus* Fabricius, 1792 (= *Erotylus nebulosus* Fabricius, 1781), original designation. Synonymy: Chevrolat (1847: 643).

***Cymatothes fumosus* (Champion, 1887)** GUA

*Pyanisia fumosa* Champion, 1887: 331.

***Cymatothes laevis* (Champion, 1893)** MEX (GE)

*Pyanisia laevis* Champion, 1893a: 561.

***Cymatothes longicollis* (Champion, 1887)** GUA

*Pyanisia longicollis* Champion, 1887: 331.

***Cymatothes nebulosus nebulosus* (Fabricius, 1781)** MEX GUA BEL NIC CRI PAN / CUB HAI LAN / SA

*Erotylus nebulosus* Fabricius, 1781: 158.

*Helops undatus* Fabricius, 1792a: 122. Synonymy: Blair (1935: 103).

***Cymatothes opacus* Solier, 1848** USA (FL) MEX (CH CI DU GE OA PU VE)

*Cymatothes opacus* Solier, 1848: 180.

*Cymatothes coarctatus* Solier, 1848: 181. Synonymy: Champion (1887: 330).

***Cymatothes unicolor* Solier, 1848** USA (AL FL TX) MEX (CI DU JA NA VE) GUA BEL NIC CRI PAN / BAH CUB PRI LAN

*Helops tristis* Laporte, 1840: 236 [junior primary homonym of *Helops tristis* Rossi, 1790].

*Cymatothes unicolor* Solier, 1848: 182. Synonymy: Champion (1887: 330).

***Cymatothes uniformis* (C.O. Waterhouse, 1878)** JAM

*Hoplonyx uniformis* C.O. Waterhouse, 1878: 306.

**Genus *Meracantha* Kirby, 1837** [F]

*Meracantha* Kirby, 1837: 237. Type species: *Meracantha canadensis* Kirby, 1837 (= *Helops contractus* Palisot de Beauvois, 1812), monotypy.

*Falacer* Laporte, 1840: 233. Type species: *Acanthopus cupreus* Laporte, 1840 (= *Helops contractus* Palisot de Beauvois, 1812), **present designation**. Synonymy: Lacordaire (1859: 466).

*Physocoelus* Haldeman, 1850: 5. Type species: *Helops contractus* Palisot de Beauvois, 1812, monotypy. Synonymy: Lacordaire (1859: 466).

***Meracantha contracta* (Palisot de Beauvois, 1812)** CAN (ON) USA (AL CT FL GA IA IN LA MD MI NC NY OH PA SC TN TX VA WI)

*Helops contractus* Palisot de Beauvois, 1812: 121.

*Meracantha canadensis* Kirby, 1837: 238. Synonymy: Melsheimer (1853: 140).

*Acanthopus cupreus* Laporte, 1840: 233. Synonymy: Lacordaire (1859: 466).

*Acanthopus rugosus* Laporte, 1840: 233. Synonym (in doubt): Papp (1961d: 135).

*Helops tumidus* Melsheimer, 1846: 61. Synonymy: Melsheimer (1853: 140).

*Psorodes inflata* Solier, 1848: 167. Synonymy (with *M. canadensis* Kirby): Schaum (1850: 181).

**Genus *Plesiophthalmus* Motschulsky, 1857** [M]

*Plesiophthalmus* Motschulsky, 1857: 34. Type species: *Plesiophthalmus nigrocyaneus* Motschulsky, 1857, monotypy.

*Cyriogeton* Pascoe, 1871: 356. Type species: *Cyriogeton insignis* Pascoe, 1871, monotypy. Synonymy: Masumoto (1989a: 536).

***Plesiophthalmus spectabilis* Harold, 1875** USA (MD)^28^ – Adventive

*Plesiophthalmus spectabilis* Harold, 1875: 293.

*Plesiophthalmus obesus* Marseul, 1876: 319. Synonymy: Harold (1876: 85).

*Plesiophthalmus arciferens* Fairmaire [in Deyrolle and Fairmaire], 1878: 120. Synonymy: Masumoto (1989b: 743).

*Plesiophthalmus subparallelus* Pic, 1916: 11. Synonymy: Masumoto (1989b: 743).

**Tribe Amphidorini LeConte, 1862**

Amphidorae LeConte, 1862a: 239. Type genus: *Amphidora* Eschscholtz, 1829.

Eleodiini Blaisdell, 1909: 27. Type genus: *Eleodes* Eschscholtz, 1829.

Eleodopsinae Blaisdell, 1939b: 51. Type genus: *Eleodopsis* Blaisdell, 1939 (= *Eleodes* Eschscholtz, 1829).

Lariversiina La Rivers, 1948: 98. Type genus: *Lariversius* Blaisdell, 1947.

Trogloderina La Rivers, 1948: 98. Type genus: *Trogloderus* LeConte, 1879.

**Genus *Eleodes* Eschscholtz, 1829^29^** [F]

*Eleodes* Eschscholtz, 1829: 8. Type species: *Eleodes dentipes* Eschscholtz, 1829, subsequent designation (Hope 1841: 124).

*Elaeodes* Gemminger [in Gemminger and Harold], 1870: 1868. Unjustified emendation of *Eleodes* Eschscholtz, 1829, not in prevailing usage.

**Subgenus Amphidora Eschscholtz, 1829**

*Amphidora* Eschscholtz, 1829: 9. Type species: *Amphidora littoralis* Eschscholtz, 1829, monotypy. **Status revised** [ADS & MAJ].

***Eleodes littoralis* (Eschscholtz, 1829)** USA (CA)

*Amphidora littoralis* Eschscholtz, 1829: 9^30^.

***Eleodes nigropilosa* (LeConte, 1851)** USA (CA) MEX (BC)

*Amphidora nigropilosa* LeConte, 1851: 136.

*Amphidora tenebrosa* Horn, 1870: 329. Synonymy: Triplehorn (1996: 13).

***Eleodes subdeplanata* (Blaisdell, 1943)** MEX (BC BS)

*Amphidora subdeplanata* Blaisdell, 1943: 252.

**Subgenus Ardeleodes Blaisdell, 1937**

*Ardeleodes* Blaisdell, 1937b: 128^31^. Type species: *Eleodes tibialis* Blaisdell, 1909, original designation.

***Eleodes tibialis* Blaisdell, 1909** MEX (BS)

*Eleodes tibialis* Blaisdell, 1909: 313.

Eleodes tibialis forma oblonga Blaisdell, 1909: 315. **New synonymy** [YB].

**Subgenus Blapylis Horn, 1870**

*Blapylis* Horn, 1870: 315. Type species: *Eleodes cordata* Eschscholtz, 1829, **present designation**. Synonymy: Doyen and Lawrence (1979: 367).

*Eleodopsis* Blaisdell, 1939b: 52. Type species: *Eleodopsis subvestita* Blaisdell, 1939, original designation. Synonymy: Spilman (1962b: 59).

***Eleodes alticola* Blaisdell, 1925** USA (CA)

*Eleodes parvicollis alticola* Blaisdell, 1925c: 387.

***Eleodes aristata* Somerby, 1977** USA (CA)

*Eleodes aristata* Somerby, 1977: 22.

***Eleodes bishopensis* Somerby and Doyen, 1976** USA (CA)

*Eleodes bishopensis* Somerby and Doyen, 1976: 257.

***Eleodes blanchardii* Blaisdell, 1909** USA (CA)

*Eleodes blanchardii* Blaisdell, 1909: 339.

***Eleodes brunnipes* Casey, 1890** USA (CA CO ID NV WY)

*Eleodes brunnipes* Casey, 1890b: 402.

Eleodes pimelioides var. brevisetosa Blaisdell, 1918b: 162. Synonymy: Blaisdell (1925a: 80).

***Eleodes caseyi* Blaisdell, 1909** USA (CA NV)

*Eleodes caseyi* Blaisdell, 1909: 388.

***Eleodes clavicornis* Eschscholtz, 1829** USA (CA)

*Eleodes clavicornis* Eschscholtz, 1829: 11.

*Eleodes impressicollis* Boheman, 1858: 90. Synonymy: LeConte (1866a: 60).

***Eleodes consobrina* LeConte, 1851** USA (CA) MEX (BC)

*Eleodes consobrina* LeConte, 1851: 135.

*Eleodes veseyi* LeConte, 1858c: 187. Synonymy: LeConte (1866a: 60).

***Eleodes constricta* LeConte, 1858** CAN (BC) USA (CA UT)

*Eleodes constrictus* LeConte, 1858c: 187.

*Eleodes* [*manni* var.] *variolosa* Blaisdell, 1917: 223. **New synonymy** [based on Somerby (1972: 161) unpublished thesis].

***Eleodes cooperi* Somerby and Doyen, 1976** USA (CA)

*Eleodes cooperi* Somerby and Doyen, 1976: 253.

***Eleodes cordata* Eschscholtz, 1829** USA (CA)

*Eleodes cordata* Eschscholtz, 1829: 12.

Eleodes cordata forma sublaevis Blaisdell, 1909: 381. **New synonymy** [based on Somerby (1972: 254) unpublished thesis].

Eleodes cordata forma intermedia Blaisdell, 1909: 381. **New synonymy** [YB].

Eleodes cordata forma oblonga Blaisdell, 1909: 383. **New synonymy** [YB].

Eleodes cordata forma elongata Blaisdell, 1909: 383. **New synonymy** [YB].

*Eleodes* [*cordata* var.] *adulterina* Blaisdell, 1917: 224. **New synonymy** [based on Somerby (1972: 254) unpublished thesis].

***Eleodes fuchsii* Blaisdell, 1909** USA (CA)

*Eleodes fuchsii* Blaisdell, 1909: 343.

Eleodes hornii var. monticula Blaisdell, 1918c: 385. **New synonymy** [based on Somerby (1972: 199) unpublished thesis].

*Eleodes manni sierra* Blaisdell, 1926a: 78. **New synonymy** [based on Somerby (1972: 199) unpublished thesis].

***Eleodes hoppingii* Blaisdell, 1909** USA (CA)

*Eleodes hoppingii* Blaisdell, 1909: 368.

***Eleodes hornii hornii* Blaisdell, 1909** USA (CA)

*Eleodes hornii* Blaisdell, 1909: 350.

***Eleodes hybrida* Blaisdell, 1917** USA (CA)

*Eleodes* [*cordata* var.] *hybrida* Blaisdell, 1917: 225.

***Eleodes inculta* LeConte, 1861** USA (CA)

*Eleodes inculta* LeConte, 1861b: 352.

Eleodes inculta var. affinis Blaisdell, 1918c: 384. Synonymy: Miller (1985: 21).

***Eleodes kaweana* Blaisdell, 1933** USA (CA)

*Eleodes kaweana* Blaisdell, 1933b: 203.

***Eleodes lariversi* Somerby and Doyen, 1976** USA (CA)

*Eleodes lariversi* Somerby and Doyen, 1976: 256.

***Eleodes lecontei* Horn, 1870** USA (CO)

*Eleodes subaspera* LeConte, 1866b: 115 [junior primary homonym of *Eleodes subaspera* Solier, 1848].

*Eleodes lecontei* Horn, 1870: 316. Replacement name for *Eleodes subaspera* LeConte, 1866.

***Eleodes manni* Blaisdell, 1917** USA (WA)

*Eleodes manni* Blaisdell, 1917: 221.

***Eleodes nana* Blaisdell, 1909** USA (CA NV)

Eleodes tenebrosa var. nana Blaisdell, 1909: 328.

***Eleodes neotomae* Blaisdell, 1909** USA (CA)

*Eleodes neotomae* Blaisdell, 1909: 347.

***Eleodes novoverrucula* Boddy, 1957** CAN (AB BC) USA (ID MT OR WA)

*Eleodes novoverrucula* Boddy, 1957: 195.

***Eleodes nunenmacheri* Blaisdell, 1918** USA (CA OR)

*Eleodes nunenmacheri* Blaisdell, 1918b: 163.

***Eleodes oregona* Blaisdell, 1941** USA (OR)

*Eleodes oregona* Blaisdell, 1941b: 157.

***Eleodes orophila* Somerby, 1977** USA (AZ NM)

*Eleodes orophilus* Somerby, 1977: 24.

***Eleodes panamintensis* Somerby, 1977** USA (CA)

*Eleodes panamintensis* Somerby, 1977: 20.

***Eleodes parvicollis* Eschscholtz, 1829** USA (CA)

*Eleodes parvicollis* Eschscholtz, 1829: 11.

Eleodes parvicollis var. squalida Blaisdell, 1918c: 380. **New synonymy** [based on Somerby (1972: 188) unpublished thesis].

***Eleodes patulicollis* Blaisdell, 1932** USA (UT)

*Eleodes manni dilaticollis* Blaisdell, 1925c: 388 [junior primary homonym of *Eleodes dilaticollis* Champion, 1884].

*Eleodes patulicollis* Blaisdell, 1932a: 78. Replacement name for *Eleodes dilaticollis* Blaisdell, 1925.

**Figure 16.**
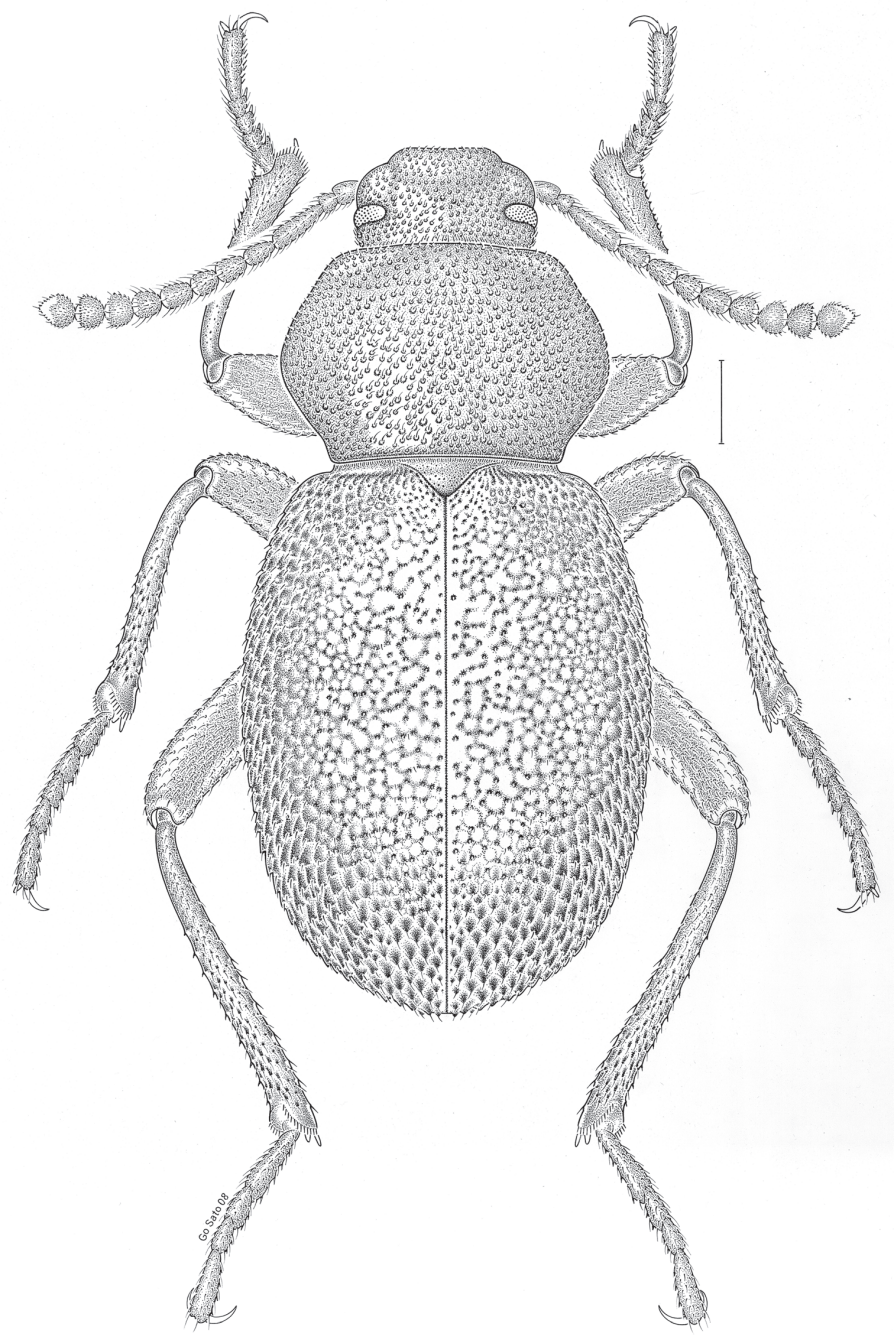
Eleodes (Blapylis) pimelioides Mannerheim, 1843. Scale bar = 1 mm.

***Eleodes pimelioides* Mannerheim, 1843** [Fig. 16] CAN (BC) USA (CA OR UT WA)

*Eleodes pimelioides* Mannerheim, 1843: 274.

*Eleodes viator* LeConte, 1858c: 188. Synonymy: Horn (1870: 318).

Eleodes nunenmacheri var. verrucula Blaisdell, 1918b: 164. Synonymy: Bousquet and Campbell (1991: 257) [based on Somerby (1972: 238) unpublished thesis].

Eleodes pimelioides var. patruelis Blaisdell, 1918c: 382. Synonymy (with *E. nunenmacheri verrucula* Blaisdell): Boddy (1957: 197).

***Eleodes planata* Eschscholtz, 1829** USA (CA)

*Eleodes planata* Eschscholtz, 1829: 12.

*Eleodes reflexicollis* Mannerheim, 1843: 270. **New synonymy** [based on Somerby (1972: 187) unpublished thesis].

Eleodes parvicollis forma farallonica Blaisdell, 1909: 356. **New synonymy** [based on Somerby (1972: 187) unpublished thesis].

***Eleodes producta* Mannerheim, 1843** USA (CA)

*Eleodes producta* Mannerheim, 1843: 271.

***Eleodes propinqua* Blaisdell, 1918** USA (CA)

*Eleodes propinqua* Blaisdell, 1918b: 165.

***Eleodes robinetti* Boddy, 1957** USA (OR WA)

*Eleodes robinetti* Boddy, 1957: 194.

***Eleodes rotundipennis* LeConte, 1857** CAN (BC) USA (OR WA)

*Eleodes rotundipennis* LeConte, 1857: 50.

*Eleodes stricta* LeConte, 1857: 50. Synonymy: Boddy (1965: 158).

*Eleodes subligata* LeConte, 1857: 50. Synonymy: Boddy (1965: 158).

*Eleodes indentata* Blaisdell, 1935a: 28. **New synonymy** [based on Somerby (1972: 237) unpublished thesis].

***Eleodes scabripennis* LeConte, 1859** USA (CA)

*Eleodes scabripennis* LeConte, 1859b: 77.

***Eleodes scabriventris* Blaisdell, 1933** USA (CA)

*Eleodes scabriventris* Blaisdell, 1933b: 202.

***Eleodes scabrosa* Eschscholtz, 1829** USA (CA OR)

*Eleodes scabrosa* Eschscholtz, 1829: 11.

*Eleodes intricata* Mannerheim, 1843: 273. **New synonymy** [based on Somerby (1972: 163) unpublished thesis].

***Eleodes schlingeri* Somerby and Doyen, 1976** USA (CA)

*Eleodes schlingeri* Somerby and Doyen, 1976: 254.

***Eleodes schwarzii* Blaisdell, 1909** USA (ID OR WA)

*Eleodes schwarzii* Blaisdell, 1909: 406.

***Eleodes snowii* Blaisdell, 1909** USA (AZ CO NM NV)

*Eleodes snowii* Blaisdell, 1909: 317.

***Eleodes spilmani* Somerby and Doyen, 1976** USA (CA)

*Eleodes spilmani* Somerby and Doyen, 1976: 258.

***Eleodes strumosa* Blaisdell, 1932** USA (UT NV)

*Eleodes strumosa* Blaisdell, 1932a: 76.

***Eleodes subvestita* (Blaisdell, 1939)** USA (CA)

*Eleodopsis subvestita* Blaisdell, 1939b: 53.

***Eleodes tenebrosa* Horn, 1870** CAN (BC) USA (CA NV UT)

*Eleodes tenebrosa* Horn, 1870: 316.

*Eleodes horni fenyesi* Blaisdell, 1926a: 77. **New synonymy** [based on Somerby (1972: 161) unpublished thesis].

***Eleodes triplehorni* Somerby and Doyen, 1976** MEX (BC)

*Eleodes triplehorni* Somerby and Doyen, 1976: 252.

***Eleodes trita* Blaisdell, 1917** USA (CA OR)

*Eleodes* [*parvicollis* var.] *trita* Blaisdell, 1917: 225.

***Eleodes tuberculata* Eschscholtz, 1829** USA (CA)

*Eleodes tuberculata* Eschscholtz, 1829: 12.

Eleodes cordata var. horrida Blaisdell, 1918c: 383. **New synonymy** [based on Somerby (1972: 255) unpublished thesis].

***Eleodes versatilis* Blaisdell, 1921** USA (OR WA)

*Eleodes rotundipennis versatilis* Blaisdell, 1921b: 217.

*Eleodes oblonga* Blaisdell, 1933b: 206 [junior primary homonym of *Eleodes tibialis oblonga* Blaisdell, 1909]. **New synonymy** [based on Somerby (1972: 190) unpublished thesis].

*Eleodes formosus* Thomas, 2005: 551. Replacement name for *Eleodes oblonga* Blaisdell, 1933.

***Eleodes volcanensis* Somerby, 1977** USA (CA OR)

*Eleodes volcanensis* Somerby, 1977: 23.

***Eleodes wakelandi* Somerby, 1977** USA (ID OR UT)

*Eleodes wakelandi* Somerby, 1977: 19.

**Subgenus Caverneleodes Triplehorn, 1975**

*Caverneleodes* Triplehorn, 1975: 39. Type species: *Eleodes easterlai* Triplehorn, 1975, original designation.

***Eleodes easterlai* Triplehorn, 1975** USA (TX) MEX (CO)

*Eleodes easterlai* Triplehorn, 1975: 39.

***Eleodes grutus* Aalbu, Smith and Triplehorn, 2012** ^32^ MEX (NL)

*Eleodes grutus* Aalbu, Smith and Triplehorn, 2012: 206.

***Eleodes guadalupensis* Aalbu, Smith and Triplehorn, 2012** USA (NM)

*Eleodes guadalupensis* Aalbu, Smith and Triplehorn, 2012: 208.

***Eleodes labialis* Triplehorn, 1975** USA (TX) MEX (CH)

*Eleodes labialis* Triplehorn, 1975: 42.

***Eleodes leptoscelis* Triplehorn, 1975** USA (AZ)

*Eleodes leptoscelis* Triplehorn, 1975: 42.

***Eleodes microps* Aalbu, Smith and Triplehorn, 2012** USA (CA)

*Eleodes microps* Aalbu, Smith and Triplehorn, 2012: 200.

***Eleodes reddelli* Triplehorn, 2007** MEX (NL)

*Eleodes reddelli* Triplehorn, 2007: 639.

***Eleodes rugosifrons* Triplehorn and Reddell, 1991** MEX (CO NL)

*Eleodes rugosifrons* Triplehorn and Reddell, 1991: 527.

***Eleodes sprousei* Triplehorn and Reddell, 1991** MEX (NL TA)

*Eleodes sprousei* Triplehorn and Reddell, 1991: 525.

***Eleodes thomasi* Aalbu, Smith and Triplehorn, 2012** MEX (CO NL)

*Eleodes thomasi* Aalbu, Smith and Triplehorn, 2012: 204.

***Eleodes wheeleri* Aalbu, Smith and Triplehorn, 2012** USA (AZ)

*Eleodes wheeleri* Aalbu, Smith and Triplehorn, 2012: 208.

***Eleodes wynnei* Aalbu, Smith and Triplehorn, 2012** USA (AZ UT)

*Eleodes wynnei* Aalbu, Smith and Triplehorn, 2012: 201.

**Subgenus Chaseleodes Thomas, 2015**

*Chaseleodes* Thomas, 2015: 122. Type species: *Elaeodes curta* Champion, 1884, original designation.

***Eleodes connata* Solier, 1848** MEX (CH DU FD ME MI MO PU TL VE)

*Eleodes connata* Solier, 1848: 243.

***Eleodes curta* Champion, 1884** MEX (JA ME MI)

*Elaeodes curta* Champion, 1884: 82.

**Subgenus Cratidus LeConte, 1862**

*Cratidus* LeConte, 1862a: 239. Type species: *Amphidora osculans* LeConte, 1851, monotypy.

***Eleodes osculans* (LeConte, 1851)** USA (CA) MEX (BC)

*Amphidora osculans* LeConte, 1851: 136.

*Cratidus fuscipilosus* Casey, 1890b: 407. Synonymy: Triplehorn (1996: 13).

*Cratidus ovipennis* Casey, 1924: 328. Synonymy: Triplehorn (1996: 13).

*Eleodes behrii* Grinnell, 1908: 213. Synonymy: Doyen and Miller (1980: 3).

*Eleodes intermedia* Grinnell, 1908: 215. Synonymy: Doyen and Miller (1980: 3).

***Eleodes ursus* Triplehorn, 1996** MEX (BC BS)

*Cratidus rotundicollis* Horn, 1870: 328 [junior secondary homonym of *Eleodes rotundicollis* Eschscholtz, 1829].

*Eleodes ursus* Triplehorn, 1996: 14. Replacement name for *Eleodes rotundicollis* (Horn, 1870).

**Subgenus Discogenia LeConte, 1866**

*Discogenia* LeConte, 1866b: 117. Type species: *Eleodes scabricula* LeConte, 1858, **present designation**.

***Eleodes acutangula* Blaisdell, 1921** USA (CA)

*Eleodes acutangula* Blaisdell, 1921b: 225.

***Eleodes marginata* Eschscholtz, 1829** USA (CA)

*Eleodes marginata* Eschscholtz, 1829: 10.

*Eleodes fischerii* Mannerheim, 1840: 137. Synonymy: Horn (1870: 320).

***Eleodes scabricula deplanata* Blaisdell, 1909** USA (CA)

Eleodes scabricula forma deplanata Blaisdell, 1909: 444.

***Eleodes scabricula scabricula* LeConte, 1858** USA (CA)

*Eleodes scabricula* LeConte, 1858c: 187.

**Subgenus Eleodes Eschscholtz, 1829**

*Eleodes* Eschscholtz, 1829: 8. Type species: *Eleodes dentipes* Eschscholtz, 1829, subsequent designation (Hope 1841: 124).

***Eleodes acuticauda* LeConte, 1851** USA (CA) MEX (BC)

*Eleodes acuticauda* LeConte, 1851: 135.

*Eleodes laticollis* LeConte, 1851: 135. Synonymy: Horn (1870: 314).

Eleodes acuticauda forma punctata Blaisdell, 1909: 278. Synonymy: Triplehorn et al. (2015: 161).

*Eleodes laticollis apprima* Blaisdell, 1921b: 219. Synonymy: Triplehorn (1996: 9).

***Eleodes acuta* (Say, 1824)** USA (CO KS NM SD TX)

*Blaps acuta* Say, 1824a: 258.

*Eleodes acuta pernigra* Blaisdell, 1937b: 128. Synonymy: Triplehorn et al. (2015: 161).

***Eleodes adumbrata* Blaisdell, 1925** MEX (BC)

*Eleodes adumbrata* Blaisdell, 1925b: 332.

***Eleodes armata* LeConte, 1851** USA (AZ CA ID NV OR UT) MEX (BC SO)

*Eleodes armata* LeConte, 1851: 134.

*Eleodes armata impotens* Blaisdell, 1895: 236. Synonymy: Triplehorn et al. (2015: 163).

Eleodes armata forma sinuata Blaisdell, 1909: 266. Synonymy: Triplehorn et al. (2015: 163).

Eleodes armata var. pumila Blaisdell, 1933b: 197. Synonymy: Triplehorn et al. (2015: 163).

*Eleodes amedeensis* Blaisdell, 1933b: 199. Synonymy: Triplehorn et al. (2015: 163).

*Eleodes striatipennis* Blaisdell, 1942: 134. Synonymy: Triplehorn et al. (2015: 163).

***Eleodes curvidens* Triplehorn and Cifuentes-Ruiz, 2011** MEX (GU MI MO PU)

*Eleodes curvidens* Triplehorn and Cifuentes-Ruiz, 2011: 66.

***Eleodes dentipes* Eschscholtz, 1829** USA (CA)

*Eleodes dentipes* Eschscholtz, 1829: 10.

*Eleodes elegans* Casey, 1890b: 401. Synonymy: Blaisdell (1909: 251).

*Eleodes prominens* Casey, 1890b: 401. Synonymy: Blaisdell (1909: 251).

*Eleodes confinis* Blaisdell, 1895: 237. Synonymy: Blaisdell (1909: 251).

Eleodes dentipes forma pertenuis Blaisdell, 1909: 253. Synonymy: Triplehorn et al. (2015: 164).

Eleodes dentipes forma elongata Blaisdell, 1909: 254. Synonymy: Gebien (1938: 57).

Eleodes dentipes forma robusta Blaisdell, 1909: 255. Synonymy: Triplehorn et al. (2015: 164).

Eleodes dentipes var. perpunctata Blaisdell, 1918c: 386. Synonymy: Gebien (1938: 57).

*Eleodes dentipes marinae* Blaisdell, 1921b: 218. **New synonymy** [ADS & MAJ].

*Eleodes dentipes montana* Blaisdell, 1925c: 385 [junior primary homonym of *Eleodes montana* Champion, 1884]. Synonymy: Triplehorn et al. (2015: 164).

*Eleodes dentipes tularensis* Blaisdell, 1925c: 386. Synonymy: Triplehorn et al. (2015: 164).

*Eleodes paradoxa* Blaisdell, 1932a: 78. Replacement name for *Eleodes montana* Blaisdell, 1925.

*Eleodes dentipes sordida* Blaisdell, 1935a: 30. Synonymy: Triplehorn et al. (2015: 164).

***Eleodes discincta* Blaisdell, 1925** MEX (BC BS)

*Eleodes discincta* Blaisdell, 1925b: 333.

***Eleodes eschscholtzii* Solier, 1848** USA (AZ NM) MEX (BS CO DU SI SO)

*Eleodes eschscholtzii* Solier, 1848: 254.

*Eleodes lucae* LeConte, 1866b: 114. Synonymy: Triplehorn (1996: 10).

*Eleodes wickhami* Horn, 1891: 41. Synonymy: Triplehorn et al. (2015: 167).

***Eleodes femorata* LeConte, 1851** USA (CA) MEX (BC BS)

*Eleodes femorata* LeConte, 1851: 134.

*Eleodes militaris* Horn, 1870: 310. Synonymy: Triplehorn (1996: 7).

Eleodes militaris forma subedentata Blaisdell, 1909: 270. Synonymy: Triplehorn et al. (2015: 167).

*Eleodes inepta* Blaisdell, 1925b: 334. Synonymy: Triplehorn (1996: 7).

*Eleodes marthae* Blaisdell, 1943: 243. Synonymy: Triplehorn (1996: 7).

***Eleodes fiski* Triplehorn, 2015** MEX (TA)

*Eleodes fiski* Triplehorn [in Triplehorn, Thomas and Smith], 2015: 170.

***Eleodes gracilis distans* Blaisdell, 1909** USA (CA)

Eleodes gracilis var. distans Blaisdell, 1909: 242.

***Eleodes gracilis gracilis* LeConte, 1858** USA (AZ NM TX) MEX (CH CO DU SI SO ZA)

*Eleodes gracilis* LeConte, 1858c: 184.

***Eleodes grandicollis grandicollis* Mannerheim, 1843** USA (AZ CA NV) MEX (BC)

*Eleodes grandicollis* Mannerheim, 1843: 266.

*Eleodes elongata* Grinnell, 1908: 215. Synonymy: Doyen and Miller (1980: 3).

***Eleodes grandicollis valida* Boheman, 1858** USA (CA)

*Eleodes valida* Boheman, 1858: 90.

***Eleodes hispilabris* (Say, 1824)** CAN (AB MB SK) USA (AZ CA CO ID KS MT ND NE NM NV OK OR SD TX UT WA WY) MEX (CH CO NL SO TA)

*Blaps hispilabris* Say, 1824a: 259.

*Eleodes sulcata* LeConte, 1852: 67 [junior primary homonym of *Eleodes sulcatus* Eschscholtz, 1829]. Synonymy: Horn (1870: 313).

*Eleodes connexa* LeConte, 1857: 49. Synonymy: Triplehorn et al. (2015: 174).

*Eleodes nupta* LeConte, 1858c: 183. Synonymy: Triplehorn et al. (2015: 174).

*Eleodes binotata* Walker, 1866: 329. Synonymy: Triplehorn et al. (2015: 174).

*Elaeodes lecontei* Gemminger, 1870: 122. Replacement name for *Elaeodes sulcata* LeConte, 1852.

Eleodes hispilabris forma sculptilis Blaisdell, 1909: 220. Synonymy: Triplehorn et al. (2015: 174).

Eleodes hispilabris forma elongata Blaisdell, 1909: 220 [primary homonym of Eleodes dentipes forma elongata Blaisdell, 1909]. Synonymy: Triplehorn et al. (2015: 174).

Eleodes hispilabris forma laevis Blaisdell, 1909: 220. Synonymy (with *E. binotata* Walker): Blair (1921: 283).

*Eleodes subpinguis* Blaisdell, 1909: 247. Synonymy: Triplehorn et al. (2015: 174).

Eleodes hispilabris var. imitabilis Blaisdell, 1918b: 167. Synonymy: Triplehorn et al. (2015: 174).

Eleodes hispilabris var. attenuata Blaisdell, 1918b: 168 [junior secondary homonym of *Eleodes attenuatus* (LeConte, 1851)]. Synonymy: Triplehorn et al. (2015: 174).

*Eleodes hispilabris immundus* Blaisdell, 1925a: 79. Replacement name for *Eleodes hispilabris elongata* Blaisdell, 1909.

***Eleodes loretensis* Blaisdell, 1923** MEX (BC BS)

*Eleodes loretensis* Blaisdell, 1923: 262.

***Eleodes mexicana* Blaisdell, 1943** MEX (BC BS)

*Eleodes mexicana* Blaisdell, 1943: 246.

*Eleodes simondsi* Blaisdell, 1943: 247. Synonymy: Triplehorn (1996: 11).

*Eleodes blaisdelli* Blackwelder, 1945: 521. Unnecessary replacement name for *Eleodes mexicana* Blaisdell, 1943.

***Eleodes mirabilis* Triplehorn, 2007** USA (TX) MEX (NL SL TA)

*Eleodes mirabilis* Triplehorn, 2007: 634.

***Eleodes moesta* Blaisdell, 1921** MEX (BC BS)

*Eleodes sanmartinensis moesta* Blaisdell, 1921b: 221.

*Eleodes morbosa* Blaisdell, 1925b: 335. Synonymy: Triplehorn (1996: 11).

***Eleodes muricatula* Triplehorn, 2007** MEX (CO SL ZA)

*Eleodes muricatulus* Triplehorn, 2007: 637.

***Eleodes obscura dispersa* LeConte, 1858** USA (AZ CO NM UT)

*Eleodes dispersa* LeConte, 1858c: 182.

*Eleodes deleta* LeConte, 1858c: 182. Synonymy: Horn (1870: 305).

***Eleodes obscura glabriuscula* Blaisdell, 1925** USA (AZ NM TX) MEX (CH)

*Eleodes obscura glabriuscula* Blaisdell, 1925c: 383.

***Eleodes obscura obscura* (Say, 1824)** USA (CO MT NE NM TX WY)

*Blaps obscura* Say, 1824a: 259.

**Figure 17.**
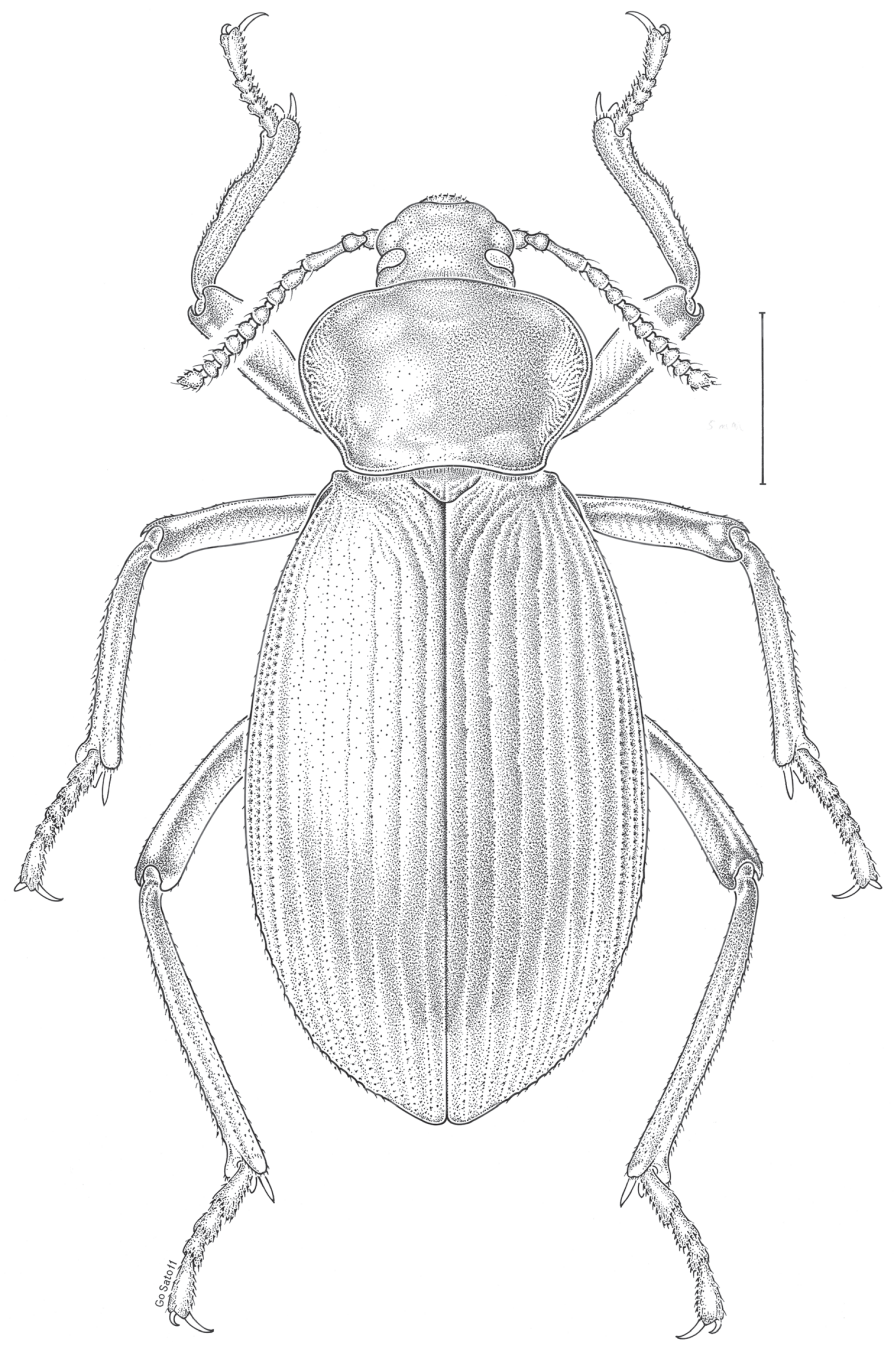
Eleodes (Eleodes) obscura sulcipennis Mannerheim, 1843. Scale bar = 1 mm.

***Eleodes obscura sulcipennis* Mannerheim, 1843** [Fig. 17] CAN (BC) USA (AZ CA ID MT NM NV OR UT WA) MEX (CH DU SO)

*Eleodes sulcipennis* Mannerheim, 1843: 266.

*Eleodes arata* LeConte, 1858c: 182. Synonymy: Horn (1870: 306).

*Eleodes conjuncta* Walker, 1866: 329. Synonymy: Leng (1920: 227).

*Eleodes convexicollis* Walker, 1866: 328. Synonymy: Leng (1920: 227).

***Eleodes rossi* Blaisdell, 1943** MEX (BS)

*Eleodes rossi* Blaisdell, 1943: 241.

***Eleodes rugosa* Perbosc, 1839** MEX (SL TA VE)

*Eleodes rugosa* Perbosc, 1839: 263.

*Eleodes caudata* Solier, 1848: 255. Synonymy: Champion (1884: 77).

***Eleodes samalayucae* Triplehorn, 2007** MEX (CH)

*Eleodes samalayucae* Triplehorn, 2007: 641.

***Eleodes sanmartinensis* Blaisdell, 1921** MEX (BC)

*Eleodes sanmartinensis* Blaisdell, 1921b: 220.

***Eleodes scyroptera* Triplehorn, 2007** MEX (AG DU GU HI NL QU ZA)

*Eleodes scyropterus* Triplehorn, 2007: 635.

***Eleodes spinipes macrura* Champion, 1892** USA (AZ NM TX) MEX (AG CH CO DU JA NA SO ZA)

*Elaeodes macrura* Champion, 1892: 511.

Eleodes ventricosa var. falli Blaisdell, 1909: 305. Synonymy: Triplehorn (2010: 377).

***Eleodes spinipes spinipes* Solier, 1848** MEX (GU HI NL QU SL TA)

*Eleodes spinipes* Solier, 1848: 253.

***Eleodes spinipes ventricosa* LeConte, 1858** USA (TX) MEX (CO NL TA)

*Eleodes ventricosa* LeConte, 1858c: 186.

***Eleodes sponsa* LeConte, 1858** USA (AZ CO NM TX UT)

*Eleodes sponsa* LeConte, 1858c: 184.

Eleodes sponsa forma convexa Blaisdell, 1909: 215. Synonymy: Triplehorn et al. (2015: 187).

***Eleodes subcylindrica* Casey, 1890** USA (AZ CA NV) MEX (BC)

*Eleodes subcylindricus* Casey, 1890b: 400.

Eleodes armata forma subedentata Blaisdell, 1909: 262. Synonymy: Blaisdell (1910: 62).

***Eleodes suturalis* (Say, 1824)** USA (AZ CO KS MN ND NE NM OK SD TX UT WY)

*Blaps suturalis* Say, 1824a: 257.

*Eleodes texana* LeConte, 1858c: 182. Synonymy: Triplehorn et al. (2009: 432).

***Eleodes tenuipes* Casey, 1890** USA (NM TX) MEX (CH)

*Eleodes tenuipes* Casey, 1890b: 399.

***Eleodes vanduzeei* Blaisdell, 1923** MEX (BS)

*Eleodes vanduzeei* Blaisdell, 1923: 264.

**Subgenus Heteropromus Blaisdell, 1909**

*Heteropromus* Blaisdell, 1909: 179. Type species: *Eleodes veterator* Horn, 1874, monotypy.

***Eleodes veterator* Horn, 1874** ^33^ USA (LA TX)

*Eleodes vetorator* Horn, 1874a: 33.

**Subgenus Litheleodes Blaisdell, 1909**

*Litheleodes* Blaisdell, 1909: 114. Type species: *Blaps extricata* Say, 1824, subsequent designation (Triplehorn and Thomas 2015: 11).

***Eleodes arcuata* Casey, 1884** USA (AZ NM TX) MEX (CH CO SO)

*Eleodes arcuata* Casey, 1884 [August]: 47.

*Elaeodes sonorae* Champion, 1884 [December]: 85. Synonymy: Gebien (1938: 53).

***Eleodes aspera* LeConte, 1866** USA (AZ CO UT)

*Eleodes aspera* LeConte, 1866b: 115.

***Eleodes corvina* Blaisdell, 1921** USA (CA OR)

*Eleodes corvina* Blaisdell, 1921b: 224.

**Figure 18.**
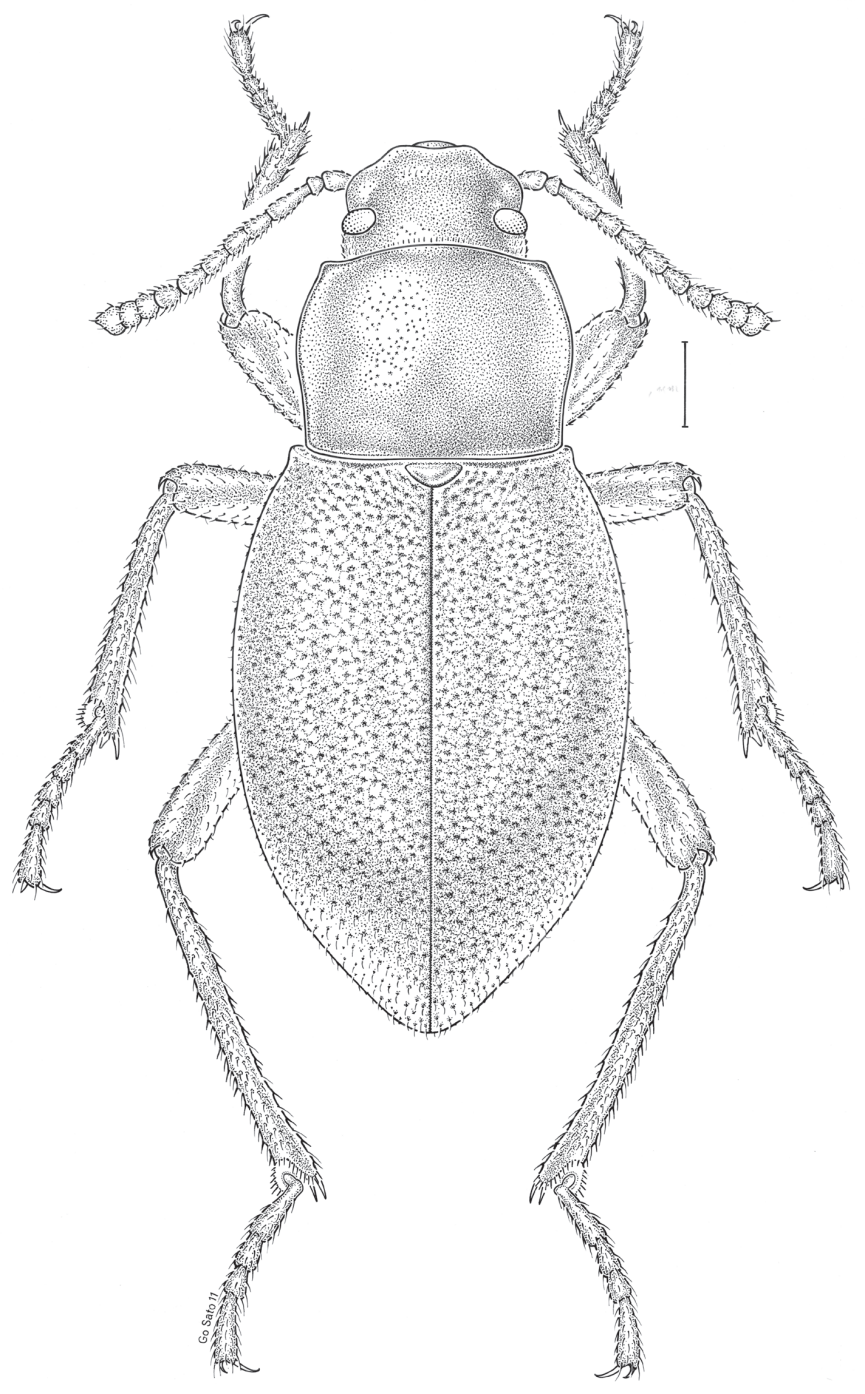
Eleodes (Litheleodes) extricata (Say, 1824). Scale bar = 1 mm.

***Eleodes extricata* (Say, 1824)** [Fig. 18] CAN (AB BC SK) USA (AZ CO ID KS MT ND NE NM NV OK OR SD TX UT WY) MEX (CH CO SO)

*Blaps extricata* Say, 1824a: 261.

*Eleodes cognata* Haldeman, 1852: 376. Synonymy: Triplehorn and Thomas (2015: 16).

Eleodes extricata var. arizonensis Blaisdell, 1909: 125. Synonymy: Triplehorn and Thomas (2015: 16).

Eleodes extricata forma elongata Blaisdell, 1909: 123. Synonymy: Triplehorn and Thomas (2015: 16).

Eleodes extricata forma convexicollis Blaisdell, 1909: 123 [junior primary homonym of *Eleodes convexicollis* Walker, 1866]. Synonymy: Triplehorn and Thomas (2015: 16).

Eleodes extricata var. utahensis Blaisdell, 1921a: 131. Synonymy: Triplehorn and Thomas (2015: 16).

*Eleodes extricata frigida* La Rivers, 1943b: 54. Synonymy: Triplehorn and Thomas (2015: 16).

*Eleodes extricatus convexinotus* Thomas, 2005: 551. Replacement name for *Eleodes extricatus convexicollis* Blaisdell, 1909.

***Eleodes granulata* LeConte, 1857** CAN (BC) USA (CA CO ID NM OR WA)

*Eleodes subaspera* Solier, 1848: 246 [*nomen dubium*, see Horn (1870: 309)].

*Eleodes granulata* LeConte, 1857: 50. Synonymy (in doubt): Horn (1870: 309).

*Eleodes obtusa* LeConte, 1861b: 352. Synonymy: Horn (1870: 309).

Eleodes letcheri var. vandykei Blaisdell, 1909: 136. Synonymy: Triplehorn and Thomas (2015: 18).

Eleodes vandykei var. modificata Blaisdell, 1921a: 131. Synonymy: Triplehorn and Thomas (2015: 18).

*Eleodes vandykei similis* Blaisdell, 1942: 142. Synonymy: Triplehorn and Thomas (2015: 18).

***Eleodes hirtipennis* Triplehorn, 1964** USA (CO)

*Eleodes hirtipennis* Triplehorn, 1964b: 60.

***Eleodes letcheri* Blaisdell, 1909** USA (ID NV OR UT)

*Eleodes letcheri* Blaisdell, 1909: 133.

***Eleodes papillosa* Blaisdell, 1917** USA (CA)

Eleodes granulata forma tuberculata Blaisdell, 1909: 131 [junior primary homonym of *Eleodes tuberculata* Eschscholtz, 1829].

*Eleodes papillosa* Blaisdell, 1917: 226. Replacement name for *Eleodes tuberculata* Blaisdell, 1909.

***Eleodes subtuberculata* Walker, 1866** CAN (BC) USA (CA ID MT OR WA)

*Eleodes subtuberculata* Walker, 1866: 328.

**Subgenus Melaneleodes Blaisdell, 1909**

*Melaneleodes* Blaisdell, 1909: 36. Type species: *Blaps carbonaria* Say, 1824, subsequent designation (Triplehorn and Thomas 2012: 254).

***Eleodes anthracina anthracina* Blaisdell, 1909** USA (AZ NM) MEX (CH SO)

Eleodes quadricollis var. anthracina Blaisdell, 1909: 87.

***Eleodes anthracina lustrans* Blaisdell, 1909** USA (AZ)

Eleodes quadricollis var. lustrans Blaisdell, 1909: 89.

***Eleodes carbonaria carbonaria* (Say, 1824)** USA (AZ CO NM NV TX UT WY) MEX (CH CO DU JA NL SL SO TA ZA)

*Blaps carbonaria* Say, 1824a: 260.

*Eleodes vicina* LeConte, 1851: 133. Synonymy: Triplehorn and Thomas (2012: 256).

*Eleodes immunis* LeConte, 1858c: 186. Synonymy: Horn (1870: 308).

*Eleodes porcatus* Casey, 1890b: 396. Synonymy: Triplehorn and Thomas (2012: 257).

Eleodes carbonaria forma interstitialis Blaisdell, 1909: 47. Synonymy: Triplehorn and Thomas (2012: 257).

Eleodes carbonaria forma glabra Blaisdell, 1909: 47. **New synonymy** [ADS & MAJ].

*Eleodes mazatzalensis* Blaisdell, 1925c: 379. Synonymy: Triplehorn and Thomas (2012: 257).

***Eleodes carbonaria chihuahuensis* Champion, 1884** USA (AZ NM) MEX (CH CO DU SO)

*Elaeodes chihuahuensis* Champion, 1884: 86.

*Eleodes nitidus* Casey, 1891: 58. Synonymy: Triplehorn and Thomas (2012: 270).

*Eleodes ampla* Blaisdell, 1909: 53. Synonymy: Triplehorn and Thomas (2012: 270).

Eleodes ampla var. dolosa Blaisdell, 1909: 57. Synonymy (with *E. nitidus* Casey): Blaisdell (1910: 65).

*Eleodes lineata* Blaisdell, 1939b: 55. Synonymy: Triplehorn and Thomas (2012: 270).

***Eleodes carbonaria disjuncta* Triplehorn and Thomas, 2012** MEX (GU HI ME ZA)

*Eleodes carbonarius disjunctus* Triplehorn and Thomas, 2012: 269.

***Eleodes carbonaria knausii* Blaisdell, 1909** USA (CO NM)

*Eleodes knausii* Blaisdell, 1909: 67.

***Eleodes carbonaria nuevoleonensis* Triplehorn and Thomas, 2012** MEX (CO NL)

*Eleodes carbonarius nuevoleonensis* Triplehorn and Thomas, 2012: 265.

***Eleodes carbonaria obsoleta* (Say, 1824)** CAN (AB MB SK) USA (AZ CO KS MT ND NE NM OK SD TX UT WY) MEX (SO)

*Blaps obsoleta* Say, 1824a: 261.

Eleodes obsoleta forma glabra Blaisdell, 1909: 60. Synonymy: Triplehorn and Thomas (2012: 272).

Eleodes obsoleta forma annectans Blaisdell, 1909: 60. Synonymy: Triplehorn and Thomas (2012: 272).

Eleodes obsoleta forma punctata Blaisdell, 1909: 60. Synonymy: Triplehorn and Thomas (2012: 272).

***Eleodes carbonaria omissoides* Blaisdell, 1935** MEX (DU NA NL SI SO ZA)

*Eleodes omissoides* Blaisdell, 1935c: 157.

***Eleodes carbonaria omissa* LeConte, 1858** USA (CA NV) MEX (BC)

*Eleodes omissa* LeConte, 1858c: 186.

*Eleodes interrupta* Blaisdell, 1892: 241. Synonymy: Blaisdell (1909: 72).

Eleodes omissa forma catalinae Blaisdell, 1909: 73. Synonymy: Triplehorn and Thomas (2012: 258).

Eleodes omissa forma communis Blaisdell, 1909: 73. Synonymy: Triplehorn and Thomas (2012: 258).

Eleodes omissa forma emarginata Blaisdell, 1909: 74. Synonymy: Triplehorn and Thomas (2012: 258).

Eleodes omissa var. pygmaea Blaisdell, 1909: 77. Synonymy: Triplehorn (1996: 5).

Eleodes omissa var. peninsularis Blaisdell, 1909: 79. Synonymy: Triplehorn (1996: 5).

*Eleodes omissa tumida* Blaisdell, 1933b: 194. Synonymy: Triplehorn (1996: 5).

***Eleodes carbonaria soror* LeConte, 1858** USA (TX) MEX (CO NL TA)

*Eleodes soror* LeConte, 1858c: 185.

***Eleodes halli* Blaisdell, 1941** USA (AZ UT)

*Eleodes fuscipilosa* Blaisdell, 1925c: 376 [junior secondary homonym of *Eleodes fuscipilosus* (Casey, 1890)].

*Eleodes halli* Blaisdell, 1941a: 37. Synonymy: Triplehorn and Thomas (2012: 275).

***Eleodes humeralis* LeConte, 1857** CAN (BC) USA (CA ID OR UT WA)

*Eleodes humeralis* LeConte, 1857: 50.

*Eleodes latiuscula* Walker, 1866: 329. Synonymy: LeConte (1873: 334).

***Eleodes neomexicana* Blaisdell, 1909** USA (NM TX)

Eleodes pedinoides var. neomexicana Blaisdell, 1909: 113.

***Eleodes parowana* Blaisdell, 1925** USA (UT)

*Eleodes parowana* Blaisdell, 1925c: 374.

*Eleodes parowana mimica* Blaisdell, 1925c: 375. Synonymy: Triplehorn and Thomas (2012: 276).

***Eleodes pedinoides* LeConte, 1858** USA (NM TX) MEX (CO NL TA)

*Eleodes pedinoides* LeConte, 1858c: 183.

*Eleodes asperata* LeConte, 1858c: 183. Synonymy: Horn (1870: 307).

***Eleodes quadricollis* Eschscholtz, 1829** USA (CA)

*Eleodes quadricollis* Eschscholtz, 1829: 12.

*Eleodes tarsalis* Casey, 1890b: 399. Synonymy: Casey (1893: 597).

*Eleodes cuneaticollis* Casey, 1890b: 397. Synonymy: Triplehorn and Thomas (2012: 272).

***Eleodes rileyi reducta* Blaisdell, 1925** USA (UT)

*Eleodes reducta* Blaisdell, 1925c: 377.

***Eleodes rileyi rileyi* Casey, 1891** USA (AZ CA CO ID MT NM NV UT WY)

*Eleodes rileyi* Casey, 1891: 57.

Eleodes humeralis forma tuberculo-muricata Blaisdell, 1909: 97. Synonymy: Triplehorn and Thomas (2012: 274).

Eleodes humeralis forma granulato-muricata Blaisdell, 1909: 97. Synonymy: Triplehorn and Thomas (2012: 274).

*Eleodes quadricollis lassenica* Blaisdell, 1925c: 373. Synonymy: Triplehorn and Thomas (2012: 274).

*Eleodes coloradensis* Blaisdell, 1925c: 380. Synonymy: Triplehorn and Thomas (2012: 274).

*Eleodes concinna* Blaisdell, 1925c: 381. Synonymy: Triplehorn and Thomas (2012: 274).

*Eleodes tanneri* Blaisdell, 1932a: 74. Synonymy: Triplehorn and Thomas (2012: 274).

***Eleodes rufipes rufipes* Pierre, 1976** MEX (PU/VE [Pico de Orizaba])

*Eleodes alticola rufipes* Pierre, 1976: 708.

***Eleodes rufipes transvolcanensis* Thomas, 2005** MEX (ME/MO/PU [Popocatépetl])

*Eleodes alticola* Pierre, 1976: 706 [junior primary homonym of *Eleodes alticola* Blaisdell, 1925].

*Eleodes transvolcanensis* Thomas, 2005: 553^34^. Replacement name for *Eleodes alticola* Pierre, 1976.

**Figure 19.**
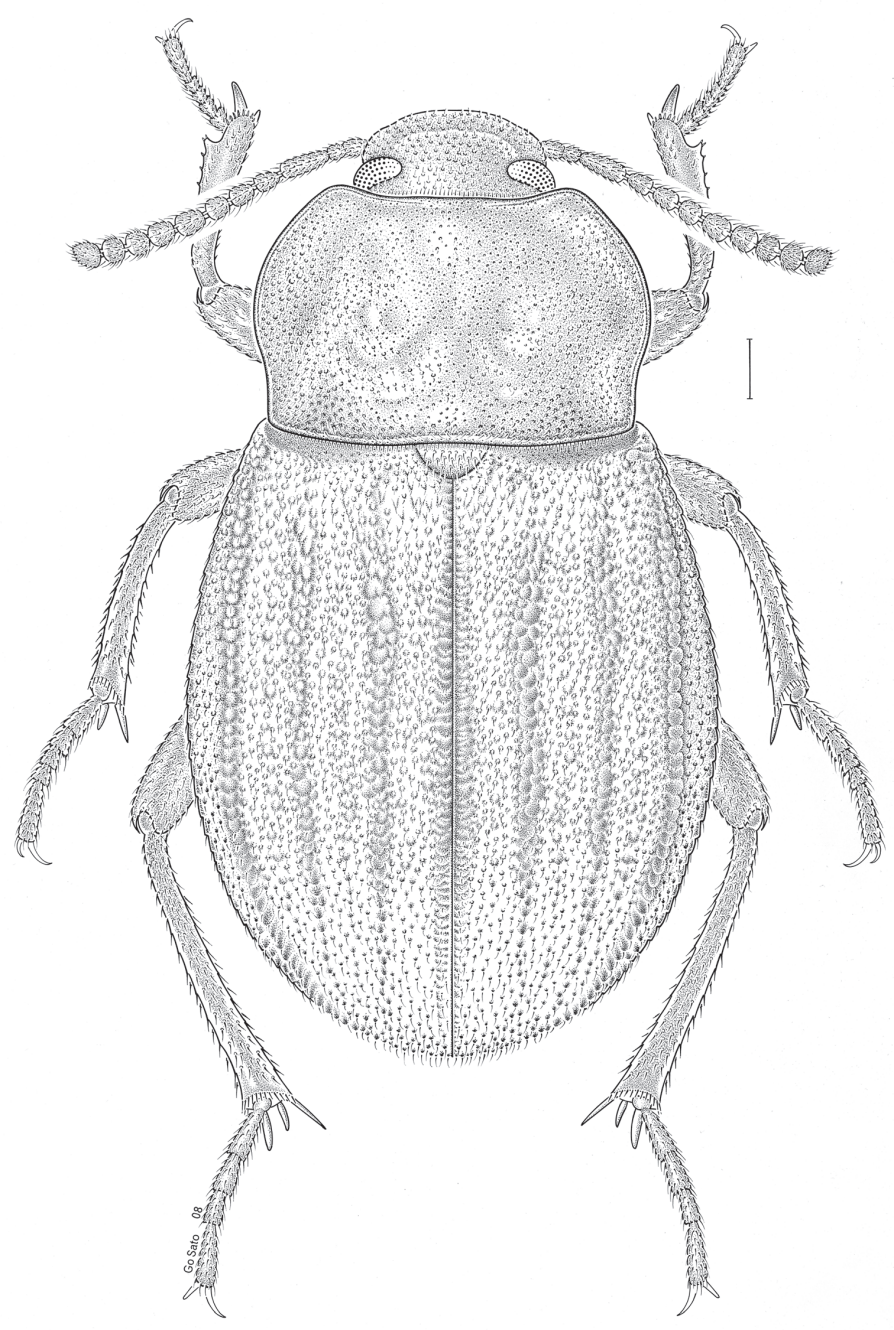
Eleodes (Melaneleodes) tricostata (Say, 1824). Scale bar = 1 mm.

***Eleodes tricostata* (Say, 1824)** [Fig. 19] CAN (AB MB SK) USA (AZ CO IA KS MN MO MT ND NE NM OK SD TX WI WY) MEX (CO TA)

*Blaps tricostata* Say, 1824a: 262.

*Pimelia alternata* Kirby, 1837: 232. Synonymy: LeConte (1851: 133).

*Eleodes planata* Solier, 1848: 366 [junior primary homonym of *Eleodes planata* Eschscholtz, 1829]. Synonymy: LeConte (1866a: 60).

*Eleodes robusta* LeConte, 1858c: 183. Synonymy: Horn (1870: 307).

Eleodes tricostata forma ovalis Blaisdell, 1909: 106. Synonymy: Triplehorn and Thomas (2012: 274).

Eleodes tricostata forma costata Blaisdell, 1909: 106. Synonymy: Triplehorn and Thomas (2012: 274).

***Eleodes wenzeli speculicollis* Blaisdell, 1925** USA (TX)

*Eleodes speculicollis* Blaisdell, 1925c: 382.

***Eleodes wenzeli wenzeli* Blaisdell, 1925** USA (NM TX)

*Eleodes wenzeli* Blaisdell, 1925c: 381.

**Subgenus Metablapylis Blaisdell, 1909**

*Metablapylis* Blaisdell, 1909: 391. Type species: *Eleodes nigrina* LeConte, 1858, **present designation**.

***Eleodes aalbui* Triplehorn, 2007** USA (CA)

*Eleodes aalbui* Triplehorn, 2007: 628.

***Eleodes californica* Blaisdell, 1929** USA (CA)

*Eleodes californica* Blaisdell, 1929a: 165.

***Eleodes delicata* Blaisdell, 1929** USA (AZ TX UT) MEX (BC)

*Eleodes delicata* Blaisdell, 1929a: 164.

***Eleodes dissimilis* Blaisdell, 1909** USA (AZ CA NM NV TX UT) MEX (SO)

*Eleodes dissimilis* Blaisdell, 1909: 398.

***Eleodes nevadensis* Blaisdell, 1909** USA (AZ CA NV UT)

Eleodes dissimilis var. nevadensis Blaisdell, 1909: 402.

***Eleodes nigrina difformis* Blaisdell, 1925** USA (ID OR WA)

*Eleodes nigrina difformis* Blaisdell, 1925c: 389.

***Eleodes nigrina maclayi* Boddy, 1957** USA (OR)

*Eleodes nigrina maclayi* Boddy, 1957: 197.

**Figure 20.**
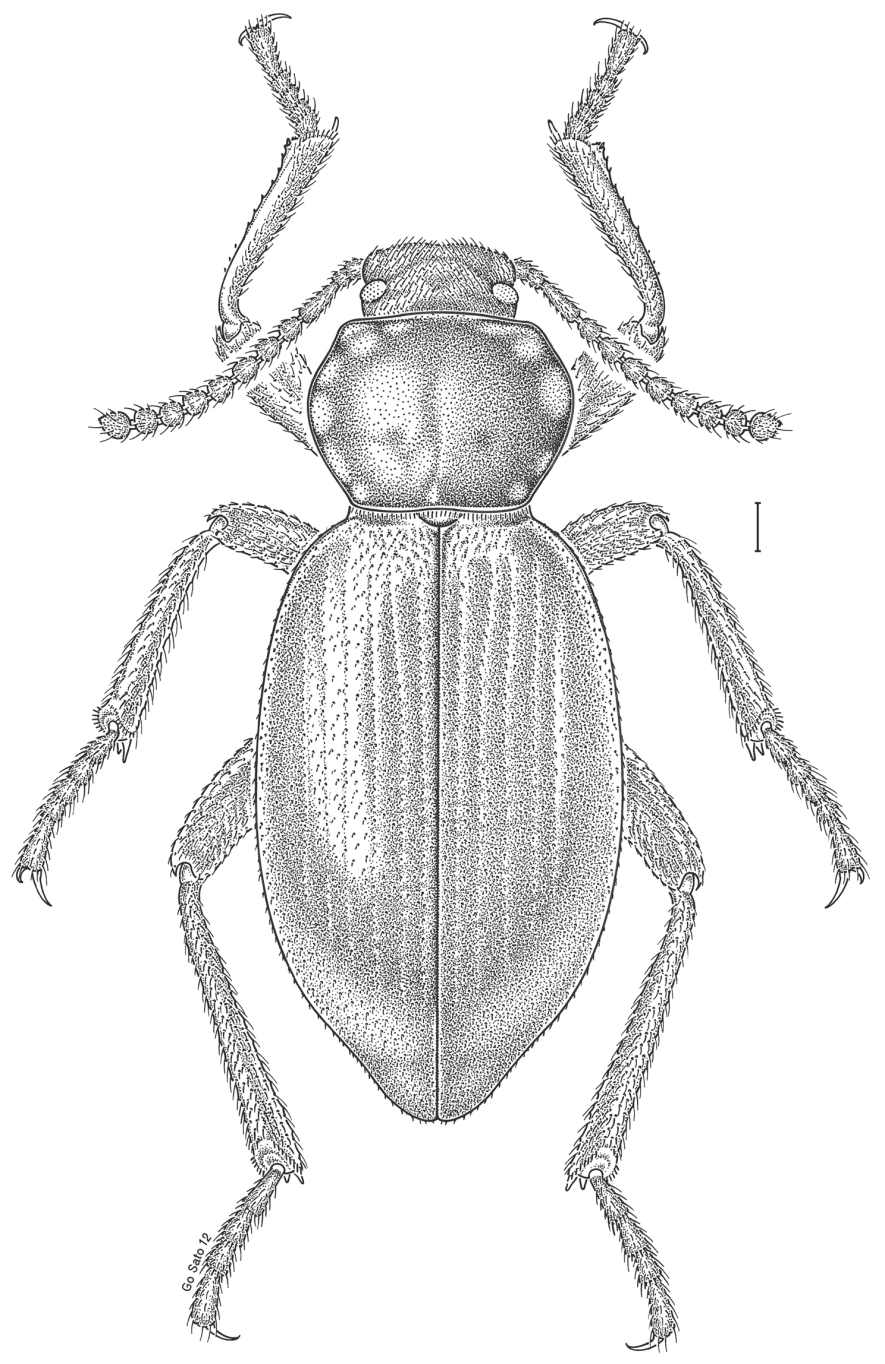
Eleodes (Metablapylis) nigrina nigrina LeConte, 1858. Scale bar = 1 mm.

***Eleodes nigrina nigrina* LeConte, 1858** [Fig. 20] CAN (BC) USA (AZ CA CO ID KS ND NE NM NV OR TX UT)

*Eleodes nigrina* LeConte, 1858c: 186.

***Eleodes nigrina perlonga* Blaisdell, 1909** USA (ID WY)

Eleodes nigrina var. perlonga Blaisdell, 1909: 398.

**Subgenus Omegeleodes Triplehorn and Thomas, 2012**

*Omegeleodes* Triplehorn and Thomas, 2012: 253. Type species: *Eleodes debilis* LeConte, 1858, original designation.

***Eleodes debilis* LeC4onte, 1858** USA (AZ NM TX) MEX (AG CH CO DU MI NL QU SI SL SO ZA)

*Eleodes debilis* LeConte, 1858c: 185.

**Subgenus Promus LeConte, 1862**

*Promus* LeConte, 1862a: 226. Type species: *Blaps opaca* Say, 1824, original designation.

***Eleodes anachronus* Triplehorn, 2010** ^35^ MEX (HI JA OA QU SL TA VE)

*Eleodes anachronus* Triplehorn, 2010: 373.

***Eleodes bidens* Triplehorn, 2007** MEX (DU)

*Eleodes bidens* Triplehorn, 2007: 641.

***Eleodes brucei* Triplehorn, 2007** MEX (DU ZA)

*Eleodes brucei* Triplehorn, 2007: 638.

***Eleodes calcarata* Champion, 1884** MEX (GU)

*Elaeodes calcarata* Champion, 1884: 86.

***Eleodes composita* Casey, 1891** USA (TX)

*Eleodes compositus* Casey, 1891: 58.

***Eleodes erratica* Champion, 1884** MEX (NA SI)

*Elaeodes erratica* Champion, 1884: 87.

***Eleodes exarata* Champion, 1884** MEX (SL)

*Elaeodes exarata* Champion, 1884: 78.

***Eleodes fusiformis* LeConte, 1858** USA (CO KS NE NM TX WY)

*Eleodes fusiformis* LeConte, 1858c: 184.

***Eleodes goryi* Solier, 1848** USA (NM TX) MEX (PU TA VE)

*Eleodes goryi* Solier, 1848: 251.

*Eleodes seriata* LeConte, 1858c: 185. Synonymy: Champion (1885: 93).

***Eleodes hoegei* Champion, 1885** MEX (PU VE)

*Elaeodes högei* Champion, 1885: 91.

***Eleodes insularis* Linell, 1899** MEX (BC BS)

*Eleodes insularis* Linell, 1899: 181.

*Eleodes terricola* Blaisdell, 1910: 61.^36^ Synonymy: Triplehorn (1971: 58).

***Eleodes knullorum* Triplehorn, 1971** USA (AZ NM TX) MEX (CO HI)

*Eleodes knullorum* Triplehorn, 1971: 56.

***Eleodes longicornis* Champion, 1884** MEX (DU)

*Elaeodes longicornis* Champion, 1884: 87.

***Eleodes madrensis* Johnston, 2015** USA (AZ NM) MEX (SO)

*Eleodes madrensis* Johnston, 2015: 14.

***Eleodes montana* Champion, 1884** MEX (SL)

*Elaeodes montana* Champion, 1884: 86.

**Figure 21.**
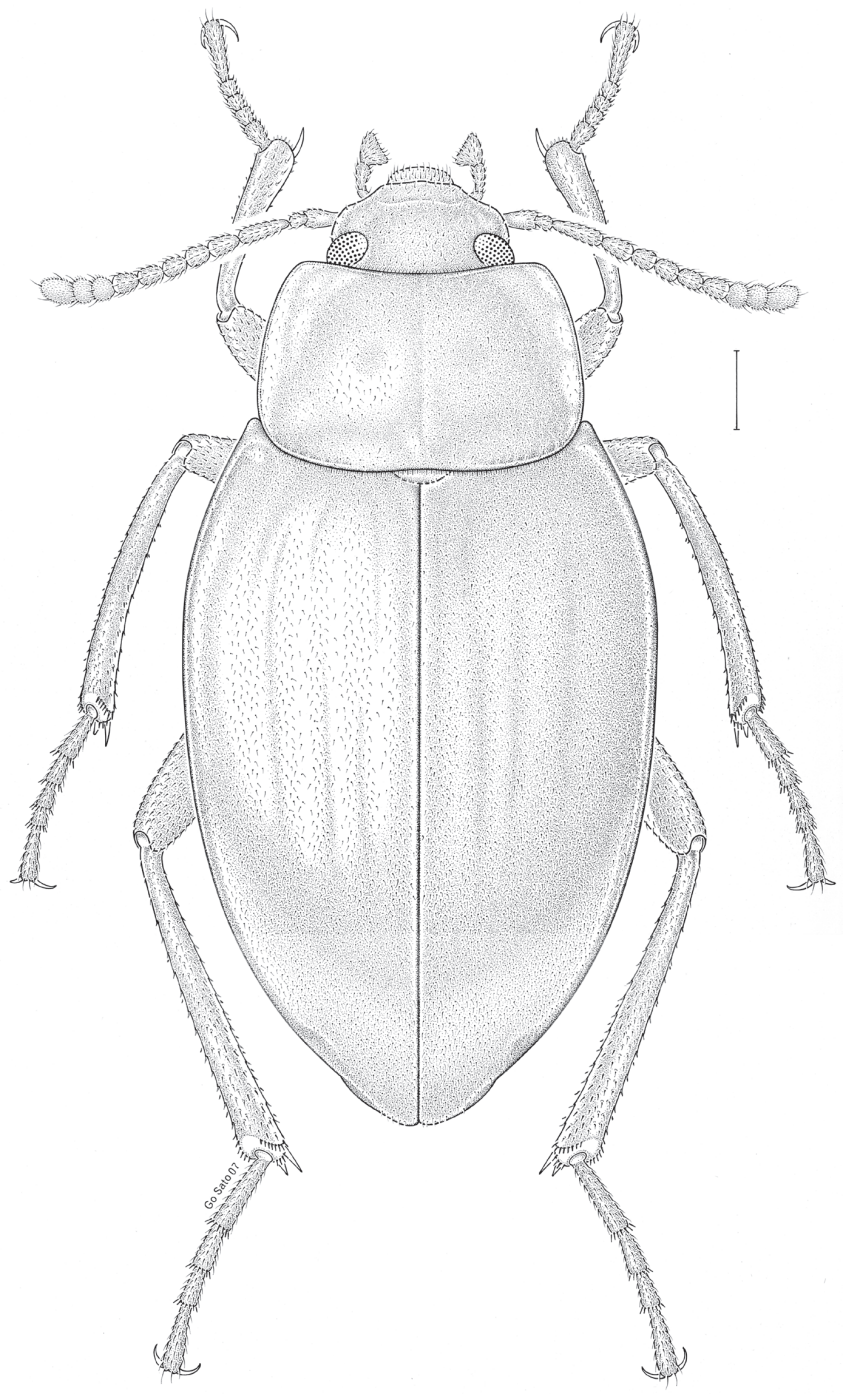
Eleodes (Promus) opaca (Say, 1824). Scale bar = 1 mm.

***Eleodes opaca* (Say, 1824)** [Fig. 21] CAN (AB MB SK) USA (CO KS ND NE OK SD TX)

*Blaps opaca* Say, 1824a: 263.

***Eleodes spiculifera* Triplehorn, 2007** USA (TX)

*Eleodes spiculiferus* Triplehorn, 2007: 632.

***Eleodes spinolae* Solier, 1848** MEX (CO FD GE GU HI ME MO OA PU VE)

*Eleodes spinolae* Solier, 1848: 253.

***Eleodes striolata* LeConte, 1858** USA (TX) MEX (CO)

*Eleodes striolata* LeConte, 1858c: 185.

***Eleodes subnitens* LeConte, 1851** USA (AZ) MEX (SO)

*Eleodes subnitens* LeConte, 1851: 134.

Eleodes subnitens forma sinuata Blaisdell, 1909: 163. Synonymy: Johnston (2015: 14).

***Eleodes watrousi* Triplehorn, 2007** MEX (DU)

*Eleodes watrousi* Triplehorn, 2007: 640.

**Subgenus Pseudeleodes Blaisdell, 1909**

*Pseudeleodes* Blaisdell, 1909: 146. Type species: *Eleodes granosa* LeConte, 1866, monotypy.

*Trichoderulus* Blaisdell, 1923: 281. Type species: *Trichoderulus longipilosus* Blaisdell, 1923 (= *Eleodes tribulus* Thomas, 2005), original designation. Synonymy: Johnston (2016: 672).

***Eleodes caudifera* LeConte, 1858** USA (AZ CO NM TX UT) MEX (CH)

*Eleodes caudifera* LeConte, 1858c: 184.

Eleodes caudifera forma glabra Blaisdell, 1909: 228. Synonymy: Johnston (2016: 671).

Eleodes caudifera forma scabra Blaisdell, 1909: 228. Synonymy: Johnston (2016: 671).

Eleodes caudifera forma sublaevis Blaisdell, 1909: 228. Synonymy: Johnston (2016: 671).

***Eleodes granosa* LeConte, 1866** USA (CA NV OR)

*Eleodes granosa* LeConte, 1866b: 116.

Eleodes granosa forma fortis Blaisdell, 1909: 150. **New synonymy** [YB].

Eleodes granosa var. pilifera Boddy, 1957: 193. Synonymy: Johnston (2016: 671).

*Eleodes inyoensis* Tanner, 1961: 68. Synonymy: Johnston (2016: 671).

***Eleodes inornata* Johnston, 2016** USA (NV)

*Eleodes inornatus* Johnston, 2016: 669.

***Eleodes leechi* Tanner, 1961** USA (CO UT)

*Eleodes leechi* Tanner, 1961: 63.

***Eleodes longipilosa* Horn, 1891** USA (CA ID NV OR)

*Eleodes longipilosa* Horn, 1891: 42.

***Eleodes pilosa* Horn, 1870** USA (CA ID NM NV OR UT WA WY)

*Eleodes pilosa* Horn, 1870: 314.

Eleodes pilosa forma ordinata Blaisdell, 1909: 143. **New synonymy** [YB].

*Eleodes obesus* Doyen, 1985b: 232. Synonymy: Johnston (2016: 673).

***Eleodes spoliata* Blaisdell, 1933** USA (OR)

*Eleodes spoliata* Blaisdell, 1933b: 196.

***Eleodes tribulus* Thomas, 2005** USA (AZ) MEX (SO)

*Amphidora caudata* Horn, 1870: 330 [junior secondary homonym of *Eleodes caudata* Solier, 1848].

*Trichoderulus longipilosus* Blaisdell, 1923: 281 [junior secondary homonym of *Eleodes longipilosus* Horn, 1891]. Synonymy: Triplehorn and Aalbu (1987: 371).

*Eleodes blaisdelli* Doyen [in Doyen and Lawrence], 1979: 367 [junior primary homonym of *Eleodes blaisdelli* Blackwelder, 1945]. Replacement name for *Eleodes longipilosus* Blaisdell, 1923.

*Eleodes tribulus* Thomas, 2005: 552. Replacement name for *Eleodes caudatus* (Horn, 1870).

**Subgenus Tricheleodes Blaisdell, 1909**

*Tricheleodes* Blaisdell, 1909: 138. Type species: *Eleodes hirsuta* LeConte, 1861, by subsequent designation (Johnston 2016: 666).

***Eleodes hirsuta* LeConte, 1861** USA (CA NV UT)

*Eleodes hirsuta* LeConte, 1861b: 352.

**Subgenus Xysta Eschscholtz, 1829**

*Xysta* Eschscholtz, 1829: 9. Type species: *Eleodes gravida* Eschscholtz, 1829, subsequent designation (Hope 1841: 124). **Status revised** [ADS & MAJ].

*Steneleodes* Blaisdell, 1909: 409. Type species: *Eleodes longicollis* LeConte, 1851, **present designation**. **New synonymy** [ADS & MAJ].

*Holeleodes* Blaisdell, 1937b: 132. Type species: *Eleodes beameri* Blaisdell, 1937 (=*Elaeodes hepburni* Champion, 1884), original designation. Synonymy (with *Steneleodes* Blaisdell): Johnston (2015: 12).

***Eleodes angulata* (Eschscholtz, 1829)** MEX (FD ME)

*Xysta angulata* Eschscholtz, 1829: 9.

***Eleodes angusta* Eschscholtz, 1829** MEX (DU FD GU HI JA ME MI OA PU VE)

*Eleodes angusta* Eschscholtz, 1829: 13.

***Eleodes blapoides* Eschscholtz, 1829** MEX (OA)

*Eleodes blapoides* Eschscholtz, 1829: 12.

*Elaeodes blaptoides* Champion, 1884: 78. Unjustified emendation of *Eleodes blapoides* Eschscholtz, 1829, not in prevailing usage.

***Eleodes coarctata* Champion, 1885** MEX (ME PU TA)

*Elaeodes coarctata* Champion, 1885: 91.

***Eleodes corrugans* Triplehorn, 2007** MEX (MI)

*Eleodes corrugans* Triplehorn, 2007: 630.

***Eleodes distincta* Solier, 1848** MEX (HI OA PU QU SL TA VE)

*Eleodes distincta* Solier, 1848: 239.

***Eleodes forreri* Champion, 1884** MEX (CH DU)

*Elaeodes forreri* Champion, 1884: 88.

***Eleodes gigantea* Mannerheim, 1843** USA (CA) MEX (BC)

*Eleodes gigantea* Mannerheim, 1843: 267.

*Eleodes gentilis* LeConte, 1858c: 187. Synonymy: Triplehorn (1996: 15).

*Eleodes estriatus* Casey, 1890b: 398. Synonymy: Triplehorn (1996: 15).

Eleodes gigantea var. meridionalis Blaisdell, 1918c: 387. Synonymy: Triplehorn (1996: 15).

***Eleodes glabricollis* Champion, 1884** MEX (AG GU NL SL)

*Elaeodes glabricollis* Champion, 1884: 85.

***Eleodes gravida* (Eschscholtz, 1829)** MEX (OA)

*Xysta gravida* Eschscholtz, 1829: 9.

***Eleodes hepburni* Champion, 1884** USA (AZ NM) MEX (CH CO DU JA SI SO)

*Elaeodes hepburni* Champion, 1884: 88.

*Eleodes compressitarsis* Blaisdell, 1935c: 158. Synonymy: Triplehorn (2010: 376).

*Eleodes beameri* Blaisdell, 1937b: 132. Synonymy: Triplehorn (2010: 376).

*Eleodes bryanti* Blaisdell, 1937b: 134. Synonymy (with *E. beameri* Blaisdell): Triplehorn and Doyen (1972: 79).

*Eleodes palmerleensis* Blaisdell, 1937b: 136. Synonymy (with *E. beameri* Blaisdell): Triplehorn and Doyen (1972: 79).

***Eleodes innocens* LeConte, 1866** MEX (BS)

*Eleodes innocens* LeConte, 1866b: 114.

***Eleodes laevigata blapsoides* Solier, 1848** MEX

*Eleodes laevigata* var. *blapsoïdes* Solier, 1848: 244.

***Eleodes laevigata laevigata* Solier, 1848** MEX (ME OA PU VE) GUA

*Eleodes laevigata* Solier, 1848: 244.

***Eleodes longicollis* LeConte, 1851** USA (AZ CO KS NM NV OR TX UT WY) MEX (AG CH CO DU MI NL SL SO ZA)

*Eleodes longicollis* LeConte, 1851: 134.

*Eleodes haydenii* LeConte, 1858c: 186. Synonymy: Horn (1870: 311).

***Eleodes mutilata* Blaisdell, 1921** MEX (BS)

*Eleodes mutilata* Blaisdell, 1921b: 222.

***Eleodes olida* Champion, 1892** MEX (GE)

*Elaeodes olida* Champion, 1892: 516.

***Eleodes ornatipennis* Blaisdell, 1937** USA (NM) MEX (CH)

*Eleodes ornatipennis* Blaisdell, 1937b: 129.

***Eleodes peropaca* Champion, 1892** MEX (DU)

*Elaeodes peropaca* Champion, 1892: 517.

***Eleodes platypennis* Triplehorn, 2007** MEX (JA)

*Eleodes platypennis* Triplehorn, 2007: 637.

***Eleodes ponderosa* Champion, 1884** MEX (OA PU)

*Elaeodes ponderosa* Champion, 1884: 84.

***Eleodes punctigera* Blaisdell, 1935** MEX (DU)

*Eleodes punctigera* Blaisdell, 1935c: 157.

***Eleodes ruida* (Say, 1835)** MEX (MO PU VE)

*Blaps ruida* Say, 1835: 183.

*Eleodes coriacea* Solier, 1848: 249. Synonymy: Champion (1884: 84).

***Eleodes sallaei* Champion, 1885** MEX (GU JA OA PU QU SL VE)

*Elaeodes sallaei* Champion, 1885: 89.

***Eleodes solieri* Champion, 1885** MEX (CH CO GU OA PU SL VE)

*Blaps celsa* Say, 1835: 185 [*nomen dubium*].

*Elaeodes solieri* Champion, 1885: 89. Synonymy (in doubt): Champion (1885: 89).

***Eleodes stolida* Champion, 1885** MEX

*Elaeodes stolida* Champion, 1885: 92.

***Eleodes sulcatula* Champion, 1884** MEX (ME)

*Elaeodes sulcatula* Champion, 1884: 83.

***Eleodes tenebricosa* Gemminger, 1870** MEX (ME OA)

*Eleodes obscura* Solier, 1848: 245 [junior secondary homonym of *Eleodes obscurus* (Say, 1824)].

*Elaeodes tenebricosa* Gemminger, 1870: 122. Replacement name for *Elaeodes obscura* Solier, 1848.

***Eleodes tessellata* Champion, 1892** MEX (MI)

*Elaeodes tessellata* Champion, 1892: 517.

[incertae sedis]

***Eleodes aequalis* (Say, 1835)** MEX (DU ME OA PU)

*Blaps aequalis* Say, 1835: 185.

*Eleodes alutacea* Solier, 1848: 240. Synonymy (in doubt): Champion (1884: 80).

*Eleodes maillei* Solier, 1848: 247. Synonymy (with *E. alutacea* Solier): Champion (1884: 80).

***Eleodes amaura* Champion, 1892** MEX (GU HI OA PU TA)

*Elaeodes amaura* Champion, 1892: 514.^37^

***Eleodes barbata* Wickham, 1918** USA (AZ CO NM UT)

*Eleodes barbata* Wickham, 1918: 256.

***Eleodes brevicollis* Gemminger, 1870** MEX

*Eleodes obsoleta* Solier, 1848: 238 [junior secondary homonym of *Eleodes obsoleta* (Say, 1823)].

*Eleodes brevicollis* Gemminger [in Gemminger and Harold], 1870: 1868. Replacement name for *Eleodes obsoleta* Solier, 1848.

***Eleodes cylindrica* (Herbst, 1799)** “Nordamerika”

*Blaps cylindrica* Herbst, 1799: 185.

***Eleodes dilaticollis* Champion, 1884** MEX (GU HI ME MI PU)

*Elaeodes dilaticollis* Champion, 1884: 83.

***Eleodes ebenina* (Solier, 1848)** MEX

*Nycterinus ebeninus* Solier, 1848: 269.

***Eleodes elongatula* Eschscholtz, 1829** MEX

*Eleodes elongatula* Eschscholtz, 1829: 13.

***Eleodes impolita* (Say, 1835)** MEX (ME OA PU VE)

*Blaps impolita* Say, 1835: 183.

*Eleodes aubei* Solier, 1848: 245. Synonymy: Champion (1885: 90).

***Eleodes maura* (Say, 1835)** MEX (GU OA PU)

*Blaps maura* Say, 1835: 184.

***Eleodes melanaria* Eschscholtz, 1829** MEX

*Eleodes melanaria* Eschscholtz, 1829: 13.

***Eleodes obliterata* (Say, 1835)** MEX

*Blaps obliterata* Say, 1835: 184.

***Eleodes polita* Champion, 1892** MEX (ME MO)

*Elaeodes polita* Champion, 1892: 513.

***Eleodes rotundicollis* (Eschscholtz, 1829)** MEX (PU SL VE)

*Xysta rotundicollis* Eschscholtz, 1829: 9.

*Blaps parva* Say, 1835: 186. Synonymy (in doubt): Champion (1884: 82).

***Eleodes scapularis* Champion, 1884** MEX (GU ME)

*Elaeodes scapularis* Champion, 1884: 81.

***Eleodes segregata* Champion, 1892** MEX (CH DU GE MI)

*Elaeodes segregata* Champion, 1892: 513.

***Eleodes striata* (Guérin-Méneville, 1834)** MEX (TA)

*Xysta striata* Guérin-Méneville, 1834: 30.

***Eleodes sulcata* (Eschscholtz, 1829)** MEX (MO)

*Xysta sulcata* Eschscholtz, 1829: 9.

**Genus *Eleodimorpha* Blaisdell, 1909** [F]

*Eleodimorpha* Blaisdell, 1909: 477. Type species: *Eleodimorpha bolcan* Blaisdell, 1909, original designation.

***Eleodimorpha bolcan* Blaisdell, 1909** USA (CA)

*Eleodimorpha bolcan* Blaisdell, 1909: 479.

**Genus *Embaphion* Say, 1824** [N]

*Embaphion* Say, 1824a: 254. Type species: *Akis muricata* Say, 1824, monotypy.

***Embaphion contractum blaisdelli* Benedict, 1927** USA (NM)

*Embaphion contractum blaisdelli* Benedict, 1927: 46.

***Embaphion contractum contractum* Blaisdell, 1909** USA (NM)

*Embaphion contractum* Blaisdell, 1909: 460.

***Embaphion contusum contusum* LeConte, 1858** USA (AZ CO KS NM WY)

*Embaphion contusum* LeConte, 1858a: 20.

***Embaphion contusum grande* Blaisdell, 1909** USA (NM)

Embaphion contusum forma grandis Blaisdell, 1909: 471.

***Embaphion contusum laminatum* Casey, 1890** USA (TX)

*Embaphion laminatum* Casey, 1890b: 403.

***Embaphion depressum* (LeConte, 1851)** USA (CA)

*Eleodes depressa* LeConte, 1851: 136.

***Embaphion elongatum* Horn, 1870** USA (CA ID NV OR UT)

*Embaphion elongatum* Horn, 1870: 321.

***Embaphion glabrum* Blaisdell, 1909** USA (AZ NM UT)

*Embaphion glabrum* Blaisdell, 1909: 457.

***Embaphion mexicanum* Blaisdell, 1935** MEX (CH)

*Embaphion mexicanum* Blaisdell, 1935c: 160.

**Figure 22.**
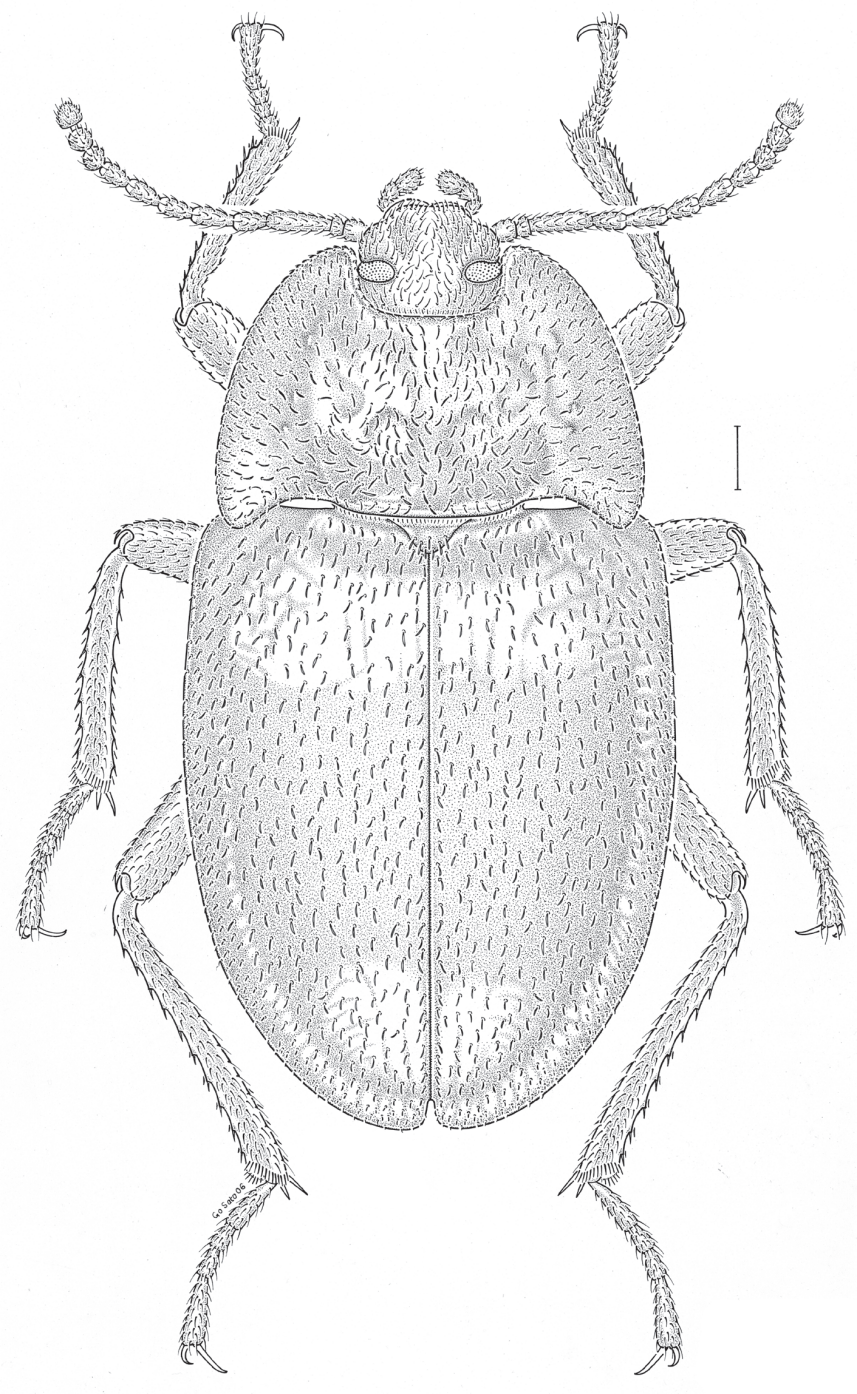
*Embaphion muricatum* (Say, 1824). Scale bar = 1 mm.

***Embaphion muricatum* (Say, 1824)** [Fig. 22] CAN (AB SK) USA (CO KS NE SD TX) MEX (TA)

*Akis muricata* Say, 1824a: 253.

*Embaphion concavum* LeConte, 1853: 446. Synonymy: Horn (1870: 320).

***Embaphion planum* Horn, 1870** USA (CO KS NM UT WY)

*Embaphion planum* Horn, 1870: 321.

**Genus *Lariversius* Blaisdell, 1947** [M]

*Lariversius* Blaisdell, 1947: 59. Type species: *Lariversius tibialis* Blaisdell, 1947, original designation.

***Lariversius tibialis* Blaisdell, 1947** USA (NV)

*Lariversius tibialis* Blaisdell, 1947: 61.

**Genus *Neobaphion* Blaisdell, 1925** [N]

*Neobaphion* Blaisdell, 1925c: 390. Type species: *Eleodes planipennis* LeConte, 1866, monotypy.

***Neobaphion alleni* Triplehorn, 1989** USA (ID OR)

*Neobaphion alleni* Triplehorn, 1989: 458.

***Neobaphion elongatum* Blaisdell, 1933** USA (CA NV)

*Neobaphion elongatum* Blaisdell, 1933b: 208.

***Neobaphion papula* Triplehorn and Aalbu, 1985** USA (NV)

*Neobaphion papula* Triplehorn and Aalbu, 1985 [11 July]: 588.

*Eleodes insolitus* Doyen, 1985b [11 July]: 230. Synonymy: Triplehorn (1989: 460).

***Neobaphion planipenne* (LeConte, 1866)** USA (AZ CO NM UT)

*Eleodes planipennis* LeConte, 1866b: 116.

**Genus *Trogloderus* LeConte, 1879** [M]

*Trogloderus* LeConte, 1879a: 2. Type species: *Trogloderus costatus* LeConte, 1879, monotypy.

***Trogloderus costatus* LeConte, 1879** USA (CA ID NV)

*Trogloderus costatus* LeConte, 1879a: 3.

***Trogloderus nevadus* La Rivers, 1943** USA (ID NV) **Status revised** [MAJ]

*Trogloderus nevadus* La Rivers, 1943a: 437.

***Trogloderus tuberculatus* Blaisdell, 1909** USA (CA) **Status revised** [MAJ]

*Trogloderus tuberculatus* Blaisdell, 1909: 490.

*Trogloderus costatus pappi* Kulzer, 1960: 310. **New synonymy** [MAJ]

***Trogloderus vandykei* La Rivers, 1946** USA (AZ CA) **Status revised** [MAJ]

*Trogloderus costatus vandykei* La Rivers, 1946: 41.

*Trogloderus costatus mayhewi* Papp, 1961a: 33. **New synonymy** [MAJ]

**Tribe Apocryphini Lacordaire, 1859**

Apocryphides Lacordaire, 1859: 432. Type genus: *Apocrypha* Eschscholtz, 1831.

**Genus *Apocrypha* Eschscholtz, 1831** [F]

*Apocrypha* Eschscholtz, 1831: 13. Type species: *Apocrypha anthicoides* Eschscholtz, 1831, monotypy.

*Compsomorphus* Solier, 1851: 208. Type species: *Compsomorphus elegans* Solier, 1851, monotypy. Synonymy: Lacordaire (1859: 433).

***Apocrypha anthicoides* Eschscholtz, 1831** USA (CA)

*Apocrypha anthicoides* Eschscholtz, 1831: 13.

*Apocrypha dyschirioides* LeConte, 1851: 137. Synonymy: Doyen and Kitayama (1980: 122).

***Apocrypha clivinoides* Horn, 1870** USA (CA)

*Apocrypha clivinoides* Horn, 1870: 391.

***Apocrypha setosa* Doyen and Kitayama, 1980** USA (CA)

*Apocrypha setosa* Doyen and Kitayama, 1980: 126.

**Genus *Pseudapocrypha* Champion, 1886** [F]

*Pseudapocrypha* Champion, 1886: 260. Type species: *Pseudapocrypha lacordairii* Champion, 1886, monotypy.

***Pseudapocrypha lacordairii* Champion, 1886** MEX (CI) GUA

*Pseudapocrypha lacordairii* Champion, 1886: 260.

**Tribe Blaptini Leach, 1815**

Blapsida Leach, 1815: 101. Type genus: *Blaps* Fabricius, 1775.

**Subtribe Blaptina Leach, 1815**

Blapsida Leach, 1815: 101. Type genus: *Blaps* Fabricius, 1775.

**Genus *Blaps* Fabricius, 1775** [F]

*Blaps* Fabricius, 1775: 254. Type species: *Tenebrio mortisagus* Linnaeus, 1758, subsequent designation (Latreille 1810: 429).

**Subgenus Blaps Fabricius, 1775**

*Blaps* Fabricius, 1775: 254. Type species: *Tenebrio mortisagus* Linnaeus, 1758, subsequent designation (Latreille 1810: 429).

***Blaps lethifera lethifera* Marsham, 1802** CAN (QC) USA (IN MD NJ NY OH VA) – Adventive

*Blaps lethifera* Marsham, 1802: 479.

*Blaps similis* Latreille, 1804: 279. Synonymy: Seidlitz (1893: 317).

***Blaps mucronata* Latreille, 1804** USA (MD NY OH) – Adventive

*Blaps mucronata* Latreille, 1804: 278.

**Tribe Bolitophagini Kirby, 1837**

Eledonaedes Billberg, 1820b: 392 [*nomen oblitum*, see Bouchard et al. 2011]. Type genus: *Eledona* Latreille, 1797.

Bolitophagidae Kirby, 1837: 236 [*nomen protectum*]. Type genus: *Bolitophagus* Illiger, 1798.

Rhipidandri LeConte, 1862a: 236. Type genus: *Rhipidandrus* LeConte, 1862.

Eutomides Lacordaire, 1865: 369. Type genus: *Eutomus* Lacordaire, 1865 (= *Rhipidandrus* LeConte, 1862).

**Genus *Bolitophagus* Illiger, 1798** [M]

*Bolitophagus* Illiger, 1798: 100. Type species: *Silpha reticulata* Linnaeus, 1767, subsequent designation (C.G. Thomson 1859: 115).

*Boletophagus* Agassiz, 1846: 48. Unjustified emendation of *Bolitophagus* Illiger, 1798, not in prevailing usage.

***Bolitophagus corticola* Say, 1826** CAN (NB NS ON PE QC) USA (CT DC FL GA IN MA MD ME MI MO NC NH NJ NY OH PA SC TN TX VA WI)

*Boletophagus corticola* Say, 1826: 238.

**Genus *Bolitotherus* Candèze, 1861** [M]

*Bolitotherus* Candèze, 1861: 367. Type species: *Bolitophagus cornutus* Fabricius, 1801, subsequent designation (LeConte 1862b: 236).

*Phellidius* LeConte, 1862a: 236. Type species: *Bolitophagus cornutus* Fabricius, 1801, original designation. Synonymy: LeConte (1866a: 62).

**Figure 23.**
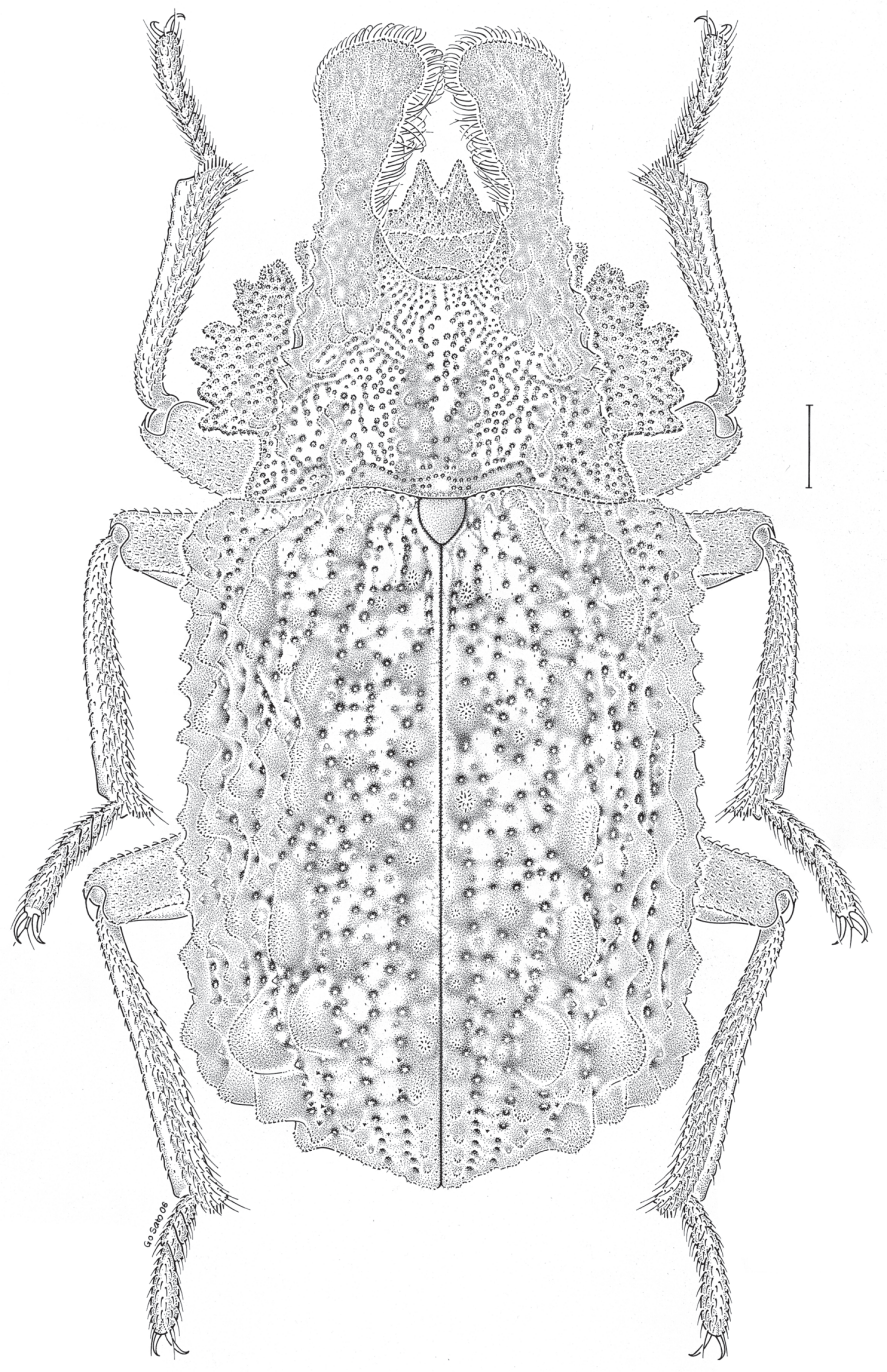
*Bolitotherus cornutus* (Fabricius, 1801). Scale bar = 1 mm.

***Bolitotherus cornutus* (Fabricius, 1801)** ^38^ [Fig. 23] CAN (AB MB NB NS ON PE QC SK) USA (CT FL GA IA IL IN KY LA MA MD ME MI MN MS NC NE NH NJ NY OH PA RI SC TN TX VAVT WI)

*Opatrum bifurcum* Fabricius, 1798: 40.

*Bolitophagus cornutus* Fabricius, 1801a: 112. Synonymy: Fabricius (1801a: 113).

*Bolitophagus cristatus* Gosse, 1840: 251. **New synonymy** [YB].

**Genus *Eleates* Casey, 1886** [M]

*Eleates* Casey, 1886: 253. Type species: *Eleates occidentalis* Casey, 1886, monotypy.

***Eleates depressus* (Randall, 1838)** CAN (BC MB NB NT ON QC SK) USA (AR GA MD ME MI NH NYOH OR PA TN VAVT WA WI)

*Eledona depressa* Randall, 1838 [February]: 21.

*Bolitophagus tetraopes* Newman, 1838 [April]: 378. Synonymy: LeConte (1854b: 219).

*Eleates explanatus* Casey, 1890b: 486. **New synonymy** [YB].

***Eleates occidentalis* Casey, 1886** USA (CA)

*Eleates occidentalis* Casey, 1886: 254.

**Genus *Megeleates* Casey, 1895** [M]

*Megeleates* Casey, 1895: 623. Type species: *Megeleates sequoiarum* Casey, 1895, monotypy.

***Megeleates sequoiarum* Casey, 1895** USA (CA OR WA)

*Megeleates sequoiarum* Casey, 1895: 624.

**Genus *Rhipidandrus* LeConte, 1862** [M]

*Rhipidandrus* LeConte, 1862a: 236. Type species: *Xyletinus flabellicornis* Sturm, 1826 (= *Melolontha paradoxa* Palisot de Beauvois, 1818), monotypy.

*Eutomus* Lacordaire, 1865: 369. Type species: *Eutomus micrographus* Lacordaire, 1865, subsequent designation (Barber 1914: 191). Synonymy: LeConte and Horn (1883: 232).

*Heptaphylla* Friedenreich, 1883: 375. Type species: *Heptaphylla fungicola* Friedenreich, 1883, monotypy. Synonymy: Arrow (1904: 31).

*Cherostus* C.O. Waterhouse, 1894: 68. Type species: *Cherostus walkeri* Waterhouse, 1894, subsequent designation (Merkl and Kompantzeva 1996: 91). Synonymy: Gebien (1939: 762).

***Rhipidandrus championi* Sharp, 1905** GUA PAN

*Rhipidandrus championi* Sharp, 1905: 691.

***Rhipidandrus cornutus* (Arrow, 1904)** MEX (DU OA) / HIS PRI LAN / SA

*Cherostus cornutus* Arrow, 1904: 31.

***Rhipidandrus fulvomaculatus* Dury, 1914** USA (FL) / BAH

*Rhipidandrus fulvomaculata* Dury, 1914: 168.

***Rhipidandrus jamaicensis* (Arrow, 1904)** JAM

*Cherostus jamaicensis* Arrow, 1904: 32.

***Rhipidandrus mexicanus* Sharp, 1905** MEX (VE) GUA BEL

*Rhipidandrus mexicanus* Sharp, 1905: 691.

***Rhipidandrus micrographus* (Lacordaire, 1865)** PRI LAN / SA

*Eutomus micrographus* Lacordaire, 1865: 370.

***Rhipidandrus panamaensis* (Barber, 1914)** PAN

*Eutomus panamaensis* Barber, 1914: 193.

**Figure 24.**
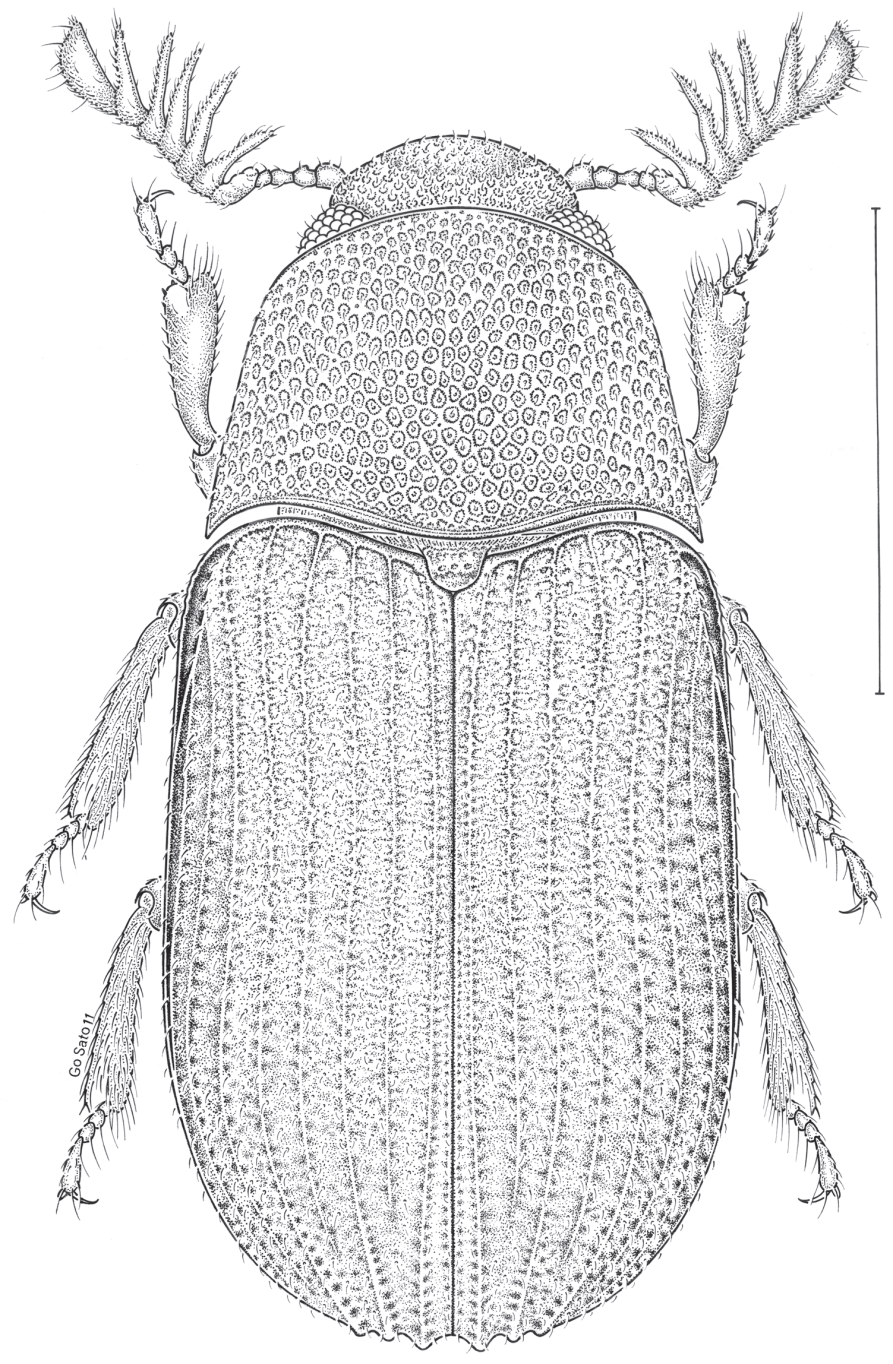
*Rhipidandrus paradoxus* (Palisot de Beauvois, 1818). Scale bar = 1 mm.

***Rhipidandrus paradoxus* (Palisot de Beauvois, 1818)** [Fig. 24] CAN (ON QC) USA (DC FL GA IN KS KY LA MD MI NC NY OH SC TX VA WI)

*Melolontha paradoxa* Palisot de Beauvois, 1818: 173.

*Xylotinus flabellicornis* Sturm, 1826: 59. Synonymy: LeConte (1873: 329, 335).

***Rhipidandrus peninsularis* Horn, 1894** USA (AZ TX) MEX (BS)

*Rhipidandrus peninsularis* Horn, 1894b: 392.

***Rhipidandrus sulcatus* (Gorham, 1898)** BAH CUB CAY HIS LAN

*Eutomus sulcatus* Gorham, 1898: 333.

**Tribe Centronopini Doyen, 1989**

Centronopini Doyen, 1989: 284. Type genus: *Centronopus* Solier, 1848.

**Genus *Centronopus* Solier, 1848** [M]

*Centronopus* Solier, 1848: 258. Type species: *Centronopus extensicollis* Solier, 1848 (= *Tenebrio suppressus* Say, 1835), original designation.

**Subgenus Centronopus Solier, 1848**

*Centronopus* Solier, 1848: 258. Type species: *Centronopus extensicollis* Solier, 1848 (= *Tenebrio suppressus* Say, 1835), original designation.

***Centronopus grandicollis* Champion, 1885** MEX (DU FD HI ME MO SL TA VE)

*Centronopus grandicollis* Champion, 1885: 100.

***Centronopus suppressus* (Say, 1835)** MEX (FD HI ME MO PU SL TA VE)

*Tenebrio suppressus* Say, 1835: 187.

*Centronopus extensicollis* Solier, 1848: 260. Synonymy: Lacordaire (1859: 361).

**Subgenus Menechides Motschulsky, 1872**

*Menechides* Motschulsky, 1872: 26. Type species: *Helops calcaratus* Fabricius, 1798, original designation.

*Scotobates* Rye, 1877: 341. Type species: *Helops calcaratus* Fabricius, 1798, subsequent designation (Lucas 1920: 587). Synonymy: Lucas (1920: 403).

*Pyres* Champion, 1885: 100. Type species: *Pyres metallicus* Champion, 1885 (= *Centronopus speciosus* Pascoe, 1883), subsequent designation (Gebien 1941: 336). Synonymy: Spilman (1962a: 3).

***Centronopus batesi* (Champion, 1885)** PAN / SA

*Pyres batesi* Champion, 1885: 101.

***Centronopus beardsleyi* Spilman, 1962** MEX (CL JA)

*Centronopus beardsleyi* Spilman, 1962a: 11.

***Centronopus bimaculatus* Champion, 1892** MEX (VE YU) BEL

*Centronopus bimaculatus* Champion, 1892: 521.

***Centronopus calcaratus* (Fabricius, 1798)** CAN (NS ON QC) USA (AL CT DC FL GA IA IL IN KS LA MA MD ME MI MO MS NC NE NH NJ NY OH PA SC TN VA VT WI WV)

*Tenebrio aeneus* DeGeer, 1775: 53 [junior primary homonym of *Tenebrio aeneus* Scopoli, 1763] [*nomen dubium*].

*Helops calcaratus* Fabricius, 1798: 52. Synonymy (in doubt): LeConte (1866a: 61).

*Tenebrio coracinus* Knoch, 1801: 172. Synonymy: LeConte (1866a: 61).

*Helops caroliniensis* Palisot de Beauvois, 1817: 162. Synonymy (in doubt): LeConte (1866a: 61).

*Tenebrio reflexus* Say, 1825: 203. Synonymy: Melsheimer (1853: 139).

***Centronopus nigrofasciatus* (Gebien, 1928)** CRI

*Pyres nigrofasciatus* Gebien, 1928b: 182.

***Centronopus opacus* LeConte, 1859** USA (AR KS MT OK SD TX)

*Centronopus opacus* LeConte, 1859a: 15.

***Centronopus speciosus* Pascoe, 1883** NIC CRI

*Centronopus speciosus* Pascoe, 1883: 439.

*Pyres metallicus* Champion, 1885: 101. Synonymy: Champion (1892: 521).

**Genus *Scotobaenus* LeConte, 1859** [M]

*Scotobaenus* LeConte, 1859b: 87. Type species: *Scotobaenus parallelus* LeConte, 1859, monotypy.

***Scotobaenus parallelus* LeConte, 1859** USA (CA OR WA) MEX

*Scotobaenus parallelus* LeConte, 1859b: 88.

***Scotobaenus punctatus* (Blaisdell, 1933)** USA (CA)

*Centronopus punctatus* Blaisdell, 1933c: 220.

***Scotobaenus simplex* (Blaisdell, 1937)** USA (CA)

*Centronopus simplex* Blaisdell, 1937a: 95.

***Scotobaenus wagneri* (Blaisdell, 1933)** USA (CA)

*Centronopus wagneri* Blaisdell, 1933c: 218.

**Genus *Tauroceras* Hope, 1841** [N]

*Tauroceras* Hope, 1841: 130. Type species: *Tenebrio cornutus* Fabricius, 1775, original designation.

*Tauroceropedus* Pic, 1913b: 4. Type species: *Tauroceropedus difformipes* Pic, 1913, subsequent designation (Gebien 1941: 344). Synonymy: Ferrer et al. (2005: 272).

***Tauroceras barclayi* Ferrer, Soldati and Delatour, 2005** JAM

*Tauroceras barclayi* Ferrer, Soldati and Delatour, 2005: 284.

***Tauroceras cornutum* (Fabricius, 1775)** CUB JAM

*Tenebrio cornutus* Fabricius, 1775: 256.

***Tauroceras girardi* Ferrer, Soldati and Delatour, 2005** MEX (QR) GUA HON NIC CRI

*Tauroceras girardi* Ferrer, Soldati and Delatour, 2005: 288.

***Tauroceras mulata* Zayas, 1988** CUB

*Tauroceras mulata* Zayas, 1988: 94.

**Tribe Cerenopini Horn, 1870**

Cerenopi Horn, 1870: 323. Type genus: *Cerenopus* LeConte, 1851.

**Genus *Argoporis* Horn, 1870** [F]

*Argoporis* Horn, 1870: 325. Type species: *Cerenopus costipennis* LeConte, 1851, subsequent designation (Gebien 1937: 797).

*Threnus* Motschulsky, 1870: 404. Type species: *Threnus niger* Motschulsky, 1870, original designation. Synonymy: Aalbu et al. (1995: 483)^39^.

***Argoporis aequalis* Blaisdell, 1923** MEX (SO)

*Argoporis aequalis* Blaisdell, 1923: 259.

***Argoporis alutacea* Casey, 1890** USA (AZ) MEX (SO)

*Argoporis alutacea* Casey, 1890b: 406.

*Argoporis labialis* Blaisdell, 1923: 258. Synonymy: Berry (1980: 18).

*Argoporis angusta* Casey, 1924: 331. Synonymy: Berry (1980: 18).

*Argoporis hebes* Casey, 1924: 332. Synonymy: Berry (1980: 18).

*Argoporis tibialis* Casey, 1924: 332. Synonymy: Berry (1980: 18).

***Argoporis apicalis apicalis* Blaisdell, 1943** MEX (BC BS)

*Argoporis apicalis* Blaisdell, 1943: 234.

*Argoporis insularis* Berry, 1980: 54. Synonymy: Sánchez Piñero and Aalbu (2002: 132).

***Argoporis apicalis californica* Berry, 1980** USA (AZ) MEX (BC)

*Argoporis apicalis californica* Berry, 1980: 22.

***Argoporis atripes* Horn, 1870** MEX (AG DU GU HI JA MI SI SL SO)

*Argoporis atripes* Horn, 1870: 325.

***Argoporis bicolor* (LeConte, 1851)** USA (AZ CA) MEX (SO)

*Cerenopus bicolor* LeConte, 1851: 143.

*Argoporis tuckeri* Casey, 1924: 332. Synonymy: Berry (1980: 27).

***Argoporis brevicollis* Champion, 1885** MEX (DU SI)

*Argoporis brevicollis* Champion, 1885: 94.

***Argoporis carinata* Berry, 1980** USA (AZ) MEX (CH NA SI SO)

*Argoporis carinata* Berry, 1980: 30.

***Argoporis cavifrons* Champion, 1885** MEX (DU SI)

*Argoporis cavifrons* Champion, 1885: 95.

***Argoporis colimensis* Berry, 1980** MEX (CL)

*Argoporis colimensis* Berry, 1980: 34.

***Argoporis costipennis* (LeConte, 1851)** USA (AZ NM) MEX (SO)

*Cerenopus costipennis* LeConte, 1851: 143.^40^

*Argoporis lateralis* Casey, 1924: 331. Synonymy: Berry (1980: 38).

***Argoporis costulata* (Horn, 1870)** MEX (BC BS)

*Cerenopus costulatus* Horn, 1870: 326.

***Argoporis craigi* Berry, 1980** MEX (CH DU)

*Argoporis craigi* Berry, 1980: 42.

***Argoporis crassicornis* Champion, 1885** MEX (DU NA SI)

*Argoporis crassicornis* Champion, 1885: 94.

***Argoporis cribrata* (LeConte, 1861)** MEX (BS)

*Cerenopus cribratus* LeConte, 1861a: 337.

***Argoporis deltodonta* Berry, 1980** MEX (Tres Marias Islands)

*Argoporis deltodonta* Berry, 1980: 46.

***Argoporis durangoensis* Berry, 1980** MEX (CH DU)

*Argoporis durangoensis* Berry, 1980: 47.

***Argoporis ebenina* Horn, 1894** MEX (BS)

*Argoporis ebenina* Horn, 1894b: 424.

***Argoporis estebanensis* Berry, 1980** MEX (BS)

*Argoporis estebanensis* Berry, 1980: 50.

***Argoporis impressa* Blaisdell, 1925** MEX (BC BS)

*Argoporis impressa* Blaisdell, 1925b: 330.

***Argoporis inconstans* Horn, 1894** MEX (BC BS)

*Argoporis inconstans* Horn, 1894b: 425.

***Argoporis laevicollis* Champion, 1892** MEX (DU SI)

*Argoporis laevicollis* Champion, 1892: 520.

***Argoporis longipes* Blaisdell, 1923** MEX (BS)

*Argoporis longipes* Blaisdell, 1923: 260.

***Argoporis nigra inflata* Berry, 1980** MEX (BS)

*Argoporis constanzae inflata* Berry, 1980: 37.

***Argoporis nigra nigra* (Motschulsky, 1870)** MEX (BS)

*Threnus niger* Motschulsky, 1870: 406^41^.

*Argoporis constanzae constanzae* Berry, 1980: 35. Synonymy: Aalbu et al. (1995: 483).

***Argoporis obregonensis* Berry, 1980** MEX (SO)

*Argoporis obregonensis* Berry, 1980: 58.

***Argoporis regalis* Berry, 1980** MEX (BS)

*Argoporis regalis* Berry, 1980: 59.

***Argoporis rufipes femorata* Berry, 1980** MEX (CH DU GU SI SL ZA)

*Argoporis rufipes femorata* Berry, 1980: 63.

***Argoporis rufipes nitida* Casey, 1890** USA (AZ NM TX) MEX (CH CO)

*Argoporis nitida* Casey, 1890b: 406.

***Argoporis rufipes rufipes* Champion, 1885** MEX (AG CH CO SL SO ZA)

*Argoporis rufipes* Champion, 1885: 94.

***Argoporis tridentata* Champion, 1892** MEX (CL GE JA MI)

*Argoporis tridentata* Champion, 1892: 519.

***Argoporis unicalcarata* Champion, 1892** MEX (AG JA NA SO)

*Argoporis unicalcarata* Champion, 1892: 519.

**Genus *Cerenopus* LeConte, 1851** [M]

*Cerenopus* LeConte, 1851: 143^42^. Type species: *Cerenopus concolor* LeConte, 1851, subsequent designation (Lucas 1920: 173).

***Cerenopus angustatus* Horn, 1894** MEX (BS)

*Cerenopus angustatus* Horn, 1894b: 426.

***Cerenopus aterrimus* Horn, 1894** MEX (BS)

*Cerenopus aterrimus* Horn, 1894b: 425.

***Cerenopus concolor* LeConte, 1851** USA (AZ CA NV) MEX (BC BS)

*Cerenopus concolor* LeConte, 1851: 143.

***Cerenopus hermanus* Berry, 1975** MEX (BS)

*Cerenopus hermanus* Berry, 1975: 931.

***Cerenopus punctatus* Berry, 1975** MEX (BS)

*Cerenopus punctatus* Berry, 1975: 932.

**Tribe Eulabini Horn, 1870**

Eulabes Horn, 1870: 323. Type genus: *Eulabis* Eschscholtz, 1829.

**Genus *Apsena* LeConte, 1862** [F]

*Apsena* LeConte, 1862a: 228. Type species: *Eulabis pubescens* LeConte, 1851, original designation.

***Apsena barbarae* Blaisdell, 1932** USA (CA)

*Apsena barbarae* Blaisdell, 1932b: 61.

***Apsena grossa* (LeConte, 1866)** USA (CA)

*Eulabis grossa* LeConte, 1866b: 118.

***Apsena insularis* Blaisdell, 1932** MEX (BC)

*Apsena insularis* Blaisdell, 1932b: 58.

***Apsena laticornis laticornis* (Casey, 1891)** USA (CA)

*Eulabis laticornis* Casey, 1891: 60.

*Apsena labreae* Pierce, 1954b: 98. Synonymy: Doyen and Miller (1980: 2).

***Apsena laticornis subvestita* Blaisdell, 1932** USA (CA)

Apsena laticornis var. subvestita Blaisdell, 1932b: 68.

***Apsena leachi* Blaisdell, 1932** USA (CA)

*Apsena leachi* Blaisdell, 1932b: 70.

***Apsena pubescens pubescens* (LeConte, 1851)** USA (CA)

*Eulabis pubescens* LeConte, 1851: 143.

*Eulabis crassicornis* Casey, 1890b: 404. Synonymy: Blaisdell (1925a: 83).

***Apsena pubescens rufescens* Blaisdell, 1932** MEX (BC)

*Apsena pubescens rufescens* Blaisdell, 1932b: 56.

***Apsena rufipes opaca* Blaisdell, 1932** USA (CA)

Apsena rufipes var. opaca Blaisdell, 1932b: 81.

***Apsena rufipes rufipes* (Eschscholtz, 1829)** USA (CA)

*Eulabis rufipes* Eschscholtz, 1829: 15.

*Eulabis montana* Casey, 1924: 330. Synonymy: Blaisdell (1925a: 84).

***Apsena rufipes simplex* Blaisdell, 1932** USA (CA)

*Apsena rufipes simplex* Blaisdell, 1932b: 84.

**Genus *Epantius* LeConte, 1851** [M]

*Epantius* LeConte, 1851: 144. Type species: *Epantius obscurus* LeConte, 1851, monotypy.

***Epantius obscurus* LeConte, 1851** USA (CA) MEX (BC)

*Epantius obscurus* LeConte, 1851: 144.

**Genus *Eulabis* Eschscholtz, 1829** [F]

*Eulabis* Eschscholtz, 1829: 14. Type species: *Eulabis bicarinata* Eschscholtz, 1829, subsequent designation (Blaisdell 1932b: 44).

***Eulabis bicarinata* Eschscholtz, 1829** USA (CA)

*Eulabis bicarinata* Eschscholtz, 1829: 15.

**Tribe Helopini Latreille, 1802**

Helopii Latreille, 1802: 176. Type genus: *Helops* Fabricius, 1775.

**Genus *Helops* Fabricius, 1775** ^43^ [M]

*Helops* Fabricius, 1775: 257. Type species: *Tenebrio caeruleus* Linnaeus, 1758, subsequent designation (Hope 1841: 133) (see ICZN 2009)^44^.

*Stenotrichus* LeConte, 1862a: 239. Type species: *Amphidora rufipes* LeConte, 1851, original designation. Synonymy: Aalbu et al. (2002: 496).

*Biomorphus* Motschulsky, 1872: 38. Type species: *Biomorphus tuberculatus* Motschulsky, 1872 (= *Amphidora attenuata* LeConte, 1851), original designation. Synonymy: Aalbu et al. (1995: 485).

*Coscinoptilix* Allard, 1876: 15 [as *Coscinopter*]^45^. Type species: *Coscinoptilix gracilicornis* Allard, 1876, monotypy. Synonymy: Champion (1887: 312).

***Helops angustus* LeConte, 1859** USA (CA)

*Helops angustus* LeConte, 1859b: 77.

***Helops arizonensis* Horn, 1874** USA (AZ NM)

*Helops arizonensis* Horn, 1874a: 36.

***Helops attenuatus* (LeConte, 1851)** USA (CA NV)

*Amphidora attenuata* LeConte, 1851: 136.

*Biomorphus tuberculatus* Motschulsky, 1872: 40. Synonymy: Aalbu et al. (1995: 485).

***Helops bachei* LeConte, 1861** USA (CA)

*Helops bachei* LeConte, 1861b: 353.

***Helops benitensis* Blaisdell, 1925** MEX (BC)

*Helops benitensis* Blaisdell, 1925b: 339.

***Helops blaisdelli* Casey, 1891** USA (CA)

*Helops blaisdelli* Casey, 1891: 66.

***Helops blandi* Bousquet and Bouchard, 2012** CAN (NB) USA (MD NJ NY SC VA)

*Helops gracilis* Bland, 1864: 319 [junior primary homonym of *Helops gracilis* Fischer von Waldheim, 1823].

*Helops blandi* Bousquet and Bouchard [in Nabozhenko et al.], 2012: 729. Replacement name for *Helops gracilis* Bland, 1864.

***Helops callosus* Casey, 1890** USA (NM)

*Helops callosa* Casey, 1890b: 489.

***Helops cavifrons* Champion, 1887** GUA

*Helops cavifrons* Champion, 1887: 313.

***Helops cisteloides* Germar, 1823** USA (AR FL GA LA MD MO NC NJ OH SC TX VA)

*Helops cisteloides* Germar, 1823: 159.

***Helops confluens* (Casey, 1924)** USA (CA) **Status revised** [RLA]

*Stenotrichus confluens* Casey, 1924: 329.

***Helops coxalis* Champion, 1887** MEX (MI)

*Helops coxalis* Champion, 1887: 317.

***Helops crockeri* Blaisdell, 1933** MEX (BC [Guadalupe Is.])

*Helops crockeri* Blaisdell, 1933a: 89.

***Helops cupripennis* Champion, 1887** MEX (OA)

*Helops cupripennis* Champion, 1887: 319.

***Helops cylindriformis* Casey, 1891** USA (NM)

*Helops cylindriformis* Casey, 1891: 68.

***Helops difficilis* Horn, 1878** USA (CO WY)

*Helops difficilis* Horn, 1878a: 57.

***Helops discipulus* Casey, 1891** USA (CA)

*Helops discipula* Casey, 1891: 67.

***Helops discretus* LeConte, 1866** USA (TX)

*Helops discretus* LeConte, 1866b: 134.

***Helops edwardsii* Horn, 1870** USA (CA OR WA)

*Helops edwardsii* Horn, 1870: 395.

***Helops enitescens* Champion, 1893** GUA

*Helops enitescens* Champion, 1893a: 557.

***Helops exsculptus* Champion, 1887** GUA

*Helops exsculptus* Champion, 1887: 314.

***Helops farctus* LeConte, 1858** USA (TX)

*Helops farcta* LeConte, 1858b: 74.

***Helops fresnoensis* Blaisdell, 1931** USA (CA)

*Helops fresnoënsis* Blaisdell, 1931: 44.

***Helops gracilicornis* (Allard, 1876)** MEX (VE)

*Coscinoptilix gracilicornis* Allard, 1876: 52.

***Helops guadalupensis* Casey, 1890** MEX (BC [Guadalupe Is.])

*Helops guadalupensis* Casey, 1890b: 488.

***Helops impolitus* LeConte, 1866** USA (TX)

*Helops impolitus* LeConte, 1866b: 132.

***Helops inanis* (Allard, 1877)** MEX (MO PU)

*Tarpela inanis* Allard, 1877b: 262.

*Helops funebris* Champion, 1887: 316. Synonymy: Champion (1893a: 556).

***Helops laetus* LeConte, 1857** CAN (BC) USA (CA OR WA)

*Helops laetus* LeConte, 1857: 50.

***Helops longicornis* Champion, 1887** MEX (DU)

*Helops longicornis* Champion, 1887: 314.

***Helops noguerai* Doyen, 1990** MEX (JA)

*Helops noguerai* Doyen, 1990: 236.

***Helops obtusangulus* Blaisdell, 1921** USA (CA)

*Helops obtusangula* Blaisdell, 1921b: 228.

***Helops opacus* LeConte, 1859** USA (CA ID NV OR UT)

*Helops opacus* LeConte, 1859c: 284.

***Helops panamensis* Champion, 1887** PAN

*Helops panamensis* Champion, 1887: 319.

***Helops perforatus* Horn, 1880** USA (TX)

*Helops perforatus* Horn, 1880: 153.

**Figure 25.**
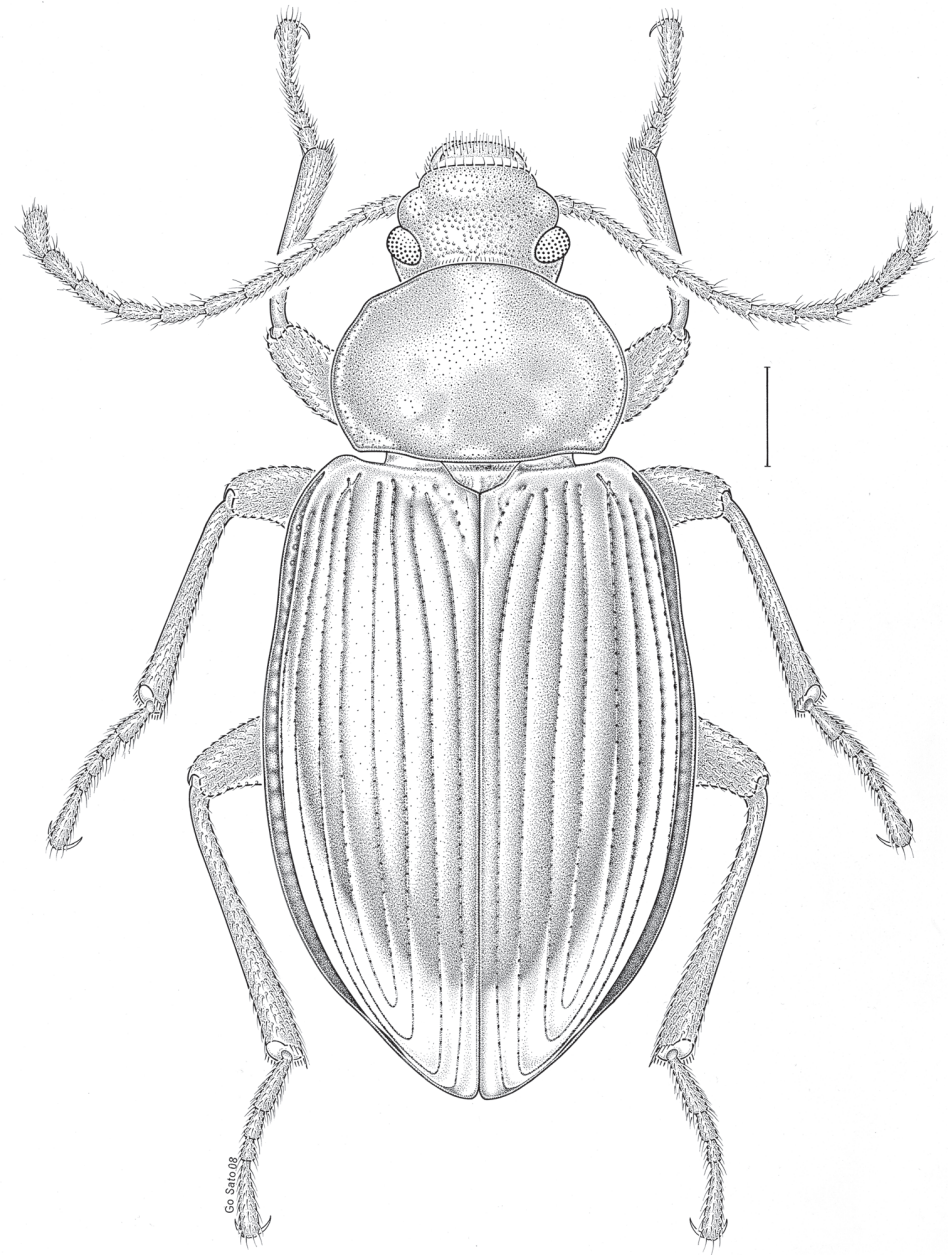
*Helops pernitens* LeConte, 1861. Scale bar = 1 mm.

***Helops pernitens* LeConte, 1861** [Fig. 25] CAN (BC) USA (CA OR WA)

*Helops pernitens* LeConte, 1861b: 353.

***Helops pinguis* Horn, 1894** MEX (BS)

*Helops pinguis* Horn, 1894b: 430.

***Helops politus* Say, 1826** USA (FL)

*Helops politus* Say, 1826: 240.

***Helops pueblensis* Champion, 1887** MEX (GE PU)

*Helops pueblensis* Champion, 1887: 317.

***Helops punctatostriatus* Champion, 1887** MEX

*Helops punctato-striatus* Champion, 1887: 316.

***Helops punctatus* Gemminger, 1870** USA (CA)

*Helops punctipennis* LeConte, 1866b: 133 [junior primary homonym of *Helops punctipennis* Lucas, 1846].

*Helops punctatus* Gemminger, 1870: 123 [junior primary homonym of *Helops punctatus* Fabricius, 1801]^46^. Replacement name for *Helops punctipennis* LeConte, 1866.

***Helops punctiventris* Champion, 1887** MEX (GU)

*Helops punctiventris* Champion, 1887: 320.

***Helops rastratus* Champion, 1893** MEX (CH)

*Helops rastratus* Champion, 1893a: 557.

***Helops rufipes* (LeConte, 1851)** USA (CA)

*Amphidora rufipes* LeConte, 1851: 136.

*Amphidora parallela* Casey, 1924: 328. Synonymy: Blaisdell (1933b: 210).

***Helops rugiceps* Champion, 1887** GUA

*Helops rugiceps* Champion, 1887: 315.

***Helops rugicollis* LeConte, 1866** USA (CA)

*Helops rugicollis* LeConte, 1866b: 133.

***Helops rugulosus* LeConte, 1851** USA (CA)

*Helops rugulosus* LeConte, 1851: 151.

***Helops scintillatus* Doyen, 1990** MEX (JA NA)

*Helops scintillatus* Doyen, 1990: 239.

***Helops seriatoporus* Champion, 1893** MEX (CH)

*Helops seriatoporus* Champion, 1893a: 558.

***Helops seriatus* (Allard, 1877)** USA (CA)

*Catomus seriatus* Allard, 1877b: 46.

***Helops simulator* Blaisdell, 1921** USA (CA OR)

*Helops simulator* Blaisdell, 1921b: 226.

***Helops sparsus* Blaisdell, 1943** MEX (BC)

*Helops sparsus* Blaisdell, 1943: 274.

***Helops spiethi* Pallister, 1954** MEX (DU)

*Helops spiethi* Pallister, 1954: 50.

***Helops spilmani* Pallister, 1954** MEX (CH DU)

*Helops spilmani* Pallister, 1954: 49.

***Helops spissicornis* Champion, 1893** MEX (DU)

*Helops spissicornis* Champion, 1893a: 558.

***Helops spretus* Horn, 1880** USA (NV)

*Helops spretus* Horn, 1880: 153.

***Helops stenotrichoides* Blaisdell, 1895** USA (CA)

*Helops stenotrichoides* Blaisdell, 1895: 238.

***Helops strigicollis* Horn, 1885** USA (CA)

*Helops strigicollis* Horn, 1885c: 161.

***Helops suavis* Champion, 1887** MEX (OA) GUA

*Helops suavis* Champion, 1887: 318.

***Helops sulcipennis* LeConte, 1866** USA (AL GA NC SC TN VA)

*Helops sulcipennis* LeConte, 1866b: 133.

***Helops sumptuosus* (Allard, 1876)** MEX

*Diastixus sumptuosus* Allard, 1876: 57.

***Helops tristis* Palisot de Beauvois, 1817** USA (SC)

*Helops tristis* Palisot de Beauvois, 1817: 138 [junior primary homonym of *Helops tristis* Rossi, 1790].^47^

***Helops tumescens* LeConte, 1866** USA (AZ CA)

*Helops tumescens* LeConte, 1866b: 134.

**Genus *Nalassus* Mulsant, 1854** [M]

*Nalassus* Mulsant, 1854: 323. Type species: *Helops dryadophilus* Mulsant, 1854, subsequent designation (Nabozhenko 2001: 630).

***Nalassus aereus* (Germar, 1823)** USA (AL CT DC DE GA IL IN KS KY MD MO MS NC NJ NY OH PA SC TN VA WV)

*Helops aereus* Germar, 1823: 160.

*Helops pullus* Say, 1826: 240. Synonymy: LeConte (1866a: 63).

*Helops aratus* Say, 1826: 241. Synonymy: LeConte (1866a: 63).

*Helops carolina* Manee, 1924: 40. Synonymy: Steiner (2009: 332).

***Nalassus californicus* (Mannerheim, 1843)** USA (CA ID NV OR WA) MEX

*Helops californicus* Mannerheim, 1843: 287.

***Nalassus convexulus* (LeConte, 1861)** CAN (AB BC) USA (CA CO ID MT NE NV OR UT WA WY)

*Helops convexulus* LeConte, 1861b: 353.

*Helops inclusus* Walker, 1866: 330. Synonymy: Blair (1921: 283).

*Helops montanus* LeConte, 1879b: 518. Synonymy: Bousquet and Campbell (1991: 258).

*Helops regulus* Blaisdell, 1921b: 227. Synonymy: Boddy (1965: 177).

**Genus *Nautes* Pascoe, 1866** [M]

*Nautes* Pascoe, 1866: 475. Type species: *Nautes fervidus* Pascoe, 1866, monotypy.

***Nautes alternans* Champion, 1893** GUA

*Nautes alternans* Champion, 1893a: 550.

***Nautes antennatus* Champion, 1887** PAN

*Nautes antennatus* Champion, 1887: 281.

***Nautes asperipennis* Allard, 1894** CUB

*Nautes asperipennis* Allard, 1894: 259.

***Nautes azurescens* (Jacquelin du Val, 1857)** USA (FL) / BAH CUB

*Helops azurescens* Jacquelin du Val, 1857: 153.

*Helops viridimicans* Horn, 1878a: 57. Synonymy: Steiner (2005: 454).

***Nautes belti* Allard, 1877** NIC PAN

*Nautes belti* Allard, 1877b: 59.

***Nautes breviceps* Champion, 1887** PAN

*Nautes breviceps* Champion, 1887: 282.

***Nautes chrysomeloides* Champion, 1887** BEL

*Nautes chrysomeloides* Champion, 1887: 284.

***Nautes enoplopoides* Champion, 1887** GUA

*Nautes enoplopoides* Champion, 1887: 287.

***Nautes fervidus* Pascoe, 1866** MEX (VE) GUA NIC

*Nautes fervidus* Pascoe, 1866: 476.

*Nautes aeneus* Bates, 1870: 270. Synonymy: Champion (1887: 278).

***Nautes glabratus* Champion, 1887** MEX (VE)

*Nautes glabratus* Champion, 1887: 278.

***Nautes guanahani* Steiner, 2006** BAH

*Nautes guanahani* Steiner, 2006: 29.

***Nautes hilaris* Champion, 1887** GUA

*Nautes hilaris* Champion, 1887: 286.

***Nautes laeviventris* Champion, 1887** GUA

*Nautes laeviventris* Champion, 1887: 285.

***Nautes magnificus* Champion, 1887** GUA

*Nautes magnificus* Champion, 1887: 284.

***Nautes nitidissimus* Champion, 1887** MEX (VE)

*Nautes nitidissimus* Champion, 1887: 286.

***Nautes nodulosus* Champion, 1887** GUA

*Nautes nodulosus* Champion, 1887: 287.

***Nautes rufipes* Allard, 1876** CUB

*Nautes rufipes* Allard, 1876: 45.

***Nautes splendens* Champion, 1887** PAN

*Nautes splendens* Champion, 1887: 280.

***Nautes stabilis* Champion, 1893** MEX (OA VE)

*Nautes stabilis* Champion, 1893a: 550.

***Nautes striatipennis* Champion, 1887** MEX (OA)

*Nautes striatipennis* Champion, 1887: 283.

***Nautes tinctus* Champion, 1887** GUA

*Nautes tinctus* Champion, 1887: 279.

***Nautes tricolor* Champion, 1893** MEX (PU)

*Nautes tricolor* Champion, 1893a: 551.

***Nautes varians* Champion, 1887** MEX (OA VE)

*Nautes varians* Champion, 1887: 281.

***Nautes versicolor* Champion, 1887** GUA

*Nautes versicolor* Champion, 1887: 284.

**Genus *Neohelops* Dajoz, 2001** [M]

*Neohelops* Dajoz, 2001: 356. Type species: *Neohelops texanus* Dajoz, 2001, original designation.

***Neohelops texanus* Dajoz, 2001** USA (TX)

*Neohelops texanus* Dajoz, 2001: 357.

**Genus *Tarpela* Bates, 1870** [F]

*Tarpela* Bates, 1870: 272. Type species: *Tarpela brownii* Bates, 1870, subsequent designation (Gebien 1943: 407).

*Lamperos* Allard, 1876: 4. Type species: *Helops micans* Fabricius, 1798, subsequent designation (Nabozhenko and Löbl 2008: 256). Synonymy: Champion (1887: 288).

***Tarpela aerifera* Allard, 1876** MEX (PU VE) PAN

*Tarpela aerifera* Allard, 1876: 47.

***Tarpela allardi* Champion, 1887** MEX (VE)

*Tarpela allardi* Champion, 1887: 307.

***Tarpela amabilis* Champion, 1887** GUA

*Tarpela amabilis* Champion, 1887: 308.

***Tarpela atra* Allard, 1876** MEX (DU JA MI PU)

*Tarpela atra* Allard, 1876: 46.

***Tarpela azteca* Champion, 1887** MEX (GU)

*Tarpela azteca* Champion, 1887: 300.

***Tarpela brownii* Bates, 1870** NIC PAN

*Tarpela brownii* Bates, 1870: 272.

***Tarpela cactivora* Zayas, 1988** CUB

*Tarpela cactivora* Zayas, 1988: 105.

***Tarpela catenata* Champion, 1895** MEX (YU)

*Tarpela catenulata* Champion, 1893a: 552 [junior primary homonym of *Tarpela catenulata* Allard, 1877].

*Tarpela catenata* Champion, 1895: 215. Unjustified emendation of *Tarpela catenulata* Champion, 1893.

***Tarpela cisteliformis* Allard, 1877** MEX GUA

*Tarpela cisteliformis* Allard, 1877b: 57.

***Tarpela contigua* Champion, 1887** MEX (MI)

*Tarpela contigua* Champion, 1887: 298.

***Tarpela corpulenta* Champion, 1887** MEX (DU)

*Tarpela corpulenta* Champion, 1887: 292.

***Tarpela costata* Champion, 1887** MEX (GE JA SI)

*Tarpela costata* Champion, 1887: 293.

***Tarpela crassipes* Champion, 1887** MEX (OA)

*Tarpela crassipes* Champion, 1887: 306.

***Tarpela cupreoviridis* Allard, 1877** MEX (YU) GUA NIC

*Tarpela cupreo-viridis* Allard, 1877b: 57.

***Tarpela cuprosa* Zayas, 1988: 106** CUB

*Tarpela cuprosa* Zayas, 1988: 106.

***Tarpela depressa* Champion, 1887** MEX (JA YU)

*Tarpela depressa* Champion, 1887: 306.

***Tarpela docilis* Champion, 1887** MEX (VE)

*Tarpela docilis* Champion, 1887: 312.

***Tarpela durangoensis* Champion, 1887** MEX (DU)

*Tarpela durangoensis* Champion, 1887: 292.

***Tarpela eximia* (Bates, 1870)** NIC

*Nautes eximius* Bates, 1870: 271.

***Tarpela fallax* Champion, 1887** MEX (TA VE)

*Tarpela fallax* Champion, 1887: 301.

***Tarpela flohri* Champion, 1893** MEX (MO)

*Tarpela flohri* Champion, 1893a: 553.

***Tarpela foveipennis* Champion, 1887** MEX (CI)

*Tarpela foveipennis* Champion, 1887: 294.

***Tarpela foveolata* Champion, 1893** MEX (TA)

*Tarpela foveolata* Champion, 1893a: 554.

***Tarpela fragilicornis* Champion, 1887** MEX (OA)

*Tarpela fragilicornis* Champion, 1887: 309.

***Tarpela granulipennis* (Jacquelin du Val, 1857)** CUB

*Helops granulipennis* Jacquelin du Val, 1857: 154.

***Tarpela guerreroensis* Champion, 1893** MEX (GE)

*Tarpela guerreroensis* Champion, 1893a: 555.

***Tarpela hispidula* Allard, 1876** MEX

*Tarpela hispidula* Allard, 1876: 47.

***Tarpela hoegei* Champion, 1887** MEX (DU)

*Tarpela högei* Champion, 1887: 297.

***Tarpela inaequalis* Champion, 1887** PAN

*Tarpela inaequalis* Champion, 1887: 290.

***Tarpela incilis* Champion, 1893** MEX (JA)

*Tarpela incilis* Champion, 1893a: 553.

***Tarpela jalapensis* Champion, 1887** MEX (GE VE)

*Tarpela jalapensis* Champion, 1887: 296.

***Tarpela marginicollis* Champion, 1887** GUA

*Tarpela marginicollis* Champion, 1887: 302.

***Tarpela micans* (Fabricius, 1798)** CAN (ON QC) USA (AL CT GA IL IN MA MD NC NY OH SC TN VA)

*Helops vittatus* Olivier, 1793: 45 [*nomen oblitum*: see Appendix 4 for supporting references].

*Helops micans* Fabricius, 1798: 51 [*nomen protectum*]. Synonymy: Illiger (1802: 343).

*Helops taeniatus* Palisot de Beauvois, 1812: 121. Synonymy: Dejean (1821: 70).

***Tarpela nigerrima* Champion, 1893** MEX (GE)

*Tarpela nigerrima* Champion, 1893a: 555.

***Tarpela oblonga* Champion, 1887** MEX (VE)

*Tarpela oblonga* Champion, 1887: 298.

***Tarpela oblongopunctata* Bates, 1870** MEX

*Tarpela oblongopunctata* Bates, 1870: 273.

***Tarpela occidentalis* (Allard, 1877)** JAM

*Nesotes occidentalis* Allard, 1877b: 40.

*Helops mutabilis* C.O. Waterhouse, 1878: 304. Synonymy: Champion (1894: lxxxv).

***Tarpela propinqua* (C.O. Waterhouse, 1878)** JAM

*Helops propinquus* C.O. Waterhouse, 1878: 305.

***Tarpela pulchra* Champion, 1893** MEX (VE)

*Tarpela pulchra* Champion, 1893a: 551.

***Tarpela puncticeps* Champion, 1887** GUA

*Tarpela puncticeps* Champion, 1887: 303.

***Tarpela reticulata* Champion, 1887** HON

*Tarpela reticulata* Champion, 1887: 293.

***Tarpela sculptilis* Champion, 1887** MEX (VE)

*Tarpela sculptilis* Champion, 1887: 295.

***Tarpela setigera* Champion, 1887** MEX (VE)

*Tarpela setigera* Champion, 1887: 297.

***Tarpela silvicola* Champion, 1887** GUA

*Tarpela silvicola* Champion, 1887: 309.

***Tarpela sinuaticollis* Champion, 1887** PAN

*Tarpela sinuaticollis* Champion, 1887: 303.

***Tarpela socia* Champion, 1887** MEX (GE JA SI)

*Tarpela socia* Champion, 1887: 299.

***Tarpela subparallela* Champion, 1887** MEX (SL)

*Tarpela subparallela* Champion, 1887: 300.

***Tarpela subvittata* Champion, 1887** GUA

*Tarpela subvittata* Champion, 1887: 305.

***Tarpela suturalis* Champion, 1887** GUA

*Tarpela suturalis* Champion, 1887: 310.

***Tarpela teapensis* Champion, 1893** MEX (TB)

*Tarpela teapensis* Champion, 1893a: 556.

***Tarpela tenuicornis* Champion, 1887** GUA

*Tarpela tenuicornis* Champion, 1887: 289.

***Tarpela thoracica* Champion, 1887** NIC

*Tarpela thoracica* Champion, 1887: 293.

***Tarpela torrida* Champion, 1887** MEX (DU YU)

*Tarpela torrida* Champion, 1887: 291.

***Tarpela totonicapamensis* Champion, 1887** GUA

*Tarpela totonicapamensis* Champion, 1887: 311.

***Tarpela tropicalis* Champion, 1887** GUA

*Tarpela tropicalis* Champion, 1887: 304.

***Tarpela undulata* (LeConte, 1866)** USA (FL GA IN MD NC OH PA SC TN VA)

*Helops americanus* Palisot de Beauvois, 1812: 122 [*nomen dubium*]^48^.

*Helops undulatus* LeConte, 1866b: 132. Synonymy: Horn (1885a: 89).

***Tarpela venusta* (Say, 1824)** USA (AL GA MD MO NC NY OH PA SC TN VA)

*Helops venustus* Say, 1824b: 284.

***Tarpela veraepacis* Champion, 1887** GUA

*Tarpela veraepacis* Champion, 1887: 295.

***Tarpela virescens* (Laporte, 1840)** “Amérique du Nord”

*Helops virescens* Laporte, 1840: 235.

**Tribe Melanimonini Seidlitz, 1894**

Microzoumates Mulsant, 1854: 176. Type genus: *Microzoum* Dejean, 1834 (= *Melanimon* Steven 1829).

Melanimonina Seidlitz, 1894: 449. Type genus: *Melanimon* Steven, 1829. Note.Use of younger family-group name conserved (ICZN 1999: Art. 40.2) (see Bouchard et al. 2005).

**Genus *Cheirodes* Gené, 1839** [M]

*Cheirodes* Gené, 1839: 73. Type species: *Cheirodes sardous* Gené, 1839, monotypy.

*Anemia* Laporte, 1840: 218. Type species: *Anemia granulata* Laporte, 1840, monotypy. Synonymy: Spilman (1973: 41).

*Chirodes* Agassiz, 1846: 81. Unjustified emendation of *Cheirodes* Gené, 1839, not in prevailing usage.

***Cheirodes californicus* (Horn, 1870)** USA (CA NV OR WA)

*Anaemia californica* Horn, 1870: 378.

**Tribe Metaclisini Steiner, 2016**

Metaclisini Steiner, 2016: 542. Type genus: *Metaclisa* Jacquelin du Val, 1861.

**Genus *Metaclisa* Jacquelin du Val, 1861** [F]

*Amarantha* Motschulsky, 1859: 141 [*nomen oblitum*, see Bouchard et al. (2007: 393)]. Type species: *Amarantha viridis* Motschulsky, 1859, monotypy.

*Metaclisa* Jacquelin du Val, 1861: 296 [*nomen protectum*]. Type species: *Platydema parallela* Fairmaire, 1855 (= *Diaperis azurea* Waltl, 1838), original designation. Synonymy: Lewis (1891: 70).

*Tharsus* LeConte, 1862a: 233. Type species: *Tharsus seditiosus* LeConte, 1862, monotypy. Synonymy: Steiner (2016: 538).

***Metaclisa atra* LeConte, 1866** USA (AL DC FL GA LA MD MO MS NC PA SC TX VA)

*Metaclisa atra* LeConte, 1866b: 127.

*Haplandrus collaris* Casey, 1924: 320. Synonymy: Steiner (2016: 537).

*Haplandrus subangusta* Casey, 1924: 320. Synonymy: Steiner (2016: 537).

***Metaclisa marginalis* Horn, 1870** CAN (BC) USA (CA OR WA)

*Metaclisa marginalis* Horn, 1870: 369.

***Metaclisa seditiosa* (LeConte, 1862)** USA (AL FL GA KY MD NC OH SC TN TX VA WV) / BAH

*Tharsus seditiosus* LeConte, 1862a: 233.

**Tribe Opatrini Brullé, 1832**

Opatrites Brullé, 1832: 213. Type genus: *Opatrum* Fabricius, 1775.

**Subtribe Opatrina Brullé, 1832**

Opatrites Brullé, 1832: 213. Type genus: *Opatrum* Fabricius, 1775.

Blapstinites Mulsant and Rey, 1853: 258. Type genus: *Blapstinus* Dejean, 1821.

**Genus *Aconobius* Casey, 1895** [M]

*Aconobius* Casey, 1895: 617. Type species: *Conibiosoma laciniata* Casey, 1891, original designation.

***Aconobius densus* Casey, 1914** USA (NM)

*Aconobius densus* Casey, 1914: 377.

***Aconobius laciniatus* (Casey, 1891)** USA (AZ)

*Conibiosoma laciniata* Casey, 1891: 64.

***Aconobius nigripes* Casey, 1914** USA (TX)

*Aconobius nigripes* Casey, 1914: 378.

**Genus *Ammodonus* Mulsant and Rey, 1859** [M]

*Ammodonus* Mulsant and Rey, 1859: 143. Type species: *Opatrum fossor* LeConte, 1847, monotypy.

*Pseudonomus* Fairmaire, 1884: 510. Type species: *Pseudonomus dermestiformis* Fairmaire, 1884, monotypy. Synonymy: Gebien (1939: 470).

*Scaptes* Champion, 1886: 222. Type species: *Scaptes squamulatus* Champion, 1886 (= *Asida tropica* Kirsch, 1866), **present designation**. Synonymy: Fall (1912: 48).

*Trichotoides* Marcuzzi, 1954b: 23. Type species: *Scaptes hintoni* Kaszab, 1949, monotypy. Synonymy: Ferrer and Moraguès (2001: 499).

***Ammodonus ciliatus* (Champion, 1896)** LAN / SA

*Scaptes ciliatus* Champion, 1896: 9.

***Ammodonus fossor* (LeConte, 1847)** CAN (ON) USA (AL AR DE IL IN KS MD MN NC NE NJ NY OH OK SC TX WI WV)

*Opatrum fossor* LeConte, 1847: 92.

***Ammodonus granosus* Fall, 1912** USA (AZ) MEX (BS)

*Ammodonus granosus* Fall, 1912: 47.

***Ammodonus tropicus* (Kirsch, 1866)** USA (AZ CA) MEX (AG CH CI JA MI NA NL OA PU SI SO VE) GUA BEL SAL HON NIC CRI PAN / CUB JAM / SA

*Asida tropica* Kirsch, 1866: 190.

*Scaptes squamulatus* Champion, 1886: 223. Synonymy: Champion (1893a: 542).

**Genus *Blapstinus* Dejean, 1821** [M]

*Blapstinus* Dejean, 1821: 66. Type species: *Blaps punctata* Fabricius, 1792, monotypy.

*Heteropus* Laporte, 1840: 221 [junior homonym of *Heteropus* Palisot de Beauvois, 1820]. Type species: *Heteropus holosericeus* Laporte, 1840, monotypy. Synonymy: Lacordaire (1859: 250).

*Pedonoeces* G.R. Waterhouse, 1845: 32. Type species: *Pedonoeces galapagoensis* G.R. Waterhouse, 1845, subsequent designation (Aalbu and Triplehorn 1991: 170). Synonymy: Aalbu and Triplehorn (1991: 170).

*Tessaromma* Boheman, 1858: 91 [junior homonym of *Tessaromma* Newman, 1840]. Type species: *Tessaromma lugubris* Boheman, 1858, subsequent designation (Aalbu and Triplehorn 1991: 170). Synonymy: Aalbu and Triplehorn (1991: 170).

*Lachnoderes* Mulsant and Rey, 1859: 166. Type species: *Pedonoeces pubescens* G.R. Waterhouse, 1845, monotypy. Synonymy: Aalbu and Triplehorn (1991: 170).

*Aspidius* Mulsant and Rey, 1859: 187. Type species: *Blaps punctata* Fabricius, 1792, **present designation**. Synonymy: Champion (1885: 124).

*Lodinus* Mulsant and Rey, 1859: 195. Type species: *Lodinus nigroaeneus* Mulsant and Rey, 1859 (= *Blapstinus punctulatus* Solier, 1851), monotypy. Synonymy: Gemminger [in Gemminger and Harold] (1870: 1923).

***Blapstinus aciculus* Blatchley, 1917** USA (FL) / BAH

*Blapstinus aciculus* Blatchley, 1917: 275.

***Blapstinus amnosus* Blaisdell, 1923** MEX (BC)

*Blapstinus amnosus* Blaisdell, 1923: 272.

***Blapstinus angustatus* Champion, 1893** MEX (OA)

*Blapstinus angustatus* Champion, 1893a: 528.

***Blapstinus aridus* Blaisdell, 1923** MEX (SO)

*Blapstinus aridus* Blaisdell, 1923: 270.

***Blapstinus atratus* Champion, 1885** MEX (GE PU YU) GUA NIC PAN

*Blapstinus atratus* Champion, 1885: 131.

***Blapstinus auripilis* Horn, 1870** USA (AZ)

*Blapstinus auripilis* Horn, 1870: 353.

***Blapstinus barri* Boddy, 1957** USA (ID OR)

*Blapstinus barri* Boddy, 1957: 198.

***Blapstinus brevicollis* LeConte, 1851** USA (AZ CA) MEX (SO)

*Blapstinus brevicollis* LeConte, 1851: 147.

*Blapstinus sonorae* Casey, 1890b: 431. **New synonymy** [based on Davis (1970: 131) unpublished thesis].

***Blapstinus buqueti* Champion, 1885** CRI PAN /LAN / SA

*Blapstinus buqueti* Champion, 1885: 128.

*Blapstinus piliferus* Fairmaire, 1892: 82. Synonymy: Marcuzzi (1951: 75).

***Blapstinus castaneus* Casey, 1890** USA (AZ CA TX)

*Blapstinus castaneus* Casey, 1890b: 432.

*Blapstinus falli* Blaisdell, 1929b: 21. **New synonymy** [based on Davis (1970: 257) unpublished thesis].

***Blapstinus cubanus cubanus* Marcuzzi, 1962** CUB CAY

*Blapstinus cubanus* Marcuzzi, 1962: 33.

***Blapstinus cubanus grandturki* Marcuzzi, 1965** BAH

*Blapstinus cubanus grandturki* Marcuzzi, 1965: 130.

***Blapstinus cylindriformis* Doyen, 1990** MEX (GE JA)

*Blapstinus cylindriformis* Doyen, 1990: 232.

***Blapstinus decui* Ardoin, 1977** CUB

*Blapstinus decui* Ardoin, 1977c: 390.

***Blapstinus debilis* Casey, 1890** USA (FL TX)

*Blapstinus debilis* Casey, 1890b: 458.

***Blapstinus densipunctatus* Blaisdell, 1943** MEX (BS)

*Blapstinus densipunctatus* Blaisdell, 1943: 256.

***Blapstinus dilatatus* LeConte, 1851** USA (AZ CA CO SD UT) MEX (CH SO)

*Opatrum pullum* Say, 1826: 237 [*nomen dubium*].

*Blapstinus dilatatus* LeConte, 1851: 146. Synonymy (in doubt): LeConte (1866a: 61).

***Blapstinus discolor* Horn, 1870** CAN (BC) USA (CA ID NV OR UT WA) MEX (BS)

*Blapstinus discolor* Horn, 1870: 354.

*Blapstinus oregonensis* Casey, 1890b: 435. Synonymy: Davis (1982: 254).

*Blapstinus fuliginosus* Casey, 1890b: 438. Synonymy: Davis (1982: 254).

*Blapstinus rufipes* Casey, 1890b: 439. Synonymy: Davis (1982: 254).

*Blapstinus crassicornis* Casey, 1890b: 440. Synonymy: Davis (1982: 254).

*Blapstinus elongatus* Casey, 1890b: 441. Synonymy: Horn (1894b: 351).

*Blapstinus lepidus* Casey, 1890b: 444. Synonymy: Davis (1982: 254).

*Blapstinus aequalis* Casey, 1890b: 445. Synonymy: Davis (1982: 254).

*Blapstinus funebris* Casey, 1890b: 446. Synonymy: Davis (1982: 254).

*Blapstinus parallelus* Casey, 1890b: 448. Synonymy: Davis (1982: 254).

*Blapstinus inquisitus* Casey, 1890b: 449. Synonymy: Davis (1982: 254).

***Blapstinus domingoensis* (Marcuzzi, 1998)** DOM

*Diastolinus domingoensis* Marcuzzi, 1998b: 222.

***Blapstinus dominicus* Marcuzzi, 1962** JAM HAI PRI LAN

*Blapstinus dominicus* Marcuzzi, 1962: 34.

***Blapstinus egenus* Champion, 1885** MEX (JA SI VE) GUA NIC PAN / SA

*Blapstinus egenus* Champion, 1885: 129.

***Blapstinus emmenastoides* Champion, 1885** MEX (OA VE) GUA

*Blapstinus emmenastoides* Champion, 1885: 131.

***Blapstinus errabundus* Champion, 1885** MEX (OA VE YU) NIC PAN

*Blapstinus errabundus* Champion, 1885: 127.

***Blapstinus exiguus* Champion, 1893** MEX (OA YU)

*Blapstinus exiguus* Champion, 1893a: 529.

***Blapstinus faulkneri* Aalbu and Triplehorn, 1991** MEX (“Revillagigedo Is.”)

*Blapstinus faulkneri* Aalbu and Triplehorn, 1991: 173.

***Blapstinus fortis* LeConte, 1878** USA (AZ CO FL GA KS LA MD MO NC NM OK SC SD TX) MEX (CO JA MO OA SL VE YU) GUA BEL NIC CRI PAN / BAH CUB CAY

*Opatrinus punctulatus* Jacquelin du Val, 1857: 141 [junior secondary homonym of *Blapstinus punctulatus* Solier, 1851].

*Blapstinus fortis* LeConte, 1878a: 420. Synonymy: Casey (1890b: 429).

*Blapstinus interstitialis* Champion, 1885: 125. Replacement name for *Blapstinus punctulatus* (Jacquelin du Val, 1857).

***Blapstinus fuscus* Casey, 1890** USA (FL LA OK TX [NM]) MEX

*Blapstinus fuscus* Casey, 1890b: 427.

***Blapstinus genaroi* (Garrido, 2004)** DOM

*Diastolinus genaroi* Garrido, 2004b: 41.

***Blapstinus grandis* Champion, 1885** MEX (JA SI) NIC CRI

*Blapstinus grandis* Champion, 1885: 125.

***Blapstinus haitensis* Marcuzzi, 1962** HAI DOM

*Blapstinus haitensis* Marcuzzi, 1962: 34.

***Blapstinus hispaniolensis* (Marcuzzi, 1998)** DOM

*Diastolinus hispaniolensis* Marcuzzi, 1998b: 220.

***Blapstinus histricus* Casey, 1890** USA (AZ CA FL NV NM TX)

*Blapstinus histricus* Casey, 1890b: 433.

*Blapstinus brunneus* Casey, 1890b: 453. **New synonymy** [based on Davis (1970: 220) unpublished thesis].

*Blapstinus coronadensis* Blaisdell, 1892: 242. **New synonymy** [based on Davis (1970: 220) unpublished thesis].

***Blapstinus humilis* Casey, 1890** USA (FL) / BAH

*Blapstinus humilis* Casey, 1890b: 459.^49^

***Blapstinus inflatitibia* (Marcuzzi, 1977)** CAY

*Diastolinus inflatitibia* Marcuzzi, 1977: 17.

***Blapstinus insularis* Champion, 1885** PAN

*Blapstinus insularis* Champion, 1885: 127.

***Blapstinus intermedius* Champion, 1885** MEX GUA NIC

*Blapstinus intermedius* Champion, 1885: 129.

***Blapstinus intermixtus* Casey, 1890** CAN (BC) USA (AZ CA ID OR UT WA)

*Blapstinus intermixtus* Casey, 1890b: 451.

*Blapstinus hesperius* Casey, 1890b: 454. **New synonymy** [based on Davis (1970: 232) unpublished thesis].

***Blapstinus jamaicensis* Marcuzzi, 1962** JAM

*Blapstinus jamaicensis* Marcuzzi, 1962: 35.

***Blapstinus kalik* Steiner, 2006** BAH

*Blapstinus kalik* Steiner, 2006: 18.

***Blapstinus kaszabi* Marcuzzi, 1985** “Ciudad, Central America”

*Blapstinus kaszabi* Marcuzzi, 1985: 183.

***Blapstinus klapperichi* (Marcuzzi, 1998)** DOM

*Diastolinus klapperichi* Marcuzzi, 1998b: 219.

***Blapstinus lecontei* Mulsant and Rey, 1859** USA (CA)

*Blapstinus pubescens* LeConte, 1851: 147 [junior secondary homonym of *Blapstinus pubescens* (G.R. Waterhouse, 1845)].

*Blapstinus lecontii* Mulsant and Rey, 1859: 192. Replacement name for *Blapstinus pubescens* LeConte, 1851.

*Blapstinus cinerascens* Fall, 1929: 58. **New synonymy** [based on Davis (1970: 100) unpublished thesis].

*Blapstinus californicus* Aalbu and Triplehorn, 1991: 170 [junior primary homonym of *Blapstinus californicus* Motschulsky, 1845]. Replacement name for *Blapstinus pubescens* LeConte, 1851.

***Blapstinus longicollis* Champion, 1885** GUA NIC

*Blapstinus longicollis* Champion, 1885: 126.

***Blapstinus longipennis* Champion, 1885** MEX (SI)

*Blapstinus longipennis* Champion, 1885: 130.

***Blapstinus longulus* LeConte, 1851** USA (AZ)

*Blapstinus longulus* LeConte, 1851: 147.

***Blapstinus marcuzzii* Aalbu, new replacement name** JAM

*Blapstinus kulzeri* Marcuzzi, 1977: 28 [junior primary homonym of *Blapstinus kulzeri* Kaszab, 1969]^50^.

*Blapstinus marcuzzii* Aalbu, new replacement name for *Blapstinus kulzeri* Marcuzzi, 1977.

***Blapstinus metallicus* (Fabricius, 1801)** CAN (AB MB NB NS ON PE QC SK) USA (CO CT DE FL GA IA IL IN KS LA MA MD MI MN MO MS NC ND NE NH NJ NY OH OR PA RI SC SD VA WI WV WY)

*Blaps metallica* Fabricius, 1801a: 143.

*Opatrum interruptum* Say, 1824a: 264. Synonymy: LeConte (1866a: 61).

*Blapstinus aeneolus* Melsheimer, 1846: 66. Synonymy: LeConte (1866a: 61).

*Blapstinus luridus* Mulsant and Rey, 1859: 193. Synonymy: LeConte (1866a: 61).

***Blapstinus mexicanus* Champion, 1885** MEX (CI YU)

*Blapstinus mexicanus* Champion, 1885: 124.

***Blapstinus moestus* Melsheimer, 1846** CAN (ON) USA (CT DC DE GA IL IN MA MD MI NC NH NJ NY OH RI SC VA WI)

*Blapstinus moestus* Melsheimer, 1846: 65.

***Blapstinus nitidus* Champion, 1885** MEX (VE)

*Blapstinus nitidus* Champion, 1885: 130.

***Blapstinus obliteratus* Champion, 1885** PAN

*Blapstinus obliteratus* Champion, 1885: 132.

***Blapstinus opacus* Mulsant and Rey, 1859** ^51^ LAN

*Blapstinus opacus* Mulsant and Rey, 1859: 186.

*Blapstinus opacus martinensis* Marcuzzi, 1977: 29. Synonymy: Ivie and Hart (2016: 466).

***Blapstinus orlandoi* Ivie and Hart, 2016** JAM

*Diastolinus jamaicensis* Garrido, 2004a: 37 [junior secondary homonym of *Blapstinus jamaicensis* Marcuzzi, 1962].

*Blapstinus orlandoi* Ivie and Hart, 2016: 466. Replacement name for *Blapstinus jamaicensis* (Garrido, 2004).

***Blapstinus pacificus* Aalbu and Triplehorn, 1991** MEX (“Revillagigedo Is.”)

*Blapstinus pacificus* Aalbu and Triplehorn, 1991: 171.

***Blapstinus palmeri* Champion, 1885** MEX (CH CO NL)

*Blapstinus palmeri* Champion, 1885: 128.

***Blapstinus paradoxus* Blaisdell, 1923** MEX (SO)

*Blapstinus paradoxus* Blaisdell, 1923: 271.

***Blapstinus pimalis* Casey, 1885** USA (AZ CA CO NM NV TX UT) MEX (SO)

*Blapstinus pimalis* Casey, 1885 [January]: 185.

*Blapstinus umbrosus* Champion, 1885 [October]: 127. Synonymy: Champion (1893a: 527).

*Blapstinus niger* Casey, 1890b: 436. **New synonymy** [based on Davis (1970: 306) unpublished thesis].

*Blapstinus cribricollis* Casey, 1890b: 437. **New synonymy** [based on Davis (1970: 306) unpublished thesis].

***Blapstinus pinorum* Casey, 1914** USA (GA NC SC)

*Blapstinus pinorum* Casey, 1914: 377.

***Blapstinus pratensis* LeConte, 1859** CAN (AB) USA (CO KS MT NE NM OK SD TX) MEX (CH DU SO TA)

*Blapstinus pratensis* LeConte, 1859a: 15.

*Blapstinus arenarius* Casey, 1890b: 457. **New synonymy** [based on Davis (1970: 177) unpublished thesis].

***Blapstinus puertoricensis* (Marcuzzi, 1977)** PRI LAN

*Diastolinus puertoricensis* Marcuzzi, 1977: 20.

***Blapstinus pulverulentus* Mannerheim, 1843** USA (CA OR WA)

*Blapstinus pulverulentus* Mannerheim, 1843: 276.

*Blapstinus californicus* Motschulsky, 1845a: 77. Synonymy: Horn (1870: 355).

***Blapstinus punctatus anxius* Mulsant and Rey, 1859** [no locality given originally but probably from the Antilles]

Blapstinus punctatus var. anxius Mulsant and Rey, 1859: 190.

***Blapstinus punctatus punctatus* (Fabricius, 1792)** PRI LAN

*Blaps punctata* Fabricius, 1792a: 109.

*Diastolinus fuscicornis* Chevrolat, 1877e: viii. Synonymy: Ivie and Hart (2016: 467).

***Blapstinus puncticollis* Champion, 1893** MEX (GE)

*Blapstinus puncticollis* Champion, 1893a: 529.

***Blapstinus simulans barbadensis* Marcuzzi, 1962** LAN

*Blapstinus simulans barbadensis* Marcuzzi, 1962: 36.

***Blapstinus simulans simulans* Marcuzzi, 1954** LAN / SA

*Blapstinus simulans* Marcuzzi, 1954b: 15.

***Blapstinus striatulus* Mulsant and Rey, 1859** PRI LAN (St. Barthélemy)

*Blapstinus striatulus* Mulsant and Rey, 1859: 183.

***Blapstinus striatus* Guérin-Méneville, 1831** CUB

*Blapstinus striatus* Guérin-Méneville, 1831a: pl. 4.

**Figure 26.**
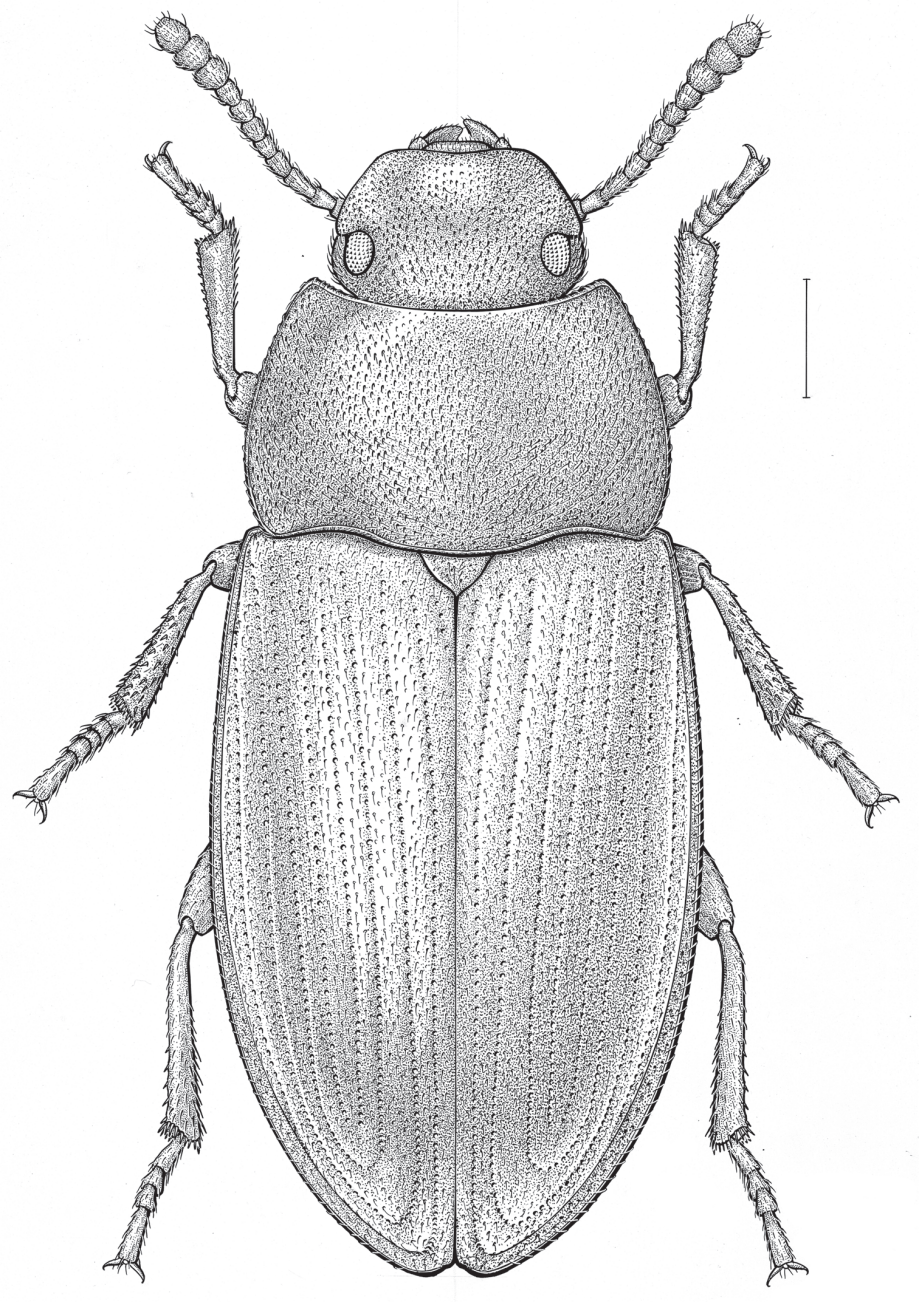
*Blapstinus substriatus* Champion, 1885. Scale bar = 1 mm.

***Blapstinus substriatus* Champion, 1885** [Fig. 26] CAN (AB BC MB SK YT) USA (AZ CA CO ID KS MN MT ND NM NV OR SD TX UT WA WY) MEX (CH CO DU FD GU ME PU SL VE) NIC

*Blapstinus substriatus* Champion, 1885: 128.

*Blapstinus gregalis* Casey, 1890b: 442. **New synonymy** [based on Davis (1970: 232) unpublished thesis].

***Blapstinus sulcatus* LeConte, 1851** USA (AZ CA CO KS NV UT)

*Blapstinus sulcatus* LeConte, 1851: 147.

*Blapstinus hydropicus* Casey, 1890b: 461. **New synonymy** [based on Davis (1970: 106) unpublished thesis].

***Blapstinus sulcipennis* Champion, 1885** MEX (YU) GUA / CUB CAY

*Blapstinus sulcipennis* Champion, 1885: 129.

***Blapstinus tibialis* Champion, 1885** GUA NIC CRI

*Blapstinus tibialis* Champion, 1885: 125.

***Blapstinus validus* Casey, 1890** USA (AZ CA)

*Blapstinus validus* Casey, 1890b: 429.

***Blapstinus vandykei* Blaisdell, 1942** USA (AZ CA NM NV TX)

*Blapstinus vandykei* Blaisdell, 1942: 136.

***Blapstinus vestitus* LeConte, 1859** USA (CO KS SD TX UT WY)

*Blapstinus vestitus* LeConte, 1859a: 15.

*Blapstinus hospes* Casey, 1890b: 455. **New synonymy** [based on Davis (1970: 187) unpublished thesis].

***Blapstinus yucatanus* Champion, 1893** MEX (YU)

*Blapstinus yucatanus* Champion, 1893a: 526.

**Genus *Bycrea* Pascoe, 1868** [F]

*Bycrea* Pascoe, 1868: xii. Type species: *Bycrea villosa* Pascoe, 1868, monotypy.

***Bycrea villosa* Pascoe, 1868** USA (AZ) MEX (DU GE GU JA MI MO NL OA PU SI SO TA YU) GUA SAL CRI / SA

*Bycrea villosa* Pascoe, 1868: xii.

**Genus *Cenophorus* Mulsant and Rey, 1859** [M]

*Cenophorus* Mulsant and Rey, 1859: 177. Type species: *Cenophorus viduus* Mulsant and Rey, 1859, monotypy.

***Cenophorus viduus* Mulsant and Rey, 1859** HAI

*Cenophorus viduus* Mulsant and Rey, 1859: 177.

**Genus *Conibiosoma* Casey, 1890** [N]

*Conibiosoma* Casey, 1890b: 476. Type species: *Conibius elongatus* Horn, 1870, monotypy.

***Conibiosoma elongatum* (Horn, 1870)** USA (CA NV)

*Conibius elongatus* Horn, 1870: 351.

**Genus *Conibius* LeConte, 1851** [M]

*Conibius* LeConte, 1851: 145. Type species: *Conibius seriatus* LeConte, 1851, subsequent designation (Lucas 1920: 199).

*Ooconibius* Casey, 1895: 618. Type species: *Notibius opacus* LeConte, 1866, monotypy. **New synonymy** [RLA].

*Euconibius* Casey, 1895: 618. Type species: *Notibius gagates* Horn, 1870, monotypy. **New synonymy** [RLA].

***Conibius brunnipes* Champion, 1885** MEX (HI JA ME MI MO NA OA PU QU SI TL) GUA

*Conibius brunnipes* Champion, 1885: 133.

***Conibius gagates* (Horn, 1870)** USA (AZ) MEX (CH SO)

*Notibius gagates* Horn, 1870: 357.

***Conibius guadalupensis* Casey, 1890** MEX (BC [Guadalupe Is.])

*Conibius guadalupensis* Casey, 1890b: 470.

***Conibius oblongus* Blaisdell, 1943** MEX (BC)

*Conibius oblongus* Blaisdell, 1943: 257.

***Conibius opacus* (LeConte, 1866)** USA (AZ) MEX (BC BS SO)

*Notibius opacus* LeConte, 1866b: 118.

*Notibius reflexus* Horn, 1894b: 429. **New synonymy** [RLA].

***Conibius rotundicollis* Linell, 1899** USA (TX) MEX (HI NL)

*Conibius rotundicollis* Linell, 1899: 182.

***Conibius rugipes* (Champion, 1885)** MEX (OA PU)

*Notibius rugipes* Champion, 1885: 132.

*Notibius affinis* Champion, 1885: 132. **New synonymy** [RLA].

***Conibius seriatus* LeConte, 1851** USA (CA OR) MEX (BC)

*Conibius seriatus* LeConte, 1851: 146.

*Conibius parallelus* LeConte, 1851: 146. **New synonymy** [RLA].

***Conibius troglodytes* Champion, 1893** MEX (GE MO PU)

*Conibius troglodytes* Champion, 1893a: 530.

***Conibius uniformis* Casey, 1890** USA (AZ NM TX) MEX (CH CO DU ZA)

*Conibius uniformis* Casey, 1890b: 471.

***Conibius ventralis* Blaisdell, 1923** MEX (BS)

*Conibius ventralis* Blaisdell, 1923: 274.

**Genus *Cybotus* Casey, 1890** [M]

*Cybotus* Casey, 1890b: 482. Type species: *Blapstinus estriatus* LeConte, 1878, monotypy.

***Cybotus estriatus* (LeConte, 1878)** USA (FL) MEX (QR) HON

*Blapstinus estriatus* LeConte, 1878a: 420.

**Genus *Diastolinus* Mulsant and Rey, 1859** [M]

*Diastolinus* Mulsant and Rey, 1859: 138. Type species: *Blaps clathrata* Fabricius, 1792, subsequent designation (Lucas 1920: 236).

*Sellio* Mulsant and Rey, 1859: 169. Type species: *Blaps tibidens* Quensel, 1806, subsequent designation (Gebien 1938: 407). Synonymy: Ivie and Hart (2016: 468).

*Ctesicles* Champion, 1896: 7. Type species: *Ctesicles insularis* Champion, 1896, subsequent designation (Lucas 1920: 214). Synonymy: Ivie and Hart (2016: 468).

***Diastolinus azuaensis* Hart and Ivie, 2016** DOM

*Diastolinus azuaensis* Hart and Ivie, 2016a: 506.

***Diastolinus clathratus* (Fabricius, 1792)** VIS (St. Croix)

*Blaps clathrata* Fabricius, 1792a: 109.

***Diastolinus chalumeaui* Hart and Ivie, 2016** LAN

*Diastolinus chalumeaui* Hart and Ivie, 2016a: 494.

***Diastolinus clavatus* Mulsant and Rey, 1859** PRI LAN

*Diastolinus clavatus* Mulsant and Rey, 1859: 155.

*Diastolinus hummelincki* Marcuzzi, 1962: 28 [junior primary homonym of *Diastolinus hummelincki* Marcuzzi, 1949]. Synonymy: Hart and Ivie (2016a: 497).

*Diastolinus mulsanti* Marcuzzi and d’Aguilar, 1971: 79. Replacement name for *Diastolinus hummelincki* Marcuzzi, 1962.

***Diastolinus coarctatus* (Mulsant and Rey, 1859)** DOM

*Sellio coarctatus* Mulsant and Rey, 1859: 170.

*Diastolinus estebani* Garrido, 2004b: 42. Synonymy: Hart and Ivie (2016a: 507).

***Diastolinus desecheo* Hart and Ivie, 2016** PRI (Desecheo Island)

*Diastolinus desecheo* Hart and Ivie, 2016a: 509.

***Diastolinus doyeni* Hart and Ivie, 2016** PRI

*Diastolinus doyeni* Hart and Ivie, 2016a: 511.

***Diastolinus espoloni* Garrido, 2007** DOM

*Diastolinus espoloni* Garrido, 2007: 46.

***Diastolinus gladiator* (Garrido, 2005)** DOM

*Sellio gladiator* Garrido, 2005: 120.

***Diastolinus hoppae* Hart and Ivie, 2016** LAN

*Diastolinus hoppae* Hart and Ivie, 2016a: 522.

***Diastolinus insularis* (Champion, 1896)** LAN (St. Vincent)

*Ctesicles insularis* Champion, 1896: 7.

***Diastolinus leewardensis* Hart and Ivie, 2016** LAN

*Diastolinus leewardensis* Hart and Ivie, 2016a: 498.

***Diastolinus maritimus* (Champion, 1896)** LAN

*Ctesicles maritimus* Champion, 1896: 8.

***Diastolinus perforatus* (Schönherr, 1806)** LAN

*Opatrum perforatum* Schönherr, 1806: 146.

***Diastolinus realinoi* Marcuzzi, 2002** [CUB]^52^

*Diastolinus realinoi* Marcuzzi, 2002: 398.

***Diastolinus shieli* Hart and Ivie, 2016** LAN (Redonda)

*Diastolinus shieli* Hart and Ivie, 2016a: 504.

***Diastolinus tibidens* (Quensel, 1806)** PRI LAN

*Blaps tibidens* Quensel [in Schönherr], 1806: 147.

***Diastolinus vaderi* Hart and Ivie, 2016** HAI

*Diastolinus vaderi* Hart and Ivie, 2016a: 518.

***Diastolinus victori* Garrido, 2002** PRI

*Diastolinus elongatus* Marcuzzi, 1977: 15 [junior primary homonym of *Diastolinus elongatus* Marcuzzi, 1976].

*Diastolinus victori* Garrido, 2002: 39. Replacement name for *Diastolinus elongatus* Marcuzzi, 1977.

**Genus *Ephalus* LeConte, 1862** [M]

*Ephalus* LeConte, 1862a: 228. Type species: *Heliopates latimanus* LeConte, 1847, monotypy.

***Ephalus latimanus* (LeConte, 1847)** CAN (NS) USA (CT MA ME NH NJ NY RI)

*Heliopates latimanus* LeConte, 1847: 92.

**Genus *Gonocephalum* Solier, 1834** [N]

*Gonocephalum* Solier, 1834: 498. Type species: *Opatrum fuscum* Herbst, 1793 (= *Opatrum rusticum* Olivier, 1811), subsequent designation (Gebien 1939: 443).

**Subgenus Gonocephalum Solier, 1834**

*Gonocephalum* Solier, 1834: 498. Type species: *Opatrum fuscum* Herbst, 1793 (= *Opatrum rusticum* Olivier, 1811), subsequent designation (Gebien 1939: 443).

***Gonocephalum sericeum* (Baudi di Selve, 1875)** USA (CA) – Adventive

*Opatrum sericeum* Baudi di Selve, 1875: 701.

**Genus *Hummelinckia* Marcuzzi, 1954** [F]

*Hummelinckia* Marcuzzi, 1954b: 19. Type species: *Hummelinckia caraibica* Marcuzzi, 1954, monotypy.

***Hummelinckia caraibica* Marcuzzi, 1954** LAN

*Hummelinckia caraibica* Marcuzzi, 1954b: 19.

**Genus *Mecysmus* Horn, 1870** [M]

*Mecysmus* Horn, 1870: 349. Type species: *Blapstinus angustus* LeConte, 1851, monotypy.

***Mecysmus advena* Casey, 1890** USA (SD TX)

*Mecysmus advena* Casey, 1890b: 466.

***Mecysmus angustus* (LeConte, 1851)** USA (AZ CA)

*Blapstinus angustus* LeConte, 1851: 147.

***Mecysmus laticollis* Casey, 1890** CAN (AB) USA (TX)

*Mecysmus laticollis* Casey, 1890b: 463.

***Mecysmus parvulus* Casey, 1890** USA (NM TX)

*Mecysmus parvulus* Casey, 1890b: 466.

***Mecysmus tenuis* Casey, 1890** USA (CA)

*Mecysmus tenuis* Casey, 1890b: 465.

**Genus *Nevisia* Marcuzzi, 1986** [F]

*Nevisia* Marcuzzi, 1985: 179. Type species: *Diastolinus barbudensis* Marcuzzi, 1962, monotypy.

***Nevisia barbudensis* (Marcuzzi, 1962)** LAN

*Diastolinus barbudensis* Marcuzzi, 1962: 29.

*Diastolinus barbudensis antiguanus* Marcuzzi, 1962: 30. Synonymy: Ivie and Hart (2016: 469).

**Genus *Nocibiotes* Casey, 1895** [M]

*Nocibiotes* Casey, 1895: 617. Type species: *Notibius granulatus* LeConte, 1851, subsequent designation (Gebien 1938: 407).

***Nocibiotes caudatus* Casey, 1895** USA (AZ) MEX (BC)

*Nocibiotes caudatus* Casey, 1895: 619, 621.

*Nocibiotes rubripes* Casey, 1895: 619. **New synonymy** [RLA].

***Nocibiotes crassipes* (Casey, 1890)** USA (CA)

*Conibius crassipes* Casey, 1890b: 475.

***Nocibiotes granulatus* (LeConte, 1851)** USA (AZ CA) MEX (BS SO)

*Notibius granulatus* LeConte, 1851: 145.

*Nocibiotes gracilis* Casey, 1895: 619. **New synonymy** [RLA].

*Nocibiotes acutus* Casey, 1895: 619, 620. **New synonymy** [RLA].

***Nocibiotes rossi* (Blaisdell, 1943)** MEX (BS)

*Tonibius rossi* Blaisdell, 1943: 260.

**Genus *Notibius* LeConte, 1851** [M]

*Notibius* LeConte, 1851: 144. Type species: *Notibius puberulus* LeConte, 1851, subsequent designation (Gebien 1938: 406).

***Notibius puberulus* LeConte, 1851** USA (AZ CA NV OR UT) MEX (BC)

*Notibius puberulus* LeConte, 1851: 145.

*Notibius substriatus* Casey, 1890b: 479. Synonymy: Horn (1894a: 41).

*Notibius laticeps* Casey, 1890b: 480. Synonymy: Horn (1894a: 41).

***Notibius puncticollis* LeConte, 1851** USA (CA NV UT)

*Notibius puncticollis* LeConte, 1851: 145.

**Genus *Opatroides* Brullé, 1832** [M]

*Opatroides* Brullé, 1832: 219. Type species: *Opatroides punctulatus* Brullé, 1832, monotypy.

***Opatroides punctulatus* Brullé, 1832** USA (CA NV) – Adventive

*Opatroides punctulatus* Brullé, 1832: 220.

**Genus *Penichrus* Champion, 1885** [M]

*Penichrus* Champion, 1885: 134. Type species: *Penichrus blapstinoides* Champion, 1885, monotypy.

***Penichrus blapstinoides* Champion, 1885** PAN

*Penichrus blapstinoides* Champion, 1885: 135.

**Genus *Platylus* Mulsant and Rey, 1859** [M]

*Platylus* Mulsant and Rey, 1859: 134. Type species: *Blaps dilatata* Fabricius, 1798, monotypy.

***Platylus dilatatus* (Fabricius, 1798)** PRI (Vieques) VIS (St. Thomas)

*Blaps dilatata* Fabricius, 1798: 47.

**Genus *Pseudephalus* Casey, 1924** [M]

*Pseudephalus* Casey, 1924: 333. Type species: *Pseudephalus brevicornis* Casey, 1924, original designation.

***Pseudephalus brevicornis* Casey, 1924** USA (AL FL) HON CRI

*Pseudephalus brevicornis* Casey, 1924: 333.

**Genus *Tonibiastes* Casey, 1895** [M]

*Tonibiastes* Casey, 1895: 617. Type species: *Notibius costipennis* Horn, 1894, original designation.

***Tonibiastes costipennis* (Horn, 1894)** MEX (BC BS)

*Notibius costipennis* Horn, 1894: 430.

**Genus *Tonibius* Casey, 1895** [M]

*Tonibius* Casey, 1895: 617. Type species: *Notibius sulcatus* LeConte, 1851, subsequent designation (Lucas 1920: 644).

***Tonibius sulcatus* (LeConte, 1851)** USA (CA) MEX (BC BS SO)

*Notibius sulcatus* LeConte, 1851: 145.

*Conibius alternatus* Casey, 1890b: 473. **New synonymy** [RLA].

**Genus *Trichoton* Hope, 1841** [N]

*Trichoton* Hope, 1841: 111. Type species: *Trichoton cayennense* Hope, 1841, original designation.

**Subgenus Trichoton Hope, 1841**

*Trichoton* Hope, 1841: 111. Type species: *Trichoton cayennense* Hope, 1841, original designation.

*Epilasium* Curtis, 1844: 222. Type species: *Epilasium rotundatum* Curtis, 1844, monotypy. Synonymy: Mulsant and Rey (1853: 245).

***Trichoton curvipes* Champion, 1885** PAN / SA

*Trichoton curvipes* Champion, 1885: 136.

***Trichoton lapidicola* Champion, 1885** NIC / SA

*Trichoton lapidicola* Champion, 1885: 136.

***Trichoton marcuzzi* Kulzer, 1961** LAN / SA

*Trichoton marcuzzi* Kulzer, 1961a: 212.

***Trichoton mexicanum* Kulzer, 1961** MEX (CL)

*Trichoton mexicanum* Kulzer, 1961a: 211.

***Trichoton sordidum* (LeConte, 1851)** USA (AZ CA NV) MEX (BC JA SO) NIC

*Blapstinus sordidus* LeConte, 1851: 146.

**Genus *Ulus* Horn, 1870** [M]

*Ulus* Horn, 1870: 358. Type species: *Blapstinus crassus* LeConte, 1851, subsequent designation (Lucas 1920: 665).

***Ulus comatus* Champion, 1893** MEX (VE)

*Ulus comatus* Champion, 1893a: 530.

***Ulus crassus* (LeConte, 1851)** USA (AZ CA UT) MEX (BS YU)

*Blapstinus crassus* LeConte, 1851: 146.

***Ulus elongatulus* Casey, 1890** USA (AZ TX)

*Ulus elongatulus* Casey, 1890b: 414.

***Ulus fimbriatus* Casey, 1890** USA (TX) MEX (CH)

*Ulus fimbriatus* Casey, 1890b: 413.

***Ulus hirsutus* Champion, 1885** MEX (CH DU JA PU SI VE YU) GUA BEL NIC CRI PAN / CAY JAM

*Ulus hirsutus* Champion, 1885: 133.

***Ulus latus* Blaisdell, 1892** USA (CA)

*Ulus latus* Blaisdell, 1892: 243.

***Ulus lineatulus* Champion, 1885** MEX (JA) GUA NIC

*Ulus lineatulus* Champion, 1885: 134.

***Ulus maritimus* Casey, 1890** USA (AL FL MS TX)

*Ulus maritimus* Casey, 1890b: 414.

***Ulus obliquus* (LeConte, 1866)** MEX (BC BS)

*Blapstinus obliquus* LeConte, 1866b: 117.

**Genus *Xerolinus* Ivie and Hart, 2016** [M]

*Xerolinus* Ivie and Hart, 2016: 470. Type species: *Diastolinus sallei* Mulsant and Rey, 1859, original designation.

***Xerolinus alfaroi* (Garrido and Gutiérrez, 1996)** CUB

*Diastolinus alfaroi* Garrido and Gutiérrez, 1996a: 228.

***Xerolinus alutaceus* (Casey, 1890)** USA (FL) / CUB

*Blapstinus opacus* LeConte, 1878a: 420 [junior primary homonym of *Blapstinus opacus* Mulsant, 1859].

*Blapstinus alutaceus* Casey, 1890b: 423. Synonymy: Casey (1890b: 423).

*Diastolinus trinitatis* Marcuzzi, 1976: 127. Synonymy: Ivie and Hart (2016: 470).

***Xerolinus armasi* (Marcuzzi, 1988)** CUB

*Diastolinus armasi* Marcuzzi, 1988: 72.

***Xerolinus bahamae* (Marcuzzi, 1965)** BAH

*Diastolinus bahamae* Marcuzzi, 1965: 125.

***Xerolinus bielawskii* (Marcuzzi, 1985)** CUB

*Diastolinus bielawskii* Marcuzzi, 1985: 182.

***Xerolinus burtoni* (Garrido and Gutiérrez, 1996)** CAY

*Diastolinus burtoni* Garrido and Gutiérrez, 1996b: 232.

***Xerolinus caguamensis* (Marcuzzi, 1988)** CUB

*Diastolinus caguamensis* Marcuzzi, 1988: 74.

***Xerolinus camanoensis* Hart and Ivie, 2016** VIS

*Xerolinus camanoensis* Hart and Ivie, 2016b: 885.

***Xerolinus caymanensis* (Marcuzzi, 1977)** CAY

*Diastolinus caymanensis* Marcuzzi, 1977: 12.

***Xerolinus cubanus* (Marcuzzi, 1962)** CUB

*Diastolinus cubanus* Marcuzzi, 1962: 30.

***Xerolinus dentipes* (Marcuzzi, 1977)** CUB CAY

*Diastolinus dentipes* Marcuzzi, 1977: 13.

*Diastolinus diformis* Marcuzzi, 1977: 14. Synonymy: Garrido and Gutiérrez (1996b: 232).

***Xerolinus difficilis* (Marcuzzi, 1976)** CUB

*Diastolinus difficilis* Marcuzzi, 1976: 126.

***Xerolinus dispar* (Casey, 1890)** USA (FL)

*Blapstinus dispar* Casey, 1890b: 424.

***Xerolinus dozieri* (Marcuzzi, 1965)** TUR

*Diastolinus dozieri* Marcuzzi, 1965: 128.

***Xerolinus elongatus* (Marcuzzi, 1976)** CUB

*Diastolinus elongatus* Marcuzzi, 1976: 126.

***Xerolinus garridoi* (Marcuzzi, 1988)** CUB

*Diastolinus garridoi* Marcuzzi, 1988: 78.

***Xerolinus hernandezi* (Marcuzzi, 1988)** CUB (Isla de Juventud)

*Diastolinus hernandezi* Marcuzzi, 1988: 72.

***Xerolinus juraguensis* (Marcuzzi, 1988)** CUB

*Diastolinus juraguensis* Marcuzzi, 1988: 75.

***Xerolinus kulzeri* (Marcuzzi, 1965)** BAH (Mayaguana)

*Diastolinus kulzeri* Marcuzzi, 1965: 127.

***Xerolinus macamboensis* (Marcuzzi, 1988)** CUB

*Diastolinus macamboensis* Marcuzzi, 1988: 77.

*Diastolinus garciai* Marcuzzi, 1988: 79. Synonymy: Garrido and Gutiérrez (1996a: 226).

***Xerolinus minor* (Marcuzzi, 1977)** CAY

*Diastolinus minor* Marcuzzi, 1977: 18.

***Xerolinus orientalis* (Garrido and Gutiérrez, 1996)** CUB

*Diastolinus orientalis* Garrido and Gutiérrez, 1996a: 226.

***Xerolinus puncticeps* (Mulsant and Rey, 1859)** CUB

*Blapstinus puncticeps* Mulsant and Rey, 1859: 181.

***Xerolinus rufoclavatus* (Zayas, 1988)** CUB

*Blapstinus rufoclavatus* Zayas, 1988: 91.

***Xerolinus sallei* (Mulsant and Rey, 1859)** HAI DOM

*Diastolinus sallei* Mulsant and Rey, 1859: 144.

*Diastolinus costipennis* Mulsant and Rey, 1859: 149. Synonymy: Ivie and Hart (2016: 473).

*Diastolinus puncticollis* Mulsant and Rey, 1859: 147. Synonymy: Ivie and Hart (2016: 473).

*Diastolinus assoi* Garrido, 2004b: 40. Synonymy: Ivie and Hart (2016: 473).

***Xerolinus smalli* (Garrido, 2004)** CUB

*Diastolinus smalli* Garrido, 2004c: 46.

***Xerolinus swearingenae* Hart and Ivie, 2016** JAM

*Xerolinus swearingenae* Hart and Ivie, 2016b: 889.

***Xerolinus that* (Steiner, 2006)** BAH

*Diastolinus that* Steiner, 2006: 25.

***Xerolinus this* (Steiner, 2006)** BAH

*Diastolinus this* Steiner, 2006: 21.

***Xerolinus waterhousii* (Mulsant and Rey, 1859)** CUB

*Diastolinus waterhousii* Mulsant and Rey, 1859: 152.

*Diastolinus kaszabi* Marcuzzi, 1976: 125. Synonymy: Ivie and Hart (2016: 474).

***Xerolinus zayasi* (Marcuzzi, 1988)** CUB

*Diastolinus zayasi* Marcuzzi, 1988: 75.

**Tribe Palorini Matthews, 2003**

Palorinae Matthews, 2003: 50. Type genus: *Palorus* Mulsant, 1854.

**Genus *Palorus* Mulsant, 1854** [M]

*Palorus* Mulsant, 1854: 250. Type species: *Hypophloeus depressus* Fabricius, 1790, monotypy.

*Caenocorse* C.G. Thomson, 1859: 117. Type species: *Hypophloeus depressus* Fabricius, 1790, original designation. Synonymy: Bedel (1906: 92).

*Eba* Pascoe, 1863: 129. Type species: *Eba cerylonoides* Pascoe, 1863, monotypy. Synonymy: Carter and Zeck (1937: 194).

*Circomus* Fleischer, 1900: 236. Type species: *Hypophloeus subdepressus* Wollaston, 1864, monotypy. Synonymy: Löbl et al. (2008a: 276).

***Palorus cerylonoides* (Pascoe, 1863)** USA (FL) / LAN – Adventive

*Eba cerylonoides* Pascoe, 1863: 129.

***Palorus genalis* Blair, 1930** BEL / LAN – Adventive

*Palorus genalis* Blair, 1930: 140.

**Figure 27.**
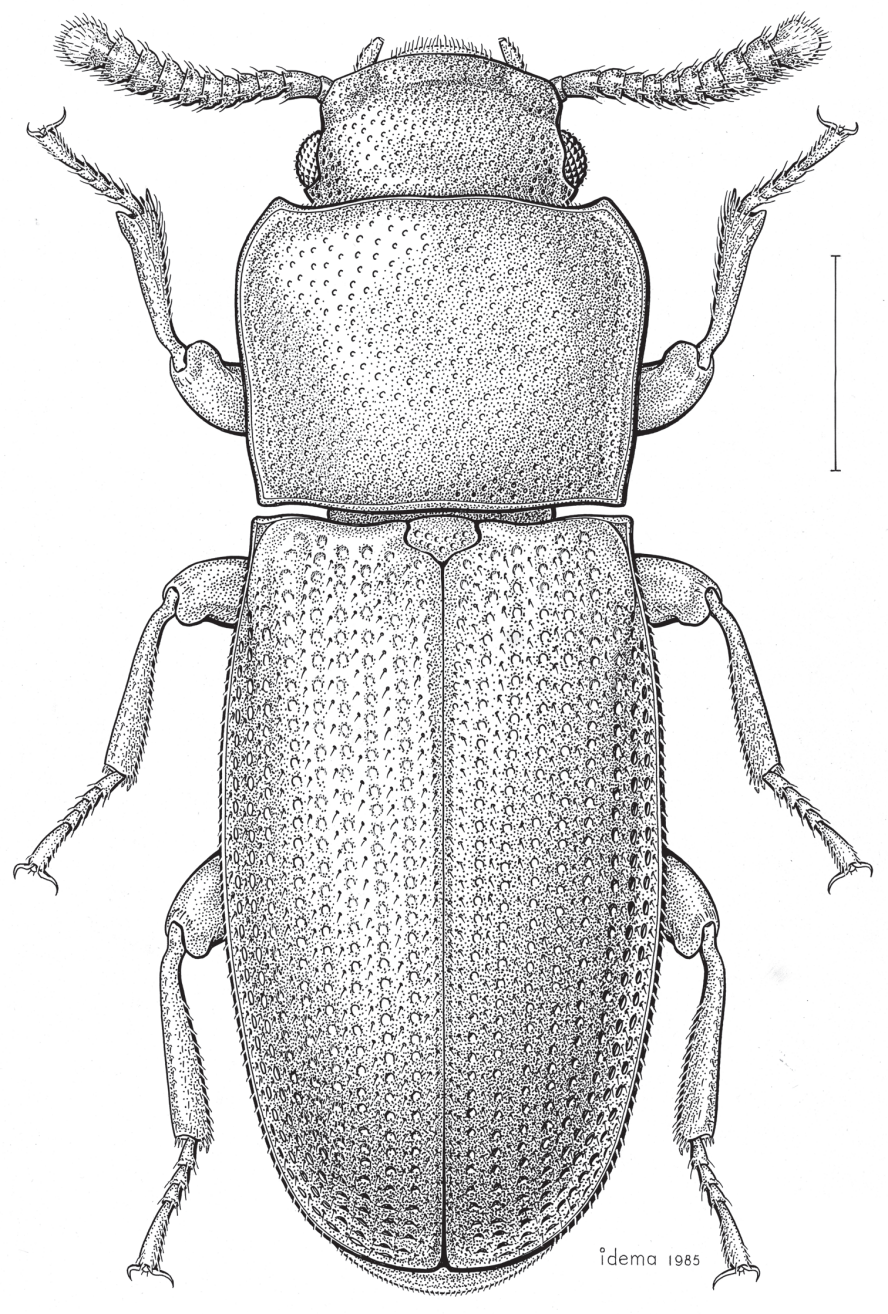
*Palorus ratzeburgii* (Wissmann, 1848). Scale bar = 1 mm.

***Palorus ratzeburgii* (Wissmann, 1848)** [Fig. 27] CAN (BC NS ON QC) USA (DC FL GA IN MI NY OH OR SC WA WI) / CUB – Adventive

*Hypophloeus ratzeburgii* Wissmann, 1848: 77.

***Palorus subdepressus* (Wollaston, 1864)** CAN (MB ON) USA (CT DC FL GA ID IN MI OH OR PA SC SD WI) MEX / LAN – Adventive

*Hypophloeus subdepressus* Wollaston, 1864: 499.

**Genus *Ulomina* Baudi di Selve, 1876** [F]

*Ulomina* Baudi di Selve, 1876: 235. Type species: *Ulomina carinata* Baudi di Selve, 1876, monotypy.

*Coelopalorus* Blair, 1930: 135. Type species: *Palorus foveicollis* Blair, 1930 (= *Ulomina carinata* Baudi di Selve, 1876), monotypy. Synonymy: Scupola (2002: 186).

***Ulomina carinata* Baudi di Selve, 1876** USA (AL FL) / CUB – Adventive

*Ulomina carinata* Baudi di Selve, 1876: 236.

*Palorus foveicollis* Blair, 1930: 136. Synonymy: Scupola (2002: 186).

**Tribe Pedinini Eschscholtz, 1829**

Pediniden Eschscholtz, 1829: 4. Type genus: *Pedinus* Latreille, 1797.

**Subtribe Leichenina Mulsant, 1854**

Leichenaires Mulsant, 1854: 179. Type genus: *Leichenum* Dejean, 1834.

**Genus *Leichenum* Dejean, 1834** [N]

*Leichenum* Dejean, 1834: 194. Type species: *Opatrum pictum* Fabricius, 1801, monotypy.

*Lichenum* Agassiz, 1846: 209. Unjustified emendation of *Leichenum* Dejean, 1834, not in prevailing usage.

***Leichenum canaliculatum variegatum* (Klug, 1833)** USA (AL FL GA MS NC SC) / BAH CUB LAN – Adventive

*Opatrum variegatum* Klug, 1833: 88.

**Subtribe Platynotina Mulsant and Rey, 1853**

Platynotaires Mulsant and Rey, 1853: 263. Type genus: *Platynotus* Fabricius, 1801.

**Genus *Alaetrinus* Iwan, 1995** [M]

*Alaetrinus* Iwan, 1995: 24. Type species: *Tenebrio pullus* Sahlberg, 1823, original designation.

***Alaetrinus aciculatus* (LeConte, 1858)** USA (CA KS NM OK TX) MEX (NL TA)

*Opatrinus aciculatus* LeConte, 1858b: 75.

***Alaetrinus minimus* (Palisot de Beauvois, 1817)** USA (AL AR CA CT DC DE FL GA IL IN KS KY LA MA MD MO MS NC NJ NY OH OK PA SC TN TX VA WV) / BAH CUB

*Tenebrio minimus* Palisot de Beauvois, 1817: 164.

*Pedinus suturalis* Say, 1824a: 263 [*nomen dubium*]. **New synonymy** [PB]^53^.

*Opatrum notum* Say, 1826: 237. Synonymy: Mulsant and Rey (1853: 309).

***Alaetrinus moestus* (Mulsant and Rey, 1853)** MEX (VE) BEL / SA

*Opatrinus moestus* Mulsant and Rey, 1853: 307.

*Opatrinus lüderwaldti* Gebien, 1928a: 112. Synonymy: Iwan (1995: 47).

***Alaetrinus pullus* (Sahlberg, 1823)** USA (FL) MEX (CI QR TB VE YU) GUA BEL HON / BER BAH CUB JAM DOM PRI LAN / SA

*Tenebrio pullus* C.R. Sahlberg, 1823: 16.

*Opatrinus anthracinus* Mulsant and Rey, 1853: 304. Synonymy: Champion (1885: 123).

*Opatrinus puertoricensis* Marcuzzi, 1977: 23. Synonymy: Iwan (1995: 38).

**Genus *Anchophthalmops* Koch, 1956** [M]

*Anchophthalmops* Koch, 1956: 173. Type species: *Anchophthalmops brevipleurum* Koch, 1956, original designation.

***Anchophthalmops menouxi* (Mulsant and Rey, 1853)** USA (KS) – Adventive

*Selinus menouxii* Mulsant and Rey, 1853: 322.

*Opatrinus sayi* Horn, 1870: 349. Synonymy: Iwan (1995: 52).

**Genus *Opatrinus* Dejean, 1821** [M]

*Opatrinus* Dejean, 1821: 66. Type species: *Opatrum clathratum* Fabricius, 1787, monotypy.

*Hopatrinus* Agassiz, 1846: 185. Unjustified emendation of *Opatrinus* Dejean, 1821, not in prevailing usage.

***Opatrinus clathratus* (Fabricius, 1787)** PAN / LAN / SA

*Opathrum clathratum* Fabricius, 1787: 379.

*Blaps gemellata* Olivier, 1795: [60] 9. Synonymy: Iwan (1995: 16).

*Helops aethiops* Fabricius, 1801a: 162. Synonymy: Gebien (1906: 212).

*Opatrinus geminatus* Erichson, 1849: 565. Synonymy: Gebien (1906: 212).

*Opatrinus gridellii* Marcuzzi, 1949: 342. Synonymy: Iwan (1995: 16).

*Opatrinus armasi* Garrido and Gutiérrez, 1994a: 121. Synonymy: Iwan (2002: 282).

***Opatrinus gibbicollis* Mulsant and Rey, 1853** PAN / SA

*Opatrinus gibbicollis* Mulsant and Rey, 1853: 303.

**Tribe Tenebrionini Latreille, 1802**

Tenebrionites Latreille, 1802: 165. Type genus: *Tenebrio* Linnaeus, 1758.

Biuini Skopin, 1978: 224. Type genus: *Bius* Dejean, 1834.

**Genus *Bius* Dejean, 1834** [M]

*Bius* Dejean, 1834: 205. Type species: *Trogosita thoracicus* Fabricius, 1792, monotypy.

***Bius estriatus* (LeConte, 1851)** CAN (AB BC YT) USA (CA ID NM MI OR WA)

*Tenebrio estriatus* LeConte, 1851: 149.

**Genus *Bouchardandrus* Steiner, 2016** [M]

*Bouchardandrus* Steiner, 2016: 543. Type species: *Haplandrus concolor* LeConte, 1866, original designation.

***Bouchardandrus concolor* (LeConte, 1866)** CAN (MB ON QC) USA (MI MN OH WI)

*Haplandrus concolor* LeConte, 1866b: 121.

**Genus *Idiobates* Casey, 1891** [M]

*Idiobates* Casey, 1891: 62. Type species: *Tenebrio castaneus* Knoch, 1801, monotypy.

***Idiobates castaneus* (Knoch, 1801)** CAN (ON QC) USA (AL AR DE FL GA IL IN KY MD MI NC NH NJ NY OH OK PA SC TN VA WI WV)

*Tenebrio castaneus* Knoch, 1801: 171.

*Tenebrio interstitialis* Say, 1824a: 266. Synonymy: Melsheimer (1853: 139).

**Genus *Neatus* LeConte, 1862** [M]

*Neatus* LeConte, 1862a: 233. Type species: *Helops tenebrioides* Palisot de Beauvois, 1812, monotypy.

***Neatus tenebrioides* (Palisot de Beauvois, 1812)** CAN (AB MB NB ON QC SK) USA (AL AR AZ CA CO CT DE FL GA IA IL IN KS KY LA MD MI MN MO MS MT NC ND NE NH NJ NM OH OK PA SC SD TN TX UT VA VT WI WV)

*Helops tenebrioïdes* Palisot de Beauvois, 1812: 121.

*Tenebrio badius* Say, 1824a: 265. Synonymy: LeConte (1859d: 156).

*Tenebrio rufinasus* Say, 1831: 8 [*nomen dubium*]. Synonymy (in doubt with *Tenebrio picipes* Herbst *sensu* North American authors = *Neatus tenebrioides*): Papp (1961d: 130, as *rufimanus*).

**Genus *Rhinandrus* LeConte, 1866** [M]

*Rhinandrus* LeConte, 1866b: 119. Type species: *Rhinandrus gracilis* LeConte, 1866, monotypy.

*Exerestus* Bates, 1870: 268. Type species: *Exerestus jansonii* Bates, 1870 (= *Rhinandrus elongatus* Horn, 1866), monotypy. Synonymy: Bates (1872: 98).

*Proderops* Fairmaire, 1873: 393. Type species: *Proderops foraminosus* Fairmaire, 1873 (= *Rhinandrus elongatus* Horn, 1866), monotypy. Synonymy (with *Exerestus* Bates): Kraatz (1880: 133).

***Rhinandrus elongatus* Horn, 1866** MEX (YU) NIC CRI

*Rhinandrus elongatus* Horn, 1866: 400.

*Exerestus jansonii* Bates, 1870: 269. Synonymy: LeConte (1873: 334).

*Proderops foraminosus* Fairmaire, 1873: 394. Synonymy: Champion (1885: 102).

***Rhinandrus foveolatus* (Kraatz, 1880)** MEX (OA)

*Proderops foveolatus* Kraatz, 1880: 133.

***Rhinandrus gracilis* LeConte, 1866** MEX (BS)

*Rhinandrus gracilis* LeConte, 1866b: 120.

***Rhinandrus helopioides* (Kraatz, 1880)** MEX (OA)

*Exerestus helopioides* Kraatz, 1880: 135.

***Rhinandrus obsoletus* Champion, 1885** MEX (DU SI)

*Rhinandrus obsoletus* Champion, 1885: 102.

**Genus *Tenebrio* Linnaeus, 1758** [M]

*Tenebrio* Linnaeus, 1758: 417. Type species: *Tenebrio molitor* Linnaeus, 1758, subsequent designation (Latreille 1810: 429).

*Menedrio* Motschulsky, 1872: 27. Type species: *Tenebrio obscurus* Fabricius, 1792, original designation. Synonymy: Heyden et al. (1883: 134).

*Tenebrionellus* Crotch, 1874: 105. Unnecessary replacement name for *Tenebrio* Linnaeus, 1758.

**Figure 28.**
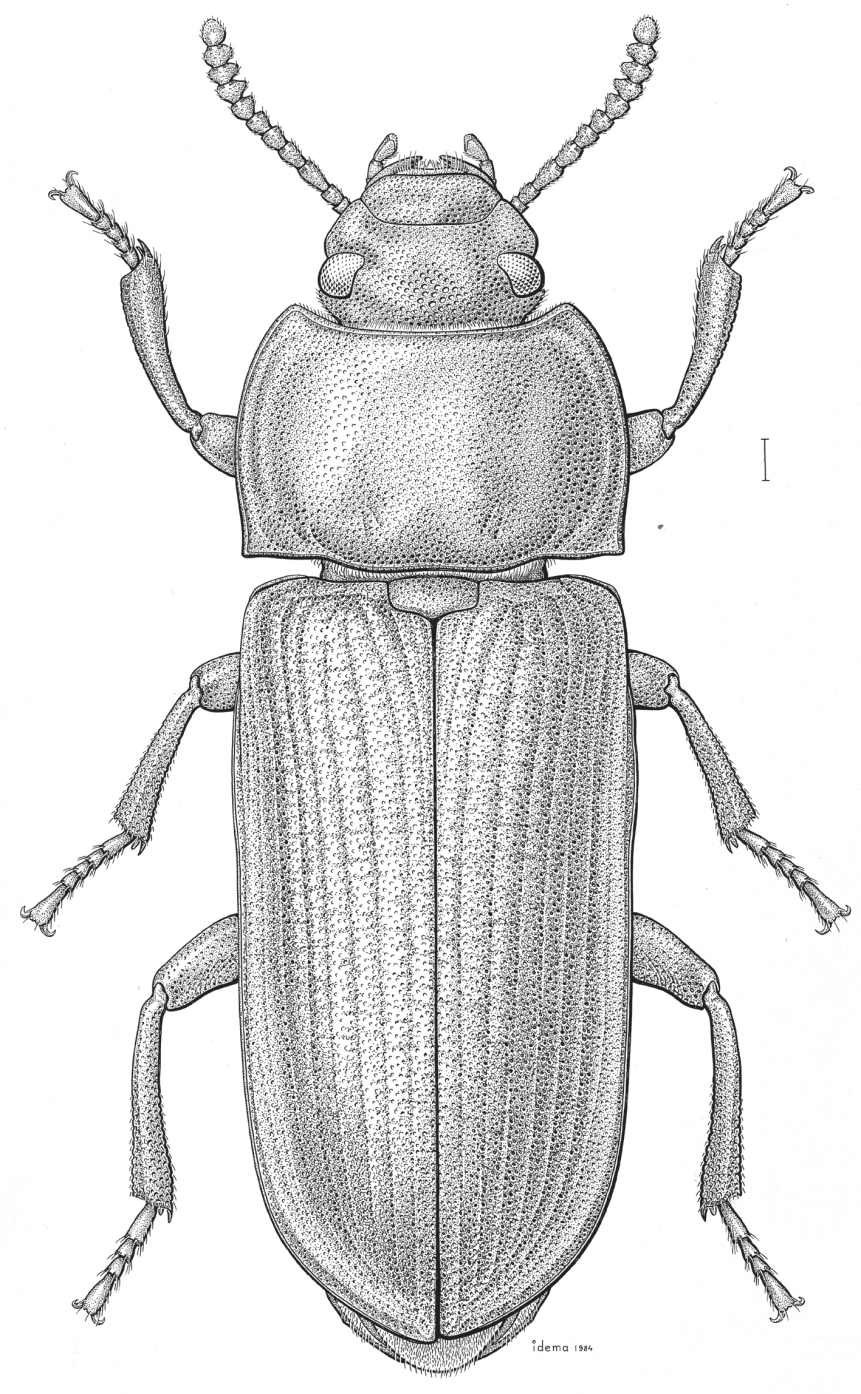
*Tenebrio molitor* Linnaeus, 1758. Scale bar = 1 mm.

***Tenebrio molitor* Linnaeus, 1758** [Fig. 28] CAN (AB BC MB NB NF NS ON PE QC SK) USA (AK FL GA ID IN MA MD MI NC OH OR SC SD WA WI) CRI / CUB PRI – Adventive

*Tenebrio molitor* Linnaeus, 1758: 417.

***Tenebrio obscurus* Fabricius, 1792** GRE CAN (AB BC NS ON QC SK) USA (FL GA ID IN MA MD MI NC OH OR SC SD WA WI) – Adventive

*Tenebrio obscurus* Fabricius, 1792a: 111.

*Menedrio longipennis* Motschulsky, 1872: 37. **New synonymy** [YB].

*Tenebrio obscurus pollens* Casey, 1924: 321. Synonymy: Bousquet and Campbell (1991: 259).

**Genus *Zophobas* Dejean, 1834** [M]

*Zophobas* Dejean, 1834 [30 June]: 204. Type species: *Helops morio* Fabricius, 1777 (= *Tenebrio atratus* Fabricius, 1775), subsequent designation (Motschulsky 1872: 26).

**Subgenus Macrozophobas Pic, 1913**

*Macrozophobas* Pic, 1913b: 6. Type species: *Macrozophobas gracilicornis* Pic, 1913 (=*Zophobas maculicollis* Kirsch, 1866), monotypy.

***Zophobas klingelhoefferi* Kraatz, 1880** MEX (VE)

*Zophobas klingelhöfferi* Kraatz, 1880: 126.

***Zophobas maculicollis* Kirsch, 1866** PAN / SA

*Zophobas maculicollis* Kirsch, 1866: 196.

*Macrozophobas gracilicornis* Pic, 1913b: 6. Synonymy: Gebien (1941: 335).

***Zophobas signatus* Champion, 1885** MEX GUA BEL HON NIC CRI PAN

*Zophobas signatus* Champion, 1885: 104.

**Subgenus Zophobas Dejean, 1834**

*Zophobas* Dejean, 1834 [30 June]: 204. Type species: *Helops morio* Fabricius, 1777 (= *Tenebrio atratus* Fabricius, 1775), subsequent designation (Motschulsky 1872: 26).

*Pythonissus* Gistel, 1834 [23 September]: 21. Type species: *Helops morio* Fabricius, 1777 (= *Tenebrio atratus* Fabricius, 1775), subsequent designation (Bousquet and Bouchard 2017a: 132). Synonymy: Bousquet and Bouchard (2017a: 132).

***Zophobas atratus* (Fabricius, 1775)** USA (CA FL) MEX (GE JA OA SL TB VE YU) GUA NIC CRI PAN / BAH CUB JAM HAI DOM PRI LAN / SA

*Tenebrio atratus* Fabricius, 1775: 256.

*Helops morio* Fabricius, 1777: 241. Synonymy: Marcuzzi and d’Aguilar (1971: 90)^54^.

*Tenebrio elongatus* Palisot de Beauvois, 1817: 164 [junior primary homonym of *Tenebrio elongatus* Herbst, 1797]. Synonymy (with *H. morio* Fabricius): Chevrolat (1853: 638).

*Zophobas rugipes* Kirsch, 1866: 197^55^. Synonymy: Tschinkel (1984: 332).

*Zophobas concolor* Wollaston, 1870: 33. Synonymy (with *H. morio* Fabricius): Champion (1896: 26).

*Zophobas alternans* Kraatz, 1880: 131. Synonymy: Ferrer (2011: 298).

*Zophobas batavorum* Marcuzzi, 1959: 88. Synonymy: Ferrer (2011: 297).

***Zophobas costatus* Pic, 1921** DOM

*Zophobas costatus* Pic, 1921a: 10.

***Zophobas diversicolor* Pic, 1921** HON

Zophobas rugipes var. diversicolor Pic, 1921a: 9.

***Zophobas macretus* Kraatz, 1880** MEX (CI ME OA TA VE YU) GUA NIC CRI

*Zophobas macretus* Kraatz, 1880: 130.

***Zophobas opacus* (Sahlberg, 1823)** USA (NM) MEX (GE VE) GUA SAL NIC CRI PAN / PRI LAN / SA

*Helops opacus* C.R. Sahlberg, 1823: 17.

*Zophabas* [sic!] *subnitidus* Motschulsky, 1872: 35. Synonymy: Ferrer (2011: 301).

*Zophabas* [sic!] *laticollis* Motschulsky, 1872: 36. Synonymy: Ferrer (2011: 301).

*Zophobas ambiguus* Kraatz, 1880: 124. Synonymy: Ferrer (2011: 301).

*Zophobas kraatzi* Champion, 1885: 105. Synonymy: Ferrer (2011: 301).

*Zophobas diversipes* Pic, 1921a: 8. Synonymy: Ferrer (2011: 301).

*Zophobas cubanus* Marcuzzi, 1976: 128. Synonymy: Ferrer (2011: 301).

***Zophobas tridentatus* Kraatz, 1880** NIC PAN / SA

*Zophobas tridentatus* Kraatz, 1880: 124.

*Zophobas kirschi* Kraatz, 1880: 127. Synonymy: Ferrer (2011: 299).

*Zophobas pedestris* Champion, 1885: 103. Synonymy: Ferrer (2011: 299).

*Zophobas elongatior* Pic, 1921a: 9. Synonymy: Ferrer (2011: 299).

[incertae sedis]

***Zophobas subnitens* (Horn, 1874)** USA (AZ) MEX (SO)

*Nyctobates subnitens* Horn, 1874a: 35.

*Rhinandrus sublaevis* Horn, 1885c: 160. Synonymy: Spilman (1962b: 60).

**Tribe Toxicini Oken, 1843**

Toxiciden Oken, 1843: 484. Type genus: *Toxicum* Latreille, 1802.

**Subtribe Dysantina Gebien, 1922**

Dysantinae Gebien, 1922: 289. Type genus: *Dysantes* Pascoe, 1869.

Eudysantina Bouchard, Lawrence, Davies and Newton, 2005: 508. Type genus: *Eudysantes* Bouchard et al., 2005 (= *Dysantes* Pascoe, 1869). Note. *Dysantes* Pascoe was first made available in 1869 [on 1 January], not in 1871 as previously noted. The name is a senior homonym of the ichneumonid *Dysantes* Förster, 1869 [May], which was incorrectly dated 1868. The replacement name of the family-group name proposed by Bouchard et al. (2005) was thus unnecessary. A full explanation about this case will be issued in a forthcoming publication by YB and PB.

**Genus *Diceroderes* Solier, 1841** [M]

*Diceroderes* Solier, 1841: 30, 46. Type species: *Diceroderes mexicanus* Solier, 1841, original designation.

*Prosomenes* Blanchard, 1845: 10. Type species: *Diceroderes mexicanus* Solier, 1841, subsequent monotypy (Chevrolat 1847: 562). Note. The generic name *Prosomenes* was listed as a junior synonym of Dicérodères by Blanchard (1845: 10). However, because the name was treated before 1961 as an available name and adopted as the name of a taxon (e.g., Chevrolat 1847: 562), it is made available thereby but dates from its first publication as a synonym (ICZN 1999: Article 11.6.1).

***Diceroderes cusucoensis* Smith, 2015** GUA HON

*Diceroderes cusucoensis* Smith [in Smith and Cifuentes-Ruiz], 2015: 62.

***Diceroderes mexicanus* Solier, 1841** MEX (HI PU VE)

*Diceroderes mexicanus* Solier, 1841: 49.

***Diceroderes ocozocoautlaensis* Smith, 2015** MEX (CI)

*Diceroderes ocozocoautlaensis* Smith [in Smith and Cifuentes-Ruiz], 2015: 63.

***Diceroderes skelleyi* Smith, 2015** GUA

*Diceroderes skelleyi* Smith [in Smith and Cifuentes-Ruiz], 2015: 65.

***Diceroderes subtriplehorni* Smith and Cifuentes-Ruiz, 2015** MEX (OA PU VE)

*Diceroderes subtriplehorni* Smith and Cifuentes-Ruiz, 2015: 67.

**Genus *Ozolais* Pascoe, 1866** [F]

*Ozolais* Pascoe, 1866: 457. Type species: *Ozolais scruposa* Pascoe, 1866, monotypy.

***Ozolais elongata* Champion, 1886** NIC PAN

*Ozolais elongata* Champion, 1886: 228.

***Ozolais lutosa* Champion, 1886** CRI

*Ozolais lutosa* Champion, 1886: 227.

***Ozolais nodosa* Champion, 1886** NIC

*Ozolais nodosa* Champion, 1886: 228.

***Ozolais tuberculifera* Champion, 1896** LAN

*Ozolais tuberculifera* Champion, 1896: 10.

***Ozolais verrucosa* Champion, 1886** PAN

*Ozolais verrucosa* Champion, 1886: 226.

**Genus *Wattius* Kaszab, 1982** ^56^ [M]

*Wattius* Kaszab, 1982: 50. Type species: *Calymmus cucullatus* Pascoe, 1871, original designation.

***Wattius andersoni* Smith and Sanchez, 2015** CUB

*Wattius andersoni* Smith and Sanchez, 2015: 118.

***Wattius emmabaconae* Smith and Sanchez, 2015** DOM

*Wattius emmabaconae* Smith and Sanchez, 2015: 121.

***Wattius variegatus* (Champion, 1886)** NIC

*Calymmus variegatus* Champion, 1886: 225.

***Wattius viatorus* Smith and Sanchez, 2015** BAH CUB

*Wattius viatorus* Smith and Sanchez, 2015: 125.

**Tribe Triboliini Gistel, 1848**

Triboliidae Gistel, 1848: [4]. Type genus: *Tribolium* MacLeay, 1825.

**Genus *Aesymnus* Champion, 1886** [M]

*Aesymnus* Champion, 1886: 168. Type species: *Aesymnus nitidus* Champion, 1886, monotypy.

***Aesymnus nitidus* Champion, 1886** MEX (VE) PAN

*Aesymnus nitidus* Champion, 1886: 168.

**Genus *Hypogena* Dejean, 1834** [F]

*Hypogena* Dejean, 1834: 199. Type species: *Tenebrio biimpressus* Latreille, 1813, monotypy.

*Ulosonia* Laporte, 1840: 220. Type species: *Uloma tricornis* Laporte, 1840 (= *Phaleria tricornis* Dalman, 1823), subsequent designation (Gebien 1940: 786). Synonymy: Jacquelin du Val (1857: 148).

***Hypogena biimpressa* (Latreille, 1813)** MEX (CI DU JA OA PU SI TA VE YU) GUA BEL NIC PAN / CUB DOM LAN / SA

*Tenebrio biimpressus* Latreille, 1813: 17.

***Hypogena canaliculata* (Champion, 1886)** NIC CRI PAN

*Ulosonia canaliculata* Champion, 1886: 164.

***Hypogena dejeani* (Champion, 1886)** GUA / SA

*Ulosonia dejeani* Champion, 1886: 165.

***Hypogena depressa* (Champion, 1886)** MEX (MO)

*Ulosonia depressa* Champion, 1886: 164.

***Hypogena marginata* (LeConte, 1851)** USA (AZ CA) MEX (BS)

*Uloma marginata* LeConte, 1851: 149.

***Hypogena tricornis* (Dalman, 1823)** USA (FL TX) MEX (BS JA OA PU VE YU) GUA BEL NIC CRI / BAH CUB CAY / SA

*Phaleria tricornis* Dalman, 1823: 59.

*Ulosonia tricornis* Laporte, 1840: 220. Synonymy: Spilman (1973: 42)^57^.

**Genus *Latheticus* C.O. Waterhouse, 1880** [M]

*Latheticus* C.O. Waterhouse, 1880: 147. Type species: *Latheticus oryzae* C.O. Waterhouse, 1880, monotypy.

**Figure 29.**
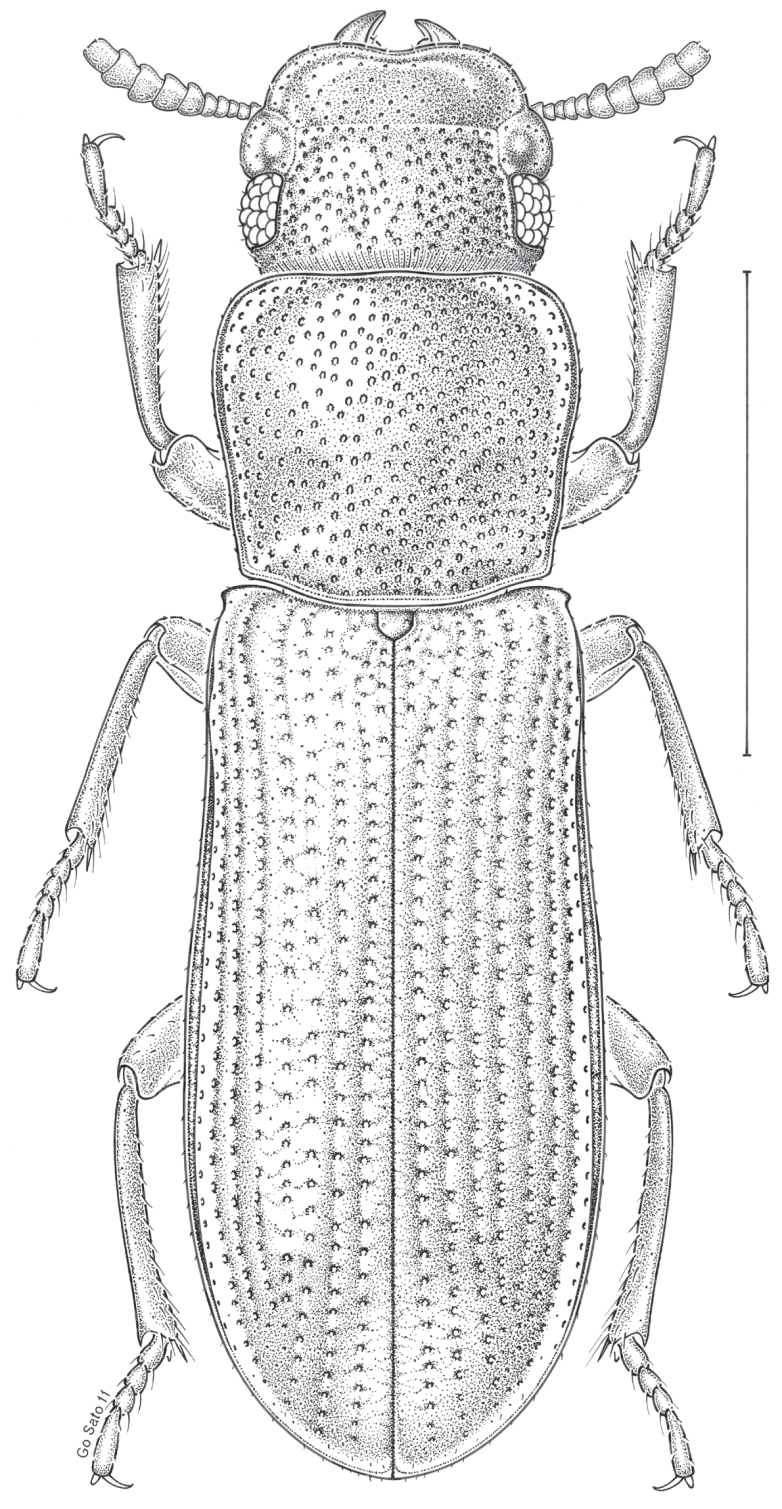
*Latheticus oryzae* C.O. Waterhouse, 1880. Scale bar = 1 mm.

***Latheticus oryzae* C.O. Waterhouse, 1880** [Fig. 29] CAN (AB MB NB QC SK) USA (FL GA MD MI NC OH SC TX) / CUB HIS – Adventive

*Latheticus oryzae* C.O. Waterhouse, 1880: 148.

***Latheticus prosopis* Chittenden, 1904** USA (AZ CA FL) MEX (BS)

*Latheticus prosopis* Chittenden, 1904: 167.

**Genus *Lyphia* Mulsant and Rey, 1859** [F]

*Lyphia* Mulsant and Rey, 1859: 166. Type species: *Lyphia ficicola* Mulsant and Rey, 1859 (=*Bius tetraphyllus* Fairmaire, 1857), monotypy.

***Lyphia tetraphylla* (Fairmaire, 1857)** USA (DC FL GA MD OH) – Adventive

*Bius tetraphyllus* Fairmaire, 1857: 534.

*Lyphia ficicola* Mulsant and Rey, 1859: 166. Synonymy: Marseul (1876: 113).

*Hypophloeus rugosus* Dury, 1902: 171. Synonymy (with *L. ficicola* Mulsant and Rey): Schwarz [in Dury] (1902: [198]).

**Genus *Metulosonia* Bates, 1873** [F]

*Metulosonia* Bates, 1873d: 261. Type species: *Metulosonia horni* Bates, 1873, subsequent designation (Gebien 1940: 1061).

***Metulosonia horni* Bates, 1873** PAN

*Metulosonia horni* Bates, 1873d: 262.

***Metulosonia reflexa* (Chevrolat, 1878)** MEX (VE) GUA BEL NIC

*Peltoides reflexus* Chevrolat, 1878c: 237.

**Genus *Mycotrogus* Horn, 1870** [M]

*Mycotrogus* Horn, 1870: 367. Type species: *Mycotrogus piceus* Horn, 1870, subsequent designation (Lucas 1920: 427).

***Mycotrogus angustus* Horn, 1870** USA (AZ CA)

*Mycotrogus angustus* Horn, 1870: 368.

***Mycotrogus mentalis* Blaisdell, 1923** USA (AZ) MEX (BC BS)

*Mycotrogus mentalis* Blaisdell, 1923: 279.

***Mycotrogus paripunctatus* Spilman, 1963** CUB

*Mycotrogus paripunctatus* Spilman, 1963: 23.

***Mycotrogus piceus* Horn, 1870** USA (CA)

*Mycotrogus piceus* Horn, 1870: 367.

**Genus *Spelaebiosis*** ^58^ **Bousquet and Bouchard, new replacement name** [F]

*Orghidania* Ardoin, 1977b: 383 [junior homonym of *Orghidania* Capuse, 1971]. Type species: *Orghidania torrei* Ardoin, 1977, monotypy.

*Ardoinia* Özdikmen, 2004: 202 [junior homonym of *Ardoinia* Kaszab, 1969]. Replacement name for *Orghidania* Ardoin, 1977.

*Spelaebiosis* Bousquet and Bouchard, new replacement name for *Ardoinia* Özdikmen, 2004.

***Spelaebiosis torrei* (Ardoin, 1977)** CUB

*Orghidania torrei* Ardoin, 1977b: 384.

**Genus *Tribolium* MacLeay, 1825** [N]

*Tribolium* MacLeay, 1825: 47. Type species: *Colydium castaneum* Herbst, 1797, monotypy.

**Subgenus Aphanotus LeConte, 1862**

*Aphanotus* LeConte, 1862a: 233. Type species: *Eulabis brevicornis* LeConte, 1859, original designation.

***Tribolium brevicorne* (LeConte, 1859)** CAN (BC) USA (CA OR WA)

*Eulabis brevicornis* LeConte, 1859b: 78.

***Tribolium parallelum* (Casey, 1890)** USA (AZ) MEX (CL)

*Aphanotus parallelus* Casey, 1890b: 483.

***Tribolium setosum* Triplehorn, 1978** USA (AZ)

*Tribolium setosum* Triplehorn, 1978: 73.

**Subgenus Tribolium MacLeay, 1825** ^59^

*Tribolium* MacLeay, 1825: 47. Type species: *Colydium castaneum* Herbst, 1797, monotypy.

*Stene* Stephens, 1829: 19. Type species: *Tenebrio ferrugineus* Fabricius *sensu auctorum* (= *Colydium castaneum* Herbst, 1797), monotypy. Synonymy: Shuckard (1840: vii).

*Margus* Dejean, 1834: 200. Type species: *Tenebrio ferrugineus* Fabricius *sensu auctorum* (= *Colydium castaneum* Herbst, 1797), monotypy. Synonymy: Guérin-Méneville (1846: cxvii).

***Tribolium audax* Halstead, 1969** CAN (AB BC MB ON QC SK) USA (FL MI MN OH PA SD UT VA)

*Tribolium audax* Halstead, 1969: 296.

***Tribolium castaneum* (Herbst, 1797)** CAN (AB BC MB NB NS ON PE QC SK) USA (AL CA FL GA ID IL IN MA MD MI MS NC NY OH OR PA SC SD TN VA WA WI) MEX (BS CO GE NL OA) GUA NIC PAN / CUB CAY JAM HAI DOM PRI LAN / SA – Adventive

*Dermestes navalis* Fabricius, 1775: 56. Note. This name was suppressed for the purposes of the Principle of Priority (ICZN 1987).

*Colydium castaneum* Herbst, 1797: 282. Synonymy: Schönherr (1806: 153).

**Figure 30.**
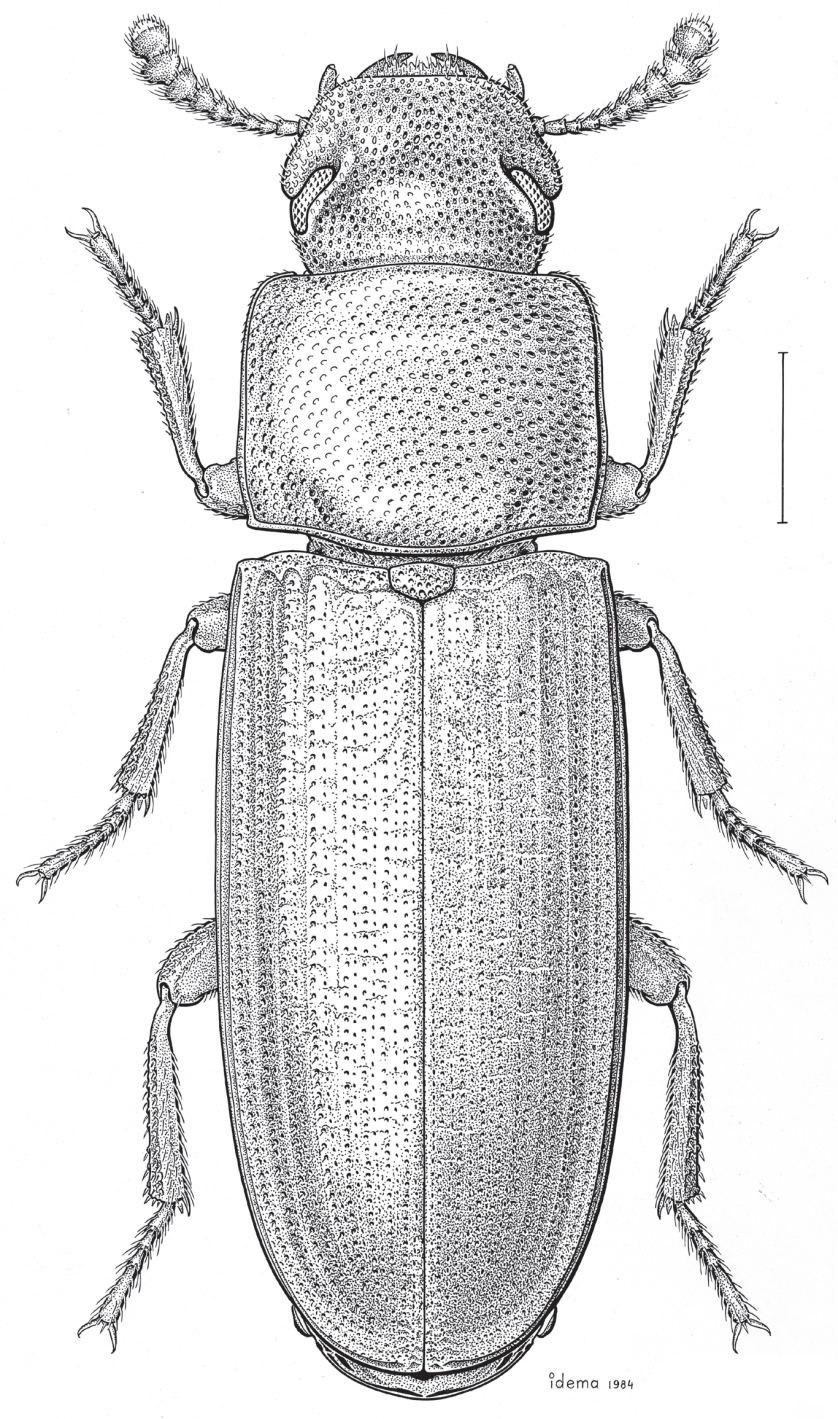
Tribolium (Tribolium) confusum Jacquelin du Val, 1862. Scale bar = 1 mm.

***Tribolium confusum* Jacquelin du Val, 1862** [Fig. 30] CAN (AB BC MB NB NF NS ON PE QC SK) USA (AK CT FL GA ID IL IN MD MI NC NJ NY OH OR PA SC SD VA WA WI) MEX (CO GU NL) PAN / CUB JAM HAI DOM PRI / SA – Adventive

*Tribolium confusum* Jacquelin du Val, 1862: 181.

***Tribolium destructor* Uyttenboogaart, 1934** GRE CAN (AB BC MB NB NF NS ON PE QC SK YT) USA (CA WA WI) – Adventive

*Tribolium destructor* Uyttenboogaart, 1934: 21.

***Tribolium madens* (Charpentier, 1825)** CAN (MB NB NS ON QC) USA (KY MD MI NM PA) – Adventive

*Tenebrio madens* Charpentier, 1825: 218.

***Tribolium linsleyi* Hinton, 1948** MEX (CL)

*Tribolium linsleyi* Hinton, 1948: 32.

**Tribe Ulomini Blanchard, 1845**

Ulomites Blanchard, 1845: 16. Type genus: *Uloma* Dejean, 1821.

Alégoriides Lacordaire, 1859: 325. Type genus: *Alegoria* Laporte, 1840.

**Genus *Alegoria* Laporte, 1840** [F]

*Alegoria* Laporte, 1840: 221. Type species: *Alegoria dilatata* Laporte, 1840, monotypy.

***Alegoria castelnaui* Fleutiaux and Sallé, 1890** ^60^ LAN

*Allegoria* [sic!] *castelnaui* Fleutiaux and Sallé, 1890: 425.

***Alegoria dilatata* Laporte, 1840** MEX GUA HON NIC PAN / LAN / SA

*Alegoria dilatata* Laporte, 1840: 221.

***Alegoria sallei* Bates, 1873** MEX (OA VE)

*Alegoria sallei* Bates, 1873a: 181.

*Alegoria sallaei* Champion, 1886: 149. Unjustified emendation of *Alegoria sallei* Bates, 1873, not in prevailing usage.

**Genus *Antimachus* Gistel, 1829** [M]

*Antimachus* Gistel, 1829: 1055. Type species: *Phaleria furcifera* Dalman, 1821, monotypy.

*Ceratupis* Perty, 1830: 57. Type species: *Ceratupis nigerrima* Perty, 1830, monotypy. Synonymy: Lacordaire (1859: 330).

***Antimachus ardoini* Chalumeau, 1982** LAN (Martinique)

*Antimachus ardoini* Chalumeau, 1982: 188.

***Antimachus coriaceus* Lacordaire, 1859** NIC PAN / SA

*Antimachus coriacea* Lacordaire, 1859: 331.

***Antimachus roudeni* Fleutiaux and Sallé, 1890** LAN

*Antimachus roudeni* Fleutiaux and Sallé, 1890: 426.

**Genus *Eutochia* LeConte, 1862** [F]

*Eutochia* LeConte, 1862a: 238. Replacement name for *Aniara* Lacordaire, 1859.

**Subgenus Eutochia LeConte, 1862**

*Aniara* Lacordaire, 1859: 336 [junior homonym of *Aniara* Hope, 1838]. Type species: *Uloma picea* Melsheimer, 1846, monotypy.

*Eutochia* LeConte, 1862a: 238. Replacement name for *Aniara* Lacordaire, 1859.

*Delopygus* LeConte, 1866b: 129. Type species: *Delopygus crenatus* LeConte, 1866, monotypy. Synonymy: Horn (1870: 372).

*Aniarus* Gemminger [in Gemminger and Harold], 1870: 1964. Unjustified emendation of *Aniara* Lacordaire, 1859, not in prevailing usage.

***Eutochia crenata* (LeConte, 1866)** USA (TX)

*Delopygus crenatus* LeConte, 1866b: 130.

**Figure 31.**
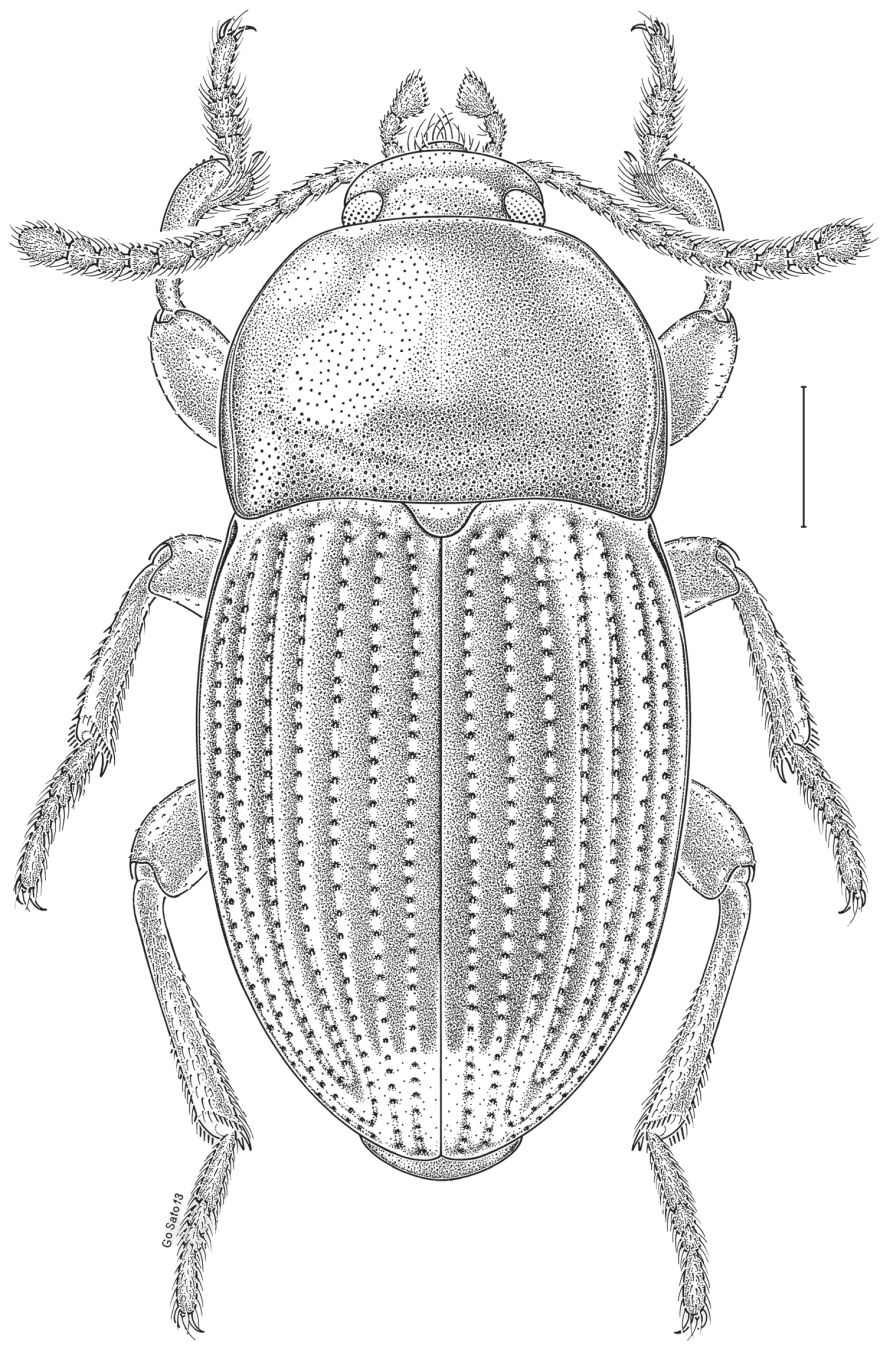
Eutochia (Eutochia) picea (Melsheimer, 1846). Scale bar = 1 mm.

***Eutochia picea* (Melsheimer, 1846)** [Fig. 31] USA (AL AR DC FL GA IL IN KY MD MO NC NJ NY OH OK PA SC TN TX VA WV)

*Uloma picea* Melsheimer, 1846: 64.

**Genus *Pheres* Champion, 1886** [M]

*Pheres* Champion, 1886: 150. Type species: *Pheres batesi* Champion, 1886, monotypy.

***Pheres batesi* Champion, 1886** PAN

*Pheres batesi* Champion, 1886: 150.

**Genus *Uleda* Laporte, 1840** [F]

*Uleda* Laporte, 1840: 220. Type species: *Uloma diaperoides* Laporte, 1840, monotypy.

***Uleda tarsalis* (Perroud and Mulsant, 1856)** MEX / SA^61^

*Melasia tarsalis* Perroud and Mulsant, 1856: 163.

*Uleda grossa* Champion, 1886: 151. Synonymy: Gebien (1940: 771).

**Genus *Uloma* Dejean, 1821** [F]

*Uloma* Dejean, 1821: 67. Type species: *Tenebrio culinaris* Linnaeus, 1758 (see ICZN 1975).

***Uloma antillarum* Champion, 1896** LAN

*Uloma antillarum* Champion, 1896: 22.

***Uloma armata* Champion, 1886** GUA BEL

*Uloma armata* Champion, 1886: 154.

***Uloma carolynae* Doyen, 1985** MEX (CI)

*Uloma carolynae* Doyen, 1985a: 518.

***Uloma divergens* Champion, 1886** GUA

*Uloma divergens* Champion, 1886: 155.

***Uloma extraordinaria* Spilman, 1961** CUB

*Uloma extraordinaria* Spilman, 1961b: 113.

***Uloma fossulata* Champion, 1886** MEX (VE) GUA BEL

*Uloma fossulata* Champion, 1886: 153.

***Uloma grenadensis* Champion, 1896** LAN

*Uloma grenadensis* Champion, 1896: 23.

***Uloma imberbis* LeConte, 1866** USA (AL AR DE FL GA IA IL IN KS KY LA MD MI MO NC NJ NY OH OK SC TN TX VA)

*Uloma imberbis* LeConte, 1866b: 123.

***Uloma impressa* Melsheimer, 1846** CAN (ON) USA (AL FL GA IA IL IN LA MA MD MI NC NE NH NJ NY OH PA RI SC VA WI)

*Uloma impressa* Melsheimer, 1846: 64.

***Uloma laevicollis* Champion, 1886** GUA NIC CRI PAN

*Uloma laevicollis* Champion, 1886: 153.

***Uloma longula* LeConte, 1861** CAN (BC) USA (CA OR WA)

*Uloma longula* LeConte, 1861b: 353.

***Uloma mentalis* Horn, 1870** CAN (ON) USA (AL AR FL GA IN KS MD MI MS NC NY OH SC TN TX WI)

*Uloma mentalis* Horn, 1870: 371.

***Uloma mexicana* (Lacordaire, 1859)** MEX (VE) GUA BEL SAL NIC CRI

*Antimachus mexicana* Lacordaire, 1859: 331.

***Uloma moensis* Marcuzzi, 2000** CUB

*Uloma moensis* Marcuzzi, 2000: 286.

***Uloma parvula* Champion, 1896** LAN

*Uloma parvula* Champion, 1896: 23.

***Uloma punctulata* LeConte, 1866** USA (FL GA IN LA MD MI MS NC NY OH SC TN TX VA WI)

*Uloma punctulata* LeConte, 1866b: 124.^62^

*Uloma cava* LeConte, 1866b: 124. Synonymy: Horn (1870: 372).

***Uloma retusa ephippigera* (Guérin-Méneville, 1831)** MEX (VE) BEL NIC CRI PAN / LAN / SA

*Phaleria ephippiger* Guérin-Méneville, 1831b: pl. 2.

*Uloma bicolor* Kirsch, 1874: 403. Synonymy: Gebien (1928a: 160).

Uloma retusa var. dimidiata Champion, 1886: 154. Synonymy (with *U. bicolor* Kirsch): Gebien (1911a: 403).

***Uloma retusa retusa* (Fabricius, 1801)** MEX (JA QR SI VE YU) / PRI LAN / SA

*Tenebrio retusus* Fabricius, 1801a: 149.

***Uloma rubens* Laporte, 1840** “Amérique du Nord”

*Uloma rubens* Laporte, 1840: 220^63^.

***Uloma spinipes* Champion, 1886** GUA

*Uloma spinipes* Champion, 1886: 155.

***Uloma sulcata* Champion, 1896** LAN

*Uloma sulcata* Champion, 1896: 21.

**Subfamily ALLECULINAE Laporte, 1840**

Alléculites Laporte, 1840: 242. Type genus: *Allecula* Fabricius, 1801.

**Tribe Alleculini Laporte, 1840**

Alléculites Laporte, 1840: 242. Type genus: *Allecula* Fabricius, 1801.

**Subtribe Alleculina Laporte, 1840**

Alléculites Laporte, 1840: 242. Type genus: *Allecula* Fabricius, 1801.

Upinellae LeConte, 1866b: 137. Type genus: *Upinella* Mulsant, 1856.

**Genus *Aeanes* Champion, 1893** [M]

*Aeanes* Champion, 1893a: 566. Type species: *Aeanes angusticollis* Champion, 1893, monotypy.

***Aeanes angusticollis* Champion, 1893** MEX (GE)

*Aeanes angusticollis* Champion, 1893a: 567.

**Genus *Alethia* Champion, 1888** [F]

*Alethia* Champion, 1888: 417. Type species: *Alethia sallaei* Champion, 1888, original designation.

***Alethia azteca* Champion, 1888** MEX (GU)

*Alethia azteca* Champion, 1888: 418.

***Alethia carbonaria* Schaeffer, 1905** USA (AZ)

*Alethia carbonaria* Schaeffer, 1905b: 176.

*Hymenorus liebecki* Fall, 1931b: 245. Synonymy: Marshall (1970b: 1).

***Alethia funerea* Champion, 1888** MEX (GE)

*Alethia funerea* Champion, 1888: 419.

***Alethia hoegei* Champion, 1888** MEX (CH)

*Alethia högei* Champion, 1888: 420.

***Alethia lepturoides* Champion, 1888** MEX

*Alethia lepturoides* Champion, 1888: 419.

***Alethia longipennis* Champion, 1888** MEX (AG)

*Alethia longipennis* Champion, 1888: 418.

***Alethia nitidipennis* Champion, 1893** MEX (GE)

*Alethia nitidipennis* Champion, 1893a: 565.

***Alethia quadricollis* (Fall, 1931)** USA (TX)

*Hymenorus quadricollis* Fall, 1931b: 246.

***Alethia sallaei* Champion, 1888** MEX (GU)

*Alethia sallaei* Champion, 1888: 417.

***Alethia subnitida* Champion, 1888** MEX (GE JA)

*Alethia subnitida* Champion, 1888: 418.

**Genus *Allecula* Fabricius, 1801** [F]

*Allecula* Fabricius, 1801b: 21. Type species: *Allecula morio* Fabricius, 1801, subsequent designation (Duponchel 1840: 283).

***Allecula angustata* Champion, 1888** MEX (HI MO)

*Allecula angustata* Champion, 1888: 416.

***Allecula belti* Champion, 1888** NIC

*Allecula belti* Champion, 1888: 414.

***Allecula brachyptera* Doyen, 1990** MEX (JA)

*Allecula brachyptera* Doyen, 1990: 241.

***Allecula caribea* Campbell, 1971** PRI

*Allecula caribea* Campbell, 1971: 67.

***Allecula castaneipennis* Champion, 1888** CRI PAN / SA

*Allecula castaneipennis* Champion, 1888: 412.

***Allecula depressa* Champion, 1888** MEX (OA)

*Allecula depressa* Champion, 1888: 415.

***Allecula ferox* Champion, 1888** GUA

*Allecula ferox* Champion, 1888: 413.

***Allecula gaumeri* Champion, 1888** MEX (YU)

*Allecula gaumeri* Champion, 1888: 414.

***Allecula inconspicua* Borchmann, 1937** MEX (VE)

*Allecula inconspicua* Borchmann, 1937: 212.

***Allecula laticeps* Champion, 1888** MEX (OA)

*Allecula laticeps* Champion, 1888: 416.

***Allecula opacipennis* Champion, 1888** MEX (OA)

*Allecula opacipennis* Champion, 1888: 415.

***Allecula pilipes* Champion, 1888** MEX (VE)

*Allecula pilipes* Champion, 1888: 414.

***Allecula ramosi* Campbell, 1971** DOM PRI

*Allecula ramosi* Campbell, 1971: 66.

***Allecula rugicollis* Champion, 1888** MEX (GE JA)

*Allecula rugicollis* Champion, 1888: 412.

***Allecula veraepacis* Champion, 1888** GUA

*Allecula veraepacis* Champion, 1888: 413.

**Genus *Amaropsis* Champion, 1893** [F]

*Amaropsis* Champion, 1893a: 567. Type species: *Amaropsis annulicornis* Champion, 1893, monotypy.

***Amaropsis annulicornis* Champion, 1893** MEX (VE)

*Amaropsis annulicornis* Champion, 1893a: 568.

**Genus *Charisius* Champion, 1888** [M]

*Charisius* Champion, 1888: 421. Type species: *Charisius fasciatus* Champion, 1888, subsequent designation (Lucas 1920: 178).

*Narses* Champion, 1888: 423. Type species: *Narses subalatus* Champion, 1888, monotypy. Synonymy: Campbell (2014a: 271).

***Charisius apterus* Campbell, 2014** MEX (OA)

*Charisius apterus* Campbell, 2014a: 278.

***Charisius fasciatus* Champion, 1888** MEX (CI) GUA SAL HON

*Charisius fasciatus* Champion, 1888: 421.

***Charisius granulatus* Campbell, 2014** GUA

*Charisius granulatus* Campbell, 2014a: 277.

***Charisius howdenorum* Campbell, 2014** MEX (CI)

*Charisius howdenorum* Campbell, 2014a: 287.

***Charisius mexicanus* Campbell, 1965** MEX (GE ME MI MO OA PU)

*Charisius mexicanus* Campbell, 1965: 49.

***Charisius picturatus* Champion, 1893** MEX (GE ME OA)

*Charisius picturatus* Champion, 1893a: 565.

***Charisius punctatus* Campbell, 2014** GUA

*Charisius punctatus* Campbell, 2014a: 290.

***Charisius salvini* Champion, 1888** GUA SAL HON NIC

*Charisius salvini* Champion, 1888: 423.

***Charisius subalatus* (Champion, 1888)** GUA SAL

*Narses subalatus* Champion, 1888: 424.

***Charisius zunilensis* Champion, 1888** MEX (CI VE) GUA HON

*Charisius zunilensis* Champion, 1888: 422.

*Charisius interstitialis* Champion, 1888: 422. Synonymy: Campbell (2014a: 285).

*Charisius floridanus* Linell, 1899: 184. Synonymy (with *C. interstitialis* Champion): Campbell (1965: 51).

**Genus *Diopoenus* Champion, 1888** [M]

*Diopoenus* Champion, 1888: 445. Type species: *Diopoenus compressicornis* Champion, 1888, monotypy.

***Diopoenus compressicornis* Champion, 1888** MEX (PU)

*Diopoenus compressicornis* Champion, 1888: 445.

**Genus *Hymenorus* Mulsant, 1852** [M]

*Hymenorus* Mulsant, 1852: 68 [as *Hymenophorus*]. Type species: *Hymenorus doublieri* Mulsant, 1852, monotypy. Note. See Bousquet et al. (2015: 133) for precedence of the spelling *Hymenorus* over *Hymenophorus*.

***Hymenorus alienus* Fall, 1931** USA (AZ)

*Hymenorus alienus* Fall, 1931b: 217.

***Hymenorus americanus* Champion, 1888** MEX (CL GE VE) GUA NIC

*Hymenorus americanus* Champion, 1888: 438.

***Hymenorus anguillae* Campbell, 1971** LAN (Anguilla)

*Hymenorus anguillae* Campbell, 1971: 76.

***Hymenorus angustatus* Champion, 1888** MEX (FD) GUA

*Hymenorus angustatus* Champion, 1888: 436.

***Hymenorus antillensis* Campbell, 1971** LAN

*Hymenorus antillensis* Campbell, 1971: 77.

***Hymenorus apacheanus* Casey, 1891** USA (AZ CA)

*Hymenorus apacheanus* Casey, 1891: 99.

***Hymenorus arkansanus* Fall, 1931** USA (FL AR)

*Hymenorus arkansanus* Fall, 1931b: 183.

***Hymenorus atratus* Fall, 1931** USA (AZ)

*Hymenorus atratus* Fall, 1931b: 189.

***Hymenorus badius* Champion, 1888** MEX (VE)

*Hymenorus badius* Champion, 1888: 433.

***Hymenorus bahamensis* Campbell, 1971** BAH CUB

*Hymenorus bahamensis* Campbell, 1971: 88.

***Hymenorus balli* Campbell, 2014** MEX (CI) GUA

*Hymenorus balli* Campbell, 2014b: 299.

***Hymenorus bifurcatus* Campbell, 2014** GUA

*Hymenorus bifurcatus* Campbell, 2014b: 301.

***Hymenorus bitumescens* Fall, 1931** USA (AZ)

*Hymenorus bitumescens* Fall, 1931b: 194.

***Hymenorus brevicornis* Champion, 1888** MEX (FD VE)

*Hymenorus brevicornis* Champion, 1888: 426.

***Hymenorus brevipes* Champion, 1888** MEX (GE)

*Hymenorus brevipes* Champion, 1888: 435.

***Hymenorus brevis* Fall, 1931** USA (AZ)

*Hymenorus brevis* Fall, 1931b: 230.

***Hymenorus caducus* Fall, 1931** USA (AL FL)

*Hymenorus caducus* Fall, 1931b: 213.

***Hymenorus canaliculatus* Champion, 1888** MEX (VE)

*Hymenorus canaliculatus* Champion, 1888: 428.

***Hymenorus capensis* Fall, 1931** MEX (BS)

*Hymenorus capensis* Fall, 1931b: 205.

***Hymenorus castaneus* Champion, 1888** MEX (DU)

*Hymenorus castaneus* Champion, 1888: 434.

***Hymenorus cassus* Fall, 1931** MEX (BC)

*Hymenorus cassus* Fall, 1931b: 197.

***Hymenorus caurinus* Fall, 1931** CAN (BC) USA (OR)

*Hymenorus caurinus* Fall, 1931b: 185.

***Hymenorus chiriquensis* Campbell, 1962** PAN

*Hymenorus chiriquensis* Campbell, 1962: 95.

***Hymenorus colonoides* Champion, 1888** MEX (GU JA PU VE) GUA

*Hymenorus colonoides* Champion, 1888: 435.

***Hymenorus communis* LeConte, 1866** USA (FL GA MD NC NY PA SC WI)

*Hymenorus communis* LeConte, 1866b: 135.

***Hymenorus confertus* LeConte, 1866** MEX (BS)

*Hymenorus confertus* LeConte, 1866b: 136.

***Hymenorus conformis* Fall, 1931** USA (TX)

*Hymenorus conformis* Fall, 1931b: 199.

***Hymenorus conicicollis* Fall, 1931** USA (GA SC)

*Hymenorus conicicollis* Fall, 1931b: 239.

***Hymenorus convexus* Casey, 1891** USA (FL TX) / BAH TUR CUB CAY

*Hymenorus convexus* Casey, 1891: 106.

***Hymenorus corticarioides* Champion, 1888** MEX (CL GE)

*Hymenorus corticarioides* Champion, 1888: 441.

***Hymenorus crinitus* Fall, 1931** USA (AZ)

*Hymenorus crinitus* Fall, 1931b: 244.

***Hymenorus curticollis* Casey, 1891** USA (AR IA IN MS PA)

*Hymenorus curticollis* Casey, 1891: 95.

***Hymenorus cubensis* Campbell, 1971** CUB

*Hymenorus cubensis* Campbell, 1971: 81.

***Hymenorus darlingtoni* Campbell, 1971** CUB

*Hymenorus darlingtoni* Campbell, 1971: 83.

***Hymenorus densus* LeConte, 1866** USA (AL FL GA IN NC SC TX) MEX (VE) / BAH

*Hymenorus densus* LeConte, 1866b: 138.

***Hymenorus deplanatus* Champion, 1888** USA (AZ) MEX (SO)

*Hymenorus deplanatus* Champion, 1888: 440.

*Hymenorus gemellus* Casey, 1891: 121. Synonymy: Fall (1931b: 231).

***Hymenorus depressus* Champion, 1888** MEX (GE)

*Hymenorus depressus* Champion, 1888: 435.

***Hymenorus dichrous* Blatchley, 1919** USA (FL GA NC SC)

*Hymenorus dichrous* Blatchley, 1919: 66.

***Hymenorus difficilis* Casey, 1891** USA (NY)

*Hymenorus difficilis* Casey, 1891: 94.

***Hymenorus digressus* Fall, 1931** USA (AZ)

*Hymenorus digressus* Fall, 1931b: 206.

***Hymenorus discrepans* Casey, 1891** USA (CA)

*Hymenorus discrepans* Casey, 1891: 98.

***Hymenorus discretus* Casey, 1891** CAN (ON QC) USA (FL GA IN MA MD MN MO NC NE NJ NY PA RI SC VA WI)

*Hymenorus discretus* Casey, 1891: 105.

***Hymenorus disparatus* Fall, 1931** USA (AZ CO NM TX)

*Hymenorus disparatus* Fall, 1931b: 215.

***Hymenorus dissensus* Casey, 1891** USA (TX)

*Hymenorus dissensus* Casey, 1891: 109.

***Hymenorus distinctus* Fall, 1931** USA (AL FL GA MS SC)

*Hymenorus distinctus* Fall, 1931b: 179.

***Hymenorus dorsalis* Schwarz, 1878** USA (AL FL GA NC SC)

*Hymenorus dorsalis* Schwarz, 1878: 370.

*Hymenorus sabalensis* Blatchley, 1919: 67. Synonymy: Fall (1931b: 212).

***Hymenorus dubius* Fall, 1931** USA (AL FL GA MS SC)

*Hymenorus dubius* Fall, 1931b: 184.

***Hymenorus durangoensis* Champion, 1888** MEX (DU)

*Hymenorus durangoensis* Champion, 1888: 426.

***Hymenorus emmenastoides* Champion, 1888** MEX (VE) GUA

*Hymenorus emmenastoides* Champion, 1888: 436.

***Hymenorus excavatus* Campbell, 2014** GUA

*Hymenorus excavatus* Campbell, 2014b: 305.

***Hymenorus exiguus* Casey, 1891** USA (AZ CA TX)

*Hymenorus exiguus* Casey, 1891: 100.

***Hymenorus exilis* Fall, 1931** USA (AZ)

*Hymenorus exilis* Fall, 1931b: 233.

***Hymenorus facetus* Fall, 1931** MEX (BS)

*Hymenorus facetus* Fall, 1931b: 234.

***Hymenorus farri* Campbell, 1971** USA (FL) MEX (VE) GUA BEL / BAH TUR CUB CAY JAM PRI LAN

*Hymenorus farri* Campbell, 1971: 84.

***Hymenorus flohri* Champion, 1888** MEX (FD MO)

*Hymenorus flohri* Champion, 1888: 429.

***Hymenorus floridanus* Casey, 1891** USA (FL)

*Hymenorus floridanus* Casey, 1891: 116.

***Hymenorus forreri* Champion, 1888** MEX (DU)

*Hymenorus forreri* Champion, 1888: 431.

***Hymenorus foveiventris* Champion, 1888** GUA

*Hymenorus foveiventris* Champion, 1888: 432.

***Hymenorus fuscipennis* Fall, 1931** USA (FL)

*Hymenorus fuscipennis* Fall, 1931b: 211.

***Hymenorus fusculus* Casey, 1891** USA (CA)

*Hymenorus fusculus* Casey, 1891: 117.

***Hymenorus fusicornis* Casey, 1891** USA (CA)

*Hymenorus fusicornis* Casey, 1891: 112.

***Hymenorus grandicollis* Champion, 1888** USA (AZ) MEX (SO)

*Hymenorus grandicollis* Champion, 1888: 429.

***Hymenorus granulatus* Blatchley, 1912** USA (FL)

*Hymenorus granulatus* Blatchley, 1912: 331.

***Hymenorus guatemalensis* Champion, 1888** GUA

*Hymenorus guatemalensis* Champion, 1888: 439.

***Hymenorus haitellus* Campbell, 1971** HAI

*Hymenorus haitellus* Campbell, 1971: 94.

***Hymenorus haitius* Campbell, 1971** HAI DOM

*Hymenorus haitius* Campbell, 1971: 93.

***Hymenorus helvinus* Casey, 1891** USA (TX)

*Hymenorus helvinus* Casey, 1891: 101.

***Hymenorus heteropygus* Fall, 1931** USA (FL GA MS)

*Hymenorus heteropygus* Fall, 1931b: 241.

***Hymenorus hispaniolensis* Campbell, 1971** HAI DOM

*Hymenorus hispaniolensis* Campbell, 1971: 78.

***Hymenorus hispidulus* Champion, 1888** MEX (VE)

*Hymenorus hispidulus* Champion, 1888: 431.

***Hymenorus horrescens* Fall, 1931** USA (NM TX)

*Hymenorus horrescens* Fall, 1931b: 235.

***Hymenorus humeralis* LeConte, 1866** USA (AL FL KY MD OH PA SC TN)

*Hymenorus humeralis* LeConte, 1866b: 135.

***Hymenorus idoneus* Fall, 1931** USA (AZ)

*Hymenorus idoneus* Fall, 1931b: 218.

***Hymenorus igualensis* Champion, 1888** MEX (GE JA)

*Hymenorus igualensis* Champion, 1888: 434.

***Hymenorus illusus* Fall, 1931** USA (AL FL GA MD SC)

*Hymenorus illusus* Fall, 1931b: 192.

***Hymenorus inaequalis* Casey, 1891** USA (AZ)

*Hymenorus inaequalis* Casey, 1891: 114.

***Hymenorus incertus* Fall, 1931** USA (AZ)

*Hymenorus incertus* Fall, 1931b: 220.

***Hymenorus indutus* Casey, 1891** USA (AZ NM TX)

*Hymenorus indutus* Casey, 1891: 119.

***Hymenorus infuscatus* Casey, 1891** USA (CA)

*Hymenorus infuscatus* Casey, 1891: 90.

***Hymenorus inopiatus* Fall, 1931** USA (FL GA MD SC)

*Hymenorus inopiatus* Fall, 1931b: 242.

***Hymenorus inquilinus* Casey, 1891** USA (CA)

*Hymenorus inquilinus* Casey, 1891: 112.

***Hymenorus insularis* Campbell, 1971** BAH

*Hymenorus insularis* Campbell, 1971: 91.

***Hymenorus intermedius* Casey, 1891** USA (AZ TX)

*Hymenorus intermedius* Casey, 1891: 102.

***Hymenorus inutilis* Fall, 1931** USA (AZ NM NV)

*Hymenorus inutilis* Fall, 1931b: 208.

***Hymenorus irritus* Fall, 1931** USA (AZ CA)

*Hymenorus irritus* Fall, 1931b: 199.

***Hymenorus jacobinus* Fall, 1931** USA (CA)

*Hymenorus jacobinus* Fall, 1931b: 206.

***Hymenorus jamaicensis* Campbell, 1971** CAY JAM

*Hymenorus jamaicensis* Campbell, 1971: 79.

***Hymenorus laticollis* Champion, 1888** MEX (FD GE JA)

*Hymenorus laticollis* Champion, 1888: 429.

***Hymenorus longicollis* Champion, 1888** MEX (VE)

*Hymenorus longicollis* Champion, 1888: 434.

***Hymenorus macilentus* Fall, 1931** USA (NM)

*Hymenorus macilentus* Fall, 1931b: 188.

***Hymenorus maritimus* Champion, 1888** GUA

*Hymenorus maritimus* Champion, 1888: 437.

***Hymenorus melsheimeri* Casey, 1891** USA (MI NY SC)

*Hymenorus melsheimeri* Casey, 1891: 92.

***Hymenorus milleporus* Fall, 1931** USA (AZ)

*Hymenorus milleporus* Fall, 1931b: 236.

***Hymenorus minutus* Campbell, 1971** BAH

*Hymenorus minutus* Campbell, 1971: 98.

***Hymenorus molestus* Fall, 1931** CAN (NB NS ON PE QC) USA (IN LA PA WI)

*Hymenorus molestus* Fall, 1931b: 182.

***Hymenorus montivagus* Fall, 1931** USA (CA)

*Hymenorus montivagus* Fall, 1931b: 207.

***Hymenorus nevadensis* Fall, 1931** USA (NV)

*Hymenorus nevadensis* Fall, 1931b: 236.

***Hymenorus niger* (Melsheimer, 1846)** CAN (MB NB NS ON PE QC) USA (FL GA IN MA MD MI MN MS NC NY PA SC TX WI)

*Mycetocharus niger* Melsheimer, 1846: 59.

***Hymenorus nitidipennis* Casey, 1891** USA (AZ)

*Hymenorus nitidipennis* Casey, 1891: 113.

***Hymenorus obesus* Casey, 1891** CAN (MB NB NS ON QC) USA (AL FL GA IN LA MA MD MI MO NC NJ NY PA SC TX VA WI)

*Hymenorus obesus* Casey, 1891: 93.

***Hymenorus oblivius* Fall, 1931** USA (TX)

*Hymenorus oblivius* Fall, 1931b: 216.

***Hymenorus obscurus* (Say, 1826)** USA (FL GA IN MA MD NJ NY PA SC TX VA WI)

*Cistela obscura* Say, 1826: 242.

***Hymenorus occidentalis* Champion, 1888** USA (TX) MEX (GU VE)

*Hymenorus occidentalis* Champion, 1888: 425.

***Hymenorus oculatus* Champion, 1888** MEX (VE) GUA

*Hymenorus oculatus* Champion, 1888: 427.

***Hymenorus pallidus* Champion, 1888** MEX (DU GE)

*Hymenorus pallidus* Champion, 1888: 439.

***Hymenorus panamensis* Campbell, 1962** PAN

*Hymenorus panamensis* Campbell, 1962: 93.

***Hymenorus papagonis* Fall, 1931** USA (AZ)

*Hymenorus papagonis* Fall, 1931b: 201.

***Hymenorus parvicollis* Champion, 1888** MEX (DU)

*Hymenorus parvicollis* Champion, 1888: 440.

***Hymenorus parvus* Fall, 1931** USA (CA) MEX (BS)

*Hymenorus parvus* Fall, 1931b: 203.

***Hymenorus perforatus* Casey, 1891** USA (GA IA IN MD NC PA SC)

*Hymenorus perforatus* Casey, 1891: 95.

***Hymenorus picipennis* Casey, 1891** CAN (NS ON QC) USA (AL MD MI NH NY PA SC WI)

*Hymenorus picipennis* Casey, 1891: 90.

***Hymenorus pilosus* (Melsheimer, 1846)** CAN (NS ON QC) USA (AL AR FL GA IA IN KS LA MA MD MI MS NC NJ NY OH PA SC SD VA WI)

*Allecula pilosa* Melsheimer, 1846: 58.

***Hymenorus pini* Champion, 1888** GUA

*Hymenorus pini* Champion, 1888: 428.

***Hymenorus planulus* Horn, 1894** MEX (BS)

*Hymenorus planulus* Horn, 1894b: 434.

***Hymenorus porosicornis* Casey, 1891** USA (NM TX)

*Hymenorus porosicornis* Casey, 1891: 101.

***Hymenorus prolixus* Casey, 1891** USA (AZ NM NV TX UT)

*Hymenorus prolixus* Casey, 1891: 103.

***Hymenorus protibialis* Fall, 1931** USA (AZ CA)

*Hymenorus protibialis* Fall, 1931b: 196.

***Hymenorus punctatissimus* LeConte, 1866** USA (AZ CA NV TX UT) MEX (SO)

*Hymenorus punctatissimus* LeConte, 1866b: 138.

*Hymenorus macer* Casey, 1891: 118. Synonymy: Fall (1931b: 221).

***Hymenorus punctulatus* (LeConte, 1859)** USA (CA)

*Allecula punctulata* LeConte, 1859b: 78.

***Hymenorus pygmaeus* Campbell, 1971** BAH

*Hymenorus pygmaeus* Campbell, 1971: 99.

***Hymenorus quietus* Fall, 1931** USA (FL MO)

*Hymenorus quietus* Fall, 1931b: 239.

***Hymenorus rotundicollis* Casey, 1891** USA (AZ)

*Hymenorus rotundicollis* Casey, 1891: 111.

***Hymenorus rufescens* Champion, 1888** MEX (VE YU)

*Hymenorus rufescens* Champion, 1888: 433.

***Hymenorus ruficollis* Champion, 1888** USA (AZ) MEX (BC BS SO)

*Hymenorus ruficollis* Champion, 1888: 438.

***Hymenorus rufohumeralis* Campbell, 1982** USA (CA)

*Hymenorus rufohumeralis* Campbell, 1982: 131.

***Hymenorus rufovalis* Fall, 1931** USA (AZ)

*Hymenorus rufovalis* Fall, 1931b: 230.

***Hymenorus segnis* Champion, 1888** MEX (GE)

*Hymenorus segnis* Champion, 1888: 430.

***Hymenorus semirufus* Fall, 1931** USA (FL)

*Hymenorus semirufus* Fall, 1931b: 203.

***Hymenorus seriatus* Casey, 1891** USA (AZ)

*Hymenorus seriatus* Casey, 1891: 109.

***Hymenorus setosus* Hatch, 1965** USA (OR)

*Hymenophorus setosus* Hatch, 1965: 185.

***Hymenorus significans* Fall, 1931** USA (TX)

*Hymenorus significans* Fall, 1931b: 237.

***Hymenorus similis* Champion, 1888** MEX (DU MO)

*Hymenorus similis* Champion, 1888: 432.

***Hymenorus simiolus* Fall, 1931** USA (TX)

*Hymenorus simiolus* Fall, 1931b: 232.

***Hymenorus sinuatus ebeninus* Fall, 1931** USA (CA)

Hymenorus sinuatus var. ebeninus Fall, 1931b: 188.

***Hymenorus sinuatus sinuatus* Fall, 1931** CAN (BC) USA (CA ID OR WA)

*Hymenorus sinuatus* Fall, 1931b: 187.

*Hymenophorus megops* Hatch, 1965: 185. **New synonymy** [YB].

*Telesicles magnus* Hatch, 1965: 185. **New synonymy** [YB].

***Hymenorus sobrinus* Casey, 1891** USA (FL MD NJ SC WI)

*Hymenorus sobrinus* Casey, 1891: 115.

***Hymenorus sordidus* Champion, 1888** MEX (VE) GUA

*Hymenorus sordidus* Champion, 1888: 427.

***Hymenorus sparsepunctatus* Campbell, 1971** CUB

*Hymenorus sparsepunctatus* Campbell, 1971: 97.

***Hymenorus spinifer* Horn, 1894** USA (AZ)

*Hymenorus spinifer* Horn, 1894b: 434.

***Hymenorus striatus* (Pic, 1930)** HAI DOM

*Cistelopsis striata* Pic, 1930: 26.

***Hymenorus tarsalis* Champion, 1888** GUA

*Hymenorus tarsalis* Champion, 1888: 426.

***Hymenorus tenellus* Casey, 1891** USA (FL GA MD NJ SC)

*Hymenorus tenellus* Casey, 1891: 115.

*Hymenorus elbertae* Blatchley, 1918: 57. Synonymy: Fall (1931b: 227).

***Hymenorus tenuistriatus* Fall, 1931** USA (AL FL NC SC)

*Hymenorus tenuistriatus* Fall, 1931b: 226.

***Hymenorus testaceus* Casey, 1891** USA (AZ)

*Hymenorus testaceus* Casey, 1891: 110.

***Hymenorus texensis* Fall, 1931** USA (TX)

*Hymenorus texensis* Fall, 1931b: 241.

***Hymenorus thoracicus* Fall, 1931** USA (CA)

*Hymenorus thoracicus* Fall, 1931b: 214.

***Hymenorus tibialis* Champion, 1888** GUA

*Hymenorus tibialis* Champion, 1888: 430.

***Hymenorus torridus* Champion, 1888** MEX (GE)

*Hymenorus torridus* Champion, 1888: 436.

***Hymenorus transversus* Campbell, 1971** BAH

*Hymenorus transversus* Campbell, 1971: 92.

***Hymenorus tritus* Fall, 1931** USA (AZ)

*Hymenorus tritus* Fall, 1931b: 219.

***Hymenorus trivialis* Fall, 1931** MEX (BC)

*Hymenorus trivialis* Fall, 1931b: 210.

***Hymenorus ulomoides* Fall, 1931** USA (CA)

*Hymenorus ulomoides* Fall, 1931b: 187.

***Hymenorus uniseriatus* Casey, 1891** USA (CA)

*Hymenorus uniseriatus* Casey, 1891: 115.

***Hymenorus vigilax* Fall, 1931** USA (AZ)

*Hymenorus vigilax* Fall, 1931b: 200.

***Hymenorus villosus* Champion, 1888** MEX (JA MO)

*Hymenorus villosus* Champion, 1888: 440.

***Hymenorus wolcotti* Campbell, 1971** PRI VIS

*Hymenorus wolcotti* Campbell, 1971: 74.

**Genus *Knausia* Fall, 1931** [F]

*Knausia* Fall, 1931a: 15. Type species: *Knausia crassicornis* Fall, 1931, monotypy.

***Knausia crassicornis* Fall, 1931** USA (NM TX)

*Knausia crassicornis* Fall, 1931a: 16.

**Genus *Latacula* Campbell, 1971** [F]

*Latacula* Campbell, 1971: 103. Type species: *Latacula beckeri* Campbell, 1971, original designation.

***Latacula beckeri* Campbell, 1971** JAM

*Latacula beckeri* Campbell, 1971: 105.

***Latacula insularis* Campbell, 1971** JAM

*Latacula insularis* Campbell, 1971: 106.

**Genus *Lobopoda* Solier, 1835** [F]

*Lobopoda* Solier, 1835a: 233. Type species: *Lobopoda striata* Solier, 1835, subsequent designation (Bousquet et al. 2015: 134).

**Subgenus Flavipoda Campbell, 1966**

*Flavipoda* Campbell, 1966: 21. Type species: *Helops flavipes* Fabricius, 1792 [as *Allecula flavipes* Jacquelin duVal, 1857], original designation.

***Lobopoda androsi* Campbell, 1971** BAH

*Lobopoda androsi* Campbell, 1971: 33.

***Lobopoda badia* Campbell, 1971** CUB

*Lobopoda badius* Campbell, 1971: 31.

***Lobopoda bahamensis* Campbell, 1966** BAH CUB

*Lobopoda bahamensis* Campbell, 1966: 27.

***Lobopoda bicolor* Campbell, 1966** CUB

*Lobopoda bicolor* Campbell, 1966: 28.

***Lobopoda cayamasensis* Campbell, 1966** CUB

*Lobopoda cayamasensis* Campbell, 1966: 33.

***Lobopoda deyrupi* Steiner, 2006** BAH

*Lobopoda deyrupi* Steiner, 2006: 32.

***Lobopoda emarginata* Campbell, 1966** CUB

*Lobopoda emarginata* Campbell, 1966: 32.

***Lobopoda flavifemoralis* Campbell, 1966** CUB

*Lobopoda flavifemoralis* Campbell, 1966: 29.

***Lobopoda flavipes* (Fabricius, 1792)** ^64^ CUB

*Helops flavipes* Fabricius, 1792a: 122 [secondary homonym of *Cistela flavipes* Fabricius, 1792b: 45].

*Cistela fuscula* Schönherr, 1808: 336. Replacement name for *Cistela flavipes* (Fabricius, 1792a).

***Lobopoda nesiotica* Campbell, 1971** BAH

*Lobopoda nesiotica* Campbell, 1971: 36.

***Lobopoda quadratinota* Campbell, 1971** CUB

*Lobopoda quadratinota* Campbell, 1971: 28.

***Lobopoda schwarzi* Campbell, 1971** CUB

*Lobopoda schwarzi* Campbell, 1971: 29.

***Lobopoda tibiodentata* Campbell, 1966** CUB

*Lobopoda tibiodentata* Campbell, 1966: 30.

***Lobopoda villasensis* Campbell, 1971** CUB

*Lobopoda villasensis* Campbell, 1971: 35.

**Subgenus Glabrilobopoda Campbell, 1966**

*Glabrilobopoda* Campbell, 1966: 46. Type species: *Lobopoda glabrata* Champion, 1888, original designation.

***Lobopoda aeneipennis* Champion, 1888** PAN

*Lobopoda aeneipennis* Champion, 1888: 408.

***Lobopoda cariniventris* Champion, 1888** PAN

*Lobopoda cariniventris* Champion, 1888: 408.

***Lobopoda coronadensis* Campbell, 1966** CRI

*Lobopoda coronadensis* Campbell, 1966: 54.

***Lobopoda darlingtoni* Campbell, 1971** DOM

*Lobopoda darlingtoni* Campbell, 1971: 40.

***Lobopoda glabrata* Champion, 1888** PAN

*Lobopoda glabrata* Champion, 1888: 409.

***Lobopoda impunctata* Campbell, 1966** CRI

*Lobopoda impunctata* Campbell, 1966: 52.

***Lobopoda irazuensis* Champion, 1888** CRI

*Lobopoda irazuensis* Champion, 1888: 406.

***Lobopoda nitens* Champion, 1888** CRI

*Lobopoda nitens* Champion, 1888: 406.

***Lobopoda nitida* Champion, 1888** PAN

*Lobopoda nitida* Champion, 1888: 407.

***Lobopoda obsoleta* Champion, 1888** MEX (VE) GUA

*Lobopoda obsoleta* Champion, 1888: 409.

***Lobopoda portobellensis* Campbell, 1966** PAN

*Lobopoda portobellensis* Campbell, 1966: 58.

***Lobopoda tilaranensis* Campbell, 1966** CRI

*Lobopoda tilaranensis* Campbell, 1966: 50.

***Lobopoda viridipennis* Champion, 1888** PAN

*Lobopoda viridipennis* Champion, 1888: 407.

**Subgenus Lobopoda Solier, 1835**

*Lobopoda* Solier, 1835a: 233. Type species: *Lobopoda striata* Solier, 1835, subsequent designation (Bousquet et al. 2015: 134).

***Lobopoda acuticauda* Campbell, 1966** NIC CRI PAN

*Lobopoda acuticauda* Campbell, 1966: 77.

***Lobopoda aeneotincta* Champion, 1888** CRI PAN

*Lobopoda aeneotincta* Champion, 1888: 405.

***Lobopoda alutacea* Campbell, 1971** CUB

*Lobopoda alutacea* Campbell, 1971: 58.

***Lobopoda apicalis* Champion, 1888** GUA

*Lobopoda apicalis* Champion, 1888: 393.

***Lobopoda atrata* Champion, 1888** NIC PAN

*Lobopoda atrata* Champion, 1888: 394.

***Lobopoda attenuata* Champion, 1888** GUA NIC CRI

*Lobopoda attenuata* Champion, 1888: 397.

***Lobopoda calcarata* Champion, 1893** MEX (OA)

*Lobopoda calcarata* Champion, 1893a: 563.

***Lobopoda championi* Campbell, 1966** CRI PAN

*Lobopoda championi* Campbell, 1966: 105.

***Lobopoda chontalensis* Champion, 1888** NIC CRI

*Lobopoda chontalensis* Champion, 1888: 399.

***Lobopoda colona* Campbell, 1971** HAI

*Lobopoda colona* Campbell, 1971: 56.

***Lobopoda convexicollis* Champion, 1888** MEX (VE YU) GUA

*Lobopoda convexicollis* Champion, 1888: 395.

***Lobopoda cordata* Campbell, 1971** BAH

*Lobopoda cordata* Campbell, 1971: 47.

***Lobopoda costaricensis* Campbell, 1966** CRI

*Lobopoda costaricensis* Campbell, 1966: 103.

***Lobopoda cubensis* Campbell, 1966** CUB

*Lobopoda cubensis* Campbell, 1966: 157.

***Lobopoda distans* Campbell, 1971** CUB

*Lobopoda distans* Campbell, 1971: 48.

***Lobopoda diversicauda* Campbell, 1966** CRI

*Lobopoda diversicauda* Campbell, 1966: 106.

***Lobopoda erythrocnemis* (Germar, 1823)** USA (AL AR FL GA KY LA MD MS NC SC TN TX)

*Allecula erythrocnemis* Germar, 1823: 164.

***Lobopoda fallaciosa* Campbell, 1971** CUB

*Lobopoda fallaciosa* Campbell, 1971: 49.

***Lobopoda femoralis* Champion, 1888** MEX (CI SL TB VE) GUA CRI PAN

*Lobopoda femoralis* Champion, 1888: 398.

***Lobopoda foveata* Champion, 1888** CRI PAN

*Lobopoda foveata* Champion, 1888: 405.

***Lobopoda galapagoensis* Linell, 1898** SAL NIC PAN / SA

*Lobopoda galapagoensis* Linell, 1898: 266.

*Lobopoda brunneipennis* Campbell, 1966: 98. Synonymy: Peck and Kukalová-Peck (1990: 1637).

***Lobopoda granulata* Campbell, 1966** CRI PAN LAN (Barbados) / SA

*Lobopoda granulata* Campbell, 1966: 85.

***Lobopoda guatemalensis* Campbell, 1966** GUA

*Lobopoda guatemalensis* Campbell, 1966: 150.

***Lobopoda guerrerensis* Campbell, 1966** MEX (GE)

*Lobopoda guerrerensis* Campbell, 1966: 152.

***Lobopoda haitensis* Campbell, 1966** HAI DOM

*Lobopoda haitensis* Campbell, 1966: 158.

***Lobopoda hirta* Champion, 1888** NIC

*Lobopoda hirta* Champion, 1888: 400.

***Lobopoda hispaniolensis* Campbell, 1971** DOM

*Lobopoda hispaniolensis* Campbell, 1971: 46.

***Lobopoda insularis* Champion, 1896** LAN

*Lobopoda insularis* Champion, 1896: 33.

***Lobopoda jamaicensis* Campbell, 1966** JAM

*Lobopoda jamaicensis* Campbell, 1966: 161.

***Lobopoda laevicollis* Champion, 1888** MEX (CI VE YU)

*Lobopoda laevicollis* Champion, 1888: 401.

***Lobopoda meridensis* Campbell, 1966** MEX (YU)

*Lobopoda meridensis* Campbell, 1966: 147.

***Lobopoda micans* Campbell, 1971** DOM

*Lobopoda micans* Campbell, 1971: 50.

***Lobopoda minuta* Champion, 1888** PAN

*Lobopoda minuta* Champion, 1888: 403.

***Lobopoda monticola* Campbell, 1966** USA (TX)

*Lobopoda monticola* Campbell, 1966: 124.

***Lobopoda mucronata* Champion, 1888** PAN

*Lobopoda mucronata* Champion, 1888: 393.

***Lobopoda nigrans* (Melsheimer, 1846)** USA (AL CT DC FL GA IL IN KS LA MA MD MI MS NC NJ NY OH PA RI SC TX VA)

*Cistela atra* Say, 1826: 242 [junior primary homonym of *Cistela ater* Fabricius, 1775 and *Cistela atra* Olivier, 1795].

*Cistela nigrans* Melsheimer, 1846: 60. Replacement name for *Cistela atra* Say, 1826.

***Lobopoda nigrissima* Campbell, 1966** MEX (TA)

*Lobopoda nigrissima* Campbell, 1966: 133.

***Lobopoda notapuncta* Campbell, 1971** HAI DOM

*Lobopoda notapuncta* Campbell, 1971: 54.

***Lobopoda oblonga* Champion, 1888** MEX (YU)

*Lobopoda oblonga* Champion, 1888: 396.

***Lobopoda opaca* Champion, 1888** MEX (CI) GUA CRI PAN

*Lobopoda opaca* Champion, 1888: 400.

*Lobopoda biolleyi* Pic, 1927: 22. Synonymy: Campbell (1966: 87).

***Lobopoda opacicollis* Champion, 1888** USA (FL LA TX) MEX (GU NL SI TA VE) GUA BEL HON NIC

*Lobopoda opacicollis* Champion, 1888: 400.

*Lobopoda subcuneata* Casey, 1891: 79. Synonymy: Campbell (1966: 83).

***Lobopoda panamensis* Champion, 1888** PAN / SA

*Lobopoda panamensis* Champion, 1888: 392.

***Lobopoda paracollis* Campbell, 1971** CUB

*Lobopoda paracollis* Campbell, 1971: 55.

***Lobopoda paracornis* Campbell, 1971** CUB

*Lobopoda paracornis* Campbell, 1971: 53.

***Lobopoda parvula* Champion, 1888** MEX (JA VE)

*Lobopoda parvula* Champion, 1888: 403.

***Lobopoda picipennis* Campbell, 1971** HAI DOM

*Lobopoda picipennis* Campbell, 1971: 61.

***Lobopoda pilosa* Champion, 1888** MEX (CI) GUA

*Lobopoda pilosa* Champion, 1888: 405.

***Lobopoda polita* Campbell, 1971** CUB

*Lobopoda polita* Campbell, 1971: 52.

***Lobopoda proxima* Champion, 1888** MEX (CI VE YU) GUA

*Lobopoda proxima* Champion, 1888: 402.

***Lobopoda puncticollis* Champion, 1888** GUA

*Lobopoda puncticollis* Champion, 1888: 396.

***Lobopoda punctulata* (Melsheimer, 1846)** USA (AL AR FL GA IA IL IN KS KY LA MD MO MS NC NJ NY OH OK PA SC TN TX VA WI) MEX (NL TA VE)

*Cistela punctulata* Melsheimer, 1846: 59.

*Lobopoda jalapensis* Champion, 1888: 402. Synonymy: Campbell (1966: 119).

*Lobopoda oculatifrons* Casey, 1891: 81. Synonymy: Campbell (1966: 119).

***Lobopoda remoinsularis* Campbell, 1966** CRI PAN

*Lobopoda remoinsularis* Campbell, 1966: 99.

***Lobopoda sandersoni* Campbell, 1971** DOM

*Lobopoda sandersoni* Campbell, 1971: 60.

***Lobopoda sculpturata* Champion, 1888** PAN

*Lobopoda sculpturata* Champion, 1888: 401.

***Lobopoda seriata* Champion, 1888** MEX (YU)

*Lobopoda seriata* Champion, 1888: 395.

***Lobopoda simplex* Champion, 1888** BEL

*Lobopoda simplex* Champion, 1888: 399.

***Lobopoda subparallela* Champion, 1888** MEX (CI GE MO OA VE)

*Lobopoda subparallela* Champion, 1888: 394.

***Lobopoda substriatus* Campbell, 1966** DOM

*Lobopoda substriatus* Campbell, 1966: 159.

***Lobopoda sulcaticollis* Pic, 1933** CUB

*Lobopoda sulcaticollis* Pic, 1933: 1.

***Lobopoda tabogensis* Campbell, 1966** PAN

*Lobopoda tabogensis* Campbell, 1966: 78.

***Lobopoda teapensis* Champion, 1893** MEX (TB)

*Lobopoda teapensis* Champion, 1893a: 564.

***Lobopoda tenuicornis* Champion, 1888** MEX (CI) PAN

*Lobopoda tenuicornis* Champion, 1888: 403.

***Lobopoda terminalis* Campbell, 1966** GUA

*Lobopoda terminalis* Campbell, 1966: 145.

***Lobopoda thomasensis* Campbell, 1971** VIS (St. Thomas)

*Lobopoda thomasensis* Campbell, 1971: 50.

***Lobopoda tropicalis* Champion, 1888** PAN

*Lobopoda tropicalis* Champion, 1888: 398.

***Lobopoda veracruzensis* Campbell, 1966** MEX (TA VE)

*Lobopoda veracruzensis* Campbell, 1966: 150.

***Lobopoda viridis* Champion, 1888** MEX (CI VE) NIC

*Lobopoda viridis* Champion, 1888: 404.

*Lobopoda longipes* Borchmann, 1937: 218. Synonymy: Campbell (1966: 135).

***Lobopoda yucatanica* Champion, 1888** MEX (YU)

*Lobopoda yucatanica* Champion, 1888: 397.

***Lobopoda wittmeri* Campbell, 1978** DOM

*Lobopoda wittmeri* Campbell, 1978c: 204.

**Subgenus Mesolobopoda Campbell, 1966**

*Mesolobopoda* Campbell, 1966: 34. Type species: *Allecula socia* LeConte, 1854, original designation.

***Lobopoda acutangula* Champion, 1888** MEX (CI HI MI VE) GUA BEL NIC CRI PAN

*Lobopoda acutangula* Champion, 1888: 390.

***Lobopoda antiguaensis* Campbell, 1971** LAN (Antigua)

*Lobopoda antiguaensis* Campbell, 1971: 39.

***Lobopoda ebenina* Champion, 1896** LAN

*Lobopoda ebenina* Champion, 1896: 34.

***Lobopoda socia* (LeConte, 1854)** USA (FL LA TX) MEX (JA NL SI SL TA TB VE YU) GUA BEL HON NIC

*Allecula socia* LeConte, 1854a: 84.

*Lobopoda mexicana* Champion, 1888: 392. Synonymy: Campbell (1966: 42).

***Lobopoda trinidadensis* Campbell, 1966** MEX (YU) / SA

*Lobopoda trinidadensis* Campbell, 1966: 39.

***Lobopoda tristis* Champion, 1888** CRI PAN

*Lobopoda tristis* Champion, 1888: 391.

**Subgenus Monoloba Solier, 1835**

*Monoloba* Solier, 1835a: 235. Type species: *Lobopoda dircaeoides* Solier, 1835, monotypy.

***Lobopoda asperula* Champion, 1888** MEX (YU)

*Lobopoda asperula* Champion, 1888: 390.

***Lobopoda gigantea* Champion, 1888** MEX (VE)

*Lobopoda gigantea* Champion, 1888: 388.

***Lobopoda grandis* Champion, 1888** NIC PAN / SA

*Lobopoda grandis* Champion, 1888: 389.

***Lobopoda tarsalis* Fleutiaux and Sallé, 1890** LAN (Guadeloupe)

*Lobopoda tarsalis* Fleutiaux and Sallé, 1890: 431.

[incertae sedis]

***Lobopoda sordida* (Horn, 1894)** MEX (BC)

*Allecula sordida* Horn, 1894b: 432.

**Genus *Madreallecula* Kanda, 2013** [F]

*Madreallecula* Kanda, 2013: 587. Type species: *Madreallecula mcclevei* Kanda, 2013, original designation.

***Madreallecula mcclevei* Kanda, 2013** USA (AZ)

*Madreallecula mcclevei* Kanda, 2013: 588.

**Genus *Menes* Champion, 1888** [M]

*Menes* Champion, 1888: 442. Type species: *Menes meridanus* Champion, 1888, subsequent designation (Bousquet et al. 2015: 134).

***Menes meridanus* Champion, 1888** MEX (YU)

*Menes meridanus* Champion, 1888: 442.

***Menes rotundatus* Champion, 1888** MEX (VE)

*Menes rotundatus* Champion, 1888: 443.

**Genus *Menoeceus* Champion, 1888** [M]

*Menoeceus* Champion, 1888: 443. Type species: *Menoeceus crassicornis* Champion, 1888, subsequent designation (Casey 1891: 122).

***Menoeceus aequalis* Champion, 1888** MEX (VE)

*Menoeceus aequalis* Champion, 1888: 444.

***Menoeceus crassicornis* Champion, 1888** MEX (GE JA MO PU VE) GUA

*Menoeceus crassicornis* Champion, 1888: 444.

***Menoeceus texanus* Champion, 1888** USA (TX)

*Menoeceus texanus* Champion, 1888: 444.

**Genus *Notacula* Campbell, 1971** [F]

*Notacula* Campbell, 1971: 107. Type species: *Notacula howdenae* Campbell, 1971, original designation.

***Notacula howdenae* Campbell, 1971** JAM

*Notacula howdenae* Campbell, 1971: 107.

**Genus *Obesacula* Campbell, 1971** [F]

*Obesacula* Campbell, 1971: 109. Type species: *Obesacula aptera* Campbell, 1971, original designation.

***Obesacula aptera* Campbell, 1971** JAM

*Obesacula aptera* Campbell, 1971: 111.

*Cyrtosoma jamaicensis* Marcuzzi, 1977: 42. Synonymy: Ivie (2005: 70).

**Genus *Parahymenorus* Campbell, 1971** [M]

*Parahymenorus* Campbell, 1971: 100. Type species: *Parahymenorus metallicus* Campbell, 1971, original designation.

***Parahymenorus metallicus caymanensis* Campbell, 1971** CAY

*Parahymenorus metallicus caymanensis* Campbell, 1971: 103.

***Parahymenorus metallicus metallicus* Campbell, 1971** JAM

*Parahymenorus metallicus metallicus* Campbell, 1971: 102.

**Genus *Phedius* Champion, 1888** [M]

*Phedius* Champion, 1888: 447. Type species: *Phedius chevrolati* Champion, 1888, subsequent designation (Lucas 1920: 500).

***Phedius carbonarius* Champion, 1888** MEX (HI)

*Phedius carbonarius* Champion, 1888: 448.

***Phedius chevrolati* Champion, 1888** MEX (VE)

*Phedius chevrolati* Champion, 1888: 447.

***Phedius cylindricollis* Champion, 1888** MEX (JA)

*Phedius cylindricollis* Champion, 1888: 449.

***Phedius funereus* Schaeffer, 1905** USA (AZ) MEX (SO)

*Phedius funereus* Schaeffer, 1905b: 176.

***Phedius funestus* Champion, 1888** MEX (OA PU TA)

*Phedius funestus* Champion, 1888: 450.

***Phedius hidalgoensis* Champion, 1888** MEX (HI)

*Phedius hidalgoensis* Champion, 1888: 448.

***Phedius hirtus* Champion, 1893** MEX (GE)

*Phedius hirtus* Champion, 1893a: 568.

***Phedius lapidicola* Champion, 1893** MEX (MO)

*Phedius lapidicola* Champion, 1893a: 568.

***Phedius mexicanus* Champion, 1888** MEX (GU)

*Phedius mexicanus* Champion, 1888: 450.

***Phedius obovatus* Champion, 1888** MEX (AG GU)

*Phedius obovatus* Champion, 1888: 449.

***Phedius opaculus* Horn, 1894** MEX (BS)

*Phedius opaculus* Horn, 1894b: 431.

**Genus *Pitholaus* Champion, 1888** [M]

*Pitholaus* Champion, 1888: 446. Type species: *Pitholaus helopioides* Champion, 1888, monotypy.

***Pitholaus helopioides* Champion, 1888** GUA

*Pitholaus helopioides* Champion, 1888: 446.

**Genus *Polyidus* Champion, 1888** [M]

*Polyidus* Champion, 1888: 441. Type species: *Polydius meridionalis* Champion, 1888, monotypy.

***Polyidus meridionalis* Champion, 1888** MEX (CI) GUA CRI

*Polyidus meridionalis* Champion, 1888: 442.

**Genus *Punctacula* Campbell, 1971** [F]

*Punctacula* Campbell, 1971: 112. Type species: *Punctacula howdeni* Campbell, 1971, original designation.

***Punctacula howdeni* Campbell, 1971** JAM

*Punctacula howdeni* Campbell, 1971: 114.

**Genus *Stenochidus* LeConte, 1862** [M]

*Stenochidus* LeConte, 1862a: 244. Type species: *Stenochia gracilis* LeConte, 1851, subsequent designation (Lucas 1920: 608).

***Stenochidus cyanescens* (LeConte, 1859)** USA (CA NV OR)

*Prionychus cyanescens* LeConte, 1859b: 78.

Stenochidus cyanescens var. carbonarius Schaeffer, 1911: 126. Synonymy: Marshall (1967a: 2).

***Stenochidus gracilis* (LeConte, 1851)** USA (CA)

*Stenochia gracilis* LeConte, 1851: 150.

***Stenochidus robustus* Schaeffer, 1911** USA (CA)

*Stenochidus robustus* Schaeffer, 1911: 125.

**Genus *Telesicles* Champion, 1888** [M]

*Telesicles* Champion, 1888: 450. Type species: *Telesicles cordatus* Champion, 1888, monotypy.

***Telesicles cordatus* Champion, 1888** USA (AZ CA CO NM TX UT) MEX (DU)

*Telesicles cordatus* Champion, 1888: 451.

**Genus *Temnes* Champion, 1888** [M]

*Temnes* Champion, 1888: 410. Type species: *Temnes caeruleus* Champion, 1888, monotypy.

***Temnes caeruleus* Champion, 1888** PAN

*Temnes caeruleus* Champion, 1888: 410.

**Genus *Theatetes* Champion, 1888** [M]

*Theatetes* Champion, 1888: 420. Type species: *Theatetes basicornis* Champion, 1888, monotypy.

***Theatetes basicornis* Champion, 1888** MEX (VE)

*Theatetes basicornis* Champion, 1888: 420.

**Subtribe Gonoderina Seidlitz, 1896**

Gonoderina Seidlitz, 1896: 83. Type genus: *Gonodera* Mulsant, 1856.

Pseudocistelini Portevin, 1934: 39. Type genus: *Pseudocistela* Crotch, 1874.

**Genus *Andrimus* Casey, 1891** [M]

*Andrimus* Casey, 1891: 155. Type species: *Cteniopus murrayi* LeConte, 1866, subsequent designation (Lucas 1920: 96).

***Andrimus murrayi* (LeConte, 1866)** USA (AL FL GA NC SC VA)

*Cteniopus murrayi* LeConte, 1866b: 141.

*Andrimus brunneus* Casey, 1891: 157. Synonymy: Peck and Thomas (1998: 109).

*Andrimus concolor* Casey, 1891: 158. **New synonymy** [based on Marshall (1964: 156) unpublished thesis].

*Andrimus nigrescens* Casey, 1891: 159. Synonymy: Peck and Thomas (1998: 109).

*Andrimus convergens* Casey, 1891: 159. **New synonymy** [based on Marshall (1964: 156) unpublished thesis].

*Andrimus confusus* Blatchley, 1912: 331. Synonymy: Peck and Thomas (1998: 109).

*Andrimus parvulus* Blatchley, 1919: 67. Synonymy: Peck and Thomas (1998: 109).

**Genus *Androchirus* LeConte, 1862** [M]

*Androchirus* LeConte, 1862a: 244. Type species: *Cistela fuscipes* Melsheimer, 1846 (= *Cistela erythropa* Kirby, 1837), original designation.

**Figure 32.**
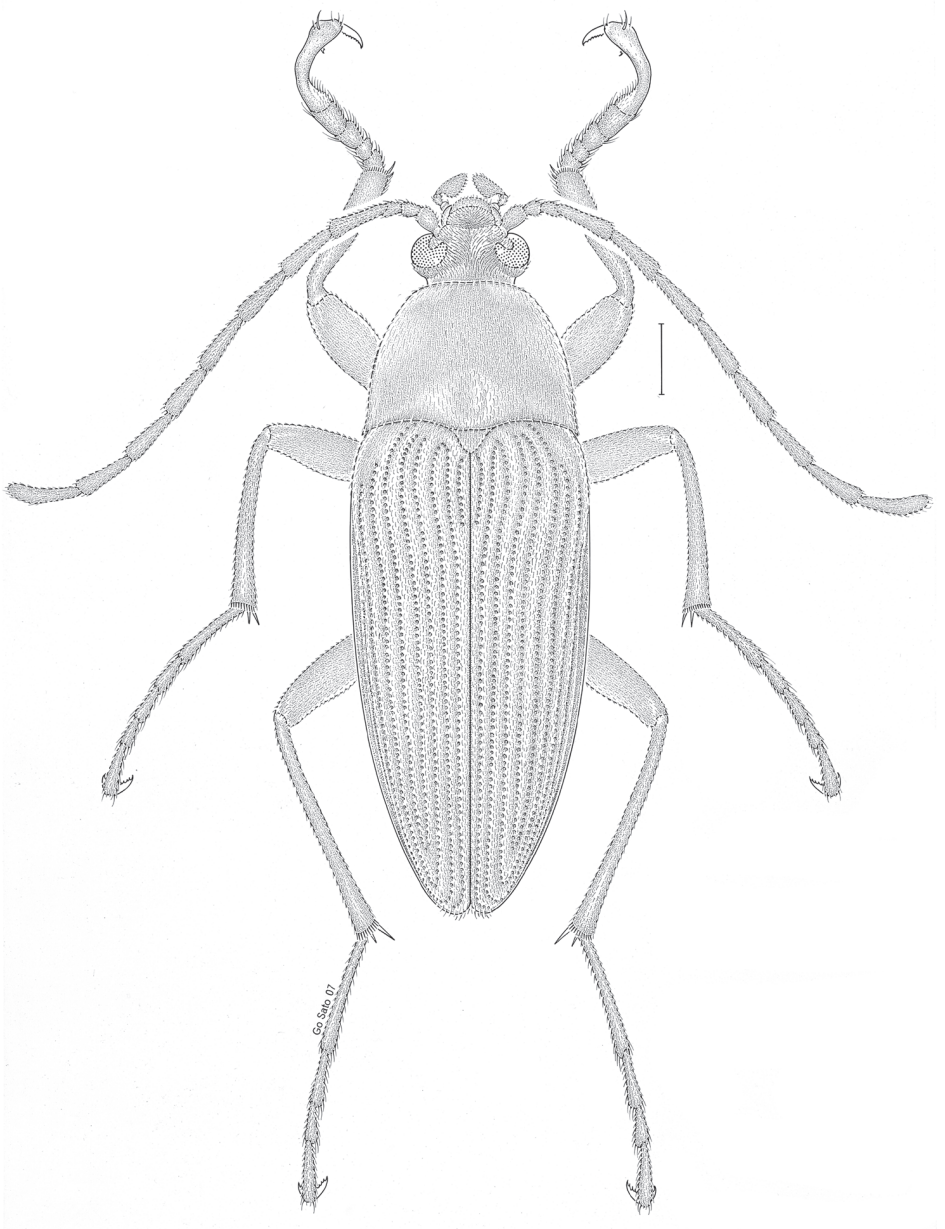
*Androchirus erythropus* (Kirby, 1837). Scale bar = 1 mm.

***Androchirus erythropus* (Kirby, 1837)** [Fig. 32] CAN (MB NB NS ON QC) USA (AL CT DC DE IA IL IN KS KY MA MD MI MN NC NH NJ NY OH PA SC VA VT WI)

*Cistela erythropa* Kirby, 1837: 239.

*Cistela fuscipes* Melsheimer, 1846: 60. Synonymy: Casey (1891: 169).

*Androchirus luteipes* LeConte, 1862a: 245. Synonymy: Horn (1876b: 192).

***Androchirus femoralis* (Olivier, 1791)** USA (FL GA LA MS OH RI SC TX)

*Cistela femoralis* Olivier, 1791: 6.

**Genus *Capnochroa* LeConte, 1862** [F]

*Capnochroa* LeConte, 1862a: 244. Type species: *Cistela fuliginosa* Melsheimer, 1846, monotypy.

***Capnochroa fuliginosa* (Melsheimer, 1846)** CAN (NB NS ON PE QC) USA (CT DC DE GA IA IL IN KY MA MD ME MI MO NC NH NJ NY OH PA RI SC TN VA WI WV)

*Cistela fuliginosa* Melsheimer, 1846: 59.

**Genus *Chromatia* LeConte, 1862** [F]

*Chromatia* LeConte, 1862a: 244. Type species: *Cistela amoena* Say, 1824, monotypy.

***Chromatia amoena* (Say, 1824)** CAN (ON QC) USA (CT GA IN KS MA MD MN NH NJ NY OH PA SC TN TX WI)

*Cistela amoena* Say, 1824a: 268.

**Genus *Isomira* Mulsant, 1856** [F]

*Isomira* Mulsant, 1856: 52. Type species: *Chrysomela murina* Linnaeus, 1758, subsequent designation (C.G. Thomson 1859: 119).

*Tedinus* Casey, 1891: 153. Type species: *Tedinus angustus* Casey, 1891, monotypy. Synonymy: Bousquet and Campbell (1991: 259).

***Isomira acuta* Campbell, 1968** MEX (CI) GUA

*Isomira acuta* Campbell, 1968: 460.

***Isomira alticola* Campbell, 1968** GUA

*Isomira alticola* Campbell, 1968: 465.

***Isomira angusta* (Casey, 1891)** USA (GA SC)

*Tedinus angustus* Casey, 1891: 154.

***Isomira brevicollis* Champion, 1888** MEX (VE)

*Isomira brevicollis* Champion, 1888: 459.

***Isomira championi* Campbell, 1968** MEX (NL)

*Isomira championi* Campbell, 1968: 453.

***Isomira comstocki* Papp, 1956** CAN (AB BC) USA (AZ CA ID NV OR UT WA WY)

*Isomira comstocki* Papp, 1956: 147.

***Isomira damnata* Marshall, 1970** USA (CA)

*Isomira damnata* Marshall, 1970c: 4.

***Isomira evanescens* Champion, 1888** GUA

*Isomira evanescens* Champion, 1888: 458.

***Isomira howdeni hidalgoensis* Campbell, 1968** MEX (HI)

*Isomira howdeni hidalgoensis* Campbell, 1968: 455.

***Isomira howdeni howdeni* Campbell, 1968** MEX (DU)

*Isomira howdeni howdeni* Campbell, 1968: 454.

***Isomira iowensis* Casey, 1891** CAN (ON) USA (AR FL GA IA IL KS MD MO NC OH PA SC TN TX VA)

*Isomira iowensis* Casey, 1891: 145.

***Isomira luscitiosa* Casey, 1891** USA (CA)

*Isomira luscitiosa* Casey, 1891: 148.

***Isomira mexicana* Campbell, 1968** MEX (CI GE OA VE)

*Isomira mexicanus* Campbell, 1968: 457.

***Isomira monticola* Casey, 1891** USA (CA)

*Isomira monticola* Casey, 1891: 150.

***Isomira oblongula* Casey, 1891** CAN (ON QC) USA (FL IL IN MI NC NY OH PA SC TX WI)

*Isomira oblongula* Casey, 1891: 151.

***Isomira obsoleta* Champion, 1888** MEX (GE OA VE) GUA

*Isomira obsoleta* Champion, 1888: 457.

***Isomira pulla* (Melsheimer, 1846)** CAN (ON QC) USA (AL AR DC DE FL GA IA IL IN KY MA MD ME MI MN MS NC NJ NY OH PA RI SC TN VA WI)

*Cistela pulla* Melsheimer, 1846: 60.

*Isomira ignora* Blatchley, 1914: 144. Synonymy: Marshall (1970a: 44).

***Isomira quadristriata* (Couper, 1865)** CAN (MB NB NS ON PE QC SK) USA (CT FL GA IL IN MA MD ME MI MN NC NH NJ NY OH PA RI SC TN VA WI WV)

*Cistela quadristriata* Couper, 1865: 62.

*Isomira velutina* LeConte, 1866b: 139. Synonymy: Casey (1891: 149).

***Isomira rotundata* Campbell, 1968** MEX (SL TA)

*Isomira rotundata* Campbell, 1968: 456.

***Isomira ruficollis* Hamilton, 1893** USA (IN KY OH PA)

*Isomira ruficollis* Hamilton, 1893: 308.

***Isomira sericea* (Say, 1824)** CAN (NB NS ON QC) USA (AR CT DC DE FL GA IA IL IN KY MA MD ME MI MN MO MS NC NH NJ NY OH PA RI SC TN VA WI WV) / BAH

*Cistela sericea* Say, 1824a: 270.

*Isomira tenebrosa* Casey, 1891: 146. Synonymy: Marshall (1970a: 46).

***Isomira subaenea guatemalensis* Campbell, 1968** GUA

*Isomira subaenea guatemalensis* Campbell, 1968: 464.

***Isomira subaenea punctata* Campbell, 1968** GUA

*Isomira subaenea punctata* Campbell, 1968: 464.

***Isomira subaenea soror* Campbell, 1968** GUA

*Isomira subaenea soror* Campbell, 1968: 463.

***Isomira subaenea subaenea* Champion, 1888** MEX (CI) GUA

*Isomira subaenea* Champion, 1888: 458.

***Isomira texana* Casey, 1891** USA (TX)

*Isomira texana* Casey, 1891: 153.

***Isomira valida* Schwarz, 1878** CAN (ON) USA (AL AR FL GA IL IN KS MD NJ OH SC WI WV)

*Isomira valida* Schwarz, 1878: 370.

*Isomira similis* Blatchley, 1910: 1278. Synonymy: Marshall (1970a: 41).

***Isomira variabilis* (Horn, 1875)** USA (AZ CA)

*Cistela variabilis* Horn, 1875: 156.

*Isomira discolor* Casey, 1891: 145. Synonymy: Marshall (1970c: 2).

**Genus *Onychomira* Campbell, 1984** [F]

*Onychomira* Campbell, 1984: 289. Type species: *Onychomira floridensis* Campbell, 1984, original designation.

***Onychomira floridensis* Campbell, 1984** USA (FL)

*Onychomira floridensis* Campbell, 1984: 291.

**Genus *Pseudocistela* Crotch, 1874** [F]

*Pseudocistela* Crotch, 1874: 108. Type species: *Cistela brevis* Say, 1824, subsequent designation (Novák and Pettersson 2008: 327).

***Pseudocistela alternans* (Champion, 1888)** MEX (OA)

*Cistela alternans* Champion, 1888: 456.

***Pseudocistela brevis* (Say, 1824)** CAN (NB ON QC) USA (CT DC FL IL IN MD MI MN MO NC NH NJ NY OH PA SC VA VT WI)

*Cistela brevis* Say, 1824a: 269 [junior primary homonym of *Cistela brevis* Illiger, 1794^65^].

*Cistela erythroptera* Ziegler, 1844: 46. Synonymy: Gemminger [in Gemminger and Harold] (1870: 2047).

***Pseudocistela calida* (Champion, 1888)** PAN

*Cistela calida* Champion, 1888: 453.

***Pseudocistela chiriquensis* (Champion, 1888)** PAN

*Cistela chiriquensis* Champion, 1888: 454.

***Pseudocistela cinerascens* (Champion, 1888)** MEX (PU)

*Cistela cinerascens* Champion, 1888: 453.

***Pseudocistela decepta* (Champion, 1888)** PAN

*Cistela decepta* Champion, 1888: 454.

***Pseudocistela delitescens* (Champion, 1888)** GUA

*Cistela delitescens* Champion, 1888: 455.

***Pseudocistela fragilicornis* (Champion, 1888)** GUA

*Cistela fragilicornis* Champion, 1888: 457.

***Pseudocistela juquilae* (Champion, 1888)** MEX (OA)

*Cistela juquilae* Champion, 1888: 456.

***Pseudocistela marginata* (Ziegler, 1844)** USA (CT GA MA MD NC NJ NY PA)

*Cistela marginata* Ziegler, 1844: 46.

***Pseudocistela nigricornis* (Champion, 1888)** MEX (CI DU GE GU VE) NIC CRI PAN

*Cistela nigricornis* Champion, 1888: 452.

***Pseudocistela occulta* (Champion, 1888)** GUA

*Cistela occulta* Champion, 1888: 455.

***Pseudocistela opaca* (LeConte, 1859)** USA (CA ID NV)

*Xystropus opacus* LeConte, 1859b: 78.

*Cistela thevenetii* Horn, 1875: 156. Synonymy: Poole and Gentili (1996: 441) [probably based on Marshall (1964: 87) unpublished thesis].

***Pseudocistela ovipennis* (Champion, 1893)** MEX (GE JA)

*Cistela ovipennis* Champion, 1893a: 569.

***Pseudocistela pectinata* Hopping, 1933** CAN (BC)

*Pseudocistela pectinata* Hopping, 1933: 285.

***Pseudocistela pinguis* (LeConte, 1859)** CAN (BC) USA (CA CO ID NM NV OR WA)

*Xystropus pinguis* LeConte, 1859a: 16.

*Pseudocistela pacifica* Hopping, 1933: 284. Synonymy: Bousquet and Campbell (1991: 260).

***Pseudocistela sandersoni* Campbell, 1971** JAM

*Pseudocistela sandersoni* Campbell, 1971: 17.

***Pseudocistela zunilensis* (Champion, 1888)** GUA

*Cistela zunilensis* Champion, 1888: 452.

**Subtribe Mycetocharina Gistel, 1848**

Mycetocharisidae Gistel, 1848: [10]. Type genus: *Mycetochara* Guérin-Méneville, 1827.

**Genus *Hymenochara* Campbell, 1978** [F]

*Hymenochara* Campbell, 1978a: 435. Type species: *Mycetophila rufipes* J.E. LeConte, 1824, original designation.

***Hymenochara arizonensis* Campbell, 1978** USA (AZ)

*Hymenochara arizonensis* Campbell, 1978a: 440.

***Hymenochara rufipes* (J.E. LeConte, 1824)** CAN (ON QC) USA (AL FL IN KY MA MD MI MO MS NC NY OH PA SC)

*Mycetophila rufipes* J.E. LeConte, 1824: 170.

**Genus *Mycetochara* Guérin-Méneville, 1827** [F]

*Mycetophila* Gyllenhal, 1810: 541 [junior homonym of *Mycetophila* Meigen, 1803]. Type species: *Cistela scapularis* Illiger, 1805 (= *Cistela humeralis* Fabricius, 1787), subsequent designation (Westwood 1838: 32).

*Mycetochara* Guérin-Méneville, 1827: 346. Replacement name for *Mycetophila* Gyllenhal, 1810. Note. See Bousquet et al. (2015: 138) for authorship of this name.

*Mycetocharis* Gyllenhal, 1827: 510. Replacement name for *Mycetophila* Gyllenhal, 1810.

*Mycetochares* Latreille, 1829a: 42. Replacement name for *Mycetophila* Gyllenhal, 1810.

*Stigmatoma* LeConte, 1862a: 244. Type species: *Cistela fraterna* Say, 1824, monotypy. Synonymy: LeConte (1866b: 139).

***Mycetochara analis* (LeConte, 1878)** CAN (AB BC MB NB ON QC SK) USA (AR AZ CO GA IN KS MA MD MI NE NJ NY OH OR PA SC WA WI)

*Mycetochares analis* LeConte, 1878b: 618.

*Mycetochares lugubris* LeConte, 1878b: 618. Synonymy: Campbell (1978b: 931).

*Mycetochara horni* Dury, 1902: 172. Synonymy: Campbell (1978b: 931).

*Mycetochara davisi* Hatch, 1965: 187. Synonymy: Campbell (1978b: 931).

***Mycetochara basillaris* (Say, 1824)** USA (PA)

*Cistela basillaris* Say, 1824a: 269.

**Figure 33.**
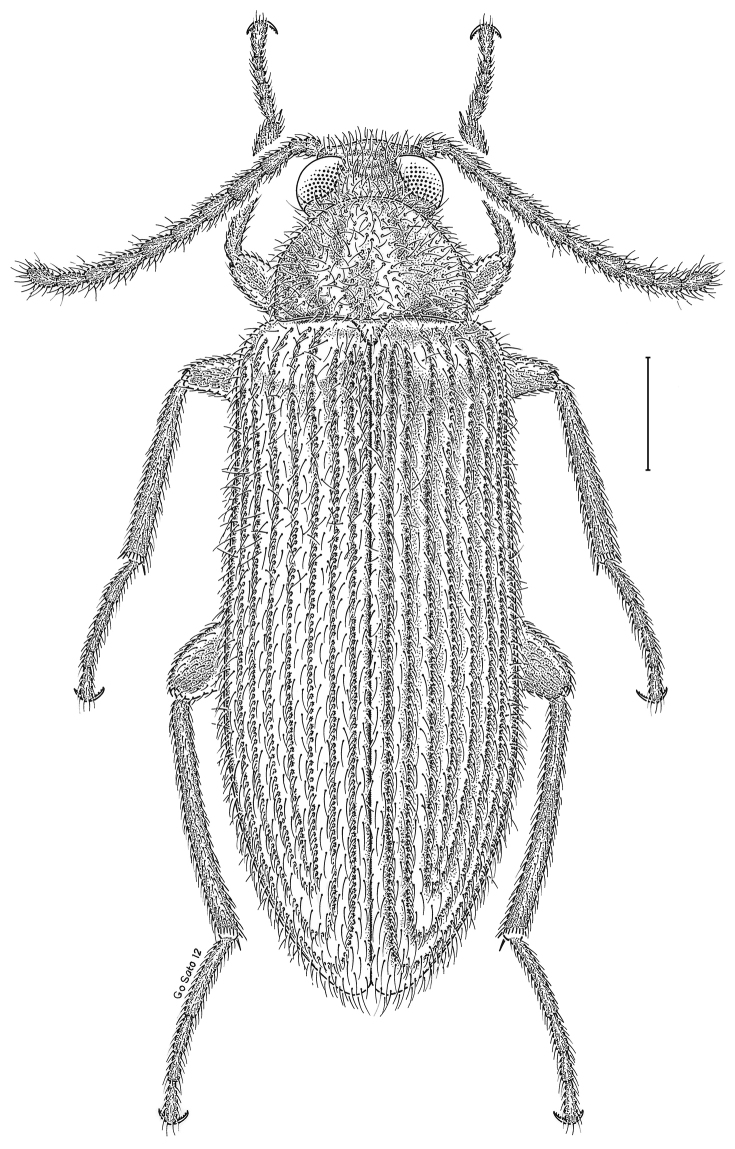
*Mycetochara bicolor* (Couper, 1865). Scale bar = 1 mm.

***Mycetochara bicolor* (Couper, 1865)** [Fig. 33] CAN (NB NS ON QC) USA (IA IN MA ME MI MO NC NH NY PA SC VA VT WI)

*Mycetochares bicolor* Couper, 1865: 62.

***Mycetochara binotata* (Say, 1824)** CAN (MB NB NS ON QC) USA (CT IN KY MA MD MI MN NC NH NY OH PA SC VA WI)

*Cistela binotata* Say, 1824b: 285.

*Mycetochares marginata* LeConte, 1878b: 618. Synonymy: Campbell (1978b: 932).

*Mycetochares longula* LeConte, 1878b: 618. Synonymy: Campbell (1978b: 932).

***Mycetochara foveata* (LeConte, 1866)** CAN (NB ON QC) USA (IA IL IN MA MD MI MO NC NH NJ NY OH RI SC VA WI)

*Mycetochares foveata* LeConte, 1866b: 140.

*Mycetochares tenuis* LeConte, 1866b: 140. Synonymy: Campbell (1978b: 929).

*Mycetochares gracilis* LeConte, 1878b: 615 [junior primary homonym of *Mycetochares gracilis* Falderman, 1837]. Synonymy: Campbell (1978b: 929).

*Mycetochara gilvipes* Casey, 1891: 131. Synonymy: Campbell (1978b: 929).

*Mycetochara lecontei* Borchmann, 1909: 714. Replacement name for *Mycetochara gracilis* (LeConte, 1878).

***Mycetochara fraterna* (Say, 1824)** CAN (AB BC MB NB NS ON QC SK) USA (CT DC IA IL IN MA MD ME NC NH NJ NY OH PA SC SD VA WI)

*Cistela fraterna* Say, 1824a: 270.

*Mycetochares laticollis* LeConte, 1878b: 617. Synonymy: Casey (1891: 128).

*Mycetochara megalops* Casey, 1891: 129. Synonymy: Campbell (1978b: 925).

*Mycetochara nigerrima* Casey, 1891: 132. Synonymy: Campbell (1978b: 925).

***Mycetochara haldemani* (LeConte, 1866)** USA (FL GA IL IN MD NC NY OH PA VA WI)

*Mycetochares haldemani* LeConte, 1866b: 140.

***Mycetochara lata* Hatch, 1965** USA (OR)

*Mycetochara lata* Hatch, 1965: 189.

***Mycetochara perplexata* Marshall, 1970** USA (CA)

*Mycetochara perplexata* Marshall, 1970b: 3.

*Mycetochara marshalli* Campbell, 1978b: 934. **New synonymy** [YB].

***Mycetochara procera* Casey, 1891** CAN (BC) USA (AZ CA ID NV OR WA)

*Mycetochara procera* Casey, 1891: 140.

*Mycetochara pacifica* Casey, 1891: 139. Synonymy: Campbell (1978b: 936).

*Mycetochara nevadensis* Casey, 1891: 142. Synonymy: Campbell (1978b: 936).

*Mycetochara crassulipes* Casey, 1891: 142. Synonymy: Campbell (1978b: 936).

*Mycetochara downei* Hatch, 1965: 187. Synonymy: Campbell (1978b: 937).

*Mycetochara angusta* Hatch, 1965: 188. Synonymy: Campbell (1978b: 937).

*Mycetochara malkini* Hatch, 1965: 188. Synonymy: Campbell (1978b: 937).

*Mycetochara caseyi* Hatch, 1965: 188. Synonymy: Campbell (1978b: 937).

***Mycetochara pubipennis* (LeConte, 1878)** USA (CA)

*Mycetochares pubipennis* LeConte, 1878b: 617.

*Mycetochara longipennis* Casey, 1891: 139. Synonymy: Campbell (1978b: 935).

***Mycetochara ruficornis* Melsheimer, 1846** USA (PA)

*Mycetocharus ruficornis* Melsheimer, 1846: 59.

**Subtribe Xystropodina Solier, 1835**

Xystropides Solier, 1835a: 229. Type genus: *Xystropus* Solier, 1835.

Lystronychides Lacordaire, 1859: 512. Type genus: *Lystronychus* Latreille, 1829.

**Genus *Anamphidora* Casey, 1924** [F]

*Anamphidora* Casey, 1924: 330. Type species: *Anamphidora parvula* Casey, 1924, original designation.

***Anamphidora campbelli* Marshall, 1967** USA (TX)

*Anamphidora campbelli* Marshall, 1967b: 209.

***Anamphidora kimberleei* Marshall, 1970** MEX (BC)

*Anamphidora kimberleei* Marshall, 1970d: 294.

***Anamphidora parvula* Casey, 1924** MEX (DU)

*Anamphidora parvula* Casey, 1924: 330.

**Genus *Cteisa* Solier, 1835** [F]

*Cteisa* Solier, 1835a: 242. Type species: *Cteisa hirta* Solier, 1835, monotypy.

***Cteisa pedinoides* Mäklin, 1875** MEX (VE) GUA PAN / JAM / SA

*Cteisa pedinoides* Mäklin, 1875b: 681.

**Genus *Erxias* Champion, 1888** [M]

*Erxias* Champion, 1888: 460. Type species: *Erxias bicolor* Champion, 1888, subsequent designation (Bousquet et al. 2015: 138).

***Erxias bicolor* Champion, 1888** PAN / SA

*Erxias bicolor* Champion, 1888: 460.

***Erxias violaceipennis* Champion, 1888** NIC

*Erxias violaceipennis* Champion, 1888: 460.

**Genus *Lystronychus* Latreille, 1829** [M]

*Lystronychus* Latreille, 1829a: 41 [as *Lystronichus*]. Type species: *Helops equestris* Fabricius, 1775, subsequent designation (Saunders 1836: 154). Note. *Lystronychus* is in prevailing usage (see Bouchard et al. 2011: 427) and so deemed to be the correct original spelling.

**Subgenus Lystronychus Latreille, 1829**

*Lystronychus* Latreille, 1829a: 41 [as *Lystronichus*]. Type species: *Helops equestris* Fabricius, 1775, subsequent designation (Saunders 1836: 154).

***Lystronychus championi* Horn, 1894** USA (TX)

*Lystronychus championi* Horn, 1894b: 433.

***Lystronychus delauneyi* (Fleutiaux and Sallé, 1890)** LAN (Guadeloupe)

*Anaedus delauneyi* Fleutiaux and Sallé, 1890: 428.

***Lystronychus piliferus* Champion, 1888** USA (TX) MEX (CI CL JA OA PU VE) GUA NIC / SA

*Lystronychus piliferus* Champion, 1888: 462.

***Lystronychus purpureipennis* Champion, 1888** GUA

*Lystronychus purpureipennis* Champion, 1888: 463.

***Lystronychus rufonotatus* Champion, 1896** LAN (St. Vincent)

*Lystronychus rufonotatus* Champion, 1896: 35.

***Lystronychus rufulus* Borchmann, 1930** MEX (PU)

*Lystronychus rufulus* Borchmann, 1930: 98.

***Lystronychus scapularis* Champion, 1888** USA (AZ) MEX (YU) GUA NIC PAN

*Lystronychus scapularis* Champion, 1888: 463.

***Lystronychus tuberculifer* Champion, 1896** LAN (Grenadines)

*Lystronychus tuberculifer* Champion, 1896: 34.

**Genus *Prostenus* Klug, 1829** [M]

*Prostenus* Klug, 1829: 5. Type species (suggested): *Prostenus periscelis* Perty, 1830 (see Bousquet et al. 2015: 139).

*Mecocerus* Solier, 1835a: 241 [junior homonym of *Mecocerus* Schönherr, 1833]. Type species: *Xystropus dejeanii* Solier, 1835, monotypy. Synonymy: Lacordaire (1859: 513).

***Prostenus panamensis* Champion, 1888** PAN

*Prostenus panamensis* Champion, 1888: 461.

**Genus *Xystropus* Solier, 1835** [M]

*Xystropus* Solier, 1835a: 241. Type species: *Xystropus pilosus* Solier, 1835, monotypy. Note. See Bousquet et al. (2015: 139) for available species originally included in this genus.

***Xystropus californicus* (Horn, 1868)** MEX (OA) NIC CRI PAN / SA

*Prostenus californicus* Horn, 1868: 138.

*Xystropus fulgidus* Mäklin, 1875b: 680. Synonymy: Casey (1891: 74).

***Xystropus fallax* Mäklin, 1875** PAN / SA

*Xystropus fallax* Mäklin, 1875b: 677.

***Xystropus lebasii* Mäklin, 1875** PAN / SA

*Xystropus lebasii* Mäklin, 1875b: 679.

**Subfamily DIAPERINAE Latreille, 1802**

Diaperialae Latreille, 1802: 161. Type genus: *Diaperis* Geoffroy, 1762.

**Tribe Crypticini Brullé, 1832**

Crypticites Brullé, 1832: 190. Type genus: *Crypticus* Latreille, 1816.

**Genus *Ellipsodes* Wollaston, 1854** [M]

*Ellipsodes* Wollaston, 1854: 485. Type species: *Sphaeridium glabratum* Fabricius, 1781, monotypy.

**Subgenus Anthrenopsis Koch, 1950**

*Anthrenopsis* Koch, 1950: 74. Type species: *Platydema scriptipenne* Fairmaire, 1875 (= *Basides ziczac* Motschulsky, 1873), original designation.

***Ellipsodes ziczac* (Motschulsky, 1873)** LAN (Guadeloupe, Grenada) – Adventive

*Basides ziczac* Motschulsky, 1873: 475.

*Platydema scriptipenne* Fairmaire, 1875: xxxiii. Synonymy: Kaszab (1975: 102).

**Genus *Gondwanocrypticus* Español, 1955** [M]

*Gondwanocrypticus* Español, 1955: 10. Type species: *Crypticus platensis* Fairmaire, 1884, original designation.

***Gondwanocrypticus aterrimus* (Champion, 1886)** MEX (CI) GUA BEL SAL HON NIC CRI PAN / SA

*Crypticus aterrimus* Champion, 1886: 138.

***Gondwanocrypticus filicornis* (Chevrolat, 1878)** JAM DOM LAN

*Platydema filicorne* Chevrolat, 1878b: 222.

***Gondwanocrypticus maculatus* (Champion, 1886)** MEX (MO) GUA NIC

*Crypticus maculatus* Champion, 1886: 138.

***Gondwanocrypticus mexicanus* (Champion, 1886)** MEX (VE)

*Crypticus mexicanus* Champion, 1886: 137.

***Gondwanocrypticus obsoletus* (Say, 1824)** USA (DE FL GA LA MD MS NC SC TX VA) / CUB

*Crypticus obsoletus* Say, 1824a: 265.

***Gondwanocrypticus ovatus* (Champion, 1886)** MEX (JA OA) GUA

*Crypticus ovatus* Champion, 1886: 137.

***Gondwanocrypticus pictus* (Gebien, 1928)** USA_i_ (AL FL GA MS NC SC) / SA

*Crypticus pictus* Gebien, 1928a: 118.

***Gondwanocrypticus platensis* (Fairmaire, 1884)** USA (AL CA DE FL GA LA MD MS NC SC TX VA WV) / BAH CAY / SA – Adventive

*Crypticus platensis* Fairmaire, 1884: 510.

***Gondwanocrypticus undatus* (Champion, 1896)** LAN

*Crypticus undatus* Champion, 1896: 5.

**Genus *Poecilocrypticus* Gebien, 1928** [M]

*Poecilocrypticus* Gebien, 1928a: 121. Type species: *Poecilocrypticus formicophilus* Gebien, 1928, monotypy.

***Poecilocrypticus formicophilus* Gebien, 1928** USA_i_ (AL FL GA LA MS NC OK SC TX) / BAH / SA

*Poecilocrypticus formicophilus* Gebien, 1928a: 122.

**Tribe Diaperini Latreille, 1802**

Diaperialae Latreille, 1802: 161. Type genus: *Diaperis* Geoffroy, 1762.

**Subtribe Adelinina LeConte, 1862**

Alphitophagida Gistel, 1856b: 185^66^. Type genus: *Alphitophagus* Stephens, 1832.

Adelinini LeConte, 1862a: 237. Type genus: Adelina Dejean, 1835.

Schedarosini Reitter, 1876: 42. Type genus: *Schedarosus* Reitter, 1876 (= Adelina Dejean, 1835).

Doliemini Reitter, 1917: 58. Type genus: *Doliema* Pascoe, 1860 (= Adelina Dejean, 1835).

Gnathocerini Skopin, 1978: 228. Type genus: *Gnatocerus* Thunberg, 1814.

**Genus Adelina Dejean, 1835** [F]

Adelina Dejean, 1835: 315. Type species: *Cucujus planus* Fabricius, 1801, monotypy.

*Doliema* Pascoe, 1860: 50. Type species: *Doliema platisoides* Pascoe, 1860, monotypy. Synonymy: Fleutiaux and Sallé (1890: 428, as Adelina LeConte).

*Schedarosus* Reitter, 1876: 42. Type species: *Schedarosus cucujiformis* Reitter, 1876 (= *Pytho pallida* Say, 1823), subsequent designation (Löbl et al. 2008b: 42). Synonymy (with *Doliema* Pascoe): Champion (1886: 157).

***Adelina angustata* (Champion, 1886)** GUA NIC

*Doliema angustata* Champion, 1886: 159.

***Adelina bacardi* Steiner, 2006** BAH

*Adelina bacardi* Steiner, 2006: 15.

***Adelina bidens* (Schaeffer, 1915)** USA (FL TX) GUA / BAH CUB CAY DOM

*Doliema bidens* Schaeffer, 1915: 238.

***Adelina bifurcata* (Champion, 1893)** USA (AZ) MEX (BS JA OA VE YU) CRI

*Doliema bifurcata* Champion, 1893a: 535.

***Adelina dominicana* (Ardoin, 1977)** DOM

*Doliema dominicana* Ardoin, 1977a: 18.

***Adelina frontalis* (Champion, 1886)** BEL / SA

*Doliema frontalis* Champion, 1886: 159.

***Adelina klapperichi* (Ardoin, 1977)** DOM

*Doliema klapperichi* Ardoin, 1977a: 12.

***Adelina latiramosa* Doyen, 1984** MEX (PU)

*Adelina latiramosa* Doyen, 1984a: 777.

***Adelina maryjoae* Steiner, 2005** BAH CAY

*Adelina maryjoae* Steiner, 2005: 449.

***Adelina mystax* Triplehorn and Ivie, 1983** VIS

*Adelina mystax* Triplehorn and Ivie, 1983: 272.

***Adelina pallida* (Say, 1824)** USA (CA FL GA IN LA MD NC OH SC TN VA) MEX (CH HI VE YU) GUA BEL NIC / CUB PRI / SA

*Pytho pallida* Say, 1824a: 271.

*Schedarosus cucujiformis* Reitter, 1876: 43. Synonymy: Champion (1893a: 535).

***Adelina pici* (Ardoin, 1977)** BAH CUB CAY LAN / SA

*Doliema pici* Ardoin, 1977a: 7.

***Adelina plana* (Fabricius, 1801)** USA (AZ CA FL IN NC) MEX (JA TB VE) GUA BEL HON NIC PAN / BAH CUB CAY DOM LAN / SA

*Cucujus planus* Fabricius, 1801b: 94.

*Adelina depressa* Erichson, 1847: 119. Synonymy (in doubt): Champion (1893a: 535).

*Adelina plana* LeConte, 1851: 149 [junior secondary homonym of *Adelina plana* (Fabricius, 1801)]. Synonymy (in doubt): Champion (1886: 157).

*Sitophagus lecontei* Horn, 1870: 346. Replacement name for *Sitophagus planus* (LeConte, 1851).

*Schedarosus scidarius* Reitter, 1876: 44. Synonymy: Champion (1886: 158).

*Doliema diabolica* Pic, 1923: 24. Synonymy: Ardoin (1977a: 3).

***Adelina quadridentata* (Champion, 1893)** MEX (JA OA) CRI

*Doliema quadridentata* Champion, 1893a: 535.

**Genus *Alphitophagus* Stephens, 1832** [M]

*Alphitophagus* Stephens, 1832: 12. Type species: *Alphitophagus quadripustulatus* Stephens, 1832 (= *Diaperis bifasciata* Say, 1824), monotypy.

*Phyletes* Redtenbacher, 1845: 128. Type species: *Phylethus populi* Redtenbacher, 1848 (= *Diaperis bifasciata* Say, 1824), subsequent monotypy in Redtenbacher (1848: 589, as *Phylethus*). Synonymy: Seidlitz (1894: 533).

**Figure 34.**
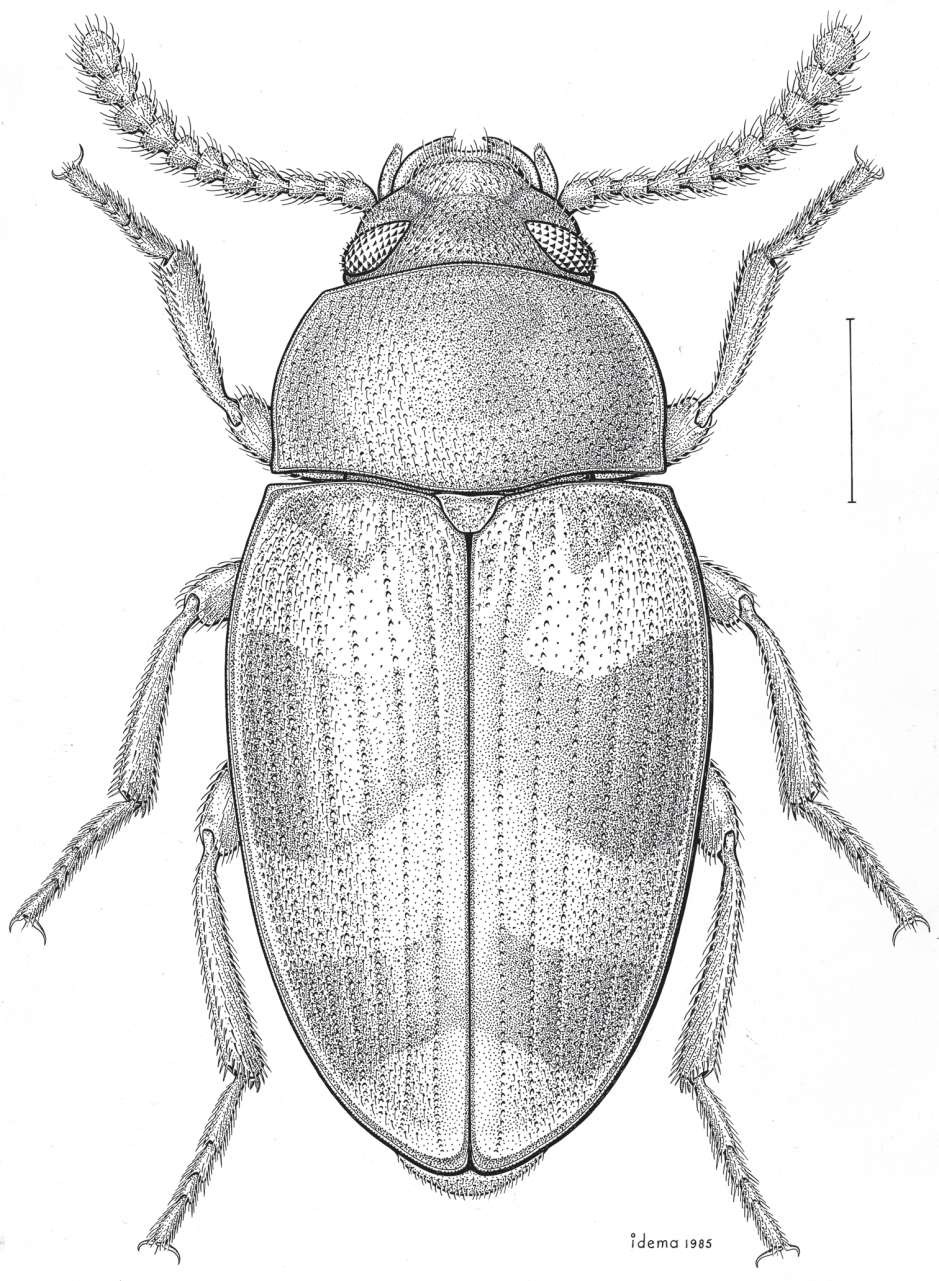
*Alphitophagus bifasciatus* (Say, 1824). Scale bar = 1 mm.

***Alphitophagus bifasciatus* (Say, 1824)** [Fig. 34] CAN (MB ON QC SK) USA (AL AR CA DC GA IA ID IL IN KS LA MD MN MO MS NC NE NJ NY OH OR PA SC SD TN TX VA WA WI) – Adventive

*Diaperis bifasciata* Say, 1824a: 268.

*Alphitophagus quadripustulatus* Stephens, 1832: 12. Synonymy: Horn (1870: 385).

*Platydema lilliputanum* Carter, 1937: 130. Synonymy: Hinton (1947b: 90).

**Genus *Cynaeus* LeConte, 1862** [M]

*Cynaeus* LeConte, 1862a: 233. Type species: *Platydema angustum* LeConte, 1851, original designation.

**Figure 35.**
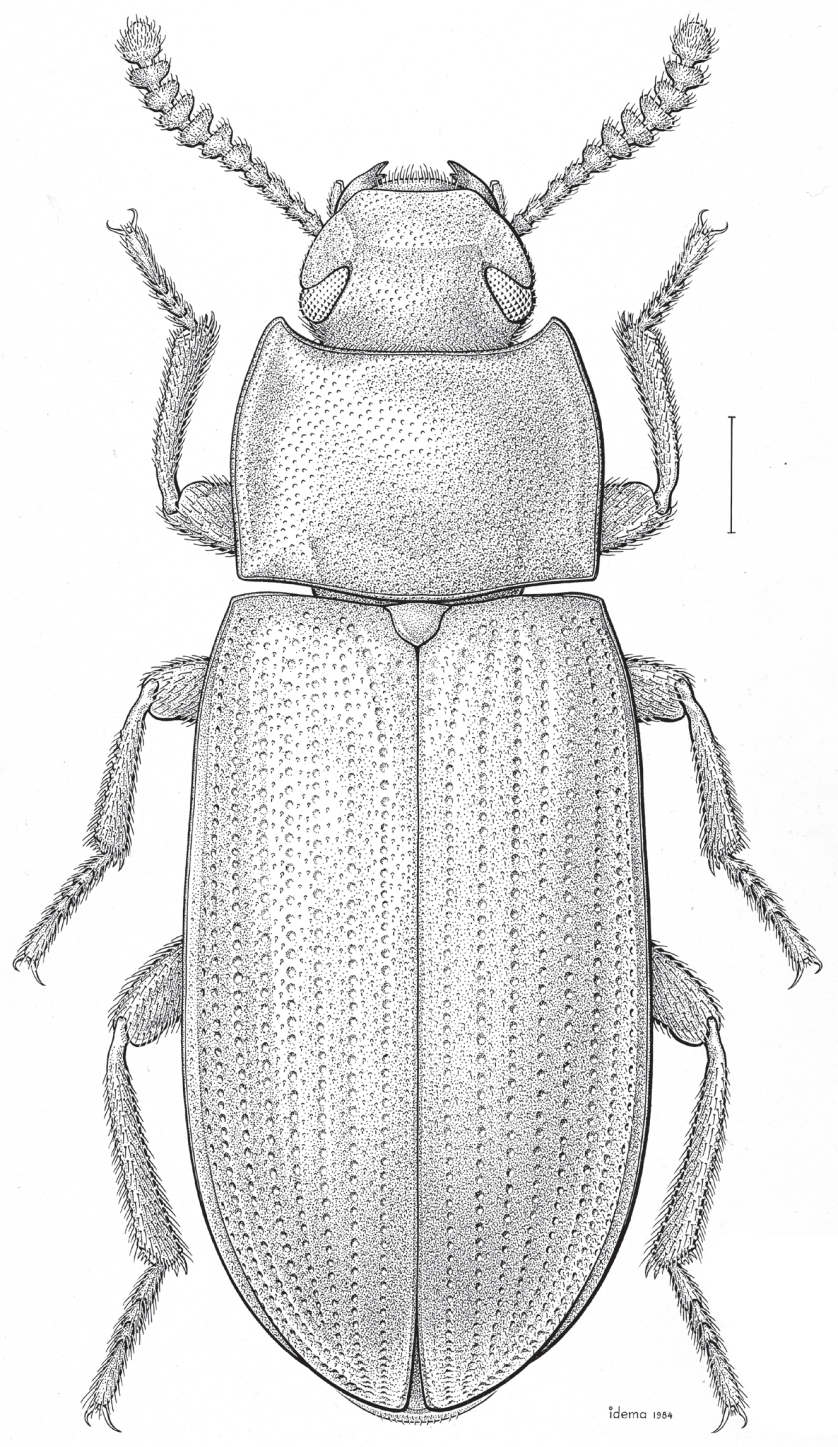
*Cynaeus angustus* (LeConte, 1851). Scale bar = 1 mm.

***Cynaeus angustus* (LeConte, 1851)** [Fig. 35] CAN (AB BC MB ON QC SK) USA (AZ CA CO GA IA ID IL IN KS MD MI MN NC ND NM OH OR SD TN TX UT VA WA WI WY) MEX (BC SO)

*Platydema angustum* LeConte, 1851: 149.

*Cynaeus opacus* Champion, 1886: 156. Synonymy: Blaisdell (1943: 267).

***Cynaeus depressus* Horn, 1870** USA (AZ CA) MEX (BC)

*Cynaeus depressus* Horn, 1870: 369.

**Genus *Doliodesmus* Spilman, 1967** [M]

*Doliodesmus* Spilman, 1967: 149. Type species: *Doliodesmus charlesi* Spilman, 1967, monotypy.

***Doliodesmus charlesi* Spilman, 1967** USA (AZ) MEX (BC BS)

*Doliodesmus charlesi* Spilman, 1967: 153.

**Genus *Doliopines* Horn, 1894** [M]

*Doliopines* Horn, 1894b: 427. Type species: *Doliopines cucujinus* Horn, 1894, monotypy.

***Doliopines cucujinus* Horn, 1894** MEX (BC BS SO)

*Doliopines cucujinus* Horn, 1894b: 428.

**Genus *Gnatocerus* Thunberg, 1814** [M]

*Gnatocerus* Thunberg, 1814: 47. Type species: *Gnatocerus ruber* Thunberg, 1814 (= *Trogosita cornuta* Fabricius, 1798), monotypy.

*Gnathocerus* Agassiz, 1846: 164. Unjustified emendation of *Gnatocerus* Thunberg, 1814, not in prevailing usage.

**Subgenus Echocerus Horn, 1870**

*Echocerus* Horn, 1870: 366. Type species: *Trogosita maxillosa* Fabricius, 1801, monotypy.

***Gnatocerus analis* (Champion, 1886)** GUA

*Echocerus analis* Champion, 1886: 146.

***Gnatocerus angelicus* (Blaisdell, 1923)** MEX (BC)

*Echocerus angelicus* Blaisdell, 1923: 277.

***Gnatocerus breviceps* (Blaisdell, 1943)** MEX (BS)

*Echocerus breviceps* Blaisdell, 1943: 266.

***Gnatocerus curvicornis* (Champion, 1893)** USA (FL) MEX (JA OA YU) / BAH CUB CAY / LAN

*Echocerus curvicornis* Champion, 1893a: 533.

*Echocerus recurvatus* Chittenden, 1895a: 2. Synonymy: Chittenden (1895b: 331).

***Gnatocerus maxillosus* (Fabricius, 1801)** USA (CA FL GA KS MD MI OH SC TX WI) MEX (GU) GUA NIC / CUB PRI LAN / SA – Adventive

*Trogosita maxillosa* Fabricius, 1801a: 155.

**Subgenus Gnatocerus Thunberg, 1814**

*Gnatocerus* Thunberg, 1814: 47. Type species: *Gnatocerus ruber* Thunberg, 1814 (= *Trogosita cornuta* Fabricius, 1798), monotypy.

*Cerandria* Dejean, 1834: 200. Type species: *Trogosita cornuta* Fabricius, 1798, subsequent designation (Duponchel 1841: 285). Synonymy: Schaum (1849: 283).

*Sicinus* Champion, 1886: 146. Type species: *Sicinus guatemalensis* Champion, 1886, subsequent designation (Löbl et al. 2008b: 43). Synonymy: Leng (1920: 233).

***Gnatocerus brevipes* (Champion, 1886)** GUA

*Sicinus brevipes* Champion, 1886: 147.

**Figure 36.**
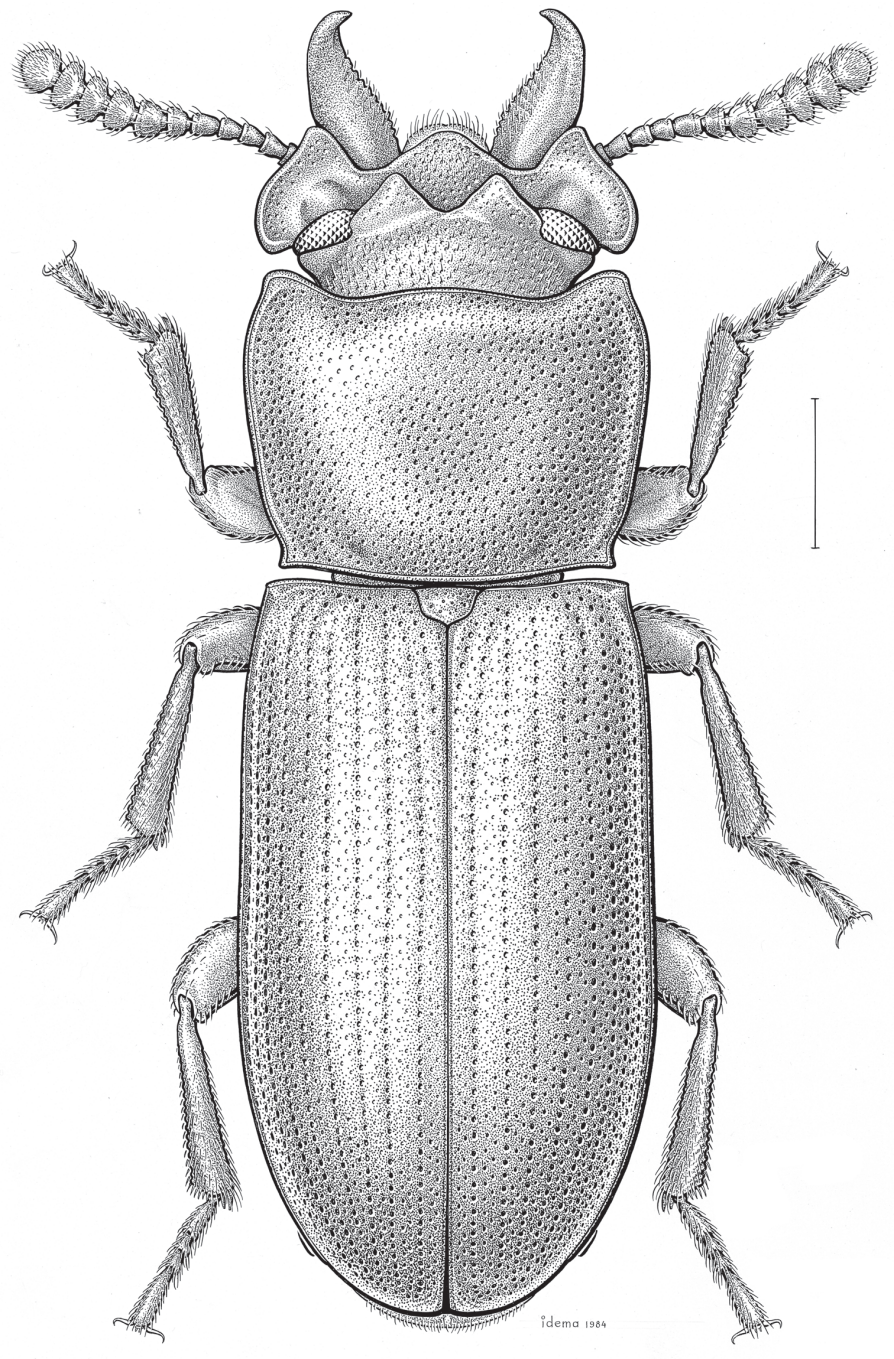
Gnatocerus (Gnatocerus) cornutus (Fabricius, 1798). Scale bar = 1 mm.

***Gnatocerus cornutus* (Fabricius, 1798)** [Fig. 36] CAN (BC MB NS ON QC) USA (CA CT FL MA MD OH OR WA) MEX (GU VE) GUA / CUB PRI – Adventive

*Trogosita cornuta* Fabricius, 1798: 51.

*Trogossita maxillaris* Palisot de Beauvois, 1812: 125; pl. 32 (fig. 4)^67^. Synonymy: Fauvel (1904: 174).

***Gnatocerus guatemalensis* (Champion, 1886)** USA (FL IL KY MD OH PA TX) MEX GUA / BAH CUB LAN

*Sicinus guatemalensis* Champion, 1886: 147.

*Echocerus dentiger* Chittenden, 1895a: 1. Synonymy: Leng (1918: 208).

**Genus *Iccius* Champion, 1886** [M]

*Iccius* Champion, 1886: 147. Type species: *Iccius cephalotes* Champion, 1886, subsequent designation (Gebien 1940: 760).

***Iccius cephalotes* Champion, 1886** MEX (VE) GUA PAN / CUB

*Iccius cephalotes* Champion, 1886: 148.

***Iccius cylindricus* Champion, 1886** USA (AZ LA TX) MEX (MO) GUA

*Iccius cylindricus* Champion, 1886: 148.

***Iccius elongatus* Kulzer, 1949** CRI

*Iccius elongatus* Kulzer, 1949: 304.

***Iccius grenadensis* Champion, 1896** LAN

*Iccius grenadensis* Champion, 1896: 19.

***Iccius monoceros* Ferrer and Ødegaard, 2005** PAN

*Iccius monoceros* Ferrer and Ødegaard, 2005: 637.

***Iccius rufotestaceus* Champion, 1896** LAN

*Iccius rufotestaceus* Champion, 1896: 18.

*Hypophlaeus dufaui* Pic, 1945: 7. Synonymy: Bremer and Triplehorn (1999: 59).

**Genus *Loxostethus* Triplehorn, 1962** [M]

*Loxostethus* Triplehorn, 1962: 504. Type species: *Loxostethus fasciatus* Triplehorn, 1962, original designation.

***Loxostethus erythroscelis* Triplehorn and Merkl, 1997** HAI DOM

*Loxostethus erythroscelis* Triplehorn and Merkl, 1997: 739.

***Loxostethus fasciatus* Triplehorn, 1962** CUB

*Loxostethus fasciatus* Triplehorn, 1962: 504.

*Loxostethus quadrimaculata* Zayas, 1988: 93. Synonymy: Ivie (1991: 400).

***Loxostethus gibbosus* Triplehorn and Merkl, 1997** CUB

*Loxostethus gibbosus* Triplehorn and Merkl, 1997: 738.

***Loxostethus gowdeyi* (Pic, 1930)** CUB JAM HAI DOM

*Pentaphyllus gowdeyi* Pic, 1930: 33.

*Loxostethus jamaicensis* Triplehorn, 1962: 506. Synonymy: Triplehorn and Merkl (1997: 739).

*Loxostethus opacifrons* Triplehorn, 1962: 506. Synonymy: Triplehorn and Merkl (1997: 739).

*Heterophylus meszarosi* Kaszab, 1977a: 123. Synonymy: Triplehorn and Merkl (1997: 739).

*Loxostethus baracoae* Garrido and Gutiérrez, 1995a: 7. Synonymy: Triplehorn and Merkl (1997: 739).

***Loxostethus guadeloupensis* (Kaszab, 1977)** LAN (Guadeloupe)

*Heterophylus guadeloupensis* Kaszab, 1977a: 122.

***Loxostethus oblongus* Triplehorn and Merkl, 1997** DOM

*Loxostethus oblongus* Triplehorn and Merkl, 1997: 737.

***Loxostethus unicolor* Triplehorn, 1962** HAI PRI

*Loxostethus unicolor* Triplehorn, 1962: 506.

*Heterophylus ruficornis* Kaszab, 1981: 80. Synonymy: Triplehorn and Merkl (1997: 737).

**Genus *Mophis* Champion, 1886** [M]

*Mophis* Champion, 1886: 168. Type species: *Mophis marginicollis* Champion, 1886, subsequent designation (Gebien 1940: 1061).

***Mophis affinis* Champion, 1886** MEX (GE OA VE)

*Mophis affinis* Champion, 1886: 169.

*Mophis aterrimus* Champion, 1886: 169. Synonymy: Champion (1893a: 536).

***Mophis cynaeoides* (Champion, 1886)** MEX (DU FD VE)

*Sitophagus cynaeoides* Champion, 1886: 162.

***Mophis marginicollis* Champion, 1886** GUA

*Mophis marginicollis* Champion, 1886: 169.

**Genus *Phayllus* Champion, 1886** [M]

*Phayllus* Champion, 1886: 167. Type species: *Phayllus minutus* Champion, 1886, monotypy.

***Phayllus minutus* Champion, 1886** MEX (VE) GUA BEL NIC PAN / SA

*Phayllus minutus* Champion, 1886: 167.

**Genus *Saptine* Champion, 1886** [F]

*Saptine* Champion, 1886: 180. Type species: *Saptine ovata* Champion, 1886, monotypy.

***Saptine ovata* Champion, 1886** MEX (VE)

*Saptine ovata* Champion, 1886: 181.

**Genus *Sitophagus* Mulsant, 1854** [M]

*Sitophagus* Mulsant, 1854: 264. Type species: *Sitophagus solieri* Mulsant, 1854 (=*Uloma hololeptoides* Laporte, 1840), monotypy.

***Sitophagus alveolatus* Doyen, 1984** USA (AZ)

*Sitophagus alveolatus* Doyen, 1984a: 779.

***Sitophagus dilatifrons* Champion, 1886** GUA PAN

*Sitophagus dilatifrons* Champion, 1886: 162.

***Sitophagus fuliginosus* Champion, 1886** GUA

*Sitophagus fuliginosus* Champion, 1886: 161.

***Sitophagus hololeptoides* (Laporte, 1840)** USA (AZ CA FL TX) MEX (DU PU VE YU) GUA BEL NIC CRI PAN / BAH CUB PRI LAN / SA

*Uloma hololeptoides* Laporte, 1840: 220.

*Sitophagus solieri* Mulsant, 1854: 265. Synonymy: Champion (1886: 161).

*Adelina farinaria* Wollaston, 1858: 414. Synonymy (with*S. solieri* Mulsant): Bates (1872: 99).

*Sitophagus castaneus* Reitter, 1877: 9. Synonymy: Champion (1886: 161).

***Sitophagus laticollis* Kulzer, 1961** MEX (OA)

*Sitophagus laticollis* Kulzer, 1961b: 540.

***Sitophagus uniformis* Doyen, 1990** MEX (GE JA OA PU)

*Sitophagus uniformis* Doyen, 1990: 250.

**Subtribe Diaperina Latreille, 1802**

Diaperialae Latreille, 1802: 161. Type genus: *Diaperis* Geoffroy, 1762.

Pentaphyllaires Mulsant, 1854: 196. Type genus: *Pentaphyllus* Dejean, 1821.

Platydeminae Reitter, 1917: 61. Type genus: *Platydema* Laporte and Brullé, 1831.

**Genus *Ceropria* Laporte and Brullé, 1831** [F]

*Ceropria* Laporte and Brullé, 1831: 332, 396. Type species: *Helops indutus* Wiedemann, 1819, subsequent designation (Gebien 1940: 422).

*Epilampus* Dejean, 1834: 198. Unnecessary replacement name for *Ceropria* Laporte and Brullé, 1831 (see Bousquet and Bouchard 2013: 52).

***Ceropria induta* (Wiedemann, 1819)** USA (FL) – Adventive

*Helops indutus* Wiedemann, 1819: 164.

**Genus *Cosmonota* Blanchard, 1842** [F]

*Cosmonota* Blanchard, 1842: pl. 14. Type species: *Cosmonota angustata* Blanchard, 1842, subsequent designation (Gebien 1940: 417).

***Cosmonota nigripes* Chevrolat, 1877** MEX (VE) GUA BEL NIC

*Cosmonota nigripes* Chevrolat, 1877b: 173.

***Cosmonota pubescens* Champion, 1886** NIC CRI PAN

*Cosmonota pubescens* Champion, 1886: 210.

***Cosmonota silphoides* (Laporte and Brullé, 1831)** MEX (JA VE) GUA BEL NIC PAN / SA

*Platydema silphoides* Laporte and Brullé, 1831: 369.

*Platydema agile* Chevrolat, 1877b: 178. Synonymy: Gebien (1940: 413).

**Genus *Diaperis* Geoffroy, 1762** [F]

*Diaperis* Geoffroy, 1762: 337. Type species: *Chrysomela boleti* Linnaeus, 1758, subsequent designation (Latreille 1810: 429).

*Allophasia* Pascoe, 1871: 351. Type species: *Allophasia fryi* Pascoe, 1871, monotypy. Synonymy: Triplehorn and Brendell (1985: 14).

***Diaperis californica* Blaisdell, 1929** USA (CA OR)

*Diaperis californica* Blaisdell, 1929c: 60.

**Figure 37.**
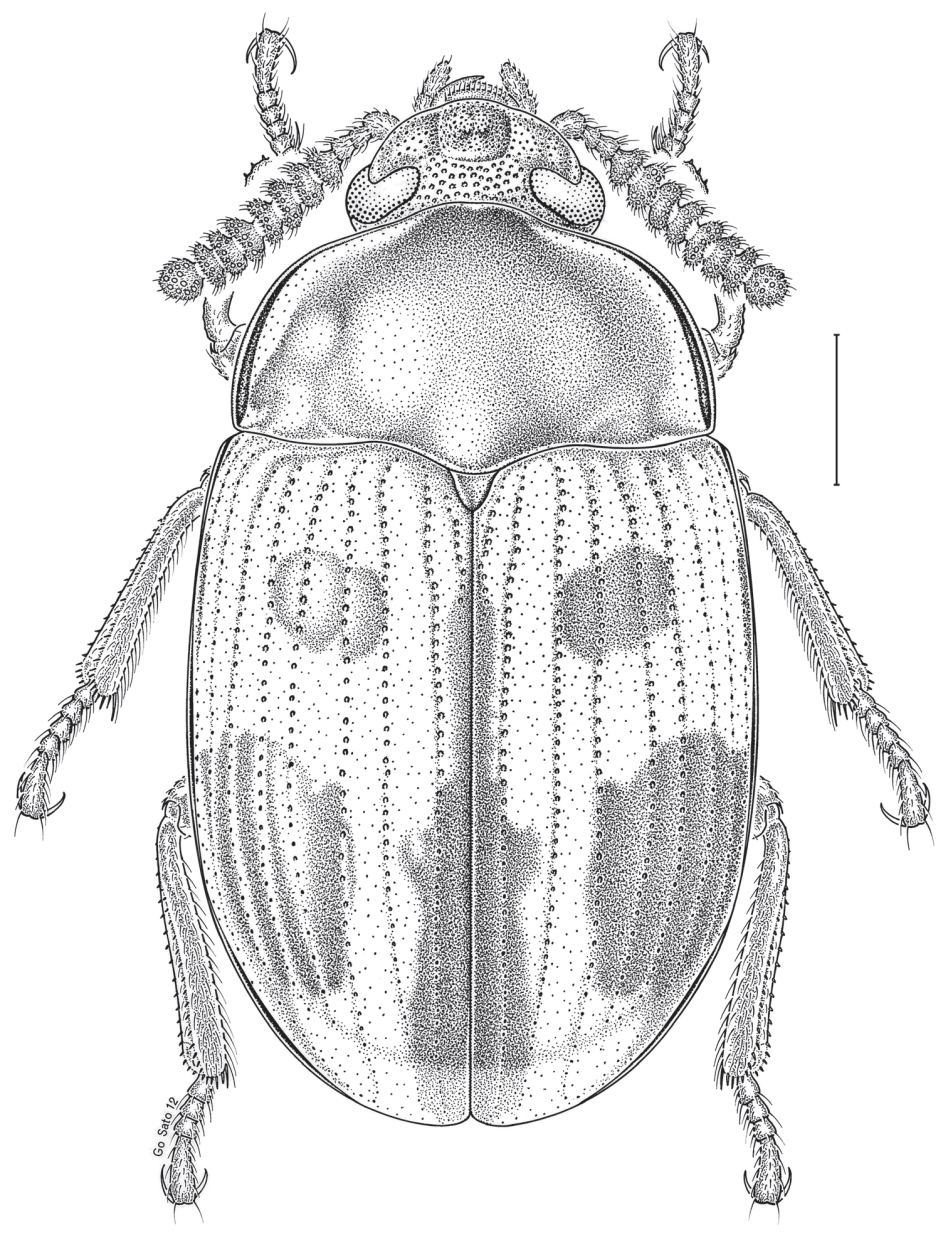
*Diaperis maculata* Olivier, 1791. Scale bar = 1 mm.

***Diaperis maculata* Olivier, 1791** [Fig. 37] CAN (MB NB NS ON PE QC SK) USA (AL AR CT DC DE FL GA IA IL IN KS KY LA MA MD ME MI MN MO MS NC ND NE NH NJ NY OH OK PA RI SC SD TN TX VA WI WV WY) MEX (VE) GUA CRI PAN / BAH CUB CAY JAM DOM PRI LAN

*Diaperis maculata* Olivier, 1791: 273.

*Diaperis hydactina* Fabricius, 1798: 178^68^. Synonymy: Latreille (1804: 307, as *D. hydni*).

*Diaperis suturalis* Chevrolat, 1877a: 170. Synonymy: Champion (1886: 174).

Diaperis maculata var. floridana Blatchley, 1912: 332. Synonymy: Peck and Thomas (1998: 106).

***Diaperis nigronotata* Pic, 1926** USA (AL AR FL GA IA IN KS LA MD MN MO MS OH OK PA SC TN TX WI WV)

Diaperis rufipes var. nigronotata Pic, 1926: 22.

Diaperis rufipes var. bicoloriceps Pic, 1926: 22. Synonymy: Triplehorn (1965: 373).

***Diaperis rufipes* Horn, 1870** USA (AZ CA NM) MEX (BS)

*Diaperis rufipes* Horn, 1870: 379.

**Genus *Lelegeis* Champion, 1886** [M]

*Lelegeis* Champion, 1886: 209. Type species: *Lelegeis aeneipennis* Champion, 1886, monotypy.

***Lelegeis aeneipennis* Champion, 1886** MEX (VE)

*Lelegeis aeneipennis* Champion, 1886: 210.

***Lelegeis apicalis* (Laporte and Brullé, 1831)** CUB PRI

*Platydema apicalis* Laporte and Brullé, 1831: 359.

***Lelegeis hispaniolae* Triplehorn, 1962** HAI DOM

*Lelegeis hispaniolae* Triplehorn, 1962: 503.

***Lelegeis nigrifrons* (Chevrolat, 1878)** MEX (VE) PAN / SA

*Platydema nigrifrons* Chevrolat, 1878g: cxlviii.

**Genus *Liodema* Horn, 1870** [F]

*Liodema* Horn, 1870: 385. Type species: *Platydema laevis* Haldeman, 1848, monotypy.

***Liodema connexa* Bates, 1873** MEX (VE) GUA PAN / SA

*Liodema connexum* Bates, 1873c: 236.

*Platydema nigro-fasciatum* Chevrolat, 1878b: 215. Synonymy: Champion (1886: 207).

***Liodema explanata* Triplehorn, 1998** CRI PAN

*Liodema explanatum* Triplehorn, 1998: 325.

***Liodema laevis* (Haldeman, 1848)** USA (FL GA MS NC SC TX) MEX (QU TA VE) GUA CRI PAN

*Platydema laevis* Haldeman, 1848: 101.

***Liodema maculata* (Fabricius, 1801)** MEX (NA PU SL TA VE YU) GUA SAL HON NIC CRI PAN / SA

*Mycetophagus maculatus* Fabricius, 1801b: 566.

*Platydema 4-notata* Laporte and Brullé, 1831: 380. Synonymy (in doubt with *L. kirschi* Bates): Champion (1886: 205).

*Liodema obydense* Bates, 1873c: 235. Synonymy: Triplehorn (1998: 327).

*Liodema kirschi* Bates, 1873c: 235. Synonymy: Gebien (1940: 417).

*Liodema fulvum* Bates, 1873c: 236. Synonymy (in doubt with *L. kirschi* Bates): Champion (1886: 205).

*Liodema horni* Bates, 1873c: 236. Synonymy: Triplehorn (1998: 328).

*Scaphidema tergocinctum* Chevrolat, 1877b: 178. Synonymy (with *L. kirshi* Bates): Chevrolat (1878d: 243).

*Scaphidema proximum* Chevrolat, 1877b: 178. Synonymy (with *L. obydense* Bates): Chevrolat (1878d: 243).

*Liodema inscriptum* Chevrolat, 1878b: 222. Synonymy (with *L. kirshi* Bates): Champion (1886: 205).

***Liodema serricornis* Bates, 1873** MEX (TA VE) GUA SAL HON NIC CRI PAN / DOM / SA

*Liodema serricorne* Bates, 1873c: 236.

*Platydema cruciatum* Chevrolat, 1877c: 182. Synonymy: Triplehorn (1998: 328).

*Platydema hamatiferum* Chevrolat, 1878f: c. Synonymy: Triplehorn (1998: 328).

*Platydema ramulosum* Chevrolat, 1878f: c. Synonymy: Triplehorn (1998: 328).

*Liodema zimmermani* Champion, 1886: 206. Synonymy: Triplehorn (1998: 328).

*Liodema flavo-variegatum* Champion, 1886: 208. Synonymy: Triplehorn (1998: 328).

**Genus *Neomida* Latreille, 1829** [F]

*Neomida* Latreille, 1829a: 29. Type species: *Ips haemorrhoidalis* Fabricius, 1787, monotypy.

*Oplocephala* Laporte and Brullé, 1831: 338. Type species: *Ips haemorrhoidalis* Fabricius, 1787, subsequent designation (Motschulsky 1845a: 80). Synonymy: Dejean (1834: 197).

*Arrhenoplita* Kirby, 1837: 235. Type species: *Ips haemorrhoidalis* Fabricius, 1787, original designation. Synonymy: Duponchel and Chevrolat (1841: 157).

*Hoplocephala* Agassiz, 1846: 185. Unjustified emendation of *Oplocephala* Laporte and Brullé, 1831, not in prevailing usage.

*Evoplus* LeConte, 1866b: 128. Type species: *Evoplus ferruginea* LeConte, 1866, monotypy. Synonymy (with *Arrhenoplita* Kirby): Champion (1886: 175).

***Neomida acera* Triplehorn, 1994** CRI PAN

*Neomida acera* Triplehorn, 1994a: 426.

***Neomida aeneipennis* Triplehorn, 1965** MEX (CI HI JA OA PU QR QU SL TA VE YU) GUA BEL SAL HON NIC CRI

*Neomida aeneipennis* Triplehorn, 1965: 382.

***Neomida armata* (Laporte and Brullé, 1831)** CUB / SA

*Oplocephala armata* Laporte and Brullé, 1831: 345.

***Neomida bicornis* (Fabricius, 1777)** CAN (MB NB NS ON PE QC) USA (AL AR CT DC DE FL GA IA IL IN KS KY LA MA MD ME MI MN MO MS NC NE NH NJ NY OH OK PA RI SC SD TN TX VA WI WV) / BAH BER CUB CAY JAM

*Hispa bicornis* Fabricius, 1777: 215.

*Hispa cornigera* Fabricius, 1781: 82. Synonymy:Triplehorn (1965: 377).

*Diaperis viridipennis* Fabricius, 1801b: 586. Synonymy:Triplehorn (1965: 377).

*Blaps metallica* Palisot de Beauvois, 1817: 140 [junior primary homonym of *Blaps metallica* Fabricius, 1801]. Synonymy: Horn (1885a: 88).

*Oplocephala virescens* Laporte and Brullé, 1831: 341. Synonymy: Melsheimer (1853: 137).

*Oplocephala capra* Laporte and Brullé, 1831: 345. Synonymy (in doubt): Triplehorn (2006: 332).

*Oplocephala gracilis* Motschulsky, 1873: 467. Synonymy: Horn (1874b: 98).

***Neomida castanea* (Bates, 1873)** MEX (VE) NIC CRI PAN / SA

*Hoplocephala castanea* Bates, 1873b: 204.

*Hoplocephala oblonga* Chevrolat, 1878f: xcvii. Synonymy: Triplehorn (2006: 325).

***Neomida cioides* (Champion, 1886)** MEX (CI QU VE) CRI PAN / DOM LAN / SA

*Arrhenoplita cioides* Champion, 1886: 180.

***Neomida clavicornis* (Champion, 1886)** MEX (VE) CI

*Arrhenoplita clavicornis* Champion, 1886: 176.

***Neomida deltocera* Triplehorn, 1994** CRI PAN LAN / SA

*Neomida deltocera* Triplehorn, 1994a: 423.

***Neomida distans* (Champion, 1886)** MEX (VE) CRI PAN / SA

*Arrhenoplita distans* Champion, 1886: 178.

***Neomida divergicornis* Triplehorn, 1994** MEX (CI VE)

*Neomida divergicornis* Triplehorn, 1994a: 420.

***Neomida dolichocera* Triplehorn, 1994** CRI

*Neomida dolichocera* Triplehorn, 1994a: 417.

***Neomida ferruginea* (LeConte, 1866)** USA (AL FL GA LA TX) MEX (CL HI NA PU SI SL TA TB VE) GUA BEL CRI PAN / CUB CAY JAM HAI DOM / SA

*Evoplus ferruginea* LeConte, 1866b: 128.

*Oplocephala castanea* Motschulsky, 1873: 467. Synonymy (in doubt): Horn (1874b: 98)

***Neomida heterocera* Triplehorn, 1994** CRI PAN

*Neomida heterocera* Triplehorn, 1994a: 421.

***Neomida hoffmanseggii* (Laporte and Brullé, 1831)** MEX (CL) CRI PAN / SA

*Oplocephala hoffmanseggii* Laporte and Brullé, 1831: 346.

***Neomida inermis* (Champion, 1886)** GUA CRI PAN / LAN (Guadeloupe)

*Arrhenoplita inermis* Champion, 1886: 179.

***Neomida lateralis* (Bates, 1873)** MEX (PU VE) CRI PAN / SA

*Hoplocephala lateralis* Bates, 1873b: 204.

*Hoplocephala dytiscoïdes* Chevrolat, 1877a: 170. Synonymy: Triplehorn (2006: 319).

*Hoplocephala lutea* Chevrolat, 1878f: xcvii. Synonymy: Triplehorn (2006: 333).

***Neomida lawrencei* Triplehorn, 1994** MEX (HI OA) BEL CRI PAN

*Neomida lawrencei* Triplehorn, 1994a: 419.

***Neomida lecontei* (Bates, 1873)** MEX (CI VE) BEL HON PAN / JAM DOM PRI LAN / SA

*Evoplus lecontii* Bates, 1873c: 233.

*Uloma guadeloupensis* Marcuzzi, 1971: 110. Synonymy: Soldati and Touroult (2014: 102), confirmed by Ivie and Hart (2017a: 116).

***Neomida nigricornis* (Champion, 1886)** GUA BEL CRI PAN

*Arrhenoplita nigricornis* Champion, 1886: 179.

***Neomida obsoleta* (Champion, 1886)** MEX (OA VE) BEL CRI PAN

*Arrhenoplita obsoleta* Champion, 1886: 178.

***Neomida occidentalis* (Champion, 1893)** MEX (BS GE JA PU SI) HON CRI

*Arrhenoplita occidentalis* Champion, 1893a: 537.

*Neomida myllocnema* Triplehorn, 1965: 386. Synonymy: Triplehorn (2006: 315).

***Neomida paurocera* Triplehorn, 1994** SAL HON

*Neomida paurocera* Triplehorn, 1994a: 424.

***Neomida pentaphylloides* (Champion, 1886)** GUA

*Arrhenoplita pentaphylloides* Champion, 1886: 180.

***Neomida picea* (Laporte and Brullé, 1831)** GUA BEL CRI PAN / SA

*Oplocephala picea* Laporte and Brullé, 1831: 344.

*Hoplocephala testaceipes* Pic, 1926: 28. Synonymy: Triplehorn (2006: 313).

***Neomida pogonocera* Triplehorn, 1994** PAN / SA

*Neomida pogonocera* Triplehorn, 1994a: 422.

***Neomida punctatissima* (Champion, 1893)** MEX (GE JA)

*Arrhenoplita punctatissima* Champion, 1893a: 537.

***Neomida suilla* (Champion, 1896)** LAN / SA

*Arrhenoplita suilla* Champion, 1896: 11.

***Neomida telecera* Triplehorn, 2006** CRI

*Neomida telecera* Triplehorn, 2006: 328.

**Genus *Paniasis* Champion, 1886** [M]

*Paniasis* Champion, 1886: 208. Type species: *Paniasis dilatipes* Champion, 1886, monotypy.

*Pseudapsida* Kulzer, 1961a: 219. Type species: *Pseudapsida brasiliensis* Kulzer, 1961, original designation. Synonymy: Ferrer and Ødegaard (2005: 637).

***Paniasis dilatipes* Champion, 1886** MEX (VE) / SA

*Paniasis dilatipes* Champion, 1886: 209.

***Paniasis kulzeri* Ferrer and Ødegaard, 2005** PAN

*Paniasis kulzeri* Ferrer and Ødegaard, 2005: 637.

**Genus *Pentaphyllus* Dejean, 1821** [M]

*Pentaphyllus* Dejean, 1821: 68. Type species: *Mycetophagus testaceus* Hellwig, 1792, monotypy.

*Iphicorynus* Jacquelin du Val, 1861: 299. Type species: *Pentaphyllus melanophthalmus* Mulsant, 1854 (= *Nitidula chrysomeloides* Rossi, 1792), monotypy. Synonymy: Gemminger [in Gemminger and Harold] (1870: 1956).

***Pentaphyllus californicus* Horn, 1870** USA (CA)

*Pentaphyllus californicus* Horn, 1870: 387.

***Pentaphyllus pallidus* LeConte, 1866** CAN (ON QC) USA (CT GA IL IN KY MD MI NJ NY OH PA SC WI)

*Pentaphyllus pallidus* LeConte, 1866b: 126.

*Pentaphyllus americanus* Motschulsky, 1873: 482. Synonymy: Horn (1874b: 98).

***Pentaphyllus testaceus* (Hellwig, 1792)** CAN (ON) – Adventive

*Mycetophagus testaceus* Hellwig, 1792: 400.

**Genus *Platydema* Laporte and Brullé, 1831** [F]

*Platydema* Laporte and Brullé, 1831: 350. Type species: *Diaperis violacea* Fabricius, 1790, subsequent designation (Westwood 1838: 32).

*Typhobia* Pascoe, 1869: 279. Type species: *Typhobia fuliginea* Pascoe, 1869, monotypy. Synonymy: Champion (1886: 181).

*Histeropsis* Chevrolat, 1878b: 221. Type species: *Platydema americanum* Laporte and Brullé, 1831, subsequent designation (Löbl et al. 2008b: 42). Synonymy: Champion (1886: 181).

***Platydema americana* Laporte and Brullé, 1831** CAN (AB BC MB NB NS ON QC SK) USA (AZ CA CO CT IA ID IL KS MD ME MI MN MO MT NC NE NH NJ NM NV NY OH OR PA SC SD TX VA WA WI WY)

*Platydema americana* Laporte and Brullé, 1831: 358.

*Platydema polita* Laporte and Brullé, 1831: 361. Synonymy: Horn (1870: 384).

***Platydema angulata* Chevrolat, 1877** MEX

*Platydema angulatum* Chevrolat, 1877d: 186.

***Platydema antennata* Laporte and Brullé, 1831** BAH CUB CAY HAI

*Platydema antennata* Laporte and Brullé, 1831: 366.

***Platydema apicenotata* Champion, 1896** LAN

*Platydema apicenotatum* Champion, 1896: 13.

***Platydema basicornis* Chevrolat, 1877** CUB

*Platydema basicorne* Chevrolat, 1877b: 178.

***Platydema bimaculata* Champion, 1886** MEX (VE) GUA BEL NIC CRI PAN / SA

*Platydema bimaculatum* Champion, 1886: 193.

***Platydema biplagiata* Champion, 1886** MEX (VE) GUA NIC PAN

*Platydema biplagiatum* Champion, 1886: 201.

***Platydema bisignata* Chevrolat, 1877** MEX GUA / SA

*Platydema bisignatum* Chevrolat, 1877c: 181.

***Platydema brevis* Champion, 1886** MEX (VE) GUA PAN

*Platydema breve* Champion, 1886: 200.

***Platydema concolor* Champion, 1893** NIC PAN

*Platydema unicolor* Champion, 1886: 203 [junior primary homonym of *Platydema unicolor* Chevrolat, 1878].

*Platydema concolor* Champion, 1893a: 539. Replacement name for *Platydema unicolor* Champion, 1886.

***Platydema cordovensis* Champion, 1886** MEX (VE) GUA

*Platydema cordovense* Champion, 1886: 203.

***Platydema cyanea* Laporte and Brullé, 1831** “Amérique septentrionale”^69^

*Platydema cyanea* Laporte and Brullé, 1831: 392.

***Platydema cyanescens* Laporte and Brullé, 1831** USA (AL FL GA IN LA MS NC OH SC TN TX)

*Platydema cyanescens* Laporte and Brullé, 1831: 356.

***Platydema dichrocera* Triplehorn, 1962** CUB

*Platydema dichrocerum* Triplehorn, 1962: 502.

***Platydema dimidiata* Chevrolat, 1878** MEX (VE) GUA BEL HON

*Platydema dimidiatum* Chevrolat, 1878a: 194.

***Platydema diophthalma* Laporte and Brullé, 1831** MEX (DU VE YU) GUA BEL HON NIC CRI PAN / CUB

*Platydema diophthalma* Laporte and Brullé, 1831: 383.

*Platydema luna* Chevrolat, 1877d: 186. Synonymy: Champion (1886: 193).

***Platydema elegans* Chevrolat, 1878** MEX (PU VE)

*Platydema elegans* Chevrolat, 1878a: 195.

***Platydema elliptica* (Fabricius, 1798)** CAN (ON) USA (AL AR CO CT DC DE FL GA IA IL IN KS KY LA MD MI MO MS NC NJ NY OH OK PA SC TN TX UT VA WI)

*Tenebrio ellipticus* Fabricius, 1798: 49.

***Platydema erotyloides* Chevrolat, 1878** PAN / SA **New North American record**

*Platydema ornatum* Chevrolat, 1878b: 209 [junior primary homonym of *Platydema ornatum* Chevrolat, 1877].

*Platydema erotyloides* Chevrolat, 1878d: 243. Replacement name for *Platydema ornatum* Chevrolat, 1878.

***Platydema erythrocera* Laporte and Brullé, 1831** USA (AL AR DC FL GA IL IN KS LA MD MO MS NC NY OH OK SC TN TX VA WV) MEX (CI HI) BEL / BAH

*Platydema erythrocera* Laporte and Brullé, 1831: 355.

*Neomida flavicornis* Motschulsky, 1873: 479. Synonymy: Horn (1874b: 98).

*Platydema hondurense* Champion, 1886: 186. Synonymy: Triplehorn (1994b: 247).

***Platydema excavata* (Say, 1824)** CAN (ON QC) USA (AL AR AZ CT DC DE FL GA IA IL IN KS KY LA MA MD MI MN MO MS NC NE NH NJ NM NY OH OK PA RI SC SD TN TX VA WI WV) MEX (JA PU VE YU) GUA BEL HON NIC CRI PAN / BAH CUB CAY HAI DOM JAM PRI LAN / SA

*Diaperis excavata* Say, 1824a: 267.

*Platydema tuberculata* Laporte and Brullé, 1831: 352. Synonymy: Champion (1886: 184).

*Platydema nigritum* Motschulsky, 1873: 470. Synonymy: Horn (1874b: 98).

*Platydema fraternum* Chevrolat, 1878b: 210. Synonymy: Champion (1886: 184).

*Platydema parvulum* Casey, 1884: 50. Synonymy: Casey (1885: 195).

***Platydema fasciatocollis* Chevrolat, 1878** MEX

*Platydema fasciato-colle* Chevrolat, 1878a: 194.

***Platydema fasciata* (Fabricius, 1801)** MEX (PU VE) GUA BEL / SA

*Mycetophagus fasciatus* Fabricius, 1801b: 567.

***Platydema ferruginea* Chevrolat, 1877** MEX (CI VE YU) GUA PAN

*Platydema ferrugineum* Chevrolat, 1877d: 186.

*Platydema bi-impressum* Chevrolat, 1878b: 214. Synonymy: Champion (1886: 190).

***Platydema flavipes* (Fabricius, 1801)** USA (AL AR DC DE FL GA ON KS KY LA MA MD MS NC ND NH NJ NY OH PA SC TN TX VA)

*Mycetophagus flavipes* Fabricius, 1801b: 567.

*Platydema basalis* Haldeman, 1848: 101. Synonymy: Horn (1870: 382).

***Platydema flexuosa* Chevrolat, 1877** CUB

*Platydema flexuosum* Chevrolat, 1877b: 178.

***Platydema fuliginosa* Laporte and Brullé, 1831** MEX

*Platydema fuliginosa* Laporte and Brullé, 1831: 374.

***Platydema guatemalensis* Champion, 1886** MEX (CI VE) GUA SAL CRI PAN /LAN / SA

*Platydema guatemalense* Champion, 1886: 197.

***Platydema hoegei* Champion, 1886** MEX (CI GE PU VE)

*Platydema högei* Champion, 1886: 195.

***Platydema immaculata* Champion, 1886** PAN

*Platydema immaculatum* Champion, 1886: 192.

***Platydema inquilina* Linell, 1899** USA (AZ)

*Platydema inquilinum* Linell, 1899: 183.

***Platydema laevipes* Haldeman, 1848** USA (AL AR DC FL GA IA IN KS LA MA MD MO MS NC NJ NY OH PA SC TX VA WI)

*Platydema laevipes* Haldeman, 1848: 101.

*Platydema crenatum* LeConte, 1878a: 422. Synonymy: Triplehorn (1965: 407).

***Platydema lucens* Champion, 1886** MEX (VE)

*Platydema lucens* Champion, 1886: 202.

***Platydema maculipennis* Champion, 1886** MEX (VE) GUA

*Platydema maculipenne* Champion, 1886: 201.

***Platydema melancholica* Champion, 1886** GUA

*Platydema melancholicum* Champion, 1886: 190.

***Platydema mexicana* Champion, 1886** USA (AZ NM) MEX (CH DU VE)

*Platydema mexicanum* Champion, 1886: 187.

***Platydema micans* Zimmerman, 1870** USA (AL AR DC FL GA IN KS LA MD MS NC SC TN TX VA) MEX (JA) / BAH TUR CUB CAY JAM HAI / SA

*Platydema micans* Zimmerman [in Horn], 1870: 383.

***Platydema monilicornis* Chevrolat, 1877** MEX

*Platydema monilicorne* Chevrolat, 1877d: 186.

***Platydema neglecta* Triplehorn, 1965** CAN (BC) USA (CA ID MT NV OR UT WA)

*Platydema neglectum* Triplehorn, 1965: 404.

***Platydema nicaraguensis* Champion, 1886** NIC

*Platydema nicaraguense* Champion, 1886: 192.

***Platydema nigrata* (Motschulsky, 1873)** USA (AL AZ CA FL GA IN KS LA MS NC NM SC TX) MEX (MO SI VE YU) GUA BEL HON NIC CRI / BAH CUB

*Neomida nigrata* Motschulsky, 1873: 478.

*Neomida texana* Motschulsky, 1873: 478. Synonymy (in doubt with *P. janus* sensu Horn, 1870 = *P. nigrita*): Horn (1874b: 98).

***Platydema nigromaculata* Champion, 1886** BEL NIC PAN

*Platydema nigromaculatum* Champion, 1886: 199.

***Platydema nitida* (Chevrolat, 1877)** MEX (YU)

*Scaphidema nitidum* Chevrolat, 1877a: 170.

***Platydema oculata* Champion, 1886** MEX (VE)

*Platydema oculatum* Champion, 1886: 191.

***Platydema oregonensis* LeConte, 1857** CAN (BC) USA (CA ID OR WA)

*Platydema oregonense* LeConte, 1857: 51.

***Platydema ornata* Chevrolat, 1877** MEX (VE)

*Platydema ornatum* Chevrolat, 1877d: 186.

***Platydema panamensis* Champion, 1886** PAN

*Platydema panamense* Champion, 1886: 198.

***Platydema picicornis* (Fabricius, 1792)** CUB HAI PRI / SA

*Mycetophagus picicornis* Fabricius, 1792b: 498.

***Platydema picilabrum* Melsheimer, 1846** USA (AL AR FL GA IL IN KS KY LA MA MD MI MO MS NC NJ NY OH PA SC TN TX VA WI WV)

*Platydema picilabrum* Melsheimer, 1846: 62.

***Platydema pilifera* Champion, 1896** LAN

*Platydema piliferum* Champion, 1896: 12.

***Platydema pretiosa* Champion, 1886** BEL

*Platydema pretiosum* Champion, 1886: 197.

***Platydema punctatostriata* Chevrolat, 1877** CUB

*Platydema punctatostriatum* Chevrolat, 1877b: 178.

***Platydema quadrimaculata* Laporte and Brullé, 1831** USA (PA)^70^

*Platydema4-maculata* Laporte and Brullé, 1831: 383.

***Platydema quindecimmaculata* (Chevrolat, 1878)** GUA NIC PAN / SA

*Platydema 15-maculatum* Chevrolat, 1878g: cxlix.

***Platydema rotundata* Chevrolat, 1877** MEX (GE JA MO PU VE YU) GUA CRI

*Platydema rotundatum* Chevrolat, 1877d: 186.

***Platydema ruficollis* Laporte and Brullé, 1831** USA (AR FL GA IA IL KY MD MS NC NJ OK SC TX VA WI)

*Platydema ruficollis* Laporte and Brullé, 1831: 375.

*Neomida sanguinicollis* Melsheimer, 1846: 61. Synonymy: Haldeman (1848: 102).

***Platydema ruficornis* (Sturm, 1826)** CAN (ON QC) USA (AL AR CO CT DC DE FL GA IA IL IN KS KY LA MA MD MI MN MO MS NC NE NJ NY OH OK PA SC TN TX VA WI WV) / BAH

*Diaperis ruficornis* Sturm, 1826: 69.

*Platydema rufiventris* Laporte and Brullé, 1831: 378. Synonymy: Melsheimer (1853: 138).

*Platydema pallens* Laporte and Brullé, 1831: 377. Synonymy (in doubt): Horn (1870: 382)^71^.

*Neomida rufa* Melsheimer, 1846: 62. Synonymy: Horn (1870: 382).

*Platydema analis* Haldeman, 1848: 101. Synonymy: Horn (1870: 382).

*Platydema opaculum* Casey, 1884: 51. Synonymy (with P. ruficorne var. anale Haldeman): Horn (1885b: 111).

***Platydema rugiceps* Champion, 1886** MEX (VE) GUA NIC PAN

*Platydema rugiceps* Champion, 1886: 191.

***Platydema sexmaculata* Chevrolat, 1878** MEX

*Platydema sexmaculatum* Chevrolat, 1878a: 194.

***Platydema sexnotata* Chevrolat, 1878** MEX (JA VE) NIC CRI

*Platydema sexnotatum* Chevrolat, 1878a: 194.

***Platydema sobrina* Chevrolat, 1877** MEX (VE) GUA BEL NIC CRI PAN / SA

*Neomida discolor* Motschulsky, 1873: 477 [*nomen dubium*].

*Platydema sobrinum* Chevrolat, 1877d: 186. Synonymy (in doubt): Champion (1886: 189).

***Platydema subcostata* Laporte and Brullé, 1831** CAN (ON QC) USA (AL AR CT DC DE FL GA IA IL IN KS KY LA MA MD MI MN MO MS NC NH NJ NY OH PA RI SC TN TX VA WI WV)

*Platydema subcostata* Laporte and Brullé, 1831: 362.

*Platydema clypeatus* Haldeman, 1848: 102. Synonymy: Horn (1870: 384).

*Platydema oblongulum* Motschulsky, 1873: 470. Synonymy: Horn (1874b: 98).

***Platydema subquadrata* (Motschulsky, 1873)** ^72^ “Amérique centrale”

*Neomida subquadrata* Motschulsky, 1873: 477.

*Platydema pernigrum* Casey, 1884: 49. Synonymy: Champion (1886: 188).

***Platydema submaculata* (Chevrolat, 1878)** MEX (PU VE YU) BEL

*Platydema submaculatum* Chevrolat, 1878f: xcix.

***Platydema teleops* Triplehorn, 1965** CAN (NB NS ON QC) USA (CT DC IA IL IN KS MA MD ME MI MN MO NC NE NH NJ NY OH OK SC TN TX VA WI WV)

*Platydema teleops* Triplehorn, 1965: 399.

***Platydema tibialis* (Chevrolat, 1878)** NIC PAN / SA

*Platydema tibiale* Chevrolat, 1878g: cxlviii.

***Platydema transversa* Laporte and Brullé, 1831** MEX (OA VE) GUA BEL PAN / SA

*Platydema transversa* Laporte and Brullé, 1831: 381.

***Platydema tricolor* Champion, 1886** GUA

*Platydema tricolor* Champion, 1886: 200.

***Platydema undata* Chevrolat, 1878** MEX (CI PU SL VE) GUA BEL SAL NIC CRI PAN / SA

*Neomida picta* Motschulsky, 1873: 480 [junior secondary homonym of *Platydema pictum* (Ménétriés, 1832)].

*Platydema undatum* Chevrolat, 1878a: 194. Synonymy: Champion (1886: 185).

*Platydema rodriguezi* Champion, 1886: 185. Synonymy: Triplehorn (1994b: 249).

***Platydema venusta* Champion, 1886** NIC PAN

*Platydema venustum* Champion, 1886: 204.

***Platydema ventralis* Chevrolat, 1877** MEX

*Platydema ventrale* Chevrolat, 1877d: 186.

***Platydema versicolor* Chevrolat, 1878** MEX (PU VE)

*Platydema versicolor* Chevrolat, 1878a: 195.

***Platydema viriditincta* Champion, 1886** MEX (OA)

*Platydema viriditinctum* Champion, 1886: 186.

***Platydema wandae* Triplehorn, 1965** USA (AZ NM)

*Platydema wandae* Triplehorn, 1965: 427.

***Platydema woldai* Triplehorn and Philips, 1998** USA_i_ (FL) MEX GUA SAL HON PAN

*Platydema woldai* Triplehorn and Philips [in Philips et al.], 1998: 291.

**Genus *Stenoscapha* Bates, 1873** [F]

*Stenoscapha* Bates, 1873c: 237. Type species: *Stenoscapha tibialis* Bates, 1873, monotypy.

***Stenoscapha jalapensis* Champion, 1886** MEX (VE)

*Stenoscapha jalapensis* Champion, 1886: 208.

**Genus *Ulomoides* Blackburn, 1888** [M]

*Ulomoides* Blackburn, 1888: 274. Type species: *Ulomoides humeralis* Blackburn, 1888, monotypy.

*Palembus* Casey, 1891: 65. Type species: *Palembus ocularis* Casey, 1891, monotypy. Synonymy: Doyen et al. (1990: 237).

*Martianus* Fairmaire, 1893: 540. Type species: *Martianus castaneus* Fairmaire, 1893 (= *Palembus ocularis* Casey, 1891), original designation. Synonymy (with *Palembus* Casey): Halstead (1974: 241).

*Tenebriomimus* Kolbe, 1901: 342. Type species: *Tenebriomimus adansoniarum* Kolbe, 1901 (= *Palembus ocularis* Casey, 1891), monotypy. Synonymy (with *Martianus* Fairmaire): Gebien (1922: 268).

***Ulomoides ocularis* (Casey, 1891)** USA (FL) / BAH CUB CAY JAM DOM PRI LAN – Adventive

*Palembus ocularis* Casey, 1891: 65.

*Martianus castaneus* Fairmaire, 1893: 541. Synonymy: Halstead (1974: 242).

*Tenebriomimus adansoniarum* Kolbe, 1901: 342. Synonymy (with *M. castaneus* Fairmaire): Gebien (1922: 268).

**Tribe Gnathidiini Gebien, 1921**

Gnathidiini Gebien, 1921: 41. Type genus: *Gnathidium* Gebien, 1921.

**Subtribe Anopidiina Jeannel and Paulian, 1945**

Anopidiini Jeannel and Paulian, 1945: 62. Type genus: *Anopidium* Jeannel and Paulian, 1945.

**Genus *Caecophloeus* Dajoz, 1972** [M]

*Caecophloeus* Dajoz, 1972: 278. Type species: *Caecophloeus franzi* Dajoz, 1972, original designation.

***Caecophloeus darlingtoni* Dajoz, 1975** HAI

*Caecophloeus darlingtoni* Dajoz, 1975: 115.

***Caecophloeus distinctus* Dajoz, 1975** MEX (CI)

*Caecophloeus distinctus* Dajoz, 1975: 117.

***Caecophloeus franzi* Dajoz, 1972** JAM

*Caecophloeus franzi* Dajoz, 1972: 280.

***Caecophloeus ineditus* Dajoz, 1975** MEX (CI)

*Caecophloeus ineditus* Dajoz, 1975: 119.

***Caecophloeus pubescens* Dajoz, 1975** PAN

*Caecophloeus pubescens* Dajoz, 1975: 117.

**Genus *Cryptozoon* Schaufuss, 1882** [N]

*Cryptozoon* Schaufuss, 1882: 47. Type species: *Cryptozoon civile* Schaufuss, 1882, **present designation**.

***Cryptozoon civile* Schaufuss, 1882** PRI

*Cryptozoon civile* Schaufuss, 1882: 47.

***Cryptozoon nitidicolle* Schaufuss, 1882** PRI

*Cryptozoon nitidicolle* Schaufuss, 1882: 47.

**Genus *Menimopsis* Champion, 1896** [F]

*Menimopsis* Champion, 1896: 16. Type species: *Menimopsis excaecus* Champion, 1896, monotypy.

*Caecomenimopsis* Kaszab, 1970: 198. Type species: *Caecomenimopsis leleupi* Kaszab, 1970, original designation. Synonymy: Peck (1990: 370).

***Menimopsis excaeca* Champion, 1896** LAN

*Menimopsis excaecus* Champion, 1896: 17.

***Menimopsis franzi* Kaszab, 1977** JAM

*Menimopsis franzi* Kaszab, 1977a: 122.

***Menimopsis jamaicensis* (Dajoz, 1975)** JAM

*Caecomenimopsis jamaicensis* Dajoz, 1975: 121.^73^

***Menimopsis jamaicensis* Kaszab, 1977** JAM

*Menimopsis jamaicensis* Kaszab, 1977a: 121 [junior secondary homonym of *Menimopsis jamaicensis* (Dajoz, 1975)].^74^

**Genus *Neanopidium* Dajoz, 1975** [N]

*Neanopidium* Dajoz, 1975: 93. Type species: *Neanopidium mexicanum* Dajoz, 1975, original designation.

***Neanopidium affine* Dajoz, 1975** MEX (HI)

*Neanopidium affinis* Dajoz, 1975: 107.

***Neanopidium convexum* Dajoz, 1975** MEX (VE)

*Neanopidium convexum* Dajoz, 1975: 104.

***Neanopidium curticorne* Dajoz, 1975** MEX (CI)

*Neanopidium curticornis* Dajoz, 1975: 100.

***Neanopidium dubium* Dajoz, 1975** MEX (HI)

*Neanopidium dubium* Dajoz, 1975: 108.

***Neanopidium humerale* Dajoz, 1975** MEX (OA)

*Neanopidium humeralis* Dajoz, 1975: 105.

***Neanopidium lawrencei* Dajoz, 1975** MEX (VE)

*Neanopidium lawrencei* Dajoz, 1975: 103.

***Neanopidium mexicanum* Dajoz, 1975** MEX (OA)

*Neanopidium mexicanum* Dajoz, 1975: 96.

***Neanopidium minutum* Dajoz, 1975** MEX (VE)

*Neanopidium minutum* Dajoz, 1975: 105.

***Neanopidium newtoni* Dajoz, 1975** MEX (VE)

*Neanopidium newtoni* Dajoz, 1975: 102.

***Neanopidium pubescens* Dajoz, 1975** MEX (VE)

*Neanopidium pubescens* Dajoz, 1975: 102.

***Neanopidium punctatum* Dajoz, 1975** MEX (SL)

*Neanopidium punctatum* Dajoz, 1975: 106.

***Neanopidium simile* Dajoz, 1975** MEX (HI)

*Neanopidium similis* Dajoz, 1975: 98.

***Neanopidium testaceum* Dajoz, 1975** MEX (TA)

*Neanopidium testaceum* Dajoz, 1975: 103.

**Genus *Sphaerognathium* Dajoz, 1975** [N]

*Sphaerognathium* Dajoz, 1975: 112. Type species: *Sphaerognathium globosum* Dajoz, 1975, original designation.

***Sphaerognathium globosum* Dajoz, 1975** HAI

*Sphaerognathium globosum* Dajoz, 1975: 113.

**Genus *Tyrtaeus* Champion, 1913** [M]

*Tyrtaeus* Champion, 1913: 76. Type species: *Tyrtaeus rufus* Champion, 1913, original designation.

***Tyrtaeus cribripennis* Champion, 1913** PAN

*Tyrtaeus cribripennis* Champion, 1913: 77.

***Tyrtaeus dobsoni* Hinton, 1947** USA (FL) – Adventive

*Tyrtaeus dobsoni* Hinton, 1947a: 852.

***Tyrtaeus rufus* Champion, 1913** ^75^ USA (FL) MEX (VE) GUA CRI PAN / CUB CAY LAN / SA

*Tyrtaeus rufus* Champion, 1913: 77.

*Tyrtaeus guadalupensis* Dajoz, 1981: 227. Synonymy: Hopp and Ivie (2008: 429).

**Tribe Hypophlaeini Billberg, 1820**

Hypophlaeides Billberg, 1820a: 33. Type genus: *Hypophlaeus* Fabricius, 1790 (= *Corticeus* Piller and Mitterpacher, 1783).

Corticeini Boddy, 1965: 144. Type genus: *Corticeus* Piller and Mitterpacher, 1783.

**Genus *Corticeus* Piller and Mitterpacher, 1783** [M]

*Corticeus* Piller and Mitterpacher, 1783: 87. Type species: *Corticeus unicolor* Piller and Mitterpacher, 1783, monotypy.

*Hypophlaeus* Fabricius, 1790: 222. Type species: *Hypophlaeus castaneus* Fabricius, 1790 (= *Corticeus unicolor* Piller and Mitterpacher, 1783), subsequent designation (Curtis 1832: pl. 430). Synonymy: Crotch (1870: 47).

**Subgenus Corticeus Piller and Mitterpacher, 1783**

*Corticeus* Piller and Mitterpacher, 1783: 87. Type species: *Corticeus unicolor* Piller and Mitterpacher, 1783, monotypy.

***Corticeus coynei* Triplehorn, 1970** HON NIC

*Corticeus coynei* Triplehorn [in Triplehorn and Moser], 1970: 47.

***Corticeus crassicornis* Champion, 1886** GUA

*Corticeus crassicornis* Champion, 1886: 173.

***Corticeus longicornis* Champion, 1886** MEX

*Corticeus longicornis* Champion, 1886: 172.

***Corticeus mexicanus mexicanus* Reitter, 1878** MEX (VE) GUA NIC PAN / SA

*Corticeus mexicanus* Reitter, 1878: 191.

*Corticeus cylindricus* Reitter, 1878: 192 [junior primary homonym of *Corticeus cylindricus* Reitter, 1877]. Synonymy: Bremer and Triplehorn (1999: 57).

*Corticeus erratus* Reitter, 1894: 16. Replacement name for *Corticeus cylindricus* Reitter, 1878.

*Hypophloeus meridanus* Pic, 1914: 15. Synonymy: Bremer and Triplehorn (1999: 57).

***Corticeus opaculus* (LeConte, 1878)** USA (AZ CA) MEX GUA

*Hypophloeus opaculus* LeConte, 1878a: 423.

***Corticeus pallidipennis* Champion, 1886** MEX (VE) GUA

*Corticeus pallidipennis* Champion, 1886: 173.

***Corticeus parallelus* (Melsheimer, 1846)** CAN (MB ON QC) USA (AL AR DC DE FL GA IL IN KY LA MA MD ME MI MN MO MS NC ND NE NH NJ NY OH PA SC TN TX VA WI WV)

*Hypophloeus parallelus* Melsheimer, 1846: 63.

***Corticeus paulostriatus* (Pic, 1945)** USA (AR FL TN) / CUB HAI DOM PRI

*Hypophlaeus paulostriatus* Pic, 1945: 8.

*Corticeus tensicollis* Triplehorn, 1979: 46. Synonymy: Bremer and Triplehorn (1999: 58).

***Corticeus praetermissus* (Fall, 1926)** CAN (AB BC MB NB NF NS NT ON QC SK YT) USA (AK AZ CA CO ID MA ME MI MN NE NH NM NV NY OR PA SD TX UT WA WI WV WY) MEX

*Hypophloeus praetermissus* Fall, 1926: 199.

***Corticeus puncticollis* Champion, 1886** GUA

*Corticeus puncticollis* Champion, 1886: 172.

***Corticeus rosei* Triplehorn, 1970** USA (AZ) MEX (CH DU JA ME NL PU) HON

*Corticeus rosei* Triplehorn [in Triplehorn and Moser], 1970: 49.

***Corticeus rufipes* (Fabricius, 1801)** MEX (OA TB VE) GUA BEL NIC PAN / CUB PRI LAN / SA

*Hypophloeus rufipes* Fabricius, 1801b: 558.

***Corticeus sordidus* Champion, 1913** GUA

*Corticeus sordidus* Champion, 1913: 162.

***Corticeus strublei* Blaisdell, 1934** USA (AZ CA CO ID NM OR SD UT WA WY) [MEX]

*Corticeus strublei* Blaisdell, 1934a: 188.

***Corticeus subopacus* (Wallis, 1933)** CAN (AB BC) USA (AK CO ID ME MI MT NC NH NY PA WA WI WV WY)

*Hypophloeus subopacus* Wallis, 1933: 247.

***Corticeus substriatus* (LeConte, 1878)** CAN (BC) USA (AZ CA CO ID MT NM NV OR SD UT WA) MEX (BC)

*Hypophloeus substriatus* LeConte, 1878a: 423.

***Corticeus tenuis* (LeConte, 1878)** CAN (AB BC NB NS ON QC) USA (AZ CA ID MA ME MI MN MT NH NY OR PA VA WA WI WV WY)

*Hypophlocus* [sic!] *tenuis* LeConte, 1878a: 424.

*Hypophloeus minor* Wallis, 1933: 248. Synonymy: Triplehorn (1990: 294).

*Hypophloeus occidentalis* Wallis, 1933: 249. Synonymy: Triplehorn (1990: 294).

**Subgenus Pogonophloeus Bremer, 1998**

*Pogonophloeus* Bremer, 1998: 9. Type species: *Hypophloeus thoracicus* Melsheimer, 1846, original designation.

***Corticeus cavus* (LeConte, 1866)** USA (AL DC IA KS KY MD MO MS NC OH OK PA TX VA WV WI)

*Hypophloeus cavus* LeConte, 1866b: 129.

***Corticeus hatchi* Boddy, 1957** USA (AZ CA CO NM OR)

*Corticeus hatchi* Boddy, 1957: 197.

***Corticeus thoracicus* (Melsheimer, 1846)** CAN (ON) USA (AL AR DC DE FL GA IN KY LA MD MN MO MS NC NJ NY OH OK PA SC TN TX VA WI WV) / BAH

*Hypophloeus thoracicus* Melsheimer, 1846: 63.

*Hypophloeus piliger* LeConte, 1878a: 422. Synonymy: Triplehorn (1990: 292).

**Subgenus Tylophloeus Bremer, 1998**

*Tylophloeus* Bremer, 1998: 10. Type species: *Hypophloeus flavipennis* Motschulsky, 1860, original designation.

***Corticeus glaber* (LeConte, 1878)** USA (AL AR DC FL GA LA MD MS NC NJ OH SC TN TX VA WV) / BAH

*Hypophloeus glaber* LeConte, 1878a: 422.

**Genus *Myonophloeus* Bremer and Lillig, 2017** [M]

*Myonophloeus* Bremer and Lillig, 2017: 68. Type species: *Corticeus tuberculatus* Triplehorn, 1979, original designation.

***Myonophloeus tuberculatus* (Triplehorn, 1979)** CUB

*Corticeus tuberculatus* Triplehorn, 1979: 48.

**Tribe Myrmechixenini Jacquelin du Val, 1858**

Myrméchixénites Jacquelin du Val, 1858: 223. Type genus: *Myrmechixenus* Chevrolat, 1835.

**Genus *Myrmechixenus* Chevrolat, 1835** [M]

*Myrmechixenus* Chevrolat, 1835: 267. Type species: *Myrmechixenus subterraneus* Chevrolat, 1835, monotypy.

*Myrmecoxenus* Agassiz, 1846: 243. Unjustified emendation of *Myrmechixenus* Chevrolat, 1835, not in prevailing usage.

***Myrmechixenus latridioides* Crotch, 1873** USA (CA DC SC TX WA) – Adventive

*Myrmecoxenus latridioides* Crotch, 1873: 363.

**Tribe Phaleriini Blanchard, 1845**

Phalériides Blanchard, 1845: 29. Type genus: *Phaleria* Latreille, 1802.

Sepedonastidae Gistel, 1856a: 382. Type genus: *Sepedonastes* Gistel, 1856 (= *Phaleria* Latreille, 1802).

Cataphronetini Reitter, 1917: 57. Type genus: *Cataphronetis* Lucas, 1846 (= *Phtora* Germar, 1836).

**Genus *Phaleria* Latreille, 1802** [F]

*Phaleria* Latreill﻿e, 1802: 162. Type species: *Tenebrio cadaverinus* Fabricius, 1792, subsequent designation (Westwood 1838: 32) (see ICZN 1975).

*Sepedonastes* Gistel, 1856a: 382. Type species: *Tenebrio bimaculatus* Herbst, 1799 (= *Dytiscus bimaculatus* Linnaeus, 1767), subsequent designation (Bouchard et al. 2005: 501). Synonymy: Bouchard et al. (2005: 501).

*Halophalerus* Crotch, 1874: 107. Type species: *Phaleria rotundata* LeConte, 1851, **present designation**. Synonymy: Austin (1880: 38).

***Phaleria championi* Triplehorn and Watrous, 1980** MEX (CL NA SI)

*Phaleria championi* Triplehorn and Watrous, 1980: 56.

***Phaleria debilis* LeConte, 1866** USA (CA) MEX (BC JA NA SO) GUA NIC

*Phaleria debilis* LeConte, 1866b: 126.

*Phaleria neotropicalis* Champion, 1886: 220. Synonymy: Triplehorn (1991: 267).

*Phaleria insularis* Champion, 1886: 221. Synonymy: Triplehorn and Watrous (1979: 288).

***Phaleria fulva* Fleutiaux and Sallé, 1890** DOM LAN / SA

*Phaleria fulva* Fleutiaux and Sallé, 1890: 423.

***Phaleria gracilipes* Casey, 1890** USA (AL LA TX) MEX (TB VE)

*Phaleria gracilipes* Casey, 1890b: 484.

*Phaleria lodingi* Blaisdell, 1932c: 116. Synonymy: Triplehorn and Watrous (1979: 292).

***Phaleria guatemalensis* Champion, 1886** MEX (GE JA OA SI) GUA / SA

*Phaleria guatemalensis* Champion, 1886: 218.

***Phaleria lata* Blaisdell, 1923** MEX (BC BS SO)

*Phaleria latus* Blaisdell, 1923: 276.

***Phaleria pacifica* Champion, 1886** MEX (NA) GUA NIC

*Phaleria pacifica* Champion, 1886: 220.

***Phaleria panamensis* Champion, 1886** MEX (BC CO GE JA MI NA SI SO TB) GUA BEL NIC PAN

*Phaleria panamensis* Champion, 1886: 218.

*Phaleria dytiscoides* Champion, 1886: 218. Synonymy: Triplehorn (1991: 263).

*Phaleria marginipennis* Champion, 1886: 219. Synonymy: Triplehorn (1991: 263).

*Phaleria opacicollis* Champion, 1886: 219. Synonymy: Triplehorn (1991: 263).

***Phaleria picipes* Say, 1824** USA (FL GA MD NC NJ SC VA) MEX (MO QR YU) BEL HON PAN / BAH CAY CUB HAI JAM PRI LAN / SA

*Phaleria picipes* Say, 1824b: 280.

*Phaleria pilatei* Chevrolat, 1879: ccxlix. Synonymy: Watrous and Triplehorn (1982: 19).

*Phaleria variabilis* Quedenfeldt, 1886: 128. Synonymy: Watrous and Triplehorn (1982: 20).

*Phaleria caymanensis* Marcuzzi, 1977: 34. Synonymy: Watrous and Triplehorn (1982: 19).

***Phaleria pilifera* LeConte, 1866** MEX (BC BS SO)

*Phaleria pilifera* LeConte, 1866b: 125.

***Phaleria punctipes* LeConte, 1878** USA (FL) MEX (QR) BEL / BAH BAR CUB CAY JAM LAN

*Phaleria punctipes* LeConte, 1878a: 421.

*Phaleria guadeloupensis* Fleutiaux and Sallé, 1890: 423. Synonymy: Watrous and Triplehorn (1982: 13).

*Phaleria jamaicensis* Marcuzzi, 1977: 36. Synonymy: Watrous and Triplehorn (1982: 13).

***Phaleria rotundata* LeConte, 1851** USA (CA) MEX (BC BS)

*Phaleria rotundata* LeConte, 1851: 148.

*Phaleria limbata* Horn, 1870: 375. Synonymy: Fall (1901: 173, as *limbalis*).

***Phaleria testacea* Say, 1824** USA (CT DE FL GA LA MA MD ME MS NC NH NJ NY RI SC TX VA) MEX (QR) / BAH CUB JAM HIS PRI LAN / SA

*Phaleria testacea* Say, 1824b: 280.

*Phaleria brasiliensis* Laporte, 1840: 219. Synonymy: Watrous and Triplehorn (1982: 19).

*Phaleria cayennensis* Laporte, 1840: 219. Synonymy: Triplehorn (1991: 267).

*Phaleria longula* LeConte, 1866b: 125. Synonymy: Triplehorn and Watrous (1979: 289).

*Phaleria angustata* Chevrolat, 1879: ccxlviii. Synonymy: Watrous and Triplehorn (1982: 19).

*Phaleria chevrolati* Fleutiaux and Sallé, 1890: 422. Synonymy: Watrous and Triplehorn (1982: 19).

Phaleria chevrolati var. thoracica Fleutiaux and Sallé, 1890: 423. Synonymy: Watrous and Triplehorn (1982: 19).

Phaleria chevrolati var. quadrinotata Fleutiaux and Sallé, 1890: 423. Synonymy: Watrous and Triplehorn (1982: 19).

*Phaleria maculipennis* Marcuzzi, 1962: 37. Synonymy: Watrous and Triplehorn (1982: 19).

***Phaleria thinophila* Watrous and Triplehorn, 1982** CRI / JAM DOM PRI LAN / SA

*Phaleria thinophila* Watrous and Triplehorn, 1982: 15.

**Genus *Phaleromela* Reitter, 1916** [F]

*Phaleromela* Reitter, 1916: 4. Type species: *Phaleria subhumeralis* Marseul, 1876, monotypy.

***Phaleromela humeralis* (Laporte, 1840)** USA (CA)

*Phaleria humeralis* Laporte, 1840: 219.

***Phaleromela picta* (Mannerheim, 1843)** CAN (BC) USA (AK CA OR WA)

*Phaleria picta* Mannerheim, 1843: 277.

*Phaleria globosa* LeConte, 1857: 51. **New synonymy** [YB].

***Phaleromela prohumeralis* Triplehorn, 1961** USA (CA)

*Phaleria humeralis* Horn, 1870: 377 [junior primary homonym of *Phaleria humeralis* Laporte, 1840].

*Phaleromela prohumeralis* Triplehorn, 1961: 127. Replacement name for *Phaleromela humeralis* (Horn, 1870).

**Figure 38.**
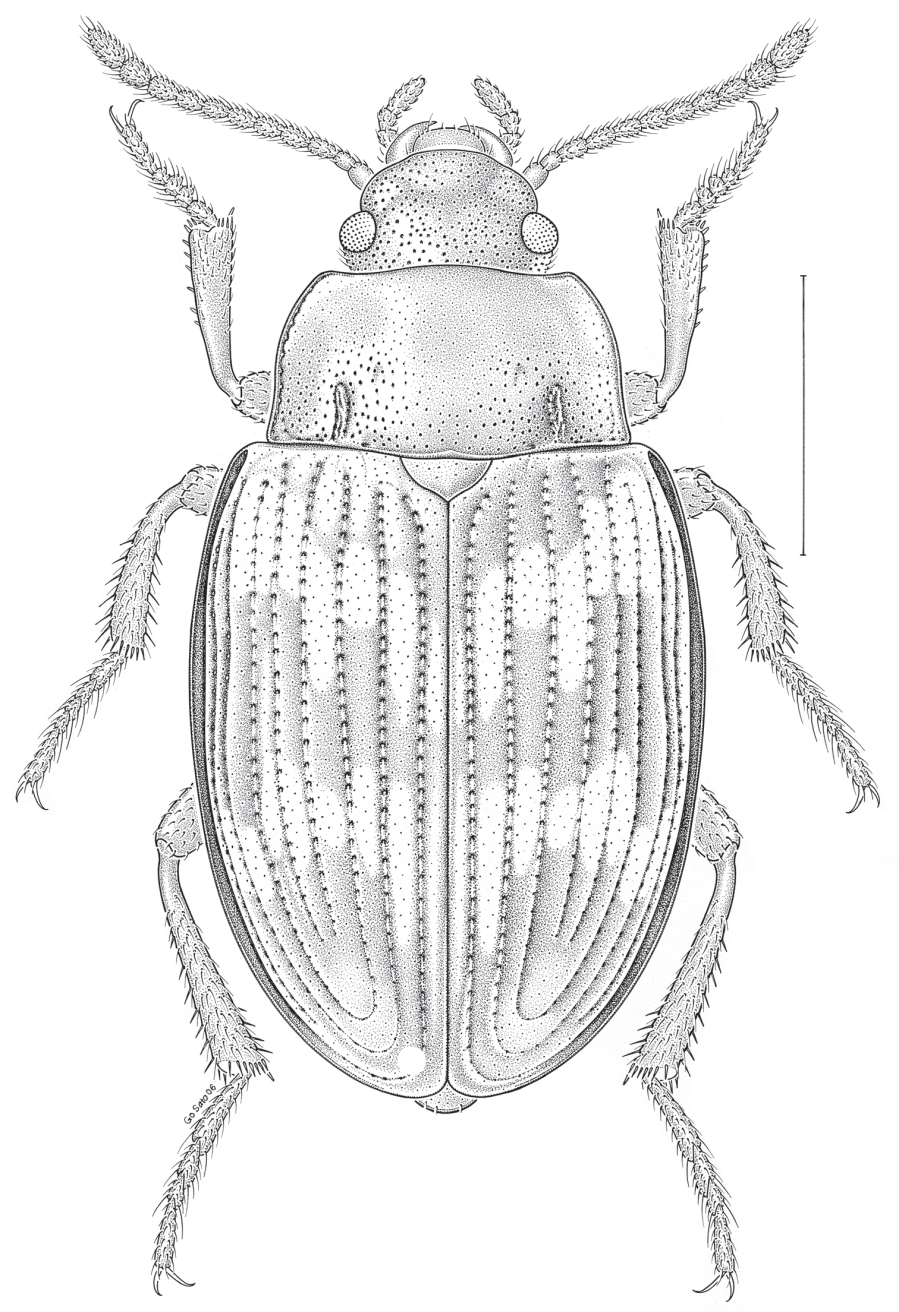
*Phaleromela variegata* Triplehorn, 1961. Scale bar = 1 mm.

***Phaleromela variegata* Triplehorn, 1961** [Fig. 38] CAN (AB BC NT SK YT) USA (CA ID OR WA)

*Scaphidema pictum* Horn, 1874a: 36 [junior secondary homonym of *Phaleromela picta* (Mannerheim, 1843)].

*Phaleromela variegata* Triplehorn, 1961: 126. Replacement name for *Phaleromela picta* (Horn, 1874).

**Tribe Scaphidemini Reitter, 1922**

Scaphidemini Reitter, 1922: 2. Type genus: *Scaphidema* Redtenbacher, 1848.

**Genus *Scaphidema* Redtenbacher, 1848** [F]

*Scaphidema* Redtenbacher, 1848: 591. Type species: *Scaphidium bicolor* Fabricius, 1798, monotypy.

*Nelites* LeConte, 1850: 232. Type species: *Nelites aeneolus* LeConte, 1850, monotypy. Synonymy: LeConte (1862a: 237).

*Microbasanus* Pic, 1921: 1. Type species: *Microbasanus jureceki* Pic, 1921, monotypy. Synonymy: Löbl et al. (2008a: 318) (see also Schawaller 2008: 384).

***Scaphidema aeneola* (LeConte, 1850)** CAN (AB BC LB MB NB NS ON PE QC SK YT) USA (AK CT IL MA MI MN NH NY OH PA WI WY)

*Nelites aeneolus* LeConte, 1850: 232.

**Tribe Trachyscelini Blanchard, 1845**

Trachyscélides Blanchard, 1845: 28. Type genus: *Trachyscelis* Latreille, 1809.

**Genus *Trachyscelis* Latreille, 1809** [F]

*Trachyscelis* Latreille, 1809: 379. Type species: *Trachyscelis aphodioides* Latreille, 1809, monotypy.

***Trachyscelis aphodioides* Latreille, 1809** USA^76^ (FL LA MD NC VA) / BAH TUR PRI LAN – Adventive

*Trachyscelis aphodioides* Latreille, 1809: 379.

*Trachyscelis flavipes* Melsheimer, 1846: 61. Synonymy: Steiner (2004: 335).

**Subfamily STENOCHIINAE Kirby, 1837**

Stenochiadae Kirby, 1837: 238. Type genus: *Stenochia* Kirby, 1819 (= *Strongylium* Kirby, 1819).

**Tribe Cnodalonini Oken, 1843**

Cnodaliden Oken, 1843: 484. Type genus: *Cnodalon* Latreille, 1797.

Coelometopidae Schaum, 1859: 71. Type genus: *Coelometopus* Solier, 1848.

Upidae C.G. Thomson, 1859: 116. Type genus: *Upis* Fabricius, 1792.

Catapiestides Lacordaire, 1859: 381. Type genus: *Catapiestus* Perty, 1831.

Polypleuri LeConte, 1862a: 229. Type genus: *Polypleurus* Eschscholtz, 1831.

Hegemonini Reitter, 1922: 3. Type genus: *Hegemona* Laporte, 1840.

Camariinae Anonymous, 1924: 164. Type genus: *Camaria* Lepeletier and Audinet-Serville, 1828.

**Genus *Alobates* Motschulsky, 1872** [M]

*Alobates* Motschulsky, 1872: 25. Type species: *Tenebrio pensylvanicus* DeGeer, 1775, original designation.

***Alobates barbatus* (Knoch, 1801)** ^77^ USA (AL AR DC DE FL GA IL IN KS KY LA MA MD MI MO MS NC NJ NY OH RI SC TN TX VA WI WV)

*Upis glabra* Herbst, 1799: 32 [*nomen dubium*].

*Tenebrio barbatus* Knoch, 1801: 166. Synonymy (in doubt): LeConte (1866a: 61).

*Nyctobates intermedia* Haldeman, 1852: 376. Synonymy: Horn (1870: 333).

***Alobates pensylvanicus* (DeGeer, 1775)** CAN (MB NB NS ON QC) USA (AL AZ CA CT DC DE FL GA IA IN KS KY LA MA MD ME MI MN MS NC NH NJ OH OR PA SC SD TN TX VA WI WV) MEX (NL)

*Tenebrio pensylvanicus* DeGeer, 1775: 53.

*Upis chrysops* Herbst, 1797: 236. Synonymy (in doubt): Knoch (1801: 168).

*Tenebrio sublaevis* Palisot de Beauvois, 1817: 163. Synonymy: LeConte (1866a: 61).

*Tenebrio striatellus* Drapiez, 1820: 327. Synonymy: Horn (1889: 212).

*Nyctobates inermis* Mannerheim, 1843: 284. Synonymy: Horn (1870: 333).

*Alobates pennsylvanicus* Champion, 1892: 522. Unjustified emendation of *Alobates pensylvanicus* (DeGeer, 1775), not in prevailing usage.

**Genus *Apsida* Lacordaire, 1859** [F]

*Apsida* Lacordaire, 1859: 309. Type species: *Apsida chrysomelina* Lacordaire, 1859, original designation.

*Hapsida* Gemminger [in Gemminger and Harold], 1870: 1955. Unjustified emendation of *Apsida* Lacordaire, 1859, not in prevailing usage.

***Apsida belti* Bates, 1873** USA (TX) MEX (TA TB VE YU) GUA NIC CRI

*Apsida belti* Bates, 1873e: 16.

***Apsida boucardi* Bates, 1873** MEX (VE) GUA BEL CRI PAN

*Apsida boucardi* Bates, 1873e: 17.

*Cosmonota geminata* Chevrolat, 1877b: 173. Synonymy: Champion (1886: 215).

*Cosmonota grammica* Chevrolat, 1877b: 173. Synonymy: Champion (1886: 215).

***Apsida chrysomelina* Lacordaire, 1859** MEX (CI VE) GUA BEL NIC CRI PAN / SA

*Apsida chrysomelina* Lacordaire, 1859: 309.

*Apsida chrysomelina* Bates, 1873e: 15 [junior primary homonym of *Apsida chrysomelina* Lacordaire, 1859]. Synonymy: Champion (1886: 211).

***Apsida gibbosa* Champion, 1886** MEX (VE) GUA BEL CRI

*Hapsida gibbosa* Champion, 1886: 212.

***Apsida lustrans* Triplehorn, 1970** CRI

*Apsida lustrans* Triplehorn, 1970: 569.

***Apsida punctipennis* Champion, 1886** GUA

*Hapsida punctipennis* Champion, 1886: 213.

***Apsida purpureomicans* Bates, 1873** MEX (VE) GUA BEL CRI PAN / SA

*Apsida purpureomicans* Bates, 1873e: 16.

*Apsida aeneomicans* Bates, 1873e: 16. Synonymy: Triplehorn (1970: 571).

***Apsida seriatopunctata* Champion, 1886** MEX (VE)

*Hapsida seriato-punctata* Champion, 1886: 212.

***Apsida simulatrix* Ferrer and Ødegaard, 2005** PAN

*Apsida simulatrix* Ferrer and Ødegaard, 2005: 640.

***Apsida terebrans* Champion, 1886** MEX (TB) GUA BEL NIC CRI PAN

*Hapsida terebrans* Champion, 1886: 214.

**Genus *Blapida* Perty, 1830** [F]

*Blapida* Perty, 1830: 58. Type species: *Blapida okeni* Perty, 1830, monotypy.

*Ryssochiton* Gray [in Griffith and Pidgeon], 1832: pl. 50. Type species: *Rhyssochiton politus* Gray, 1832, monotypy. Synonymy: Lacordaire (1859: 425).

***Blapida alternata* Gebien, 1919** CRI PAN

*Blapida alternata* Gebien, 1919: 137.

***Blapida castaneipennis* Champion, 1896** LAN

*Blapida castaneipennis* Champion, 1896: 28.

***Blapida neotropicalis* Champion, 1886** GUA NIC

*Blapida neotropicalis* Champion, 1886: 247.

**Genus *Bothynocephalus* Doyen, 1988** [M]

*Bothynocephalus* Doyen, 1988: 315. Type species: *Bothynocephalus cristatus* Doyen, 1988, original designation.

***Bothynocephalus cristatus* Doyen, 1988** HON

*Bothynocephalus cristatus* Doyen, 1988: 316.

***Bothynocephalus foveolatus* Doyen, 1995** MEX (OA)

*Bothynocephalus foveolatus* Doyen, 1995: 12.

***Bothynocephalus ribardoi* Doyen, 1995** CRI

*Bothynocephalus ribardoi* Doyen, 1995: 13.

***Bothynocephalus thoracicus* Doyen, 1995** CRI

*Bothynocephalus thoracicus* Doyen, 1995: 12.

**Genus *Brosimapsida* Ferrer and Ødegaard, 2005** [F]

*Brosimapsida* Ferrer and Ødegaard, 2005: 640. Type species: *Brosimapsida gonospoides* Ferrer and Ødegaard, 2005, original designation.

***Brosimapsida gonospoides* Ferrer and Ødegaard, 2005** PAN

*Brosimapsida gonospoides* Ferrer and Ødegaard, 2005: 640.

**Genus *Calydonella* Doyen, 1995** [F]

*Calydonella* Doyen, 1995: 8. Type species: *Calydonella lisa* Doyen, 1995, original designation.

***Calydonella lisa* Doyen, 1995** CRI PAN

*Calydonella lisa* Doyen, 1995: 10.

**Genus *Camaria* Lepeletier and Audinet-Serville, 1828** [F]

*Camaria* Lepeletier and Audinet-Serville, 1828: 454. Type species: *Camaria nitida* Lepeletier and Audinet-Serville, 1828, monotypy.

***Camaria laevis* Gebien, 1919** “Zentralamerika” / SA

*Camaria laevis* Gebien, 1919: 52.

***Camaria parallela* Champion, 1886** PAN

*Camaria parallela* Champion, 1886: 246.

**Genus *Choastes* Champion, 1893** [M]

*Choaspes* Champion, 1885: 118 [junior homonym of *Choaspes* Moore, 1881]. Type species: *Choaspes purpureus* Champion, 1885, subsequent designation (Gebien 1941: 338).

*Choastes* Champion, 1893a: 526. Replacement name for *Choaspes* Champion, 1885.

***Choastes angulicollis* (Champion, 1885)** NIC PAN

*Choaspes angulicollis* Champion, 1885: 119.

***Choastes purpureus* (Champion, 1885)** GUA BEL NIC PAN

*Choaspes purpureus* Champion, 1885: 119.

**Genus *Cibdelis* Mannerheim, 1843** [F]

*Cibdelis* Mannerheim, 1843: 282. Type species: *Cibdelis blaschkii* Mannerheim, 1843, monotypy.

*Scotera* Motschulsky, 1845b: 365. Type species: *Scotera gibbosa* Motschulsky, 1845, monotypy.^78^ Synonymy: Motschulsky (1845b: 365).

***Cibdelis bachei* LeConte, 1861** USA (CA)

*Cibdelis bachei* LeConte, 1861b: 353.

***Cibdelis blaschkii* Mannerheim, 1843** USA (CA)

*Cibdelis blaschkii* Mannerheim, 1843: 284.

***Cibdelis cylindrica* Casey, 1924** USA (CA)

*Cibdelis cylindrica* Casey, 1924: 323.

***Cibdelis gibbosa* (Motschulsky, 1845)** USA (CA)

*Scotera gibbosa* Motschulsky, 1845b: 365.

*Cibdelis laevigata* Casey, 1891: 60. Synonymy: Leng (1920: 235).

***Cibdelis ventricosa* Casey, 1924** USA (CA)

*Cibdelis ventricosa* Casey, 1924: 322.

**Genus *Cnephalura* Doyen, 1988** [F]

*Cnephalura* Doyen, 1988: 313. Type species: *Cnephalura umbrata* Doyen, 1988, original designation.

***Cnephalura umbrata* Doyen, 1988** MEX (CI)

*Cnephalura umbrata* Doyen, 1988: 313.

**Genus *Cnodalon* Latreille, 1797** [N]

*Cnodalon* Latreille, 1797: 23. Type species: *Cnodalum viride* Latreille, 1804, subsequent monotypy in Latreille (1804: 321).

*Cnodalum* Agassiz, 1846: 91. Unjustified emendation for *Cnodalon* Latreille, 1797, not in prevailing usage.

***Cnodalon viride* Latreille, 1804** HAI

*Cnodalum viride* Latreille, 1804: 321.

**Genus *Coelocnemis* Mannerheim, 1843** [F]

*Coelocnemis* Mannerheim, 1843: 280. Type species: *Coelocnemis dilaticollis* Mannerheim, 1843, subsequent designation (Lucas 1920: 194).

***Coelocnemis dilaticollis* Mannerheim, 1843** ^79^CAN (AB BC) USA (CA ID MT NV OR UT WA WY)

*Coelocnemis dilaticollis* Mannerheim, 1843: 282.

*Coelocnemis californica* Mannerheim, 1843: 282. Synonymy: Horn (1870: 336).

*Coelocnemis rugosa* Linell, 1899: 181. Synonymy: Doyen (1973: 93).

*Coelocnemis columbiana* Casey, 1924: 314. Synonymy: Boddy (1965: 175).

*Coelocnemis rauca* Casey, 1924: 315. Synonymy: Doyen (1973: 93).

*Coelocnemis ovipennis* Casey, 1924: 315. Synonymy: Doyen (1973: 93).

*Coelocnemis utensis* Casey, 1924: 316. Synonymy: Doyen (1973: 93).

*Coelocnemis spaldingi* Casey, 1924: 316. Synonymy: Doyen (1973: 93).

*Coelocnemis basalis* Casey, 1924: 316. Synonymy: Doyen (1973: 93).

*Coelocnemis idahoensis* Casey, 1924: 318. Synonymy: Boddy (1965: 175).

*Coelocnemis barretti* Blaisdell, 1928: 163. Synonymy: Doyen (1973: 93).

***Coelocnemis lucia* Doyen, 1973** USA (CA)

*Coelocnemis lucia* Doyen, 1973: 96.

***Coelocnemis magna* LeConte, 1851** USA (AZ CA NM) MEX (BC)

*Coelocnemis magna* LeConte, 1851: 150.

*Coelocnemis obesa* LeConte, 1851: 150. Synonymy: Doyen (1973: 87).

*Coelocnemis caudicalis* Casey, 1924: 316. Synonymy: Doyen (1973: 87).

*Coelocnemis caudicalis deserta* Casey, 1924: 316. Synonymy: Doyen (1973: 87).

*Coelocnemis antennalis* Casey, 1924: 317. Synonymy: Doyen (1973: 87).

*Coelocnemis aequalis* Casey, 1924: 318. Synonymy: Doyen (1973: 87).

*Coelocnemis smithiana* Casey, 1924: 318. Synonymy: Doyen (1973: 87).

*Coelocnemis rotundicollis* Casey, 1924: 319. Synonymy: Doyen (1973: 87).

*Coelocnemis longicollis* Casey, 1924: 319. Synonymy: Doyen (1973: 87).

***Coelocnemis punctata* LeConte, 1854** USA (AZ CA CO ID NM NV OR UT)

*Coelocnemis punctatus* LeConte, 1854c: 225.

*Coelocnemis angusta* Casey, 1924: 320. Synonymy: Doyen (1973: 98).

*Coelocnemis tanneri* Blaisdell, 1928: 164. Synonymy: Doyen (1973: 98).

***Coelocnemis rugulosa* Doyen, 1973** USA (CA OR)

*Coelocnemis rugulosa* Doyen, 1973: 97.

***Coelocnemis slevini* Blaisdell, 1925** MEX (BC)

*Coelocnemis slevini* Blaisdell, 1925b: 337.

***Coelocnemis sulcata* Casey, 1895** USA (AZ CA ID NV UT)

*Coelocnemis sulcata* Casey, 1895: 615.

**Genus *Cyrtosoma* Perty, 1830** [N]

*Cyrtosoma* Perty, 1830: 59. Type species: *Cyrtosoma unicolor* Perty, 1830, monotypy.

***Cyrtosoma arimense* Marcuzzi, 1999** LAN

*Cyrtosoma arimensis* Marcuzzi, 1999: 85.

***Cyrtosoma decemlineatum* Champion, 1886** MEX (VE) BEL NIC PAN

*Cyrtosoma decem-lineatum* Champion, 1886: 244.

***Cyrtosoma denticolle* Chevrolat, 1878** MEX (VE) GUA BEL NIC CRI PAN / SA

*Cyrtosoma denticolle* Chevrolat, 1878e: 273.

***Cyrtosoma grenadense* Marcuzzi, 1999** LAN (Grenada)

*Cyrtosoma grenadensis* Marcuzzi, 1999: 85.

***Cyrtosoma lherminierii* (Guérin-Méneville, 1844)** LAN

*Cnodalon atrum* Guérin-Méneville, 1833: pl. 31 [junior primary homonym of *Cnodalon atrum* Lepeletier and Audinet-Serville, 1825].

*Cnodalon l’herminierii* Guérin-Méneville^80^, 1844: 123. Replacement name for *Cnodalon atrum* Guérin-Méneville, 1833.

***Cyrtosoma martiniquense* Marcuzzi, 1999** LAN (Martinique)

*Cyrtosoma martiniquensis* Marcuzzi, 1999: 83.

***Cyrtosoma piceum* (Laporte and Brullé, 1831)** LAN (Guadeloupe)

*Platydema picea* Laporte and Brullé, 1831: 362.

***Cyrtosoma williamsi* Marcuzzi, 1992** PAN

*Cyrtosoma williamsi* Marcuzzi, 1992: 237.

**Genus *Dinomus* Brême, 1842** [M]

*Dinomus* Brême, 1842: 113. Type species: *Dinomus perforatus* Brême, 1842, monotypy.

***Dinomus perforatus* Brême, 1842** MEX

*Dinomus perforatus* Brême, 1842: 114.

**Genus *Elomosda* Bates, 1870** [F]

*Elomosda* Bates, 1870: 273. Type species: *Elomosda beltii* Bates, 1870, monotypy.

***Elomosda beltii* Bates, 1870** GUA NIC CRI

*Elomosda beltii* Bates, 1870: 275.

**Genus *Epicalla* Champion, 1886** [F]

*Epicalla* Champion, 1886: 249. Type species: *Epicalla varipes* Champion, 1886, subsequent designation (Lucas 1920: 268).

***Epicalla aenipes* Ferrer and Ødegaard, 2005** PAN

*Epicalla aenipes* Ferrer and Ødegaard, 2005: 642.

***Epicalla agnata* Gebien, 1928** CRI

*Epicalla agnata* Gebien, 1928b: 217.

***Epicalla avia* Gebien, 1928** BEL

*Epicalla avia* Gebien, 1928b: 209.

***Epicalla cupreonitens* Champion, 1886** PAN

*Epicalla cupreo-nitens* Champion, 1886: 250.

***Epicalla elongata* Ferrer and Ødegaard, 2005** PAN

*Epicalla elongata* Ferrer and Ødegaard, 2005: 642.

***Epicalla famula* Gebien, 1928** CRI

*Epicalla famula* Gebien, 1928b: 216.

***Epicalla hera* Gebien, 1928** CRI

*Epicalla hera* Gebien, 1928b: 207.

***Epicalla instriata* Pic, 1921** PAN

*Epicalla instriata* Pic, 1921b: 28.

***Epicalla juvenca* Gebien, 1928** NIC CRI

*Epicalla juvenca* Gebien, 1928b: 218.

***Epicalla lata* Champion, 1886** MEX (JA SI)

*Epicalla lata* Champion, 1886: 250.

***Epicalla nevermanni* Gebien, 1928** CRI

*Epicalla nevermanni* Gebien, 1928b: 215.

***Epicalla pygmaea* Ferrer and Ødegaard, 2005** PAN

*Epicalla pygmaea* Ferrer and Ødegaard, 2005: 642.

***Epicalla varipes* Champion, 1886** NIC

*Epicalla varipes* Champion, 1886: 249.

**Genus *Glyptotus* LeConte, 1858** [M]

*Glyptotus* LeConte, 1858b: 75. Type species: *Glyptotus cribratus* LeConte, 1858, monotypy.

***Glyptotus cribratus* LeConte, 1858** USA (AL FL GA MS NC SC TX VA) MEX / BAH

*Glyptotus cribratus* LeConte, 1858b: 75.

***Glyptotus nitidus* Champion, 1885** MEX (VE) NIC

*Glyptotus nitidus* Champion, 1885: 113.

***Glyptotus yucatanus* Champion, 1892** MEX (YU)

*Glyptotus yucatanus* Champion, 1892: 524.

**Genus *Gonospa* Champion, 1886** [F]

*Gonospa* Champion, 1886: 216. Type species: *Gonospa phaedonoides* Champion, 1886, subsequent designation (Gebien 1940: 426).

***Gonospa phaedonoides* Champion, 1886** PAN

*Gonospa phaedonoides* Champion, 1886: 217.

***Gonospa similis* Ferrer and Ødegaard, 2005** PAN

*Gonospa similis* Ferrer and Ødegaard, 2005: 639.

**Genus *Haplandrus* LeConte, 1862** [M]

*Haplandrus* LeConte, 1862a: 230. Type species: *Helops femoratus* Fabricius, 1798 (= *Upis fulvipes* Herbst, 1797), monotypy.

***Haplandrus deyruporum* Steiner, 2016** USA (FL)

*Haplandrus deyruporum* Steiner, 2016: 537.

***Haplandrus fulvipes* (Herbst, 1797)** CAN (NS ON QC) USA (CT FL GA IA IN MD MI NC NY OH PA RI SC TN VA WI)

*Upis fulvipes* Herbst, 1797: 238.

*Helops femoratus* Fabricius, 1798: 53. Synonymy: Schönherr (1806: 157).

**Genus *Hegemona* Laporte, 1840** [F]

*Hegemona* Laporte, 1840: 230. Type species: *Hegemona resplendens* Laporte, 1840, monotypy.

*Eucamptus* Germar, 1842: 444 [junior homonym of *Eucamptus* Chevrolat, 1833]. Type species: *Eucamptus iridis* Germar, 1842 (= *Hegemona resplendens* Laporte, 1840), monotypy. Synonymy: Duponchel (1845: 498).

*Eusarca* Chevrolat, 1845: 526. Type species: *Eusarca iridipennis* Chevrolat, 1845 (= *Hegemona resplendens* Laporte, 1840), monotypy. Synonymy: Duponchel (1845: 498).

***Hegemona alternata* Pic, 1936** GUA

*Hegemona alternatus* Pic, 1936: 15.

***Hegemona angustata* Champion, 1887** GUA

*Hegemona angustatus* Champion, 1887: 272.

***Hegemona bicaudata* Champion, 1887** GUA

*Hegemona bicaudatus* Champion, 1887: 270.

***Hegemona chiriquensis* Champion, 1887** CRI PAN

*Hegemona chiriquensis* Champion, 1887: 273.

***Hegemona compressa* Allard, 1877** MEX GUA

*Hegemona compressus* Allard, 1877b: 61, 254.

***Hegemona costaricensis* Champion, 1887** CRI

*Hegemona costaricensis* Champion, 1887: 275.

***Hegemona elongata* Allard, 1877** MEX (YU)

*Hegemona elongatus* Allard, 1877b: 61, 253.

***Hegemona flibuster* (J. Thomson, 1856)** MEX GUA BEL NIC CRI

*Eucamptus flibuster* J. Thomson, 1856: 475.

*Hegemona filibuster* Champion, 1887: 275. Unjustified emendation of *Hegemona flibuster* (J. Thomson, 1856), not in prevailing usage.

***Hegemona furcillata* Allard, 1877** MEX

*Hegemona furcillatus* Allard, 1877b: 61, 252.

***Hegemona guatemalensis* Champion, 1887** GUA

*Hegemona guatemalensis* Champion, 1887: 274.

***Hegemona hondurensis* Champion, 1887** BEL

*Hegemona hondurensis* Champion, 1887: 269.

***Hegemona interrupta* Champion, 1887** CRI

*Hegemona interruptus* Champion, 1887: 275.

***Hegemona lineata* Champion, 1887** GUA BEL

*Hegemona lineatus* Champion, 1887: 271.

***Hegemona mexicana* Champion, 1887** MEX (PU)

*Hegemona mexicanus* Champion, 1887: 274.

***Hegemona nigra* Champion, 1887** GUA HON

*Hegemona niger* Champion, 1887: 271.

***Hegemona refulgens* Champion, 1893** GUA

*Hegemona refulgens* Champion, 1893a: 549.

***Hegemona resplendens* Laporte, 1840** MEX (VE YU)

*Hegemona resplendens* Laporte, 1840: 230.

*Eucamptus iridis* Germar, 1842: 444. Synonymy: Duponchel (1845: 498).

*Eusarca iridipennis* Chevrolat, 1845: 526. Synonymy: Duponchel (1845: 498).

***Hegemona retrodentata* Allard, 1877** MEX (CI OA)

*Hegemona retrodentatus* Allard, 1877b: 61, 253.

***Hegemona zunilensis* Champion, 1887** GUA

*Hegemona zunilensis* Champion, 1887: 272.

**Genus *Hesiodus* Champion, 1885** [M]

*Hesiodus* Champion, 1885: 115. Type species: *Hesiodus longitarsus* Champion, 1885, subsequent designation (Lucas 1920: 323).

***Hesiodus caraibus* Fleutiaux and Sallé, 1890** LAN (Guadeloupe)

*Hesiodus caraibus* Fleutiaux and Sallé, 1890: 424.

***Hesiodus conspurcatus* Champion, 1885** PAN

*Hesiodus conspurcatus* Champion, 1885: 116.

***Hesiodus debilis* Champion, 1885** GUA

*Hesiodus debilis* Champion, 1885: 117.

***Hesiodus ellipticus* Champion, 1893** NIC

*Hesiodus ellipticus* Champion, 1893a: 525.

***Hesiodus jansoni* Champion, 1885** MEX (YU) NIC

*Hesiodus jansoni* Champion, 1885: 116.

***Hesiodus longitarsis* Champion, 1885** MEX (VE) BEL NIC

*Hesiodus longitarsis* Champion, 1885: 115.

***Hesiodus sordidus* Champion, 1885** MEX (VE)

*Hesiodus sordidus* Champion, 1885: 116.

**Genus *Hicetaon* Champion, 1885** [M]

*Hicetaon* Champion, 1885: 111. Type species: *Hicetaon frontalis* Champion, 1885, monotypy.

***Hicetaon frontalis* Champion, 1885** MEX (VE YU) BEL

*Hicetaon frontalis* Champion, 1885: 112.

**Genus *Ilus* Champion, 1885** [M]

*Ilus* Champion, 1885: 117. Type species: *Ilus apicicornis* Champion, 1885, monotypy.

***Ilus apicicornis* Champion, 1885** BEL CRI

*Ilus apicicornis* Champion, 1885: 118.

**Genus *Iphthiminus* Spilman, 1973** [M]

*Iphthiminus* Spilman, 1973: 42. Type species: *Iphthimus italicus* Truqui, 1857, original designation.

***Iphthiminus lewisii* (Horn, 1870)** USA (AZ CA CO NM NV TX UT WY) MEX (BC CH)

*Iphthimus lewisii* Horn, 1870: 335.

*Iphthimus laevissimus* Casey, 1890b: 408. Synonymy: Gardiner and Pollock (2015: 364).

***Iphthiminus opacus* (LeConte, 1866)** CAN (AB MB NB NS ON QC SK) USA (AZ CA CT IN MA ME MI MN MT NH NY OH PA SD VA VT WI WY)

*Iphthimus opacus* LeConte, 1866b: 121.

**Figure 39.**
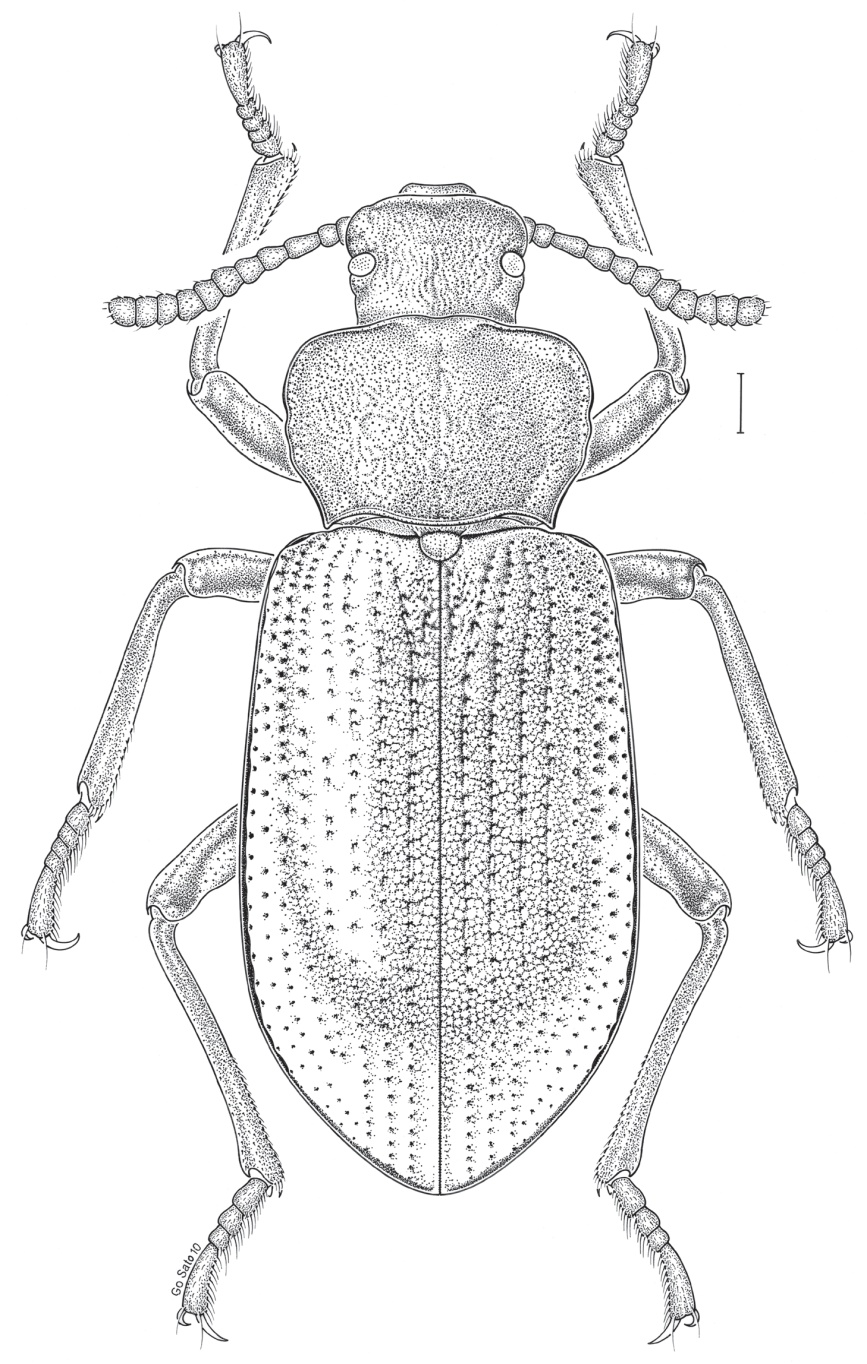
*Iphthiminus serratus* (Mannerheim, 1843). Scale bar = 1 mm.

***Iphthiminus serratus* (Mannerheim, 1843)** [Fig. 39] CAN (AB BC) USA (CA ID MT NE NV OR WA WY)

*Nyctobates serrata* Mannerheim, 1843: 284.

*Nyctobates sublaevis* Bland, 1865: 382. Synonymy: Gardiner and Pollock (2015: 356).

*Iphthinus servilis* Walker, 1866: 326. Synonymy: Horn (1870: 334).

*Iphthinus servator* Walker, 1866: 327. Synonymy: Horn (1870: 334).

*Iphthinus subligatus* Walker, 1866: 327. Synonymy: Horn (1870: 334).

*Iphthimus salebrosus* Casey, 1924: 327. Synonymy: Gardiner and Pollock (2015: 356).

**Genus *Isaminas* Champion, 1887** [M]

*Isaminas* Champion, 1887: 266. Type species: *Isaminas gibbipennis* Champion, 1887, subsequent designation (Gebien 1943: 401).

*Pteroglymmius* Gebien, 1928b: 223. Type species: *Pteroglymmius erotyloides* Gebien, 1928, monotypy. Synonymy: Doyen (1988: 301).

***Isaminas breedlovei* Doyen, 1988** MEX (CI)

*Isaminas breedlovei* Doyen, 1988: 303.

***Isaminas brevicollis* Champion, 1887** MEX (CI) GUA

*Isaminas brevicollis* Champion, 1887: 267.

***Isaminas erotyloides* (Gebien, 1928)** HON

*Pteroglymmius erotyloides* Gebien, 1928b: 224.

***Isaminas gibbipennis* Champion, 1887** NIC CRI

*Isaminas gibbipennis* Champion, 1887: 267.

***Isaminas reticuloides* Doyen, 1988** MEX (CI)

*Isaminas reticuloides* Doyen, 1988: 303.

***Isaminas sullivani* Doyen, 1988** CRI

*Isaminas sullivani* Doyen, 1988: 306.

**Genus *Isicerdes* Champion, 1885** [M]

*Isicerdes* Champion, 1885: 113. Type species: *Isicerdes occultus* Champion, 1885, subsequent designation (Lucas 1920: 353).

***Isicerdes funebris* Champion, 1885** GUA

*Isicerdes funebris* Champion, 1885: 114.

***Isicerdes occultus* Champion, 1885** MEX (VE) GUA BEL PAN

*Isicerdes occultus* Champion, 1885: 114.

***Isicerdes vicinus* Champion, 1892** MEX (JA YU) CRI

*Isicerdes vicinus* Champion, 1892: 524.

**Genus *Lenkous* Kaszab, 1973** [M]

*Lenkous* Kaszab, 1973: 315. Type species: *Lenkous myrmecophilus* Kaszab, 1973, original designation.

***Lenkous ibisca* Ferrer and Ødegaard, 2005** PAN

*Lenkous ibisca* Ferrer and Ødegaard, 2005: 639.

**Genus *Merinus* LeConte, 1862** [M]

*Merinus* LeConte, 1862a: 230. Type species: *Tenebrio laevis* Olivier, 1795, original designation.

**Figure 40.**
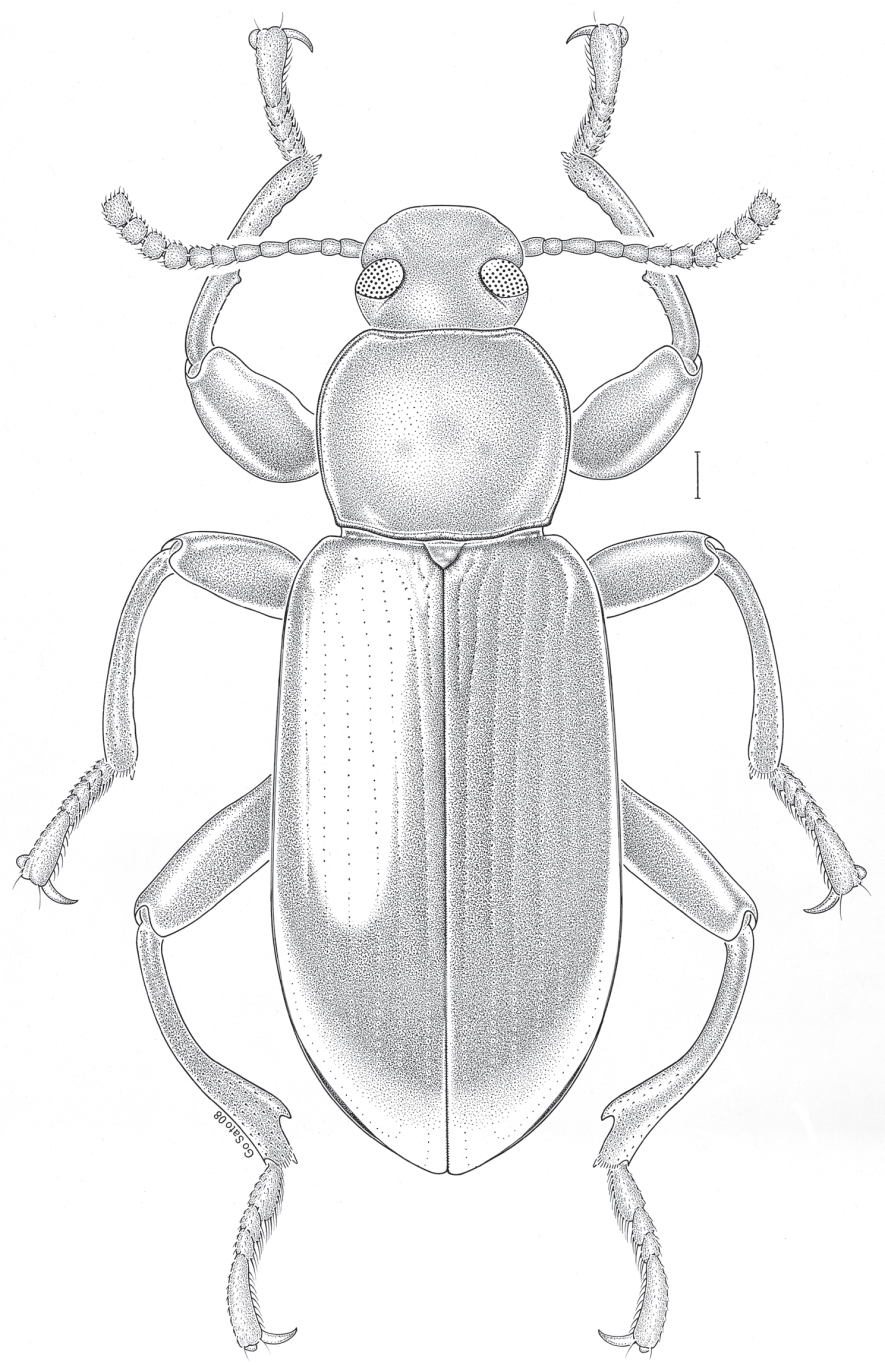
*Merinus laevis* (Olivier, 1795). Scale bar = 1 mm.

***Merinus laevis* (Olivier, 1795)** [Fig. 40] CAN (ON QC) USA (AL CT FL DC GA IA IL IN KS MD MI MO MS NC NJ NY OH OK PA RI SC TN VA WI WV)

*Tenebrio laevis* Olivier, 1795: [57] 10.

**Genus *Mitys* Champion, 1885** [M]

*Mitys* Champion, 1885: 97. Type species: *Mitys inflatus* Champion, 1885, subsequent designation (Gebien 1943: 402).

***Mitys inflatus* Champion, 1885** MEX (OA VE)

*Mitys inflatus* Champion, 1885: 97.

***Mitys opacus* Champion, 1885** MEX (TA)

*Mitys opacus* Champion, 1885: 98.

***Mitys politus* (Brême, 1842)** MEX (PU VE)

*Sphoerotus politus* Brême, 1842: 109.

*Mitys laevis* Champion, 1885: 98. Synonymy: Champion (1892: 520).

**Genus *Moeon* Champion, 1886** [N]^81^

*Moeon* Champion, 1886: 251. Type species: *Moeon isthmicus* Champion, 1886, subsequent designation (Gebien 1942: 330).

***Moeon isthmicum* Champion, 1886** PAN

*Moeon isthmicus* Champion, 1886: 251.

***Moeon panamense* Champion, 1886** PAN

*Moeon panamensis* Champion, 1886: 251.

**Genus *Mophon* Champion, 1886** [M]

*Mophon* Champion, 1886: 247. Type species: *Mophon tinctipennis* Champion, 1886, monotypy.

***Mophon tinctipennis* Champion, 1886** NIC PAN

*Mophon tinctipennis* Champion, 1886: 248.

**Genus *Mylaris* Pallas, 1781** [F]

*Mylaris* Pallas, 1781: 37^82^. Type species: *Tenebrio gigas* Linnaeus, 1763, subsequent designation (Guérin-Méneville 1844: 120).

*Iphthinus* Dejean, 1834 [30 June]: 203. Type species: *Tenebrio gigas* Linnaeus, 1763, subsequent designation (Spilman 1973: 42). Synonymy: Spilman (1973: 42).

*Cecrops* Gistel, 1834 [23 September]: 21. Type species: *Tenebrio gigas* Linnaeus, 1763, subsequent designation (Bousquet and Bouchard 2017a: 131). Synonymy: Bousquet and Bouchard (2017a: 132).

*Nyctobates* Guérin-Méneville, 1834 [“31 December”]: 33. Type species: *Tenebrio gigas* Linnaeus, 1763, original designation. Synonymy: Ferrer and Siliansky (2007: 186).

***Mylaris gigas* (Linnaeus, 1763)** MEX GUA NIC CRI PAN / SA

*Tenebrio gigas* Linnaeus, 1763: 13.

*Mylaris gigantea* Pallas, 1781: 37. Synonymy: Pallas (1781: 37).

*Tenebrio laminatus* Fabricius, 1787: 211. Synonymy: Fabricius (1801a: 144).

***Mylaris procera* (Champion, 1885)** MEX (SI VE) GUA BEL PAN / SA

*Nyctobates procerus* Champion, 1885: 107.

**Genus *Nesocyrtosoma* Marcuzzi, 1976** [N]

*Nesocyrtosoma* Marcuzzi, 1976: 137. Type species: *Cyrtosoma inflatum* Marcuzzi, 1976, designation of the International Commission on Zoological Nomenclature (ICZN 2017).

*Pachycyrtosoma* Marcuzzi, 1999: 81. Type species: *Cyrtosoma merkli* Marcuzzi, 1999, original designation. Synonymy: Hopp and Ivie (2009: 13).

*Serrania* Garrido, 2003: 50. Type species: *Diaperis viridula* Zayas, 1988 (= *Platydema virens* Laporte and Brullé, 1831), monotypy. Synonymy: Hopp and Ivie (2009: 13).

***Nesocyrtosoma altagracia* Hopp and Ivie, 2009** DOM

*Nesocyrtosoma altagracia* Hopp and Ivie, 2009: 40.

***Nesocyrtosoma bankense* Hopp and Ivie, 2009** PRI LAN

*Nesocyrtosoma bankense* Hopp and Ivie, 2009: 57.

***Nesocyrtosoma basilense* Hopp and Ivie, 2009** HAI

*Nesocyrtosoma basilense* Hopp and Ivie, 2009: 41.

***Nesocyrtosoma bestiola* Hopp and Ivie, 2009** DOM

*Nesocyrtosoma bestiola* Hopp and Ivie, 2009: 20.

***Nesocyrtosoma crenulatum* Hopp and Ivie, 2009** HAI DOM

*Nesocyrtosoma crenulatum* Hopp and Ivie, 2009: 44.

*Nesocyrtosoma bromelicolus* Garrido and Varela, 2010: 33. Synonymy: Hopp (2011: 242).

***Nesocyrtosoma critalense* (Zayas, 1988)** CUB

*Cnodalon critalensis* Zayas, 1988: 99.

***Nesocyrtosoma cubanense* (Kulzer, 1961)** CUB

*Apsida cubanensis* Kulzer, 1961a: 217.

***Nesocyrtosoma cuproso* (Zayas, 1988)** ^83^ CUB

*Cnodalon cuproso* Zayas, 1988: 98.

***Nesocyrtosoma curvum* Hopp and Ivie, 2009** PRI

*Nesocyrtosoma curvum* Hopp and Ivie, 2009: 68.

***Nesocyrtosoma darlingtoni* Hopp and Ivie, 2009** HAI

*Nesocyrtosoma darlingtoni* Hopp and Ivie, 2009: 47.

***Nesocyrtosoma dentatum* Hopp and Ivie, 2009** CUB

*Nesocyrtosoma dentatum* Hopp and Ivie, 2009: 76.

***Nesocyrtosoma dolosum* Hopp and Ivie, 2009** HAI

*Nesocyrtosoma dolosum* Hopp and Ivie, 2009: 42.

***Nesocyrtosoma elongatum* (Zayas, 1988)** CUB

*Cnodalon elongatus* Zayas, 1988: 101.

***Nesocyrtosoma fernandoi* Hopp and Ivie, 2009** CUB

*Nesocyrtosoma fernandoi* Hopp and Ivie, 2009: 59.

***Nesocyrtosoma ferrugineum* (Garrido and Gutiérrez, 1996)** CUB

*Cyrtosoma ferruginea* Garrido and Gutiérrez, 1996c: 282.

***Nesocyrtosoma garridoi* Hopp and Ivie, 2009** CUB

*Nesocyrtosoma garridoi* Hopp and Ivie, 2009: 60.

***Nesocyrtosoma gebieni* (Marcuzzi, 1976)** CUB

*Cyrtosoma gebieni* Marcuzzi, 1976: 139.

*Cnodalon punctatum* Zayas, 1988: 103. Synonymy: Garrido and Gutiérrez (1996c: 282).

***Nesocyrtosoma guerreroi* Hopp and Ivie, 2009** DOM

*Nesocyrtosoma guerreroi* Hopp and Ivie, 2009: 69.

***Nesocyrtosoma hispaniolae* (Marcuzzi, 1999)** DOM

*Cyrtosoma hispaniolae* Marcuzzi, 1999: 83.

***Nesocyrtosoma inflatum* (Marcuzzi, 1976)** CUB

*Cyrtosoma inflatum* Marcuzzi, 1976: 138.

*Cnodalon trinitatis* Zayas, 1988: 102 [junior secondary homonym of *Cyrtosoma trinitatis* Marcuzzi, 1976]. Synonymy: Hopp and Ivie (2009: 31).

*Cyrtosoma iviei* Marcuzzi, 1998a: 160. Replacement name for *Cyrtosoma trinitatis* (Zayas, 1988).

***Nesocyrtosoma lacrima* Hopp and Ivie, 2009** LAN

*Nesocyrtosoma lacrima* Hopp and Ivie, 2009: 36.

***Nesocyrtosoma larseni* Hopp and Ivie, 2009** CUB DOM

*Nesocyrtosoma larseni* Hopp and Ivie, 2009: 50.

***Nesocyrtosoma merkli* (Marcuzzi, 1999)** DOM

*Cyrtosoma merkli* Marcuzzi, 1999: 82.

***Nesocyrtosoma mutabile* Hopp and Ivie, 2009** DOM

*Nesocyrtosoma mutabile* Hopp and Ivie, 2009: 48.

***Nesocyrtosoma nearnsi* Hopp and Ivie, 2009** HAI DOM

*Nesocyrtosoma nearnsi* Hopp and Ivie, 2009: 65.

***Nesocyrtosoma neibaense* Hopp and Ivie, 2009** HAI DOM

*Nesocyrtosoma neibaense* Hopp and Ivie, 2009: 29.

***Nesocyrtosoma otus* Hopp and Ivie, 2009** HAI DOM

*Nesocyrtosoma otus* Hopp and Ivie, 2009: 45.

***Nesocyrtosoma parallelum* (Zayas, 1988)** CUB

*Cnodalon parallelus* Zayas, 1988: 96.

***Nesocyrtosoma productum* Hopp and Ivie, 2009** DOM

*Nesocyrtosoma productum* Hopp and Ivie, 2009: 67.

***Nesocyrtosoma puertoricense* Hopp and Ivie, 2009** PRI

*Nesocyrtosoma puertoricense* Hopp and Ivie, 2009: 61.

***Nesocyrtosoma purpureum* Hopp and Ivie, 2009** DOM

*Nesocyrtosoma purpureum* Hopp and Ivie, 2009: 43.

***Nesocyrtosoma scabrosum* Hopp and Ivie, 2009** HAI

*Nesocyrtosoma scabrosum* Hopp and Ivie, 2009: 35.

***Nesocyrtosoma serratum* Hopp and Ivie, 2009** DOM

*Nesocyrtosoma serratum* Hopp and Ivie, 2009: 62.

***Nesocyrtosoma skelleyi* Hopp and Ivie, 2009** DOM

*Nesocyrtosoma skelleyi* Hopp and Ivie, 2009: 63.

***Nesocyrtosoma simplex* Hopp and Ivie, 2009** HAI DOM

*Nesocyrtosoma simplex* Hopp and Ivie, 2009: 37.

***Nesocyrtosoma teresitae* Hopp and Ivie, 2009** CUB

*Nesocyrtosoma teresitae* Hopp and Ivie, 2009: 67.

***Nesocyrtosoma tumefactum* (Marcuzzi, 1976)** CUB

*Cyrtosoma tumefactum* Marcuzzi, 1976: 138.

*Cnodalon tumefactum* Zayas, 1988: 95 [junior secondary homonym of *Cyrtosoma tumefactum* Marcuzzi, 1976]. Synonymy: Garrido and Gutiérrez (1996c: 282).

*Cnodalon inflatum* Zayas, 1988: 101 [junior secondary homonym of *Cyrtosoma inflatum* Marcuzzi, 1976]. Synonymy: Hopp and Ivie (2009: 24).

*Cyrtosoma zayasi* Marcuzzi, 1998a: 160. Replacement name for *Cyrtosoma tumefactum* (Zayas, 1988).

*Cyrtosoma gundlachi* Marcuzzi, 1998a: 160. Replacement name for *Cyrtosoma inflatum* (Zayas, 1988).

***Nesocyrtosoma turquinense* (Zayas, 1988)** CUB

*Cnodalon turquinensis* Zayas, 1988: 96.

***Nesocyrtosoma virens* (Laporte and Brullé, 1831)** CUB PRI

*Platydema virens* Laporte and Brullé, 1831: 391.

*Hoplocephala flavicornis* Chevrolat, 1877a: 170. Synonymy: Chevrolat (1878d: 243).

*Diaperis viridula* Zayas, 1988: 92. Synonymy: Hopp and Ivie (2009: 71).

**Genus *Nuptis* Motschulsky, 1872** [M]

*Nuptis* Motschulsky, 1872: 25. Type species: *Nuptis tenuis* Motschulsky, 1872, original designation.

***Nuptis caliginosus* Champion, 1885** MEX (VE YU)

*Nuptis caliginosus* Champion, 1885: 109.

***Nuptis cornutus* Champion, 1885** GUA NIC CRI PAN / SA

*Nuptis cornutus* Champion, 1885: 108.

***Nuptis corticalis* Champion, 1885** NIC PAN

*Nuptis corticalis* Champion, 1885: 110.

***Nuptis inquinatus* Champion, 1885** MEX (CI JA) GUA NIC

*Nuptis inquinatus* Champion, 1885: 109.

***Nuptis laticollis* Champion, 1892** PAN

*Nuptis laticollis* Champion, 1892: 523.

***Nuptis tenebrosus* Champion, 1885** MEX (VE) GUA PAN

*Nuptis tenebrosus* Champion, 1885: 110.

***Nuptis tenuis* Motschulsky, 1872** NIC

*Nuptis tenuis* Motschulsky, 1872: 32.

***Nuptis validus* Champion, 1885** MEX (VE) GUA

*Nuptis validus* Champion, 1885: 110.

**Genus *Oeatus* Champion, 1885** [M]

*Oeatus* Champion, 1885: 111. Type species: *Oeatus chevrolati* Champion, 1885, subsequent designation (Gebien 1941: 342).

***Oeatus chevrolati* Champion, 1885** MEX (VE) GUA BEL

*Oeatus chevrolati* Champion, 1885: 111.

***Oeatus similis* Champion, 1892** MEX (CI OA) GUA BEL CRI

*Oeatus similis* Champion, 1892: 523.

**Genus *Oenopion* Champion, 1885** [M]

*Oenopion* Champion, 1885: 98. Type species: *Oenopion gibbosus* Champion, 1885, monotypy.

***Oenopion adeptus* Doyen, 1971** MEX (NL PU)

*Oenopion adeptus* Doyen, 1971: 114.

***Oenopion gibbosus* Champion, 1885** MEX (VE)

*Oenopion gibbosus* Champion, 1885: 99.

***Oenopion zopheroides* (Horn, 1874)** USA (NM TX) MEX (SL)

*Iphthimus zopheroides* Horn, 1874a: 34.

**Genus *Othryoneus* Champion, 1886** [M]

*Othryoneus* Champion, 1886: 245. Type species: *Othryoneus erotyloides* Champion, 1886, subsequent designation (Gebien 1942: 315).

*Gaurobates* Gebien, 1928b: 184. Type species: *Gaurobates pictus* Gebien, 1928, monotypy. Synonymy: Ferrer (2010: 82).

***Othryoneus erotyloides* Champion, 1886** NIC

*Othryoneus erotyloides* Champion, 1886: 246.

***Othryoneus triplehorni* Ferrer and Ødegaard, 2005** PAN

*Othryoneus triplehorni* Ferrer and Ødegaard, 2005: 637.

**Genus *Oxidates* Champion, 1886** [M]

*Oxidates* Champion, 1886: 263. Type species: *Oxidates planicollis* Champion, 1886, subsequent designation (Gebien 1943: 402).

***Oxidates aurichalceus* Champion, 1887** MEX

*Oxidates aurichalceus* Champion, 1887: 265.

***Oxidates elongatus* Champion, 1893** MEX (GE)

*Oxidates elongatus* Champion, 1893a: 548.

***Oxidates gibbus* Champion, 1893** MEX (VE)

*Oxidates gibbus* Champion, 1893a: 548.

***Oxidates gravidus* (Brême, 1842)** MEX (VE)

*Sphoerotus gravidus* Brême, 1842: 109.

***Oxidates mexicanus* (Brême, 1842)** MEX (VE)

*Sphoerotus mexicanus* Brême, 1842: 110.

***Oxidates planicollis* Champion, 1886** MEX (VE)

*Oxidates planicollis* Champion, 1886: 264.

***Oxidates princeps* Champion, 1887** MEX (OA VE)

*Oxidates princeps* Champion, 1887: 265.

***Oxidates puncticeps* Champion, 1887** MEX (VE)

*Oxidates puncticeps* Champion, 1887: 266.

***Oxidates thoracicus* (Brême, 1842)** MEX (VE)

*Sphoerotus thoracicus* Brême, 1842: 110.

**Genus *Polopinus* Casey, 1924** [M]

*Polopinus* Casey, 1924: 326. Type species: *Polypleurus nitidus* LeConte, 1866, original designation.

***Polopinus hubbelli* Kritsky, 1989** USA (FL)

*Polopinus hubbelli* Kritsky, 1989: 132.

***Polopinus ingens* Casey, 1924** USA (FL GA)

*Polopinus ingens* Casey, 1924: 327.

***Polopinus nitidus* (LeConte, 1866)** USA (FL)

*Polypleurus nitidus* LeConte, 1866b: 118.

*Polopinus nitidus subdepressus* Casey, 1924: 327. Synonymy: Kritsky (1989: 128).

*Polopinus nitidus brevior* Casey, 1924: 327. Synonymy: Kritsky (1989: 128).

***Polopinus youngi* Kritsky, 1989** USA (FL)

*Polopinus youngi* Kritsky, 1989: 130.

**Genus *Polypleurus* Eschscholtz, 1831** [M]

*Polypleurus* Eschscholtz, 1831: 10, 11. Type species: *Polypleurus geminatus* Eschscholtz, 1831, monotypy.

***Polypleurus geminatus* Eschscholtz, 1831** USA (FL GA)

*Polypleurus geminatus* Eschscholtz, 1831: 11.

***Polypleurus perforatus* (Germar, 1823)** USA (AL AR FL GA IL LA MD MO MS NC NJ OK PA SC TX VA WV)

*Upis perforata* Germar, 1823: 148.

*Polypleurus punctatus* Solier, 1838: 197. Synonymy: LeConte (1866a: 61).

**Genus *Saziches* Champion, 1886** [M]

*Saziches* Champion, 1886: 261. Type species: *Saziches subcaudatus* Champion, 1886, monotypy.

***Saziches giesberti* Doyen, 1988** CRI

*Saziches giesberti* Doyen, 1988: 310.

***Saziches subcaudatus* Champion, 1886** GUA

*Saziches subcaudatus* Champion, 1886: 262.

**Genus *Sthenoboea* Champion, 1885** [F]

*Sthenoboea* Champion, 1885: 112. Type species: *Sthenoboea apicalis* Champion, 1885, monotypy.

***Sthenoboea apicalis* Champion, 1885** MEX

*Sthenoboea apicalis* Champion, 1885: 113.

**Genus *Upis* Fabricius, 1792** [M]

*Upis* Fabricius, 1792b: 515. Type species: *Attelabus ceramboides* Linnaeus, 1758, monotypy.

**Figure 41.**
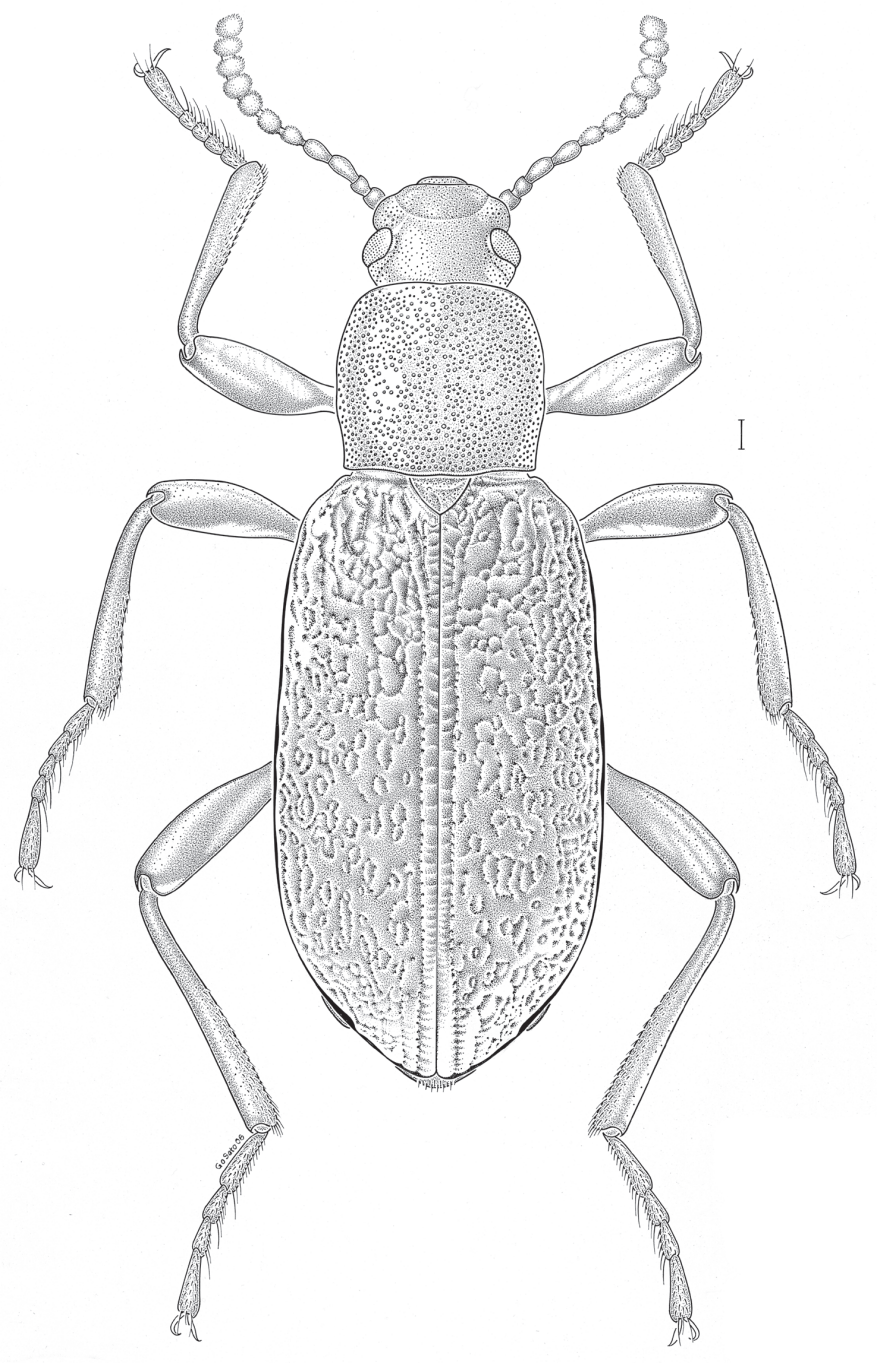
*Upis ceramboides* (Linnaeus, 1758). Scale bar = 1 mm.

***Upis ceramboides* (Linnaeus, 1758)** [Fig. 41] CAN (AB BC MB NB NF NS NT ON QC PE SK YT) USA (AK ID ME MI NH NY OH OR PA SD VT WA WI WY) – Holarctic

*Attelabus ceramboides* Linnaeus, 1758: 388.

*Tenebrio variolosus* DeGeer, 1775: 32. Synonymy: DeGeer (1775: 32).

*Tenebrio reticulatus* Say, 1824b: 279. Synonymy: Dejean (1834: 204).

**Genus *Xenius* Champion, 1886** [M]

*Xenius* Champion, 1886: 224. Type species: *Xenius scabripennis* Champion, 1886, monotypy.

***Xenius scabripennis* Champion, 1886** NIC PAN

*Xenius scabripennis* Champion, 1886: 224.

**Genus *Xylopinus* LeConte, 1862** [M]

*Xylopinus* LeConte, 1862a: 230. Type species: *Tenebrio anthracinus* Knoch, 1801 (= *Tenebrio saperdoides* Olivier, 1795), subsequent designation (Gebien 1941: 336).

*Taenobates* Motschulsky, 1872: 25. Type species: *Tenebrio saperdoides* Olivier, 1795, original designation. Synonymy: C.O. Waterhouse (1876: 288).

***Xylopinus aenescens* LeConte, 1866** CAN (NB ON QC) USA (AL CT DC FL GA IA IN LA MA MD MI NC NJ NY OH PA RI SC TN VA WI)

*Xylopinus aenescens* LeConte, 1866b: 120.

***Xylopinus saperdoides* (Olivier, 1795)** CAN (NB NS ON QC) USA (AL AR CT DC DE FL GA IA IL IN KS LA MD MI MN MO MS NC NH NY OH OK PA SC TN TX VA VT WI WV)

*Tenebrio saperdoides* Olivier, 1795: [57] 11.

*Helops spinipes* Fabricius, 1798: 53. Synonymy: Illiger (1802: 344).

*Tenebrio anthracinus* Knoch, 1801: 169. Synonymy (with *H. spinipes* Fabricius): Illiger (1802: 344).

*Tenebrio rufipes* Say, 1825: 203. Synonymy: Melsheimer (1853: 139).

**Tribe Stenochiini Kirby, 1837**

Stenochiadae Kirby, 1837: 238. Type genus: *Stenochia* Kirby, 1819 (= *Strongylium* Kirby, 1819).

Strongyliides Lacordaire, 1859: 478. Type genus: *Strongylium* Kirby, 1819.

**Genus *Cuphotes* Champion, 1887** [M]

*Spheniscus* Kirby, 1819: 421 [junior homonym of *Spheniscus* Moehring, 1758]. Type species: *Spheniscus erotyloides* Kirby, 1819, monotypy.

*Cuphotes* Champion, 1887: 332. Replacement name for *Spheniscus* Kirby, 1819.

*Phygoscotus* Schulz, 1902: 134. Replacement name for *Spheniscus* Kirby, 1819.

***Cuphotes cinctus* (Olivier, 1795)** NIC CRI PAN / SA

*Helops cinctus* Olivier, 1795 [58]: 13.

*Erotylus unifasciatus* Fabricius, 1798: 101. Synonymy: Chevrolat (1843: 80).

*Spheniscus 4maculatus* Erichson, 1847: 120. Synonymy: Champion (1887: 334).

*Spheniscus 4-plagiatus* Kirsch, 1866: 202. Synonymy: Champion (1887: 334).

***Cuphotes corallifer* (J. Thomson, 1859)** PAN / SA

*Spheniscus corallifer* J. Thomson, 1859: 108.

***Cuphotes elongatus* (J. Thomson, 1859)** NIC PAN / SA

*Spheniscus elongatus* J. Thomson, 1859: 112.

***Cuphotes jansoni* Champion, 1887** NIC

*Cuphotes jansoni* Champion, 1887: 333.

***Cuphotes multimaculatus* Pic, 1918** CRI

*Cuphotes multimaculatus* Pic, 1918b: 7.

***Cuphotes nigromaculatus marginicollis* (J. Thomson, 1859)** MEX GUA

*Spheniscus marginicollis* J. Thomson, 1859: 110.

***Cuphotes nigromaculatus nigromaculatus* (J. Thomson, 1859)** MEX (JA VE) GUA NIC CRI PAN

*Spheniscus nigro-maculatus* J. Thomson, 1859: 110.

***Cuphotes unicolor* Champion, 1887** NIC

*Cuphotes unicolor* Champion, 1887: 334.

**Genus *Mentes* Champion, 1893** [M]

*Mentes* Champion, 1893a: 559. Type species: *Mentes ruficollis* Champion, 1893, subsequent designation (Lucas 1920: 404).

***Mentes aeneopiceus* Champion, 1896** LAN

*Mentes aeneopiceus* Champion, 1896: 30.

***Mentes cisteloides* Doyen, 1990** MEX (JA)

*Mentes cisteloides* Doyen, 1990: 254.

***Mentes fusiformis* Champion, 1893** GUA

*Mentes fusiformis* Champion, 1893a: 560.

***Mentes ruficollis* Champion, 1893** PAN

*Mentes ruficollis* Champion, 1893a: 559.

***Mentes setipennis* Champion, 1893** GUA

*Mentes setipennis* Champion, 1893a: 560.

**Genus *Oploptera* Chevrolat, 1844** [F]

*Oploptera* Chevrolat [in Guérin-Méneville], 1844: 126. Type species: *Strongylium serraticorne* Guérin-Méneville, 1834, monotypy.

*Otocerus* Mäklin, 1867: 484. Unnecessary replacement name for *Oploptera* Chevrolat, 1844.

*Hoploptera* Gemminger [in Gemminger and Harold], 1870: 2037. Unjustified emendation of *Oploptera* Chevrolat, 1844, not in prevailing usage.

**Subgenus Oploptera Chevrolat, 1844** [F]

*Oploptera* Chevrolat [in Guérin-Méneville], 1844: 126. Type species: *Strongylium serraticorne* Guérin-Méneville, 1834, monotypy.

***Oploptera angelicae* (Ferrer and Ødegaard, 2005)** PAN

*Otocerus angelicae* Ferrer and Ødegaard, 2005: 644.

***Oploptera chamelensis* (Doyen, 1990)** MEX (JA)

*Otocerus chamelensis* Doyen, 1990: 256.

***Oploptera delicata* (Ferrer and Ødegaard, 2005)** PAN

*Otocerus delicatus* Ferrer and Ødegaard, 2005: 649.

***Oploptera dilaticornis* (Champion, 1888)** PAN

*Otocerus dilaticornis* Champion, 1888: 378.

***Oploptera hamata* (Champion, 1888)** NIC

*Otocerus hamatus* Champion, 1888: 381.

***Oploptera interrupta* (Champion, 1888)** PAN

*Otocerus interruptus* Champion, 1888: 380.

***Oploptera microps* (Champion, 1888)** NIC

*Otocerus microps* Champion, 1888: 381.

***Oploptera nicaraguensis* (Champion, 1888)** NIC

*Otocerus nicaraguensis* Champion, 1888: 379.

***Oploptera torolae* (Champion, 1888)** GUA

*Otocerus torolae* Champion, 1888: 378.

**Subgenus Plicatocerus Pic, 1918**

*Plicatocerus* Pic, 1918b: 11. Type species: *Otocerus impressipennis* Champion, 1888, monotypy.

***Oploptera impressipennis* (Champion, 1888)** PAN

*Otocerus impressipennis* Champion, 1888: 382.

**Genus *Poecilesthus* Dejean, 1834** [M]

*Poecilesthus* Dejean, 1834: 207. Type species: *Erotylus fasciatus* Fabricius, 1781, subsequent designation (Hope 1841: 133).

*Diestica* Pascoe, 1868: xii. Type species: *Diestica viridipennis* Pascoe, 1868, monotypy. Synonymy: Gebien (1911b: 589).

***Poecilesthus cupripennis* Champion, 1893** PAN

*Poecilesthus cupripennis* Champion, 1893a: 562.

***Poecilesthus fragilicornis* Champion, 1887** CRI PAN

*Poecilesthus fragilicornis* Champion, 1887: 338.

***Poecilesthus guatemalensis* Champion, 1887** GUA

*Poecilesthus guatemalensis* Champion, 1887: 339.

***Poecilesthus immaculatus* Champion, 1887** PAN

*Poecilesthus immaculatus* Champion, 1887: 340.

***Poecilesthus laeviceps* Champion, 1887** PAN

*Poecilesthus laeviceps* Champion, 1887: 340.

***Poecilesthus laticollis* Champion, 1887** MEX (CI VE) GUA

*Poecilesthus laticollis* Champion, 1887: 339.

***Poecilesthus latus* Champion, 1887** NIC PAN

*Poecilesthus latus* Champion, 1887: 338.

***Poecilesthus maklini* Champion, 1887** GUA

*Poecilesthus mäklini* Champion, 1887: 341.

***Poecilesthus nigropunctatus* Champion, 1887** MEX PAN / SA

*Poecilesthus nigro-punctatus* Champion, 1887: 336.

***Poecilesthus variipes* Champion, 1887** NIC PAN

*Poecilesthus variipes* Champion, 1887: 337.

**Genus *Pseudotocerus* Champion, 1888** [M]

*Pseudotocerus* Champion, 1888: 383. Type species: *Stenochia longipes* Lucas, 1859, subsequent designation (Gebien 1948: 542).

***Pseudotocerus attenuatus* Champion, 1888** NIC

*Pseudotocerus attenuatus* Champion, 1888: 383^84^.

**Genus *Strongylium* Kirby, 1819** [N]

*Strongylium* Kirby, 1819: 417. Type species: *Strongylium chalconatum* Kirby, 1819, monotypy.

*Stenochia* Kirby, 1819: 423. Type species: *Stenochia rufipes* Kirby, 1819, subsequent designation (Hope 1841: 133). Synonymy: Latreille (1829b: 683).

*Gentinadis* Laporte, 1840: 240. Type species: *Stenochia caerulea* Laporte, 1840 (= *Helops azureus* Germar, 1823), monotypy. Synonymy: Lacordaire (1859: 484).

*Saerangodes* Sturm, 1843: 163. Type species: *Helops interpunctatus* Germar, 1823, monotypy. Synonymy (with *Stenochia* Kirby): Blanchard (1845: 33).

*Reminius* Casey, 1924: 321. Type species: *Reminius ocularis* Casey, 1924 (= *Tenebrio terminatus* Say, 1824), original designation. Synonymy: Spilman (1959: 63).

***Strongylium acraeum* Garrido and Armas, 2012** PRI

*Strongylium acraeum* Garrido and Armas, 2012b: 76.

***Strongylium amethystinum* (Guérin-Méneville, 1838)** CUB

*Stenochia amethystina* Guérin-Méneville [in Guérin-Méneville and Chevrolat], 1838: 281.

***Strongylium angustulum* Mäklin, 1867** PAN / SA

*Strongylium angustulum* Mäklin, 1867: 314.

***Strongylium antennale* Mäklin, 1867** CUB

*Strongylium antennale* Mäklin, 1867: 270.

***Strongylium anthrax* Schwarz, 1878** USA (FL)

*Strongylium anthrax* Schwarz, 1878: 369.

***Strongylium apache* Triplehorn and Spilman, 1973** USA (AZ NM) MEX [SO]

*Strongylium apache* Triplehorn and Spilman, 1973: 10.

***Strongylium apicicorne* Mäklin, 1867** MEX (VE)

*Strongylium apicicorne* Mäklin, 1867: 324.

***Strongylium armatum* Mäklin, 1867** MEX (CI VE) GUA PAN

*Strongylium armatum* Mäklin, 1867: 311.

***Strongylium atrum* Champion, 1888** USA (AZ NM) MEX (CH DU SI)

*Strongylium atrum* Champion, 1888: 360.

***Strongylium aulicum* Mäklin, 1867** USA (FL TX) MEX (OA VE) GUA NIC PAN

*Strongylium aulicum* Mäklin, 1867: 363.

***Strongylium auratum* (Laporte, 1840)** MEX (CI GE PU VE YU) GUA BEL NIC CRI PAN / SA

*Stenochia aurata* Laporte, 1840: 240.

Stenochia auratum var. hilaris Mäklin, 1867: 402. Synonymy: Champion (1888: 360).

***Strongylium azureum* (Germar, 1823)** CUB / SA

*Helops azureus* Germar, 1823: 153.

*Stenochia caerulea* Laporte, 1840: 240. Synonymy: Mäklin (1867: 404).

***Strongylium baetianum* Garrido and Armas, 2012** HAI

*Strongylium baetianum* Garrido and Armas, 2012a: 64.

***Strongylium basiclavis* Zayas, 1988** CUB

*Strongylium basiclavis* Zayas, 1988: 110.

***Strongylium belti* Champion, 1888** NIC

*Strongylium belti* Champion, 1888: 358.

***Strongylium bivittatum* Champion, 1888** MEX (OA)

*Strongylium bivittatum* Champion, 1888: 361.

***Strongylium blandum* Mäklin, 1867** MEX (VE)

*Strongylium blandum* Mäklin, 1867: 341.

***Strongylium brevipes* Champion, 1888** NIC PAN

*Strongylium brevipes* Champion, 1888: 372.

***Strongylium canaliculatum* Champion, 1887** MEX GUA

*Strongylium canaliculatum* Champion, 1887: 346.

***Strongylium cancellatum* Mäklin, 1867** MEX (VE) BEL

*Strongylium cancellatum* Mäklin, 1867: 320.

***Strongylium carinipenne* Champion, 1888** PAN

*Strongylium carinipenne* Champion, 1888: 374.

***Strongylium chalcopterum* Mäklin, 1867** LAN (Martinique)

*Strongylium chalcopterum* Mäklin, 1867: 431.

***Strongylium championi* Gebien, 1948** USA (TX) MEX (JA VE) GUA BEL

*Strongylium varians* Champion, 1888: 365 [junior secondary homonym of *Strongylium varians* (Pascoe, 1883)].

*Strongylium championi* Gebien, 1948: 532. Replacement name for *Strongylium varians* Champion, 1888.

***Strongylium chiriquense* Champion, 1887** PAN

*Strongylium chiriquense* Champion, 1887: 351.

***Strongylium chontalense* Champion, 1887** NIC

*Strongylium chontalense* Champion, 1887: 344.

***Strongylium cinctum* Mäklin, 1867** MEX (VE)

*Strongylium cinctum* Mäklin, 1867: 337.

***Strongylium clavicorne* Champion, 1893** MEX (VE)

*Strongylium clavicorne* Champion, 1893a: 562.

***Strongylium colombianum* Champion, 1888** PAN / SA

*Strongylium colombianum* Champion, 1888: 354.

***Strongylium conicicolle* Mäklin, 1867** NIC CRI PAN

*Strongylium conicicolle* Mäklin, 1867: 447.

***Strongylium conradti* Champion, 1893** GUA

*Strongylium conradti* Champion, 1893a: 563.

***Strongylium costaricense* Champion, 1888** CRI

*Strongylium costaricense* Champion, 1888: 353.

***Strongylium crassicorne* Champion, 1887** NIC

*Strongylium crassicorne* Champion, 1887: 347.

***Strongylium crenatum* Mäklin, 1867** USA (AL AR FL GA IA KS LA MD MO MS NC OH OK SC TN TX VA)

*Strongylium crenatum* Mäklin, 1867: 307.

***Strongylium cribripes* Mäklin, 1867** MEX (VE) NIC PAN

*Strongylium cribripes* Mäklin, 1867: 275.

***Strongylium cruentatum* Mäklin, 1867** MEX (VE)

*Strongylium cruentatum* Mäklin, 1867: 335.

***Strongylium cultellatum* Mäklin, 1867** USA (FL) – Adventive

*Strongylium cultellatum* Mäklin, 1867: 453.

***Strongylium cupeyal* Zayas, 1988** CUB

*Strongylium cupeyal* Zayas, 1988: 110.

***Strongylium cuproso* Garrido, 2004** CUB

*Strongylium cuproso* Garrido, 2004d: 52.

***Strongylium curticorne* Champion, 1888** MEX (CI)

*Strongylium curticorne* Champion, 1888: 369.

***Strongylium decoratum* Mäklin, 1867** NIC CRI PAN / SA

*Strongylium decoratum* Mäklin, 1867: 365.

***Strongylium delauneyi* Fleutiaux and Sallé, 1890** LAN

*Strongylium delauneyi* Fleutiaux and Sallé, 1890: 429.

***Strongylium dentatum* Champion, 1887** NIC

*Strongylium dentatum* Champion, 1887: 348.

***Strongylium discoidale* Mäklin, 1867** MEX (VE)

*Strongylium discoidale* Mäklin, 1867: 339.

***Strongylium elongatum* Garrido and Armas, 2012** DOM

*Strongylium elongatum* Garrido and Armas, 2012a: 65.

***Strongylium eminens* Mäklin, 1867** MEX (JA VE)

*Strongylium eminens* Mäklin, 1867: 374.

***Strongylium erraticum* Champion, 1888** NIC

*Strongylium erraticum* Champion, 1888: 373.

***Strongylium exaratum* Champion, 1887** GUA PAN

*Strongylium exaratum* Champion, 1887: 350.

***Strongylium excavatum* Mäklin, 1867** MEX (OA VE) GUA NIC PAN

*Strongylium excavatum* Mäklin, 1867: 274.

***Strongylium eximium* Mäklin, 1867** CUB

*Strongylium eximium* Mäklin, 1867: 269.

***Strongylium fossifrons* Mäklin, 1867** PAN / SA

*Strongylium fossifrons* Mäklin, 1867: 285.

***Strongylium fragile* Champion, 1888** PAN

*Strongylium fragile* Champion, 1888: 377.

***Strongylium frontale* Champion, 1888** PAN

*Strongylium frontale* Champion, 1888: 357.

***Strongylium funestum* Mäklin, 1867** MEX

*Strongylium funestum* Mäklin, 1867: 295.

***Strongylium gerstaeckeri* Mäklin, 1867** MEX (VE) GUA NIC CRI PAN

*Strongylium gerstaeckeri* Mäklin, 1867: 277.

***Strongylium gibbum* Mäklin, 1867** MEX GUA

*Strongylium gibbum* Mäklin, 1867: 252.

***Strongylium gregarium* Champion, 1888** PAN

*Strongylium gregarium* Champion, 1888: 373.

***Strongylium guadeloupense* Gebien, 1911** LAN (Guadeloupe)

*Strongylium inaequale* Fleutiaux and Sallé, 1890: 430 [junior primary homonym of *Strongylium inaequale* Mäklin, 1867].

*Strongylium guadeloupense* Gebien, 1911b: 596. Replacement name for *Strongylium inaequale* Fleutiaux and Sallé, 1890.

***Strongylium hemistriatum* Triplehorn and Spilman, 1973** USA (TX)

*Strongylium hemistriatum* Triplehorn and Spilman, 1973: 20.

***Strongylium hoepfneri chevrolatii* Mäklin, 1867** MEX

*Strongylium chevrolatii* Mäklin, 1867: 235.

***Strongylium hoepfneri hoepfneri* Mäklin, 1867** MEX (VE) GUA

*Strongylium hoepfneri* Mäklin, 1867: 232.

***Strongylium hoepfneri immundum* Mäklin, 1867** MEX

*Strongylium immundum* Mäklin, 1867: 234.

***Strongylium hoepfneri pectorale* Mäklin, 1867** MEX NIC

*Strongylium pectorale* Mäklin, 1867: 233.

***Strongylium hoepfneri scutellare* Mäklin, 1867** MEX

*Strongylium scutellare* Mäklin, 1867: 233.

***Strongylium ignitum* Champion, 1887** NIC PAN

*Strongylium ignitum* Champion, 1887: 348.

***Strongylium impressicolle* Mäklin, 1867** MEX (DU JA VE) GUA NIC CRI

*Strongylium impressicolle* Mäklin, 1867: 301.

***Strongylium languidum* Mäklin, 1867** MEX (VE) GUA

*Strongylium languidum* Mäklin, 1867: 312.

***Strongylium langurioides* Champion, 1888** NIC

*Strongylium langurioides* Champion, 1888: 355.

***Strongylium laterale* Mäklin, 1867** MEX (VE)

*Strongylium laterale* Mäklin, 1867: 334.

***Strongylium limitatum* Mäklin, 1867** MEX (VE)

*Strongylium limitatum* Mäklin, 1867: 342.

***Strongylium lucidum* Mäklin, 1867** CRI

*Strongylium lucidum* Mäklin, 1867: 283.

***Strongylium maculicolle* Champion, 1887** NIC CRI PAN

*Strongylium maculicolle* Champion, 1887: 342.

***Strongylium maisi* Garrido, 2004** CUB

*Strongylium maisi* Garrido, 2004d: 52.

***Strongylium marginale* Mäklin, 1867** MEX (VE)

*Strongylium marginale* Mäklin, 1867: 338.

***Strongylium misantlae* Champion, 1888** MEX (OA VE) GUA

*Strongylium misantlae* Champion, 1888: 367.

***Strongylium montebarreto* Garrido, 2004** CUB

*Strongylium montebarreto* Garrido, 2004d: 50.

***Strongylium nigrum* Zayas, 1988** CUB

*Strongylium nigra* Zayas, 1988: 110.

***Strongylium nitidiceps* Champion, 1888** MEX (VE)

*Strongylium nitidiceps* Champion, 1888: 364.

***Strongylium nubeculosum* Mäklin, 1867** MEX (YU) NIC

*Strongylium nubeculosum* Mäklin, 1867: 336.

***Strongylium oculatum* Champion, 1888** GUA NIC

*Strongylium oculatum* Champion, 1888: 371.

***Strongylium opacipenne* Champion, 1888** MEX (VE)

*Strongylium opacipenne* Champion, 1888: 361.

***Strongylium paddai* Ivie and Triplehorn, 1986** VIS

*Strongylium paddai* Ivie and Triplehorn, 1986: 423.

***Strongylium panamense* Champion, 1888** PAN

*Strongylium panamense* Champion, 1888: 363.

***Strongylium permodicum* Mäklin, 1867** GUA NIC PAN / LAN / SA

*Strongylium permodicum* Mäklin, 1867: 320.

***Strongylium preciosus* Zayas, 1988^85^** CUB

*Strongylium preciosus* Zayas, 1988: 108.

***Strongylium pulvinatum* Mäklin, 1867** PRI

*Strongylium pulvinatum* Mäklin, 1867: 265.

***Strongylium pumilum* Garrido and Armas, 2012** PRI

*Strongylium pumilum* Garrido and Armas, 2012b: 73.

***Strongylium punctifrons* Mäklin, 1867** MEX (CI VE) BEL

*Strongylium punctifrons* Mäklin, 1867: 296.

***Strongylium punctipes* Champion, 1888** MEX (JA) GUA

*Strongylium punctipes* Champion, 1888: 375.

***Strongylium quisqueyanum* Garrido and Armas, 2012** DOM

*Strongylium quisqueyanum* Garrido and Armas, 2012a: 66.

***Strongylium ramosum* Mäklin, 1867** MEX (VE)

*Strongylium ramosum* Mäklin, 1867: 340.

***Strongylium sallei* Mäklin, 1867** MEX (VE) GUA NIC

*Strongylium sallei* Mäklin, 1867: 257.

*Strongylium sallaei* Champion, 1887: 345. Unjustified emendation of *Strongylium sallei* Mäklin, 1867, not in prevailing usage.

***Strongylium semistriatum* Mäklin, 1867** MEX (VE)

*Strongylium semistriatum* Mäklin, 1867: 251.

***Strongylium simplicipes* Pic, 1918** PAN

*Strongylium simplicipes* Pic, 1918b: 15.

***Strongylium simplicicolle* LeConte, 1878** USA (AL FL GA MS NC SC TN VA)

*Strongylium simplicicolle* LeConte, 1878a: 424.

***Strongylium subcostatum* Mäklin, 1867** MEX GUA

*Strongylium subcostatum* Mäklin, 1867: 316.

***Strongylium suturale* Mäklin, 1867** MEX (VE) GUA

*Strongylium suturale* Mäklin, 1867: 337.

**Figure 42.**
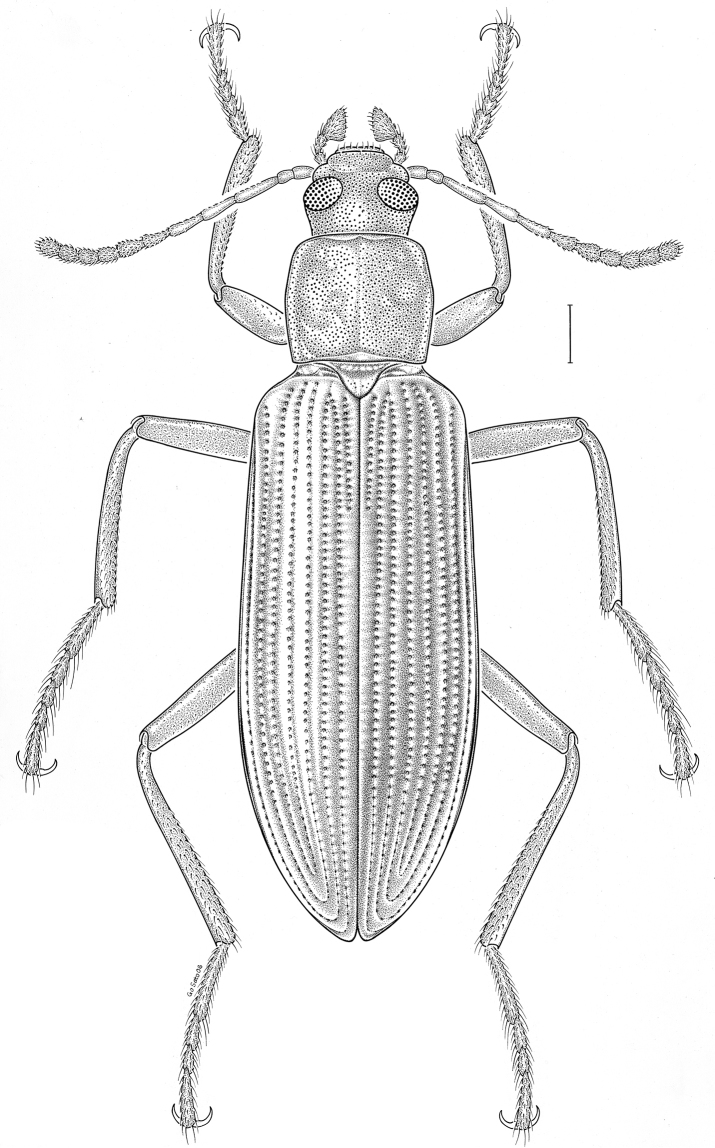
*Strongylium tenuicolle* (Say, 1826). Scale bar = 1 mm.

***Strongylium tenuicolle* (Say, 1826)** [Fig. 42] CAN (MB ON QC) USA (AL AR CO CT DC DE FL GA IA IL IN KS KY LA MA MD MI MN MO MS NC NH NJ NY OH OK PA RI SC SD TN TX VA WI WV)

*Helops tenuicollis* Say, 1826: 241.

***Strongylium terminatum* (Say, 1824)** USA (AL DC FL IA IL IN KS KY LA MD MI MO MS NC NE NJ NY OH OK PA SC SD TX VA WI)

*Tenebrio terminatus* Say, 1824a: 267.

*Reminius ocularis* Casey, 1924: 322. Synonymy: Spilman (1959: 63).

***Strongylium tinctipes* Champion, 1887** NIC PAN

*Strongylium tinctipes* Champion, 1887: 349.

***Strongylium turquinense* Zayas, 1988** CUB

*Strongylium turquinensis* Zayas, 1988: 107.

***Strongylium variicorne* Champion, 1887** PAN

*Strongylium variicorne* Champion, 1887: 352.

***Strongylium ventrale* Champion, 1888** PAN

*Strongylium ventrale* Champion, 1888: 356.

***Strongylium venustum* Zayas, 1988** CUB

*Strongylium venusta* Zayas, 1988: 109.

***Strongylium verde* Garrido and Armas, 2012** PRI

*Strongylium verde* Garrido and Armas, 2012b: 74.

***Strongylium vikenae* Ferrer and Ødegaard, 2005** PAN

*Strongylium vikenae* Ferrer and Ødegaard, 2005: 644.

***Strongylium virescens* Zayas, 1988** CUB

*Strongylium virescens* Zayas, 1988: 110.

***Strongylium viridipes* Mäklin, 1867** MEX (PU VE)

*Strongylium viridipes* Mäklin, 1867: 274.

***Strongylium viriditinctum* Champion, 1888** GUA

*Strongylium viriditinctum* Champion, 1888: 359.

***Strongylium woodruffi* Garrido and Armas, 2012** DOM

*Strongylium woodruffi* Garrido and Armas, 2012a: 67.

**Tribe Talanini Champion, 1887**

Dignamptini LeConte and Horn, 1883: 385. Type genus: *Dignamptus* LeConte, 1878 (= *Talanus* Jacquelin du Val, 1857).

Talanides Champion, 1887: 321. Type genus: *Talanus* Jacquelin du Val, 1857. Note. Family-group name conserved over Dignamptini (see Bouchard et al. 2005).

**Genus *Talanus* Jacquelin du Val, 1857** [M]

*Talanus* Jacquelin du Val, 1857: 156. Type species: *Talanus cribrarius* Jacquelin du Val, 1857, monotypy.

*Dignamptus* LeConte, 1878a: 421. Type species: *Dignamptus stenochinus* LeConte, 1878, **present designation**. Synonymy: Champion (1887: 321).

***Talanus aeneipennis* Champion, 1887** MEX (TB VE) BEL

*Talanus aeneipennis* Champion, 1887: 327.

***Talanus apterus* Champion, 1887** GUA PAN

*Talanus apterus* Champion, 1887: 328.

***Talanus ater* Champion, 1887** PAN

*Talanus ater* Champion, 1887: 327.

***Talanus columbianus* Mäklin, 1878** PAN / SA

*Talanus columbianus* Mäklin, 1878a: 99.

***Talanus cribrarius* Jacquelin du Val, 1857** CUB

*Talanus cribrarius* Jacquelin du Val, 1857: 156.

***Talanus ferrugineus* Champion, 1896** LAN

*Talanus ferrugineus* Champion, 1896: 31.

***Talanus guadeloupensis* Fleutiaux and Sallé, 1890** LAN

*Talanus guadeloupensis* Fleutiaux and Sallé, 1890: 430.

***Talanus guatemalensis* Champion, 1887** GUA

*Talanus guatemalensis* Champion, 1887: 326.

***Talanus insularis* Mäklin, 1878** PRI LAN

*Talanus insularis* Mäklin, 1878a: 98.

***Talanus interstitialis* Champion, 1887** ME (CI JA) GUA NIC

*Talanus interstitialis* Champion, 1887: 324.

***Talanus laevicollis* Champion, 1896** LAN

*Talanus laevicollis* Champion, 1896: 32.

***Talanus laevipennis* Champion, 1887** GUA

*Talanus laevipennis* Champion, 1887: 322.

***Talanus langurinus* (LeConte, 1878)** USA (FL TX)

*Dignamptus langurinus* LeConte, 1878a: 421.

***Talanus laticeps* Champion, 1887** PAN

*Talanus laticeps* Champion, 1887: 325.

***Talanus lecontei* Champion, 1887** MEX (TB VE YU) GUA BEL

*Talanus lecontei* Champion, 1887: 323.

***Talanus longicornis* Champion, 1887** GUA BEL NIC PAN

*Talanus longicornis* Champion, 1887: 328.

***Talanus mecoscelis* Triplehorn, 1968** USA (TX)

*Talanus mecoscelis* Triplehorn, 1968a: 33.

***Talanus neotropicalis* Champion, 1887** MEX (JA VE YU) GUA CRI PAN / SA

*Talanus neotropicalis* Champion, 1887: 322.

***Talanus spilmani* Triplehorn, 1968** USA (FL)

*Talanus spilmani* Triplehorn, 1968a: 35.

***Talanus stenochinus* (LeConte, 1878)** USA (FL LA)

*Dignamptus stenochinus* LeConte, 1878a: 421.

*Talanus okeechobensis* Blatchley, 1914: 143. Synonymy: Fall (1932: 148).

***Talanus subexaratus* Mäklin, 1878** MEX (CI GE SI VE) GUA BEL NIC CRI PAN / SA

*Talanus subexaratus* Mäklin, 1878a: 102.

***Talanus subopacus* Champion, 1887** MEX (VE) BEL

*Talanus subopacus* Champion, 1887: 323.

***Talanus victori* Garrido and Gutiérrez, 2004** PRI

*Talanus victori* Garrido and Gutiérrez, 2004: 63.

Incertae sedis: Tenebrionidae

***Tenebrio calculensis* Scudder, 1895** CAN (ON)

*Tenebrio calculensis* Scudder, 1895: 31^86^.

## Footnotes

^2^ The name derives from the Greek *Lepidos* (scale) and the generic name *Cnemeplatia* Costa. The gender is feminine.

^3^ All geographical units are provided using their English names as listed in the third edition of Merriam-Webster’s Geographical Dictionary (2001).

^4^ *Adelonia sulcatus* used by Leng and Mutchler (1914: 464) and often listed as a synonym of this species is an incorrect subsequent spelling not in prevailing usage.

^5^ Perty (1832) used two spellings for this name: *Goniadera* (p. 62) and *Gonyodera* (p. 63). Later Perty (1833: 14) used the spelling *Goniadera*. As so he is deemed to be the First Reviser (ICZN 1999: Article 24.2.4) and *Goniadera* is the correct original spelling..

^6^ In their revision of the genus, Ferrer and Delatour (2007: 287) reported this species and the type species of the subgenus as “*Goniadera tuberculatum* Gebien, 1928” and “*G. binotatum* Gebien, 1928” respectively. Both names are *nomina nuda*.

^7^ Mälkin was Finnish, not German. Therefore the umlaut is deleted and no “e” is to be inserted after the “a”.

^8^ This genus was transferred from the Mycetophagidae to the Lagriinae by Lawrence and Newton (1995: 886).

^9^ *Nilio* and *Nilio lebasi* are incorrect subsequent spellings in prevailing usage and attributed to the publication of the original spelling; therefore they are to be preserved and deemed to be the correct original spellings (ICZN 1999: Article 33.3.1).

^10^ The species-group name *bifurcus* is a name in apposition and therefore need not agree in gender with the generic name (M.A. Alonso-Zarazaga personal communication).

^11^ Described from Chile, which is probably a mistake (Champion 1884: 54).

^11^ A junior secondary homonym replaced before 1961 is permanently invalid unless the substitute name is not in use and the relevant taxa are no longer considered congeneric (ICZN 1999: Article 59.3); in this case, the relevant taxa are no longer considered congeneric and the substitute name has not been used subsequently as valid as far as we know.

^13^ A junior secondary homonym replaced before 1961 is permanently invalid unless the substitute name is not in use and the relevant taxa are no longer considered congeneric (ICZN 1999: Article 59.3); in this case, the relevant taxa are no longer considered congeneric and the substitute name is not currently in use.

^14^ Described from the La Brea tar pits, but not actually a fossil [ADS]

^15^ A junior secondary homonym replaced before 1961 is permanently invalid unless the substitute name is not in use and the relevant taxa are no longer considered congeneric (ICZN 1999: Article 59.3); in this case, the relevant taxa are no longer considered congeneric and the substitute name has not been used subsequently as valid as far as we know.

^16^ Casey’s specimens are mislabelled from Texas.

^17^ “*Branchus cubensis* Marcuzzi” has been cited twice by Marcuzzi (1998a: 153) and Peck (2005: 146) but the name was never proposed by anyone and is a *nomen nudum*.

^18^ Described from La Brea tar pits but thought to represent a modern undescribed species by Doyen and Miller (1980: 3).

^19^ Champion (1892: 510, 511) used two different spellings for this species: *obliteratus* (p. 510) and *obsoletus* (p. 511). Doyen (1984b: 41) acted as First Reviser (ICZN 1999: Article 24.2) and selected *obliteratus* as the correct spelling.

^20^ Since the dates of publication of *C. asperata* (Horn, 1870) and *C. asperata* (Motschulsky, 1870) could not be determined besides the year, Aalbu et al. (1995: 482) acted as First Revisers (ICZN 1999: Article 24.2) and selected Horn’s name to have precedence. However, evidence gathered recently [YB] indicate that Motschulsky’s paper in the *Bulletin de la Société Impériale des Naturalistes de Moscou* was published before Horn’s paper in the *Transactions of the American Philosophical Society*. At this point, we prefer not to make any change.

^21^ *Stibia opaca* credited to Blaisdell (1925b: 329), listed as a synonym of *S. cribrata* by Papp (1961d: 103), is considered here as a lapsus for *S. cribrata* Blaisdell.

^22^ *Telabis* is neither Latin or Greek and since it was proposed as a subgenus, it was not placed in agreement with any specific names. Therefore, by application of Article 30.2.4 (ICZN 1999) the name has to be treated as masculine (M.A. Alonso-Zarazaga, personal communication). The fact that Casey subsequently used the name *Telabis* as feminine is irrelevant.

^23^ The species name “*minor*” is an adjective in comparative degree and is the masculine and feminine form. The neuter form is “*minus*” (M.A. Alonso-Zarazaga, personal communication).

^24^ Listed as a synonym of *T. laevis* LeConte by Horn (1870: 259) and Champion (1884: 2) but treated as valid by Casey (1907: 438).

^25^ The specific name was obviously proposed for Auguste Sallé [b. 1820, d. 1896], who collected some of the original specimens, and should have been spelled “*sallei*.” Because Champion did not provided the etymology of the name, the original spelling should be maintained and Article 32.5.1 (ICZN 1999) does not apply in this case. However, the spelling “*sallei*” is in prevailing usage (36 hits in Google Scholar vs 11 hits for “*sallaei*”) and so deemed to be the original spelling (ICZN 1999, Article 33.3.1).

^26^ Gebien (1908a: 287) originally placed this name in synonymy with *Nyctoporis cristata* Eschscholtz and latter (1910a: 118; 1937: 698) with *Nyctoporis carinata* LeConte.

^27^ The generic name is spelled “*Caribanoisis*” in the running title (p. 569). Since the running title was generated by the publisher, it is not considered as a different original spelling and no First Reviser action is required here.

^28^ This Oriental species is known in North America from a single specimen (Steiner and Paraskevoudakis 2014) and whether it is established on this continent remains to be ascertained.

^29^ The names *Eleodes chevrolati* and *Eleodes nitidulithorax* used by Pierre (1976: 705, 706) are *nomina nuda*. The same is true for the name *Eleodes dentipes sublitoralis* listed by Gebien (1938: 57). The following names, being infrasubspecific, are *nomina nuda*: *Eleodes omissa* var. *pygmaea* forma *borealis* Blaisdell, 1909: 79; *Eleodes extricata* forma *cognata punctata* Blaisdell, 1909: 123; *Eleodes letcheri* var. *vandykei* forma *parvula* Blaisdell, 1909: 137; *Eleodes dentipes* forma *elongata laevis* Blaisdell, 1909: 254; *Eleodes dentipes* forma *elongata punctata* Blaisdell, 1909: 254; *Eleodes acuticauda* var. *laticollis* forma *minor* Blaisdell, 1909: 283; Eleodes (Eleodes) acuticauda var. *laticollis* forma *insularis* Blaisdell, 1909: 284; *Eleodes eschscholtzi* var. *lucae* forma *ecaudata* Blaisdell, 1909: 290; *Eleodes eschscholtzi* var. *lucae* forma *grandis* Blaisdell, 1909: 291; *Eleodes eschscholtzi* var. *lucae* forma *inflata* Blaisdell, 1909: 291. Even though some of these names have been listed as valid subspecies by Papp (1961), article 45.6.4.1 (ICZN 1999) does not apply here and the names are not available.

^30^ This species is usually credited to Eschscholtz (1831: 13) where the species is described. However, in 1829 Eschscholtz described the genus *Amphidora* and listed by name the sole species (*A. littoralis*) included in it. As such it is a combined description of a new nominal genus and a single new nominal species which then provides an indication for each name (ICZN 1999: Article 12.2.6). Sherborn (1927: 3618) also credited Eschscholtz (1829) for the availability of *A. littoralis*.

^31^ Blaisdell (1937b) used two different spelling for this taxon: *Arpeleodes* (p. 128) and *Ardeleodes* (p. 128, 129). Gebien (1938: 63) acted as First Reviser (ICZN 1999: Article 24.2.3) and selected *Ardeleodes* as the correct original spelling.

^32^ The species name *grutus* is neither Latin or Greek but appears to be derived from the Spanish word “gruta (cave)” and transformed as an adjective (M.A. Zlonso-Zarazaga, personal communication). Since it is a creation of the authors, the name must be treated as a noun in apposition.

^33^ This specific name was originally spelled *vetorator* but *veterator* is an incorrect subsequent spelling in prevailing usage and attributed to the publication of the original spelling; therefore the subsequent spelling is deemed to be the correct original spelling (ICZN 1999: Article 33.3.1).

^34^ Thomas (2005: 553) retained the name *transvolcanensis* for the species and *rufipes* for the subspecies. However, under Article 47.2 of the ICZN (1999) the oldest name for the species must be retained.

^35^ The species name *anachronus* is not a classical Greek or Latin name and seems a creation of Triplehorn (M.A. Alonso-Zarazaga, personal communication). Therefore the name is deemed to be a noun in apposition.

^36^ We consider this name available from Blaisdell (1910: 61) with the description by indication (ICZN 1999, Article 12.2.1) to his monograph of the Eleodiini published in 1909.

^37^ Described by indication.

^38^ An application to the International Commission on Zoological Nomenclature was recently published (Bousquet and Bouchard 2017b) to retain *Bolitotherus cornutus* (Fabricius, 1801) as the valid name for this taxon.

^39^ As for *Amblycyphus asperatus* (see footnote #20), evidence gathered recently indicates that Motschulsky's manuscript was published prior to that of Horn and that *Threnus* has precedence. The Reversal of precedence cannot be used in this case since *Threnus* was listed as valid after 1899 in some catalogues (e.g., Leng 1920: 224). An application to the Commission will be necessary to retain Horn's name as valid.

^40^ A number of authors, including LeConte (1866a: 61) and Horn (1870: 325), mistakenly used the name *sulcipennis* for this species.

^41^ Species described from “Californie. St. Francisco.”

^42^ Berry (1975: 929) proposed the new subgenus *Longicerenopus* to include *C. angustatus* Horn, *C. aterrimus* Horn, *C. hermanus* Berry, and *C. punctatus* Berry. Unfortunately the author failed to designate a type species and therefore his name is unavailable from that date. Staff of the Zoological Society of London (Anonymous 1979: 217) designated *C. angustatus* Horn as type species but since the person responsible for the nomenclatural act cannot be determined from the work itself, the act must be attributed to “Anonymous.” Names and nomenclatural acts published anonymously after 1950 are unavailable (ICZN 1999: Article 14) and so consequently the name *Longicerenopus* is still unavailable.

^43^ *Helops martinensis* Marcuzzi, 2001 (p. 252) is a *nomen nudum* since Marcuzzi (2001) failed to indicate where the syntypes will be (or are) deposited and the name and location of the collection, a mandatory requirement for every new species-group name published after 1999 (ICZN 1999: Article 16.4.2).

^44^ According to M.A. Alonso-Zarazaga (personal communication), *Tenebrio caeruleus* Linnaeus, 1758, based on a syntype, is a chrysomelid species of the genus *Timarcha*. This problem will be addressed in a future publication.

^45^ Allard (1877a: 36) corrected the name *Coscinopter* to *Coscinoptilix* in the errata issued in one part of the same journal volume. Therefore *Coscinoptilix* is the correct original spelling (ICZN 1999: Article 32.5).

^46^ *Helops punctatus* Fabricius, 1801 (currently *Taraxides punctatus*) and *Helops punctatus* Gemminger, 1870 are primary homonyms, both presently used as valid, that have not been considered congeneric after 1899. In such case, the junior homonym is not to be replaced and the case should be referred to the Commission (ICZN 1999: Article 23.9.5).

^47^ Horn (1885a: 90) mentioned that this species “is certainly not a *Helops*” and that he was unable to identify it. He also questioned the fact that the species was of American origin.

^48^ Horn (1885a: 89) placed *H. americanus* Palisot de Beauvois in synonymy with *Helops undulatus* LeConte based on the original description only. His action was not followed by subsequent authors. Therefore we prefer to continue using LeConte’s name and consider Palisot de Beauvois’ name as a *nomen dubium*. The reversal of precedence (ICZN 1999: Article 23.9) cannot be used here since *Helops americanus* has been used as a valid name at least once after 1899 (i.e., Scott and Fiske 1902: 33).

^49^ Removed from synonymy with *B. fuscus* Casey by Steiner (2006).

^50^ Kaszab (1969) used two different spellings for this taxon: *kulzrei* (p. 318) and *kulzeri* (pp. 318, 337). Nobody has acted as First Reviser (ICZN 1999: Article 32.2.1) to our knowledge. We act as First Revisers and choose *kulzeri* (based on H. Kulzer), which was obviously Kaszab’s intention although this was not explicitely mentioned in the text.

^51^ *Diastolinus bardubensis* [sic] *guadeloupensis* Marcuzzi, 2001 (p. 250) and *Diastolinus bardubensis* [sic] *martiniquensis* Marcuzzi, 2001 (p. 250) were placed in synonymy with *Blapstinus opacus* Mulsant and Rey by Soldati and Touroult (2014: 98) and Ivie and Hart (2016: 466) respectively. However, these two taxa are *nomina nuda* since Marcuzzi (2001) failed to indicate where the syntypes will be (or are) deposited and the name and location of the collection, a mandatory requirement for every new species-group name published after 1999 (ICZN 1999: Article 16.4.2).

^52^ According to Marcuzzi (2002: 399), the holotype and sole known specimen of this species was probably transported by ship from Cuba to Curaçao.

^53^ This species, originally described from specimen(s) collected in Missouri, has never been interpreted except by Horn (1870: 402) who mentioned that it probably belongs to the genus *Opatrinus* (North American members of this genus are currently placed in the genus *Alaetrinus*). The name is tentatively placed here in synonymy with *A. minimus* (Palisot de Beauvois), a common species.

^54^ Ferrer (2006) mentioned that *Z. atratus* (Fabricius) and *morio* (Fabricius) are “two forms of the same species” and that specimens of both forms emerge in laboratory from the same parents. Later (Ferrer 2011: 299) considered *morio* as a distinct species. However based on the data presented in Ferrer’s papers of 2006 and 2011, we believe that specimens of the form *morio* are probably conspecific with those of *atratus*. Soldati and Touroult (2014: 103) also considered *morio* as a synonym of *atratus*.

^55^ Considered a valid species by Garrido and Gutiérrez (1994b), with *Zophobas cubanus* Marcuzzi, 1976 listed as a junior synonym. Ferrer (2011: 297) listed *Z. rugipes* as a junior synonym of *Z. atratus* (Fabricius).

^56^ Most previous literature records attributed to *Wattius cucullatus* (Pascoe, 1871) belong to multiple undescribed species [ADS]. *Wattius cucullatus* is currently known only from Brazil (Smith and Sanchez 2015: 115).

^57^ Spilman (1973: 42) mentioned that the *Ulosonia tricornis* of Laporte (1840) was in fact a reference to *Phaleria tricornis* Dalman, 1823 with an incorrect author. We believe these two names are different nomenclatural acts though they are probably synonymous as indicated by Spilman.

^58^ The name derives from the Greek root *Spalae* (cave) and the noun *biosis* (manner of live) in reference to the cave-inhabiting sole species currently included in the genus.

^59^ *Eusemostene* proposed by Gebien (1940: 764) is regularly listed as a junior synonym of this taxon. Although listed as “nom. nov.” Gebien in fact proposed a new subgeneric taxon by providing a bibliographic reference to Seidlitz’s (1890: 518) misidentification of *Stene* Stephens, 1829. However, the name is unavailable because Gebien failed to designate a type species for his new subgenus (see ICZN 1999: Article 13.3). The name has not been made available subsequently to our knowledge.

^60^ Reported under the unavailable name *Alegoria laportei* Fleutiaux and Sallé, 1890 by some authors (e.g., Peck 2011: 30).

^61^ Only one specimen, from Mexico, is known north of Colombia (Champion 1886: 152); possibly the specimen is mislabelled.

^62^ *Melasia gagatina* Perroud and Mulsant, 1861, has been listed from North America (Gebien 1911a: 402) and placed in synonymy with *Uloma punctulata* LeConte, 1866 (Gebien 1940: 776). In fact, the species was described from Sicily and is a junior synonym of *Uloma culinaris* (Linnaeus, 1758) (see Löbl et al. 2008: 302).

^63^ This taxon has been listed in synonymy with *Tribolium castaneum* (Herbst) by some authors. However, the size originally given, 3⅓ lig. (= 7.4 mm) prevent an association with *T. castaneum*. We concur with Good (1936: 5) that Laporte’s species probably belongs to the genus *Uloma* Dejean. Unfortunately, based on the short original description, it cannot be confidently associated with any of the species described from North America.

^64^ The name of this species has been credited to Jacquelin du Val (1857: 157) by some authors (e.g., Blackwelder 1945: 506; Campbell 1966: 25; Campbell 1971: 27). However, Jacquelin du Val referred the name to Dejean’s catalogue (1834: 214) who used the replacement name *fuscula* Schönherr as valid and listed *flavipes* Fabricius in synonymy. Therefore we consider that Jacquelin du Val did not propose a new species under the name “*Allecula flavipes* Dejean.”

^65^ The junior and senior homonyms for *Cistela brevis* apply to taxa not considered congeneric since 1899. In such case, the junior homonym must not automatically be replaced; the case should be referred to the Commission for a ruling and meanwhile prevailing usage of both names is to be maintained (ICZN 1999: Article 23.9.5).

^66^ Although this name is older than Adelinina, Bouchard et al. (2011: 428) recommended that an application be submitted to the Commission to conserve usage of Adelinina LeConte, 1862.

^67^ Palisot de Beauvois (1812) used two different spellings for this taxon: *Trogossita maxillosa* (p. 125) and *Trogossita maxillaris* (pl. 32, fig. 4). Page 125 and plate 32 were both issued in 1812 in livraison 8 of Palisot de Beauvois’ work. We have not found anyone who acted as First Reviser. We here select *T. maxillaris* as the correct spelling. This name was used regularly for Palisot de Beauvois’ taxon probably because *T. maxillosus* was already in use by Fabricius in 1801.

^68^ *Diaperis hydni* is an incorrect subsequent spelling of *D. hydactina* Fabricius, 1798 introduced by Fabricius (1801b: 585).

^69^ LeConte (1870: 404) believed the species was “wrongly attributed to North America.”

^70^ LeConte (1870: 404) believed the species was “wrongly attributed to North America.”

^71^ LeConte (1870: 404) noted that *Platydema pallens* “is found in South America, and must therefore be erased from the list of the Coleoptera of North America.”

^72^ This species is listed in synonymy with *Platydema nigratum* (Motschulsky) in some catalogues. However, Triplehorn (1965: 424) indicated that it was “quite distinct.”

^73^ Dajoz (1975) used two spellings for the specific name, *jamaicensis* (p. 118) and *jamaiscensis* (pp. 120, 121). Ivie and Hart (2017b: 118) acted as “First Revisers” and choose *jamaicensis* as the correct original spelling.

^74^ Until the type specimens of *Menimopsis jamaicensis* (Dajoz) are studied, we prefer not to propose a replacement name for Kaszab’s taxon.

^75^ Soldati and Touroult (2014: 106) believed this species to be a Cerylonidae and not a Tenebrionidae.

^76^ May not be established as there is no record since the 1950’s (Peck 2011: 29).

^77^ This species has passed under the name of *Alobates morio* (Fabricius) in many publications following the study of putative types by Blair (1914: 487) in the Banks collection as pointed out by Spilman (1962b: 60). However, Ferrer (2006: 232) mentioned that the “types” seen by Blair were not syntypes and that the true syntype he studied at the Museum of Copenhagen belongs in fact to *Zophobas atratus* (Fabricius, 1775).

^78^ The generic name *Scotera* and the specific name *gibbosa* were proposed as synonyms of *Cibdelis blaschkii* by Motschulsky (1845b: 365, 366). However, both taxa are available from Motschulsky’s work (ICZN 1999: Article 11.6.1) because they were treated as valid names before 1961 (e.g., Chevrolat 1848: 454).

^79^ Doyen (1973) retained *C. californica* as the valid name for this taxon. However, Horn (1870: 336) acted as First Reviser (ICZN 1999, Article 24.2) in synonymizing *C. californica* and *C. dilaticollis* together and chose *C. dilaticollis* as the valid name for the taxon. Therefore *C. dilaticollis* is the valid name for this species.

^80^ Credited to Chevrolat (1844) in previous catalogues.

^81^ *Moeon* is the Latinization of the Greek noun *moion* (privy parts) and is neuter (M.A. Alonso-Zarazaga, personal communication).

^82^ Pallas (1781) proposed four species in *Mylaris* but did not described the genus. In a footnote on page 38, Pallas mentioned “Nomen *Mylaridis*, –molendinariae appropriatum, male applicavit Meloïdibus Fabricius [The name of *Mylaris*, –appropriate for a miller species, was incorrectly applied to Meloids by Fabricius].” It seems that Pallas used an incorrect subsequent spelling of *Mylabris* Fabricius, which was proposed by Fabricius (1775) for some meloid species, and decided to apply the name to tenebrionids. As such *Mylaris*, used by Pallas (1781), should be an incorrect subsequent spelling and not an available name. No action is taken at this time.

^83^ The name “*cuproso*” was used three times in the publication (pp. 98, 99, 203) and is not a lapsus. To our knowledge the species was treated only once subsequently (Hopp and Ivie 2009: 54) who used the spelling “*cuprosum.*” We do not consider that *cuprosum* is in prevailing usage and therefore, *cuproso* must be retained as the proper spelling.

^84^ Listed in the genus *Otocerus* by Ferrer and Ødegaard (2005: 648).

^85^ The name *preciosus* is a blend of Spanish and Latin, and as such must be treated as a noun in apposition (M.A. Alonso-Zarazaga, personal communication).

^86^ This Pleistocene fossil species was described from marine shale.

## Appendix 1

List of impression fossil Tenebrionidae taxa described from North America. ^1^ = species originally described from Germany but subsequently reported from Greenland by Heer (1883: 145). ^2^ = assignment of this species to the tribe Blaptini (e.g., Kirejtshuk et al. 2008) is doubtful since this tribe is not represented by any extant taxa in North America.

**Table:**

Species	Origin	Age	Placement
*Blapstinus linellii* Wickham, 1913a: 298	USA (Colorado)	37.2 to 33.9 Ma	Tenebrioninae: Opatrini: Opatrina
*Capnochroa senilis* Wickham, 1913b: 365	USA (Colorado)	37.2 to 33.9 Ma	Alleculinae: Alleculini: Gonoderina
*Cistelites minor* Heer, 1874: 25	Greenland	61.7 - 58.7 Ma	Alleculinae
*Cistelites punctulatus* Heer, 1870: 484	Greenland	61.7 - 58.7 Ma	Alleculinae
*Ephalus adumbratus* Scudder, 1893: pl. 1 (fig. 3)	USA (Colorado)	37.2 to 33.9 Ma	Tenebrioninae: Opatrini: Opatrina
*Gonodera antiqua* (Wickham, 1913b: 365)	USA (Colorado)	37.2 to 33.9 Ma	Alleculinae: Alleculini: Gonoderina
*Gonodera vulcanica* (Wickham, 1914a: 485)	USA (Colorado)	37.2 to 33.9 Ma	Alleculinae: Alleculini: Gonoderina
*Helops wetteravicus* C. von Heyden & L. von Heyden, 1865: 33^1^	Greenland	61.7 - 58.7 Ma	Tenebrioninae: Helopini
*Hymenorus haydeni* Wickham, 1914a: 486	USA (Colorado)	37.2 to 33.9 Ma	Alleculinae: Alleculini: Alleculina
*Isomira aurora* Wickham, 1914b: 268	USA (Colorado)	37.2 to 33.9 Ma	Alleculinae: Alleculini: Gonoderina
*Isomira florissantensis* Wickham, 1914a: 486	USA (Colorado)	37.2 to 33.9 Ma	Alleculinae: Alleculini: Gonoderina
*Meracantha lacustris* Wickham, 1909: 129	USA (Colorado)	37.2 to 33.9 Ma	Tenebrioninae: Amarygmini
*Miostenosis lacordairei* Wickham, 1913a: 297	USA (Colorado)	37.2 to 33.9 Ma	Pimeliinae: Stenosini
Pelecyphorus (Stenosides) primus (Wickham, 1910: 51)	USA (Colorado)	37.2 to 33.9 Ma	Pimeliinae: Asidini
*Platydema antiquorum* Wickham, 1912: 32	USA (Colorado)	37.2 to 33.9 Ma	Diaperinae: Diaperini: Diaperina
*Platydema bethunei* Wickham, 1913a: 299	USA (Colorado)	37.2 to 33.9 Ma	Diaperinae: Diaperini: Diaperina
*Proteleates centralis* Wickham, 1914b: 267^2^	USA (Colorado)	37.2 to 33.9 Ma	Tenebrioninae: Blaptini
*Protoplatycera laticornis* Wickham, 1914a: 484	USA (Colorado)	37.2 to 33.9 Ma	Tenebrionidae
*Tenebrio primigenius* Scudder, 1879: 183B	Canada (British Columbia)	55.8 to 40.4 Ma	Tenebrioninae: Tenebrionini
*Teneobrionites alatus* Cockerell, 1927: 586	USA (Colorado)	37.2 to 33.9 Ma	Tenebrionidae
*Ulus minutus* Wickham, 1914b: 266	USA (Colorado)	37.2 to 33.9 Ma	Tenebrioninae: Opatrini: Opatrina

## Appendix 2

List of amber fossil Tenebrionidae taxa described from North America. ^1^ = new replacement name for *Hymenorus oculatus* Doyen and Poinar, 1994: 37 which is a junior primary homonym of *Hymenorus oculatus* Champion, 1888. ^2^ = Species originally described in the genus *Hesiodobates* Kaszab and Schawaller, 1984 which is currently considered a junior synonym of *Nesocyrtosoma* Marcuzzi, 1976 (Doyen and Poinar 1994).

**Table:**

Species	Origin	Age	Placement
*Corticeus tertiarius* Vitali, 2007: 2	Dominican Republic	20.4 – 13.7 Ma	Diaperinae: Hypophlaeini
*Cymatothes dominicus* Doyen and Poinar, 1994: 36	Dominican Republic	20.4 – 13.7 Ma	Tenebrioninae: Amarygmini
*Hymenorus campbelli* Bouchard, new replacement name^1^	Dominican Republic	20.4 – 13.7 Ma	Alleculinae: Alleculini: Alleculina
*Hymenorus chiapasensis* Campbell, 1963: 41	Mexico (Chiapas)	23.0 – 16.0 Ma	Alleculinae: Alleculini: Alleculina
*Hypogena marginalis* Doyen and Poinar, 1994: 35	Dominican Republic	20.4 – 13.7 Ma	Tenebrioninae: Triboliini
*Liodema phalacroides* Doyen and Poinar, 1994: 43	Dominican Republic	20.4 – 13.7 Ma	Diaperinae: Diaperini: Diaperina
*Lobopoda annosa* Doyen and Poinar, 1994: 37	Dominican Republic	20.4 – 13.7 Ma	Alleculinae: Alleculini: Alleculina
*Lorelus angulatus* Doyen and Poinar, 1994: 28	Dominican Republic	20.4 – 13.7 Ma	Lagriinae: Lupropini
*Lorelus foraminosus* Doyen and Poinar, 1994: 28	Dominican Republic	20.4 – 13.7 Ma	Lagriinae: Lupropini
*Lorelus minutulis* Doyen and Poinar, 1994: 30	Dominican Republic	20.4 – 13.7 Ma	Lagriinae: Lupropini
*Lorelus wolcotti* Doyen and Poinar, 1994: 30	Dominican Republic	20.4 – 13.7 Ma	Lagriinae: Lupropini
*Neomida senicula* Doyen and Poinar, 1994: 41	Dominican Republic	20.4 – 13.7 Ma	Diaperinae: Diaperini: Diaperina
*Nesocyrtosoma antiquum* (Kaszab and Schawaller, 1984: 3)^2^	Dominican Republic	20.4 – 13.7 Ma	Stenochiinae: Cnodalonini
*Nesocyrtosoma celadonum* Doyen and Poinar, 1994: 46	Dominican Republic	20.4 – 13.7 Ma	Stenochiinae: Cnodalonini
*Nesocyrtosoma hadratum* Doyen and Poinar, 1994: 47	Dominican Republic	20.4 – 13.7 Ma	Stenochiinae: Cnodalonini
*Nesocyrtosoma impensum* Doyen and Poinar, 1994: 47	Dominican Republic	20.4 – 13.7 Ma	Stenochiinae: Cnodalonini
*Nesocyrtosoma minisculum* Hopp and Ivie, 2009: 80	Dominican Republic	20.4 – 13.7 Ma	Stenochiinae: Cnodalonini
*Nesocyrtosoma phthanatum* Doyen and Poinar, 1994: 48	Dominican Republic	20.4 – 13.7 Ma	Stenochiinae: Cnodalonini
*Nilio dominicana* Poinar and Brown, 2011: 233	Dominican Republic	20.4 – 13.7 Ma	Nilioninae
*Rhipidandrus quadripapillatus* Doyen and Poinar, 1994: 33	Dominican Republic	20.4 – 13.7 Ma	Tenebrioninae: Bolitophagini
*Statira dermoidea* Doyen and Poinar, 1994: 30	Dominican Republic	20.4 – 13.7 Ma	Lagriinae: Lagriini: Statirina
*Trientoma nascens* Doyen and Poinar, 1994: 31	Dominican Republic	20.4 – 13.7 Ma	Pimeliinae: Edrotini
*Tyrtaeus azureus* Doyen and Poinar, 1994: 39	Dominican Republic	20.4 – 13.7 Ma	Diaperinae: Gnathidiini: Anopidiina
*Tyrtaeus cupreorutilans* Vitali, 2008: 12	Dominican Republic	20.4 – 13.7 Ma	Diaperinae: Gnathidiini: Anopidiina
*Tyrtaeus elongatus* Doyen and Poinar, 1994: 40	Dominican Republic	20.4 – 13.7 Ma	Diaperinae: Gnathidiini: Anopidiina
*Tyrtaeus flavoantennatus* Doyen and Poinar, 1994: 40	Dominican Republic	20.4 – 13.7 Ma	Diaperinae: Gnathidiini: Anopidiina
*Tyrtaeus thoracicus* Doyen and Poinar, 1994: 41	Dominican Republic	20.4 – 13.7 Ma	Diaperinae: Gnathidiini: Anopidiina
*Wattius reflexus* Doyen and Poinar, 1994: 33	Dominican Republic	20.4 – 13.7 Ma	Tenebrioninae: Toxicini: Dysantina

## Appendix 3

List of species incorrectly recorded from North America.

*Allophasia marseuli* Bates, 1873c: 237. The species was described from a unique specimen with no locality data. It was doubtfully recorded from North America by Papp (1961d: 122). Triplehorn and Brendell (1985: 12) found a few conspecific specimens from South America suggesting that it occurs in South America.

*Ammophorus denticollis* Boheman, 1858: 89. The species was originally reported from Panama. However Van Dyke (1953: 95) believe the specimen(s) was mislabelled as some others of Boheman’s species listed from Panama and was in fact collected in the Galápagos Islands. Since the genus is found only in Chile, Peru, mainland Ecuador, and the Galápagos Islands (Peck 2006: 219), Van Dyke’s statement is likely correct.

*Ammophorus obscurus* G.R. Waterhouse, 1845: 32. Waterhouse described this species from the Galápagos Islands. Blaisdell (1943: 238) reported seeing one specimen of the species from San José del Cabo in Baja California Sur but also suggested that its provenance should be taken with some doubt. For the reason outlined for the previous species, the Mexican record is unreliable and certainly based on a mislabelled specimen.

*Blapstinus latifrons* LeConte, 1874: 70. This species was described from one specimen collected on Vancouver Island. The name is currently a junior synonym of *Gonocephalum bilineatum* Walker, a widespread species in southeast Asia (Kaszab 1952: 682). As pointed out by Aalbu and Triplehorn (1985: 273), this isolated record is probably the result of an accidental interception.

*Cistelopsis instriata* Pic, 1930: 23. This species, described from “Indes Méridionales,” was recorded from “West Indies” by Blackwelder (1945: 508). However we believe the record is based on a misinterpretation of the region given by Pic and the species is probably an inhabitant of India (see next entry).

*Cistelopsis rufomarginata* Pic, 1930: 28. This species, along with its variety *ruficolor*, were described from “Indes Méridionales” and recorded from “West Indies” by Blackwelder (1945: 508) apparently from a misinterpretation of the locality given by Pic (1930: 28). The species, which is currently included in the genus *Stilbocistela* Borchmann, is known from India (Novák 2013: 180).

*Cyclosattus websteri* Casey, 1891: 710. The original locality given for this species is “Colorado.” However, Doyen et al. (1990: 244) pointed out that the species is a synonym of the Australian *Cilibe costatus* Solier, 1848 which is currently placed in the genus *Saragus* Erichson, 1842.

*Cyrtosoma excisicollis* Gebien, 1928b: 198. This species was described from specimens originating in “Paramaribo” and “Amazonas” and doubtfully recorded from North America by Papp (1961d: 131). We have found no record indicating that the species occurs in North America.

*Emmenastus rugosus* Motschulsky, 1845a: 76. This taxon was originally described on specimens from “Sitka” in Alaska. Aalbu et al. (1995: 484) studied a syntype of the species from the Zoological Museum of the University of Moscow and concluded that it belongs to the genus *Oxycara* Solier, 1835. The genus is not found in North America and therefore the locality originally listed is incorrect.

*Helops ovipennis* Casey, 1890b: 487. The locality given for this species was “Majave Desert, California”. Spilman (1959: 63) matched the holotype in USNM to members of *Adelium* Kirby which are found in the Australian Region and South America. Matthews (1998: 777) added that the holotype belongs to a valid species from New South Wales closely related to *Adelium heterodoxum* Lea and usually confused with it in collections. The specimen is obviously mislabelled.

*Hyocis championi* Fauvel, 1904: 166. This species has been listed from “Baja Calif.” by Blackwelder (1945: 525). The species belongs to the genus *Parahyocis* Kaszab and is found in New Caledonia, Vanuatu and Samoa (Kaszab 1955: 645). Blackwelder’s record is incorrect.

*Iphthinus aereus* Melsheimer, 1846: 65. The locality listed for this taxon was “Pennsylvania.” The species actually belongs to the genus *Encyalesthus* Motschulsky, 1860 (= *Derosphaerus* J. Thomson, 1858) from the Oriental Region as first pointed by LeConte (1873: 335). The original locality is obviously incorrect. Melsheimer’s species is the type species of the genus *Pachyurgus* LeConte, 1862 which is a junior synonym of *Derosphaerus*.

*Maracia haagi* Gebien, 1919: 35. This species was described from one specimen without collection locality. It was reported from South America by Blackwelder (1945: 538) but from Central America by Papp (1961d: 131). As there is no way to be certain that the species occurs in North America, it is not included in this catalogue. The only other species placed in the genus, *M. femoralis* (Kirsch), is known from Colombia.

*Opatrum agricola* Herbst, 1783: 35. The only record of this species in North America is from Horn (1870: 389) who, under the new specific name *Eledona fungicola*, mentioned that it was collected in the “Middle States.” Since the species was not found subsequently, it is likely that Horn’s specimen(s) was mislabelled. *Eledona fungicola* Horn was placed in synonymy with *E. agricola* (Herbst) by LeConte and Horn (1883: 384).

*Oplocephala chalybea* Laporte and Brullé, 1831: 341. The authors mentioned “La patrie de cette espèce est Philadelphie.” Horn (1870: 380) stated that the species is “probably not North American,” which is also the opinion of Triplehorn (2006: 332).

*Oplocephala collaris* Laporte and Brullé, 1831: 347. Laporte and Brullé stated “Cette espèce fait partie de la collection de M. Dupont, qui l’a reçue de Philadelphie.” Triplehorn (2006: 332) pointed out that the statement do not indicate that the species was collected in Philadelphia. From the description, including the size, the name cannot be attributed to any species from northeastern North America. Likely the species was not collected in North America.

*Phaleria ornata* Wollaston, 1864: 494. This species was recorded by Papp (1961d: 121) from “Alas. to Can.” and the subspecies *P. ornata bockeri* Heyden and *P. ornata nigrothoracica* Heyden from “N. Am.” Actually the species *Phaleria ornata*, of which the two Heyden’s names are junior synonyms, is endemic to the Canary Islands (Löbl et al. 2008: 316). Papp (1961d: 121) records are obviously incorrect.

*Phrenapates mandibularis* Gebien, 1910b: 504. This species was described from “Nördl. Süd-Amerika” and doubtfully recorded from North America by Papp (1961d: 124). We have not found any record that would indicate that the species occurs in North America.

?*Platolenes desfontainesi* Pic, 1952: 2. This species was originally described from Haiti. It was doubtfully transferred to the genus *Amarygmus* Dalman,1823 by Bremer (2001: 57). Since the genus *Amarygmus* is not represented in the Western Hemisphere, it is possible that Pic’s specimen(s) were mislabelled.

*Platydema infuscata* Laporte and Brullé, 1831: 373. This species was originally reported from “Colombie.” It was doubtfully listed from North America by Papp (1961d: 123). We have found no record indicating that the species occurs in North America.

*Tenebrio angulatus* Perty, 1830: 57. This species, described from “Prov. Piauhiensi” in Brazil, was reported under the generic name *Tauroceras* Hope, 1841 from several places in North America (see Champion 1885: 106; Champion 1892: 523; Blackwelder 1945: 535; Papp 1961d: 129). According to Ferrer et al. (2005) who revised the genus *Tauroceras*, Perty’s *T. angulatus* occurs only in South America.

*Tenebrio olivensis* Wollaston, 1864: 501. This species is listed from Canada by Papp (1961d: 130). The name is actually a junior synonym of *Zidalus niloticus* (Mulsant and Rey, 1853). The species is found only in the Canaries, Libya, Yemen and the Afrotropical Region (Löbl et al. 2008: 291). Papp’s record from North America is obviously incorrect.

*Tenebrio picipes* Herbst, 1797: 245. This species, which belongs to the genus *Neatus* LeConte, is listed as occurring in the Nearctic Region by Löbl et al. (2008: 299) apparently based on old records from the North American literature. Actually the species is found only in the Palaearctic Region and the records from North America refers to *Neatus tenebrioides* (Palisot de Beauvois).

*Trogosita bidens* Fabricius, 1792: 116. This species was originally reported from “Gallia.” Gebien (1906: 233) transferred the species to the genus *Alegoria* Laporte and mentioned that the original locality is incorrect. Gebien (1940: 768) listed the species from “Am. mer.,” Blackwelder (1945: 531) from “South America” and Papp (1961d: 126) from “N. Am.” Until a North American locality is recorded, the species is not included for the North American fauna.

## Appendix 4

Supporting references for conservation of *Tarpela micans* (Fabricius, 1798) over *Tarpela vittata* (Olivier, 1793) through reversal of precedence (ICZN 1999: Art. 23.9.2). To our knowledge *Tarpela vittata* (Olivier) has not been used as a valid name after 1899.

Anonymous (2013) Patuxent Research Refuge: comprehensive conservation plan. United States Fish and Wildlife Service, Laurel, Maryland.

Bousquet Y, Bouchard P, Davies AE, Sikes DS (2013) Checklist of beetles (Coleoptera) of Canada and Alaska. Second edition. Pensoft, Sofia-Moscow, 402 pp.

Bousquet Y, Campbell JM (1991) Family Tenebrionidae - darkling beetles. In: Bousquet Y (Ed.) Checklist of beetles of Canada and Alaska. Agriculture Canada, Ottawa, 253–261.

Cifuentes-Ruiz P, Zaragoza-Caballero S, Ochoterena-Booth H, Morón MA (2014) A preliminary phylogenetic analysis of the New World Helopini (Coleoptera, Tenebrionidae, Tenebrioninae) indicates the need for profound rearrangements of the classification. ZooKeys 415: 191–216. https://doi.org/10.3897/zookeys.415.6882

Ciegler HC (2014) Tenebrionoidea of South Carolina (Coleoptera: Mycetophagidae, Archeocrypticidae, Tetratomidae, Melandryidae, Mordellidae, Ripiphoridae, Zopheridae, Tenebrionidae, Synchroidae, Oedemeridae, Stenotrachelidae, Meloidae, Mycteridae, Boridae, Pythidae, Pyrochroidae, Salpingidae, Anthicidae, Ischaliidae, and Aderidae). Clemson University Public Service Publishing, Clemson, 243 pp.

Dillon E, Dillon LS (1972) A manual of common beetles of eastern North America. Volume II. Dover Publications, Inc., New York, 435–894.

Downie NM, Arnett RH Jr (1996) The beetles of northeastern North America. Volume II: Polyphaga: Series Bostrichiformia through Curculionoidea. The Sandhill Crane Press, Gainesville (Florida), x +891–1721.

Dunford JC, Young DK (2004) An annotated checklist of Wisconsin darkling beetles (Coleoptera: Tenebrionidae) with comparisons to the western Great Lakes fauna. Transactions of the American Entomological Society 130: 57–76.

Evans AV (2014) Beetles of eastern North America. Princeton University Press, Princeton & Oxford, 560 pp. https://doi.org/10.1515/9781400851829

Gardner JE (1986) Invertebrate fauna from Missouri caves and springs. Missouri Department of Conservation, Natural History Series, no. 3, i–vi, 1–72.

Guarnieri FG (2010) A survey of the beetles (Coleoptera) of Pocomoke River State Park, Worcester County and Tuckahoe State Park, Caroline County, Maryland. The Maryland Entomologist 5(2): 5–28.

Headstrom R (1977) The beetles of America. Barnes & Company, Cranbury (NJ).

Laplante S, Bousquet Y, Bélanger P, Chantal C (1991) Liste des espèces de coléoptères du Québec. Fabreries Supplément 6, 136 pp.

Majka CG, Chandler DS, Donahue CP (2011). Cheklist of the beetles of Maine, USA. Empty Mirrors Press, Halifax, 328 pp.

Matthews EG, Lawrence JF, Bouchard P, Steiner WE, Ślipiński SA (2010) Tenebrionidae Latreille, 1802. In: Leschen RAB, Beutel RG, Lawrence JF (Eds) Handbook of Zoology. Coleoptera, Beetles. Volume 2: Morphology and systematics (Elateroidea, Bostrichiformia partim). De Gruyter, Berlin, New York, 574–659.

Nabozhenko M, Löbl I (2008) tribe Helopini Latreille, 1802. In: Löbl I, Smetana A (Eds) Catalogue of Palaearctic Coleoptera. Volume 5. Tenebrionoidea. Apollo Books, Stenstrup, 241–257.

Poole RW, Gentili P (Eds) (1996) Nomina Insecta Nearctica. A Check List of the Insects of North America. Volume 1: Coleoptera, Strepsiptera. Entomological Information Services, Rockville, Maryland, 820 pp.

Shockley FW, Cline AR (2004) A contribution to the inventory of Coleoptera of Missouri: new records from Benton County. Journal of the Kansas Entomological Society 77: 280–284. https://doi.org/10.2317/0304.21.1

Sikes DS (2004) The beetle fauna of Rhode Island: an annotated checklist. The Biota of Rhode Island, volume 3. The Rhode Island Natural History Survey, Kingston, 286 pp.

Steiner WE Jr (1995) Structures, behavior and diversity of the pupae of Tenebrionidae (Coleoptera). In: Pakaluk J, Slipinski SA (Eds) Biology, phylogeny, and classification of Coleoptera. Papers Celebrating the 80th Birthday of Roy A. Crowson. Muzeum i Instytut Zoologii PAN, Warszawa, 503–539.

Steiner WE Jr (2008) A checklist of the darkling beetles (Insecta: Coleoptera: Tenebrionidae) of Maryland, with notes on the species recorded from Plummers Island through the 20th Century. Bulletin of the Biological Society of Washington 15: 133–140. https://doi.org/10.2988/0097-0298(2008)15[133:ACOTDB]2.0.CO;2

Steiner WE Jr (2009) The Helopini (Coleoptera: Tenebrionidae) of Virginia. In: Roble SM, Mitchell JC (Eds) A lifetime of contributions to myriapodology and the natural history of Virginia: A Festschrift in honor of Richard L. Hoffman’s 80th birthday. Virginia Museum of Natural History Special Publication No. 16, Martinsville, USA, 331–339.

Tschinkel WR, Doyen JT (1980) Comparative anatomy of the defensive glands, ovipositors and female genital tubes of tenebrionid beetles (Coleoptera). International Journal of Insect Morphology and Embryology 9: 321–368. https://doi.org/10.1016/0020-7322(80)90009-4

Triplehorn CA, Karns KD (2016) The darkling beetles of Ohio. Ohio Biological Survey Bulletin (new series) 18(1), 58 pp.

Wiggins GJ, Grant JF, Lambdin PL (2007) Diversity of darkling beetles (Coleoptera: Tenebrionidae) from Arnold Air Force Base in the barrens of the Eastern Highland Rim, Tennessee. Natural Areas Journal 27: 66–71. https://doi.org/10.3375/0885-8608(2007)27[66:DODBCT]2.0.CO;2
